# Expedient radical phosphonylations *via* ligand to metal charge transfer on bismuth†

**DOI:** 10.1039/d4sc00692e

**Published:** 2024-04-09

**Authors:** Jatin Patra, Akshay M. Nair, Chandra M. R. Volla

**Affiliations:** a Department of Chemistry, Indian Institute of Technology Bombay Powai Mumbai 400076 India Chandra.volla@chem.iitb.ac.in

## Abstract

Bismuth, in spite of its low cost and low toxicity, has found limited application in organic synthesis. Although the photoactivity of Bi(iii) salts has been well studied, this has not been effectively exploited in photocatalysis. To date, only a single report exists for the Bi-based photocatalysis, wherein carbon centered radicals were generated using ligand to metal charge transfer (LMCT) on bismuth. In this regard, expanding the horizon of bismuth LMCT catalysis for the generation of heteroatom centered radicals, we hereby report an efficient radical phosphonylation using BiCl_3_ as the LMCT catalyst. Phosphonyl radicals generated *via* visible-light induced LMCT of BiCl_3_ were subjected to a variety of transformations like alkylation, amination, alkynylation and cascade cyclizations. The catalytic system tolerated a wide range of substrate classes, delivering excellent yields of the scaffolds. The reactions were scalable and required low catalytic loading of bismuth. Detailed mechanistic studies were carried out to probe the reaction mechanism. Diverse radical phosphonylations leading to the formation of sp^3^-C–P, sp^2^-C–P, sp-C–P, and P–N bonds in the current work present the candidacy of bismuth as a versatile photocatalyst for small molecule activation.

## Abstract 2

Herein, we have developed efficient radical phosphonylations tailored upon visible-light mediated LMCT on bismuth. Phosphonylation of a broad range of substrate classes was carried out under mild energy efficient conditions using BiCl_3_ as the catalyst.

## Graphical abstract

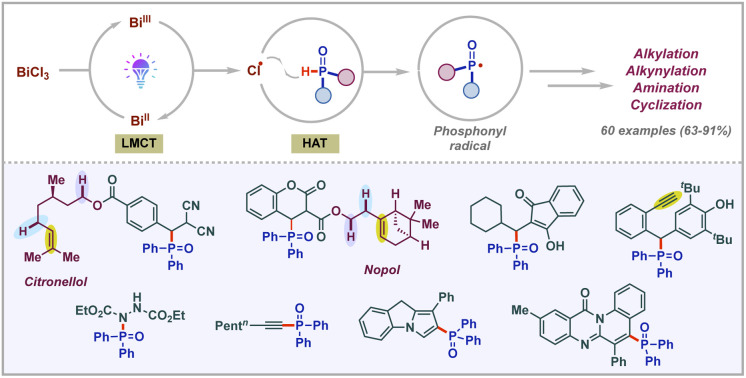

## Introduction

**Scheme 1:**
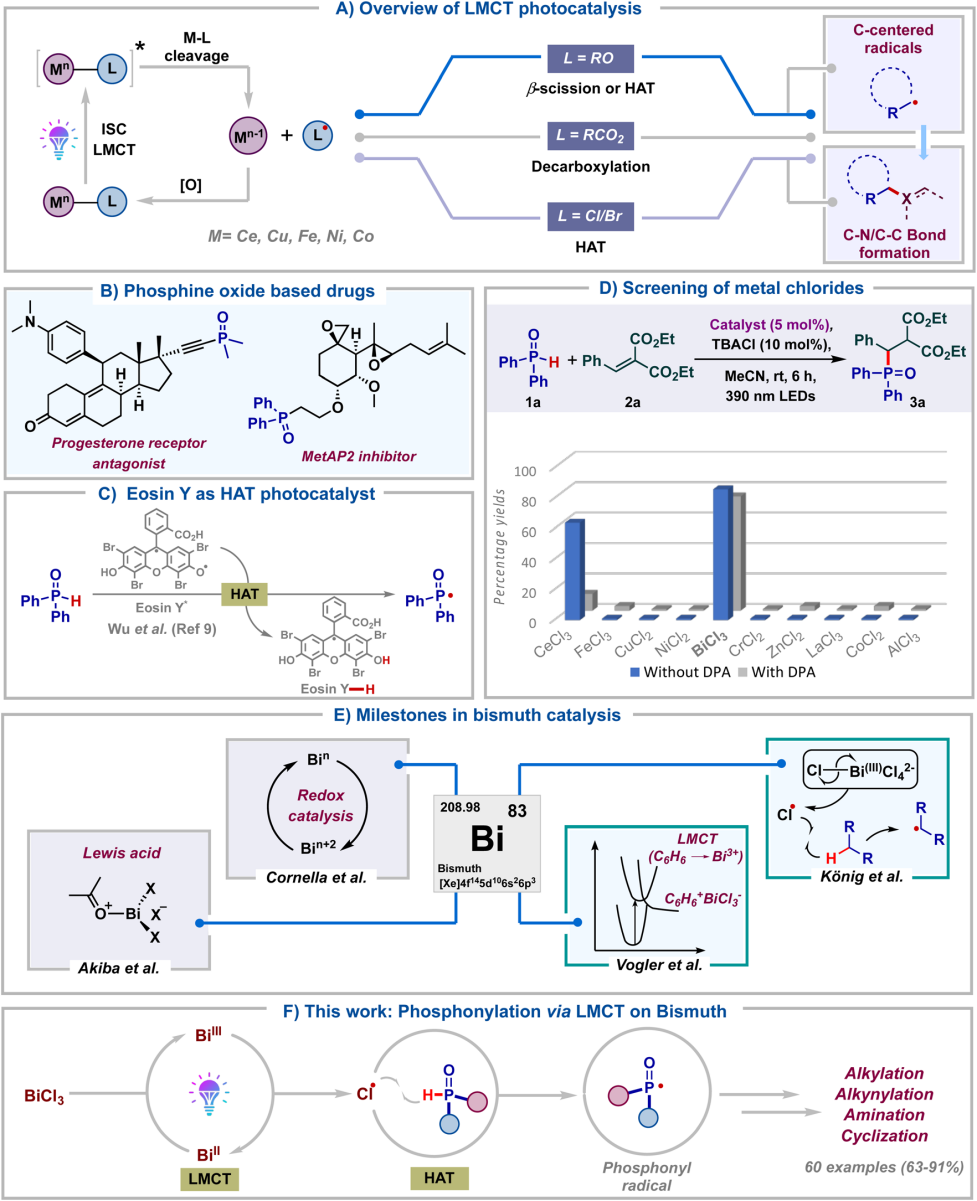
Overview of the work.

Over the past decade, visible light photocatalysis has emerged as one of the most efficient and sustainable platforms to carry out single electron transformations.^1^ These reactions rely on photocatalysts, which upon photoexcitation can act as either single-electron oxidants or reductants. However, the requirement of long-lived excited state half-life of photocatalysts and the prerequisite of an intricate balance of redox potentials between the substrate and photocatalyst make these reactions less general. As a result, visible-light mediated ligand to metal charge transfer (LMCT) using inexpensive metal salts has emerged as a viable alternative towards the generation of electrophilic radicals.^2^ This approach allows the photoinduced homolytic cleavage of metal–ligand bonds, leading to the formation of oxidized ligand radicals and reduced metal complexes (Scheme 1A). Zuo and co-workers in pioneering reports have documented the generation of alkoxy radicals *via* the LMCT of Ce(iv) alkoxide complexes.^3^ At the same time, LMCT of Ce(iv) carboxylate salts was illustrated by König's group to furnish carboxyl radicals.^4^ Subsequently, the last couple of years have seen the application of LMCT of other metal salts to generate halogen radicals.^5^ Notably, in all these protocols, the *in situ* generated alkoxy, carboxyl or halogen radicals typically undergo β-scission, decarboxylation or hydrogen atom transfer (HAT) to generate carbon radicals. Despite these advancements, the formation of heteroatom-centered radicals and their subsequent functionalization using LMCT catalysis remain underexplored.^6^ In particular, though LMCT mediated HAT of C–H bonds has been well documented, the same has not been reported for heteroatom–H (X–H) bonds. Consequently, the development of heteroatom–carbon and heteroatom–heteroatom bond forming reactions tailored upon LMCT is of broad interest.

Organo-phosphorous compounds find significant applications as drugs, agrochemicals and functional materials (Scheme 1B).^7^ Due to their rich synthetic chemistry, they are also valuable building blocks in organic synthesis. Considering these and our long-standing interest in radical chemistry^8^ we embarked on exploring the synthesis of organo-phosphorous compounds utilizing LMCT photocatalysis. We rationalized that the chlorine radicals generated *via* LMCT of metal chlorides could undergo hydrogen atom transfer with *H*-phosphine oxides to furnish phosphonyl radicals. Formation of these radicals *via* direct HAT with Eosin Y* was recently reported by Wu.^9^ Our LMCT protocol could form an elegant alternative to this (Scheme 1C). With this goal, we proceeded to screen various commonly studied metal chlorides in LMCT catalysis for the generation of phosphonyl radicals. Thinking of a reaction which is least likely to fail once the phosphonyl radical is formed *in situ,* Giese type radical addition was chosen as the model transformation. Initially, diphenylphosphine oxide 1a was reacted with alkene 2a in the presence of TBACl (10 mol%) in acetonitrile under purple LED irradiation, employing 5 mol% of different metal chlorides (Scheme 1D). To our surprise, BiCl_3_ was found to be the most optimal catalyst for the generation of phosphonyl radicals, delivering the desired product 3a in 86%. In contrast, the use of CeCl_3_ led to the product in 63% only. Intriguingly, commonly studied salts like FeCl_3_, CuCl_2_ and NiCl_2_ proved ineffective, leading to <5% yields of 3a. Furthermore, other salts like CrCl_2_, ZnCl_2_, LaCl_3_, CoCl_3_ and AlCl_3_ failed to deliver 3a. The addition of 9,10-diphenylanthracene (DPA),^3*d*^ a well-documented electron transfer catalyst, as a redox mediator led to reduced yields with both BiCl_3_ and CeCl_3_. A similar observation was also made employing FeCl_3._ This again ratified that both Ce and Fe are not efficient for the generation of phosphonyl radicals from *H*-phosphine oxides. The comprehensive catalyst screening underscores the remarkable selectivity of BiCl_3_ for the generation of phosphonyl radicals over commonly studied LMCT photocatalysts like CeCl_3_, FeCl_3_, NiCl_2_ and CuCl_2_.

This initial screening highlighted the indispensable role of BiCl_3_ in generating phosphonyl radicals *via* LMCT and we proceeded to study this in detail. Bismuth belongs to group 15 of the periodic table along with other pnictogens and is the heaviest stable element. In spite of its low cost and low toxicity, bismuth has found limited synthetic applications until recently (Scheme 1E).^10^ Bi(iii) salts have been traditionally used as non-redox Lewis acid catalysts for the activation of carbonyls, alcohols and dienes.^11^ The labile nature of Bi(iii)–C bonds has led to the application of organo-bismuth compounds in transmetalation reactions.^12^ Bi(v)–C compounds have found utility as oxidants in organic transformations and they have also been used directly for carrying out oxidative ligand coupling reactions.^13^ Very recently, Cornella and co-workers have engaged in utilizing the superior redox properties of both high and low valent bismuth complexes for the development of novel radical coupling reactions.^14^

On the other hand, photophysical and photoluminescent properties (LMCT) of bismuth complexes were well studied decades ago by Vogler using BiCl_3_ in benzene.^15^ MLCT of bismuth complexes was reported recently by the Marshak group.^16^ In spite of all these, the utility of Bi-salts in photocatalytic transformations remains underexplored and only a single report exists documenting the same. In 2023, König's group in a seminal work tested the ability of various metal chlorides to undergo LMCT.^5*d*^ They found bismuth(iii) chloride as a superior LMCT catalyst for the generation of alkyl radicals *via* chlorine radical mediated HAT of light hydrocarbons. Considering the importance of bismuth catalysis and organo-phosphorous chemistry, our protocol for the generation of heteroatom (P-centered) radicals would open up new avenues in Bi-photocatalysis. In light of all these, we hereby report bismuth chloride as an efficient LMCT catalyst for the generation of phosphonyl radicals from *H*-phosphine oxides (Scheme 1F). Efficient Bi catalyzed phosphonylation of a wide range of substrates like alkenes, coumarin esters, indan-di-ones, *para*-quinone methides, azodicarboxylates, alkynyl bromides and internal alkynes was carried out. Remarkably, we observed the facile formation of sp^3^-C–P, sp^2^-C–P, sp-C–P, and P–N bonds. The reactions delivered excellent product yields using low catalyst loading of inexpensive BiCl_3_ under mild energy efficient conditions, adding significant value from a sustainability point of view. More importantly, our work along with the one by König presents the candidacy of bismuth as a versatile photocatalyst for small molecule activation.

## Results and discussion

**Scheme 2:**
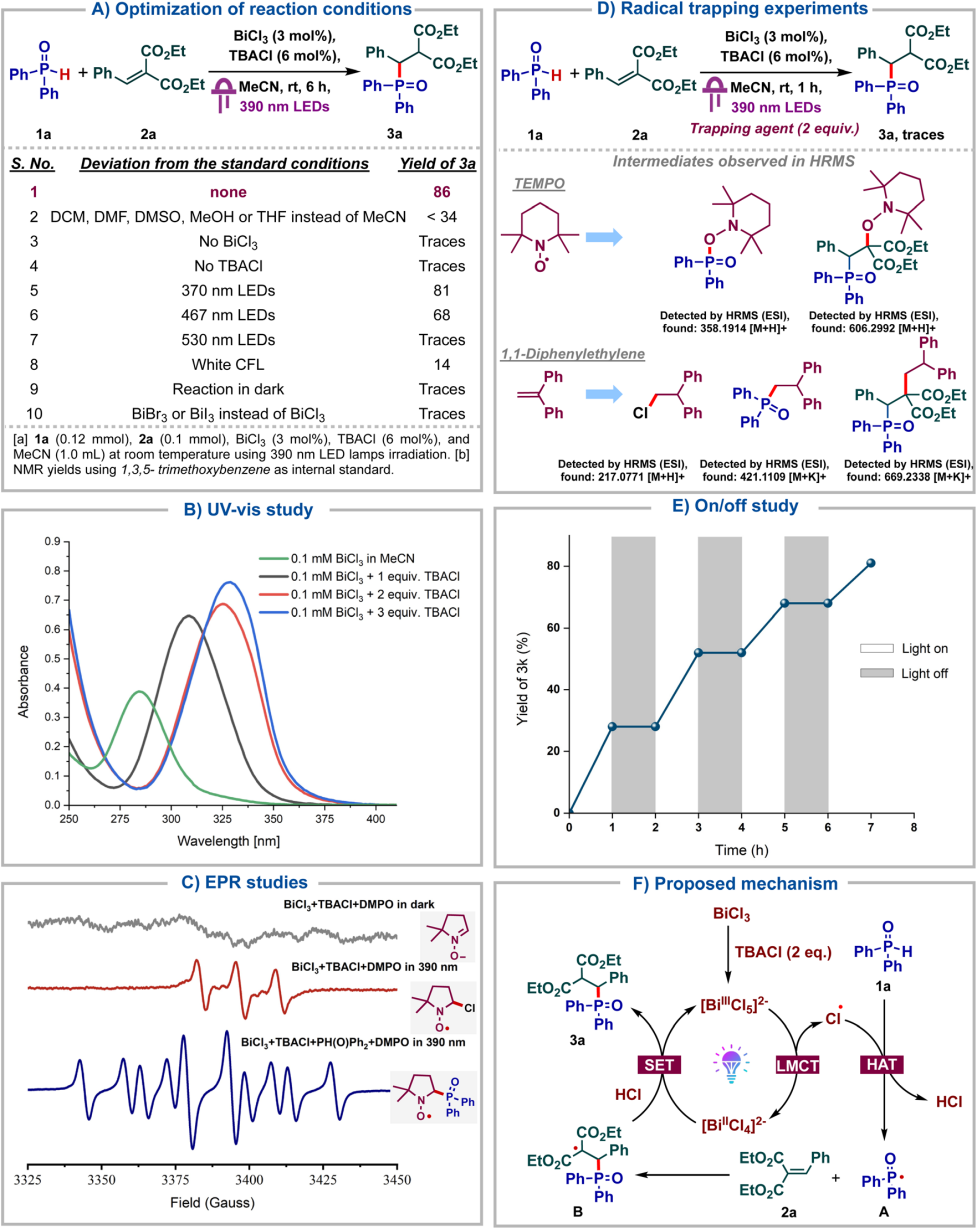
Reaction optimization, mechanistic studies and plausible mechanism.

Having identified bismuth as a viable LMCT catalyst for the generation of phosphonyl radicals, we proceed to further screen the reaction conditions (Scheme 2A). Notably, the reaction failed to proceed in solvents other than MeCN, including DCM, DMF, DMSO or MeOH, highlighting the key role of MeCN as a solvent (entry 2). Both BiCl_3_ and TBACl were deemed indispensable for the reaction as no product was formed in their absence (entries 3–4). Using other wavelengths instead of purple such as blue, green or white was found to be deleterious (entries 5–8). Finally, no product formation was observed in the absence of light (entry 9). The use of BiBr_3_ or BiI_3_ instead of BiCl_3_ was found to be futile, indicating the importance of generating chlorine radicals under LMCT (entry 10). Next, UV-Vis titration of BiCl_3_ with TBACl was carried out wherein, we observed a bathochromic shift upon addition of 1 eq. of TBACl to BiCl_3_, which can be attributed to the formation of [BiCl_4_]^−^ (Scheme 2B). The further addition of 1 more eq. of TBACl again led to a bathochromic shift due to [BiCl_5_]^2−^. No further shift was observed upon the addition of third equivalent of TBACl, indicating that [BiCl_6_]^3−^ is not formed. These observations imply that [BiCl_5_]^2−^ could be the active catalyst and is the preferred coordination mode for bismuth chlorides in the presence of excess chloride ions. As reported by Hunt and co-workers, [BiCl_5_]^2−^ adopts a stable square pyramidal structure.^17^ These UV-Vis studies were consistent with those reported by König's group.^5*d*^

To confirm the generation of phosphonyl radicals under bismuth LMCT catalysis, EPR studies were carried out (Scheme 2C). A mixture comprising the free radical spin trapping agent DMPO (5,5-dimethyl- 1-pyrroline *N*-oxide), BiCl_3_ and TBACl under darkness exhibited no EPR signal. However, upon irradiation with a 390 nm lamp for 10 min, a distinct EPR signal emerged, which could be assigned to a radical adduct of the chlorine radical with DMPO. This indicated that the chlorine radical was generated *via* visible light induced LMCT of BiCl_3_, which underwent rapid trapping by DMPO. Subsequent addition of diphenylphosphine oxide to the same mixture, followed by irradiation at 390 nm for 10 min, led to a distinct EPR signal arising from the adduct of the phosphonyl radical with DMPO. This suggested that the chlorine radicals underwent HAT with diphenylphosphine oxide to form more stable phosphonyl radicals, which then add onto DMPO. Next, we proceeded to carry out radical quenching studies and observed that the reaction failed to proceed in the presence of the radical quenchers such as TEMPO or 1,1-diphenylethylene, indicating that the reaction proceeds *via* stable radical intermediates (Scheme 2D). Furthermore, the adducts corresponding to chloride, phosphonyl and alkyl radicals were observed with TEMPO and diphenylethylene using HRMS analysis confirming them as the intermediates in our reaction. Light on–off studies were carried out wherein the reaction was alternately subjected to 1 h intervals of light irradiation and darkness. We observed that the reaction failed to proceed whenever subjected to darkness (Scheme 2E). Quantum yield (*Φ*) of the transformation was measured to be 0.82 (see ESI†), suggesting a closed photocatalytic cycle and the absence of radical chain propagation. Additionally, direct coordination of the phosphine oxide oxygen with Bi and subsequent LMCT were ruled out, as NMR titrations of BiCl_3_ with 1a showed no interactions (see ESI†). Based on these control studies, we propose the following reaction mechanism (Scheme 2F). Initially, BiCl_3_ reacts with 2 eq. of TBACl in MeCN to form the catalytically active [BiCl_5_]^2−^ species, which upon photoexcitation undergoes LMCT to form the complex [BiCl_4_]^2−^ and the key chlorine radical. This chlorine radical then undergoes hydrogen atom transfer with the *H*-phosphine oxide 1a, forming the phosphonyl radical A and HCl. Radical A then adds onto the alkene 2a to form the alkyl radical B which then undergoes reduction by Bi(ii) species. Subsequent protonation with HCl leads to the product 3a and regenerates the active catalyst.

**Scheme 3:**
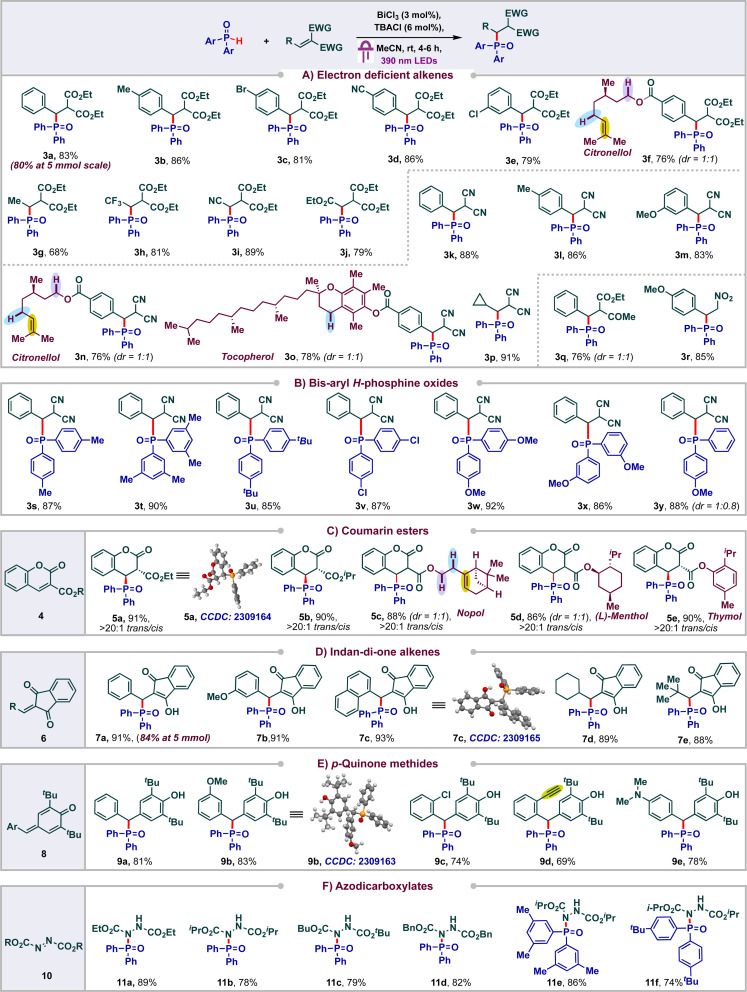
Scope of bismuth catalyzed phosphonylation.

With the optimized conditions, we proceeded to carry out phosphonylation of a variety of electron deficient alkenes (Scheme 3A). Initially, we tested the reactivity of diethyl malonate derived alkenes bearing a variety of substituents. Various functional groups were tolerated on the aromatic ring of the di-ester alkene ranging from electron rich to electron poor, furnishing the corresponding products in good to excellent yields. To our delight, citronellol derived di-ester alkene delivered the product 3f (in 76% yield) selectively wherein the competing HAT of reactive C–H or phosphonylation of the electron rich alkene was not observed.

We found that the aryl ring of the alkene could be replaced with other groups like Me-, CF_3_-, CN-, and CO_2_Et- to afford good yields of the products 3g–3j (68–81%). Next, we proceeded to probe the reactivity of diversely substituted alkenes derived from malononitrile and found that both aryl and alkyl substituents were well tolerated to form the products 3k–3p in appreciable yields (76–91%). Notably, even alkenes conjugated to natural products like citronellol and tocopherol were also amenable and side products arising from the HAT or alkene addition on these natural products were not observed. The keto-ester derived alkene delivered the product 3q in 76% yield in 1 : 1 dr. Phosphonylation of nitroalkene was also amenable, leading to 85% of 3r. Next, we probed the reactivity of various bis aryl *H*-phosphine oxides and found that substituents like Me, *tert*-Bu, Cl, OMe were tolerated on various positions of the phenyl ring, leading to the products 3s–3x in excellent yields (85–92%) (Scheme 3B). Unsymmetric aryl *H*-phosphine oxide delivered the product 3y in 88% in 1 : 0.8 dr.

Coumarins are one of the most abounded classes of natural products and find significant medical applications due to their anti-cancer, anti-bacterial, anti-inflammatory and antioxidant activities.^18^ Hence the synthesis of diversely substituted coumarins is of wide interest. In particular, Michael addition and C–H functionalization of coumarin esters have emerged as an elegant approach to access wide libraries of coumarin derivatives. In spite of these, radical addition to coumarin esters has been less studied and to the best of our knowledge the addition of phosphonyl radicals to these systems has not been reported.^19^ With this in mind, we proceeded to carry out the phosphonylation of coumarin esters 4 (Scheme 3C). To our delight, a variety of coumarin (ethyl or *iso*-propyl) esters underwent facile phosphonylations under our standard conditions to deliver the products 5a and 5b in excellent yields (91% and 90% respectively). Even, coumarins conjugated to natural products such as nopol, menthol and thymol were compatible, leading to the products 5c–5e (86–90%). The structure and observed diastereoselectivity of these products were confirmed by single crystal X-ray diffraction analysis of 5a, wherein the phosphonyl group and the ester group remain *trans* to each other.

Indan-di-one derivatives have found applications as anti-coagulants, anti-inflammatory agents and as psychotropic agents.^20^ They are also valuable precursors in a range of multicomponent reactions.^21^ As a result, the synthesis of diversely substituted indandione derivatives is of significant interest. Michael addition to 2-aryl-indan-1,3-dione derivatives has emerged as the go to approach for accessing these derivatives. In sharp contrast, only a single report documenting radical addition has been reported and the addition of phosphonyl radicals has not been studied.^22^ With this in mind, we proceeded to carry out phosphonylation of various 2-aryl or alkyl-indan-1,3-diones 6 and observed the formation of the corresponding phosphonylated products 7a–7e in excellent yields (Scheme 3D).

To further highlight the versatility and efficiency of our protocol, we proceeded to study the viability of *p*-quinone methides 8 under our standard reaction conditions. Gratifyingly, an array of diversely substituted *p*-quinone methides were found to be amenable, delivering excellent yields of the corresponding phenol derivatives 9a–9e (69–83%) (Scheme 3E). The structures of the products 7c and 9b were unambiguously confirmed using single crystal XRD. Next, we proceed to study the viability of azodicarboxylates in our protocol to achieve P–N bond formation by addition of phosphonyl radicals across the *N*,*N* double bond (Scheme 3F). To our delight, these azodicarboxylates 10 underwent efficient phosphonylation under our standard reaction conditions to deliver good yields of products 11a–11f. The reactions were found to be scalable as the products 3a and 7a were obtained in 80% and 84% yields respectively at 5 mmol scale. On the other hand, substrates such as acrylonitrile, cyclohexanone, methyl acrylate, diethyl phosphite and diphenyl phosphite were found to be unreactive in the current protocol (see ESI†).

**Scheme 4:**
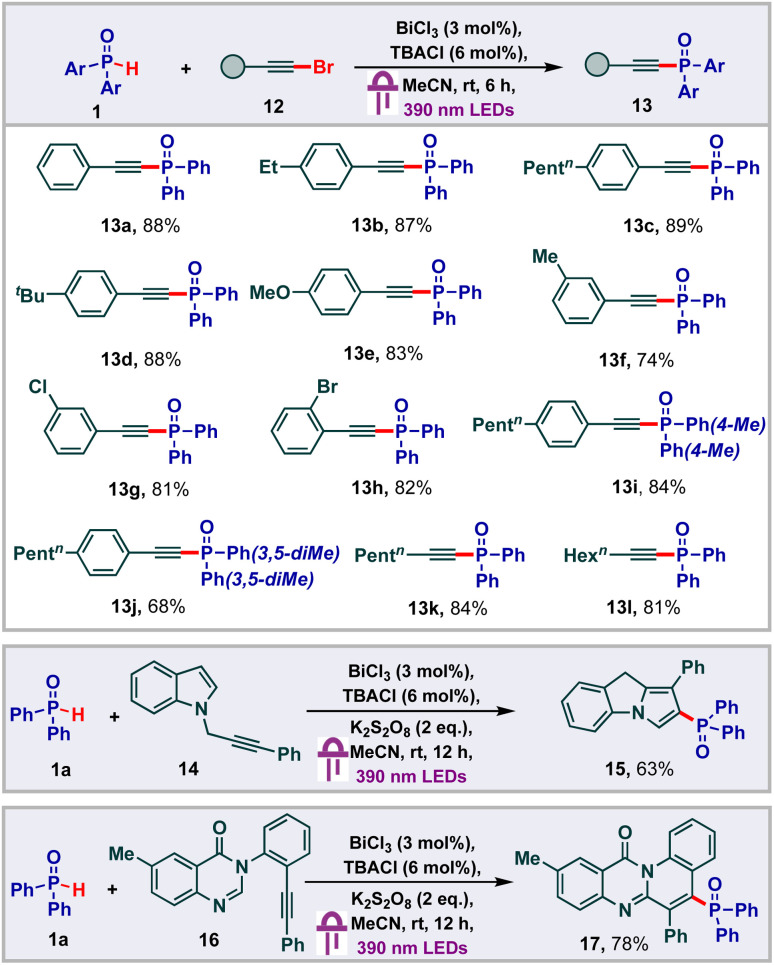
Bismuth catalyzed alkynylation and cascade cyclization.

Having studied addition reactions using *H*-phosphine oxides, we subsequently proceeded to carry out phosphonylation of alkynyl bromides (Scheme 4).^23^ Initially we found that phenylethynyl bromide 12 underwent facile phosphonylation under our standard conditions to deliver the alkynyl phosphonate 13a in 88% yield. With this, we proceeded to probe the substrate scope of this transformation and found that a variety of substituents were tolerated on the phenyl ring of the aryl alkynyl bromides to deliver good yields of the products 13b–13h. Other phosphine oxide derivatives also underwent facile alkynylation to give the products 13i and 13j in 84% and 68% yields respectively. Remarkably, aliphatic alkynes were also found to be viable for the transformation, providing the corresponding products 13l and 13m in 84% and 81% yields respectively. Next, we thought to probe the effectiveness of the bismuth LMCT system to function under oxidative conditions to achieve cascade functionalizations. In this regard, we proceeded to carry out the cyclization of the indole alkyne^24^14 under our standard conditions along with the addition of K_2_S_2_O_8_ as the oxidant. To our delight, the desired 9*H*-pyrrolo[1,2-*a*]indolyl phosphine oxide 15 was isolated in 63% yield. Under similar conditions, we were also able to carry out the cyclization of the quinazolinone alkyne^25^16 to obtain 78% yield of the 12*H*-quinolino[2,1-*b*]quinazolin-12-one 17.

## Conclusions

In conclusion, we have developed efficient radical phosphonylations tailored upon visible-light mediated LMCT on bismuth. The thorough screening of catalysts highlights the unprecedented selectivity of BiCl_3_ for the generation of phosphonyl radicals over commonly studied LMCT photocatalysts like CeCl_3_, FeCl_3_, NiCl_2_ and CuCl_2_. Phosphonylation of a broad range of substrate classes like alkenes, coumarin esters, indan-di-ones, *para*-quinone methides, azodicarboxylates, alkynyl bromides and internal alkynes was carried out under mild energy efficient conditions using cheap, non-toxic BiCl_3_ as the catalyst. The transformations under study delivered the products in good to excellent yields and reactions were found to be scalable. Furthermore, detailed mechanistic studies were carried out to probe the reaction mechanism. More importantly, this forms only the second report on bismuth LMCT photocatalysis and is among the handful of studies documenting the functionalization of heteroatom centered radicals using LCMT catalysis.

## Data availability

Detailed synthetic procedures and complete characterization data for all new compounds can be found in the ESI.†

## Author contributions

JP, AMN, and CMRV designed the project. JP and AMN performed and analysed the experiments. JP, AMN and CMRV wrote the manuscript, CMRV supervised and directed the research. All authors have discussed the results and approved the final version of the manuscript.

## Conflicts of interest

There are no conflicts to declare.

## Supplementary Material

SC-015-D4SC00692E-s001

SC-015-D4SC00692E-s002
