# Supplementary material for: Expedient radical phosphonylations via ligand to metal charge transfer on bismuth
Source: Chem Sci. 2024 Apr 9;15(19):7136–43. doi: 10.1039/d4sc00692e (PMC11095378; doi:10.1039/d4sc00692e)
Supplement: SC-015-D4SC00692E-s001 [file SC-015-D4SC00692E-s001.pdf]

Electronic Supplementary Material (ESI) for Chemical Science.
This journal is © The Royal Society of Chemistry 2024

Expedient Radical Phosphonylations *via* Ligand to Metal Charge Transfer on Bismuth

Jatin Patra, Akshay M. Nair, Chandra M. R. Volla*

Department of Chemistry, Indian Institute of Technology Bombay

Powai, Mumbai-400076, India.

E–mail: chandra.volla@chem.iitb.ac.in

Supporting Information

Table of contents

1	**General considerations and reagent information**	**S2**
2	**General procedures for the synthesis of starting materials**	**S3**
3	**Spectroscopy data for new starting materials**	**S10**
4	**Optimization of the reaction conditions**	**S13**
5	**General synthetic procedures**	**S16**
6	**Failed substrates**	**S18**
7	**Mechanistic investigation**	**S18**
8	**Proposed Mechanisms**	**S26**
9	**Spectroscopic data of final products**	**S28**
10	**References**	**S67**
11	**X-ray Crystallography data for the compound**	**S69**
12	**^{1}H and ^{13}C NMR spectra of the compounds**	**S75**

1. General considerations reagent information.

Unless otherwise stated, all reactions were carried out under air atmosphere in screw cap reaction tubes. All the solvents were bought from Aldrich in sure-seal bottle and were used as received. For column chromatography, silica gel (100–200 mesh) from Finar Co. was used. A gradient elution using petroleum ether and ethyl acetate was performed based on Merck aluminum TLC sheets (silica gel 60F254) that visualized under UV light irradiation (254 nm). Starting materials and reagents were purchased from commercial suppliers (Sigma Aldrich, Alfa Aesar, spectrochem, TCI and BLD pharma.) and were used without further purification. $BiCl_3$ and n-Bu_4NCl were stored and weighed out in glovebox.

a) Analytical information

All isolated compounds are characterized by ^{1}H-NMR, ^{13}C-NMR, and ^{31}P-NMR spectroscopy. In addition, all the compounds are further characterized by HRMS. HRMS spectra were recorded by EI, ESI, FI method. Copies of NMR data can be found in the supporting information. Nuclear magnetic resonance spectra were recorded either on a Bruker 500 or a 400 MHz instrument. All ^{1}H-NMR experiments were reported in units, parts per million (ppm), and were measured relative to the signals for residual chloroform (7.26 ppm) in the deuterated solvent, unless otherwise stated. Abbreviations used for signal multiplicity: ^{1}H-NMR: br = broad, s = singlet, d = doublet, t = triplet, q = quartet, dd = doublet of doublets, ddd = doublet of doublet of doublets, dt = doublet of triplets, and m = multiplet. All ^{13}C-NMR spectra were reported in ppm relative to deuterated chloroform (77.16 ppm), unless otherwise stated, and all were obtained with ^{1}H decoupling. All ^{31}P-NMR were obtained with ^{13}C decoupling.

b) Photoredox setup in batch reactions

In order to set up the photoreactions on 40W LEDs, an 8 ml glass vial is positioned in the middle of a stir plate. To ensure that reaction tubes are equally exposed to the LEDs (at around 4 cm from the light source), two parallel LED lamps (Kessil PR160L 390nm) are mounted perpendicular to the sidewall of reaction tubes. Throughout the reaction, a clip fan (12-Inch fan) on the side of response tubes is always on.

Note: The fan is required to stabilise the reaction temperature (below 35 °C) for repeatable results and to balance the heat produced by the LED light.

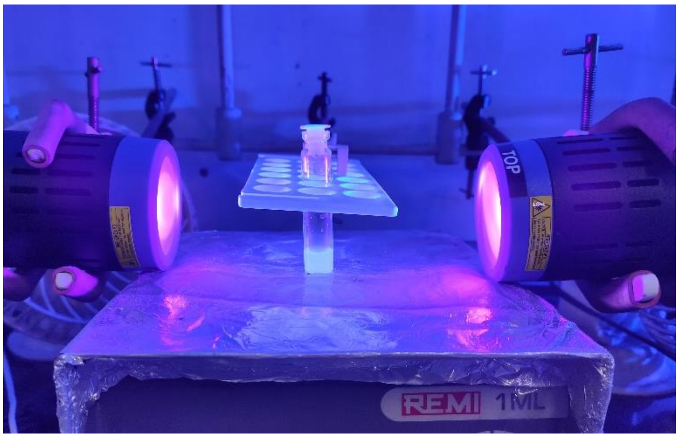

Figure S1. Photochemical reaction setup

2. General procedures for the synthesis of starting materials

Substrates **2g, 2ah- 2ak, 3a, 3b, 3i** were purchased from commercial sources and used as received.

General procedure A

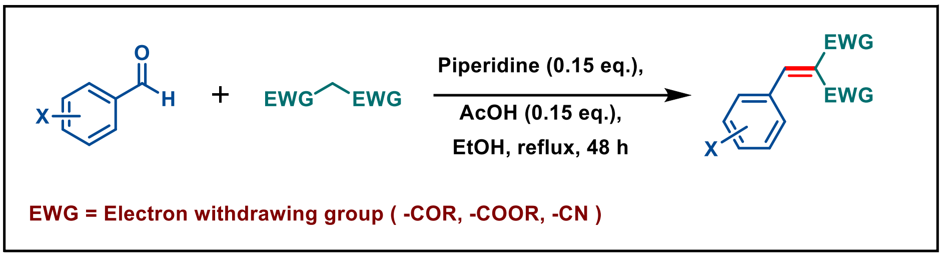

Starting aldehyde (1equiv.), active methylene compound (1.1 equiv.), piperidine (0.15 equiv.), glacial acetic acid (0.15 equiv.) and absolute ethanol (10 mL/mmol of aldehyde) were introduced in a round-bottom flask with a condenser. The mixture is heated at reflux for 48 hours. Then, the solvent is evaporated and a work up with brine and dichloromethane is performed. The residue was either distilled (when liquid) or recrystallized from ethanol (when solid) to obtain the purified activated olefin. NMR spectra and melting points for the thus obtained compounds **2a-2f, 2k-2u** agreed with literature values.

Note: Natural product tethered aldehydes for **2o-2q** were synthesized following the general procedure **E**.

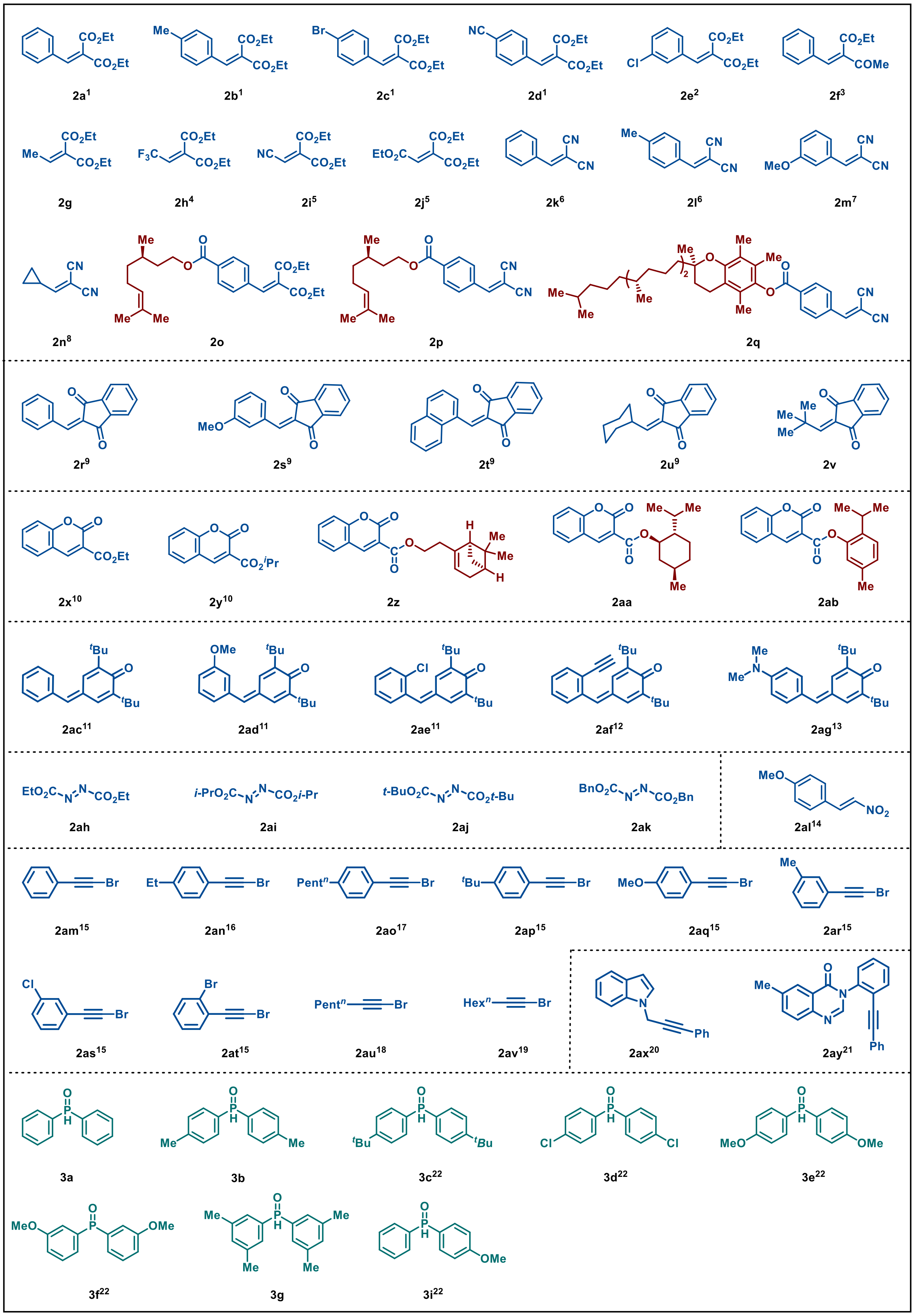

Scheme S1. List of starting materials

General procedure B

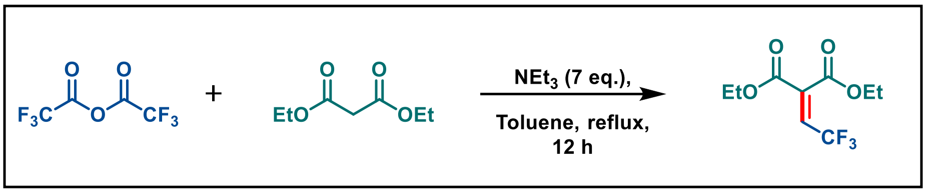

The 1,3-dicarbony substrate (0.50 mmol), trifluoroacetic anhydride ((2.50 mmol, 5.0 equiv.), Et_3N (3.5 mmol, 7.0 equiv.), and toluene (5.0 mL) were added to a reaction tube equipped with a stir bar. The mixture was stirred at 100 °C for 20 h. The reaction mixture was diluted with ethyl acetate (30 mL), washed with saturated brine (30 mL), and water (20 mL), dried over MgSO4. The solvent was removed by rotary evaporation and the resulting product **2h** was purified by column chromatography over silica gel. NMR spectra for obtained compound agreed with literature value.

General procedure C

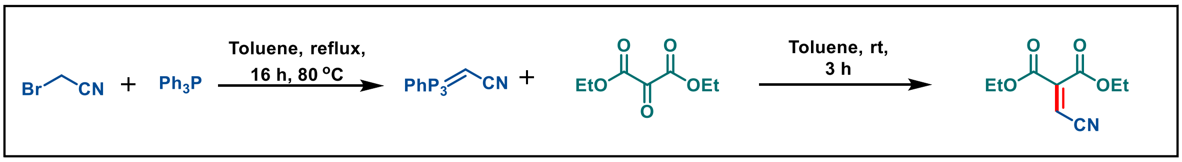

An oven dried round bottom flask equipped with magnetic stir bar was charged with bromoacetonitrile (2.62 g, 21.8 mmol), triphenylphosphine (5.83 g, 22.2 mmol) and toluene (25 mL). The reaction mixture stirred for 16 h at 85 °C. The solvent was evaporated in vacuo, and the residue was then dissolved in CH_2Cl_2 (10 mL). To this solution 2M NaOH (20 mL) was added and the reaction mixture stirred for 3 h. The organic layer was separated, and the aqueous layer was extracted with CH_2Cl_2 (3 × 20 mL). The combined organic layers were washed with brine, and then dried over Na_2SO_4. After removal of all volatiles in vacuo (Triphenylphosphoranylidene)acetonitrile was obtained as a white solid. Without further purification it was added to a round bottom flask containing Diethyl Benzylidenemalonates and toluene (25 mL) and allowed to stir for 3 hours at room temperature to afford product **2i**. NMR spectra for obtained compound agreed with literature value.

General procedure D

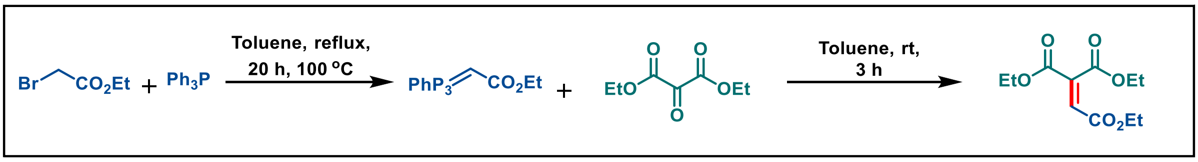

An oven dried round bottom flask equipped with a magnetic stir bar was charged with ethy 2-bromoacetate (1.00 g, 6.00 mmol), triphenylphosphine (1.75 g, 6.67 mmol) and toluene (25 mL). The reaction mixture was heated to 100 °C and stirred for 20 h. The formed precipitate was filtered off and washed with toluene (3 × 15 mL). After all volatiles were removed in vacuo the resulting solid was dissolved in water (10 mL). Subsequently 1M NaOH (20 mL) was added. The reaction mixture was vigorously shaken for 20 min. The formed precipitate was filtered off, washed with diethyl ether (3 × 15 mL) and dried in vacuo to yield ethyl (triphenylphosphoranylidene)acetate as white a solid. Without further purification it was added to a round bottom flask containing diethyl 2,3,4-trioxopentanedioate and toluene (25 mL) and allowed to stir for 3 hours at room temperature to afford product **2j**. NMR spectra for obtained compound agreed with literature value.

General procedure E

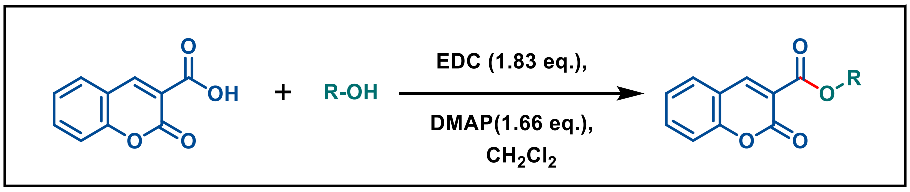

A solution of alcohol, 2-oxo-2H-chromene-3-carboxylic acid (1.1 equiv.), EDC (1.83 equiv. and DMAP (1.66 equiv.) in dry CH_2Cl_2 was stirred at room temperature overnight. The reaction mixture was diluted with 2N HCl and extracted with ethyl acetate. The organic phase was washed with saturated $NaHCO_3$ and brine until neutral, dried Na_2SO_4, and evaporated under vacuum. The residue was chromatographed on silica gel with petroleum ether/ethyl acetate =98/2 as eluent to afford products **2x-2ab**. NMR spectra for obtained compounds agreed with literature value.

General procedure F

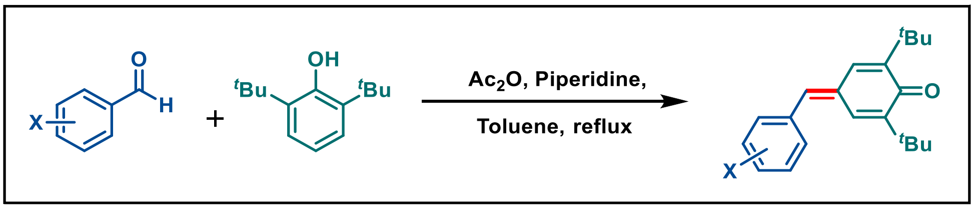

The aldehyde (10 mmol) was added to a solution of the phenol (10 mmol) in toluene (40 mL). The reaction mixture was heated in a Dean-Stark apparatus to reflux. Piperidine (20 mmol) was added dropwise over 1h, and heating was continued until all starting material had been consumed. After the mixture had cooled just below the boiling point of toluene (100 °C), acetic anhydride (20 mmol) was added, and the solution was stirred for 15 min. The residue was extracted three times with dichloromethane. The combined organic layers were washed with water and brine sequentially, dried over magnesium sulphate, filtered, and concentrated. The crude product was purified by flash column chromatography on silica gel to afford the corresponding products **2ac-2ag.** NMR spectra for obtained compounds agreed with literature value.

General procedure G

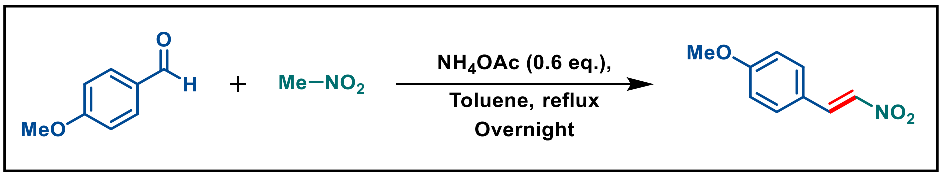

Nitromethane (80 equiv.) was added to a solution of 4-methoxy benzaldehyde and ammonium acetate (0.6 equiv.) in toluene and heated at 100 °C overnight. After the solution had cooled, water was added and the resulting mixture was extracted with ethyl acetate (3 × 20 mL). The organic layer was washed with water (3 × 30 mL), brine (2 × 30 mL) and dried with Na_2SO_4. Solvent was removed under vacuum to give the corresponding β-nitrostyrene **2al**. The crude product was further purified using silica column chromatography using EtOAc: Hexanes (30:70). NMR spectra for obtained compounds agreed with literature value.

General procedure H

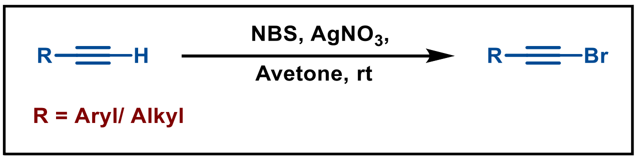

To the mixture of terminal alkyne (10 mmol), NBS (12 mmol) and 30 mL acetone, AgNO3 (10 mol %) was added and the mixture was stirred at room temperature for five hours. After completion of the reaction, the mixture was filtered. And the filtrate was added 30mL of water, extracted with diethyl ether (30 mL x 3), The organic phase was washed with brine, dried over anhydrous MgSO4, filtered, and concentrated under reduced pressure. The residue was purified by column chromatography to give alkynyl bromides **3am-3av**. NMR spectra for obtained compounds agreed with literature value.

General procedure I

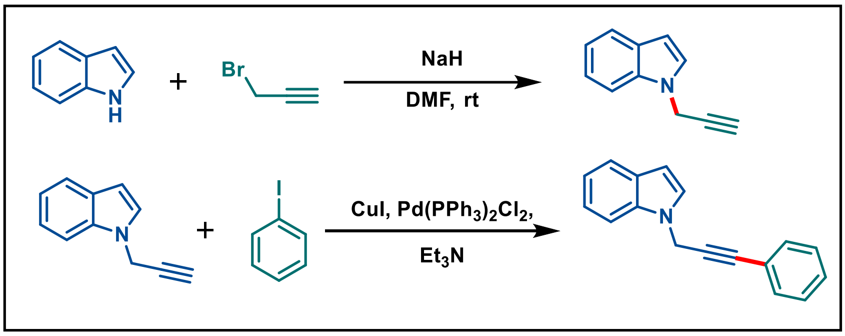

To a solution of the indole (20 mmol) in DMF (50 mL) was added NaH ((40 mmol) slowly at 0 °C. The resulting solution was stirred for 1 h at 0 °C. Then, the propargylic bromide (40 mmol) was added dropwise through a syringe. The reaction mixture was stirred at room temperature for another 2 h. When the reaction was considered complete as determined by TLC analysis, the mixture was quenched with water (40 mL) and extracted with ethyl acetate (3 × 30 mL). The combined organic layers were dried over anhydrous Na_2SO_4, the organic phase was concentrated under reduced pressure and purified by silica gel flash column chromatography (petroleum ether/ ethyl acetate: 30/1).

An oven-dried round bottom flask (250 mL) with a magnetic stir bar was charged with previous product (1.16 g, 6.9 mmol, 1.0 equiv.), triethylamine (45 mL), iodobenzene (1.55 g, 7.6 mmol, 1.1 equiv.), bis(triphenylphosphine)palladium dichloride (96.9 mg, 0.14 mmol, 0.02 equiv.), and copper iodide (13.4 mg, 0.07 mmol, 0.01 equiv.). The reaction mixture is stirred at 40 °C for 6 h. The reaction mixture was filtered, and then diluted with 150 mL of ethyl acetate. The mixture was washed with brine three times. The combined organic phases are dried over anhydrous Na_2SO_4, filtered, and concentrated under vacuum and the crude product was purified by silica gel chromatography using a (petroleum ether/ ethyl acetate: 20/1) mixture as the eluent to give the product **2ax** as a yellowish oil. NMR spectra for obtained compound agreed with literature value.

General procedure J

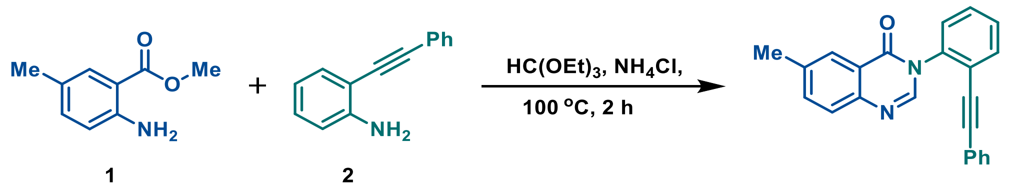

A mixture of **1** (3 mmol, 1 equiv.) and **2** (3.6 mmol, 1.2 equiv.), triethyl orthoformate (4.5 mmol, 1.5 equiv.), and NH4Cl (1.2 mmol, 0.4 equiv.) were heated with stirring at 100 °C in oil bath for 1 h. After cooling, 15 mL H_2O was added and the product was extracted with CH_2Cl_2 (3 x 15 mL). The organic layer was dried with anhydrous Na_2SO_4, and the solvent was evaporated under a vacuum. Crude product was purified by silica gel chromatography using petroleum ether and ethyl acetate as eluent to give the desired product **2ay**. NMR spectra for obtained compound agreed with literature value.

General procedure K

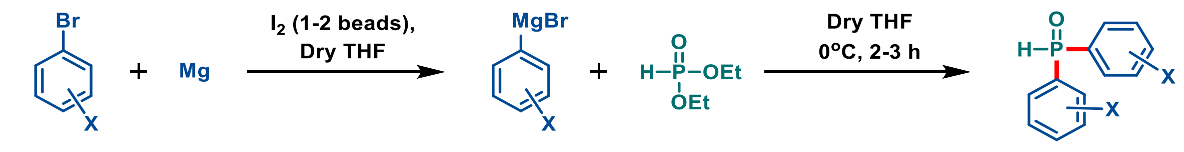

Diethylphosphite (1.29 ml, 10.0 mmol) was added dropwise at 0 °C to a solution of phenyl magnesium bromide in tetrahydrofuran, which was prepared from arylbromides (32.6 mmol) and magnesium (0.95g, 39.6 mmol). The mixture was aged for 15 minutes at 0 °C, then stirred at ambient temperature for two hours. After that, it was cooled again to 0 °C and 75 ml NH_4Cl aqueous was washed with aqueous $NaHCO_3$ and brine, then it was dried over Na_2SO_4. After the solvent had been completely removed, the residue was purified by column chromatography on silica gel to give the products **3c-3g**. NMR spectra for obtained compounds agreed with literature value.

3. Spectroscopy data for new starting materials

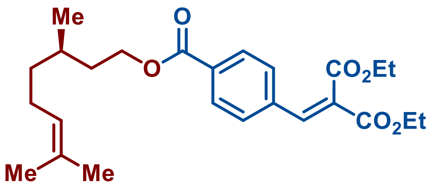

2o

diethyl (R)-2-(4-(((3,7-dimethyloct-6-en-1-yl)oxy)carbonyl)benzylidene)malonate

86% yield, Colourless oil

***R_f*:** 0.80 (hexane/ethyl acetate, 1:9 v/v)

^{1}H NMR (500 MHz, $CDCl_3$) δ 8.02 (d, *J* = 8.5 Hz, 2H), 7.74 (s, 1H), 7.49 (d, *J* = 8.4 Hz, 2H), 5.08 (m, 1H), 4.41 – 4.23 (m, 5H), 1.99 (m, 2H), 1.80 (m, 1H), 1.66 (s, 3H), 1.64 – 1.60 (m, 1H), 1.59 (s, 3H), 1.54 (dd, *J* = 7.8, 6.2 Hz, 1H), 1.43 – 1.35 (m, 1H), 1.33 (t, *J* = 7.2 Hz, 3H), 1.27 (m, 4H), 1.23 – 1.17 (m, 1H), 0.95 (d, *J* = 6.6 Hz, 3H).

^{13}C NMR (126 MHz, $CDCl_3$) δ 166.3, 165.9, 163.8, 140.9, 137.2, 132.0, 131.5, 130.0, 129.3, 128.4, 124.6, 63.9, 62.0, 37.0, 35.5, 29.6, 25.8, 25.5, 19.6, 17.8, 14.2, 14.0.

HRMS (ESI-TOF) m/z: $[M + Na]^+$ Calcd. for $C_{25}H_{34}NaO_6$ 453.2248; Found 453.2224.

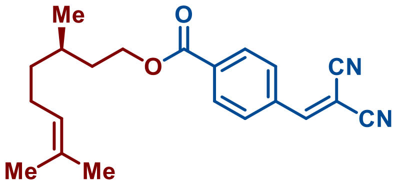

2p

(R)-3,7-dimethyloct-6-en-1-yl 4-(2,2-dicyanovinyl)benzoate

92% yield, White solid

***R_f*:** 0.80 (hexane/ethyl acetate, 1:9 v/v)

^{1}H NMR (500 MHz, $CDCl_3$) δ 8.20 (d, *J* = 8.5 Hz, 2H), 7.99 (d, *J* = 8.5 Hz, 2H), 7.87 (s, 1H), 5.15 – 5.08 (m, 1H), 4.48 – 4.36 (m, 2H), 2.14 – 1.93 (m, 2H), 1.90 – 1.80 (m, 1H), 1.69 (s, 3H), 1.67 – 1.64 (m, 1H), 1.62 (s, 3H), 1.61 – 1.58 (m, 1H), 1.48 – 1.33 (m, 1H), 1.32 – 1.20 (m, 1H), 1.00 (d, *J* = 6.5 Hz, 3H).

^{13}C NMR (126 MHz, CDCl$_3$) δ 165.1, 158.7, 135.4, 134.3, 131.6, 130.6, 130.5, 124.5, 113.33, 112.2, 85.4, 64.4, 37.0, 35.5, 29.6, 25.8, 25.5, 19.6, 17.8.

HRMS (ESI-TOF) m/z: [M + Na]$^+$ Calcd. for $C_{21}H_{24}N_2NaO_2$ 359.1730; Found 359.1730.

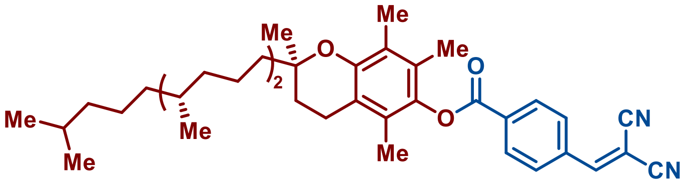

2q

(R)-2,5,7,8-tetramethyl-2-((4R,8R)-4,8,12-trimethyltridecyl) chroman-6-yl 4-(2,2-dicyanovinyl) benzoate

88% yield, White sticky solid

R_f: 0.70 (hexane/ethyl acetate, 1:9 v/v)

^{1}H NMR (400 MHz, CDCl$_3$) δ 8.55 – 8.36 (m, 2H), 8.10 – 7.96 (m, 2H), 7.88 (s, 1H), 2.64 (t, *J* = 6.8 Hz, 2H), 2.14 (s, 3H), 2.07 (s, 3H), 2.04 (s, 3H), 1.84 (m, 2H), 1.56 (m, 2H), 1.48 – 1.39 (m, 4H), 1.34 – 1.26 (m, 9H), 1.21 – 1.08 (m, 7H), 0.89 (dd, *J* = 6.9, 5.5 Hz, 13H).

^{13}C NMR (101 MHz, CDCl$_3$) δ 163.8, 158.6, 149.8, 140.5, 134.9, 134.3, 131.0, 130.6, 126.7, 125.0, 123.4, 117.7, 113.3, 112.1, 85.5, 75.2, 39.4, 37.61, 37.58, 37.51, 37.46, 37.44, 37.40, 37.3, 32.8, 32.8, 32.7, 28.0, 24.9, 24.8, 24.5, 22.8, 22.7, 21.1, 20.7, 19.8, 19.8, 19.7, 19.7, 19.67, 13.1, 12.3, 11.9.

HRMS (ESI-TOF) m/z: [M + K]$^+$ Calcd. for $C_{40}H_{54}KN_2O_3$ 649.3766; Found 649.3729.

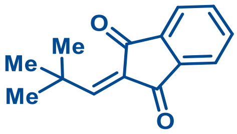

2v

2-(2,2-dimethylpropylidene)-1H-indene-1,3(2H)-dione

71% yield, Colourless oil

R_f: 0.70 (hexane/ethyl acetate, 1:9 v/v)

^{1}H NMR (500 MHz, CDCl$_3$) δ 7.98 (m, 2H), 7.86 – 7.77 (m, 2H), 7.44 (s, 1H), 1.42 (s, 9H).

^{13}C NMR (126 MHz, CDCl$_3$) δ 189.9, 189.0, 164.4, 142.7, 140.2, 135.5, 135.3, 130.9, 123.6, 123.4, 34.6, 29.6.

HRMS (ESI-TOF) m/z: [M + H]$^+$ Calcd. for $C_{14}H_{15}O_2$ 215.1067; Found 215.1054.

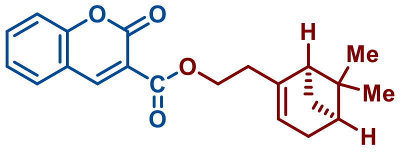

2z

2-((1R,5S)-6,6-dimethylbicyclo [3.1.1] hept-2-en-2-yl) ethyl 2-oxo-2H-chromene-3-carboxylate

85% yield, Light yellow sticky solid

***R$_f$*:** 0.70 (hexane/ethyl acetate, 1:9 v/v)

^{1}H NMR (400 MHz, CDCl$_3$) δ 8.47 (s, 1H), 7.70 – 7.52 (m, 2H), 7.39 – 7.27 (m, 2H), 5.33 (tt, *J* = 3.0, 1.5 Hz, 1H), 4.40 – 4.28 (m, 2H), 2.41 (m, 2H), 2.35 (dt, *J* = 8.5, 5.6 Hz, 1H), 2.28 – 2.13 (m, 2H), 2.11 – 1.99 (m, 2H), 1.24 (s, 3H), 1.14 (d, *J* = 8.6 Hz, 1H), 0.81 (s, 3H).

^{13}C NMR (101 MHz, CDCl$_3$) δ 162.8, 156.6, 155.2, 148.6, 143.8, 134.4, 129.6, 124.9, 119.2, 118.2, 117.9, 116.8, 64.1, 45.7, 40.7, 38.0, 35.9, 31.7, 31.4, 26.3, 21.2.

HRMS (ESI-TOF) m/z: [M + H]$^+$ Calcd. for $C_{21}H_{23}O_4$ 339.1591; Found 339.1569.

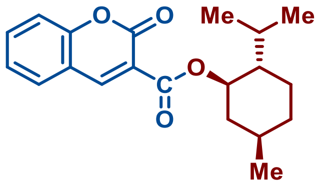

2aa

(1R,2S,5R)-2-isopropyl-5-methylcyclohexyl 2-oxo-2H-chromene-3-carboxylate

94% yield, White solid

***R$_f$*:** 0.75 (hexane/ethyl acetate, 1:9 v/v)

^{1}H NMR (500 MHz, CDCl$_3$) δ 8.42 (s, 1H), 7.62 – 7.51 (m, 2H), 7.28 (dd, *J* = 8.2, 5.9 Hz, 2H), 4.90 (m, 1H), 2.08 (m, 1H), 1.96 (m, 1H), 1.67 (m, 2H), 1.50 (m, 2H), 1.14 – 1.01 (m, 2H), 0.88 (d, *J* = 1.7 Hz, 4H), 0.87 (d, *J* = 2.1 Hz, 4H), 0.75 (d, *J* = 7.0 Hz, 3H).
^{13}C NMR (126 MHz, CDCl$_3$) δ 162.5, 156.6, 155.0, 148.0, 134.2, 129.5, 124.8, 118.7, 117.9, 116.6, 76.0, 46.9, 40.7, 34.1, 31.4, 26.1, 23.3, 22.0, 20.8, 16.2.
HRMS (ESI-TOF) m/z: [M + H]$^+$ Calcd. for $C_{20}H_{25}O_4$ 329.1747; Found 329.1728.

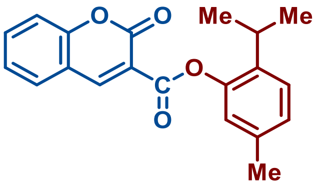

2ab

2-isopropyl-5-methylphenyl 2-oxo-2H-chromene-3-carboxylate

91% yield, White solid

***R$_f$*:** 0.75 (hexane/ethyl acetate, 1:9 v/v)

^{1}H NMR (500 MHz, CDCl$_3$) δ 8.75 (s, 1H), 7.76 – 7.65 (m, 2H), 7.48 – 7.34 (m, 2H), 7.30 – 7.24 (m, 1H), 7.10 (dd, *J* = 8.0, 1.7 Hz, 1H), 6.98 (d, *J* = 1.9 Hz, 1H), 3.14 (hept, *J* = 6.9 Hz, 1H), 2.36 (s, 3H), 1.25 (d, *J* = 6.9 Hz, 6H).
^{13}C NMR (126 MHz, CDCl$_3$) δ 162.1, 156.5, 155.5, 149.9, 147.8, 137.2, 136.8, 134.9, 129.9, 127.6, 126.6, 125.1, 122.7, 117.9, 117.7, 117.0, 27.2, 23.2, 20.9.
HRMS (ESI-TOF) m/z: [M + H]$^+$ Calcd. for $C_{20}H_{19}O_4$ 323.1278; Found 323.1292.

4. Optimization of the reaction conditions

a) Screening of metal catalysts

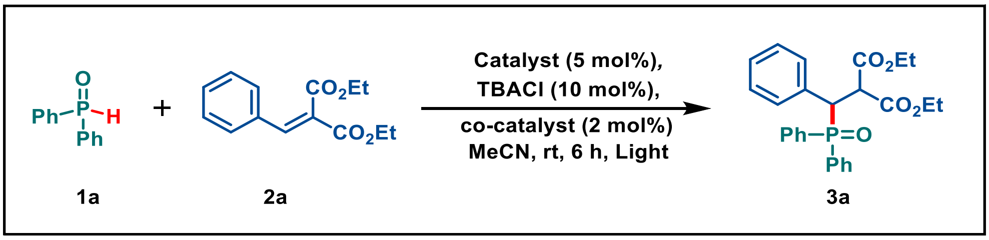

Entry	Catalysts	co-catalyst	Light source	% Yields[b]
1.	$CeCl_3$	-	390 nm	64
2.	$CeCl_3$	DPA	390 nm	11
3.	$CeCl_3$	-	467 nm	43
4.	$FeCl_3$	-	390 nm	NR
5.	$FeCl_3$	DPA	390 nm	Trace
6.	$CuCl_2$	-	390 nm	NR
7.	$CuCl_2$	DPA	390 nm	NR
8.	$NiCl_2$	-	467 nm	NR
9.	$NiCl_2$	DPA	467 nm	NR
10.	$BiCl_3$	-	390 nm	87
11.	$BiCl_3$	DPA	390 nm	75
12.	$CrCl_2$	-	390 nm	NR
13.	$CrCl_2$	DPA	390 nm	NR
14.	$ZnCl_2$	-	467 nm	NR
15.	$ZnCl_2$	DPA	467 nm	Trace
16.	$LaCl_3$	-	390 nm	NR
17.	$LaCl_3$	DPA	390 nm	NR
18.	$CoCl_2$	-	467 nm	NR
19.	$CoCl_2$	DPA	467 nm	Trace
20.	$AlCl_3$	-	390 nm	NR
21.	$AlCl_3$	DPA	390 nm	NR

[a] Reactions were carried out with **1a** (0.12 mmol), **2a** (0.1 mmol), Catalyst (5 mol%), TBACl (10 mol%), and MeCN (1.0 mL) at ambient temperature using 390/ 467 nm LED lamps irradiation. [b] NMR yields using *1,3,5- trimethoxy benzene* as internal standard.

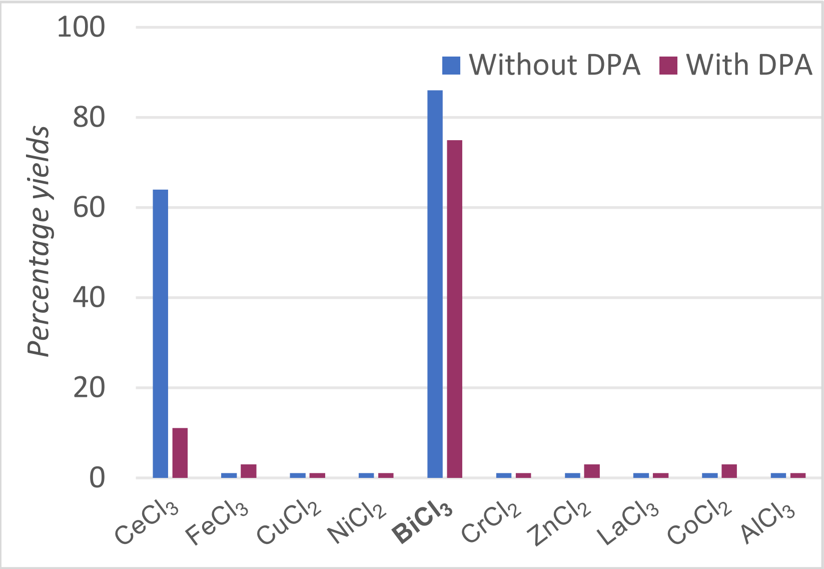

Figure S2. Graphical representation of metal catalyst screening

b) Optimization of reaction conditions *via* bismuth catalyst and solvents screening

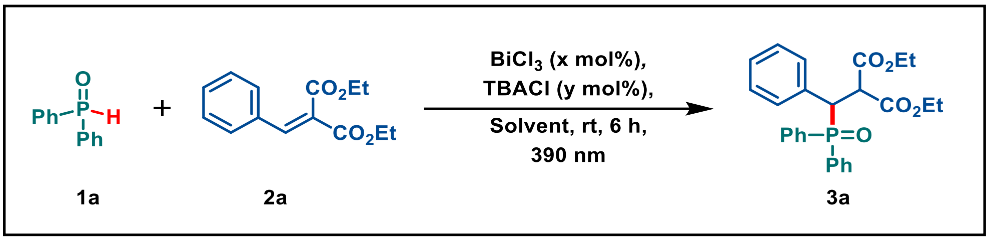

Entry	x	y	Solvent	% Yields[b]
1.	5	10	MeCN	87
2.	5	10	DCM	24
3.	5	10	DMF	34
4.	5	10	DMSO	NR
5.	5	10	MeOH	NR
6.	5	10	THF	NR
7.	5	5	MeCN	73
8.	3	10	MeCN	84
9.	3	6	MeCN	86
10. [c]	3	6	MeCN	81
11. [d]	3	6	MeCN	68
12. [e]	3	6	MeCN	Trace
13. [f]	3	6	MeCN	14

14. [g]	3	6	MeCN	NR
15. [h]	3	6	MeCN	82
16. [i]	-	6	MeCN	Trace
17. [j]	3	-	MeCN	Trace

[a] Reactions were carried out with **1a** (0.12 mmol), **2a** (0.1 mmol), $BiCl_3$ (x mol%), TBACl (y mol%), and solvent (1.0 mL) at ambient temperature using 390 nm LED lamp irradiation [b] NMR yields using *1,3,5- trimethoxy benzene* as internal standard [c] 370 nm LED irradiation [d] 467 nm LED irradiation [e] 540 nm LED irradiation [f] White CFL irradiation [g] No light [h] 0.2 mmol of **1a** [i] No $BiCl_3$ [j] No TBACl.

c) Screening of bismuth precursors

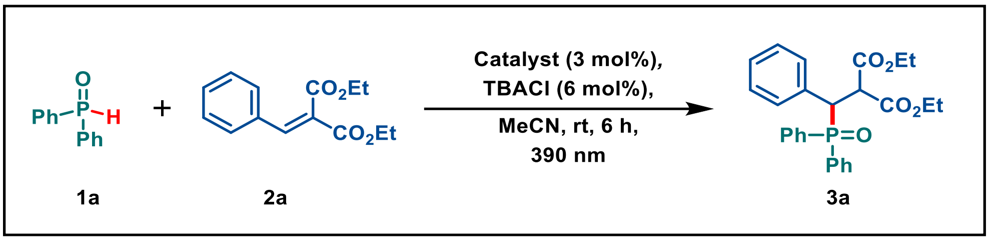

Entry	catalyst	% Yields[b]
**1.**	$BiCl_3$	86
**2.**	$BiBr_3$	Trace
**3.**	BiI_3	Trace

[a] Reactions were carried out with **1a** (0.12 mmol), **2a** (0.1 mmol), $BiCl_3$ (3 mol%), TBACl (6 mol%), and MeCN (1.0 mL) at ambient temperature using 390 nm LED lamp irradiation. [b] NMR yields using *1,3,5- trimethoxy benzene* as internal standard.

5. General synthetic procedures

Procedure for phosphonylation of alkenes

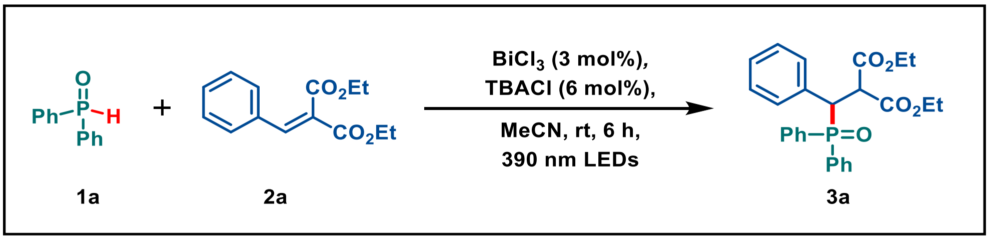

An 8 mL glass vial equipped with a Teflon-coated stirring bar was charged with substrate **1a** (0.12 mmol, 1.2 equiv.), substrate **2a** (0.1 mmol, 1.0 equiv.), $BiCl_3$ (0.003 mmol, 3 mol%) and

TBACl (0.006 mmol, 6 mol%) in glove box. Then 1 mL anhydrous MeCN was added via syringe and flask was irradiated by 390 nm Kessil LED lamps at ambient temperature with a cooling fan. After the consumption of substrate **2a**, the mixture was concentrated in vacuo, the residue was purified by column chromatography on silica gel (eluting with ethyl acetate and petroleum ether) to afford the desired products **3a**.

Procedure for dehalogenative phosphonylation

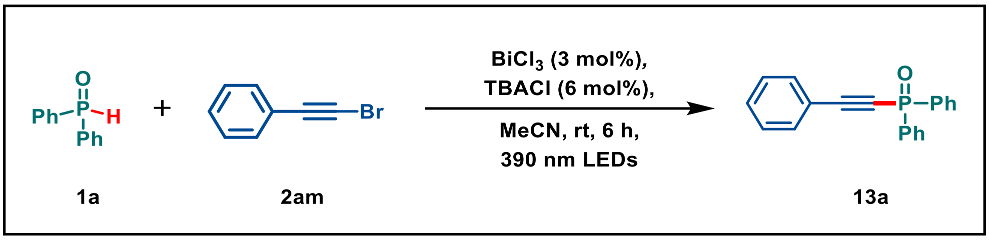

An 8 mL glass vial equipped with a Teflon-coated stirring bar was charged with substrate **1a** (0.12 mmol, 1.2 equiv.), substrate **2am** (0.1 mmol, 1.0 equiv.), $BiCl_3$ (0.003 mmol, 3 mol%) and TBACl (0.006 mmol, 6 mol%) in glove box. Then 1 mL anhydrous MeCN was added via syringe and flask was irradiated by 390 nm Kessil LED lamps at ambient temperature with a cooling fan. After 5-6 hours, the mixture was concentrated in vacuo, the residue was purified by column chromatography on silica gel (eluting with ethyl acetate and petroleum ether) to afford the desired products **13a**.

Procedure for cascade cyclization

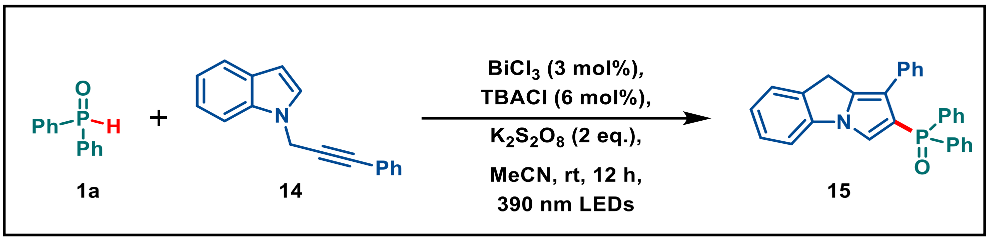

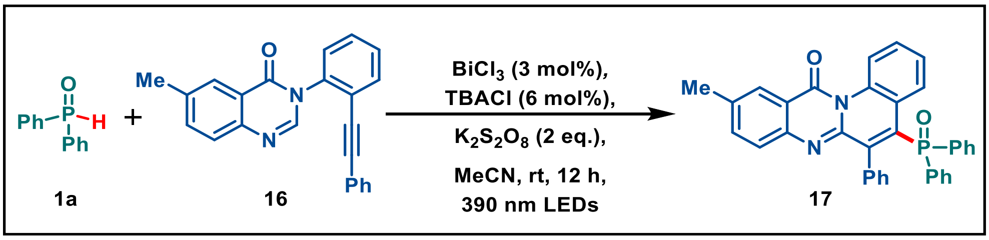

An 8 mL glass vial equipped with a Teflon-coated stirring bar was charged with substrate **1a** (0.3 mmol, 1.5 equiv.), substrate **14** or **16** (0.2 mmol, 1.0 equiv.), $BiCl_3$ (0.006 mmol, 3 mol%), $K_2S_2O_8$ (0.4 mmol, 2 equiv.), and TBACl (0.012 mmol, 6 mol%) in glove box. Then 2 mL anhydrous MeCN was added via syringe and flask was irradiated by 390 nm Kessil LED lamps at ambient temperature with a cooling fan. After 12 hours, the mixture was concentrated in

vacuo, the residue was purified by column chromatography on silica gel (eluting with ethyl acetate and petroleum ether) to afford the desired products **15** or **17**.

6. Failed Substrates

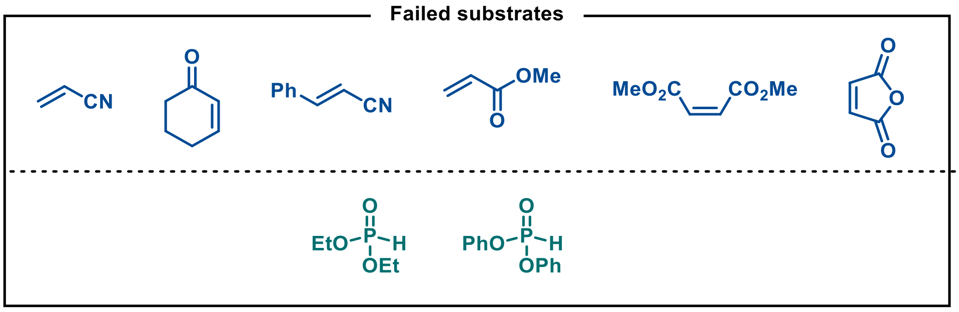

7. Mechanistic investigation

(a) Radical trapping experiments

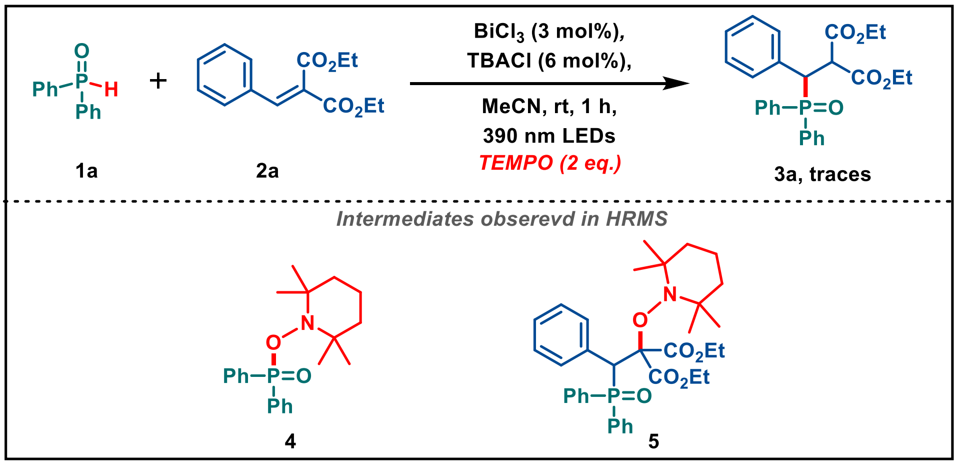

An 8 mL glass vial equipped with a Teflon-coated stirring bar was charged with substrate **1a** (0.12 mmol, 1.2 equiv.), substrate **2a** (0.1 mmol, 1.0 equiv.), $BiCl_3$ (0.003 mmol, 3 mol%), TBACl (0.006 mmol, 6 mol%) and TEMPO (31.3 mg, 0.2 mmol, 2.0 equiv.) in glove box. Then 1 mL anhydrous MeCN was added via syringe and flask was irradiated by 390 nm Kessil LED lamps at ambient temperature with a cooling fan. The reaction mixture was then analyzed by HRMS. The HRMS spectrum showed evidence for the formation of **4** and **5**, which demonstrated the existence of phosphonyl radical and carbon centred radical in this reaction condition.

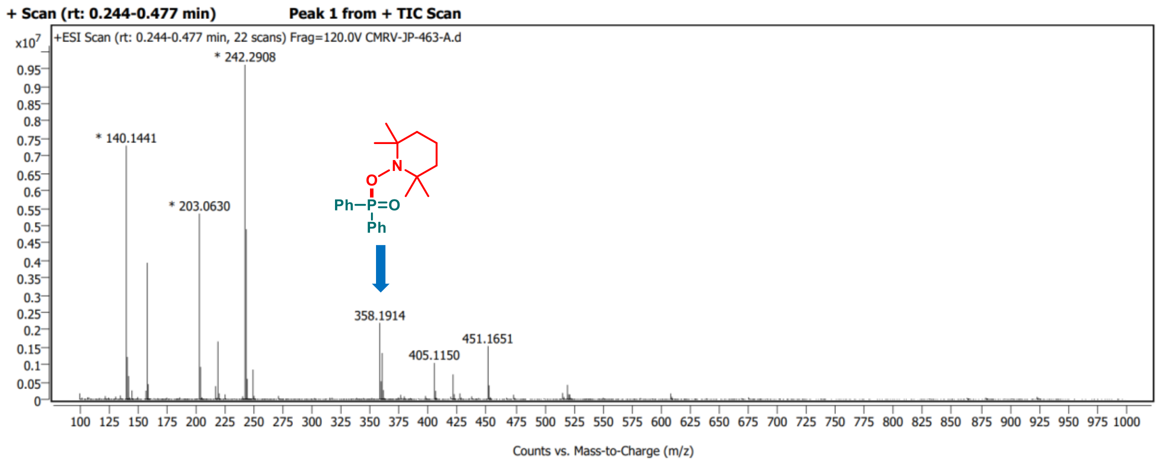

Figure S3. HRMS (ESI) of phosphonyl radical quenching

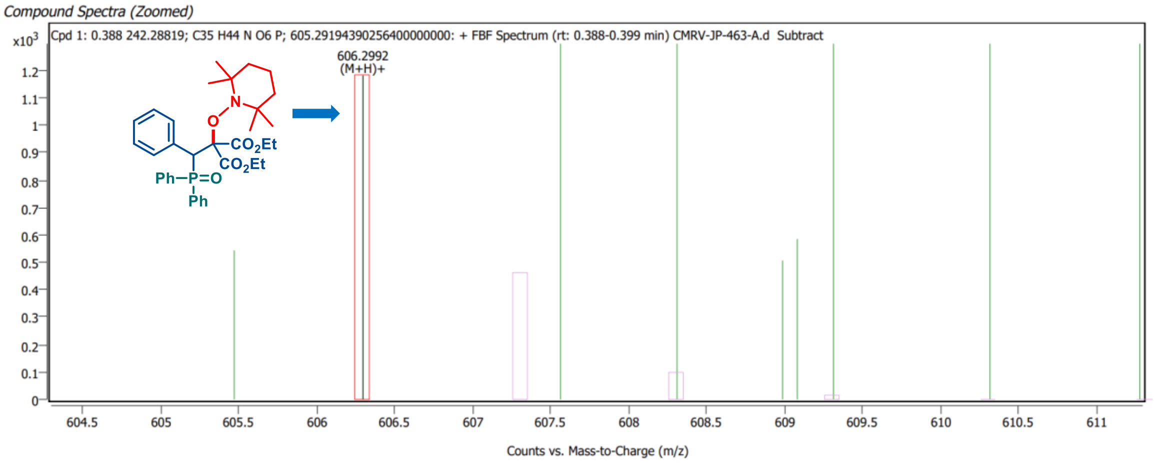

Figure S4. HRMS (ESI) of carbon centred radical quenching

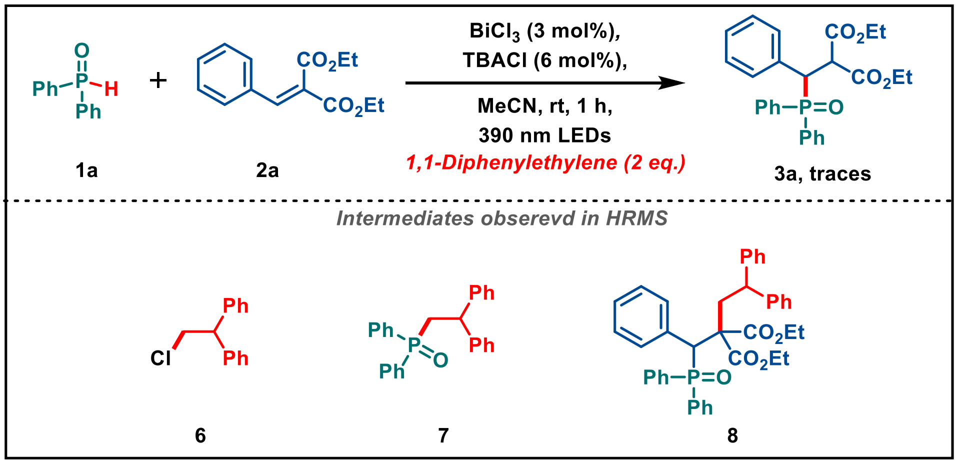

An 8 mL glass vial equipped with a Teflon-coated stirring bar was charged with substrate **1a** (0.12 mmol, 1.2 equiv.), substrate **2a** (0.1 mmol, 1.0 equiv.), $BiCl_3$ (0.003 mmol, 3 mol%), TBACl (0.006 mmol, 6 mol%) and 1,1-Diphenylethylene (36.0 mg, 0.2 mmol, 2.0 equiv.) in glove box. Then 1 mL anhydrous MeCN was added via syringe and flask was

irradiated by 390 nm Kessil LED lamps at ambient temperature with a cooling fan. The reaction mixture was then analyzed by HRMS. The HRMS spectrum showed evidence for the formation of **6, 7** and **8**, which demonstrated the existence of chlorine radical, phosphonyl radical and carbon centred radical in this reaction condition.

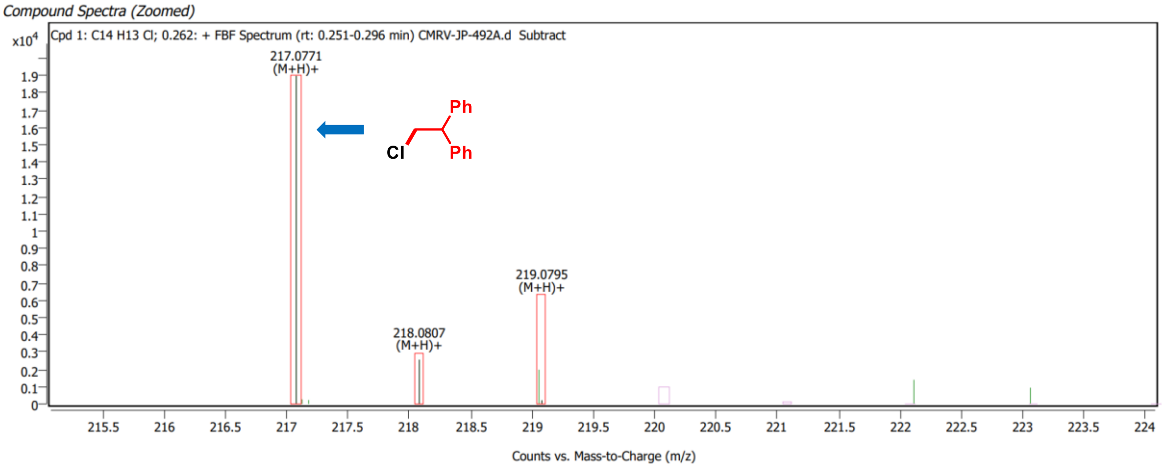

Figure S5. HRMS (ESI) of chlorine radical quenching

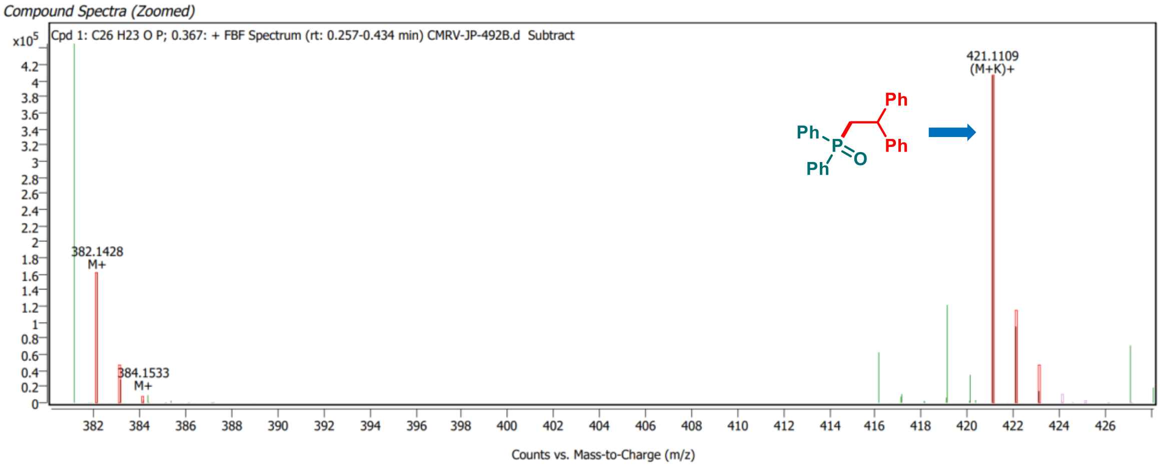

Figure S6. HRMS (ESI) of phosphorus radical quenching

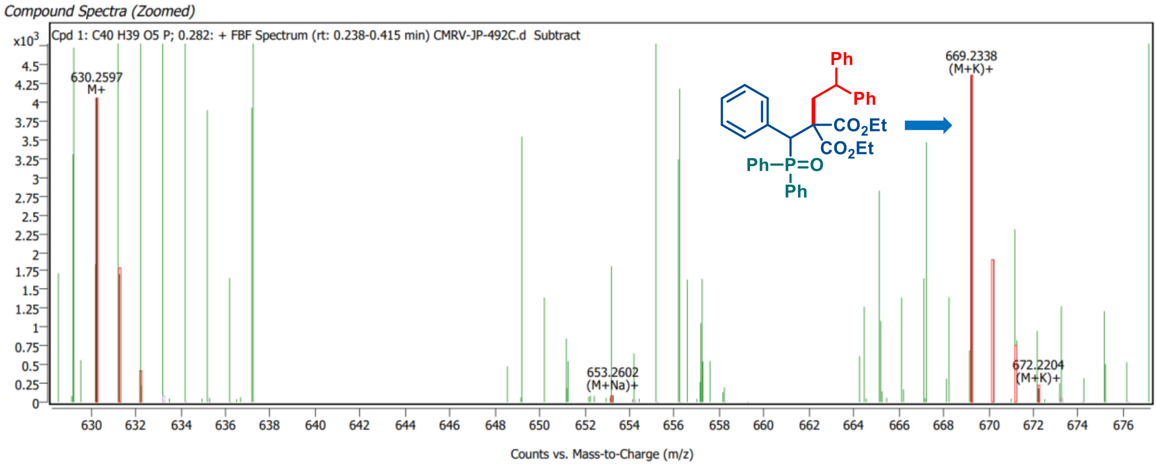

Figure S7. HRMS (ESI) of carbon centred radical quenching

(b) UV-vis experiments

The absorption spectra were measured using a Shimadzu UV 3600i plus spectrophotometer.

For the solutions of Bi(III)Cl_n complexes in $BiCl_3$: TBACl were prepared 1.0×10^{-4} mol/L in MeCN (Figure S8).

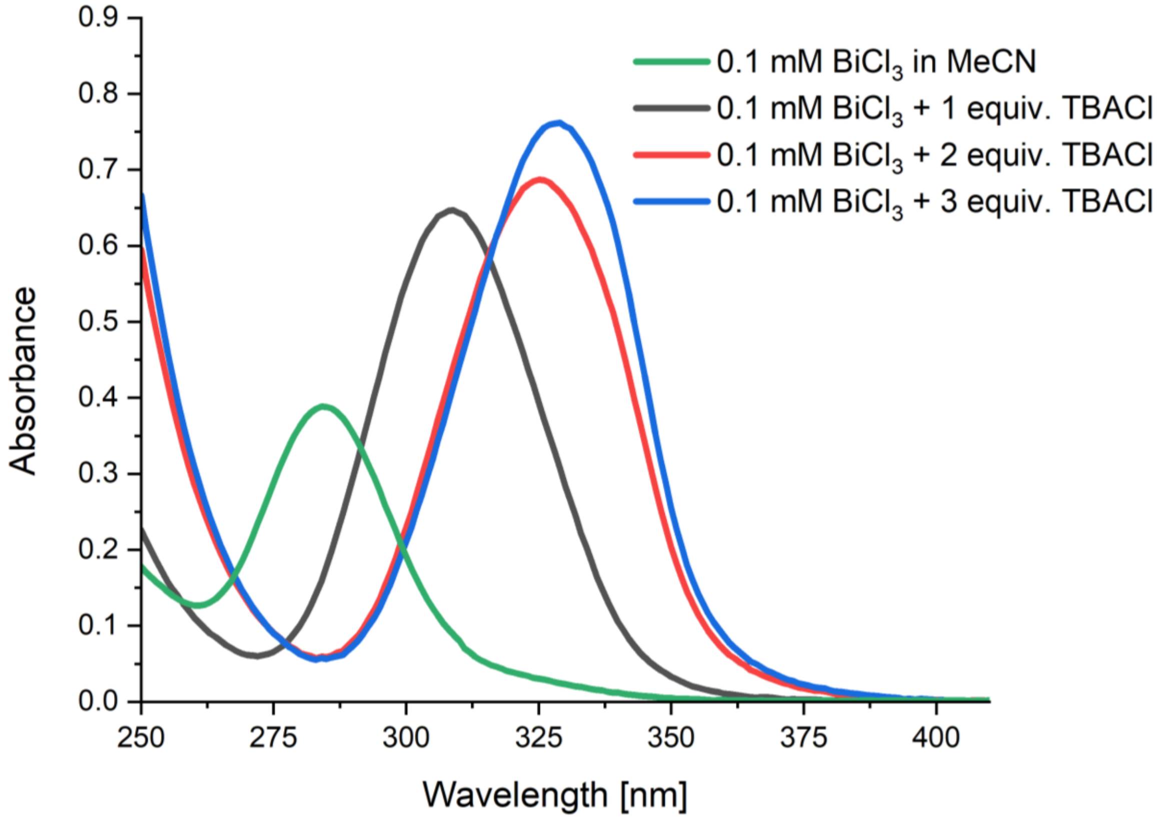

Figure S8. UV-vis absorption

(c) Electron Paramagnetic resonance (EPR) experiment

An 8 mL glass vial equipped with a Teflon-coated stirring bar was charged with substrate $BiCl_3$ (31mg, 0.1 mmol) and TBACl (56 mg, 0.2 mmol) in glove box. Then 2 mL anhydrous MeCN was added via syringe and flask was stirred for 30 mins in dark, followed by addition of DMPO (14mg, 0.12 mmol) and stirred at room temperature for 5 mins in dark. Then, this reaction was taken out by capillary and was analyzed by EPR at room temperature.

Then the same flask was then irradiated by 390 nm Kessil LED lamps at ambient temperature with a cooling fan for 15 mins. Then, this reaction mixture was taken out by capillary and was analyzed by EPR at room temperature.

Another 8 mL glass vial equipped with a Teflon-coated stirring bar was charged with substrate Diphenylphosphine oxide (20mg, 0.1 mmol), $BiCl_3$ (0.003 mmol, 3 mol%) and TBACl (0.006 mmol, 6 mol%) in glove box. Then 2 mL anhydrous MeCN was added via syringe and flask was irradiated by 390 nm Kessil LED lamps at ambient temperature with a cooling fan for 15 mins. Then, this reaction was taken out by capillary and was analyzed by EPR at room temperature.

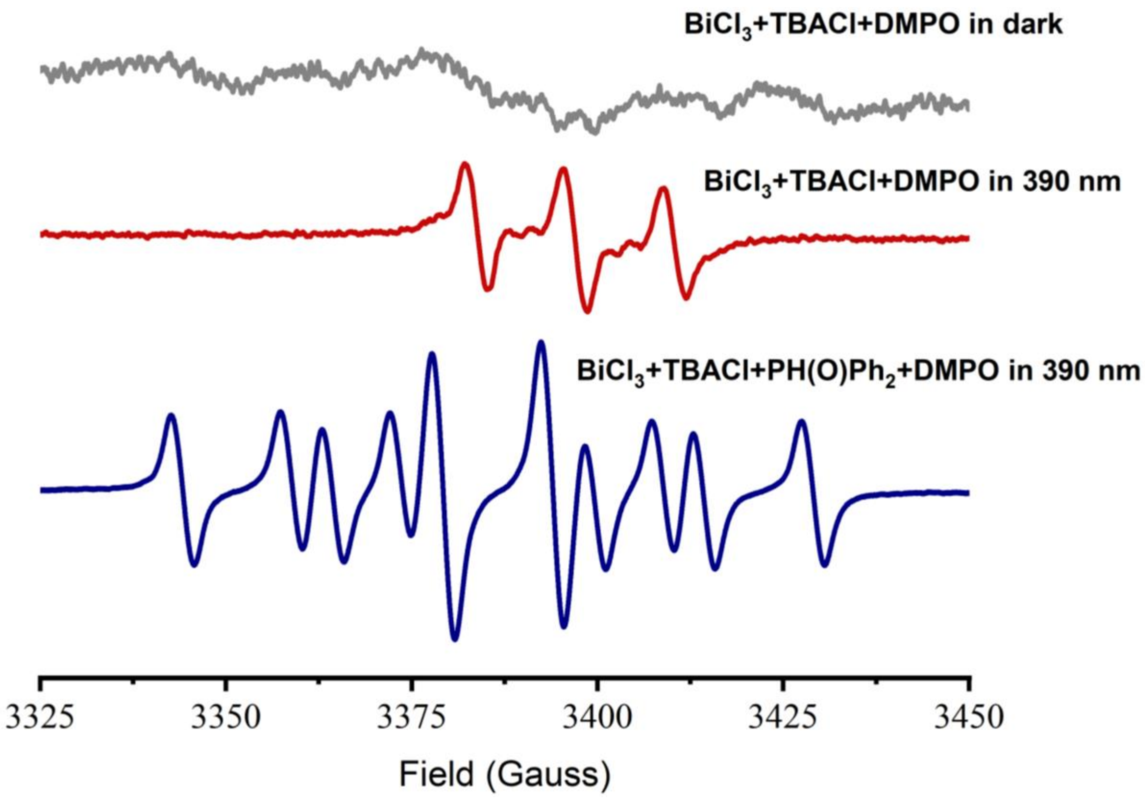

Figure S9. EPR spectrum of chlorine and phosphonyl radical

(d) Light on/off experiment

An 8 mL glass vial equipped with a Teflon-coated stirring bar was charged with substrate **1a** (0.24 mmol, 1.2 equiv.), substrate **2k** (0.2 mmol, 1.0 equiv.), $BiCl_3$ (0.006 mmol, 3 mol%) and TBACl (0.012 mmol, 6 mol%) in glove box. Then 2 mL anhydrous MeCN was added via syringe and flask was irradiated by 390 nm Kessil LED lamps at ambient temperature with a cooling fan. Afterwards, the ^1H NMR was taken to determine the yield

of **3k** with 1,3,5-trimethoxybenzene as an internal standard. The results shown that when the light was switched off, the reaction hardly carried out. These results demonstrated that the reaction might not undergo a radical-chain process.

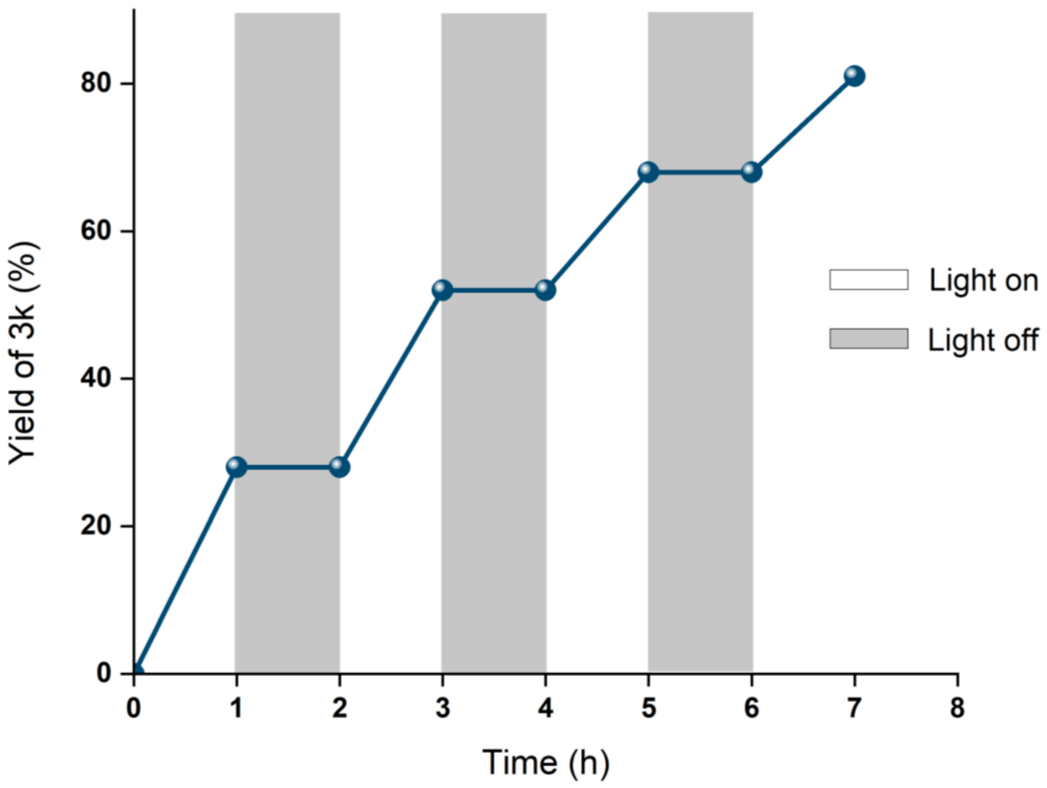

Figure S10. Graphical representation of on/off experiment

(e) Quantum yield measurement

The photon flux was determined by ferrioxalate actinometry similar to a procedure by Yoon[23]. For this purpose, the following two solutions were prepared:

Solution A:

Potassium ferrioxalate hydrate (737 mg, 1.50 mmol) was dissolved in aq. H_2SO_4 (0.05 M, 10 mL) to afford a 0.15 M ferrioxalate solution.

Solution B:

1,10-Phenanthroline monohydrate (25 mg, 0.13 mmol), NaOAc (5.63 g, 68.63 mmol) were dissolved in aq. H_2SO_4 (0.5 M, 25 mL).

Both solutions were stored in dark. First, the photon flux of the 390 nm LED was determined. For this, solution A (2.0 mL) was filled in an 8 mL glass vial and irradiated for 60 s, at λ_{max} = 390 nm. After irradiation, solution B (0.35 mL) was added to the glass vial

and the mixture was stirred in the dark for 1 h to ensure coordination of Fe(II)- ions by phenanthroline. The solution was poured into a quartz cuvette and the absorption of the solution was measured at 510 nm. Sample preparation and measurement were repeated two more times. In a similar way a non-irradiated control sample was prepared, measured for absorbance at 510 nm, which was repeated twice.

The amount of ferrous ion formed was calculated as follows:

$$n(Fe^{+2}) = \frac{V \cdot \Delta A(510nm)}{l \cdot \varepsilon}$$

V refers to the total volume (0.00235 L) of the solution (after addition of solution B), *ΔA* is the difference in absorption of irradiated and non-irradiated samples between at 510 nm, *l* is the path length (1.0 cm) of the cuvette, and *ε* is the molar extinction coefficient of the ferrioxalate actinometer at 510 nm (11100 L mol^{-1} cm^{-1})[24].

The photon flux (Φ_q) was calculated as follows:

$$\phi_q = \frac{n(Fe^{+2})}{\phi_F \cdot t \cdot f}$$

Φ_F refers to the quantum yield for the ferrioxalate actinometer (1.13 at 392 nm)[25], *t* is the irradiation time for solution A (60 s), and *f* is the fraction of light absorbed at λ_{ex} = 390 nm by the ferrioxalate actinometer. This value is calculated using following equation, where *A* is the absorption of the ferrioxalate solution at 390 nm. A measured absorbance value of >3 at 390 nm indicates the fraction of absorbed light to be ≅ 1.

$$f = 1 - 10^{-A}$$

Thus, the average photon flux was calculated to be 1.11×10^{-8} Einsteins s^{-1}.

Determination of the quantum yield:

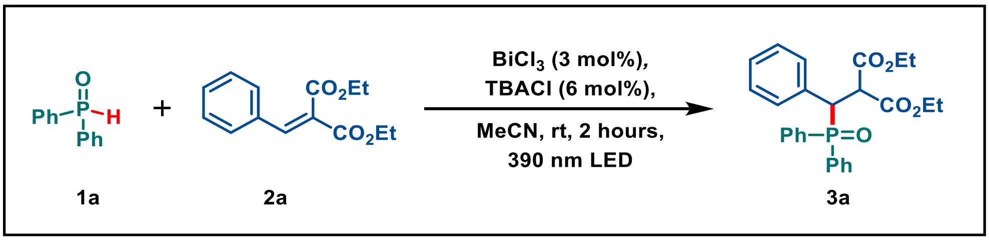

An 8 mL glass vial equipped with a Teflon-coated stirring bar was charged with substrate **1a** (0.12 mmol, 1.2 equiv.), **2a** (0.1 mmol, 1.0 equiv.), $BiCl_3$ (0.003 mmol, 3 mol%) and TBACl (0.006 mmol, 6 mol%) in glove box. Then 1 mL anhydrous MeCN was added via syringe and flask was irradiated by single 390 nm Kessil LED lamp. After 2 hours of

irradiation, the molar number of the product **3a** was determined by using *1,3,5-trimethoxybenzene* as the internal standard. The yield was 56.9% (0.0569 mmol).

The quantum yield (Φ) was calculated as follows:

$$\phi = \frac{n(product)}{\phi_q \cdot t \cdot f_r}$$

where the photon flux (Φ_q) is 1.11×10^{-8} Einsteins s^{-1} (see above), t is the reaction time (2 hours = 7200 s) and f_R is the fraction of light absorbed by the reaction mixture ($1 - 10^{-A} = 1 - 10^{-0.87205} = 0.8657$).

Quantum yield (Φ) was determined to be **0.82**, indicating a closed photocatalytic cycle absence of radical chain propagation pathways.

(f) NMR titration experiment

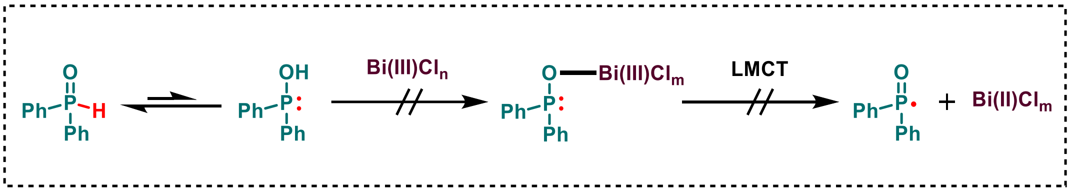

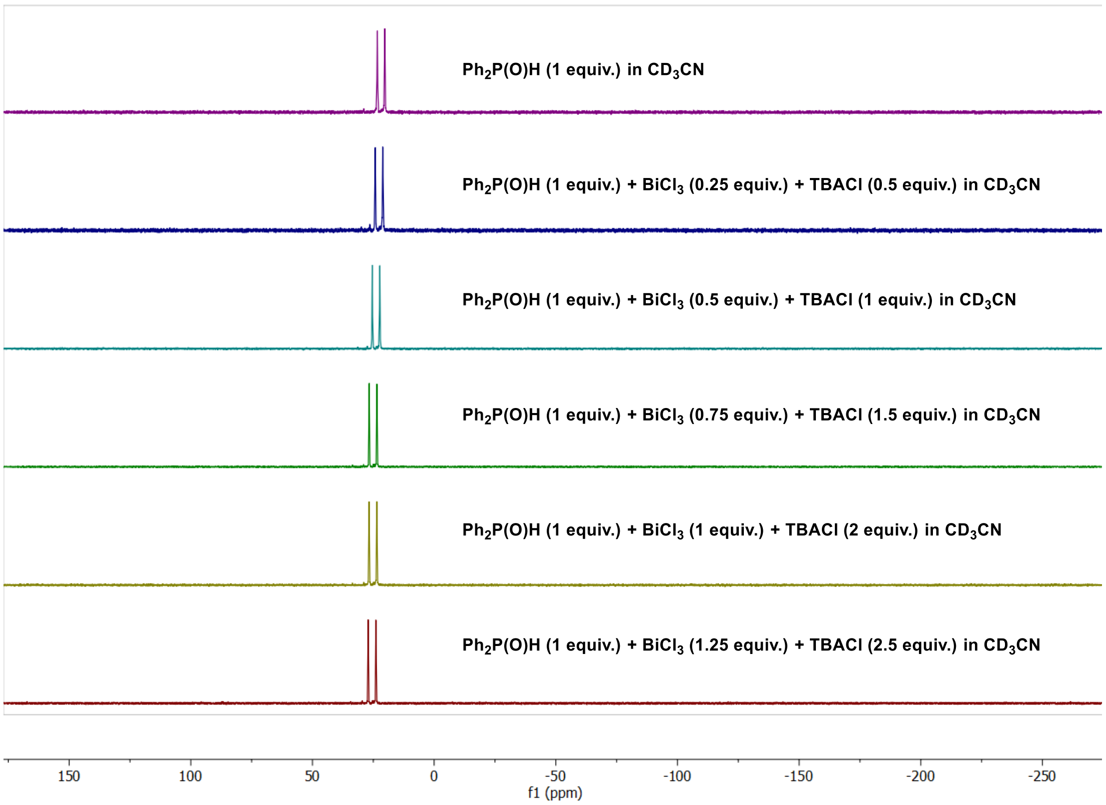

Figure S11. NMR titration with loading of catalyst

To ensure that there is no possibility of the formation of a Bi-O-P complex that results in P-radical *via* homolysis of Bi-O bond, NMR titration of the phosphine oxide **1a** was conducted using increasing amounts of $BiCl_3$ and TBACL. No change in chemical shifts in ^{31}P- NMR of these mixtures indicated the absence of Bi-O-P complex formation.

8. Proposed mechanism

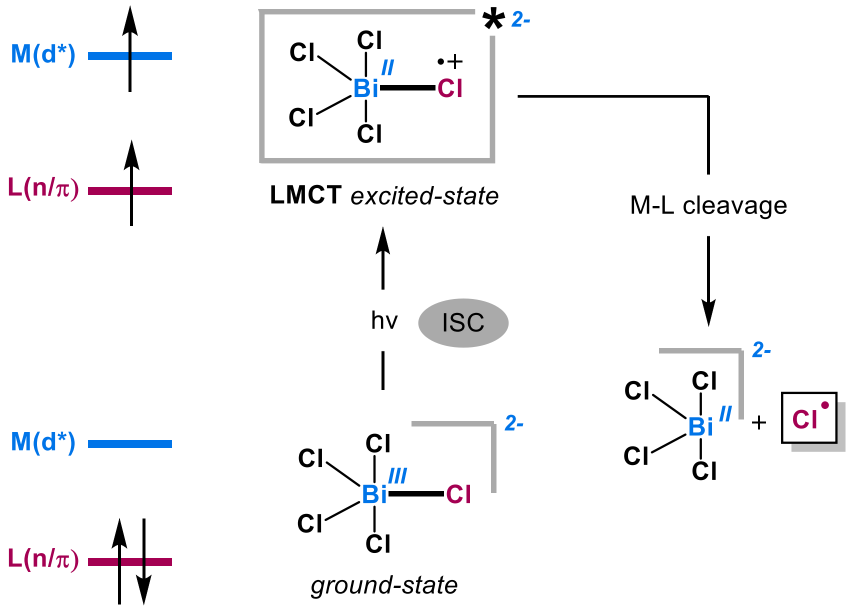

Figure S12. The basic process of LMCT states involving $BiCl_3$

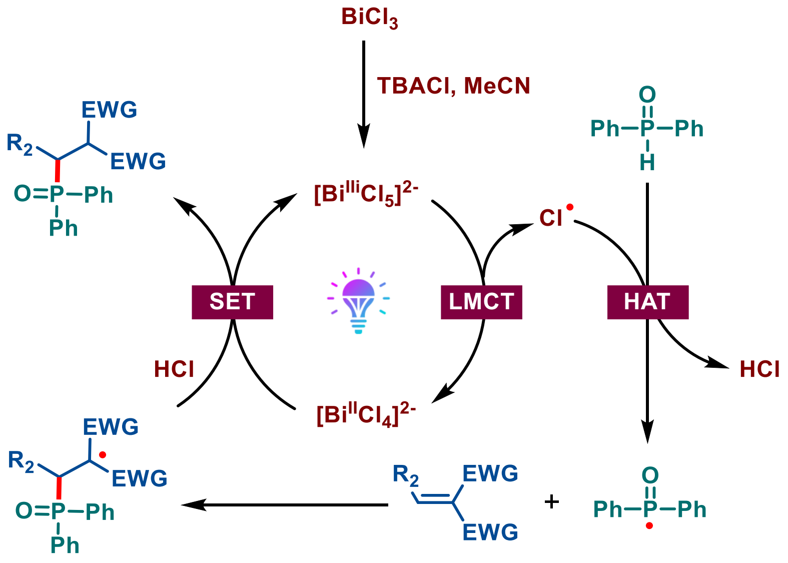

Scheme S2. Proposed mechanism for hydrophophonylation

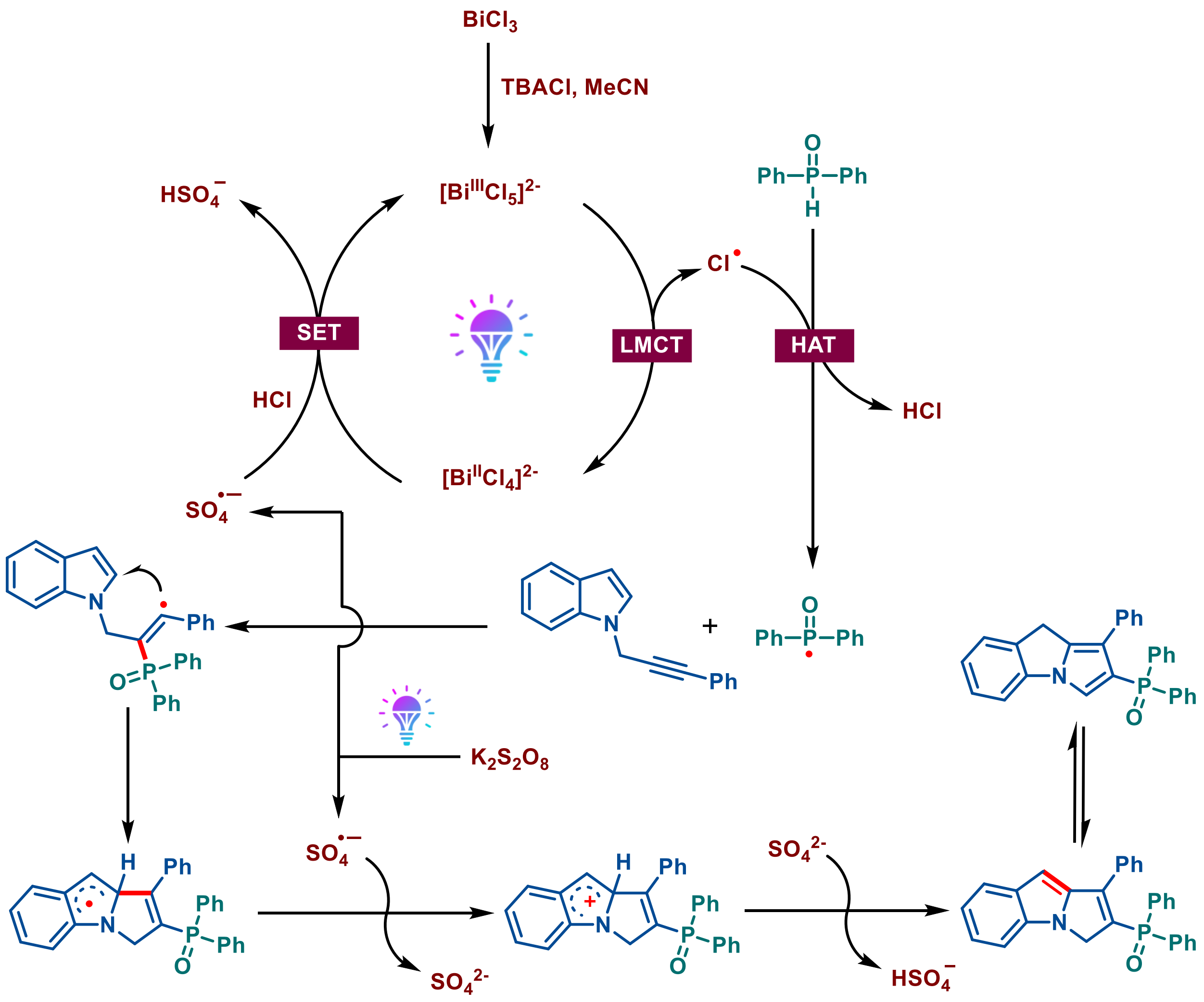

Scheme S3. Proposed mechanism for cascade cyclization of 1-(3-phenylprop-2-yn-1-yl)-1*H*-indole

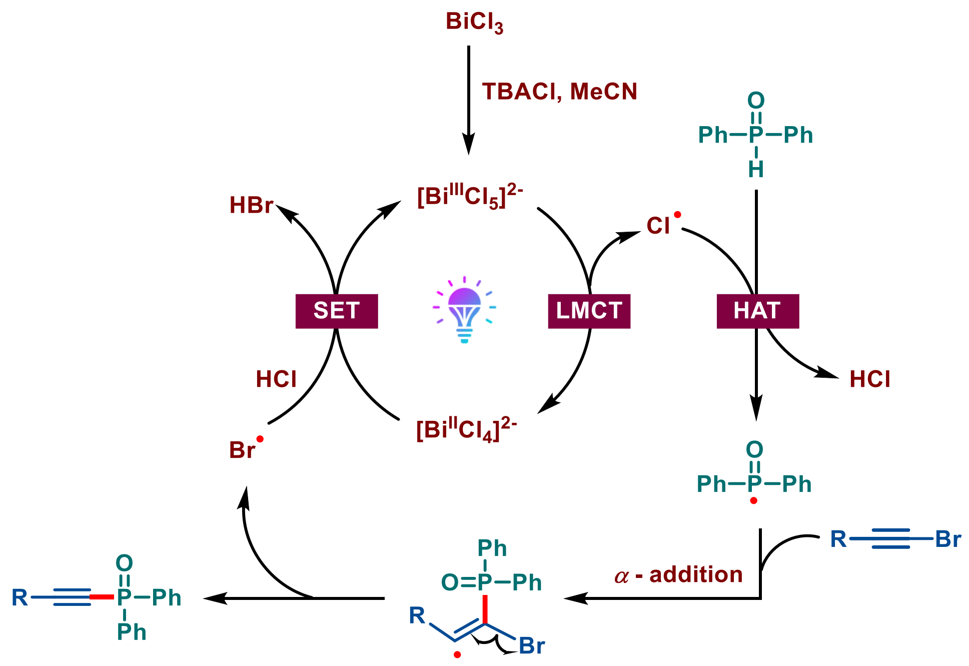

Scheme S4. Proposed mechanism for dehalogenative phosphoalkynylation

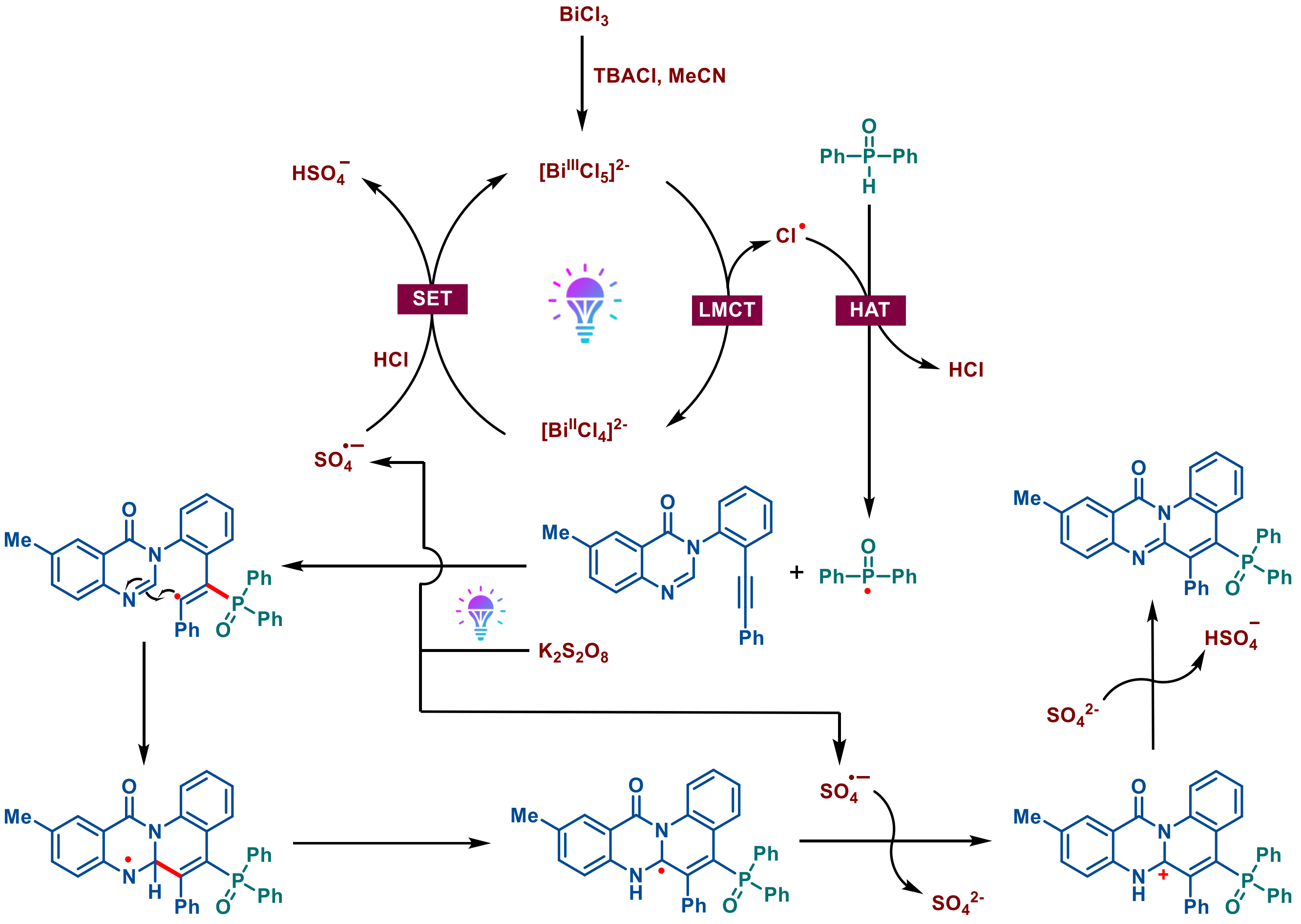

Scheme S5. Proposed mechanism for cascade cyclization of 3-(2-(Ethynyl)phenyl)quinazolinones

9. Spectroscopic data of final products

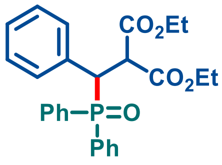

3a

diethyl-2-((diphenylphosphoryl)(phenyl)methyl)malonate

Physical Appearance: White solid (Mp: 138-140 °C)

Yield: 83% (37.3 mg)

***R_f*:** 0.3 (hexane/ethyl acetate, 7:3 v/v)

^{1}H NMR (500 MHz, CDCl$_3$) δ 7.94 (dd, *J* = 12.0, 7.2 Hz, 2H), 7.58 – 7.41 (m, 5H), 7.36 – 7.29 (m, 1H), 7.28 – 7.17 (m, 4H), 7.14 – 7.06 (m, 3H), 4.53 (dd, *J* = 11.2, 6.6 Hz, 1H), 4.45 (t, *J* = 10.8 Hz, 1H), 3.85 – 3.69 (m, 3H), 3.53 (dd, *J* = 10.7, 7.2 Hz, 1H), 1.06 (t, *J* = 7.1 Hz, 3H), 0.84 (t, *J* = 7.1 Hz, 3H).
^{13}C NMR (126 MHz, CDCl$_3$) δ 167.4, 166.9 (d, $J_{c\text{-}p}$ = 15.0 Hz), 133.6 (d, $J_{c\text{-}p}$ = 5.2 Hz), 132.0 (d, $J_{c\text{-}p}$ = 2.8 Hz), 131.9 (d, $J_{c\text{-}p}$ = 9.3 Hz), 131.5 (d, $J_{c\text{-}p}$ = 2.7 Hz), 131.30 (d, $J_{c\text{-}p}$ = 94.8 Hz), 131.27 (d, $J_{c\text{-}p}$ = 8.5 Hz), 130.9 (d, $J_{c\text{-}p}$ = 102.9 Hz), 130.5 (d, $J_{c\text{-}p}$ = 5.3 Hz), 128.4 (d, $J_{c\text{-}p}$ = 11.6 Hz), 128.1 (d, $J_{c\text{-}p}$ = 3.0 Hz), 128.0 (d, $J_{c\text{-}p}$ = 7.1 Hz), 127.4 (d, $J_{c\text{-}p}$ = 2.5 Hz), 61.7 (d, $J_{c\text{-}p}$ = 28.5 Hz), 52.5, 46.2 (d, $J_{c\text{-}p}$ = 65.4 Hz), 13.6 (d, $J_{c\text{-}p}$ = 20.4 Hz).
^{31}P NMR (202 MHz, CDCl$_3$) δ 30.47.
HRMS (ESI-TOF) m/z: $[M + H]^+$ Calcd. for $C_{26}H_{28}O_5P$ 451.1669; Found 451.1635.

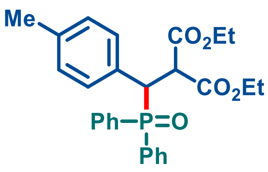

3b

diethyl-2-((diphenylphosphoryl)(p-tolyl)methyl)malonate

Physical Appearance: Light yellow sticky solid

Yield: 86% (39.9 mg)

***R_f*:** 0.3 (hexane/ethyl acetate, 7:3 v/v)

^{1}H NMR (400 MHz, CDCl$_3$) δ 7.95 – 7.86 (m, 2H), 7.57 – 7.42 (m, 5H), 7.36 – 7.30 (m, 1H), 7.27 – 7.20 (m, 2H), 7.11 – 7.05 (m, 2H), 6.89 (d, *J* = 7.8 Hz, 2H), 4.50 (dd, *J* = 11.2, 6.8 Hz, 1H), 4.43 – 4.36 (m, 1H), 3.84 – 3.68 (m, 3H), 3.53 (m, 1H), 2.18 (s, 3H), 1.06 (t, *J* = 7.1 Hz, 3H), 0.86 (t, *J* = 7.1 Hz, 3H).
^{13}C NMR (101 MHz, CDCl$_3$) δ 167.6, 167.0 (d, $J_{c\text{-}p}$ = 15.1 Hz), 137.1 (d, $J_{c\text{-}p}$ = 2.7 Hz), 132.04 (d, $J_{c\text{-}p}$ = 14.5 Hz), 131.98 (d, $J_{c\text{-}p}$ = 9.0 Hz), 131.5 (d, $J_{c\text{-}p}$ = 2.8 Hz), 131.4 (d, $J_{c\text{-}p}$ = 8.5 Hz), 131.1 (d, $J_{c\text{-}p}$ = 101.5 Hz), 130.8 (d, $J_{c\text{-}p}$ = 80.9 Hz), 130.5 (d, $J_{c\text{-}p}$ = 5.3 Hz), 130.4, 128.8 (d, $J_{c\text{-}p}$ = 2.0 Hz), 128.4 (d, $J_{c\text{-}p}$ = 11.6 Hz), 128.1 (d, $J_{c\text{-}p}$ = 11.9 Hz), 61.7 (d, $J_{c\text{-}p}$ = 22.2 Hz), 52.6, 45.8 (d, $J_{c\text{-}p}$ = 65.8 Hz), 21.1, 13.7 (d, $J_{c\text{-}p}$ = 14.5 Hz).
^{31}P NMR (162 MHz, CDCl$_3$) δ 30.42.
HRMS (ESI-TOF) m/z: $[M + K]^+$ Calcd. for $C_{27}H_{29}KO_5P$ 503.1384; Found 503.1366.

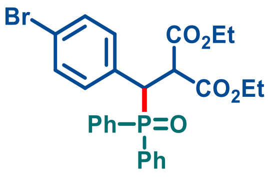

3c

diethyl-2-((4-bromophenyl)(diphenylphosphoryl)methyl)malonate

Physical Appearance: White sticky solid

Yield: 81% (42.7 mg)

***R_f*:** 0.3 (hexane/ethyl acetate, 7:3 v/v)

^{1}H NMR (400 MHz, CDCl$_3$) δ 8.05 – 7.86 (m, 2H), 7.64 – 7.43 (m, 5H), 7.39 – 7.31 (m, 1H), 7.30 – 7.19 (m, 4H), 7.12 (dd, J = 8.5, 2.0 Hz, 2H), 4.48 (dd, J = 11.0, 6.5 Hz, 1H), 4.38 (t, J = 10.7 Hz, 1H), 3.81 (m, 2H), 3.71 (m, 1H), 3.49 (m, 1H), 1.04 (t, J = 7.2 Hz, 3H), 0.90 (t, J = 7.1 Hz, 3H).

^{13}C NMR (101 MHz, CDCl$_3$) δ 167.3 (d, J_{c-p} = 1.5 Hz), 166.8 (d, J_{c-p} = 14.6 Hz), 132.9 (d, J_{c-p} = 5.3 Hz), 132.3 (d, J_{c-p} = 5.5 Hz), 132.2 (d, J_{c-p} = 2.8 Hz), 132.0 (d, J_{c-p} = 9.2 Hz), 131.8 (d, J_{c-p} = 2.7 Hz), 131.2, 131.2 (d, J_{c-p} = 6.3 Hz), 131.1 (d, J_{c-p} = 95.1 Hz), 130.8 (d, J_{c-p} = 101.9 Hz), 128.5 (d, J_{c-p} = 11.8 Hz), 128.3 (d, J_{c-p} = 11.9 Hz), 121.7 (d, J_{c-p} = 3.0 Hz), 61.9 (d, J_{c-p} = 14.6 Hz), 52.3, 45.6 (d, J_{c-p} = 64.7 Hz), 13.7 (d, J_{c-p} = 5.9 Hz).

^{31}P NMR (162 MHz, CDCl$_3$) δ 29.79.

HRMS (ESI-TOF) m/z: [M + H]$^+$ Calcd. for $C_{26}H_{25}BrO_5P$ 529.0774; Found 529.0772.

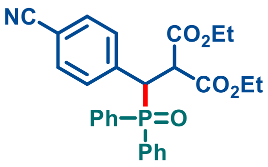

3d

diethyl-2-((4-cyanophenyl)(diphenylphosphoryl)methyl)malonate

Physical Appearance: Light yellow sticky solid

Yield: 86% (40.8 mg)

***R$_f$*:** 0.35 (hexane/ethyl acetate, 7:3 v/v)

^{1}H NMR (400 MHz, CDCl$_3$) δ 7.99 – 7.90 (m, 2H), 7.61 – 7.42 (m, 5H), 7.43 – 7.33 (m, 5H), 7.32 – 7.23 (m, 2H), 4.56 (dd, *J* = 10.9, 6.3 Hz, 1H), 4.44 (t, *J* = 10.7 Hz, 1H), 3.91 – 3.76 (m, 2H), 3.78 – 3.64 (m, 1H), 3.53 – 3.40 (m, 1H), 1.03 (t, *J* = 7.1 Hz, 3H), 0.90 (t, *J* = 7.1 Hz, 3H).

^{13}C NMR (101 MHz, CDCl$_3$) δ 167.1 (d, J_{c-p} = 1.7 Hz), 166.7 (d, J_{c-p} = 14.3 Hz), 139.8 (d, J_{c-p} = 5.4 Hz), 132.5 (d, J_{c-p} = 2.9 Hz), 132.1 (d, J_{c-p} = 2.7 Hz), 132.0 (d, J_{c-p} = 9.3 Hz), 131.8 (d, J_{c-p} = 2.0 Hz), 131.3 (d, J_{c-p} = 5.2 Hz), 131.0 (d, J_{c-p} = 8.7 Hz), 130.5 (d, J_{c-p} = 102.1 Hz), 130.5 (d, J_{c-p} = 96.2 Hz), 128.6 (d, J_{c-p} = 12.2 Hz), 128.5, 118.6, 111.3 (d, J_{c-p} = 2.6 Hz), 62.1 (d, J_{c-p} = 16.5 Hz), 52.1, 46.3 (d, J_{c-p} = 63.1 Hz), 13.7.

^{31}P NMR (162 MHz, CDCl$_3$) δ 29.67.

HRMS (ESI-TOF) m/z: [M + Na]$^+$ Calcd. for $C_{27}H_{26}NNaO_5P$ 498.1441; Found 498.1422.

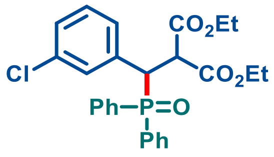

3e

diethyl-2-((3-chlorophenyl)(diphenylphosphoryl)methyl)malonate

Physical Appearance: Colourless sticky solid

Yield: 81% (39.2 mg)

***R$_f$*:** 0.3 (hexane/ethyl acetate, 7:3 v/v)

^{1}H NMR (400 MHz, CDCl$_3$) δ 7.98 – 7.88 (m, 2H), 7.58 – 7.43 (m, 5H), 7.41 – 7.31 (m, 1H), 7.32 – 7.21 (m, 2H), 7.18 (t, *J* = 1.9 Hz, 1H), 7.18 – 7.10 (m, 1H), 7.10 – 6.96 (m, 2H), 4.49 (dd, *J* = 11.0, 6.6 Hz, 1H), 4.45 – 4.34 (m, 1H), 3.83 (m, 2H), 3.78 – 3.69 (m, 1H), 3.53 (m, 1H), 1.05 (t, *J* = 7.1 Hz, 3H), 0.90 (t, *J* = 7.1 Hz, 3H).

^{13}C NMR (101 MHz, CDCl$_3$) δ 167.3, 166.8 (d, J_{c-p} = 14.6 Hz), 136.0 (d, J_{c-p} = 5.2 Hz), 133.9 (d, J_{c-p} = 2.2 Hz), 132.2 (d, J_{c-p} = 2.9 Hz), 132,0 (d, J_{c-p} = 9.2 Hz), 131.8 (d, J_{c-p} = 2.8 Hz), 131.3 (d, J_{c-p} = 8.6 Hz), 131.1 (d, J_{c-p} = 95.4 Hz), 130.78 (d, J_{c-p} = 5.3 Hz), 130.76 (d, J_{c-p} = 102.0 Hz), 129.3 (d, J_{c-p} = 2.0 Hz), 128.7 (d, J_{c-p} = 5.2 Hz), 128.5 (d, J_{c-p} = 11.7 Hz), 128.3 (d, J_{c-p} = 12.0 Hz), 127.7 (d, J_{c-p} = 2.5 Hz), 62.0 (d, J_{c-p} = 16.4 Hz), 52.3, 46.0 (d, J_{c-p} = 64.4 Hz), 13.7 (d, J_{c-p} = 10.5 Hz).

^{31}P NMR (162 MHz, CDCl$_3$) δ 30.04.

HRMS (ESI-TOF) m/z: [M + H]$^+$ Calcd. for $C_{26}H_{27}ClO_5P$ 485.1279; Found 485.1271.

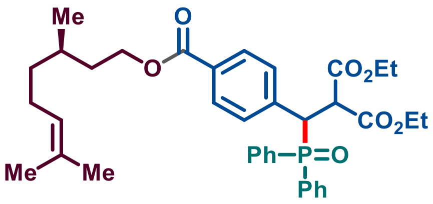

3f

diethyl-((4-(((3,7-dimethyloct-6-en-1-yl)oxy)carbonyl)phenyl)(diphenylphosphoryl)methyl)malonate *(dr= 1:1).*

Physical Appearance: Colourless sticky solid

Yield: 79% (49.9 mg)

***R$_f$*:** 0.3 (hexane/ethyl acetate, 7:3 v/v)

^{1}H NMR (500 MHz, CDCl$_3$) δ 7.99 – 7.89 (m, 2H), 7.77 (d, *J* = 8.5 Hz, 2H), 7.57 – 7.44 (m, 5H), 7.39 – 7.29 (m, 3H), 7.28 – 7.22 (m, 2H), 5.20 – 4.97 (m, 1H), 4.59 (dd, *J* = 11.1, 6.8 Hz, 1H), 4.46 (t, *J* = 10.8 Hz, 1H), 4.36 – 4.22 (m, 2H), 3.87 – 3.69 (m, 3H), 3.52 (m, 1H), 1.99 (m, 2H), 1.82 – 1.70 (m, 2H), 1.67 (s, 3H), 1.59 (s, 3H), 1.53 (m, 1H), 1.43 – 1.33 (m, 1H), 1.24 – 1.18 (m, 1H), 1.06 (t, *J* = 7.1 Hz, 3H), 0.94 (d, *J* = 6.5 Hz, 3H), 0.88 (t, *J* = 7.1 Hz, 3H).

^{13}C NMR (101 MHz, CDCl$_3$) δ 167.0 (d, $J_{c\text{-}p}$ = 43.7 Hz), 166.4 (d, $J_{c\text{-}p}$ = 28.5 Hz), 139.1 (d, $J_{c\text{-}p}$ = 5.3 Hz), 132.1 (d, $J_{c\text{-}p}$ = 2.9 Hz), 131.9 (d, $J_{c\text{-}p}$ = 9.2 Hz), 131.7 (d, $J_{c\text{-}p}$ = 2.6 Hz), 131.4 (d, $J_{c\text{-}p}$ = 2.7 Hz), 131.1 (d, $J_{c\text{-}p}$ = 8.7 Hz), 130.49 (d, $J_{c\text{-}p}$ = 5.2 Hz), 130.48, 130.1, 129.4 (d, $J_{c\text{-}p}$ = 2.5 Hz), 129.2, 129.1, 128.4 (d, $J_{c\text{-}p}$ = 11.8 Hz), 128.2 (d, $J_{c\text{-}p}$ = 11.9 Hz), 124.5, 63.6, 61.8 (d, $J_{c\text{-}p}$ = 18.7 Hz), 52.3, 46.2 (d, $J_{c\text{-}p}$ = 63.8 Hz), 37.0, 35.4, 29.5, 25.7, 25.4, 19.5, 17.7, 13.7, 13.6.

^{31}P NMR (202 MHz, CDCl$_3$) δ 29.78.

HRMS (ESI-TOF) m/z: [M + H]$^+$ Calcd. for $C_{37}H_{46}O_7P$ 633.2976; Found 633.2968.

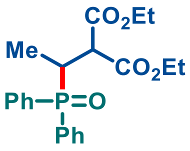

3g

diethyl-2-(1-(diphenylphosphoryl)ethyl)malonate

Physical Appearance: White solid (Mp: 110-113 °C)

Yield: 68% (26.4 mg)

***R_f*:** 0.3 (hexane/ethyl acetate, 7:3 v/v)

^{1}H NMR (400 MHz, $CDCl_3$) δ 7.87 – 7.72 (m, 4H), 7.59 – 7.36 (m, 6H), 4.20 – 4.03 (m, 2H), 4.00 – 3.84 (m, 1H), 3.84 – 3.70 (m, 2H), 3.40 – 3.24 (m, 1H), 1.33 – 1.12 (m, 6H), 1.14 – 1.04 (m, 3H).

^{13}C NMR (101 MHz, $CDCl_3$) δ 168.1 (d, J_{c-p} = 9.3 Hz), 167.6 (d, J_{c-p} = 9.1 Hz), 131.99 (d, J_{c-p} = 5.9 Hz), 131.98, 131.45 (d, J_{c-p} = 9.3 Hz), 131.39 (d, J_{c-p} = 96.2 Hz), 131.3 (d, J_{c-p} = 8.5 Hz), 131.0 (d, J_{c-p} = 97.9 Hz), 128.7 (d, J_{c-p} = 11.6 Hz), 128.6 (d, J_{c-p} = 11.5 Hz), 61.7 (d, J_{c-p} = 15.2 Hz), 50.6, 32.7 (d, J_{c-p} = 71.2 Hz), 13.9 (d, J_{c-p} = 17.4 Hz), 11.0 (d, J_{c-p} = 2.2 Hz).

^{31}P NMR (162 MHz, $CDCl_3$) δ 34.43.

HRMS (ESI-TOF) m/z: $[M + Na]^+$ Calcd. for $C_{21}H_{25}NaO_5P$ 411.1332; Found 411.1319.

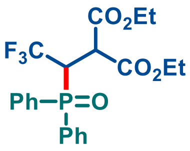

3h

diethyl-2-(1-(diphenylphosphoryl)-2,2,2-trifluoroethyl)malonate

Physical Appearance: Light yellow oil

Yield: 81% (35.8 mg)

***R_f*:** 0.3 (hexane/ethyl acetate, 7:3 v/v)

^{1}H NMR (500 MHz, $CDCl_3$) δ 7.90 – 7.79 (m, 4H), 7.62 – 7.55 (m, 1H), 7.57 – 7.50 (m, 3H), 7.51 – 7.44 (m, 2H), 4.35 (m, 1H), 4.18 (q, J = 7.2 Hz, 2H), 4.14 – 4.01 (m, 3H), 1.23 (t, J = 7.1 Hz, 3H), 1.16 (td, J = 7.2, 1.1 Hz, 3H).

^{13}C NMR (126 MHz, $CDCl_3$) δ 166.7 (d, J_{c-p} = 5.0 Hz), 166.4 (d, J_{c-p} = 8.8 Hz), 132.6 (d, J_{c-p} = 3.0 Hz), 132.3 (d, J_{c-p} = 10.0 Hz), 131.3, 131.2, 130.5, 129.8 (d, J_{c-p} = 102.1 Hz), 128.9 (d, J_{c-p} = 12.4 Hz), 128.4 (d, J_{c-p} = 12.7 Hz), 124.6 (q, J = 280.4 Hz), 62.4 (d, J_{c-p} = 12.9 Hz), 48.7, 46.3 (dd, J = 60.2, 27.2 Hz), 13.7 (d, J_{c-p} = 3.3 Hz).

^{19}F NMR (377 MHz, CDCl$_3$) δ -56.38.

^{31}P NMR (162 MHz, CDCl$_3$) δ 26.79.

HRMS (ESI-TOF) m/z: [M + Na]$^+$ Calcd. for $C_{21}H_{22}F_3NaO_5P$ 465.1049; Found 465.1049.

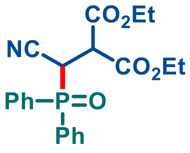

3i

diethyl-2-(cyano(diphenylphosphoryl)methyl)malonate

Physical Appearance: Colourless liquid

Yield: 89% (35.5 mg)

***R_f*:** 0.3 (hexane/ethyl acetate, 7:3 v/v)

^{1}H NMR (400 MHz, CDCl$_3$) δ 8.03 – 7.95 (m, 2H), 7.93 – 7.85 (m, 2H), 7.68 – 7.58 (m, 2H), 7.62 – 7.48 (m, 4H), 4.32 (dd, *J* = 16.4, 6.6 Hz, 1H), 4.25 – 4.14 (m, 2H), 4.14 – 3.88 (m, 3H), 1.24 (t, *J* = 7.1 Hz, 3H), 1.15 (t, *J* = 7.3 Hz, 3H).

^{13}C NMR (101 MHz, CDCl$_3$) δ 165.9 (d, $J_{c\text{-}p}$ = 7.3 Hz), 165.4 (d, $J_{c\text{-}p}$ = 6.5 Hz), 133.42, 133.41 (d, $J_{c\text{-}p}$ = 6.0 Hz), 132.5 (d, $J_{c\text{-}p}$ = 9.3 Hz), 131.3 (d, $J_{c\text{-}p}$ = 9.8 Hz), 129.6 (d, $J_{c\text{-}p}$ = 105.5 Hz), 129.2 (d, $J_{c\text{-}p}$ = 12.6 Hz), 128.8 (d, $J_{c\text{-}p}$ = 12.6 Hz), 127.4 (d, $J_{c\text{-}p}$ = 103.1 Hz), 115.3 (d, $J_{c\text{-}p}$ = 3.3 Hz), 62.8 (d, $J_{c\text{-}p}$ = 45.3 Hz), 48.4, 32.8 (d, $J_{c\text{-}p}$ = 60.8 Hz), 13.8 (d, $J_{c\text{-}p}$ = 15.7 Hz).

^{31}P NMR (162 MHz, CDCl$_3$) δ 25.81.

HRMS (ESI-TOF) m/z: [M + Na]$^+$ Calcd. for $C_{21}H_{22}NNaO_5P$ 422.1128; Found 422.1117.

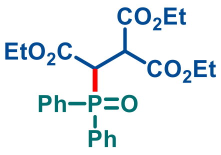

3j

triethyl-2-(diphenylphosphoryl)ethane-1,1,2-tricarboxylate

Physical Appearance: Colourless liquid

Yield: 89% (35.2 mg)

***R_f*:** 0.3 (hexane/ethyl acetate, 7:3 v/v)

^{1}H NMR (500 MHz, $CDCl_3$) δ 8.00 – 7.91 (m, 2H), 7.71 (m, 2H), 7.56 m, 1H), 7.50 (m, 3H), 7.41 (m, 2H), 4.51 (dd, J = 13.3, 11.6 Hz, 1H), 4.30 (dd, J = 11.6, 7.6 Hz, 1H), 4.12 (m, 2H), 3.96 (m, 2H), 3.69 (m, 1H), 3.59 (m, 1H), 1.19 (t, J = 7.1 Hz, 3H), 1.10 (t, J = 7.2 Hz, 3H), 0.87 (t, J = 7.2 Hz, 3H).

^{13}C NMR (126 MHz, $CDCl_3$) δ 168.1 (d, $J_{c\text{-}p}$ = 2.8 Hz), 167.7 (d, $J_{c\text{-}p}$ = 14.4 Hz), 166.7, 132.4 (d, $J_{c\text{-}p}$ = 2.9 Hz), 132.4 (d, $J_{c\text{-}p}$ = 2.8 Hz), 131.9 (d, $J_{c\text{-}p}$ = 9.5 Hz), 131.5 (d, $J_{c\text{-}p}$ = 9.6 Hz), 131.3 (d, $J_{c\text{-}p}$ = 103.7 Hz), 130.4 (d, $J_{c\text{-}p}$ = 101.2 Hz), 128.6 (d, $J_{c\text{-}p}$ = 12.6 Hz), 128.3 (d, $J_{c\text{-}p}$ = 12.2 Hz), 62.2, 62.1, 61.6, 50.5, 48.8 (d, $J_{c\text{-}p}$ = 56.0 Hz), 14.0, 13.7, 13.5.

^{31}P NMR (202 MHz, $CDCl_3$) δ 28.09.

HRMS (ESI-TOF) m/z: $[M + Na]^+$ Calcd. for $C_{23}H_{27}NaO_7P$ 469.1387; Found 469.1377.

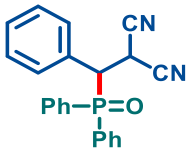

3k

2-((diphenylphosphoryl)(phenyl)methyl)malononitrile

Physical Appearance: White solid (Mp: 131-134 °C)

Yield: 88% (31.3 mg)

***R_f*:** 0.3 (hexane/ethyl acetate, 7:3 v/v)

^{1}H NMR (400 MHz, $CDCl_3$) δ 8.03 – 7.93 (m, 2H), 7.71 – 7.63 (m, 1H), 7.62 – 7.55 (m, 2H), 7.55 – 7.44 (m, 2H), 7.43 – 7.33 (m, 3H), 7.33 – 7.22 (m, 5H), 4.74 (t, J = 7.6 Hz, 1H), 4.06 (t, J = 7.7 Hz, 1H).

^{13}C NMR (101 MHz, $CDCl_3$) δ 133.3 (d, $J_{c\text{-}p}$ = 2.9 Hz), 132.5 (d, $J_{c\text{-}p}$ = 3.0 Hz), 131.38, 131.36 (d, $J_{c\text{-}p}$ = 17.5 Hz), 130.9 (d, $J_{c\text{-}p}$ = 4.1 Hz), 130.47, 129.8 (d, $J_{c\text{-}p}$ = 5.2 Hz), 129.44, 129.42 (d, $J_{c\text{-}p}$ = 9.1 Hz), 129.36, 128.8, 128.5 (d, $J_{c\text{-}p}$ = 12.4 Hz), 111.5, 111.4 (d, $J_{c\text{-}p}$ = 4.9 Hz), 47.09 (d, $J_{c\text{-}p}$ = 63.4 Hz), 24.9 (d, $J_{c\text{-}p}$ = 1.7 Hz).

^{31}P NMR (162 MHz, $CDCl_3$) δ 28.68.

HRMS (ESI-TOF) m/z: $[M + H]^+$ Calcd. for $C_{22}H_{18}N_2OP$ 357.1151; Found 357.1135.

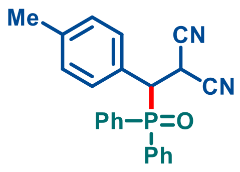

3l

2-((diphenylphosphoryl)(p-tolyl)methyl)malononitrile

Physical Appearance: White sticky solid

Yield: 86% (31.8 mg)

***R_f*:** 0.3 (hexane/ethyl acetate, 7:3 v/v)

^{1}H NMR (400 MHz, $CDCl_3$) δ 7.98 – 7.89 (m, 2H), 7.62 – 7.44 (m, 6H), 7.37 (t, *J* = 7.5 Hz, 1H), 7.25 (d, *J* = 8.8 Hz, 3H), 7.04 (d, *J* = 8.0 Hz, 2H), 4.68 (t, *J* = 7.4 Hz, 1H), 4.03 (t, *J* = 7.8 Hz, 1H), 2.24 (s, 3H).

^{13}C NMR (101 MHz, $CDCl_3$) δ 139.4, 133.7, 133.2, 132.4 (d, $J_{c\text{-}p}$ = 2.8 Hz), 132.3 (d, $J_{c\text{-}p}$ = 10.2 Hz), 131.4 (d, $J_{c\text{-}p}$ = 9.1 Hz), 130.3 (d, $J_{c\text{-}p}$ = 72.8 Hz), 130.0, 129.7 (d, $J_{c\text{-}p}$ = 5.2 Hz), 129.3 (d, $J_{c\text{-}p}$ = 11.7 Hz), 129.2 (d, $J_{c\text{-}p}$ = 13.0 Hz), 128.6 (d, $J_{c\text{-}p}$ = 66.5 Hz), 128.5 (d, $J_{c\text{-}p}$ = 12.4 Hz), 127.7 (d, $J_{c\text{-}p}$ = 4.3 Hz), 111.5 (d, $J_{c\text{-}p}$ = 9.3 Hz), 111.4, 46.7 (d, $J_{c\text{-}p}$ = 64.1 Hz), 25.0, 21.2.

^{31}P NMR (162 MHz, $CDCl_3$) δ 28.58.

HRMS (ESI-TOF) m/z: $[M + H]^+$ Calcd. for $C_{23}H_{20}N_2OP$ 371.1308; Found 371.1312.

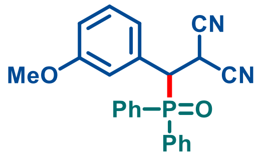

3m

2-((diphenylphosphoryl)(3-methoxyphenyl)methyl)malononitrile

Physical Appearance: White solid (Mp: 138-141 ^{o}C)

Yield: 83% (32.0 mg)

***R_f*:** 0.3 (hexane/ethyl acetate, 7:3 v/v)

^{1}H NMR (400 MHz, $CDCl_3$) δ 7.97 (dd, *J* = 10.0, 8.2 Hz, 2H), 7.74 – 7.44 (m, 5H), 7.38 (t, *J* = 6.7 Hz, 1H), 7.28 (dd, *J* = 7.9, 3.4 Hz, 1H), 7.17 (t, *J* = 8.0 Hz, 1H), 6.97 (d, *J* = 6.8 Hz, 1H),

6.93 (s, 1H), 6.80 (d, J = 8.4 Hz, 1H), 4.77 (t, J = 7.5 Hz, 1H), 4.09 (t, J = 7.8 Hz, 1H), 3.66 (s, 3H).

^{13}C NMR (101 MHz, $CDCl_3$) δ 159.9, 132.4 (d, $J_{c\text{-}p}$ = 2.8 Hz), 132.19 (d, $J_{c\text{-}p}$ = 13.8 Hz), 132.16, 131.4 (d, $J_{c\text{-}p}$ = 1.7 Hz), 131.3 (d, $J_{c\text{-}p}$ = 1.6 Hz), 130.3, 130.0 (d, $J_{c\text{-}p}$ = 97.8 Hz), 129.30 (d, $J_{c\text{-}p}$ = 11.9 Hz), 129.28 (d, $J_{c\text{-}p}$ = 104.5 Hz), 129.1 (d, $J_{c\text{-}p}$ = 12.7 Hz), 128.4 (d, $J_{c\text{-}p}$ = 12.4 Hz), 122.1 (d, $J_{c\text{-}p}$ = 5.5 Hz), 115.4 (d, $J_{c\text{-}p}$ = 2.0 Hz), 114.9 (d, $J_{c\text{-}p}$ = 5.1 Hz), 111.5 (d, $J_{c\text{-}p}$ = 8.5 Hz), 55.3, 46.8 (d, $J_{c\text{-}p}$ = 63.4 Hz), 24.8 (d, $J_{c\text{-}p}$ = 1.7 Hz).

^{31}P NMR (162 MHz, $CDCl_3$) δ 28.70.

HRMS (ESI-TOF) m/z: $[M + Na]^+$ Calcd. for $C_{23}H_{19}N_2NaO_2P$ 409.1076; Found 409.1066.

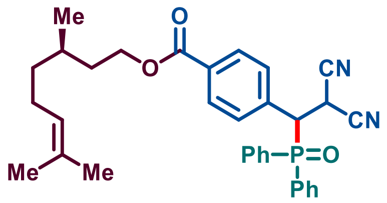

3n

3,7-dimethyloct-6-en-1-yl 4-(2,2-dicyano-1-(diphenylphosphoryl)ethyl)benzoate *(dr= 1:1).*

Physical Appearance: White sticky solid

Yield: 76% (40.9 mg)

R_f: 0.3 (hexane/ethyl acetate, 7:3 v/v)

^{1}H NMR (400 MHz, $CDCl_3$) δ 8.01 (m, 2H), 7.95 (d, J = 8.4 Hz, 2H), 7.71 – 7.65 (m, 1H), 7.65 – 7.58 (m, 2H), 7.58 – 7.50 (m, 4H), 7.47 – 7.36 (m, 1H), 7.30 (m, 2H), 5.11 (m, 1H), 4.78 (t, J = 7.5 Hz, 1H), 4.34 (m, 2H), 4.20 (t, J = 7.6 Hz, 1H), 2.12 – 1.92 (m, 2H), 1.87 – 1.74 (m, 1H), 1.68 (s, 3H), 1.64 (m, 1H), 1.61 (s, 3H), 1.57 (m, 1H), 1.40 (m, 1H), 1.32 – 1.17 (m, 1H), 0.97 (d, J = 6.5 Hz, 3H).

^{13}C NMR (101 MHz, $CDCl_3$) δ 165.9, 135.8 (d, $J_{c\text{-}p}$ = 4.1 Hz), 133.5 (d, $J_{c\text{-}p}$ = 2.9 Hz), 132.7 (d, $J_{c\text{-}p}$ = 2.9 Hz), 131.5, 131.39 (d, $J_{c\text{-}p}$ = 1.7 Hz), 131.38 (d, $J_{c\text{-}p}$ = 9.2 Hz), 131.2 (d, $J_{c\text{-}p}$ = 9.2 Hz), 130.4, 129.9 (d, $J_{c\text{-}p}$ = 5.1 Hz), 129.51 (d, $J_{c\text{-}p}$ = 12.0 Hz), 129.50 (d, $J_{c\text{-}p}$ = 98.4 Hz), 128.8 (d, $J_{c\text{-}p}$ = 90.3 Hz), 128.7 (d, $J_{c\text{-}p}$ = 12.4 Hz), 124.6, 111.2 (d, $J_{c\text{-}p}$ = 8.7 Hz), 63.9, 46.8 (d, $J_{c\text{-}p}$ = 62.2 Hz), 37.0, 35.5, 25.8, 25.4, 24.7, 19.6, 17.8.

^{31}P NMR (162 MHz, $CDCl_3$) δ 28.49.

HRMS (ESI-TOF) m/z: $[M + H]^+$ Calcd. for $C_{33}H_{36}N_2O_3P$ 539.2458; Found 539.2455.

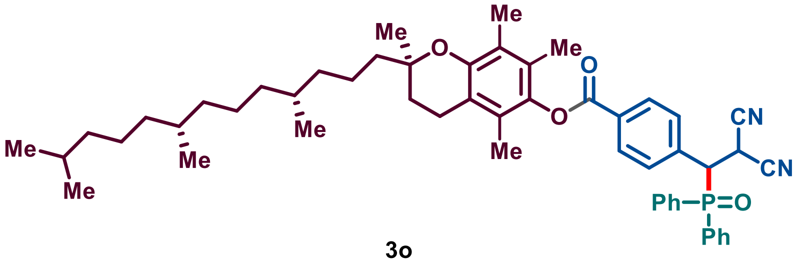

3o

2,5,7,8-tetramethyl-2-((4R,8R)-4,8,12-trimethyltridecyl)chroman-6-yl-4-(2,2-dicyano-1-(diphenylphosphoryl)ethyl)benzoate *(dr= 1:1).*

Physical Appearance: White sticky solid

Yield: 78% (63.3 mg)

***Rf*:** 0.25 (hexane/ethyl acetate, 7:3 v/v)

^{1}H NMR (500 MHz, $CDCl_3$) δ 8.16 (d, *J* = 8.2 Hz, 1H), 8.07 – 8.00 (m, 1H), 7.71 (td, *J* = 7.4, 1.5 Hz, 1H), 7.66 (dt, *J* = 7.0, 4.3 Hz, 1H), 7.62 – 7.53 (m, 2H), 7.46 (td, *J* = 7.5, 1.5 Hz, 1H), 7.35 (td, *J* = 7.7, 3.2 Hz, 1H), 4.81 (t, *J* = 7.4 Hz, 1H), 4.21 (t, *J* = 7.5 Hz, 1H), 2.64 (t, *J* = 6.8 Hz, 2H), 2.14 (s, 3H), 2.05 (s, 3H), 2.01 (s, 3H), 1.91 – 1.76 (m, 2H), 1.65 – 1.50 (m, 3H), 1.46 – 1.32 (m, 6H), 1.29 (m, 7H), 1.22 – 1.03 (m, 7H), 0.89 (m, 13H).

^{13}C NMR (126 MHz, $CDCl_3$) δ 164.4, 149.6, 140.5, 136.3 (d, $J_{c\text{-}p}$ = 4.1 Hz), 133.5 (d, $J_{c\text{-}p}$ = 2.9 Hz), 132.7 (d, $J_{c\text{-}p}$ = 3.0 Hz), 131.3 (d, $J_{c\text{-}p}$ = 9.1 Hz), 131.2 (d, $J_{c\text{-}p}$ = 9.2 Hz), 130.9, 130.5, 130.1 (d, $J_{c\text{-}p}$ = 5.0 Hz), 129.5 (d, $J_{c\text{-}p}$ = 12.1 Hz), 129.4 (d, $J_{c\text{-}p}$ = 98.4 Hz), 128.9 (d, $J_{c\text{-}p}$ = 105.1 Hz), 128.7 (d, $J_{c\text{-}p}$ = 12.4 Hz), 126.8, 125.0, 123.2, 117.6, 111.1 (d, $J_{c\text{-}p}$ = 4.2 Hz), 111.0 (d, $J_{c\text{-}p}$ = 4.9 Hz), 75.2, 47.0 (d, $J_{c\text{-}p}$ = 61.6 Hz), 39.4, 37.5, 37.4, 37.3, 32.81, 32.80, 28.0, 24.84, 24.82, 24.6, 24.5, 22.8, 22.6, 21.1, 20.6, 19.8, 19.72, 19.69, 19.66, 19.6, 13.1, 12.3, 11.9.

^{31}P NMR (202 MHz, $CDCl_3$) δ 28.44.

HRMS (ESI-TOF) m/z: $[M + Na]^+$ Calcd. for $C_{52}H_{65}N_2NaO_4P$ 835.4574; Found 835.4542.

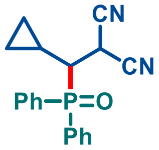

3p

2-(cyclopropyl(diphenylphosphoryl)methyl)malononitrile

Physical Appearance: White sticky solid

Yield: 91% (29.1 mg)

***R_f*:** 0.3 (hexane/ethyl acetate, 7:3 v/v)

^{1}H NMR (500 MHz, CDCl$_3$) δ 7.97 – 7.88 (m, 2H), 7.87 – 7.78 (m, 2H), 7.68 – 7.60 (m, 2H), 7.55 (m, 4H), 4.65 (dd, *J* = 8.3, 3.1 Hz, 1H), 2.27 – 2.18 (m, 1H), 1.16 (m, 1H), 0.89 (m, 1H), 0.73 (m, 1H), 0.47 – 0.37 (m, 1H), -0.10 (m, 1H).

^{13}C NMR (126 MHz, CDCl$_3$) δ 133.3 (d, $J_{c\text{-}p}$ = 2.8 Hz), 133.2 (d, $J_{c\text{-}p}$ = 3.0 Hz), 132.4 (d, $J_{c\text{-}p}$ = 9.4 Hz), 131.2 (d, $J_{c\text{-}p}$ = 9.8 Hz), 130.2 (d, $J_{c\text{-}p}$ = 99.5 Hz), 129.4 (d, $J_{c\text{-}p}$ = 11.8 Hz), 128.9 (d, $J_{c\text{-}p}$ = 12.1 Hz), 127.6 (d, $J_{c\text{-}p}$ = 100.1 Hz), 112.9 (d, $J_{c\text{-}p}$ = 13.8 Hz), 110.8 (d, $J_{c\text{-}p}$ = 2.7 Hz), 45.9 (d, $J_{c\text{-}p}$ = 67.0 Hz), 23.7 (d, $J_{c\text{-}p}$ = 3.3 Hz), 8.9, 6.1 (d, $J_{c\text{-}p}$ = 10.8 Hz), 5.4.

^{31}P NMR (162 MHz, CDCl$_3$) δ 31.18.

HRMS (ESI-TOF) m/z: $[M + H]^+$ Calcd. for $C_{19}H_{18}N_2OP$ 321.1151; Found 321.1150.

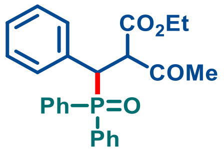

3q

ethyl 2-((diphenylphosphoryl)(phenyl)methyl)-3-oxobutanoate *(dr= 1:1).*

Physical Appearance: Light yellow oil

Yield: 76% (31.9 mg)

***R_f*:** 0.3 (hexane/ethyl acetate, 7:3 v/v)

^{1}H NMR (500 MHz, CDCl$_3$) δ 7.97 – 7.84 (m, 4H), 7.61 – 7.39 (m, 11H), 7.36 – 7.27 (m, 2H), 7.26 – 7.18 (m, 4H), 7.19 – 7.12 (m, 2H), 7.08 (m, 7H), 4.78 – 4.65 (m, 3H), 4.55 (dd, *J* = 11.2, 7.1 Hz, 1H), 3.84 – 3.63 (m, 3H), 3.60 – 3.50 (m, 1H), 2.13 (s, 3H), 1.96 (s, 3H), 1.05 (t, *J* = 7.1 Hz, 3H), 0.82 (t, *J* = 7.1 Hz, 3H).

^{13}C NMR (126 MHz, CDCl$_3$) δ 200.8 (d, $J_{c\text{-}p}$ = 11.9 Hz), 200.3, 167.4, 166.7 (d, $J_{c\text{-}p}$ = 14.9 Hz), 134.4 (d, $J_{c\text{-}p}$ = 5.0 Hz), 133.8 (d, $J_{c\text{-}p}$ = 5.2 Hz), 132.2 (d, $J_{c\text{-}p}$ = 2.7 Hz), 132.1 (d, $J_{c\text{-}p}$ = 2.8 Hz), 131.96, 131.95 (d, $J_{c\text{-}p}$ = 6.8 Hz), 131.9 (d, $J_{c\text{-}p}$ = 7.1 Hz), 131.63 (d, $J_{c\text{-}p}$ = 8.6 Hz), 131.61, 131.5 (d, $J_{c\text{-}p}$ = 2.8 Hz), 131.3 (d, $J_{c\text{-}p}$ = 8.6 Hz), 130.9, 130.3 (d, $J_{c\text{-}p}$ = 8.1 Hz), 130.1, 128.6 (d, $J_{c\text{-}p}$ = 11.7 Hz), 128.5, 128.4 (d, $J_{c\text{-}p}$ = 9.1 Hz), 128.2 (d, $J_{c\text{-}p}$ = 2.1 Hz), 128.1 (d, $J_{c\text{-}p}$

= 7.9 Hz), 128.0 (d, $J_{c\text{-}p}$ = 7.9 Hz), 127.5 (d, $J_{c\text{-}p}$ = 2.6 Hz), 127.3 (d, $J_{c\text{-}p}$ = 2.7 Hz), 61.9, 61.7, 60.0, 59.5, 46.5 (d, $J_{c\text{-}p}$ = 7.9 Hz), 45.9 (d, $J_{c\text{-}p}$ = 7.7 Hz), 30.4, 30.3, 13.7 (d, $J_{c\text{-}p}$ = 21.7 Hz).

^{31}P NMR (162 MHz, CDCl$_3$) δ 31.78, 30.86.

HRMS (ESI-TOF) m/z: [M + Na]$^+$ Calcd. for $C_{25}H_{25}NaO_4P$ 443.1383; Found 443.1380.

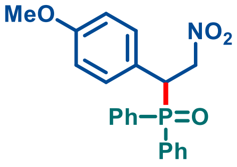

3r

(1-(4-methoxyphenyl)-2-nitroethyl)diphenylphosphine oxide

Physical Appearance: Light Yellow solid (Mp: 124-126 °C)

Yield: 85% (32.4 mg)

***R$_f$*:** 0.3 (hexane/ethyl acetate, 7:3 v/v)

^{1}H NMR (400 MHz, CDCl$_3$) δ 7.96 (dd, *J* = 9.5, 8.0 Hz, 2H), 7.67 – 7.57 (m, 3H), 7.50 – 7.37 (m, 3H), 7.30 (dd, *J* = 7.8, 3.3 Hz, 2H), 7.20 (dd, *J* = 8.7, 1.9 Hz, 2H), 6.74 (d, *J* = 8.7 Hz, 2H), 5.10 – 4.98 (m, 1H), 4.77 – 4.64 (m, 1H), 4.42 – 4.31 (m, 1H), 3.73 (s, 3H).

^{13}C NMR (101 MHz, CDCl$_3$) δ 159.6 (d, $J_{c\text{-}p}$ = 1.8 Hz), 132.8 (d, $J_{c\text{-}p}$ = 2.6 Hz), 132.2 (d, $J_{c\text{-}p}$ = 2.9 Hz), 131.3 (d, $J_{c\text{-}p}$ = 8.8 Hz), 131.2 (d, $J_{c\text{-}p}$ = 9.2 Hz), 130.7 (d, $J_{c\text{-}p}$ = 5.0 Hz), 129.44 (d, $J_{c\text{-}p}$ = 11.5 Hz), 129.38 (d, $J_{c\text{-}p}$ = 77.9 Hz), 129.2 (d, $J_{c\text{-}p}$ = 84.1 Hz), 128.5 (d, $J_{c\text{-}p}$ = 12.2 Hz), 123.3 (d, $J_{c\text{-}p}$ = 5.8 Hz), 114.4 (d, $J_{c\text{-}p}$ = 1.7 Hz), 76.1 (d, $J_{c\text{-}p}$ = 6.7 Hz), 55.3, 45.2 (d, $J_{c\text{-}p}$ = 65.2 Hz).

^{31}P NMR (162 MHz, CDCl$_3$) δ 30.16.

HRMS (ESI-TOF) m/z: [M + Na]$^+$ Calcd. for $C_{21}H_{20}NNaO_4P$ 404.1022; Found 404.1007.

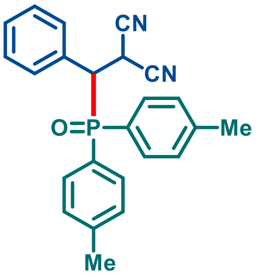

3s

2-((di-p-tolylphosphoryl)(phenyl)methyl)malononitrile

Physical Appearance: White solid (Mp: 153-155 °C)

Yield: 87% (33.4 mg)

R_f: 0.3 (hexane/ethyl acetate, 7:3 v/v)

^{1}H NMR (400 MHz, CDCl$_3$) δ 7.83 (dd, *J* = 11.2, 7.8 Hz, 2H), 7.47 – 7.39 (m, 2H), 7.45 – 7.32 (m, 4H), 7.31 – 7.23 (m, 3H), 7.05 (dd, *J* = 8.2, 3.1 Hz, 2H), 4.73 (td, *J* = 7.2, 1.3 Hz, 1H), 4.06 (m, 1H), 2.41 (s, 3H), 2.25 (s, 3H).

^{13}C NMR (101 MHz, CDCl$_3$) δ 143.9 (d, $J_{c\text{-}p}$ = 2.8 Hz), 143.0 (d, $J_{c\text{-}p}$ = 3.0 Hz), 131.34 (d, $J_{c\text{-}p}$ = 9.6 Hz), 131.33 (d, $J_{c\text{-}p}$ = 9.3 Hz), 131.1 (d, $J_{c\text{-}p}$ = 4.1 Hz), 130.0 (d, $J_{c\text{-}p}$ = 12.2 Hz), 129.9 (d, $J_{c\text{-}p}$ = 5.2 Hz), 129.3 (d, $J_{c\text{-}p}$ = 1.8 Hz), 129.2 (d, $J_{c\text{-}p}$ = 1.8 Hz), 129.1, 126.8 (d, $J_{c\text{-}p}$ = 100.4 Hz), 126.1 (d, $J_{c\text{-}p}$ = 107.3 Hz), 111.6 (d, $J_{c\text{-}p}$ = 8.5 Hz), 111.5 (d, $J_{c\text{-}p}$ = 8.8 Hz), 46.9 (d, $J_{c\text{-}p}$ = 63.6 Hz), 24.9 (d, $J_{c\text{-}p}$ = 1.8 Hz), 21.7, 21.6.

^{31}P NMR (162 MHz, CDCl$_3$) δ 29.35.

HRMS (ESI-TOF) m/z: [M + Na]$^+$ Calcd. for $C_{24}H_{21}N_2NaOP$ 407.1284; Found 407.1274.

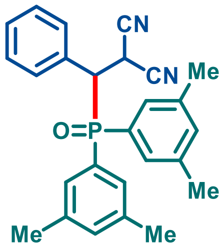

3t

2-((bis(3,5-dimethylphenyl)phosphoryl)(phenyl)methyl)malononitrile

Physical Appearance: White sticky solid

Yield: 91% (37.5 mg)

R_f: 0.3 (hexane/ethyl acetate, 7:3 v/v)

^{1}H NMR (400 MHz, CDCl$_3$) δ 7.50 (dd, *J* = 11.7, 1.5 Hz, 2H), 7.40 (m, 2H), 7.26 (dd, *J* = 4.9, 1.9 Hz, 3H), 7.22 (s, 1H), 7.02 (dd, *J* = 12.1, 1.6 Hz, 2H), 6.96 (s, 1H), 4.64 (t, *J* = 7.2 Hz, 1H), 3.97 (t, *J* = 7.5 Hz, 1H), 2.36 (s, 6H), 2.14 (s, 6H).

^{13}C NMR (101 MHz, CDCl$_3$) δ 139.2 (d, $J_{c\text{-}p}$ = 12.4 Hz), 138.1 (d, $J_{c\text{-}p}$ = 13.1 Hz), 134.9 (d, $J_{c\text{-}p}$ = 2.9 Hz), 134.1 (d, $J_{c\text{-}p}$ = 3.1 Hz), 131.1 (d, $J_{c\text{-}p}$ = 4.1 Hz), 130.0 (d, $J_{c\text{-}p}$ = 5.2 Hz), 129.6

(d, $J_{c\text{-}p}$ = 96.7 Hz), 129.3 (d, $J_{c\text{-}p}$ = 1.9 Hz), 129.1, 129.0 (d, $J_{c\text{-}p}$ = 103.8 Hz), 129.0 (d, $J_{c\text{-}p}$ = 5.0 Hz), 128.9 (d, $J_{c\text{-}p}$ = 4.9 Hz), 111.6 (d, $J_{c\text{-}p}$ = 8.9 Hz), 111.4 (d, $J_{c\text{-}p}$ = 8.0 Hz), 46.8 (d, $J_{c\text{-}p}$ = 62.8 Hz), 24.9 (d, $J_{c\text{-}p}$ = 1.8 Hz), 21.5, 21.2.

^{31}P NMR (162 MHz, $CDCl_3$) δ 29.44.

HRMS (ESI-TOF) m/z: [M + Na]$^+$ Calcd. for $C_{26}H_{25}N_2NaOP$ 435.1597; Found 435.1584.

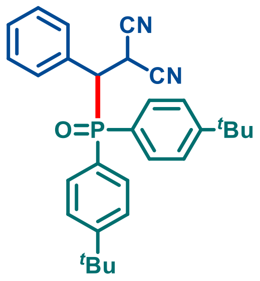

3u

2-((bis(4-(tert-butyl)phenyl)phosphoryl)(phenyl)methyl)malononitrile

Physical Appearance: Yellow sticky solid

Yield: 75% (35.1 mg)

***R_f*:** 0.35 (hexane/ethyl acetate, 7:3 v/v)

^{1}H NMR (400 MHz, $CDCl_3$) δ 7.87 (dd, J = 11.1, 8.5 Hz, 2H), 7.58 (dd, J = 8.4, 2.9 Hz, 2H), 7.47 – 7.36 (m, 5H), 7.31 – 7.26 (m, 4H), 4.71 (t, J = 7.0 Hz, 1H), 4.02 (dd, J = 8.5, 6.8 Hz, 1H), 1.34 (s, 9H), 1.21 (s, 9H).

^{13}C NMR (101 MHz, $CDCl_3$) δ 156.9 (d, $J_{c\text{-}p}$ = 2.8 Hz), 156.1 (d, $J_{c\text{-}p}$ = 2.9 Hz), 131.3 (d, $J_{c\text{-}p}$ = 3.7 Hz), 131.2 (d, $J_{c\text{-}p}$ = 3.6 Hz), 131.0 (d, $J_{c\text{-}p}$ = 4.0 Hz), 130.0 (d, $J_{c\text{-}p}$ = 5.3 Hz), 129.3 (d, $J_{c\text{-}p}$ = 1.9 Hz), 129.2, 126.7 (d, $J_{c\text{-}p}$ = 100.5 Hz), 126.6, 126.4 (d, $J_{c\text{-}p}$ = 12.1 Hz), 125.5 (d, $J_{c\text{-}p}$ = 12.6 Hz), 111.7 (d, $J_{c\text{-}p}$ = 9.7 Hz), 111.5 (d, $J_{c\text{-}p}$ = 7.9 Hz), 47.2 (d, $J_{c\text{-}p}$ = 63.4 Hz), 35.2, 35.0, 31.1, 31.0, 25.1 (d, $J_{c\text{-}p}$ = 1.9 Hz).

^{31}P NMR (162 MHz, $CDCl_3$) δ 29.22.

HRMS (ESI-TOF) m/z: [M + Na]$^+$ Calcd. for $C_{30}H_{33}N_2NaOP$ 491.2223; Found 491.2212.

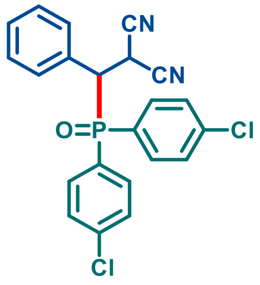

3v

2-((bis(4-chlorophenyl)phosphoryl)(phenyl)methyl)malononitrile

Physical Appearance: White sticky solid

Yield: 86% (36.5 mg)

R_f: 0.35 (hexane/ethyl acetate, 7:3 v/v)

^{1}H NMR (400 MHz, $CDCl_3$) δ 7.88 (dd, *J* = 11.1, 8.5 Hz, 2H), 7.56 (dd, *J* = 8.5, 2.5 Hz, 2H), 7.43 – 7.21 (m, 9H), 4.78 (t, *J* = 7.8 Hz, 1H), 4.07 (t, *J* = 7.9 Hz, 1H).
^{13}C NMR (101 MHz, $CDCl_3$) δ 140.4 (d, $J_{c\text{-}p}$ = 3.4 Hz), 139.5 (d, $J_{c\text{-}p}$ = 3.6 Hz), 132.7 (d, $J_{c\text{-}p}$ = 8.1 Hz), 132.6 (d, $J_{c\text{-}p}$ = 8.3 Hz), 130.4 (d, $J_{c\text{-}p}$ = 4.2 Hz), 129.9 (d, $J_{c\text{-}p}$ = 12.6 Hz), 129.8 (d, $J_{c\text{-}p}$ = 2.0 Hz), 129.7 (d, $J_{c\text{-}p}$ = 5.6 Hz), 129.6, 129.0 (d, $J_{c\text{-}p}$ = 13.0 Hz), 128.1 (d, $J_{c\text{-}p}$ = 99.2 Hz), 127.5 (d, $J_{c\text{-}p}$ = 107.0 Hz), 111.3 (d, $J_{c\text{-}p}$ = 1.7 Hz), 111.2 (d, $J_{c\text{-}p}$ = 4.3 Hz), 46.9 (d, $J_{c\text{-}p}$ = 64.9 Hz), 24.8 (d, $J_{c\text{-}p}$ = 1.7 Hz).
^{31}P NMR (162 MHz, $CDCl_3$) δ 28.06.
HRMS (ESI-TOF) m/z: $[M + Na]^+$ Calcd. for $C_{22}H_{15}Cl_2N_2NaOP$ 447.0191; Found 447.0176.

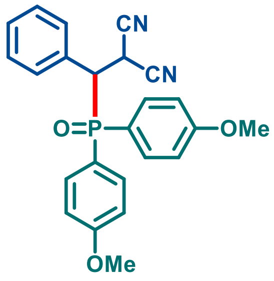

3w

2-((bis(4-methoxyphenyl)phosphoryl)(phenyl)methyl)malononitrile

Physical Appearance: white sticky solid

Yield: 92% (38.3 mg)

***R_f*:** 0.30 (hexane/ethyl acetate, 7:3 v/v)

^{1}H NMR (500 MHz, CDCl$_3$) δ 7.91 – 7.78 (m, 2H), 7.42 – 7.34 (m, 4H), 7.31 – 7.26 (m, 3H), 7.08 (dd, *J* = 8.9, 2.4 Hz, 2H), 6.78 (dd, *J* = 8.9, 2.5 Hz, 2H), 4.79 (t, *J* = 7.2 Hz, 1H), 4.02 (t, *J* = 7.9 Hz, 1H), 3.87 (s, 3H), 3.75 (s, 3H).

^{13}C NMR (126 MHz, CDCl$_3$) δ 163.4 (d, $J_{c\text{-}p}$ = 2.7 Hz), 162.7 (d, $J_{c\text{-}p}$ = 3.0 Hz), 133.4 (d, $J_{c\text{-}p}$ = 10.6 Hz), 133.3 (d, $J_{c\text{-}p}$ = 10.5 Hz), 131.2 (d, $J_{c\text{-}p}$ = 4.0 Hz), 129.9 (d, $J_{c\text{-}p}$ = 5.1 Hz), 129.29, 129.26, 120.9 (d, $J_{c\text{-}p}$ = 105.3 Hz), 120.4 (d, $J_{c\text{-}p}$ = 112.2 Hz), 114.9 (d, $J_{c\text{-}p}$ = 12.9 Hz), 114.0 (d, $J_{c\text{-}p}$ = 13.4 Hz), 111.7 (d, $J_{c\text{-}p}$ = 8.6 Hz), 111.6 (d, $J_{c\text{-}p}$ = 8.5 Hz), 55.5, 55.3, 47.4 (d, $J_{c\text{-}p}$ = 64.0 Hz), 24.8.

^{31}P NMR (162 MHz, CDCl$_3$) δ 29.30.

HRMS (ESI-TOF) m/z: $[M + H]^+$ Calcd. for $C_{24}H_{22}N_2O_3P$ 417.1363; Found 417.1363.

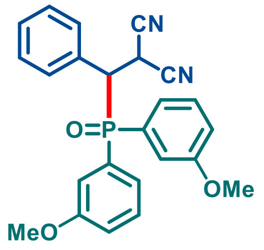

3x

2-((bis(3-methoxyphenyl)phosphoryl)(phenyl)methyl)malononitrile

Physical Appearance: Light brown solid (Mp: 181-184 °C)

Yield: 83% (34.5 mg)

***R_f*:** 0.30 (hexane/ethyl acetate, 7:3 v/v)

^{1}H NMR (500 MHz, CDCl$_3$) δ 7.54 – 7.45 (m, 3H), 7.43 – 7.37 (m, 2H), 7.29 (dd, *J* = 5.5, 1.8 Hz, 3H), 7.21 – 7.12 (m, 2H), 7.03 (d, *J* = 1.8 Hz, 1H), 7.00 (dt, *J* = 5.4, 3.8 Hz, 1H), 6.93 – 6.88 (m, 1H), 4.71 (t, *J* = 7.4 Hz, 1H), 4.02 (t, *J* = 7.7 Hz, 1H), 3.85 (s, 3H), 3.65 (s, 3H).

^{13}C NMR (126 MHz, CDCl$_3$) δ 160.2 (d, $J_{c\text{-}p}$ = 14.8 Hz), 159.4 (d, $J_{c\text{-}p}$ = 15.0 Hz), 131.1 (d, $J_{c\text{-}p}$ = 97.0 Hz), 130.9 (d, $J_{c\text{-}p}$ = 4.2 Hz), 130.63 (d, $J_{c\text{-}p}$ = 14.2 Hz), 130.57 (d, $J_{c\text{-}p}$ = 104.4 Hz), 129.9 (d, $J_{c\text{-}p}$ = 5.3 Hz), 129.7 (d, $J_{c\text{-}p}$ = 14.9 Hz), 129.5 (d, $J_{c\text{-}p}$ = 2.0 Hz), 129.4, 123.3 (d, $J_{c\text{-}p}$ = 9.8 Hz), 123.1 (d, $J_{c\text{-}p}$ = 9.1 Hz), 119.2 (d, $J_{c\text{-}p}$ = 2.9 Hz), 119.0 (d, $J_{c\text{-}p}$ = 2.8 Hz), 116.8 (d, $J_{c\text{-}p}$ = 9.9 Hz), 116.1 (d, $J_{c\text{-}p}$ = 9.9 Hz), 111.4 (d, $J_{c\text{-}p}$ = 9.2 Hz), 111.3, 55.7, 55.4, 47.0 (d, $J_{c\text{-}p}$ = 63.6 Hz), 25.0.

^{31}P NMR (162 MHz, CDCl$_3$) δ 29.09.

HRMS (ESI-TOF) m/z: [M +Na]$^+$ Calcd. for $C_{24}H_{21}N_2NaO_3P$ 439.1182; Found 439.1170

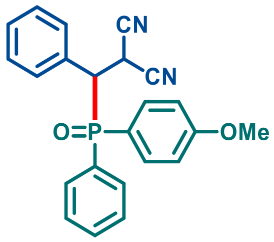

3y

2-(((4-methoxyphenyl)(phenyl)phosphoryl)(phenyl)methyl)malononitrile *(dr= 1:0.8).*

Physical Appearance: White solid (Mp: 210-213 °C)

Yield: 83% (34.5 mg)

***R$_f$*:** 0.30 (hexane/ethyl acetate, 7:3 v/v)

^{1}H NMR (500 MHz, CDCl$_3$) δ 7.97 – 7.86 (m, 4H), 7.65 – 7.53 (m, 3H), 7.46 (dd, *J* = 11.8, 7.7 Hz, 2H), 7.42 – 7.31 (m, 7H), 7.25 (m, 8H), 7.06 (m, 2H), 6.76 (dd, *J* = 8.8, 2.5 Hz, 2H), 4.78 (m, 2H), 4.08 (m, 2H), 3.84 (d, *J* = 3.3 Hz, 3H), 3.72 (d, *J* = 3.3 Hz, 3H).

^{13}C NMR (126 MHz, CDCl$_3$) δ 163.5 (d, J_{c-p} = 2.8 Hz), 162.8 (d, J_{c-p} = 3.0 Hz), 133.4 (d, J_{c-p} = 10.6 Hz), 133.3, 133.1 (d, J_{c-p} = 2.5 Hz), 132.2 (d, J_{c-p} = 2.6 Hz), 131.4, 131.2 (d, J_{c-p} = 9.5 Hz), 130.6 (d, J_{c-p} = 107.3 Hz), 130.3 (d, J_{c-p} = 98.3 Hz), 129.85 (d, J_{c-p} = 5.7 Hz), 129.79, 129.32, 129.27, 129.2, 128.4, 128.3, 120.5 (d, J_{c-p} = 66.9 Hz), 119.7 (d, J_{c-p} = 73.8 Hz), 115.0 (d, J_{c-p} = 12.9 Hz), 114.1 (d, J_{c-p} = 13.4 Hz), 111.7 – 111.4 (m), 55.5, 55.3, 47.0 (d, J_{c-p} = 64.0 Hz), 47.0 (d, J_{c-p} = 63.3 Hz), 24.9, 24.8.

^{31}P NMR (162 MHz, CDCl$_3$) δ 29.20.

HRMS (ESI-TOF) m/z: [M +H]$^+$ Calcd. for $C_{23}H_{20}N_2O_2P$ 387.1257; Found 387.1248.

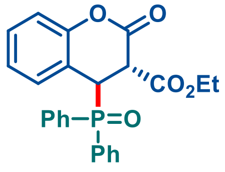

5a

ethyl4-(diphenylphosphoryl)-2-oxochromane-3-carboxylate

Physical Appearance: White solid (Mp: 180- 184 °C)

Yield: 91% (38.2 mg)

***R_f*:** 0.40 (hexane/ethyl acetate, 7:3 v/v)

^{1}H NMR (400 MHz, CDCl$_3$) δ 7.91 (m, 2H), 7.68 – 7.44 (m, 6H), 7.38 (m, 2H), 7.20 (dt, *J* = 7.7, 1.8 Hz, 1H), 7.02 (dd, *J* = 8.3, 1.2 Hz, 1H), 6.82 (t, *J* = 7.5 Hz, 1H), 6.50 (dt, *J* = 7.7, 2.0 Hz, 1H), 4.36 (dd, *J* = 10.0, 1.2 Hz, 1H), 4.15 – 3.97 (m, 3H), 1.04 (t, *J* = 7.1 Hz, 3H).

^{13}C NMR (101 MHz, CDCl$_3$) δ 166.8 (d, $J_{c\text{-}p}$ = 17.3 Hz), 162.0, 152.1 (d, $J_{c\text{-}p}$ = 4.3 Hz), 133.0 (d, $J_{c\text{-}p}$ = 2.8 Hz), 132.8 (d, $J_{c\text{-}p}$ = 2.9 Hz), 131.8 (d, $J_{c\text{-}p}$ = 9.0 Hz), 131.6 (d, $J_{c\text{-}p}$ = 8.9 Hz), 129.71 (d, $J_{c\text{-}p}$ = 6.1 Hz), 129.71, 129.3 (d, $J_{c\text{-}p}$ = 11.8 Hz), 129.1 (d, $J_{c\text{-}p}$ = 97.7 Hz), 128.6 (d, $J_{c\text{-}p}$ = 12.0 Hz), 128.3 (d, $J_{c\text{-}p}$ = 100.5 Hz), 124.2 (d, $J_{c\text{-}p}$ = 2.7 Hz), 117.6 (d, $J_{c\text{-}p}$ = 2.7 Hz), 115.2 (d, $J_{c\text{-}p}$ = 5.1 Hz), 63.0, 46.2, 42.0 (d, $J_{c\text{-}p}$ = 65.7 Hz), 13.8.

^{31}P NMR (162 MHz, CDCl$_3$) δ 30.84.

HRMS (ESI-TOF) m/z: $[M+Na]^+$ Calcd. for $C_{24}H_{21}NaO_5P$ 443.1019; Found 443.1003.

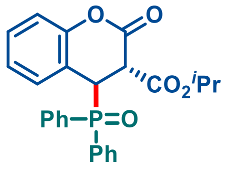

5b

Isopropyl-4-(diphenylphosphoryl)-2-oxochromane-3-carboxylate

Physical Appearance: White sticky solid

Yield: 90% (39.0 mg)

***R_f*:** 0.40 (hexane/ethyl acetate, 7:3 v/v)

^{1}H NMR (400 MHz, CDCl$_3$) δ 7.98 – 7.84 (m, 2H), 7.69 – 7.44 (m, 6H), 7.38 (td, *J* = 7.6, 3.0 Hz, 2H), 7.21 (tt, *J* = 7.7, 1.7 Hz, 1H), 7.02 (d, *J* = 8.2 Hz, 1H), 6.81 (t, *J* = 7.5 Hz, 1H), 6.49 (dt, *J* = 7.8, 1.9 Hz, 1H), 4.85 (hept, *J* = 6.3 Hz, 1H), 4.32 (dd, *J* = 9.7, 1.2 Hz, 1H), 4.04 (dd, *J* = 9.5, 1.2 Hz, 1H), 1.10 (d, *J* = 6.3 Hz, 3H), 0.88 (d, *J* = 6.2 Hz, 3H).

^{13}C NMR (101 MHz, CDCl$_3$) δ 166.4 (d, $J_{c\text{-}p}$ = 17.2 Hz), 162.2, 152.2 (d, $J_{c\text{-}p}$ = 4.2 Hz), 132.9 (d, $J_{c\text{-}p}$ = 2.9 Hz), 132.8 (d, $J_{c\text{-}p}$ = 2.9 Hz), 131.8 (d, $J_{c\text{-}p}$ = 9.0 Hz), 131.6 (d, $J_{c\text{-}p}$ = 9.0 Hz), 129.67 (d, $J_{c\text{-}p}$ = 6.3 Hz), 129.67, 129.3 (d, $J_{c\text{-}p}$ = 11.6 Hz), 129.0, 128.6 (d, $J_{c\text{-}p}$ = 12.1 Hz),

128.0, 124.2 (d, $J_{c\text{-}p}$ = 2.7 Hz), 117.5 (d, $J_{c\text{-}p}$ = 2.5 Hz), 115.4 (d, $J_{c\text{-}p}$ = 5.1 Hz), 71.1, 46.5, 42.2 (d, $J_{c\text{-}p}$ = 65.7 Hz), 21.3, 21.2.

^{31}P NMR (162 MHz, $CDCl_3$) δ 30.57.

HRMS (ESI-TOF) m/z: [M +Na]$^+$ Calcd. for $C_{25}H_{23}NaO_5P$ 457.1175; Found 457.1154.

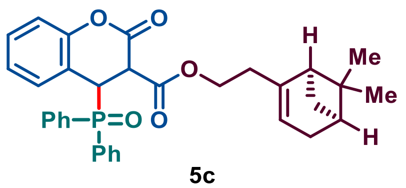

5c

2-((1R,5S)-6,6-dimethylbicyclo[3.1.1]hept-2-en-2-yl)ethyl-4-(diphenylphosphoryl)-2-oxochromane-3-carboxylate *(dr= 1:1).*

Physical Appearance: White sticky solid

Yield: 86% (46.5 mg)

***R_f*:** 0.40 (hexane/ethyl acetate, 7:3 v/v)

^{1}H NMR (500 MHz, $CDCl_3$) δ 7.89 (td, *J* = 9.2, 2.8 Hz, 2H), 7.61 (t, *J* = 7.6 Hz, 1H), 7.57 – 7.45 (m, 5H), 7.36 (td, *J* = 7.6, 3.3 Hz, 2H), 7.20 (t, *J* = 8.2 Hz, 1H), 6.99 (d, *J* = 8.4 Hz, 1H), 6.84 – 6.77 (m, 1H), 6.48 (d, *J* = 7.8 Hz, 1H), 5.08 (d, *J* = 10.0 Hz, 1H), 4.38 (d, *J* = 10.2 Hz, 1H), 4.13 – 3.91 (m, 3H), 2.25 (tt, *J* = 8.8, 4.0 Hz, 1H), 2.18 – 2.04 (m, 4H), 1.91 – 1.84 (m, 1H), 1.19 (s, 3H), 1.06 – 0.85 (m, 1H), 0.80 – 0.56 (m, 3H).

^{13}C NMR (126 MHz, $CDCl_3$) δ 174.2, 166.7 (d, $J_{c\text{-}p}$ = 17.1 Hz), 161.5, 152.0 (d, $J_{c\text{-}p}$ = 4.2 Hz), 143.1 (d, $J_{c\text{-}p}$ = 5.8 Hz), 133.0 (d, $J_{c\text{-}p}$ = 2.7 Hz), 132.8 (d, $J_{c\text{-}p}$ = 2.7 Hz), 131.8 (d, $J_{c\text{-}p}$ = 9.1 Hz), 131.5 (d, $J_{c\text{-}p}$ = 9.0 Hz), 129.7, 129.6, 129.3, 129.2, 128.6 (d, $J_{c\text{-}p}$ = 12.0 Hz), 128.4 (d, $J_{c\text{-}p}$ = 5.5 Hz), 127.6, 124.2 (d, $J_{c\text{-}p}$ = 2.7 Hz), 119.2 (d, $J_{c\text{-}p}$ = 8.2 Hz), 117.5 (d, $J_{c\text{-}p}$ = 2.6 Hz), 114.9 (d, $J_{c\text{-}p}$ = 4.2 Hz), 64.9, 64.8, 46.1, 45.5, 45.4, 42.0 (d, $J_{c\text{-}p}$ = 4.1 Hz), 41.5 (d, $J_{c\text{-}p}$ = 3.9 Hz), 40.6, 37.93, 37.88, 35.6, 35.5, 31.6, 31.5, 31.3, 26.18, 26.16, 21.1, 21.0.

^{31}P NMR (162 MHz, $CDCl_3$) δ 32.35.

HRMS (ESI-TOF) m/z: [M +Na]$^+$ Calcd. for $C_{33}H_{33}NaO_5P$ 563.1958; Found 563.1931.

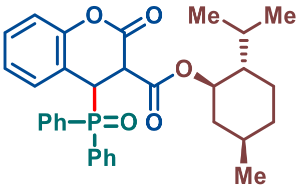

5d

(1R,2S,5R)-2-isopropyl-5-methylcyclohexyl-4-(diphenylphosphoryl)-2-oxochromane-3-carboxylate *(dr= 1:1).*

Physical Appearance: White sticky solid

Yield: 86% (45.6 mg)

***R_f*:** 0.40 (hexane/ethyl acetate, 7:3 v/v)

^{1}H NMR (400 MHz, $CDCl_3$) δ 7.96 – 7.86 (m, 2H), 7.64 – 7.48 (m, 6H), 7.37 (m, 2H), 7.26 – 7.18 (m, 1H), 7.01 (m, 1H), 6.81 (m, 1H), 6.48 (m, 1H), 4.52 (m, 1H), 4.37 – 4.28 (m, 1H), 4.06 (m, 1H), 2.05 – 1.77 (m, 1H), 1.67 – 1.46 (m, 3H), 1.40 – 1.12 (m, 3H), 0.97 – 0.85 (m, 1H), 0.79 (dd, J = 15.9, 6.8 Hz, 4H), 0.71 (d, J = 6.5 Hz, 2H), 0.57 (dd, J = 11.3, 6.9 Hz, 3H), 0.19 (d, J = 7.0 Hz, 1H).

^{13}C NMR (101 MHz, $CDCl_3$) δ 166.6 (d, $J_{c\text{-}p}$ = 17.5 Hz), 166.5 (d, $J_{c\text{-}p}$ = 17.1 Hz), 162.2, 162.1 (d, $J_{c\text{-}p}$ = 1.7 Hz), 152.3 (d, $J_{c\text{-}p}$ = 4.2 Hz), 152.2 (d, $J_{c\text{-}p}$ = 4.1 Hz), 132.94 (d, $J_{c\text{-}p}$ = 4.8 Hz), 132.92, 132.8 (d, $J_{c\text{-}p}$ = 2.8 Hz), 132.7 (d, $J_{c\text{-}p}$ = 2.9 Hz), 131.8, 131.75, 131.67, 131.6 (d, $J_{c\text{-}p}$ = 8.9 Hz), 129.8 (d, $J_{c\text{-}p}$ = 3.9 Hz), 129.63, 129.61 (d, $J_{c\text{-}p}$ = 9.9 Hz), 129.60, 129.38, 129.36, 129.3, 129.2, 128.6 (d, $J_{c\text{-}p}$ = 100.7 Hz), 128.64, 128.62, 128.60, 128.52, 128.51, 128.3 (d, $J_{c\text{-}p}$ = 100.3 Hz), 124.1 (d, $J_{c\text{-}p}$ = 2.6 Hz), 117.6 (d, $J_{c\text{-}p}$ = 3.0 Hz), 117.5, 115.4 (d, $J_{c\text{-}p}$ = 5.0 Hz), 115.2 (d, $J_{c\text{-}p}$ = 5.0 Hz), 46.6, 46.54, 46.48, 46.3, 42.4 (d, $J_{c\text{-}p}$ = 65.5 Hz), 42.3 (d, $J_{c\text{-}p}$ = 65.9 Hz), 40.2, 39.9, 33.9, 31.4, 31.2, 26.0, 25.3, 23.1, 22.6, 21.9, 21.8, 20.8, 20.7, 16.0, 15.1.

^{31}P NMR (162 MHz, $CDCl_3$) δ 30.86, 30.35.

HRMS (ESI-TOF) m/z: $[M+Na]^+$ Calcd. for $C_{32}H_{35}NaO_5P$ 563.1958; Found 563.1931.

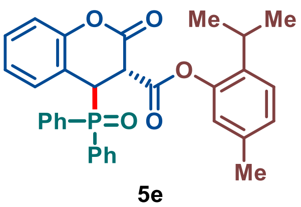

5e

2-isopropyl-5-methylphenyl-4-(diphenylphosphoryl)-2-oxochromane-3-carboxylate

Physical Appearance: White sticky solid

Yield: 90% (47.1 mg)

***R_f*:** 0.40 (hexane/ethyl acetate, 7:3 v/v)

^{1}H NMR (500 MHz, $CDCl_3$) δ 8.01 – 7.94 (m, 2H), 7.65 (td, *J* = 7.3, 1.5 Hz, 1H), 7.61 – 7.51 (m, 5H), 7.40 (td, *J* = 8.0, 3.0 Hz, 2H), 7.28 (d, *J* = 8.2 Hz, 1H), 7.09 (d, *J* = 8.2 Hz, 1H), 7.05 (d, *J* = 7.9 Hz, 1H), 6.95 (dd, *J* = 8.0, 1.7 Hz, 1H), 6.88 (t, *J* = 7.5 Hz, 1H), 6.57 (dt, *J* = 7.8, 1.9 Hz, 1H), 6.53 (s, 1H), 4.48 (d, *J* = 9.8 Hz, 1H), 4.37 (dd, *J* = 9.6, 1.2 Hz, 1H), 2.26 – 2.20 (m, 1H), 2.20 (s, 3H), 0.90 (d, *J* = 6.9 Hz, 3H), 0.81 (d, *J* = 6.9 Hz, 3H).

^{13}C NMR (126 MHz, $CDCl_3$) δ 166.1 (d, $J_{c\text{-}p}$ = 17.6 Hz), 161.6, 152.2 (d, $J_{c\text{-}p}$ = 4.2 Hz), 147.2, 136.7 (d, $J_{c\text{-}p}$ = 11.7 Hz), 133.0 (d, $J_{c\text{-}p}$ = 2.8 Hz), 133.0 (d, $J_{c\text{-}p}$ = 2.8 Hz), 131.8 (d, $J_{c\text{-}p}$ = 9.0 Hz), 131.6 (d, $J_{c\text{-}p}$ = 9.0 Hz), 129.9 (d, $J_{c\text{-}p}$ = 3.0 Hz), 129.8 (d, $J_{c\text{-}p}$ = 3.7 Hz), 129.4 (d, $J_{c\text{-}p}$ = 11.7 Hz), 129.0 (d, $J_{c\text{-}p}$ = 97.9 Hz), 128.6 (d, $J_{c\text{-}p}$ = 12.0 Hz), 128.2 (d, $J_{c\text{-}p}$ = 100.7 Hz), 127.9, 126.5, 124.4 (d, $J_{c\text{-}p}$ = 2.7 Hz), 121.9, 117.8 (d, $J_{c\text{-}p}$ = 2.6 Hz), 115.3 (d, $J_{c\text{-}p}$ = 4.9 Hz), 46.4, 42.3 (d, $J_{c\text{-}p}$ = 65.1 Hz), 26.6, 23.0, 22.9, 20.7.

^{31}P NMR (162 MHz, $CDCl_3$) δ 30.77.

HRMS (ESI-TOF) m/z: $[M+Na]^+$ Calcd. for $C_{32}H_{29}NaO_5P$ 547.1645; Found 547.1619.

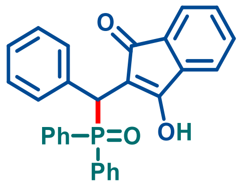

7a

2-((diphenylphosphoryl)(phenyl)methyl)-3-hydroxy-1H-inden-1-one

Physical Appearance: Yellow solid (Mp- 205-207 °C)

Yield: 91% (39.7 mg)

***R_f*:** 0.75 (hexane/ethyl acetate, 7:3 v/v)

^{1}H NMR (500 MHz, $CDCl_3$) δ 14.25 (s, 1H), 7.95 – 7.88 (m, 2H), 7.53 (m, 3H), 7.46 – 7.37 (m, 3H), 7.34 (dd, *J* = 6.3, 4.7 Hz, 3H), 7.30 (td, *J* = 7.8, 3.2 Hz, 2H), 7.27 – 7.22 (m, 3H), 7.19 – 7.11 (m, 3H), 5.02 (d, *J* = 9.0 Hz, 1H).

^{13}C NMR (126 MHz, CDCl$_3$) δ 193.7 (d, $J_{c\text{-}p}$ = 9.1 Hz), 175.6 (d, $J_{c\text{-}p}$ = 5.5 Hz), 140.0, 135.4 (d, $J_{c\text{-}p}$ = 4.6 Hz), 132.8 (d, $J_{c\text{-}p}$ = 2.8 Hz), 132.7 (d, $J_{c\text{-}p}$ = 60.2 Hz), 132.4, 131.3 (d, $J_{c\text{-}p}$ = 9.7 Hz), 131.0 (d, $J_{c\text{-}p}$ = 9.2 Hz), 130.3 (d, $J_{c\text{-}p}$ = 23.7 Hz), 130.0, 129.6 (d, $J_{c\text{-}p}$ = 4.9 Hz), 129.5, 129.2 (d, $J_{c\text{-}p}$ = 11.8 Hz), 128.7, 128.5, 128.4 (d, $J_{c\text{-}p}$ = 14.8 Hz), 127.3 (d, $J_{c\text{-}p}$ = 2.6 Hz), 120.8, 119.3, 105.1 (d, $J_{c\text{-}p}$ = 3.9 Hz), 41.8 (d, $J_{c\text{-}p}$ = 66.6 Hz).

^{31}P NMR (162 MHz, CDCl$_3$) δ 43.27.

HRMS (ESI-TOF) m/z: [M +Na]$^+$ Calcd. for $C_{28}H_{21}NaO_3P$ 459.1121; Found 459.1106

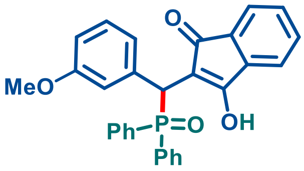

7b

2-((diphenylphosphoryl)(3-methoxyphenyl)methyl)-3-hydroxy-1H-inden-1-one

Physical Appearance: Yellow sticky solid

Yield: 91% (42.4 mg)

***R$_f$*:** 0.75 (hexane/ethyl acetate, 7:3 v/v)

^{1}H NMR (400 MHz, CDCl$_3$) δ 14.18 (s, 1H), 7.99 – 7.81 (m, 2H), 7.55 – 7.44 (m, 3H), 7.43 – 7.36 (m, 3H), 7.35 – 7.25 (m, 5H), 7.21 (m, 1H), 7.04 (t, J = 8.0 Hz, 1H), 6.84 (m, 1H), 6.71 (d, J = 2.2 Hz, 1H), 6.67 (m, 1H), 4.96 (d, J = 9.1 Hz, 1H), 3.60 (s, 3H).

^{13}C NMR (101 MHz, CDCl$_3$) δ 193.7 (d, $J_{c\text{-}p}$ = 9.0 Hz), 175.7 (d, $J_{c\text{-}p}$ = 5.6 Hz), 159.4 (d, $J_{c\text{-}p}$ = 2.1 Hz), 140.0, 136.7 (d, $J_{c\text{-}p}$ = 4.5 Hz), 132.9, 132.8 (d, $J_{c\text{-}p}$ = 2.9 Hz), 132.4 (d, $J_{c\text{-}p}$ = 2.9 Hz), 132.4, 131.3 (d, $J_{c\text{-}p}$ = 9.8 Hz), 130.9 (d, $J_{c\text{-}p}$ = 9.1 Hz), 130.0, 130.0 (d, $J_{c\text{-}p}$ = 98.5 Hz), 129.4 (d, $J_{c\text{-}p}$ = 2.3 Hz), 129.22 (d, $J_{c\text{-}p}$ = 11.7 Hz), 129.18 (d, $J_{c\text{-}p}$ = 102.8 Hz), 128.4 (d, $J_{c\text{-}p}$ = 12.5 Hz), 122.0 (d, $J_{c\text{-}p}$ = 5.1 Hz), 120.7, 119.3, 114.8 (d, $J_{c\text{-}p}$ = 4.9 Hz), 113.4 (d, $J_{c\text{-}p}$ = 2.6 Hz), 105.0 (d, $J_{c\text{-}p}$ = 4.0 Hz), 55.1, 41.8 (d, $J_{c\text{-}p}$ = 66.5 Hz).

^{31}P NMR (162 MHz, CDCl$_3$) δ 43.19.

HRMS (ESI-TOF) m/z: [M +Na]$^+$ Calcd. for $C_{29}H_{23}NaO_4P$ 489.1226; Found 489.1207

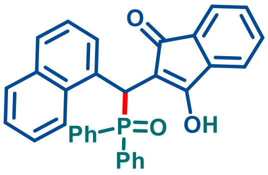

7c

2-((diphenylphosphoryl)(naphthalen-1-yl)methyl)-3-hydroxy-1H-inden-1-one

Physical Appearance: Light Yellow solid (Mp: 222-225 °C)

Yield: 93% (45.2 mg)

***R_f*:** 0.75 (hexane/ethyl acetate, 7:3 v/v)

^{1}H NMR (400 MHz, $CDCl_3$) δ 14.33 (s, 1H), 8.08 (dd, *J* = 6.2, 3.9 Hz, 1H), 8.04 – 7.94 (m, 3H), 7.73 – 7.63 (m, 2H), 7.66 – 7.48 (m, 3H), 7.45 (t, *J* = 7.8 Hz, 1H), 7.37 – 7.26 (m, 5H), 7.26 – 7.15 (m, 1H), 7.19 – 7.05 (m, 3H), 6.94 (td, *J* = 7.8, 3.4 Hz, 2H), 5.95 (d, *J* = 9.4 Hz, 1H).

^{13}C NMR (101 MHz, $CDCl_3$) δ 193.9 (d, J_{c-p} = 9.0 Hz), 176.0 (d, J_{c-p} = 5.5 Hz), 140.3, 133.6, 132.9 (d, J_{c-p} = 2.9 Hz), 132.8, 132.4, 132.2 (d, J_{c-p} = 2.9 Hz), 132.1 (d, J_{c-p} = 4.2 Hz), 131.4 (d, J_{c-p} = 5.2 Hz), 131.2 (d, J_{c-p} = 2.6 Hz), 131.1 (d, J_{c-p} = 3.6 Hz), 130.05, 129.98 (d, J_{c-p} = 98.4 Hz), 129.3 (d, J_{c-p} = 11.8 Hz), 128.9 (d, J_{c-p} = 99.9 Hz), 128.6, 128.3, 128.1 (d, J_{c-p} = 5.4 Hz), 128.0 (d, J_{c-p} = 12.3 Hz), 126.1, 125.6 (d, J_{c-p} = 2.7 Hz), 125.3, 123.0, 120.8, 119.3, 106.1 (d, J_{c-p} = 4.0 Hz), 35.8 (d, J_{c-p} = 67.3 Hz).

^{31}P NMR (162 MHz, $CDCl_3$) δ 43.22.

HRMS (ESI-TOF) m/z: $[M + H]^+$ Calcd. for $C_{32}H_{24}O_3P$ 487.1458; Found 487.1442

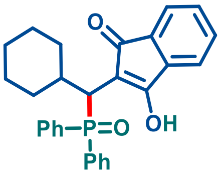

7d

2-(cyclohexyl(diphenylphosphoryl)methyl)-3-hydroxy-1H-inden-1-one

Physical Appearance: Yellow sticky solid

Yield: 89% (39.3 mg)

***R_f*:** 0.70 (hexane/ethyl acetate, 7:3 v/v)

^{1}H NMR (400 MHz, $CDCl_3$) δ 13.68 (s, 1H), 7.86 – 7.67 (m, 4H), 7.62 – 7.48 (m, 3H), 7.46 – 7.30 (m, 5H), 7.27 – 7.21 (m, 2H), 3.72 (dd, J = 9.2, 3.2 Hz, 1H), 2.30 – 2.19 (m, 1H), 1.79 – 1.61 (m, 2H), 1.60 – 1.40 (m, 3H), 1.16 (m, 1H), 1.07 – 0.90 (m, 4H).

^{13}C NMR (101 MHz, $CDCl_3$) δ 194.9 (d, $J_{c\text{-}p}$ = 8.9 Hz), 177.0 (d, $J_{c\text{-}p}$ = 5.6 Hz), 140.1, 133.0, 132.5 (d, $J_{c\text{-}p}$ = 2.9 Hz), 132.35, 132.29 (d, $J_{c\text{-}p}$ = 2.7 Hz), 131.6 (d, $J_{c\text{-}p}$ = 99.7 Hz), 131.0 (d, $J_{c\text{-}p}$ = 9.8 Hz), 130.9, 130.4 (d, $J_{c\text{-}p}$ = 9.1 Hz), 129.9, 129.1 (d, $J_{c\text{-}p}$ = 5.8 Hz), 129.0 (d, $J_{c\text{-}p}$ = 5.5 Hz), 120.7, 119.1, 102.5 (d, $J_{c\text{-}p}$ = 4.3 Hz), 40.3, 38.9 (d, $J_{c\text{-}p}$ = 69.0 Hz), 33.3 (d, $J_{c\text{-}p}$ = 12.8 Hz), 30.1 (d, $J_{c\text{-}p}$ = 2.1 Hz), 26.6 (d, $J_{c\text{-}p}$ = 1.7 Hz), 26.3, 25.7.

^{31}P NMR (162 MHz, $CDCl_3$) δ 45.04.

HRMS (ESI-TOF) m/z: $[M +Na]^+$ Calcd. for $C_{28}H_{27}NaO_3P$ 465.1590; Found 465.1577.

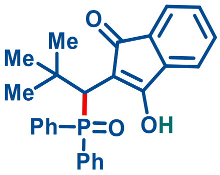

7e

2-(1-(diphenylphosphoryl)-2,2-dimethylpropyl)-3-hydroxy-1H-inden-1-one

Physical Appearance: Light yellow sticky solid

Yield: 88% 36.6 mg)

***R_f*:** 0.70 (hexane/ethyl acetate, 7:3 v/v)

^{1}H NMR (500 MHz, $CDCl_3$) δ 13.75 (s, 1H), 7.92 – 7.84 (m, 2H), 7.80 – 7.72 (m, 2H), 7.51 (m, 3H), 7.41 – 7.28 (m, 5H), 7.26 – 7.19 (m, 2H), 3.82 (d, J = 8.5 Hz, 1H), 0.99 (s, 9H).

^{13}C NMR (126 MHz, $CDCl_3$) δ 194.6 (d, $J_{c\text{-}p}$ = 8.1 Hz), 176.0 (d, $J_{c\text{-}p}$ = 5.8 Hz), 139.9, 133.4 (d, $J_{c\text{-}p}$ = 101.1 Hz), 132.9 (d, $J_{c\text{-}p}$ = 93.1 Hz), 132.4, 132.2, 132.1 (d, $J_{c\text{-}p}$ = 3.1 Hz), 131.7 (d, $J_{c\text{-}p}$ = 2.6 Hz), 131.0 (d, $J_{c\text{-}p}$ = 10.2 Hz), 129.8, 129.4 (d, $J_{c\text{-}p}$ = 8.7 Hz), 128.9 (d, $J_{c\text{-}p}$ = 10.5 Hz), 128.8 (d, $J_{c\text{-}p}$ = 10.2 Hz), 120.6, 118.9, 105.0 (d, $J_{c\text{-}p}$ = 4.0 Hz), 43.7 (d, $J_{c\text{-}p}$ = 66.5 Hz), 38.7, 30.5, 30.4.

^{31}P NMR (202 MHz, $CDCl_3$) δ 41.23.

HRMS (ESI-TOF) m/z: $[M +Na]^+$ Calcd. for $C_{26}H_{25}NaO_3P$ 439.1434; Found 439.1418.

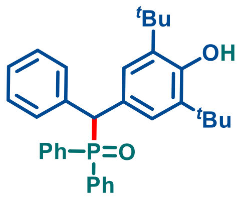

9a

((3,5-di-tert-butyl-4-hydroxyphenyl)(phenyl)methyl)diphenylphosphine oxide

Physical Appearance: White solid (Mp: 220-222 °C)

Yield: 81% (40.2 mg)

***R_f*:** 0.40 (hexane/ethyl acetate, 7:3 v/v)

^{1}H NMR (400 MHz, CDCl$_3$) δ 7.73 (m, 2H), 7.61 (dt, *J* = 8.1, 1.3 Hz, 2H), 7.55 – 7.47 (m, 2H), 7.44 – 7.34 (m, 4H), 7.33 – 7.27 (m, 2H), 7.24 (dd, *J* = 8.3, 6.6 Hz, 2H), 7.19 – 7.14 (m, 1H), 7.11 (d, *J* = 1.8 Hz, 2H), 5.08 (s, 1H), 4.68 (d, *J* = 9.7 Hz, 1H), 1.32 (s, 18H).

^{13}C NMR (101 MHz, CDCl$_3$) δ 152.7 (d, J_{c-p} = 2.5 Hz), 137.7 (d, J_{c-p} = 3.9 Hz), 135.5 (d, J_{c-p} = 1.7 Hz), 133.1 (d, J_{c-p} = 98.6 Hz), 132.5 (d, J_{c-p} = 95.5 Hz), 131.5 (d, J_{c-p} = 8.5 Hz), 131.38 (d, J_{c-p} = 8.6 Hz), 131.36, 131.1 (d, J_{c-p} = 2.8 Hz), 129.9 (d, J_{c-p} = 7.2 Hz), 128.5, 128.3 (d, J_{c-p} = 11.4 Hz), 127.9 (d, J_{c-p} = 11.5 Hz), 126.8, 126.7, 126.6 (d, J_{c-p} = 5.5 Hz), 53.6 (d, J_{c-p} = 66.1 Hz), 34.2, 30.2.

^{31}P NMR (162 MHz, CDCl$_3$) δ 31.23.

HRMS (ESI-TOF) m/z: [M +H]$^+$ Calcd. for $C_{33}H_{38}O_2P$ 497.2604; Found 497.2594.

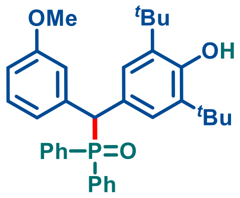

9b

((3,5-di-tert-butyl-4-hydroxyphenyl)(3-methoxyphenyl)methyl)diphenylphosphine oxide

Physical Appearance: Light Yellow solid (Mp: 137-138 °C)

Yield: 84% (44.2 mg)

***R_f*:** 0.40 (hexane/ethyl acetate, 7:3 v/v)

^{1}H NMR (500 MHz, CDCl$_3$) δ 7.79 – 7.68 (m, 2H), 7.54 – 7.44 (m, 2H), 7.42 – 7.33 (m, 4H), 7.28 (dd, *J* = 7.7, 2.9 Hz, 1H), 7.20 – 7.16 (m, 2H), 7.13 (t, *J* = 7.8 Hz, 1H), 7.08 (d, *J* = 1.8 Hz, 2H), 6.70 (m, 1H), 4.64 (d, *J* = 9.8 Hz, 1H), 3.71 (s, 3H), 1.30 (s, 18H).
^{13}C NMR (126 MHz, CDCl$_3$) δ 159.5, 152.9 (d, $J_{c\text{-}p}$ = 2.5 Hz), 139.2 (d, $J_{c\text{-}p}$ = 3.8 Hz), 135.6, 133.1 (d, $J_{c\text{-}p}$ = 68.7 Hz), 132.4 (d, $J_{c\text{-}p}$ = 65.7 Hz), 131.6 (d, $J_{c\text{-}p}$ = 8.5 Hz), 131.5, 131.4 (d, $J_{c\text{-}p}$ = 8.6 Hz), 131.2 (d, $J_{c\text{-}p}$ = 2.8 Hz), 129.5, 128.4 (d, $J_{c\text{-}p}$ = 11.3 Hz), 128.0 (d, $J_{c\text{-}p}$ = 11.4 Hz), 126.8 (d, $J_{c\text{-}p}$ = 5.9 Hz), 126.5 (d, $J_{c\text{-}p}$ = 5.5 Hz), 122.4 (d, $J_{c\text{-}p}$ = 7.3 Hz), 115.1 (d, $J_{c\text{-}p}$ = 7.3 Hz), 113.0, 55.2, 53.6 (d, $J_{c\text{-}p}$ = 66.0 Hz), 34.3, 30.2.
^{31}P NMR (162 MHz, CDCl$_3$) δ 31.19.
HRMS (ESI-TOF) m/z: $[M+H]^+$ Calcd. for $C_{34}H_{40}O_3P$ 527.2710; Found 527.2693.

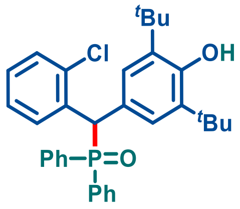

9c

((2-chlorophenyl)(3,5-di-tert-butyl-4-hydroxyphenyl)methyl)diphenylphosphine oxide

Physical Appearance: Light Yellow solid (Mp: 182-184 ^{o}C)

Yield: 74% (39.2 mg)

***R_f*:** 0.40 (hexane/ethyl acetate, 7:3 v/v)

^{1}H NMR (400 MHz, CDCl$_3$) δ 8.46 (dt, *J* = 7.9, 1.7 Hz, 1H), 7.79 m, 2H), 7.51 – 7.34 (m, 6H), 7.28 (m, 3H), 7.28 – 7.17 (m, 1H), 7.14 (d, *J* = 1.8 Hz, 2H), 7.12 – 7.04 (m, 1H), 5.30 (d, *J* = 9.4 Hz, 1H), 5.09 (s, 1H), 1.45 – 1.22 (m, 18H).
^{13}C NMR (101 MHz, CDCl$_3$) δ 153.0 (d, $J_{c\text{-}p}$ = 2.2 Hz), 136.2, 135.6, 134.1 (d, $J_{c\text{-}p}$ = 10.4 Hz), 133.1 (d, $J_{c\text{-}p}$ = 100.9 Hz), 132.2 (d, $J_{c\text{-}p}$ = 96.1 Hz), 131.7 (d, $J_{c\text{-}p}$ = 2.7 Hz), 131.4, 131.35 (d, $J_{c\text{-}p}$ = 5.3 Hz), 131.26 (d, $J_{c\text{-}p}$ = 5.0 Hz), 129.5, 128.5 (d, $J_{c\text{-}p}$ = 11.4 Hz), 128.2, 128.1, 128.0, 127.3, 127.2 (d, $J_{c\text{-}p}$ = 5.8 Hz), 125.1 (d, $J_{c\text{-}p}$ = 5.4 Hz), 48.1 (d, $J_{c\text{-}p}$ = 67.7 Hz), 34.3, 30.3.
^{31}P NMR (162 MHz, CDCl$_3$) δ 32.68.
HRMS (ESI-TOF) m/z: $[M+Na]^+$ Calcd. for $C_{33}H_{36}ClNaO_2P$ 553.2034; Found 553.2013.

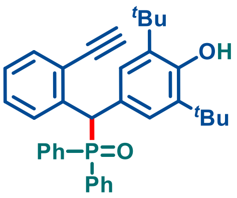

9d

((3,5-di-tert-butyl-4-hydroxyphenyl)(2-ethynylphenyl)methyl)diphenylphosphine oxide

Physical Appearance: Yellow sticky solid

Yield: 69% (35.9 mg)

R_f: 0.40 (hexane/ethyl acetate, 7:3 v/v)

^{1}H NMR (400 MHz, $CDCl_3$) δ 8.41 (dt, *J* = 8.0, 1.4 Hz, 1H), 7.83 (m, 2H), 7.53 – 7.44 (m, 2H), 7.38 (m, 5H), 7.32 – 7.26 (m, 3H), 7.22 (d, *J* = 1.8 Hz, 2H), 7.10 (m, 1H), 5.46 (d, *J* = 9.0 Hz, 1H), 5.10 (s, 1H), 3.43 (s, 1H), 1.34 (s, 18H).

^{13}C NMR (101 MHz, $CDCl_3$) δ 152.9 (d, $J_{c\text{-}p}$ = 2.4 Hz), 140.8 (d, $J_{c\text{-}p}$ = 2.5 Hz), 135.5 (d, $J_{c\text{-}p}$ = 1.7 Hz), 133.3 (d, $J_{c\text{-}p}$ = 100.1 Hz), 133.0, 132.2 (d, $J_{c\text{-}p}$ = 96.1 Hz), 131.5 (d, $J_{c\text{-}p}$ = 2.7 Hz), 131.29, 131.28 (d, $J_{c\text{-}p}$ = 17.0 Hz), 131.2 (d, $J_{c\text{-}p}$ = 2.8 Hz), 129.6 (d, $J_{c\text{-}p}$ = 5.2 Hz), 129.5, 128.4 (d, $J_{c\text{-}p}$ = 11.3 Hz), 128.0 (d, $J_{c\text{-}p}$ = 11.7 Hz), 127.1 (d, $J_{c\text{-}p}$ = 5.9 Hz), 126.7, 126.0 (d, $J_{c\text{-}p}$ = 5.5 Hz), 122.1 (d, $J_{c\text{-}p}$ = 9.9 Hz), 82.7, 81.9, 49.5 (d, $J_{c\text{-}p}$ = 67.5 Hz), 34.3, 30.3.

^{31}P NMR (162 MHz, $CDCl_3$) δ 32.66.

HRMS (ESI-TOF) m/z: $[M +H]^+$ Calcd. for $C_{35}H_{38}O_2P$ 521.2604; Found 521.2588.

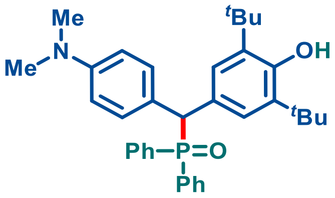

9e

((3,5-di-tert-butyl-4-hydroxyphenyl)(4-(dimethylamino)phenyl)methyl)diphenylphosphine oxide

Physical Appearance: White solid (Mp: 210-212 °C)

Yield: 78% (42.0 mg)

R_f: 0.35 (hexane/ethyl acetate, 7:3 v/v)

^{1}H NMR (400 MHz, CDCl$_3$) δ 7.76 – 7.67 (m, 2H), 7.50 – 7.38 (m, 5H), 7.35 (m, 3H), 7.26 (m, 2H), 7.06 (d, J = 1.9 Hz, 2H), 6.60 (d, J = 8.9 Hz, 1H), 5.00 (s, 1H), 4.57 (d, J = 10.4 Hz, 1H), 2.86 (s, 6H), 1.89 (s, 1H), 1.29 (s, 18H).

^{13}C NMR (101 MHz, CDCl$_3$) δ 152.6 (d, $J_{c\text{-}p}$ = 2.7 Hz), 149.5, 135.4 (d, $J_{c\text{-}p}$ = 1.7 Hz), 133.7 (d, $J_{c\text{-}p}$ = 65.7 Hz), 132.8 (d, $J_{c\text{-}p}$ = 64.0 Hz), 131.7 (d, $J_{c\text{-}p}$ = 8.4 Hz), 131.5 (d, $J_{c\text{-}p}$ = 8.6 Hz), 131.3 (d, $J_{c\text{-}p}$ = 2.7 Hz), 131.0 (d, $J_{c\text{-}p}$ = 2.7 Hz), 130.6 (d, $J_{c\text{-}p}$ = 7.2 Hz), 128.3 (d, $J_{c\text{-}p}$ = 11.3 Hz), 127.9 (d, $J_{c\text{-}p}$ = 11.5 Hz), 127.5 (d, $J_{c\text{-}p}$ = 4.9 Hz), 126.7 (d, $J_{c\text{-}p}$ = 6.0 Hz), 125.5 (d, $J_{c\text{-}p}$ = 3.9 Hz), 112.9, 52.8 (d, $J_{c\text{-}p}$ = 66.5 Hz), 40.7, 34.3, 30.3.

^{31}P NMR (162 MHz, CDCl$_3$) δ 31.43.

HRMS (ESI-TOF) m/z: $[M + H]^+$ Calcd. for $C_{35}H_{43}NO_2P$ 540.3026; Found 540.3011.

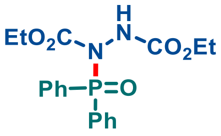

11a

diethyl 1-(diphenylphosphoryl)hydrazine-1,2-dicarboxylate

Physical Appearance: White solid (Mp: 158-162 ^{o}C)

Yield: 74% (27.8 mg)

***R_f*:** 0.2 (hexane/ethyl acetate, 7:3 v/v)

^{1}H NMR (400 MHz, CDCl$_3$) δ 8.22 (dd, J = 13.0, 6.8 Hz, 2H), 7.87 (dd, J = 12.9, 7.2 Hz, 2H), 7.63 – 7.54 (m, 2H), 7.53 – 7.46 (m, 3H), 7.40 m, 2H), 4.11 – 4.01 (m, 4H), 1.11 (t, J = 7.1 Hz, 3H), 0.84 (t, J = 7.2 Hz, 3H).

^{13}C NMR (101 MHz, CDCl$_3$) δ 156.4, 155.0 (d, $J_{c\text{-}p}$ = 11.5 Hz), 132.5, 132.4, 132.14 (d, $J_{c\text{-}p}$ = 10.3 Hz), 132.09 (d, $J_{c\text{-}p}$ = 10.3 Hz), 130.4, 129.9, 128.7 (d, $J_{c\text{-}p}$ = 13.9 Hz), 128.2 (d, $J_{c\text{-}p}$ = 13.5 Hz), 63.7, 62.3, 14.5, 13.7.

^{31}P NMR (162 MHz, CDCl$_3$) δ 32.45.

HRMS (ESI-TOF) m/z: $[M + Na]^+$ Calcd. for $C_{18}H_{21}N_2NaO_5P$ 399.1080; Found 399.1059.

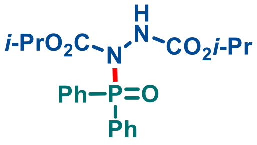

11b

diisopropyl 1-(diphenylphosphoryl)hydrazine-1,2-dicarboxylate

Physical Appearance: White solid (Mp: 205-208 °C)

Yield: 89% (35.9 mg)

***Rf*:** 0.2 (hexane/ethyl acetate, 7:3 v/v)

^{1}H NMR (400 MHz, CDCl$_3$) δ 8.25 (dd, *J* = 12.9, 6.5 Hz, 2H), 7.87 (dd, *J* = 12.8, 7.2 Hz, 2H), 7.64 – 7.42 (m, 4H), 7.41 – 7.28 (m, 3H), 4.90 – 4.71 (m, 2H), 1.17 (d, *J* = 6.3 Hz, 3H), 1.04 (d, *J* = 6.3 Hz, 3H), 0.98 (d, *J* = 6.3 Hz, 3H), 0.71 (d, *J* = 6.5 Hz, 3H).

^{13}C NMR (101 MHz, CDCl$_3$) δ 155.9, 154.5 (d, $J_{c\text{-}p}$ = 11.2 Hz), 132.3 (d, $J_{c\text{-}p}$ = 2.8 Hz), 132.2 (d, $J_{c\text{-}p}$ = 3.0 Hz), 132.1 (d, $J_{c\text{-}p}$ = 2.4 Hz), 132.0, 130.4 (d, $J_{c\text{-}p}$ = 75.6 Hz), 128.7, 128.6 (d, $J_{c\text{-}p}$ = 13.9 Hz), 128.2 (d, $J_{c\text{-}p}$ = 13.4 Hz), 72.2, 70.0, 21.85, 21.79, 21.5, 21.0.

^{31}P NMR (162 MHz, CDCl$_3$) δ 31.69.

HRMS (ESI-TOF) m/z: $[M+Na]^+$ Calcd. for $C_{20}H_{25}N_2NaO_5P$ 427.1393; Found 427.1372.

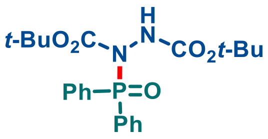

11c

di-tert-butyl 1-(diphenylphosphoryl)hydrazine-1,2-dicarboxylate

Physical Appearance: White sticky solid

Yield: 89% (35.9 mg)

R_f: 0.2 (hexane/ethyl acetate, 7:3 v/v)

^{1}H NMR (500 MHz, CDCl$_3$) δ 8.27 (dd, *J* = 13.0, 7.3 Hz, 2H), 7.85 (dd, *J* = 12.7, 7.7 Hz, 2H), 7.59 – 7.48 (m, 3H), 7.43 (t, *J* = 7.5 Hz, 1H), 7.35 (td, *J* = 7.7, 3.4 Hz, 2H), 6.64 (s, 1H), 1.30 (s, 9H), 1.13 (s, 9H).

^{13}C NMR (126 MHz, CDCl$_3$) δ 155.0, 153.4 (d, $J_{c\text{-}p}$ = 10.5 Hz), 132.1, 132.1 (d, $J_{c\text{-}p}$ = 2.9 Hz), 132.03 (d, $J_{c\text{-}p}$ = 132.7 Hz), 132.02, 131.9, 129.8 (d, $J_{c\text{-}p}$ = 131.4 Hz), 128.5 (d, $J_{c\text{-}p}$ = 13.7 Hz), 128.1 (d, $J_{c\text{-}p}$ = 13.4 Hz), 84.5, 81.5, 28.1, 27.6.

^{31}P NMR (162 MHz, CDCl$_3$) δ 31.11.

HRMS (ESI-TOF) m/z: $[M+Na]^+$ Calcd. for $C_{22}H_{29}N_2NaO_5P$ 455.1706; Found 455.1698.

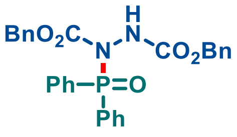

11d

dibenzyl 1-(diphenylphosphoryl)hydrazine-1,2-dicarboxylate

Physical Appearance: White sticky solid

Yield: 82% (41.0 mg)

***R_f*:** 0.2 (hexane/ethyl acetate, 7:3 v/v)

^{1}H NMR (400 MHz, $CDCl_3$) δ 8.33 (s, 1H), 8.21 – 8.11 (m, 2H), 7.89 – 7.79 (m, 2H), 7.55 – 7.47 (m, 1H), 7.46 – 7.37 (m, 3H), 7.34 – 7.25 (m, 5H), 7.24 – 7.10 (m, 5H), 6.94 (d, J = 7.6 Hz, 2H), 5.16 – 4.94 (m, 4H).

^{13}C NMR (101 MHz, $CDCl_3$) δ 156.3, 155.0 (d, $J_{c\text{-}p}$ = 11.6 Hz), 135.8, 134.5, 132.4 (d, $J_{c\text{-}p}$ = 2.8 Hz), 132.3 (d, $J_{c\text{-}p}$ = 3.0 Hz), 132.0 (d, $J_{c\text{-}p}$ = 10.5 Hz), 132.0 (d, $J_{c\text{-}p}$ = 10.7 Hz), 130.4 (d, $J_{c\text{-}p}$ = 131.6 Hz), 128.9 (d, $J_{c\text{-}p}$ = 131.2 Hz), 128.6 (d, $J_{c\text{-}p}$ = 14.0 Hz), 128.4 (d, $J_{c\text{-}p}$ = 15.7 Hz), 128.2 (d, $J_{c\text{-}p}$ = 13.3 Hz), 128.1, 128.0, 127.7, 69.2, 67.4.

^{31}P NMR (162 MHz, $CDCl_3$) δ 33.07.

HRMS (ESI-TOF) m/z: $[M+Na]^+$ Calcd. for $C_{28}H_{25}N_2NaO_5P$ 523.1393; Found 523.1378.

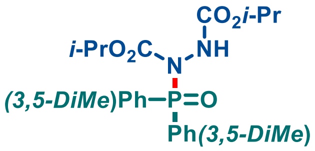

11e

diisopropyl 1-(bis(3,5-dimethylphenyl)phosphoryl)hydrazine-1,2-dicarboxylate

Physical Appearance: Light yellow sticky solid

Yield: 86% (39.6 mg)

***R_f*:** 0.3 (hexane/ethyl acetate, 7:3 v/v)

^{1}H NMR (400 MHz, $CDCl_3$) δ 7.78 (d, J = 13.1 Hz, 2H), 7.47 (d, J = 13.1 Hz, 2H), 7.14 (s, 1H), 7.07 (s, 1H), 6.89 (s, 1H), 4.83 (m, 2H), 2.34 (s, 6H), 2.26 (s, 6H), 1.18 (d, J = 6.3 Hz, 3H), 1.04 (dd, J = 8.4, 6.1 Hz, 6H), 0.78 (d, J = 6.3 Hz, 3H).

^{13}C NMR (101 MHz, $CDCl_3$) δ 155.7, 154.6 (d, $J_{c\text{-}p}$ = 11.1 Hz), 138.2 (d, $J_{c\text{-}p}$ = 14.6 Hz), 137.8 (d, $J_{c\text{-}p}$ = 14.2 Hz), 134.1, 133.8 (d, $J_{c\text{-}p}$ = 3.1 Hz), 131.3 (d, $J_{c\text{-}p}$ = 129.1 Hz), 129.5 (d, $J_{c\text{-}p}$ = 10.4 Hz), 129.2 (d, $J_{c\text{-}p}$ = 129.1 Hz), 72.0, 69.9, 21.9, 21.7, 21.5, 21.4, 21.3, 21.0.

^{31}P NMR (162 MHz, CDCl$_3$) δ 32.83.

HRMS (ESI-TOF) m/z: [M +Na]$^+$ Calcd. for $C_{24}H_{33}N_2NaO_5P$ 483.2019; Found 483.2007.

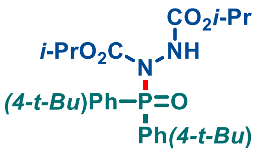

11f

diisopropyl 1-(bis(4-(tert-butyl)phenyl)phosphoryl)hydrazine-1,2-dicarboxylate

Physical Appearance: Colourless sticky solid

Yield: 74% (38.2 mg)

***R$_f$*:** 0.2 (hexane/ethyl acetate, 7:3 v/v)

^{1}H NMR (400 MHz, CDCl$_3$) δ 8.17 (dd, *J* = 12.5, 8.1 Hz, 2H), 7.80 (dd, *J* = 12.4, 8.1 Hz, 2H), 7.51 (dd, *J* = 8.4, 3.3 Hz, 2H), 7.43 – 7.36 (m, 2H), 7.16 – 6.97 (m, 1H), 4.88 – 4.72 (m, 2H), 1.31 (s, 9H), 1.25 (s, 9H), 1.17 (d, *J* = 6.3 Hz, 3H), 1.05 (dd, *J* = 6.3, 2.1 Hz, 3H), 0.96 (dd, *J* = 6.2, 1.4 Hz, 3H), 0.66 (d, *J* = 6.1 Hz, 3H).

^{13}C NMR (101 MHz, CDCl$_3$) δ 155.7, 154.6 (d, J_{c-p} = 11.0 Hz), 132.1 (d, J_{c-p} = 4.8 Hz), 132.0 (d, J_{c-p} = 4.7 Hz), 128.2 (d, J_{c-p} = 134.3 Hz), 126.9, 125.5 (d, J_{c-p} = 13.9 Hz), 125.2 (d, J_{c-p} = 13.5 Hz), 72.0, 69.9, 35.1, 35.0, 31.2, 31.1, 21.9, 21.8, 21.6, 20.8.

^{31}P NMR (162 MHz, CDCl$_3$) δ 32.70.

HRMS (ESI-TOF) m/z: [M]$^+$ Calcd. for $C_{30}H_{56}N_2O_5P$ 555.3932; Found 555.3925.

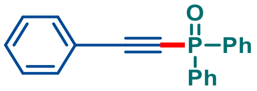

13a

diphenyl(phenylethynyl)phosphine oxide

Physical Appearance: White solid (Mp: 100-102 ^{o}C)

Yield: 88% (26.6 mg)

***R$_f$*:** 0.5 (hexane/ethyl acetate, 7:3 v/v)

^{1}H NMR (500 MHz, CDCl$_3$) δ 7.90 (m, 4H), 7.58 (dd, *J* = 7.1, 1.8 Hz, 2H), 7.56 – 7.52 (m, 2H), 7.49 (m, 4H), 7.43 (m, 1H), 7.36 (m, 2H).

^{13}C NMR (126 MHz, CDCl$_3$) δ 133.0 (d, $J_{c\text{-}p}$ = 122.3 Hz), 132.6 (d, $J_{c\text{-}p}$ = 2.1 Hz), 132.3 (d, $J_{c\text{-}p}$ = 3.0 Hz), 131.0 (d, $J_{c\text{-}p}$ = 11.3 Hz), 130.8, 128.8, 128.7 (d, $J_{c\text{-}p}$ = 4.7 Hz), 119.9 (d, $J_{c\text{-}p}$ = 4.0 Hz), 105.6 (d, $J_{c\text{-}p}$ = 30.1 Hz), 82.9 (d, $J_{c\text{-}p}$ = 170.0 Hz).

^{31}P NMR (202 MHz, CDCl$_3$) δ 8.45.

HRMS (ESI-TOF) m/z: [M +Na]$^+$ Calcd. for $C_{20}H_{15}NaOP$ 325.0753; Found 325.0745.

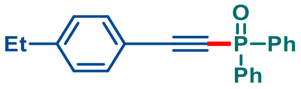

13b

((4-ethylphenyl)ethynyl)diphenylphosphine oxide

Physical Appearance: Light Yellow solid (Mp: 100-101 ^{o}C)

Yield: 87% (28.7 mg)

***R_f*:** 0.5 (hexane/ethyl acetate, 7:3 v/v)

^{1}H NMR (400 MHz, CDCl$_3$) δ 7.95 – 7.85 (m, 4H), 7.57 – 7.44 (m, 8H), 7.20 (d, J = 8.6 Hz, 2H), 2.67 (q, J = 7.6 Hz, 2H), 1.23 (t, J = 7.6 Hz, 3H).

^{13}C NMR (101 MHz, CDCl$_3$) δ 147.7, 133.3 (d, $J_{c\text{-}p}$ = 122.2 Hz), 132.7 (d, $J_{c\text{-}p}$ = 2.0 Hz), 132.3 (d, $J_{c\text{-}p}$ = 3.0 Hz), 131.1 (d, $J_{c\text{-}p}$ = 11.4 Hz), 128.7 (d, $J_{c\text{-}p}$ = 13.5 Hz), 128.3, 117.2, 106.2 (d, $J_{c\text{-}p}$ = 30.6 Hz), 82.3 (d, $J_{c\text{-}p}$ = 171.9 Hz), 29.1, 15.3.

^{31}P NMR (162 MHz, CDCl$_3$) δ 8.38.

HRMS (ESI-TOF) m/z: [M +Na]$^+$ Calcd. for $C_{22}H_{19}NaOP$ 353.1066; Found 353.1055.

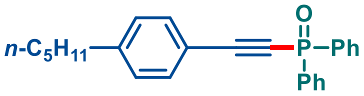

13c

((4-pentylphenyl)ethynyl)diphenylphosphine oxide

Physical Appearance: Light yellow oil

Yield: 89% (26.8 mg)

***R_f*:** 0.5 (hexane/ethyl acetate, 7:3 v/v)

^{1}H NMR (400 MHz, CDCl$_3$) δ 7.95 – 7.84 (m, 4H), 7.59 – 7.43 (m, 8H), 7.22 – 7.15 (m, 2H), 2.66 – 2.58 (m, 2H), 1.66 – 1.54 (m, 2H), 1.40 – 1.21 (m, 4H), 0.88 (t, J = 6.9 Hz, 3H).

^{13}C NMR (101 MHz, CDCl$_3$) δ 146.5, 133.3 (d, $J_{c\text{-}p}$ = 122.1 Hz), 132.6 (d, $J_{c\text{-}p}$ = 2.0 Hz), 132.3 (d, $J_{c\text{-}p}$ = 3.0 Hz), 131.1 (d, $J_{c\text{-}p}$ = 11.3 Hz), 128.8, 128.7, 117.1 (d, $J_{c\text{-}p}$ = 4.0 Hz), 106.2 (d, $J_{c\text{-}p}$ = 30.6 Hz), 82.3 (d, $J_{c\text{-}p}$ = 172.0 Hz), 36.1, 31.5, 30.9, 22.6, 14.1.
^{31}P NMR (162 MHz, CDCl$_3$) δ 8.36.
HRMS (ESI-TOF) m/z: $[M+Na]^+$ Calcd. for $C_{25}H_{25}NaOP$ 353.1066; Found 353.1055.

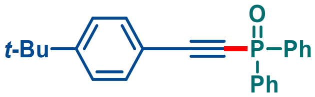

13d

((4-(tert-butyl)phenyl)ethynyl)diphenylphosphine oxide

Physical Appearance: Colourless oil

Yield: 88% (31.5 mg)

***R_f*:** 0.5 (hexane/ethyl acetate, 7:3 v/v)

^{1}H NMR (400 MHz, CDCl$_3$) δ 7.96 – 7.85 (m, 4H), 7.57 – 7.43 (m, 8H), 7.42 – 7.38 (m, 2H), 1.31 (s, 9H).
^{13}C NMR (126 MHz, CDCl$_3$) δ 154.6, 133.3 (d, $J_{c\text{-}p}$ = 122.1 Hz), 132.5 (d, $J_{c\text{-}p}$ = 2.0 Hz), 132.3 (d, $J_{c\text{-}p}$ = 3.0 Hz), 131.1 (d, $J_{c\text{-}p}$ = 11.4 Hz), 128.7 (d, $J_{c\text{-}p}$ = 13.4 Hz), 125.7, 116.9 (d, $J_{c\text{-}p}$ = 4.0 Hz), 106.2 (d, $J_{c\text{-}p}$ = 30.6 Hz), 82.3 (d, $J_{c\text{-}p}$ = 172.0 Hz), 35.2, 31.1.
^{31}P NMR (162 MHz, CDCl$_3$) δ 8.45.
HRMS (ESI-TOF) m/z: $[M+Na]^+$ Calcd. for $C_{24}H_{23}NaOP$ 381.1379; Found 381.1371.

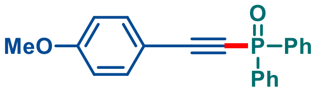

13e

((4-methoxyphenyl)ethynyl)diphenylphosphine oxide

Physical Appearance: Colourless oil

Yield: 83% (27.5 mg)

***R_f*:** 0.5 (hexane/ethyl acetate, 7:3 v/v)

^{1}H NMR (500 MHz, CDCl$_3$) δ 7.90 (dd, J = 13.9, 7.4 Hz, 4H), 7.54 (d, J = 8.5 Hz, 4H), 7.52 – 7.45 (m, 4H), 6.88 (d, J = 8.7 Hz, 2H), 3.83 (s, 3H).

^{13}C NMR (126 MHz, CDCl$_3$) δ 161.6, 134.5 (d, $J_{c\text{-}p}$ = 1.6 Hz), 133.4 (d, $J_{c\text{-}p}$ = 122.2 Hz), 132.3 (d, $J_{c\text{-}p}$ = 2.7 Hz), 131.1 (d, $J_{c\text{-}p}$ = 11.2 Hz), 128.8 (d, $J_{c\text{-}p}$ = 13.5 Hz), 114.4, 111.9 (d, $J_{c\text{-}p}$ = 4.6 Hz), 106.3 (d, $J_{c\text{-}p}$ = 31.0 Hz), 81.8 (d, $J_{c\text{-}p}$ = 173.3 Hz), 55.6.
^{31}P NMR (202 MHz, CDCl$_3$) δ 8.30.
HRMS (ESI-TOF) m/z: [M +Na]$^+$ Calcd. for $C_{21}H_{17}NaOP$ 355.0858; Found 355.0848.

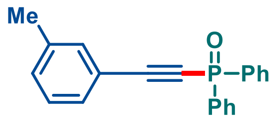

13f

diphenyl(m-tolylethynyl)phosphine oxide
Physical Appearance: White sticky solid

Yield: 74% (27.5 mg)

***R_f*:** 0.5 (hexane/ethyl acetate, 7:3 v/v)

^{1}H NMR (500 MHz, CDCl$_3$) δ 7.88 (m, 4H), 7.64 – 7.54 (m, 3H), 7.54 – 7.45 (m, 5H), 7.46 – 7.40 (m, 1H), 7.32 (d, J = 7.8 Hz, 1H), 2.16 (s, 3H).
^{13}C NMR (126 MHz, CDCl$_3$) δ 134.7, 132.8 (d, $J_{c\text{-}p}$ = 122.4 Hz), 132.6 (d, $J_{c\text{-}p}$ = 2.9 Hz), 132.3 (d, $J_{c\text{-}p}$ = 2.1 Hz), 131.1 (d, $J_{c\text{-}p}$ = 11.4 Hz), 130.8 (d, $J_{c\text{-}p}$ = 1.9 Hz), 130.0, 128.9 (d, $J_{c\text{-}p}$ = 13.6 Hz), 127.3 (d, $J_{c\text{-}p}$ = 303.2 Hz), 121.8 (d, $J_{c\text{-}p}$ = 4.1 Hz), 103.5 (d, $J_{c\text{-}p}$ = 29.2 Hz), 84.2 (d, $J_{c\text{-}p}$ = 166.3 Hz), 31.0.
^{31}P NMR (202 MHz, CDCl$_3$) δ 8.46.
HRMS (ESI-TOF) m/z: [M +Na]$^+$ Calcd. for $C_{21}H_{17}NaOP$ 339.0909; Found 339.0882.

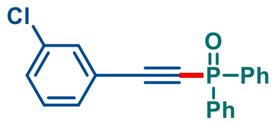

13g

((3-chlorophenyl)ethynyl)diphenylphosphine oxide

Physical Appearance: Light yellow oil

Yield: 81% (27.2 mg)

***R_f*:** 0.5 (hexane/ethyl acetate, 7:3 v/v)

^{1}H NMR (400 MHz, CDCl$_3$) δ 7.95 – 7.81 (m, 4H), 7.63 – 7.54 (m, 3H), 7.54 – 7.46 (m, 5H), 7.42 (m, 1H), 7.31 (t, *J* = 7.9 Hz, 1H).

^{13}C NMR (101 MHz, CDCl$_3$) δ 134.0 (d, $J_{c\text{-}p}$ = 127.6 Hz), 132.5 (d, $J_{c\text{-}p}$ = 2.9 Hz), 132.3 (d, $J_{c\text{-}p}$ = 2.0 Hz), 132.2, 131.2, 131.0, 130.8 (d, $J_{c\text{-}p}$ = 2.0 Hz), 130.0, 128.9 (d, $J_{c\text{-}p}$ = 13.5 Hz), 121.8 (d, $J_{c\text{-}p}$ = 4.0 Hz), 103.5 (d, $J_{c\text{-}p}$ = 29.1 Hz), 84.2 (d, $J_{c\text{-}p}$ = 166.4 Hz).

^{31}P NMR (162 MHz, CDCl$_3$) δ 8.39.

HRMS (ESI-TOF) m/z: [M +Na]$^+$ Calcd. for $C_{20}H_{14}ClNaOP$ 359.0363; Found 359.0355.

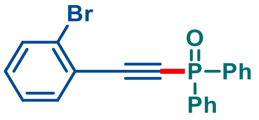

13h

((2-bromophenyl)ethynyl)diphenylphosphine oxide

Physical Appearance: Colourless oil

Yield: 82% (31.1 mg)

***R_f*:** 0.5 (hexane/ethyl acetate, 7:3 v/v)

^{1}H NMR (400 MHz, CDCl$_3$) δ 7.94 – 7.82 (m, 4H), 7.58 – 7.54 (m, 2H), 7.54 – 7.52 (m, 2H), 7.51 – 7.47 (m, 4H), 7.46 – 7.42 (m, 2H).

^{13}C NMR (126 MHz, CDCl$_3$) δ 134.0 (d, $J_{c\text{-}p}$ = 1.9 Hz), 132.7 (d, $J_{c\text{-}p}$ = 122.5 Hz), 133.1, 132.5 (d, $J_{c\text{-}p}$ = 2.8 Hz), 132.1, 131.1 (d, $J_{c\text{-}p}$ = 11.3 Hz), 128.8 (d, $J_{c\text{-}p}$ = 13.6 Hz), 128.4, 125.6, 118.9 (d, $J_{c\text{-}p}$ = 4.1 Hz), 104.3 (d, $J_{c\text{-}p}$ = 29.8 Hz), 84.1 (d, $J_{c\text{-}p}$ = 168.0 Hz).

^{31}P NMR (162 MHz, CDCl$_3$) δ 8.54.

HRMS (ESI-TOF) m/z: [M +Na]$^+$ Calcd. for $C_{20}H_{14}ClNaOP$ 402.9858; Found 402.9851.

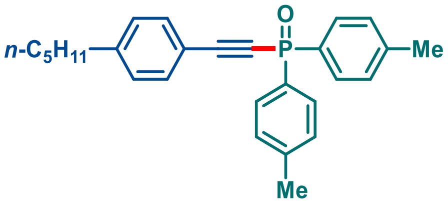

13i

((4-pentylphenyl)ethynyl)di-p-tolylphosphine oxide

Physical Appearance: Colourless oil

Yield: 84% (33.6 mg)

***R_f*:** 0.5 (hexane/ethyl acetate, 7:3 v/v)

^{1}H NMR (500 MHz, CDCl$_3$) δ 7.77 (dd, *J* = 13.6, 8.1 Hz, 4H), 7.58 – 7.44 (m, 2H), 7.28 (dd, *J* = 8.1, 3.0 Hz, 4H), 7.17 (d, *J* = 8.0 Hz, 2H), 2.67 – 2.58 (m, 2H), 2.40 (s, 6H), 1.60 (p, *J* = 7.4 Hz, 2H), 1.31 (m, 4H), 0.88 (t, *J* = 7.0 Hz, 3H).
^{13}C NMR (126 MHz, CDCl$_3$) δ 146.3, 142.8 (d, $J_{c\text{-}p}$ = 2.8 Hz), 132.6 (d, $J_{c\text{-}p}$ = 2.0 Hz), 131.1 (d, $J_{c\text{-}p}$ = 11.6 Hz), 130.3 (d, $J_{c\text{-}p}$ = 124.6 Hz), 129.5 (d, $J_{c\text{-}p}$ = 13.9 Hz), 128.8, 117.3 (d, $J_{c\text{-}p}$ = 4.0 Hz), 105.7 (d, $J_{c\text{-}p}$ = 30.4 Hz), 82.7 (d, $J_{c\text{-}p}$ = 170.4 Hz), 36.1, 31.5, 30.9, 22.6, 21.8, 14.1.
^{31}P NMR (162 MHz, CDCl$_3$) δ 8.85.
HRMS (ESI-TOF) m/z: [M +Na]$^+$ Calcd. for $C_{27}H_{29}NaOP$ 423.1848; Found 423.1834.

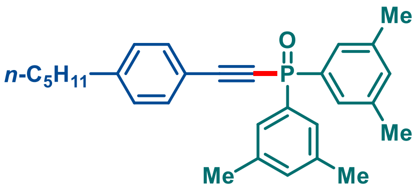

13j

Physical Appearance: Colourless oil

Yield: 68% (29.1 mg)

***R_f*:** 0.5 (hexane/ethyl acetate, 7:3 v/v)

^{1}H NMR (500 MHz, CDCl$_3$) δ 7.54 – 7.44 (m, 6H), 7.25 – 7.14 (m, 4H), 2.75 – 2.56 (m, 2H), 2.35 (s, 12H), 1.65 – 1.55 (m, 2H), 1.31 (m, 4H), 0.88 (t, *J* = 6.9 Hz, 3H).
^{13}C NMR (126 MHz, CDCl$_3$) δ 146.2, 138.4 (d, $J_{c\text{-}p}$ = 14.2 Hz), 134.0 (d, $J_{c\text{-}p}$ = 2.9 Hz), 133.1 (d, $J_{c\text{-}p}$ = 120.7 Hz), 132.6 (d, $J_{c\text{-}p}$ = 2.0 Hz), 128.7 (d, $J_{c\text{-}p}$ = 14.9 Hz), 128.6, 117.4 (d, $J_{c\text{-}p}$ = 4.2 Hz), 105.6 (d, $J_{c\text{-}p}$ = 29.9 Hz), 82.8 (d, $J_{c\text{-}p}$ = 169.4 Hz), 36.1, 31.5, 30.9, 22.6, 21.4, 14.1.
^{31}P NMR (162 MHz, CDCl$_3$) δ 9.20.
HRMS (ESI-TOF) m/z: [M +H]$^+$ Calcd. for $C_{29}H_{34}OP$ 429.2342; Found 429.2366.

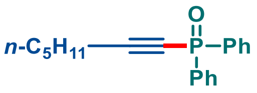

13k

hept-1-yn-1-yldiphenylphosphine oxide
Physical Appearance: Colourless oil

Yield: 84% (24.9 mg)

***R_f*:** 0.6 (hexane/ethyl acetate, 7:3 v/v)

^{1}H NMR (400 MHz, CDCl$_3$) δ 7.82 (m, 4H), 7.59 – 7.37 (m, 6H), 2.44 (td, J = 7.2, 3.6 Hz, 2H), 1.63 (p, J = 7.2 Hz, 2H), 1.45 – 1.27 (m, 4H), 0.88 (t, J = 7.1 Hz, 3H).

^{13}C NMR (101 MHz, CDCl$_3$) δ 133.5 (d, $J_{c\text{-}p}$ = 121.9 Hz), 132.2 (d, $J_{c\text{-}p}$ = 2.9 Hz), 131.0 (d, $J_{c\text{-}p}$ = 11.3 Hz), 128.7 (d, $J_{c\text{-}p}$ = 13.3 Hz), 110.1 (d, $J_{c\text{-}p}$ = 30.7 Hz), 74.9 (d, $J_{c\text{-}p}$ = 175.6 Hz), 31.1, 27.4 (d, $J_{c\text{-}p}$ = 1.8 Hz), 22.2, 19.9 (d, $J_{c\text{-}p}$ = 3.2 Hz), 14.0.

^{31}P NMR (162 MHz, CDCl$_3$) δ 8.27.

HRMS (ESI-TOF) m/z: [M +Na]$^+$ Calcd. for $C_{19}H_{21}NaOP$ 319.1222; Found 319.1213.

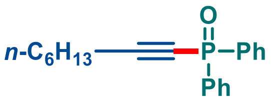

13l

oct-1-yn-1-yldiphenylphosphine oxide

Physical Appearance: Colourless oil

Yield: 84% (19.0 mg)

***R$_f$*:** 0.6 (hexane/ethyl acetate, 7:3 v/v)

^{1}H NMR (500 MHz, CDCl$_3$) δ 7.82 (m, 4H), 7.52 (m, 2H), 7.45 (m, 4H), 2.44 (td, J = 7.1, 3.5 Hz, 2H), 1.62 (q, J = 7.2 Hz, 2H), 1.46 – 1.36 (m, 2H), 1.32 – 1.26 (m, 4H), 0.89 – 0.85 (m, 3H).

^{13}C NMR (126 MHz, CDCl$_3$) δ 133.6 (d, $J_{c\text{-}p}$ = 121.7 Hz), 132.1 (d, $J_{c\text{-}p}$ = 2.9 Hz), 131.0 (d, $J_{c\text{-}p}$ = 11.3 Hz), 128.6 (d, $J_{c\text{-}p}$ = 13.5 Hz), 110.0 (d, $J_{c\text{-}p}$ = 30.7 Hz), 75.0 (d, $J_{c\text{-}p}$ = 175.3 Hz), 31.3, 28.6, 27.6 (d, $J_{c\text{-}p}$ = 1.8 Hz), 22.6, 19.9 (d, $J_{c\text{-}p}$ = 3.1 Hz), 14.1.

^{31}P NMR (202 MHz, CDCl$_3$) δ 7.61.

HRMS (ESI-TOF) m/z: [M +Na]$^+$ Calcd. for $C_{20}H_{23}NaOP$ 333.1379; Found 333.1368.

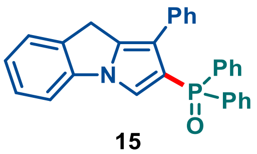

15

diphenyl(1-phenyl-9H-pyrrolo[1,2-a]indol-2-yl)phosphine oxide

Physical Appearance: Light Yellow solid (Mp: 261-264 °C)

Yield: 63% (27.1 mg)

***R$_f$*:** 0.25 (hexane/ethyl acetate, 7:3 v/v)

^{1}H NMR (400 MHz, CDCl$_3$) δ 7.75 (dd, *J* = 12.2, 7.0 Hz, 4H), 7.59 (d, *J* = 7.2 Hz, 2H), 7.42 (t, *J* = 7.3 Hz, 3H), 7.34 (td, *J* = 7.6, 2.7 Hz, 4H), 7.29 (d, *J* = 7.6 Hz, 1H), 7.23 (d, *J* = 7.7 Hz, 1H), 7.16 (dt, *J* = 10.8, 7.5 Hz, 3H), 7.04 (t, *J* = 7.4 Hz, 1H), 6.96 (d, *J* = 4.0 Hz, 1H), 4.02 (s, 2H).

^{13}C NMR (101 MHz, CDCl$_3$) δ 139.8, 135.5 (d, $J_{c\text{-}p}$ = 11.0 Hz), 134.6, 134.3, 133.2, 132.0 (d, $J_{c\text{-}p}$ = 10.0 Hz), 131.4 (d, $J_{c\text{-}p}$ = 2.6 Hz), 128.8, 128.2 (d, $J_{c\text{-}p}$ = 12.1 Hz), 128.1, 127.8, 126.31, 126.26, 124.8, 122.0 (d, $J_{c\text{-}p}$ = 8.6 Hz), 119.5 (d, $J_{c\text{-}p}$ = 21.6 Hz), 117.3 (d, $J_{c\text{-}p}$ = 119.5 Hz), 110.7, 29.7.

^{31}P NMR (202 MHz, CDCl$_3$) δ 21.79.

HRMS (ESI-TOF) m/z: [M +H]$^+$ Calcd. for $C_{29}H_{23}NOP$ 432.1512; Found 432.1496.

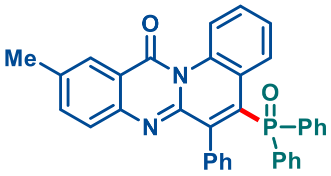

17

5-(diphenylphosphoryl)-10-methyl-6-phenyl-12H-quinolino[2,1-b]quinazolin-12-one

Physical Appearance: Yellow sticky solid

Yield: 78% (41.8 mg)

***R*$_f$:** 0.45 (hexane/ethyl acetate, 7:3 v/v)

^{1}H NMR (500 MHz, CDCl$_3$) δ 8.96 (d, *J* = 8.7 Hz, 1H), 8.26 (s, 1H), 8.02 (d, *J* = 8.1 Hz, 1H), 7.55 (dd, *J* = 8.4, 2.2 Hz, 1H), 7.49 – 7.37 (m, 6H), 7.24 (dd, *J* = 7.5, 1.7 Hz, 1H), 7.21 – 7.09 (m, 8H), 7.01 (t, *J* = 7.7 Hz, 2H), 2.54 (s, 3H).

^{13}C NMR (126 MHz, CDCl$_3$) δ 162.6, 146.9 (d, $J_{c\text{-}p}$ = 8.1 Hz), 146.5 (d, $J_{c\text{-}p}$ = 15.5 Hz), 144.3 (d, $J_{c\text{-}p}$ = 1.5 Hz), 138.0, 136.2, 135.6 (d, $J_{c\text{-}p}$ = 98.9 Hz), 135.5 (d, $J_{c\text{-}p}$ = 6.1 Hz), 134.7 (d, $J_{c\text{-}p}$ = 106.1 Hz), 133.7 (d, $J_{c\text{-}p}$ = 8.4 Hz), 132.9, 131.0 (d, $J_{c\text{-}p}$ = 9.4 Hz), 130.8 (d, $J_{c\text{-}p}$ = 2.7 Hz), 129.0 (d, $J_{c\text{-}p}$ = 5.9 Hz), 128.9, 128.3 (d, $J_{c\text{-}p}$ = 12.4 Hz), 128.2 (d, $J_{c\text{-}p}$ = 11.7 Hz), 127.3, 126.6, 125.7, 125.0 (d, $J_{c\text{-}p}$ = 9.3 Hz), 120.8, 120.2, 21.7.

^{31}P NMR (202 MHz, CDCl$_3$) δ 24.25.

HRMS (ESI-TOF) m/z: [M +H]$^+$ Calcd. for $C_{35}H_{26}N_2O_2P$ 537.1726; Found 537.1711.

10. References

1) J. Wang, Y. Zhou, L. Zhang, Z. Li, X. Chen and H. Liu, *Org. Lett.*, 2013, **15**, 1508–1511.
2) O. Kaumanns, R. Lucius and H. Mayr, *Chemistry*, 2008, **14**, 9675–9682.
3) Y. Hu, Y.-H. He and Z. Guan, *Catal. Commun.*, 2010, **11**, 656–659.
4) Y. Chen, Y. You and Z. Weng, *Org. Chem. Front.*, 2019, **6**, 213–217.
5) K. Okuro and H. Alper, *J. Org. Chem.*, 2012, **77**, 4420–4424.
6) H. Yang, W. Zhang and Q. Liu, *ChemistrySelect*, 2019, **4**, 10819–10827.
7) M. Tavakolian and M. M. Najafpour, *New J Chem*, 2019, **43**, 16437–16440.
8) Y. Zhou, C.-F. Gao, H. Ma, J. Nie, J.-A. Ma and F.-G. Zhang, *Chem. Asian J.*, 2022, **17**, e202200436.
9) Y. Li, H. Zhang, R. Wei and Z. Miao, *Adv. Synth. Catal.*, 2017, **359**, 4158–4164.
10) Sonam, V. N. Shinde and A. Kumar, *J. Org. Chem.*, 2022, **87**, 2651–2661.
11) A. G. Burra, D. Uredi, D. R. Motati, F. R. Fronczek and E. B. Watkins, *European J. Org. Chem.*, 2022, e202200191.
12) A. S. Jadhav, Y. A. Pankhade and R. Vijaya Anand, *J. Org. Chem.*, 2018, **83**, 8615–8626.
13) L. Roiser and M. Waser, *Org. Lett.*, 2017, **19**, 2338–2341.
14) D. J. Fansher and D. R. J. Palmer, *Angew. Chem. Int. Ed.*, 2023, **62**, e202214539.
15) H. Ruan, L.-G. Meng, H. Xu, Y. Liang and L. Wang, *Org. Biomol. Chem.*, 2020, **18**, 1087–1090.
16) Y.-S. Feng, Z.-Q. Xu, L. Mao, F.-F. Zhang and H.-J. Xu, *Org. Lett.*, 2013, **15**, 1472–1475.
17) Z.-C. Miao, D. Wang, Y.-M. Zhang, Z.-K. Jin, F. Liu, F.-F. Wang and H. Yang, *Liq. Cryst.*, 2012, **39**, 1291–1296.
18) N. V. S. D. K. Bhupathiraju, M. Sayeedi, W. Rizvi, S. Singh, J. D. Batteas and C. Michael Drain, *Tetrahedron Lett.*, 2018, **59**, 3629–3631.

19) A. Verlee, T. Heugebaert, T. van der Meer, P. Kerchev, K. Van Hecke, F. Van Breusegem and C. V. Stevens, *ACS Catal.*, 2019, **9**, 7862–7869.

20) J. Wu, B. Qian, Y. Liu and Y. Shang, *ChemistrySelect*, 2020, **5**, 10269–10275.

21) F.-L. Zeng, Z.-Y. Zhang, P.-C. Yin, F.-K. Cheng, X.-L. Chen, L.-B. Qu, Z.-Y. Cao and B. Yu, *Org. Lett.*, 2022, **24**, 7912–7917.

22) Y. Chen, Z. Yu, Z. Jiang, J.-P. Tan, J.-H. Wu, Y. Lan, X. Ren and T. Wang, *ACS Catal.*, 2021, **11**, 14168–14180.

23) M. A. Cismesia and T. P. Yoon, *Chem. Sci.*, 2015, **6**, 5426–5434.

24) C.-G. Hatchard and C.-A. Parker, *Proc. R. Soc. Lond.*, 1956, **235**, 518–536.

25) H.-J. Kuhn, S.-E. Braslavsky and R. Schmidt, *Pure Appl. Chem.*, 2004, **76**, 2105–2146.

11. X-ray Crystallography data for the compound

Single crystals of all compounds were mounted on a Cryoloop with a drop of Paratone oil and positioned in the cold nitrogen stream on a Rigaku Saturn724+ (2x2 bin mode) diffractometer for **5a**, **7c** and **9b**. The data collections were performed at 150 K using a graphite monochromated MoKα (λ = 0.71073) radiation source for compounds 7 and 51. The data were reduced using CrysalisPro Red 171.41_64.93a software. The structures were solved using Olex21 with the ShelXT2 structure solution program using intrinsic phasing and refined with the SHELXL3 refinement package using least-squares minimization. These data can be obtained free of charge from The Cambridge Crystallographic Data Centre via www.ccdc.cam.ac.uk/data_request/cif.

a. X-ray Crystallography data for the compound of 5a

Crystal of the compound **5a** was obtained after slow evaporation of chloroform solvent. Molecular structure of **5a** with 50% ellipsoid probability.

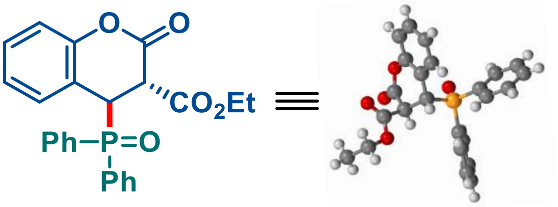

5a, 91%   **5a,** *CCDC:* 2309164

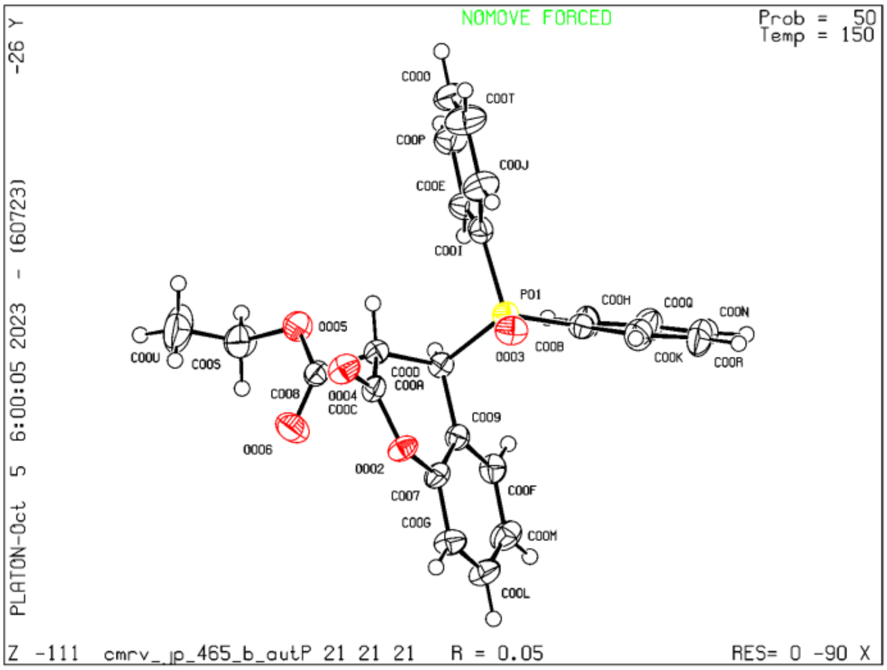

Figure S12. Molecular structure of **5a** with 50% ellipsoid probability.

Table 1 Crystal data and structure refinement for CMRV_JP_465_B_autored.	
Identification code	CMRV_JP_465_B_autored
Empirical formula	$C_{24}H_{21}O_5P$
Formula weight	420.38
Temperature/K	150.00(10)
Crystal system	orthorhombic
Space group	$P2_12_12_1$
a/Å	8.7082(5)
b/Å	11.7522(5)
c/Å	20.0325(12)
α/°	90
β/°	90
γ/°	90
Volume/Å^3	2050.14(19)
Z	4
ρ_{calc}g/cm^3	1.362
μ/mm^{-1}	0.168
F(000)	880.0
Crystal size/mm^3	0.0875 × 0.0652 × 0.0367
Radiation	MoKα (λ = 0.71073)
2Θ range for data collection/°	4.018 to 49.978
Index ranges	$-10 \leq h \leq 10$, $-13 \leq k \leq 13$, $-23 \leq l \leq 23$
Reflections collected	19704
Independent reflections	3612 [R_{int} = 0.1058, R_{sigma} = 0.0677]
Data/restraints/parameters	3612/0/272
Goodness-of-fit on F^2	0.881
Final R indexes [I>=2σ (I)]	R_1 = 0.0525, wR_2 = 0.1323
Final R indexes [all data]	R_1 = 0.0616, wR_2 = 0.1491
Largest diff. peak/hole / e Å^{-3}	0.33/-0.36
Flack parameter	0.09(11)

b. X-ray Crystallography data for the compound of 7c

Crystal of the compound **7c** was obtained after slow evaporation of chloroform solvent. Molecular structure of **7c** with 50% ellipsoid probability.

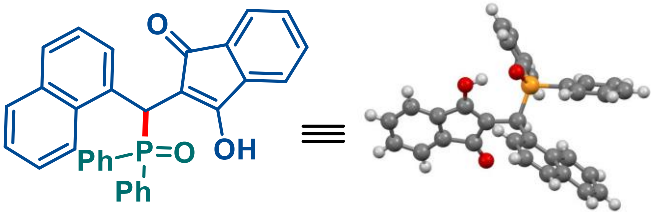

7c, 93% **7c**, *CCDC:* 2309165

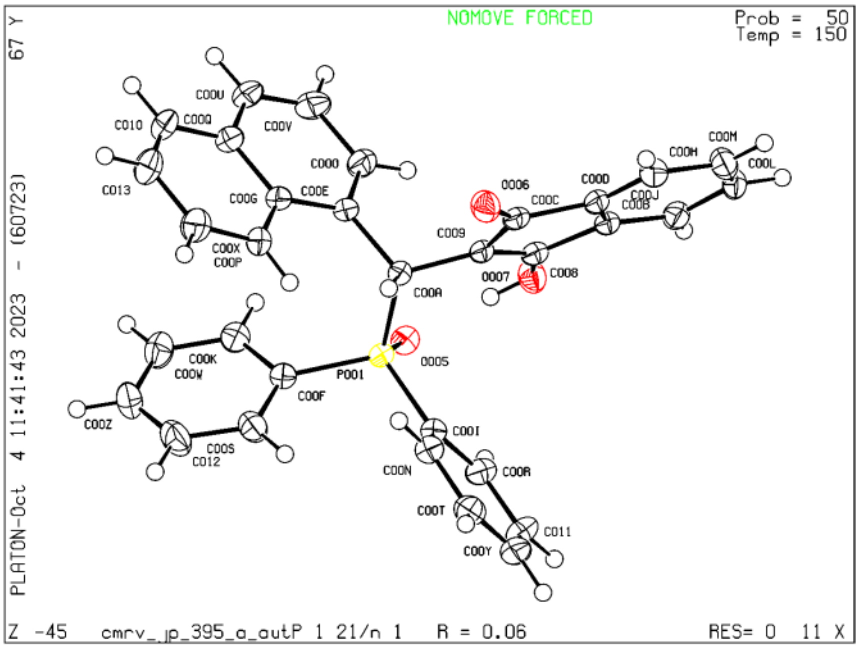

Figure S13. Molecular structure of **7c** with 50% ellipsoid probability.

**Table 2 Crystal data and structure refinement for CMRV_JP_395_A_autored.**	
Identification code	CMRV_JP_395_A_autored
Empirical formula	$C_{33}H_{24}Cl_3O_3P$
Formula weight	605.84
Temperature/K	150.00(10)
Crystal system	monoclinic
Space group	$P2_1/n$
a/Å	12.6015(3)
b/Å	16.8085(2)

c/Å	14.3074(3)
α/°	90
β/°	112.713(2)
γ/°	90
Volume/Å^3	2795.47(10)
Z	4
ρ_{calc}g/cm^3	1.440
μ/mm^{-1}	0.420
F(000)	1248.0
Crystal size/mm^3	0.27 × 0.24 × 0.23
Radiation	Mo Kα (λ = 0.71073)
2Θ range for data collection/°	3.924 to 50
Index ranges	$-14 \leq h \leq 14$, $-19 \leq k \leq 19$, $-17 \leq l \leq 17$
Reflections collected	53767
Independent reflections	4896 [R_{int} = 0.0756, R_{sigma} = 0.0305]
Data/restraints/parameters	4896/0/326
Goodness-of-fit on F^2	1.062
Final R indexes [I>=2σ (I)]	R_1 = 0.0597, wR_2 = 0.1533
Final R indexes [all data]	R_1 = 0.0681, wR_2 = 0.1606
Largest diff. peak/hole / e Å^{-3}	0.38/-0.43

c. X-ray Crystallography data for the compound of 9b

Crystal of the compound **9b** was obtained after slow evaporation of acetonitrile solvent. Molecular structure of **9b** with 50% ellipsoid probability.

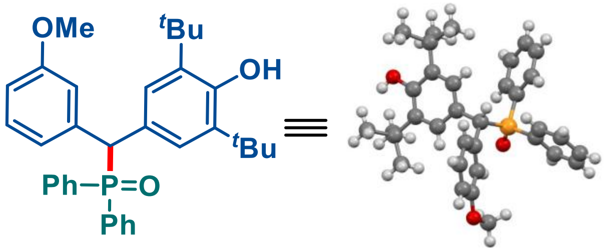

9b, 83% **9b,** *CCDC:* 2309163

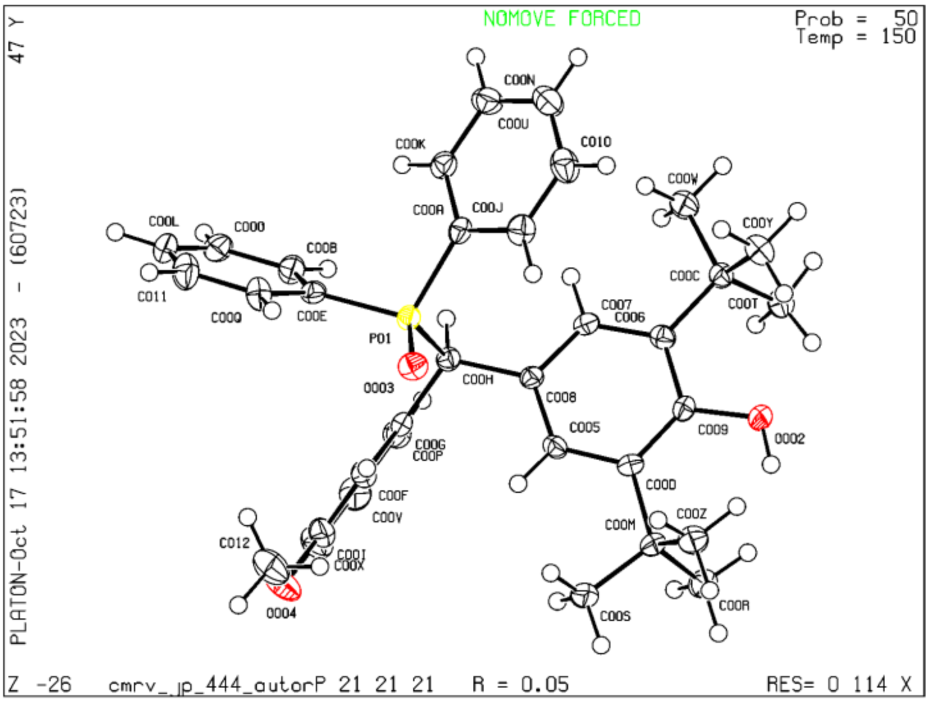

Figure S14. Molecular structure of **9b** with 50% ellipsoid probability.

**Table 3 Crystal data and structure refinement for CMRV_JP_444_autored.**	
Identification code	CMRV_JP_444_autored
Empirical formula	$C_{34}H_{39}O_3P$
Formula weight	526.62
Temperature/K	150.00(10)
Crystal system	orthorhombic
Space group	$P2_12_12_1$
a/Å	9.2907(3)
b/Å	14.7314(4)
c/Å	21.2629(6)
α/°	90
β/°	90
γ/°	90
Volume/Å^3	2910.15(15)
Z	4
ρ_{calc}g/cm^3	1.202

μ/mm^{-1}	0.127
F(000)	1128.0
Crystal size/mm^3	0.089 × 0.067 × 0.035
Radiation	Mo Kα (λ = 0.71073)
2Θ range for data collection/°	3.364 to 49.994
Index ranges	$-9 \leq h \leq 11$, $-17 \leq k \leq 17$, $-25 \leq l \leq 23$
Reflections collected	30959
Independent reflections	5130 [R_{int} = 0.1037, R_{sigma} = 0.0680]
Data/restraints/parameters	5130/0/354
Goodness-of-fit on F^2	1.044
Final R indexes [I>=2σ (I)]	R_1 = 0.0467, wR_2 = 0.1063
Final R indexes [all data]	R_1 = 0.0565, wR_2 = 0.1149
Largest diff. peak/hole / e Å^{-3}	0.20/-0.36
Flack parameter	-0.10(10)

12. ^{1}H and ^{13}C NMR spectra of the compounds

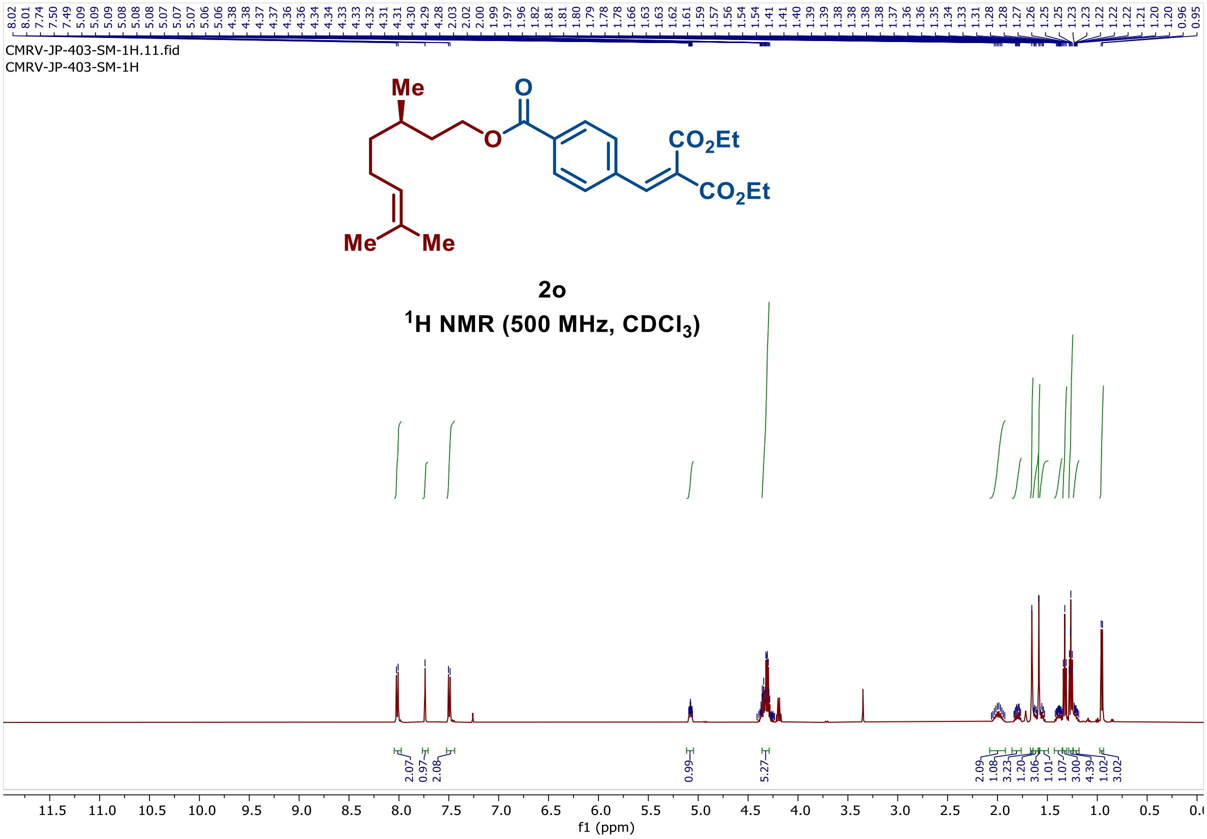

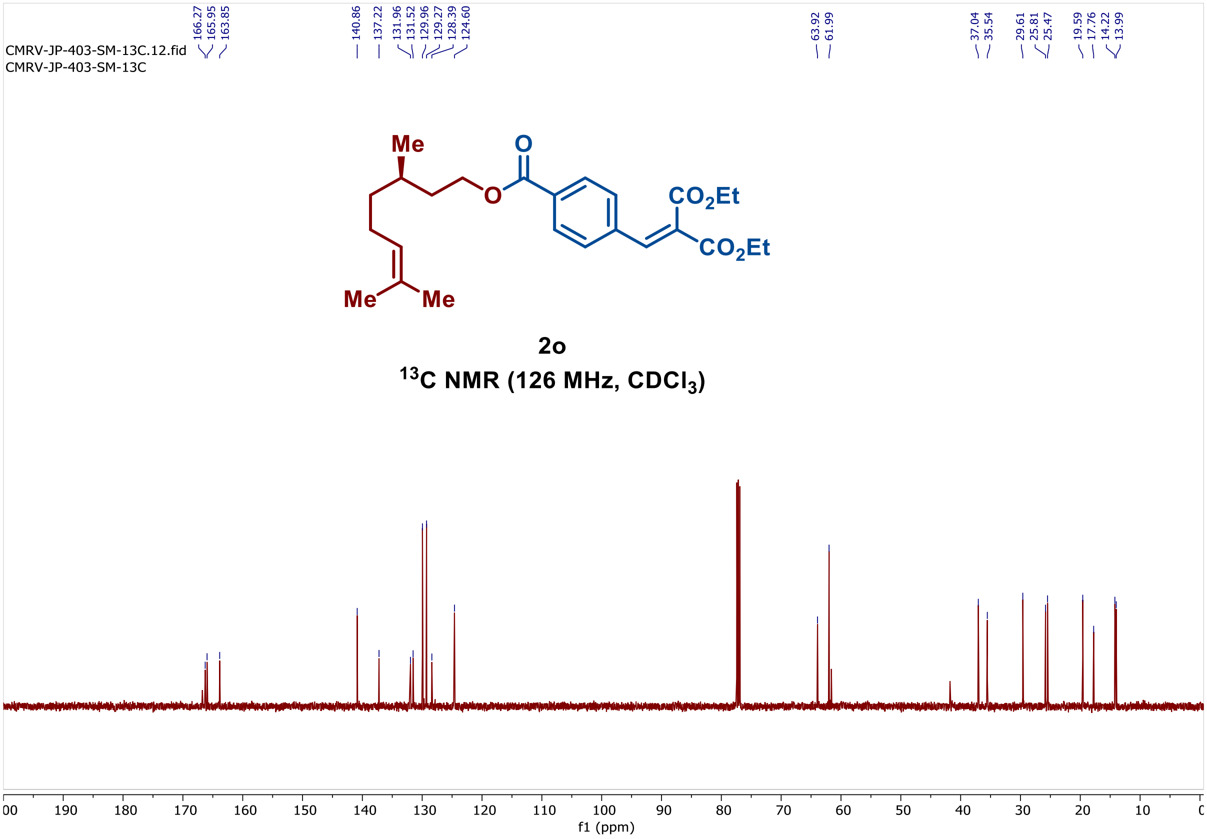

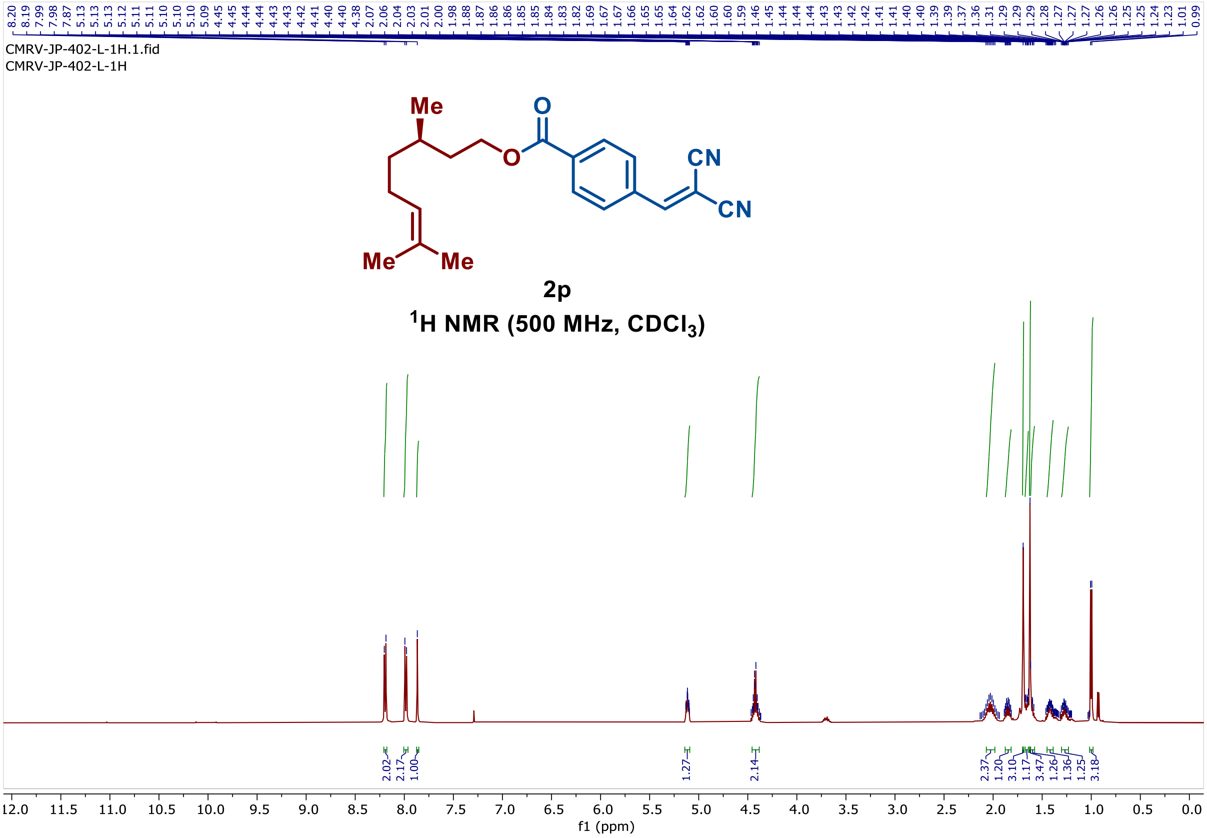

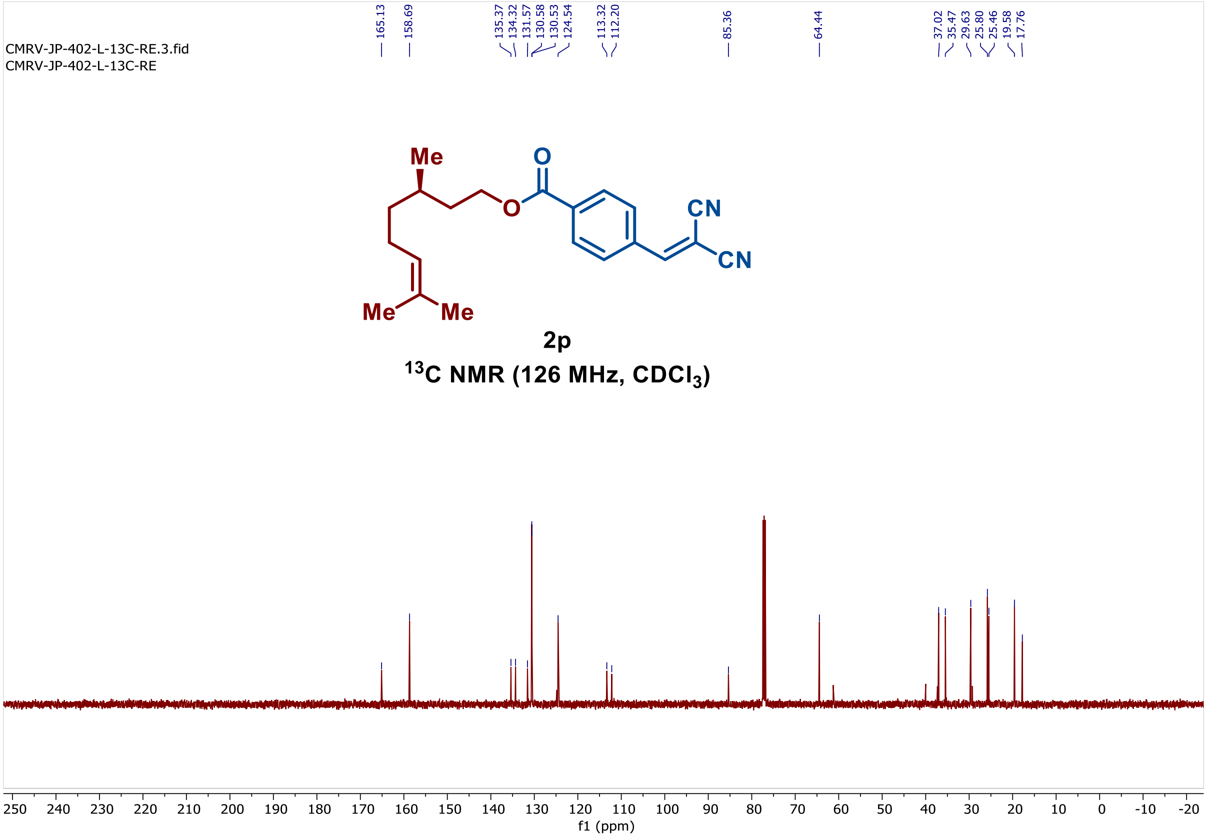

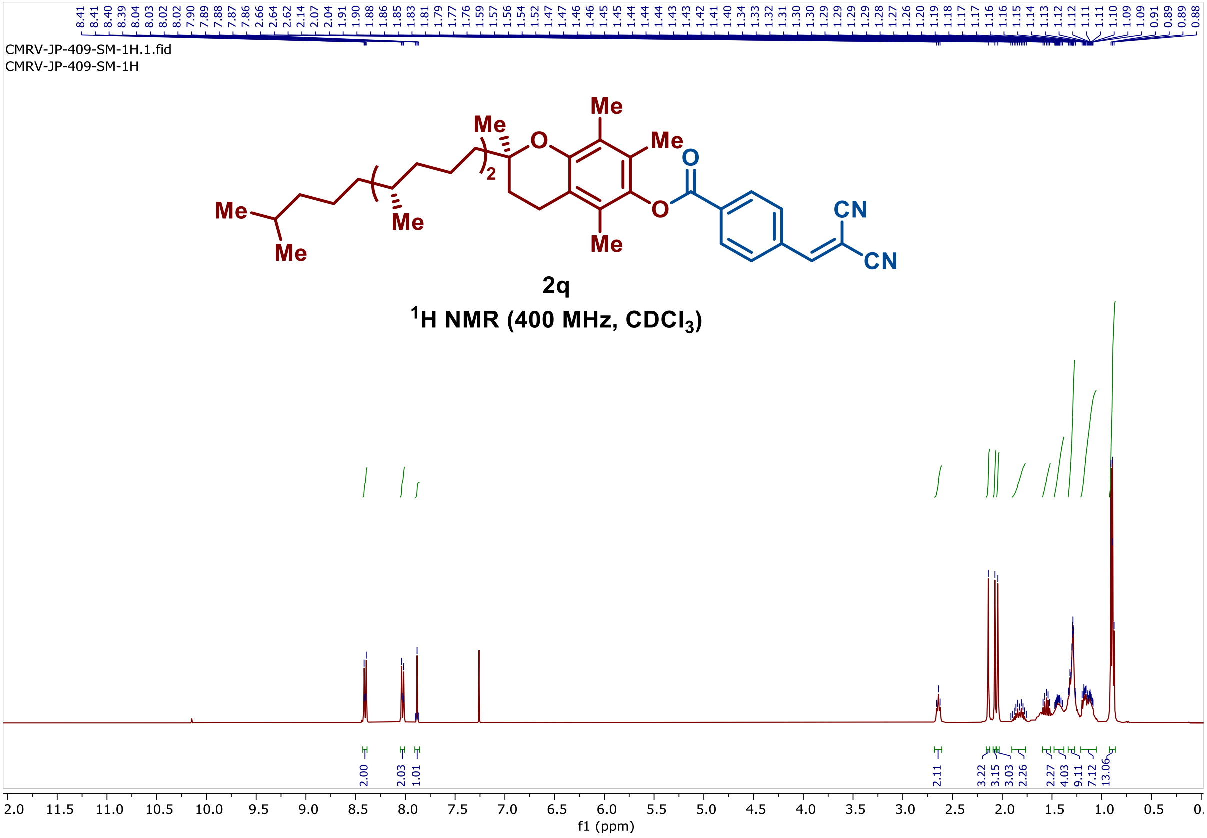

8.41 8.41 8.40 8.39 8.04 8.03 8.02 8.02 7.90 7.89 7.88 7.87 7.86 2.66 2.64 2.62 2.14 2.07 2.04 1.91 1.90 1.88 1.86 1.85 1.83 1.81 1.79 1.77 1.76 1.59 1.57 1.56 1.54 1.52 1.47 1.47 1.46 1.46 1.45 1.45 1.44 1.44 1.43 1.43 1.42 1.41 1.40 1.34 1.33 1.32 1.31 1.30 1.30 1.29 1.29 1.29 1.29 1.28 1.27 1.26 1.20 1.19 1.18 1.17 1.17 1.16 1.16 1.15 1.14 1.13 1.12 1.12 1.11 1.11 1.11 1.10 1.09 1.09 0.91 0.89 0.89 0.88
CMRV-JP-409-SM-1H.1.fid
CMRV-JP-409-SM-1H
Me
Me
O
Me
2
O
O
CN
CN
Me
Me
Me
Me
2q
1H NMR (400 MHz, CDCl3)
2.00 2.03 1.01 2.11 3.22 3.15 3.03 2.26 2.27 4.03 9.11 7.12 13.06
2.0 11.5 11.0 10.5 10.0 9.5 9.0 8.5 8.0 7.5 7.0 6.5 6.0 5.5 5.0 4.5 4.0 3.5 3.0 2.5 2.0 1.5 1.0 0.5 0.
f1 (ppm)

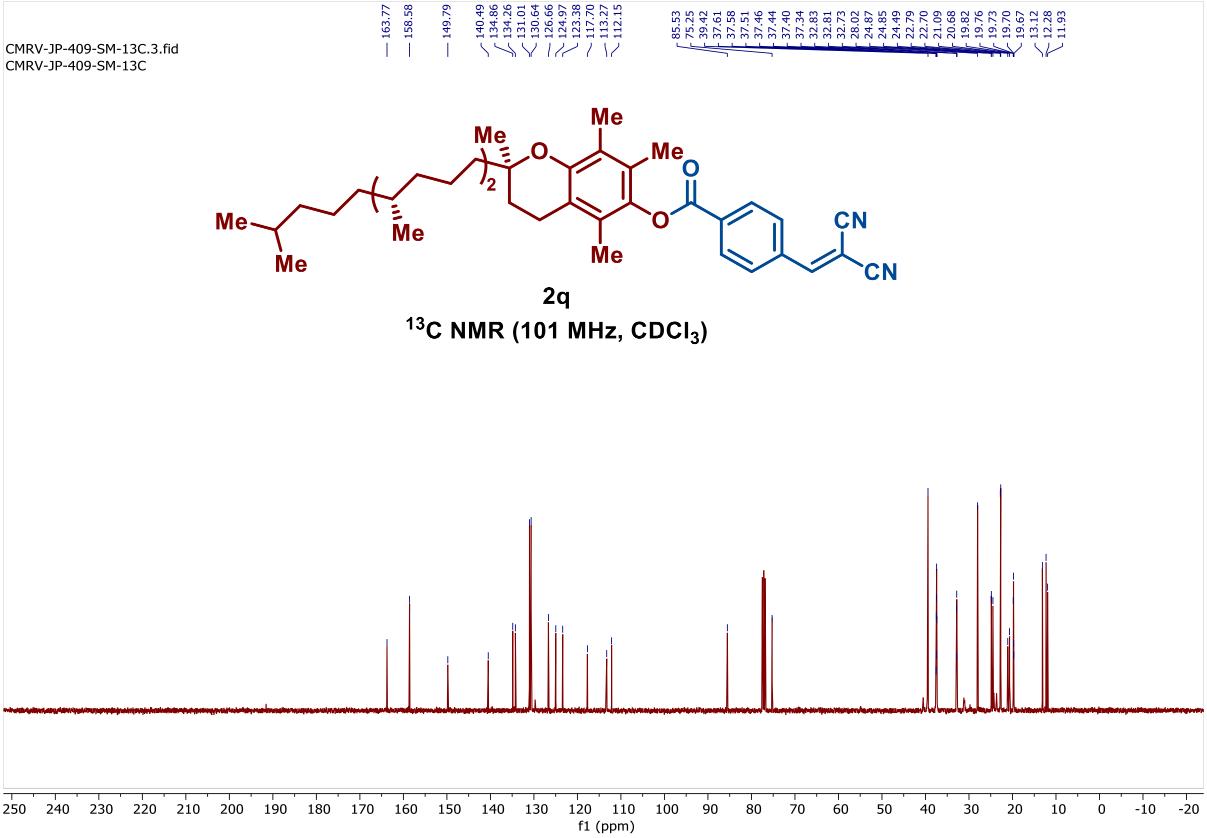

163.77 158.58 149.79 140.49 134.86 134.26 131.01 130.64 126.66 124.97 123.38 117.70 113.27 112.15 85.53 75.25 39.42 37.61 37.58 37.51 37.46 37.44 37.40 37.34 32.83 32.81 32.73 28.02 24.87 24.85 24.49 22.79 22.70 21.09 20.68 19.82 19.76 19.73 19.70 19.67 13.12 12.28 11.93
CMRV-JP-409-SM-13C.3.fid
CMRV-JP-409-SM-13C
2q
13C NMR (101 MHz, CDCl3)
250 240 230 220 210 200 190 180 170 160 150 140 130 120 110 100 90 80 70 60 50 40 30 20 10 0 -10 -20
f1 (ppm)

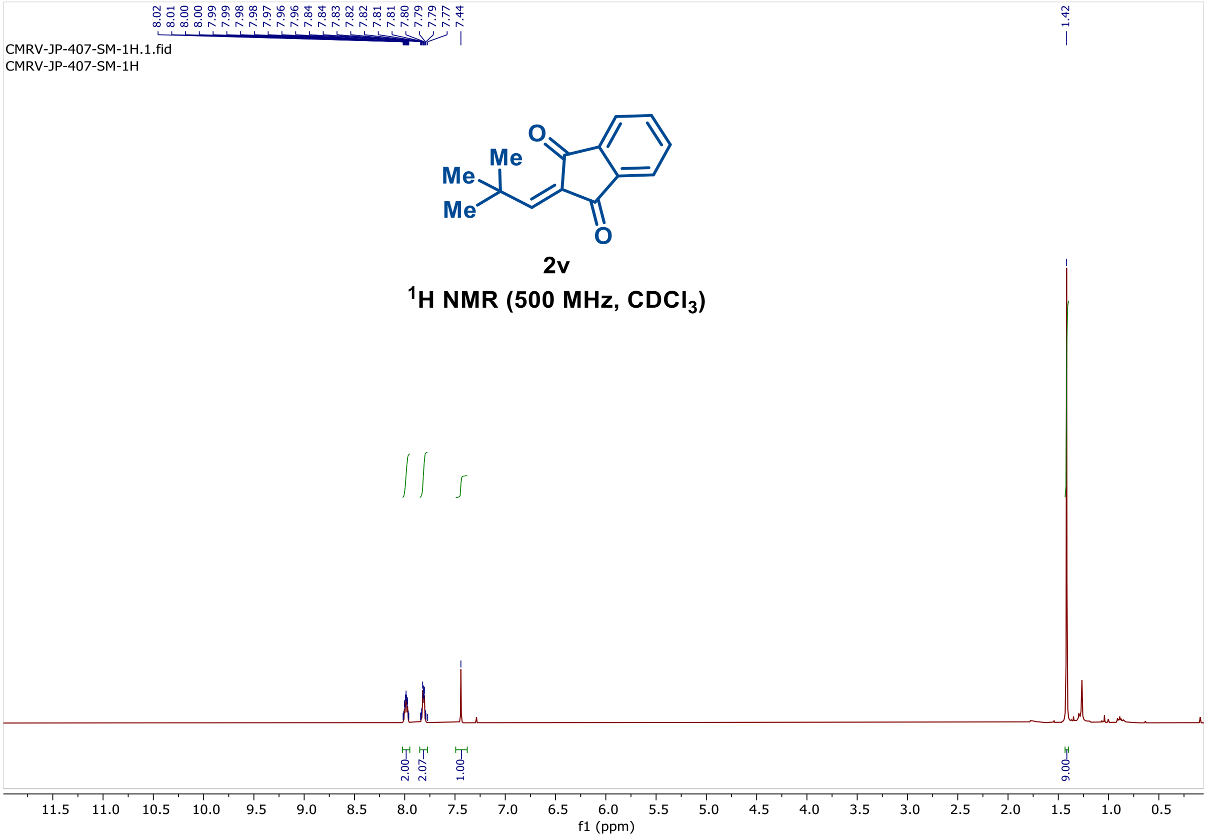
8.02
8.01
8.00
7.99
7.99
7.98
7.98
7.97
7.96
7.96
7.84
7.84
7.83
7.82
7.82
7.81
7.81
7.80
7.79
7.79
7.77
7.44
1.42
CMRV-JP-407-SM-1H.1.fid
CMRV-JP-407-SM-1H
Me
Me
Me
O
O
2v
^{1}H NMR (500 MHz, CDCl$_3$)
2.00
2.07
1.00
9.00
11.5
11.0
10.5
10.0
9.5
9.0
8.5
8.0
7.5
7.0
6.5
6.0
5.5
5.0
4.5
4.0
3.5
3.0
2.5
2.0
1.5
1.0
0.5
f1 (ppm)

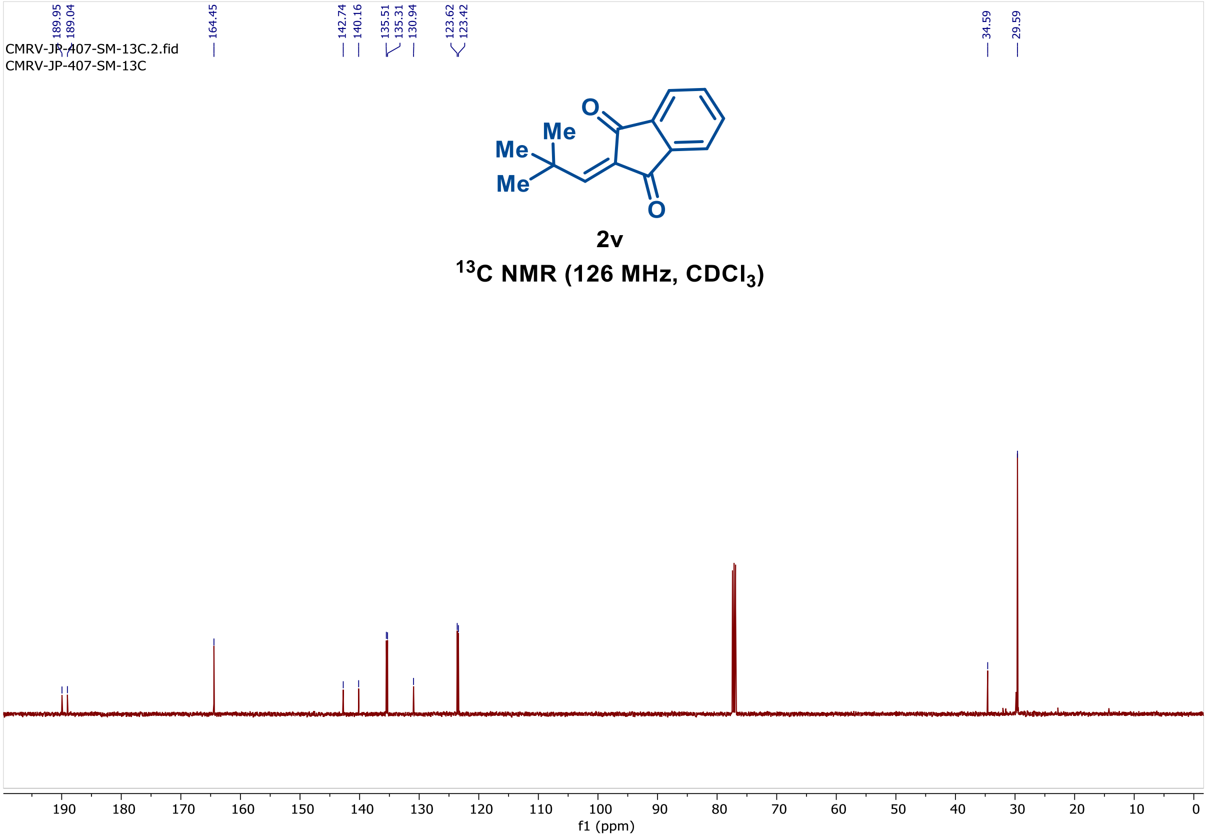
189.95
189.04
164.45
142.74
140.16
135.51
135.31
130.94
123.62
123.42
34.59
29.59
CMRV-JP-407-SM-13C.2.fid
CMRV-JP-407-SM-13C
Me
Me
Me
O
O
2v
^{13}C NMR (126 MHz, CDCl$_3$)
190
180
170
160
150
140
130
120
110
100
90
80
70
60
50
40
30
20
10
0
f1 (ppm)

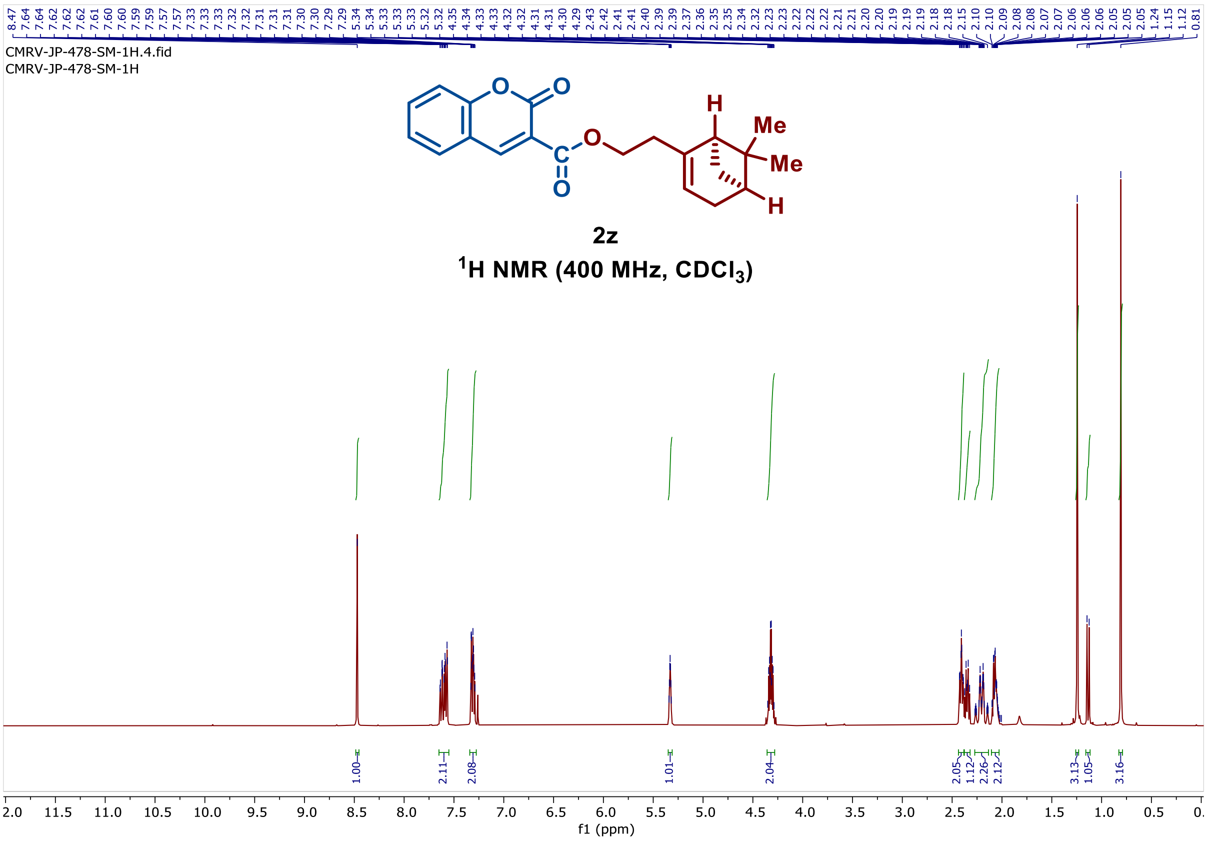
CMRV-JP-478-SM-1H.4.fid
CMRV-JP-478-SM-1H
2z
1H NMR (400 MHz, CDCl3)
f1 (ppm)

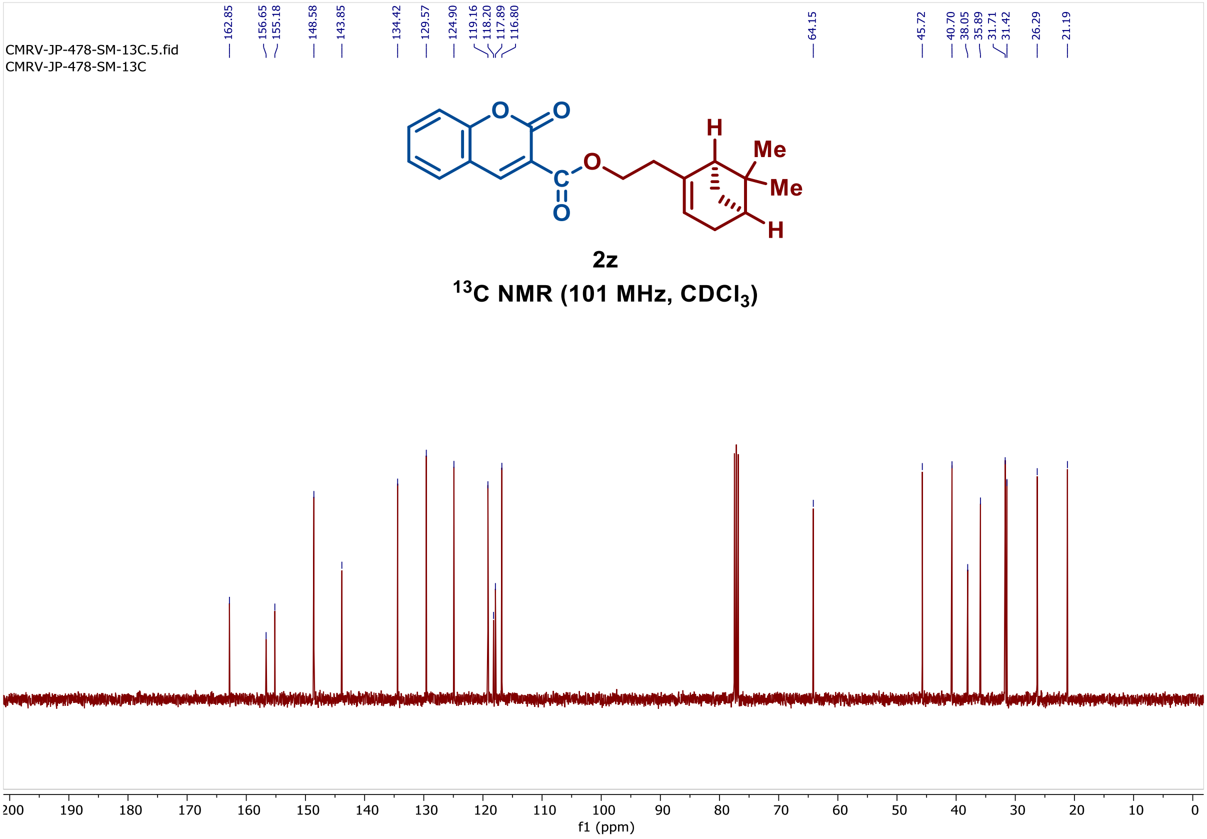
162.85
156.65
155.18
148.58
143.85
134.42
129.57
124.90
119.16
118.20
117.89
116.80
64.15
45.72
40.70
38.05
35.89
31.71
31.42
26.29
21.19
CMRV-JP-478-SM-13C.5.fid
CMRV-JP-478-SM-13C
2z
13C NMR (101 MHz, CDCl3)
f1 (ppm)

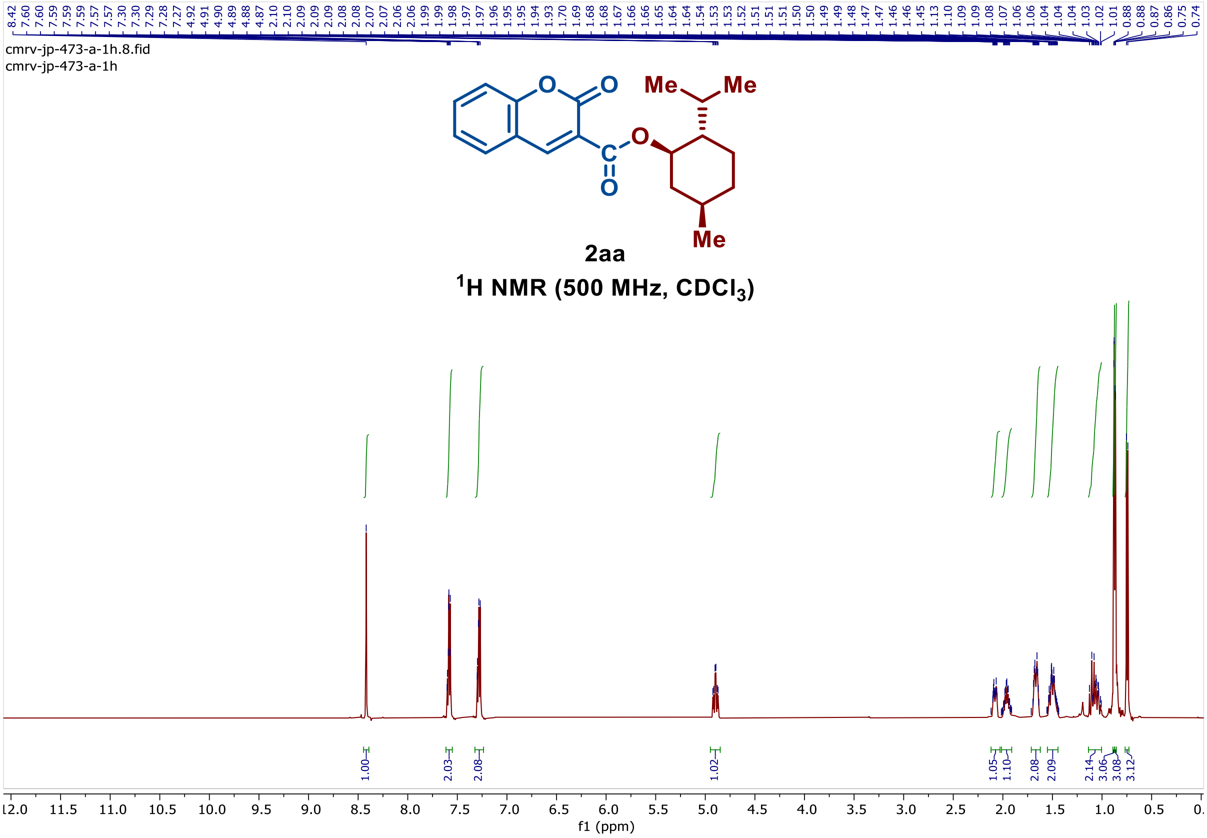
cmrv-jp-473-a-1h.8.fid
cmrv-jp-473-a-1h
2aa
^{1}H NMR (500 MHz, $CDCl_3$)
f1 (ppm)

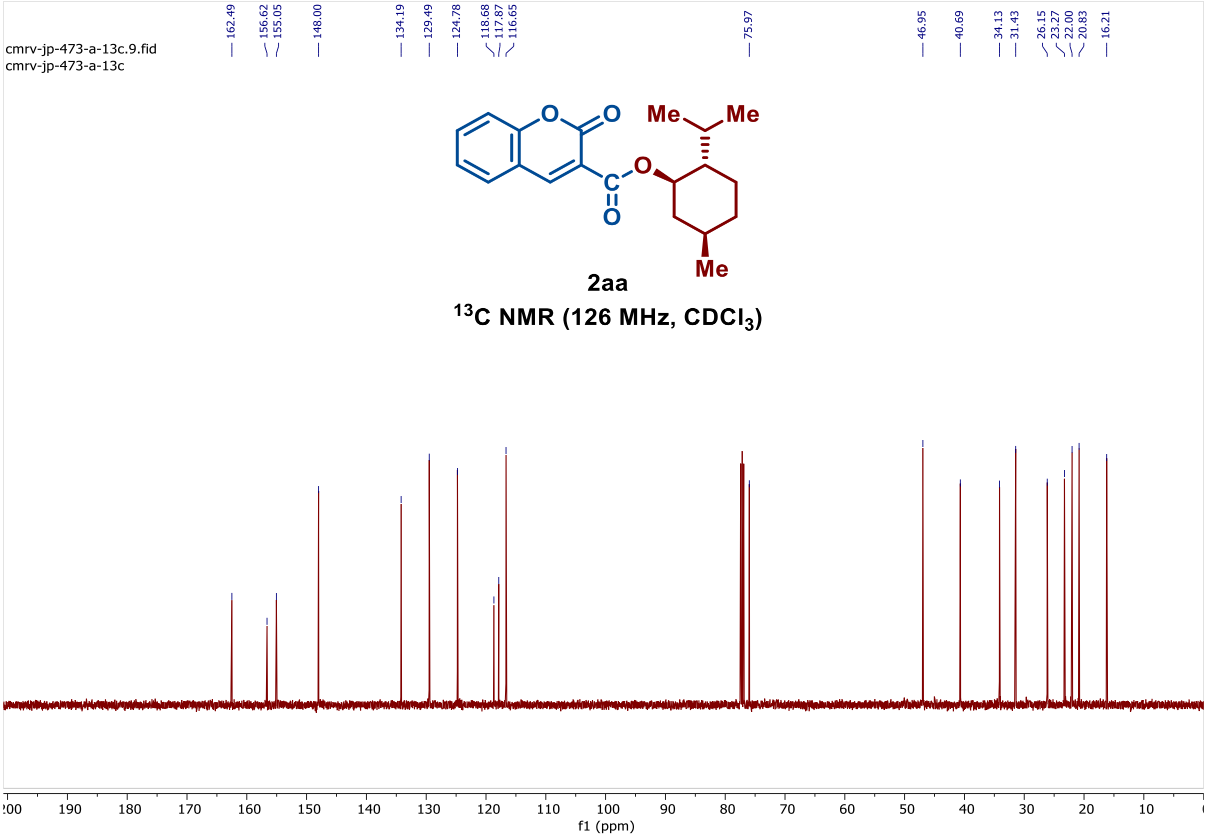
162.49
156.62
155.05
148.00
134.19
129.49
124.78
118.68
117.87
116.65
75.97
46.95
40.69
34.13
31.43
26.15
23.27
22.00
20.83
16.21
cmrv-jp-473-a-13c.9.fid
cmrv-jp-473-a-13c
2aa
^{13}C NMR (126 MHz, $CDCl_3$)
f1 (ppm)

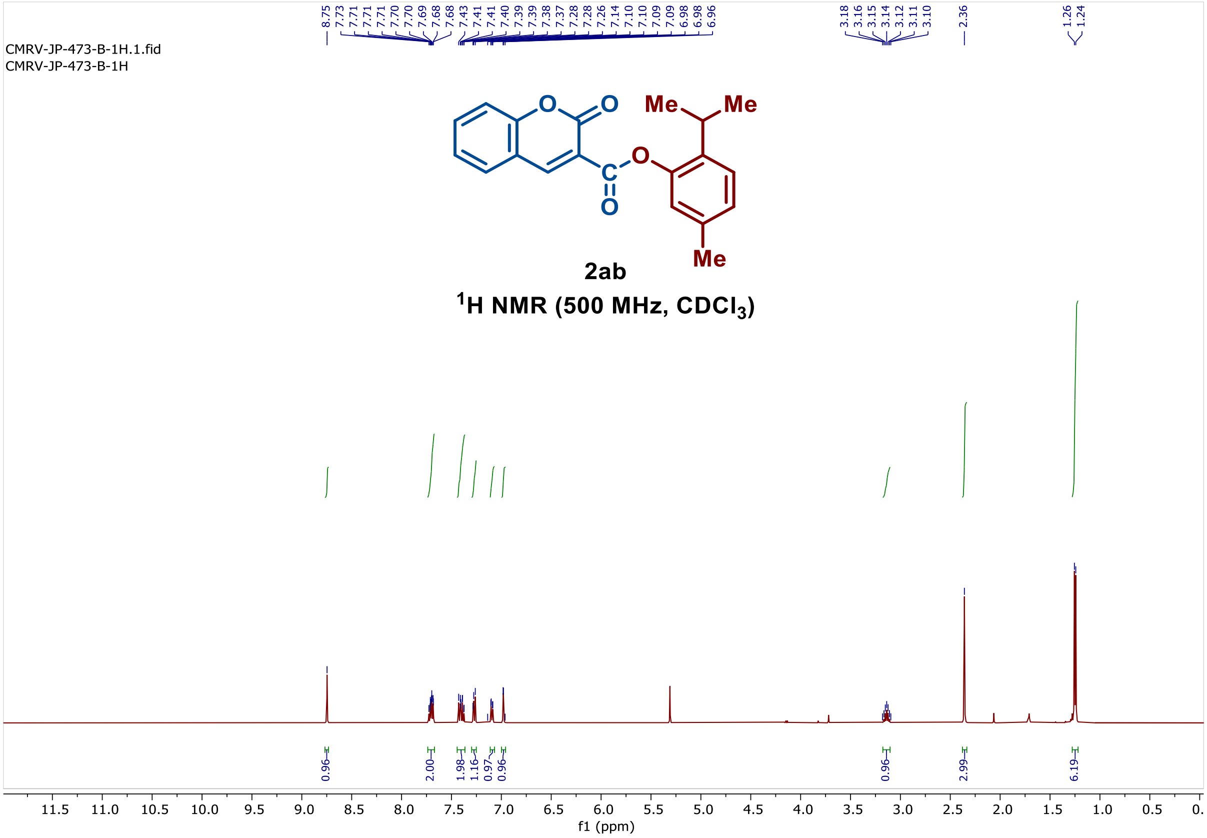
CMRV-JP-473-B-1H.1.fid
CMRV-JP-473-B-1H
2ab
1H NMR (500 MHz, CDCl3)
f1 (ppm)

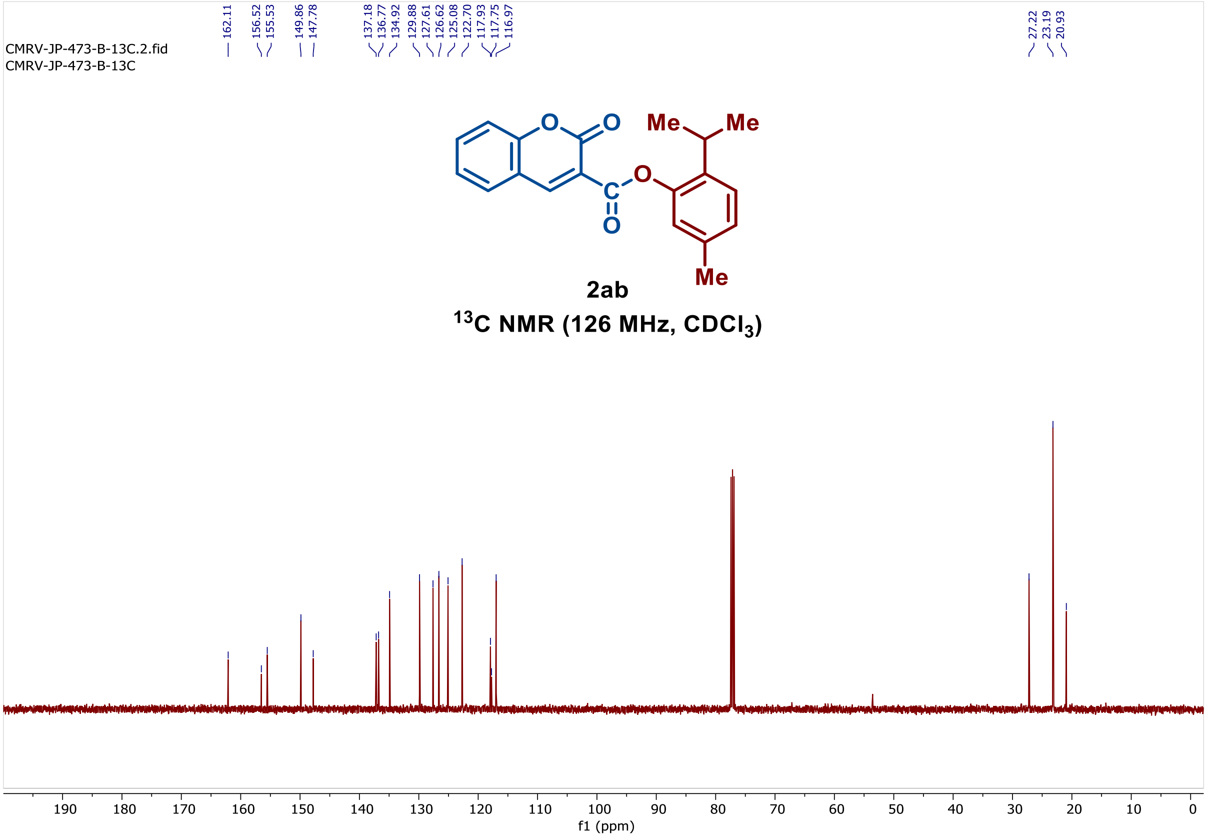
CMRV-JP-473-B-13C.2.fid
CMRV-JP-473-B-13C
162.11
156.52
155.53
149.86
147.78
137.18
136.77
134.92
129.88
127.61
126.62
125.08
122.70
117.93
117.75
116.97
27.22
23.19
20.93
2ab
13C NMR (126 MHz, CDCl3)
f1 (ppm)

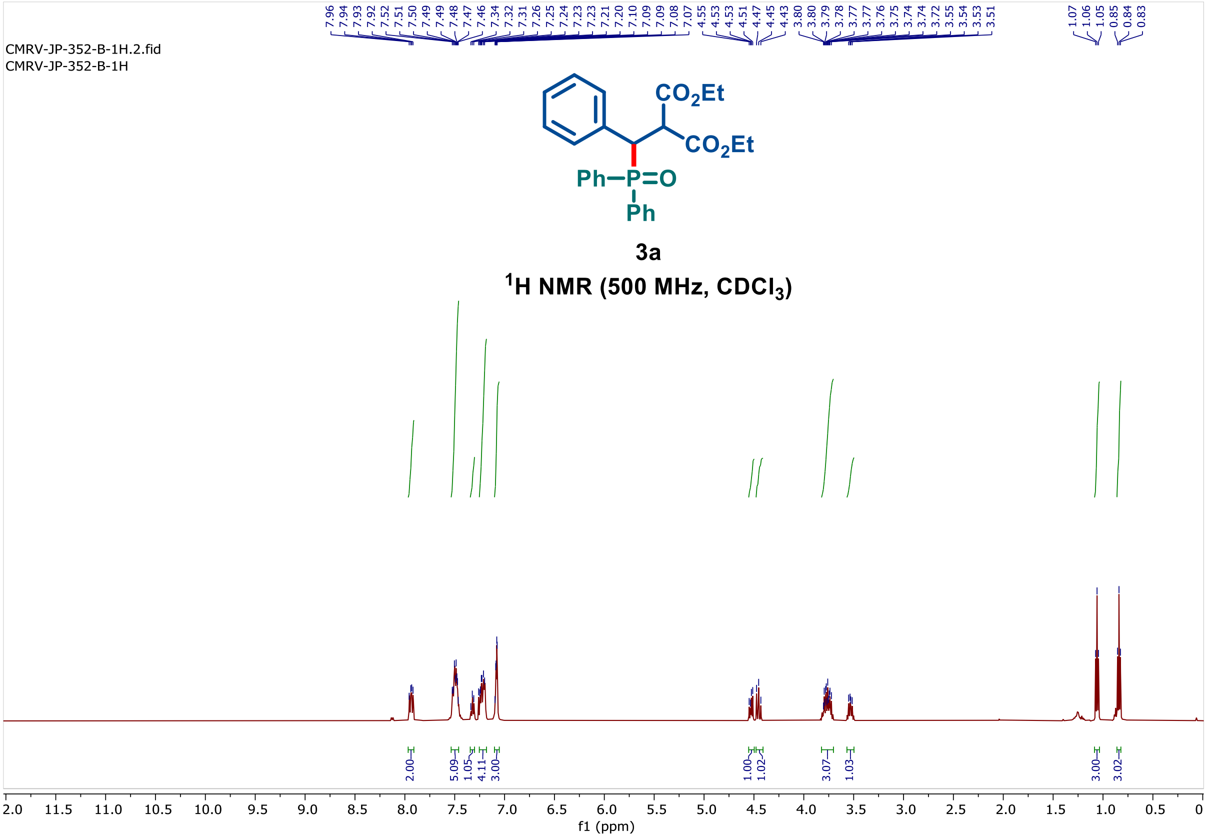
CMRV-JP-352-B-1H.2.fid
CMRV-JP-352-B-1H
CO2Et
CO2Et
Ph–P=O
Ph
3a
1H NMR (500 MHz, CDCl3)
f1 (ppm)

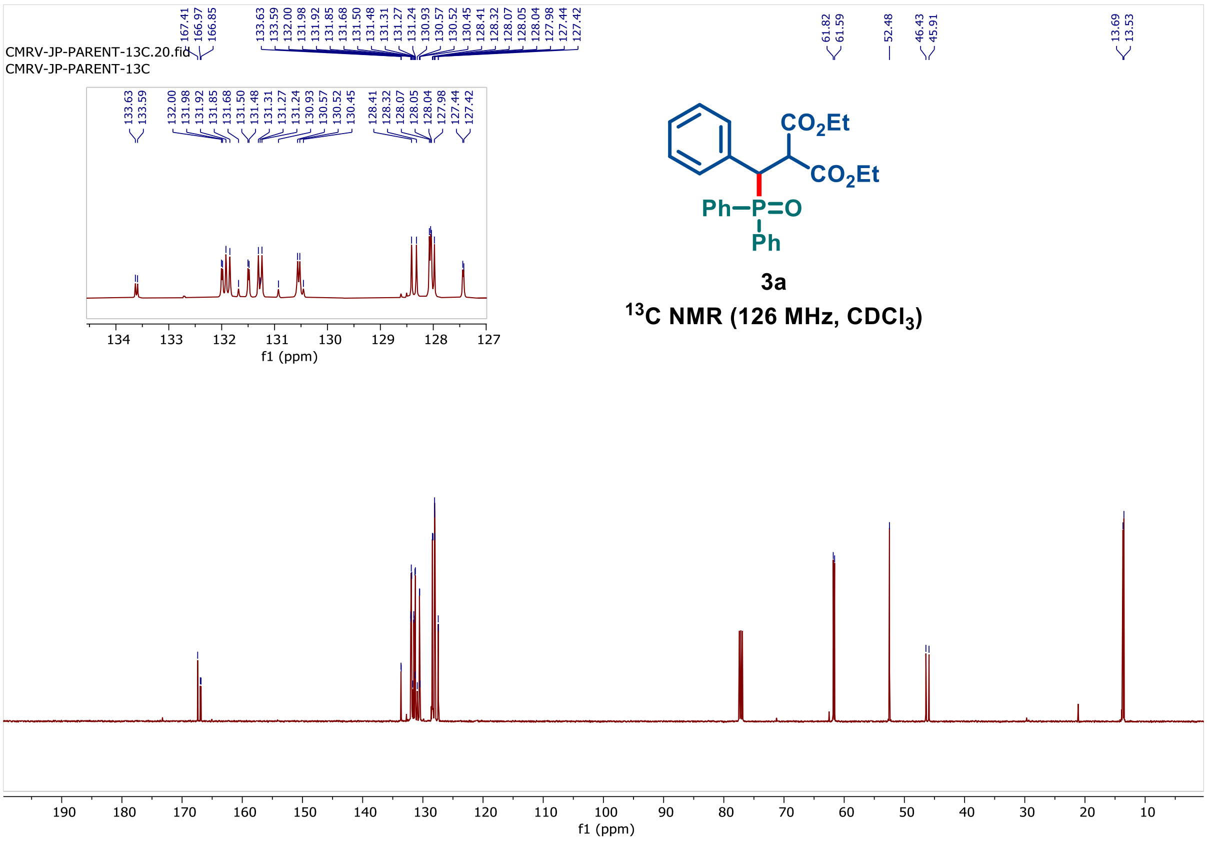
CMRV-JP-PARENT-13C.20.fid
CMRV-JP-PARENT-13C
CO2Et
CO2Et
Ph–P=O
Ph
3a
13C NMR (126 MHz, CDCl3)
f1 (ppm)

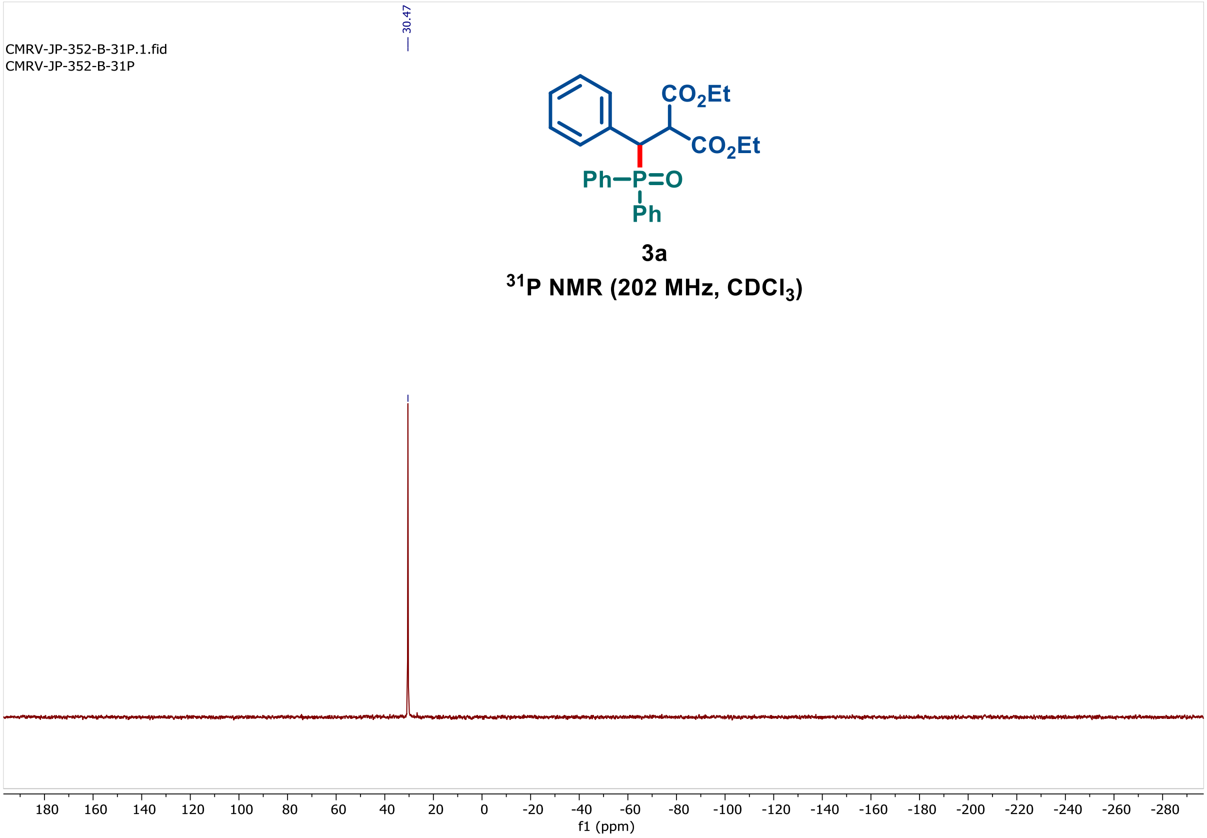
30.47
CMRV-JP-352-B-31P.1.fid
CMRV-JP-352-B-31P
CO2Et
CO2Et
Ph–P=O
Ph
3a
31P NMR (202 MHz, CDCl3)
f1 (ppm)

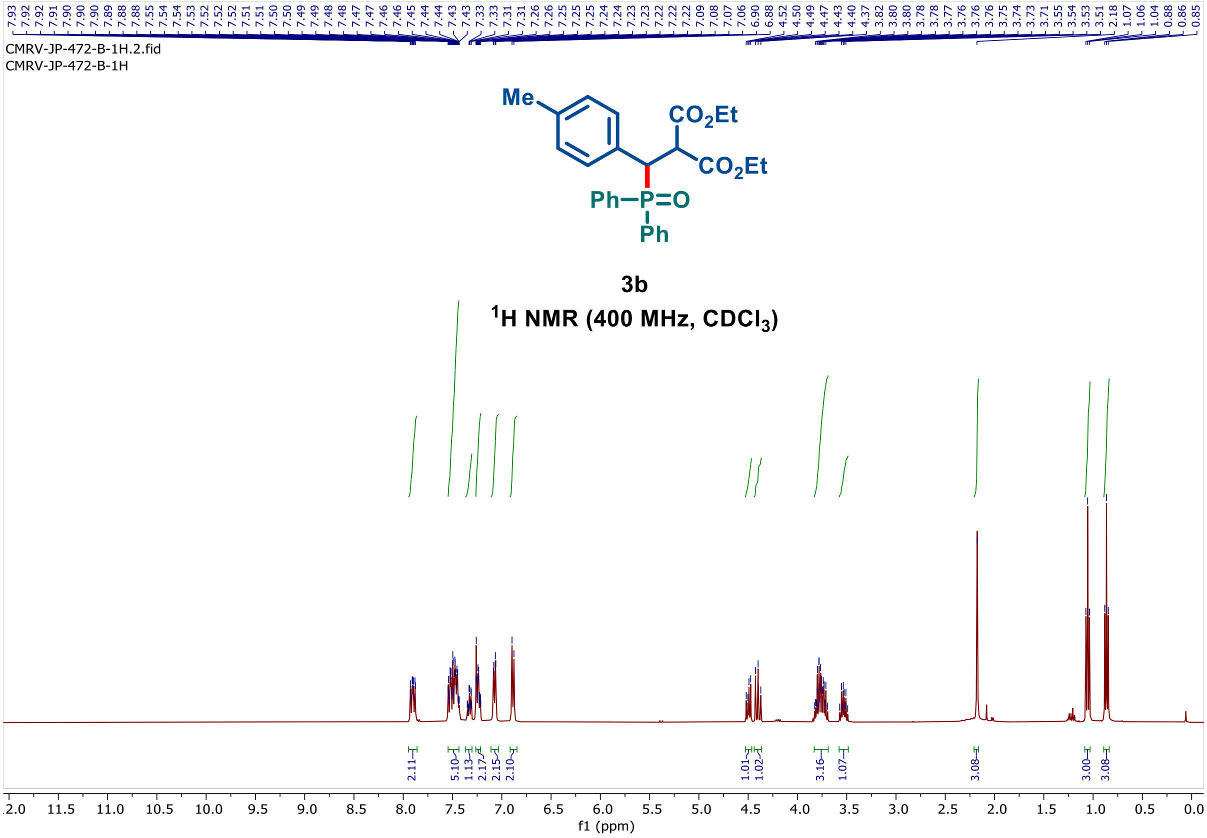
CMRV-JP-472-B-1H.2.fid
CMRV-JP-472-B-1H
Me
CO2Et
CO2Et
Ph–P=O
Ph
3b
1H NMR (400 MHz, CDCl3)
f1 (ppm)

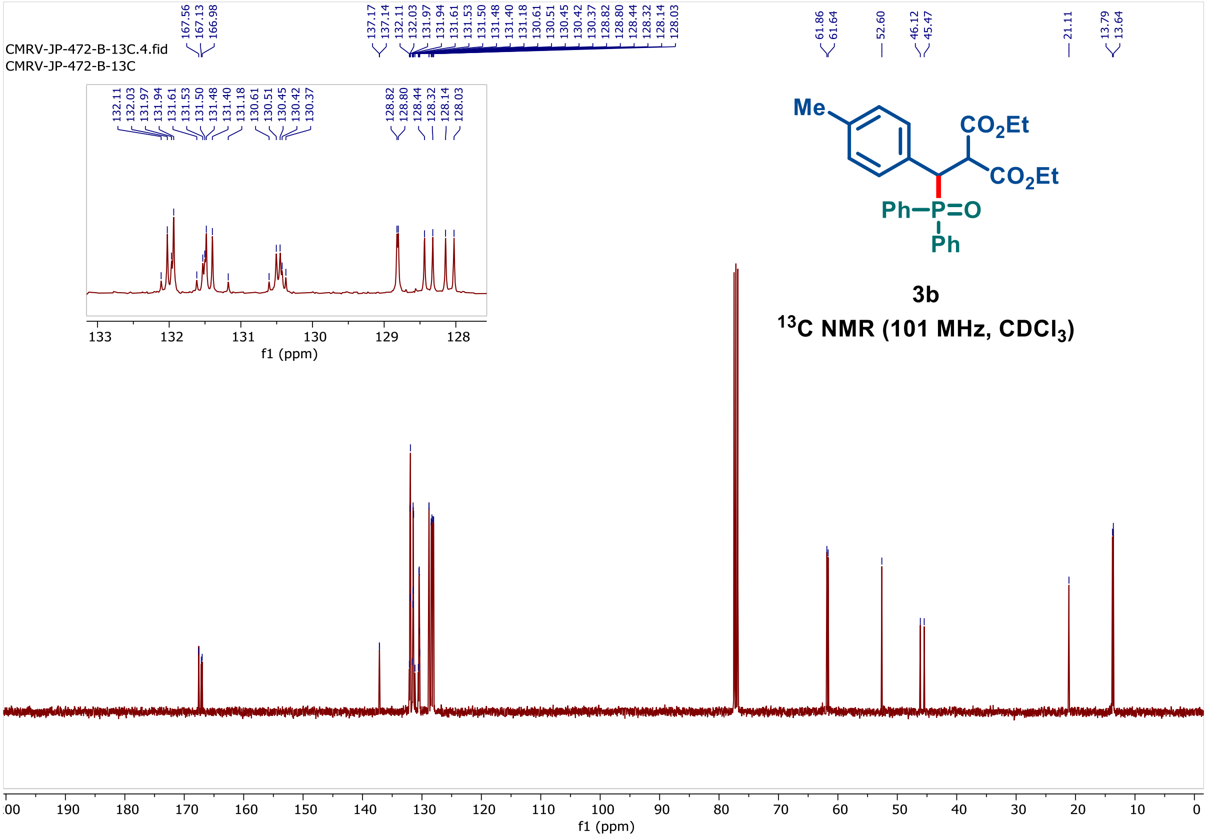
CMRV-JP-472-B-13C.4.fid
CMRV-JP-472-B-13C
167.56
167.13
166.98
137.17
137.14
132.11
132.03
131.97
131.94
131.61
131.53
131.50
131.48
131.40
131.18
130.61
130.51
130.45
130.42
130.37
128.82
128.80
128.44
128.32
128.14
128.03
61.86
61.64
52.60
46.12
45.47
21.11
13.79
13.64
Me
CO2Et
CO2Et
Ph—P=O
Ph
3b
13C NMR (101 MHz, CDCl3)
f1 (ppm)

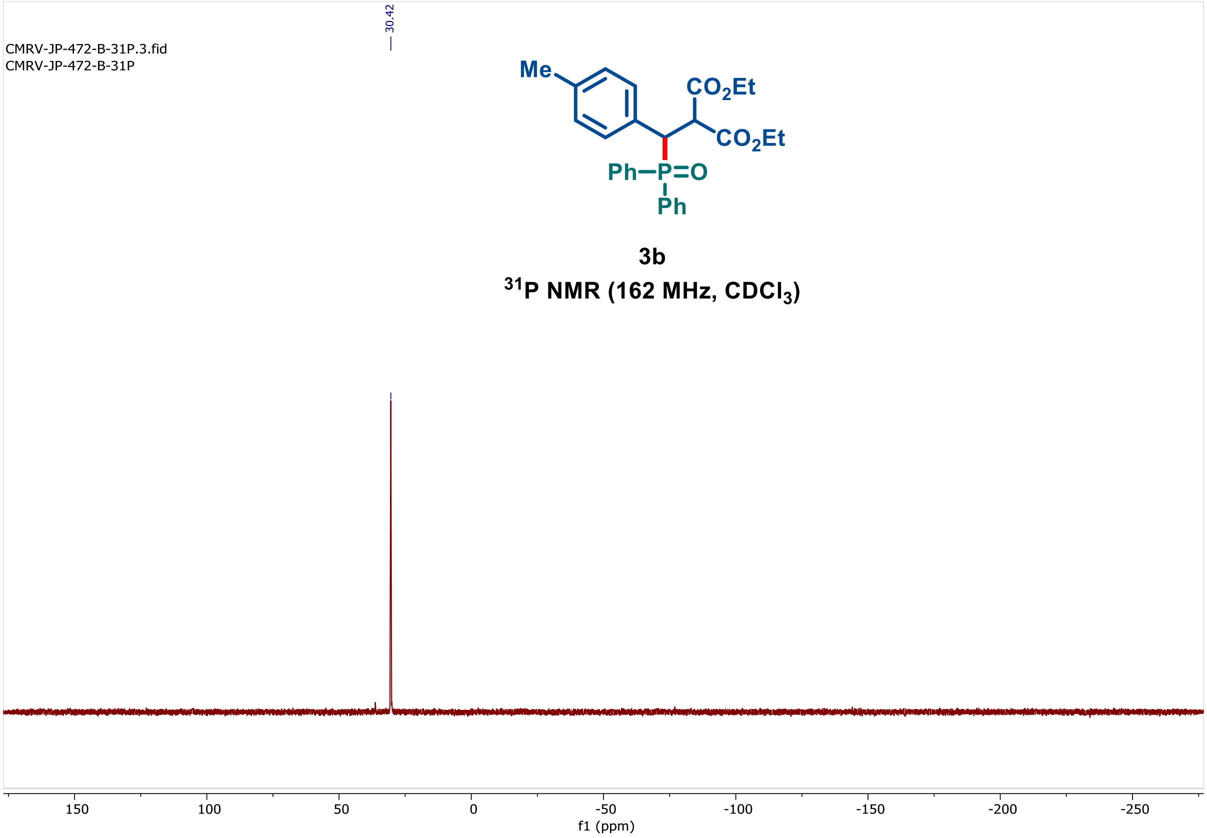
CMRV-JP-472-B-31P.3.fid
CMRV-JP-472-B-31P
30.42
Me
CO2Et
CO2Et
Ph—P=O
Ph
3b
31P NMR (162 MHz, CDCl3)
f1 (ppm)

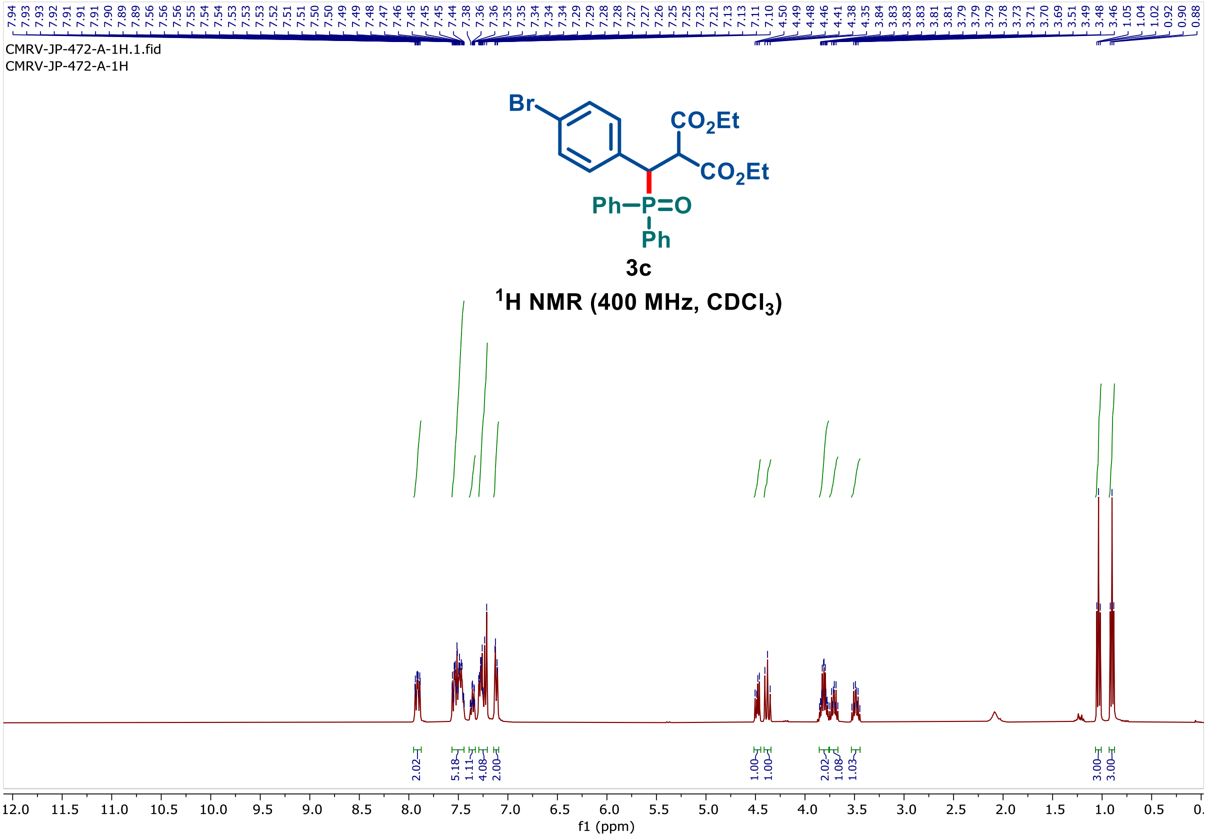
CMRV-JP-472-A-1H.1.fid
CMRV-JP-472-A-1H
3c
1H NMR (400 MHz, CDCl3)
f1 (ppm)

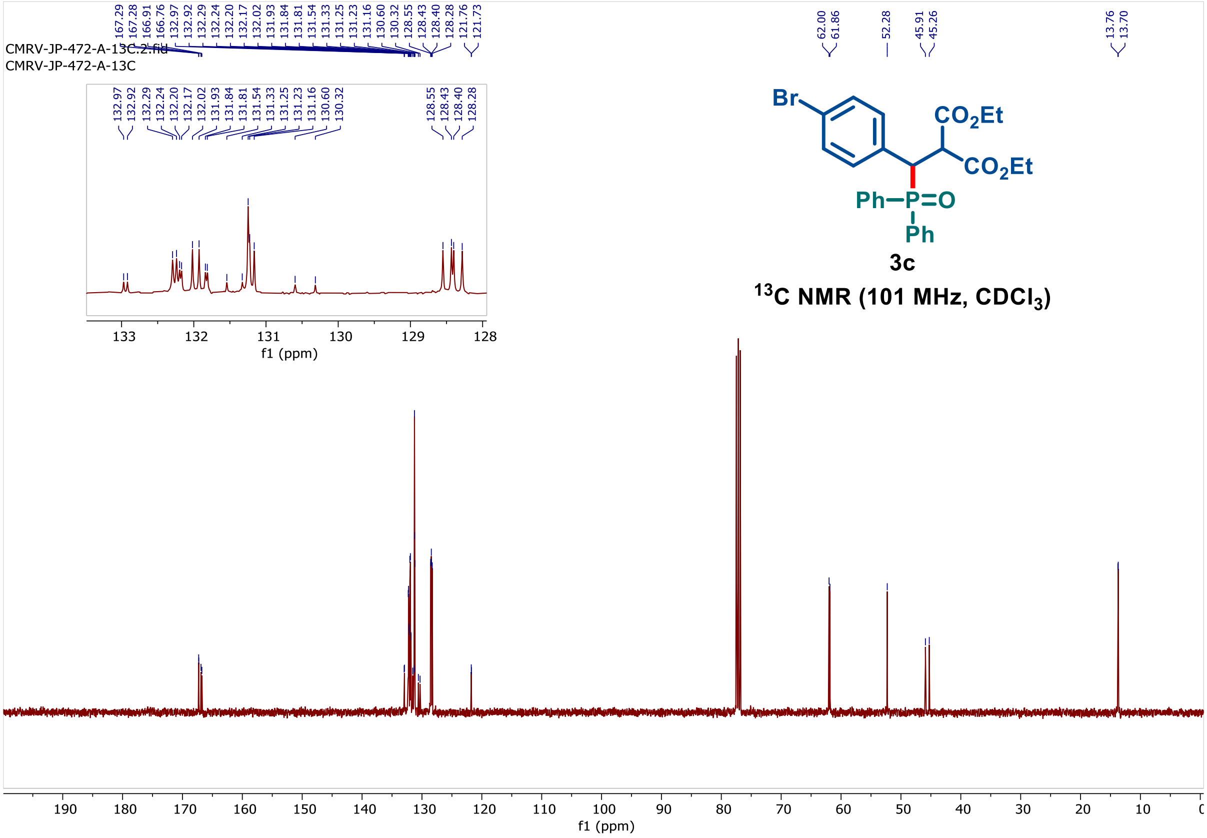
CMRV-JP-472-A-13C.2.fid
CMRV-JP-472-A-13C
3c
13C NMR (101 MHz, CDCl3)
f1 (ppm)

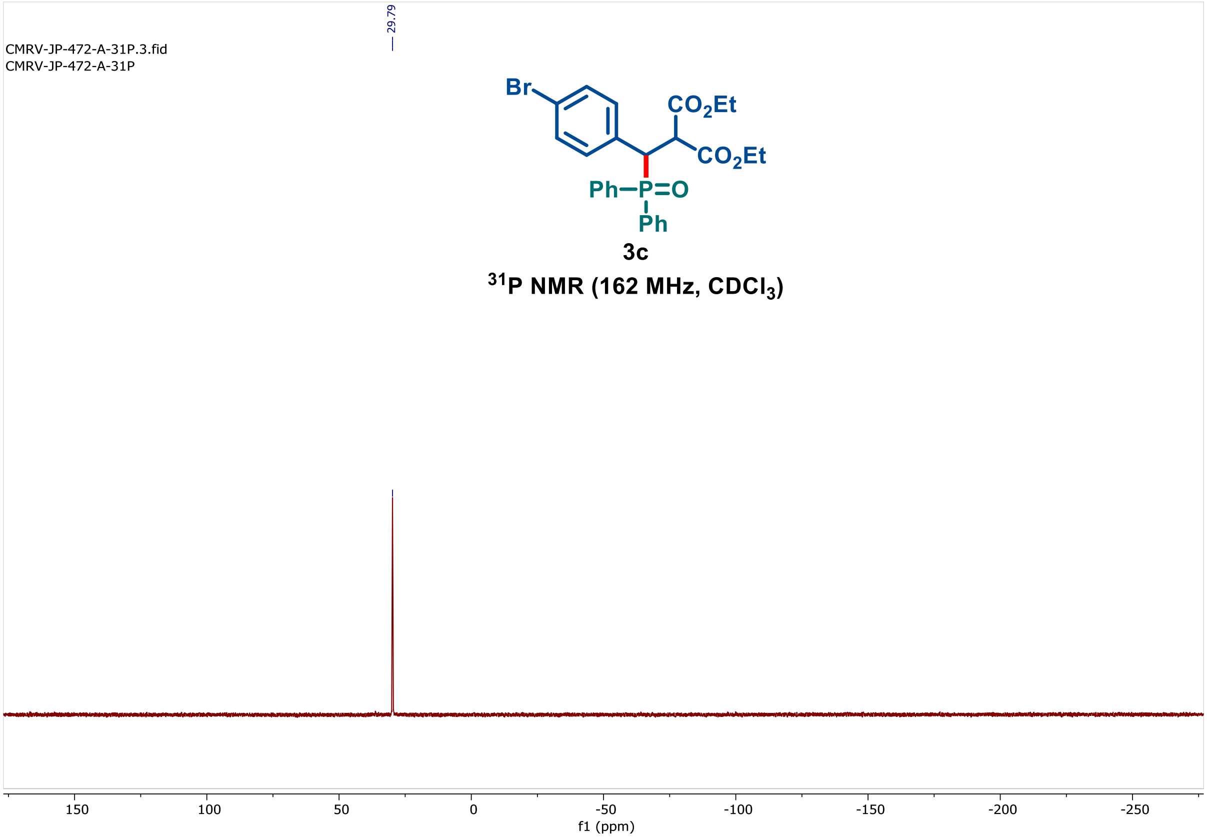
CMRV-JP-472-A-31P.3.fid
CMRV-JP-472-A-31P
29.79
Br
CO2Et
CO2Et
Ph–P=O
Ph
3c
31P NMR (162 MHz, CDCl3)
f1 (ppm)

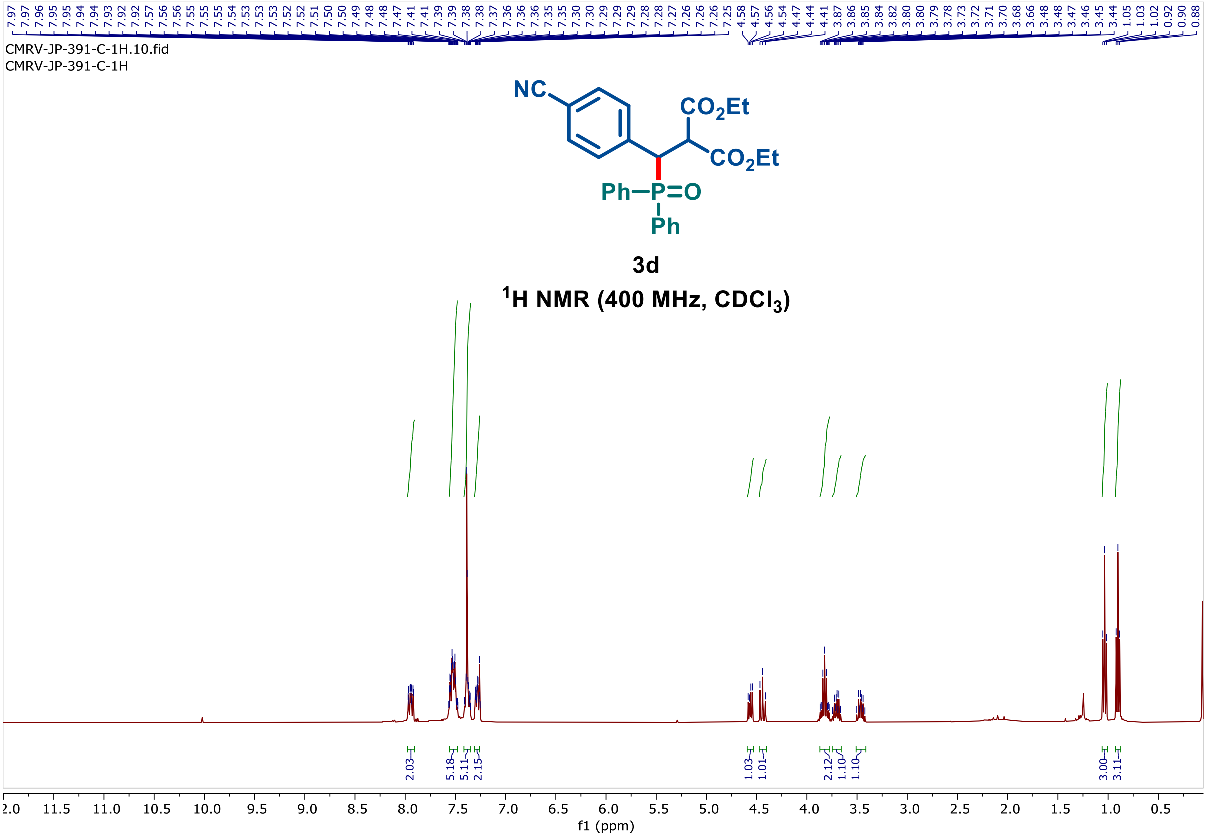
CMRV-JP-391-C-1H.10.fid
CMRV-JP-391-C-1H
NC
CO2Et
CO2Et
Ph–P=O
Ph
3d
1H NMR (400 MHz, CDCl3)
2.03
5.18
5.11
2.15
1.03
1.01
2.12
1.10
1.10
3.00
3.11
f1 (ppm)

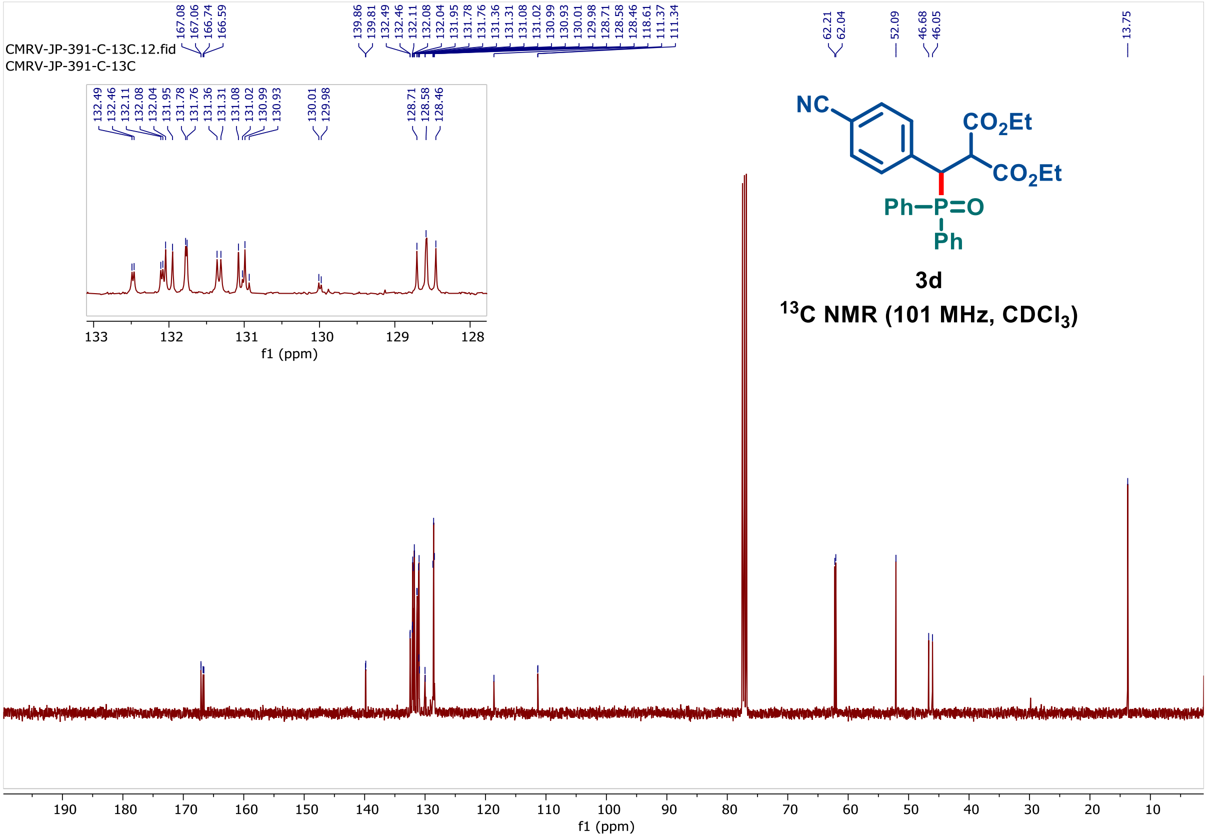
CMRV-JP-391-C-13C.12.fid
CMRV-JP-391-C-13C
167.08
167.06
166.74
166.59
139.86
139.81
132.49
132.46
132.11
132.08
132.04
131.95
131.78
131.76
131.36
131.31
131.08
131.02
130.99
130.93
130.01
129.98
128.71
128.58
128.46
118.61
111.37
111.34
62.21
62.04
52.09
46.68
46.05
13.75
NC
CO2Et
CO2Et
Ph–P=O
Ph
3d
13C NMR (101 MHz, CDCl3)
f1 (ppm)

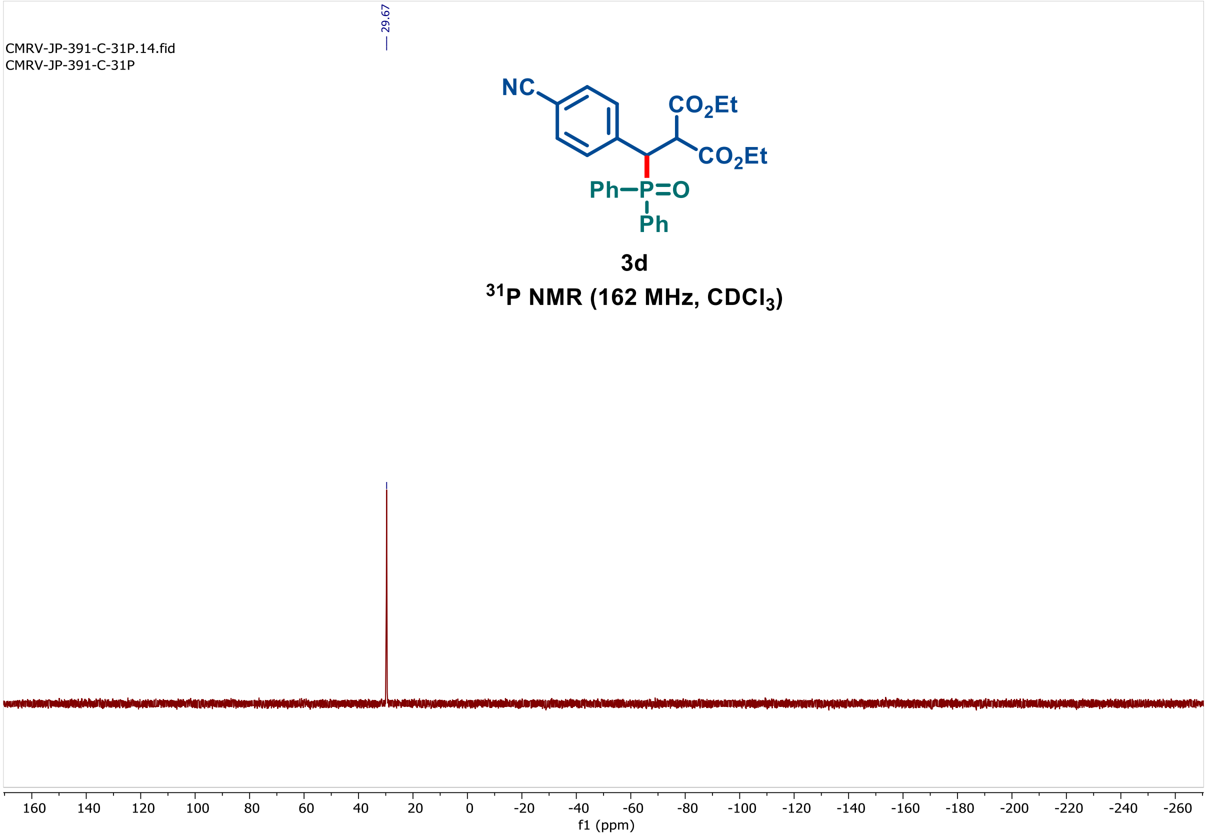
CMRV-JP-391-C-31P.14.fid
CMRV-JP-391-C-31P
29.67
NC
CO2Et
CO2Et
Ph–P=O
Ph
3d
31P NMR (162 MHz, CDCl3)
f1 (ppm)

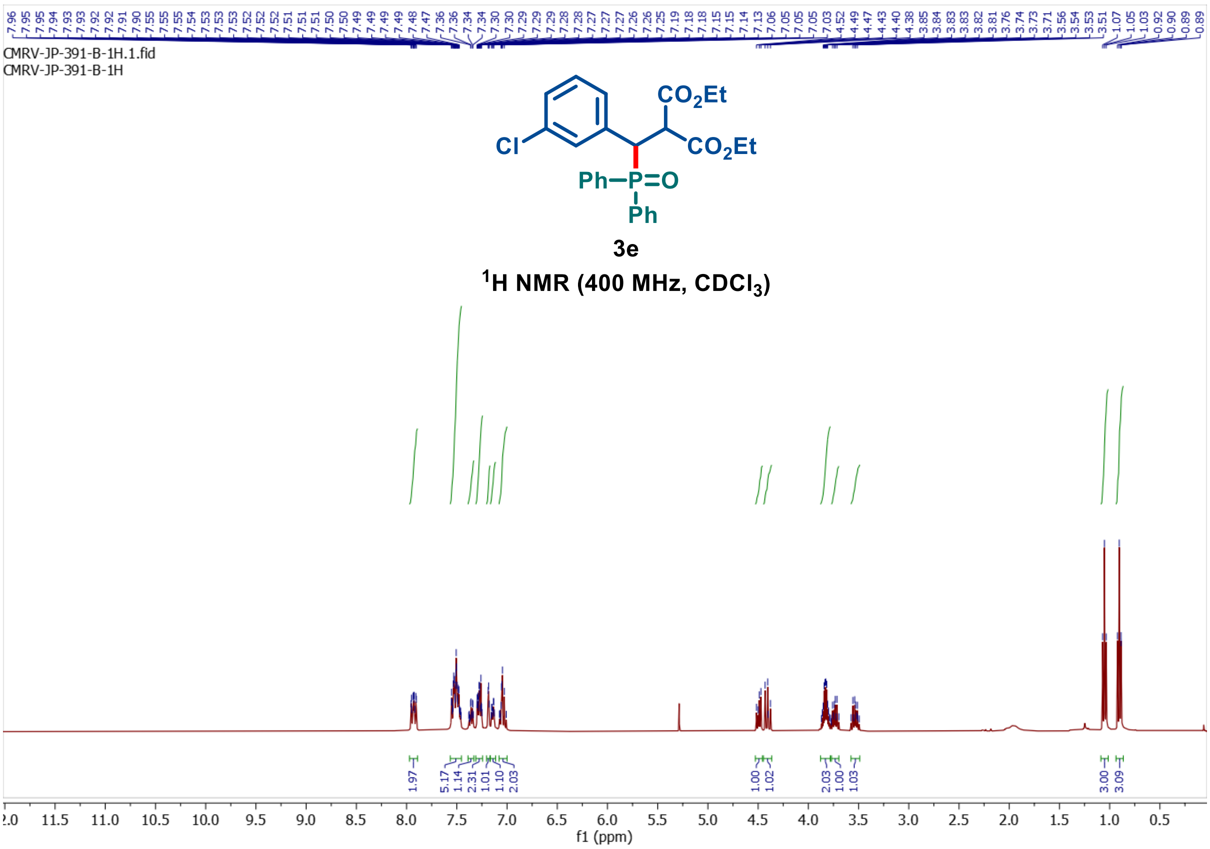
CMRV-JP-391-B-1H.1.fid
CMRV-JP-391-B-1H
Cl
CO2Et
CO2Et
Ph—P=O
Ph
3e
1H NMR (400 MHz, CDCl3)
f1 (ppm)

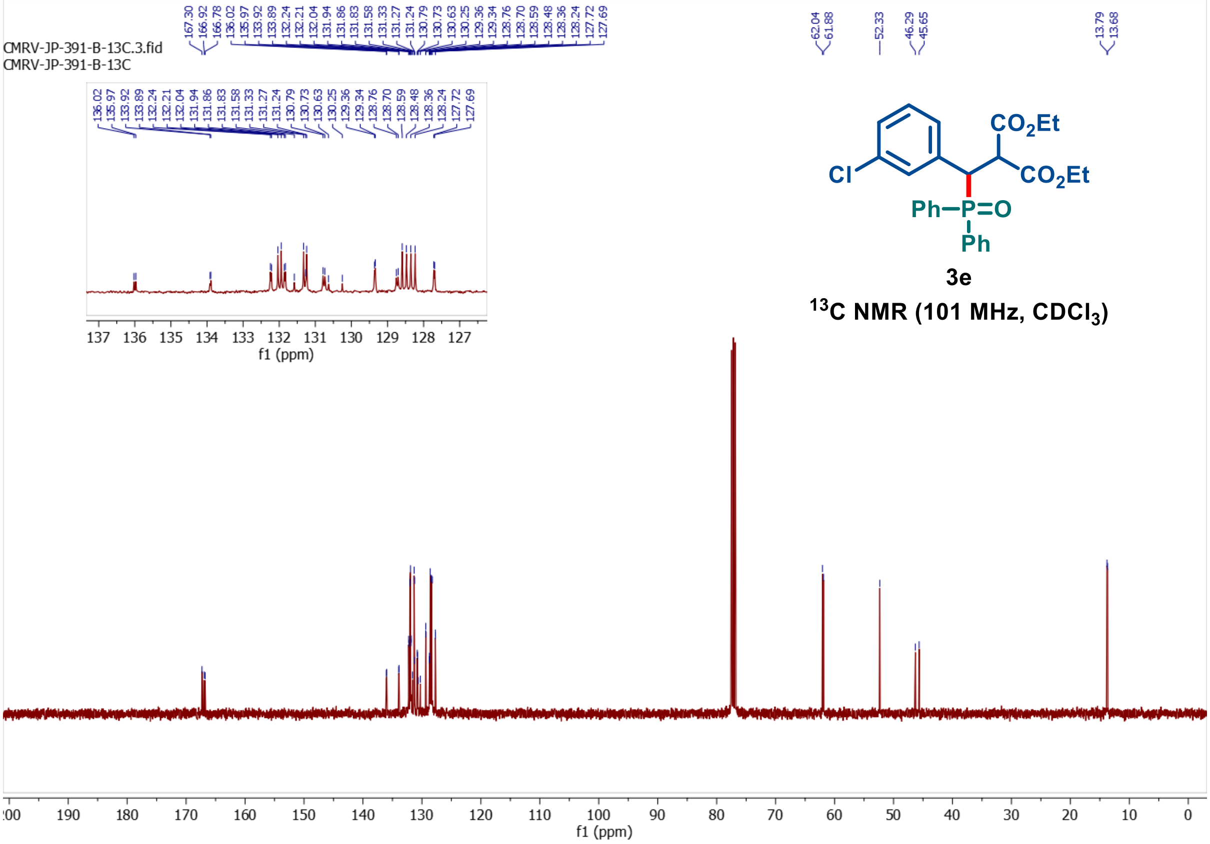
CMRV-JP-391-B-13C.3.fid
CMRV-JP-391-B-13C
Cl
CO2Et
CO2Et
Ph—P=O
Ph
3e
13C NMR (101 MHz, CDCl3)
f1 (ppm)

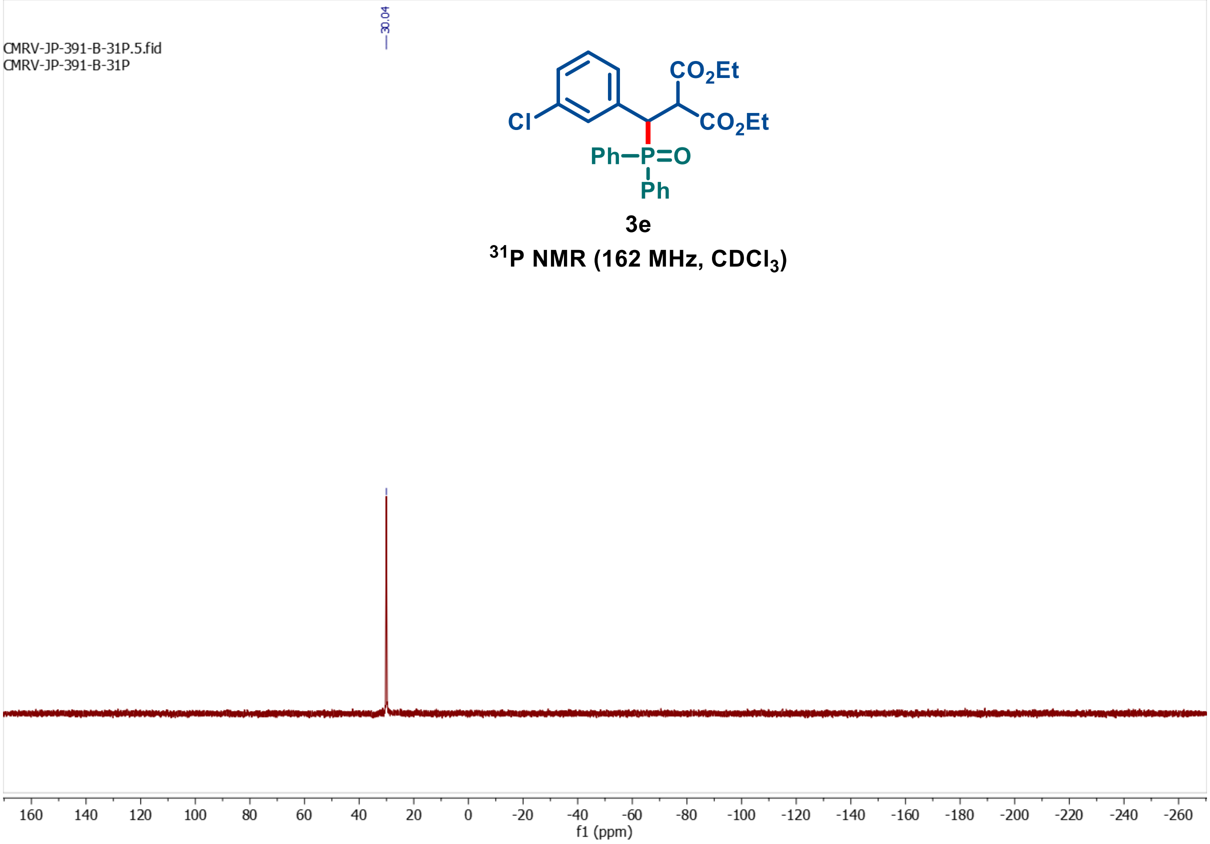
CMRV-JP-391-B-31P.5.fid
CMRV-JP-391-B-31P
30.04
CO2Et
CO2Et
Cl
Ph–P=O
Ph
3e
31P NMR (162 MHz, CDCl3)
f1 (ppm)

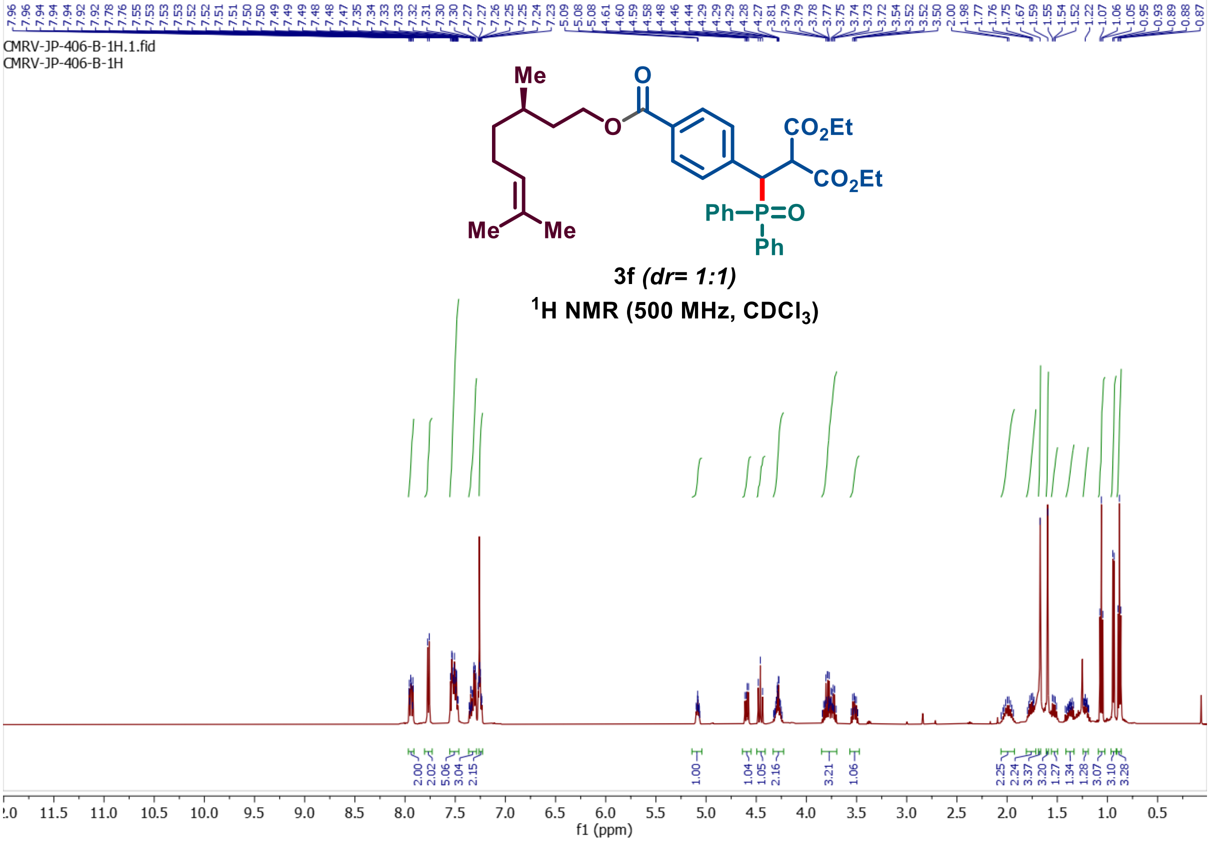
CMRV-JP-406-B-1H.1.fid
CMRV-JP-406-B-1H
Me
O
CO2Et
CO2Et
Me
Me
Ph–P=O
Ph
3f (dr= 1:1)
1H NMR (500 MHz, CDCl3)
2.00
2.02
5.06
3.04
2.15
1.00
1.04
1.05
2.16
3.21
1.06
2.25
2.24
3.37
3.20
1.27
1.34
1.28
3.07
3.10
3.28
f1 (ppm)

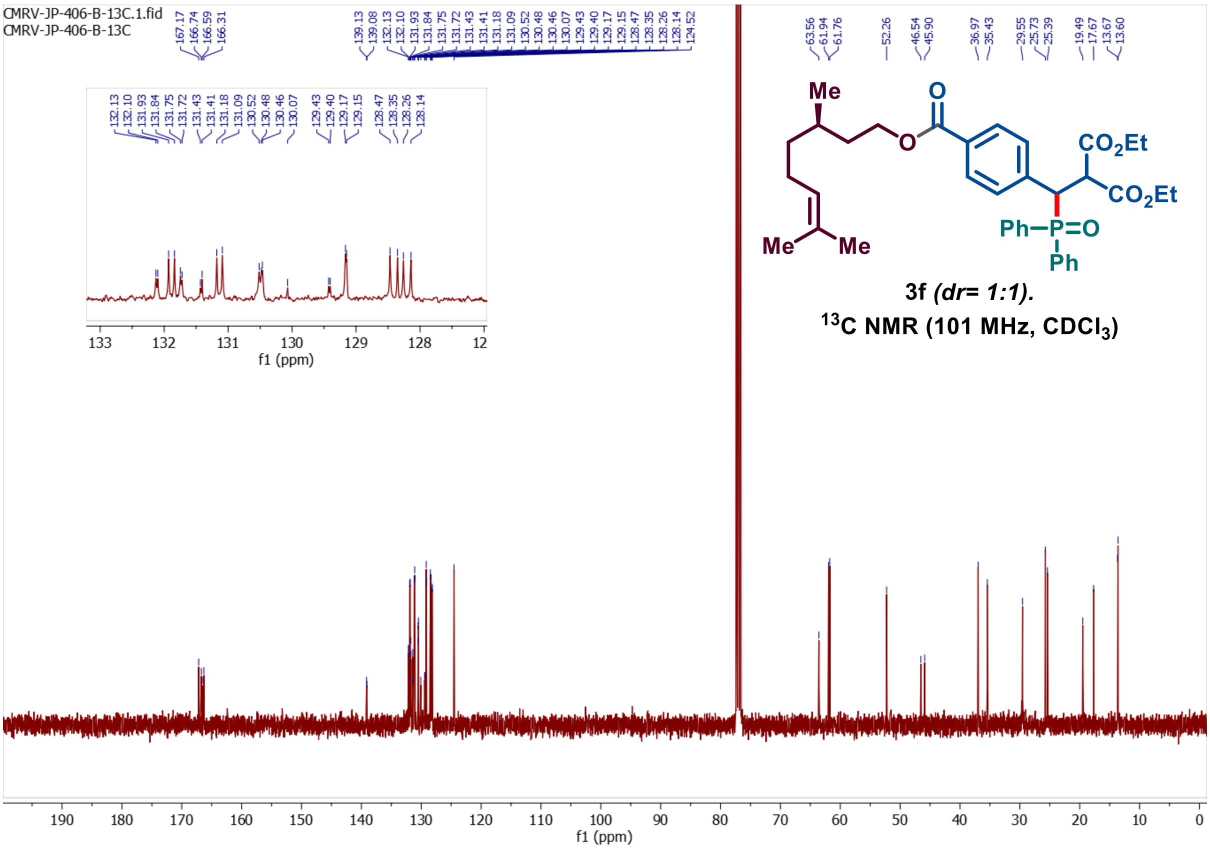
CMRV-JP-406-B-13C.1.fid
CMRV-JP-406-B-13C
3f (dr= 1:1).
^{13}C NMR (101 MHz, CDCl$_3$)
f1 (ppm)

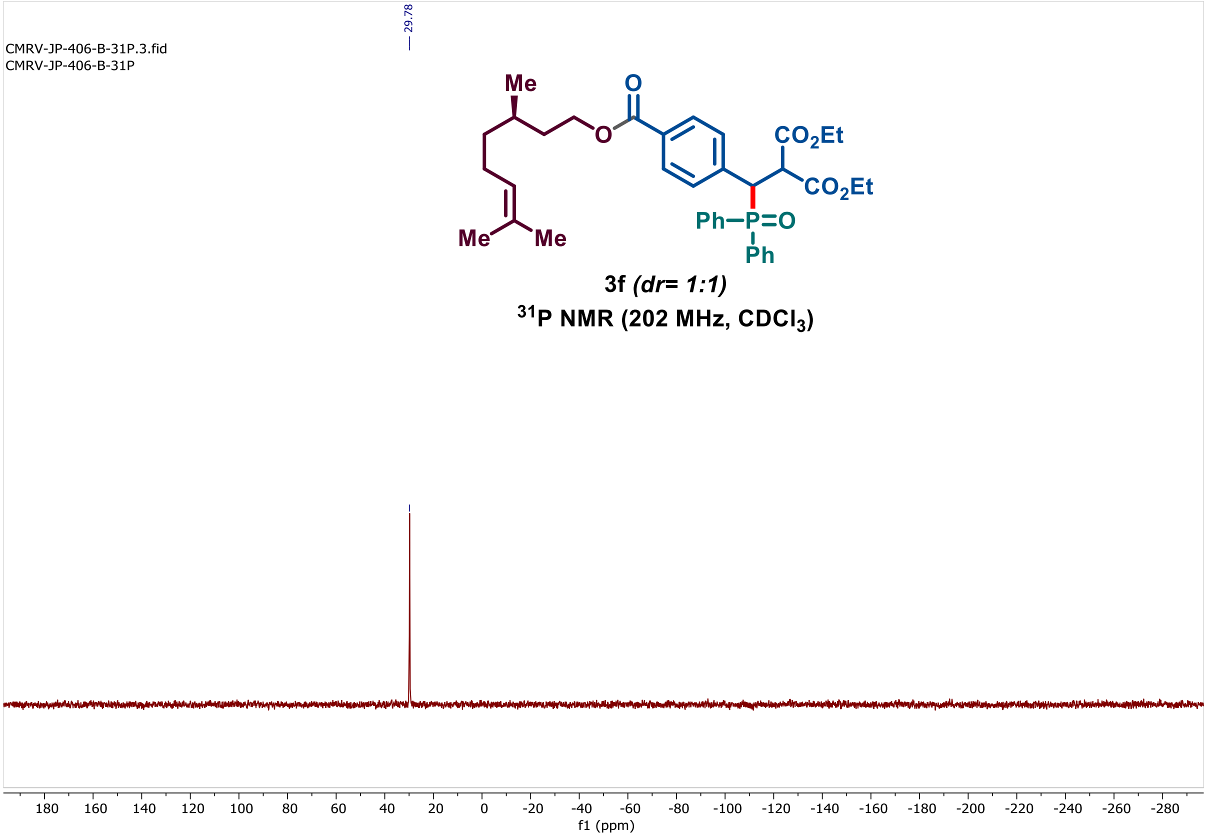
CMRV-JP-406-B-31P.3.fid
CMRV-JP-406-B-31P
29.78
3f (dr= 1:1)
^{31}P NMR (202 MHz, CDCl$_3$)
f1 (ppm)

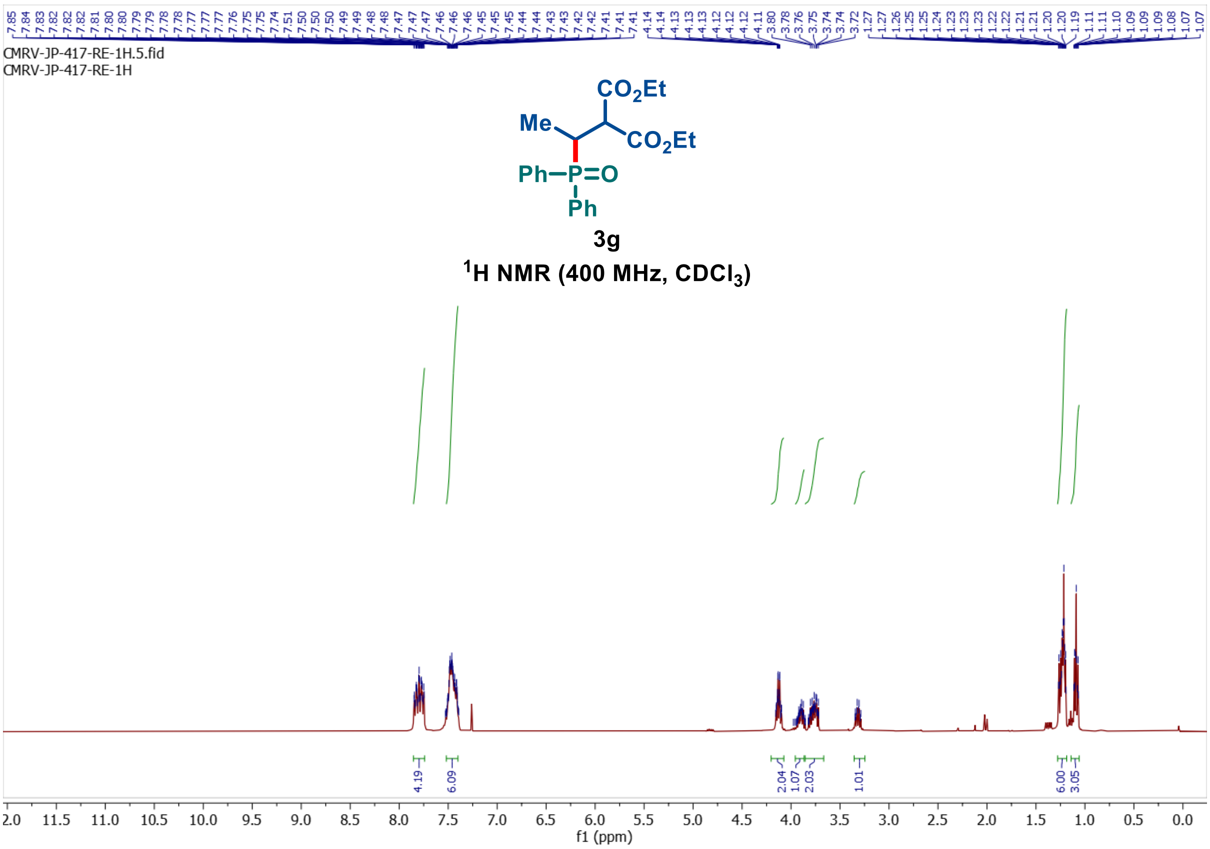
7.85 7.84 7.83 7.82 7.82 7.82 7.81 7.80 7.80 7.79 7.79 7.78 7.78 7.77 7.77 7.76 7.75 7.75 7.74 7.51 7.50 7.50 7.50 7.49 7.49 7.48 7.48 7.47 7.47 7.46 7.46 7.45 7.45 7.45 7.44 7.44 7.43 7.43 7.42 7.42 7.41 7.41 4.14 4.14 4.13 4.13 4.12 4.12 4.12 4.11 3.80 3.78 3.76 3.75 3.74 3.74 3.72 1.27 1.26 1.25 1.24 1.23 1.23 1.23 1.22 1.22 1.21 1.21 1.20 1.19 1.11 1.11 1.10 1.09 1.09 1.08 1.07 1.07
CMRV-JP-417-RE-1H.5.fid
CMRV-JP-417-RE-1H
CO2Et
Me
CO2Et
Ph–P=O
Ph
3g
1H NMR (400 MHz, CDCl3)
4.19 6.09 2.04 1.07 2.03 1.01 6.00 3.05
f1 (ppm)

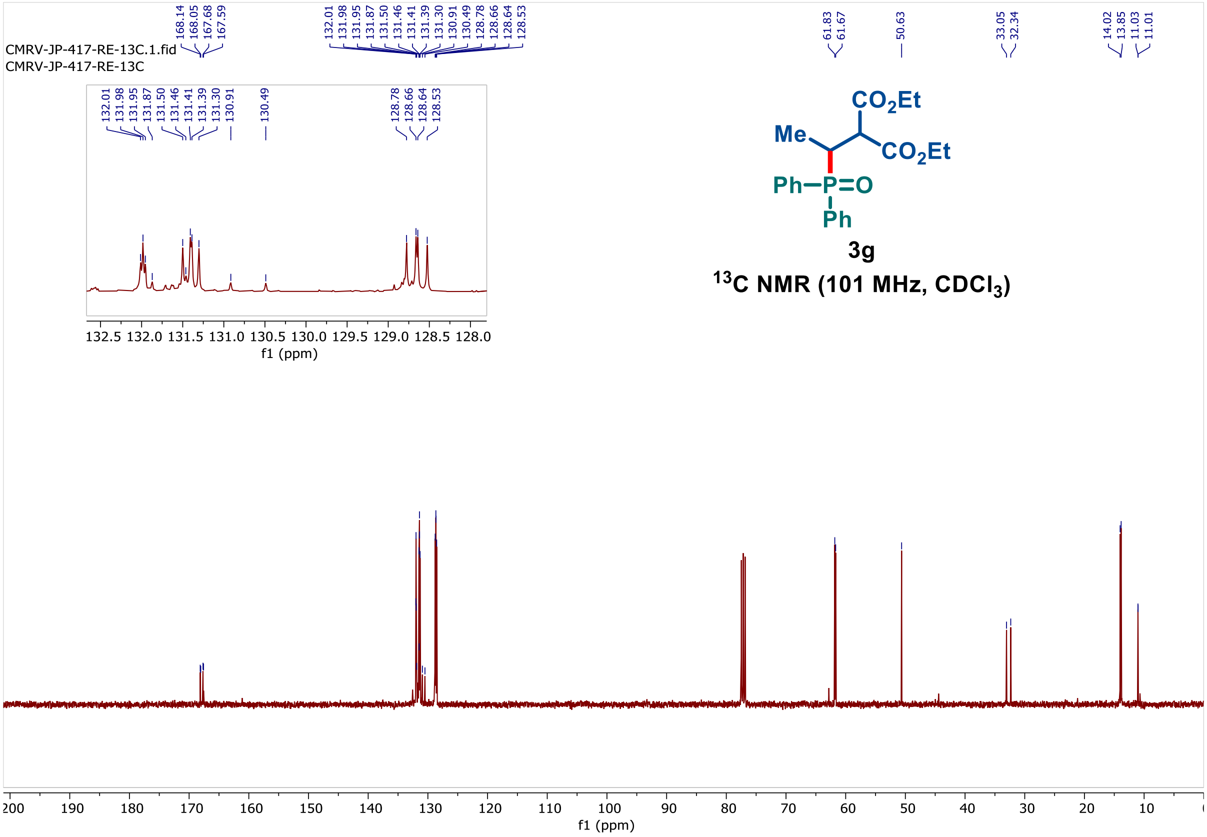
168.14 168.05 167.68 167.59
132.01 131.98 131.95 131.87 131.50 131.46 131.41 131.39 131.30 130.91 130.49 128.78 128.66 128.64 128.53
61.83 61.67
50.63
33.05 32.34
14.02 13.85 11.03 11.01
CMRV-JP-417-RE-13C.1.fid
CMRV-JP-417-RE-13C
f1 (ppm)
CO2Et
Me
CO2Et
Ph–P=O
Ph
3g
13C NMR (101 MHz, CDCl3)

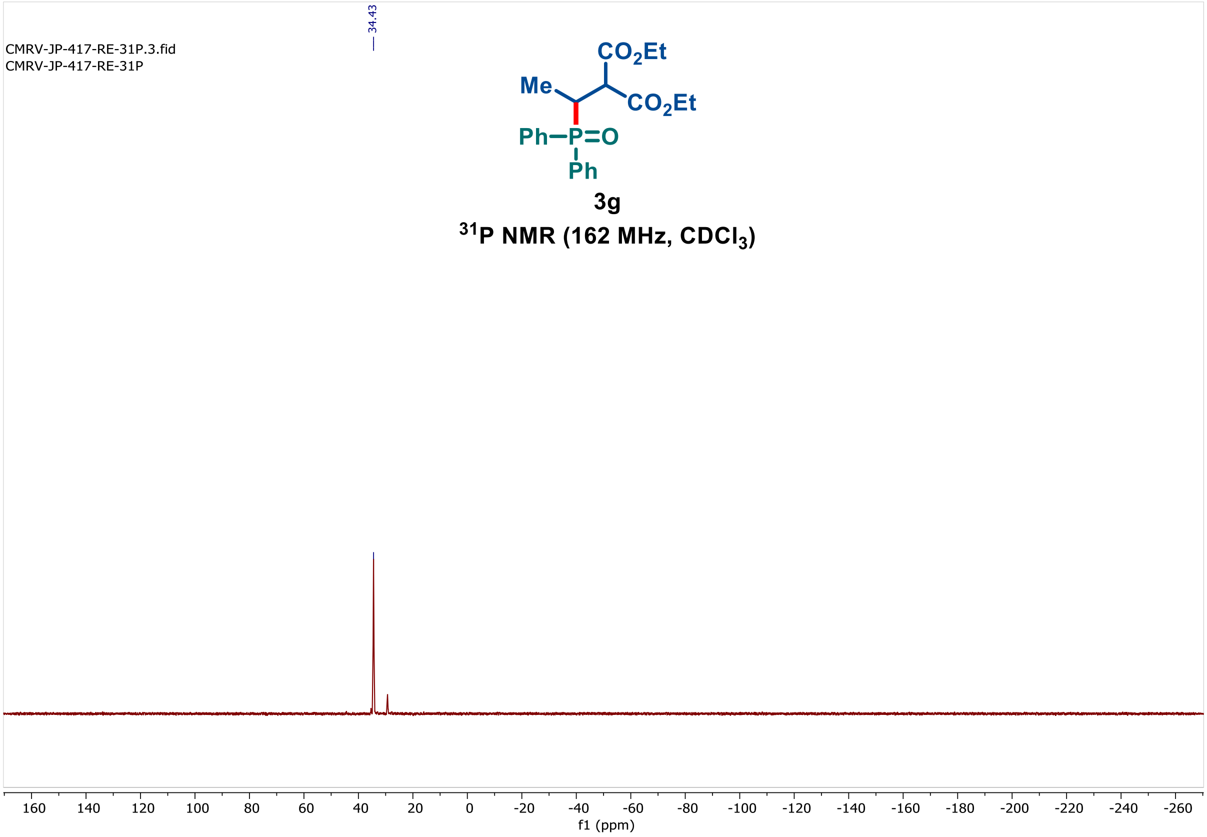
34.43
CMRV-JP-417-RE-31P.3.fid
CMRV-JP-417-RE-31P
CO2Et
Me
CO2Et
Ph–P=O
Ph
3g
31P NMR (162 MHz, CDCl3)
160 140 120 100 80 60 40 20 0 -20 -40 -60 -80 -100 -120 -140 -160 -180 -200 -220 -240 -260
f1 (ppm)

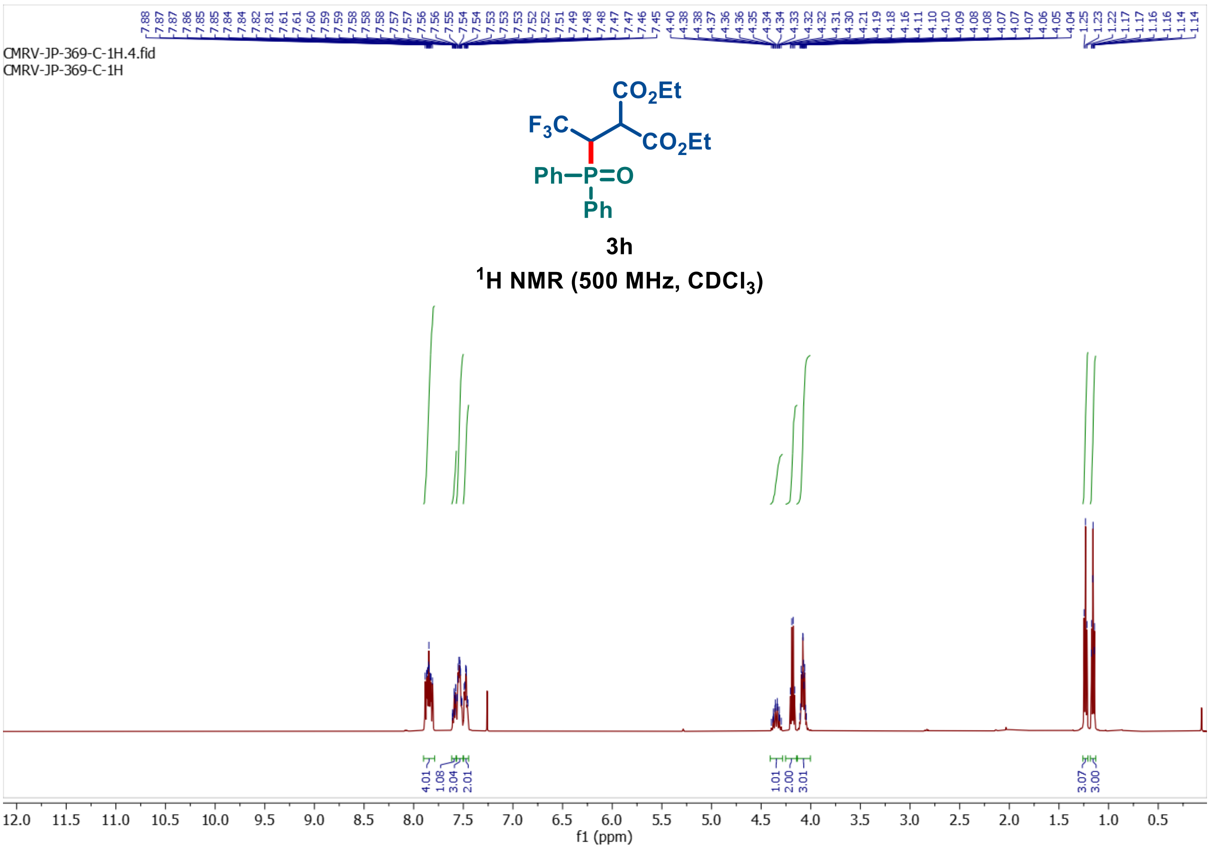
7.88 7.87 7.87 7.86 7.85 7.85 7.84 7.84 7.82 7.81 7.61 7.60 7.59 7.59 7.58 7.58 7.58 7.57 7.57 7.56 7.56 7.55 7.54 7.54 7.53 7.53 7.53 7.52 7.52 7.51 7.49 7.48 7.48 7.47 7.46 7.45 4.40 4.38 4.38 4.37 4.36 4.36 4.35 4.34 4.34 4.33 4.32 4.32 4.31 4.30 4.21 4.19 4.18 4.16 4.11 4.10 4.10 4.09 4.08 4.08 4.07 4.07 4.07 4.06 4.05 4.04 1.25 1.23 1.22 1.17 1.17 1.16 1.16 1.14
CMRV-JP-369-C-1H.4.fid
CMRV-JP-369-C-1H
CO2Et
F3C
CO2Et
Ph–P=O
Ph
3h
1H NMR (500 MHz, CDCl3)
4.01 1.08 3.04 2.01
1.01 2.00 3.01
3.07 3.00
12.0 11.5 11.0 10.5 10.0 9.5 9.0 8.5 8.0 7.5 7.0 6.5 6.0 5.5 5.0 4.5 4.0 3.5 3.0 2.5 2.0 1.5 1.0 0.5
f1 (ppm)

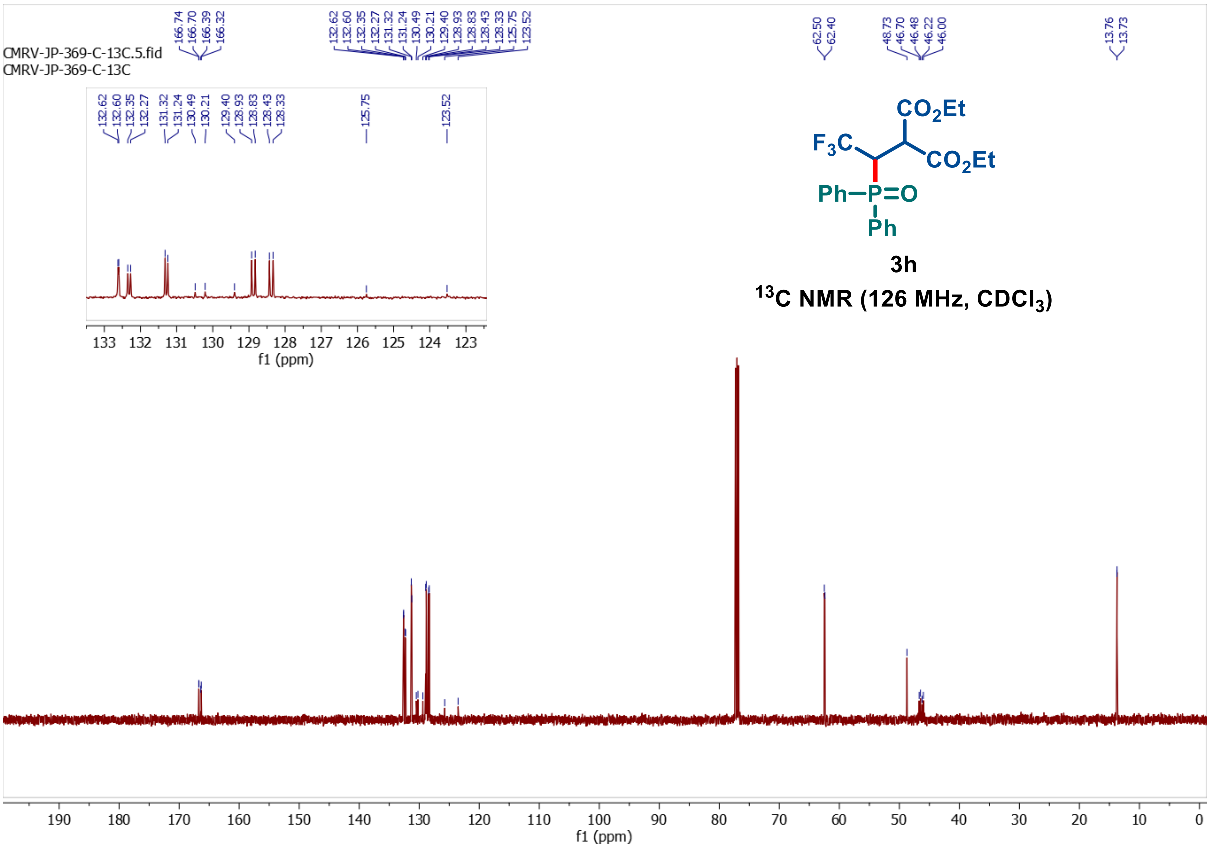
CMRV-JP-369-C-13C.5.fid
CMRV-JP-369-C-13C
166.74
166.70
166.39
166.32
132.62
132.60
132.35
132.27
131.32
131.24
130.49
130.21
129.40
128.93
128.83
128.43
128.33
125.75
123.52
62.50
62.40
48.73
46.70
46.48
46.22
46.00
13.76
13.73
CO_2Et
F_3C
CO_2Et
Ph–P=O
Ph
3h
^{13}C NMR (126 MHz, $CDCl_3$)
f1 (ppm)

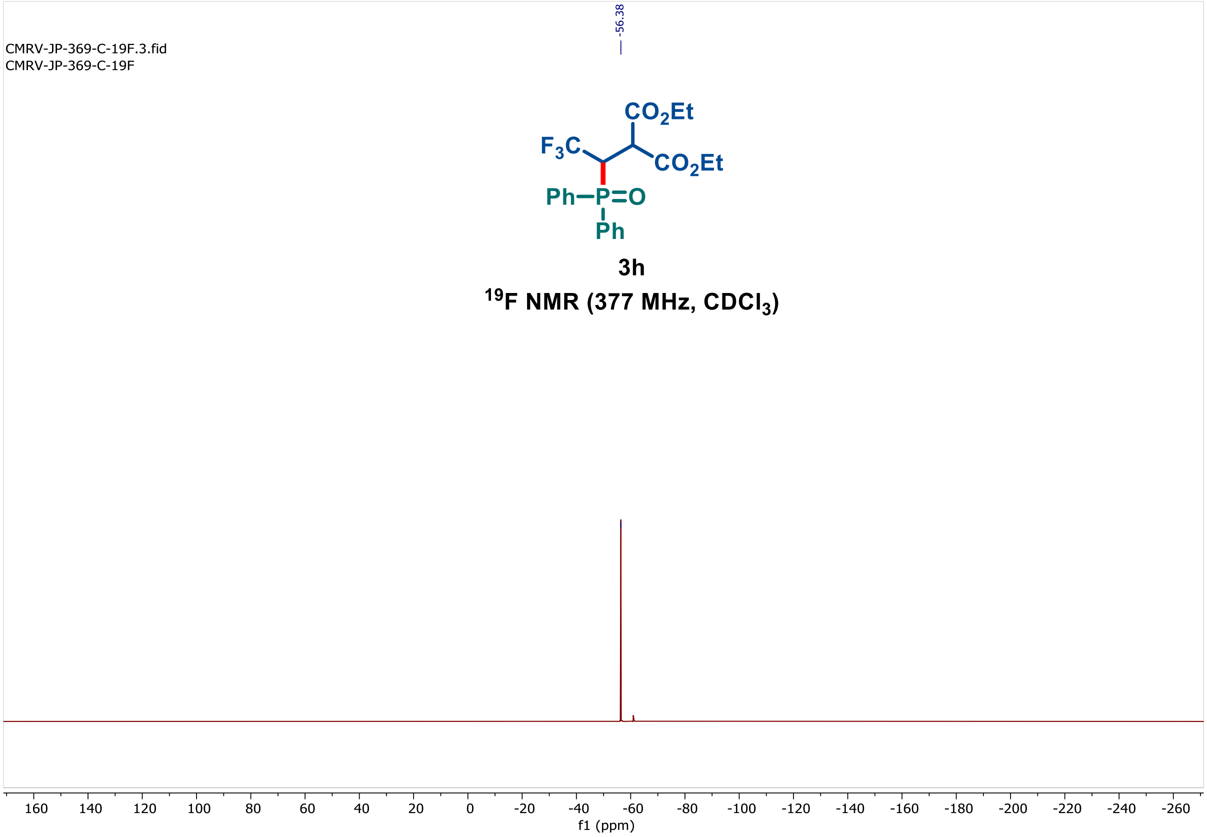
CMRV-JP-369-C-19F.3.fid
CMRV-JP-369-C-19F
-56.38
CO_2Et
F_3C
CO_2Et
Ph–P=O
Ph
3h
^{19}F NMR (377 MHz, $CDCl_3$)
f1 (ppm)

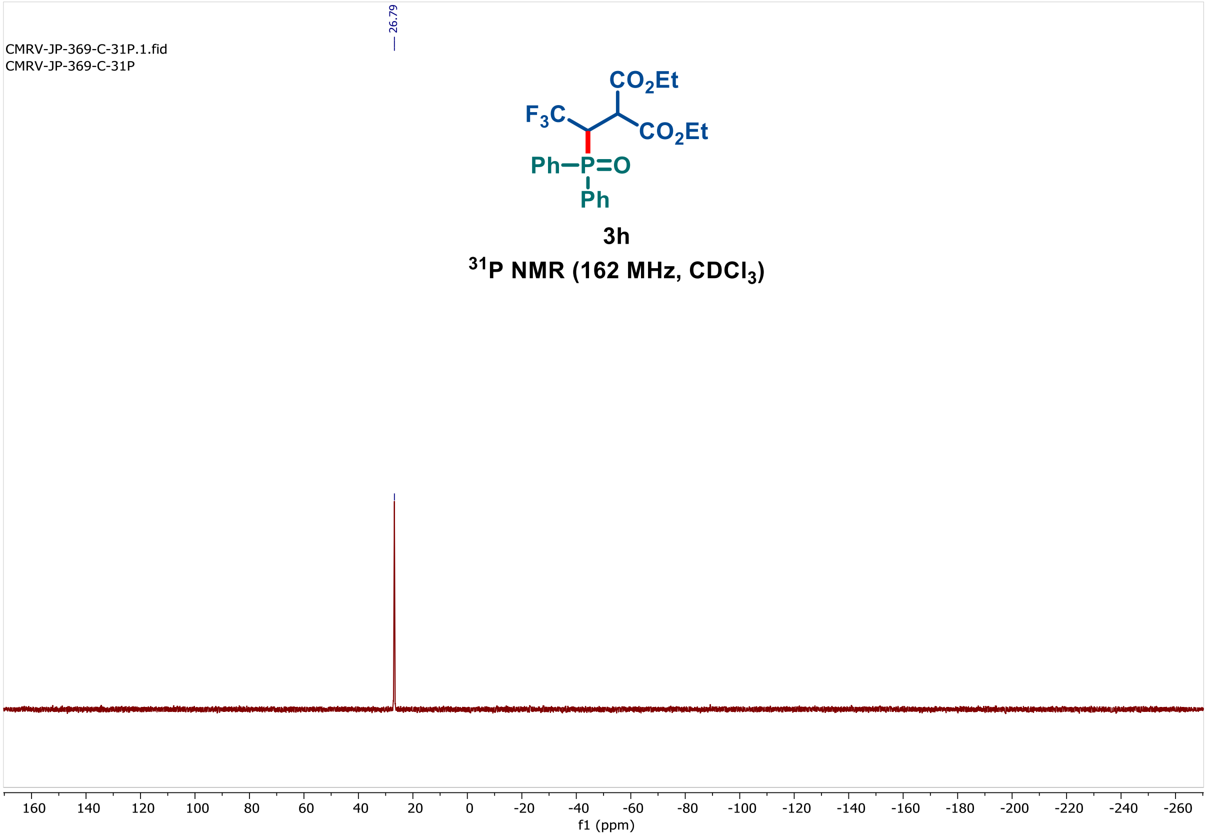
CMRV-JP-369-C-31P.1.fid
CMRV-JP-369-C-31P
26.79
3h
31P NMR (162 MHz, CDCl3)
f1 (ppm)

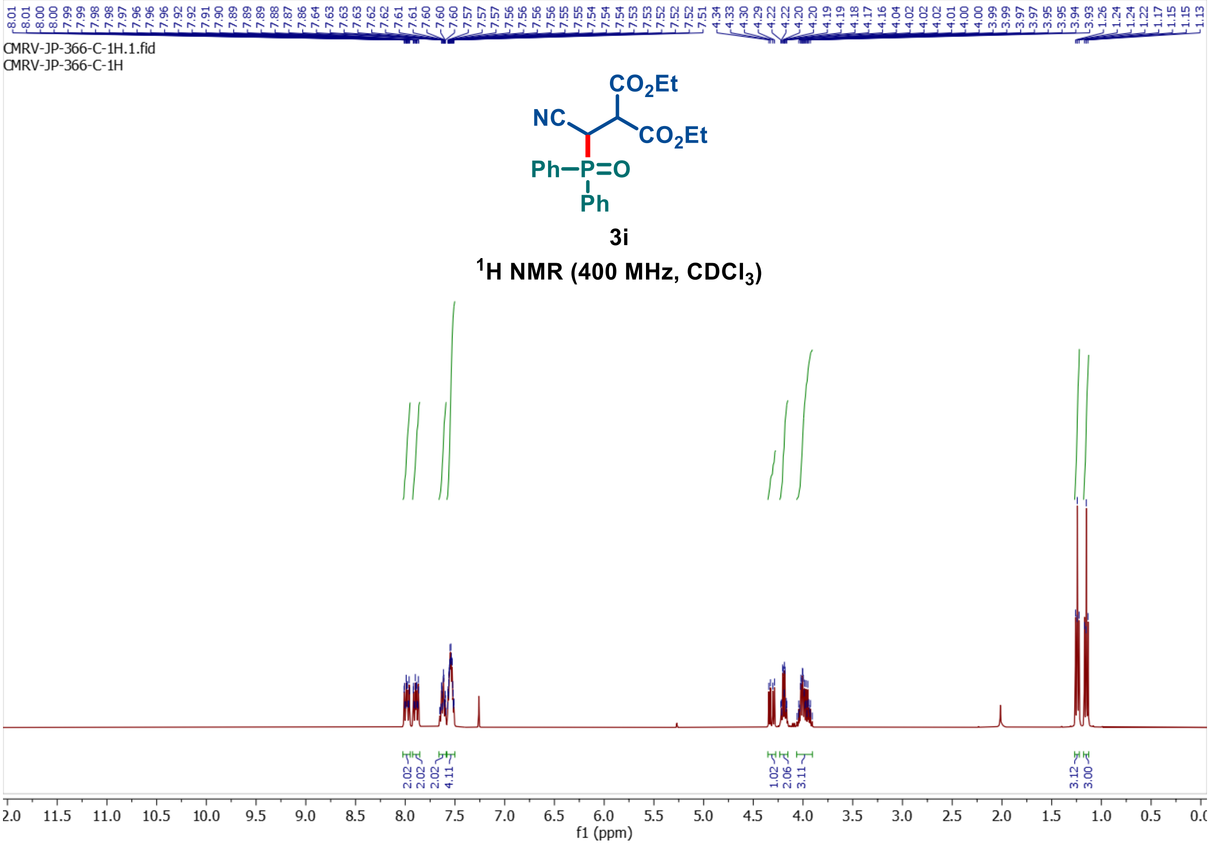
CMRV-JP-366-C-1H.1.fid
CMRV-JP-366-C-1H
3i
1H NMR (400 MHz, CDCl3)
f1 (ppm)

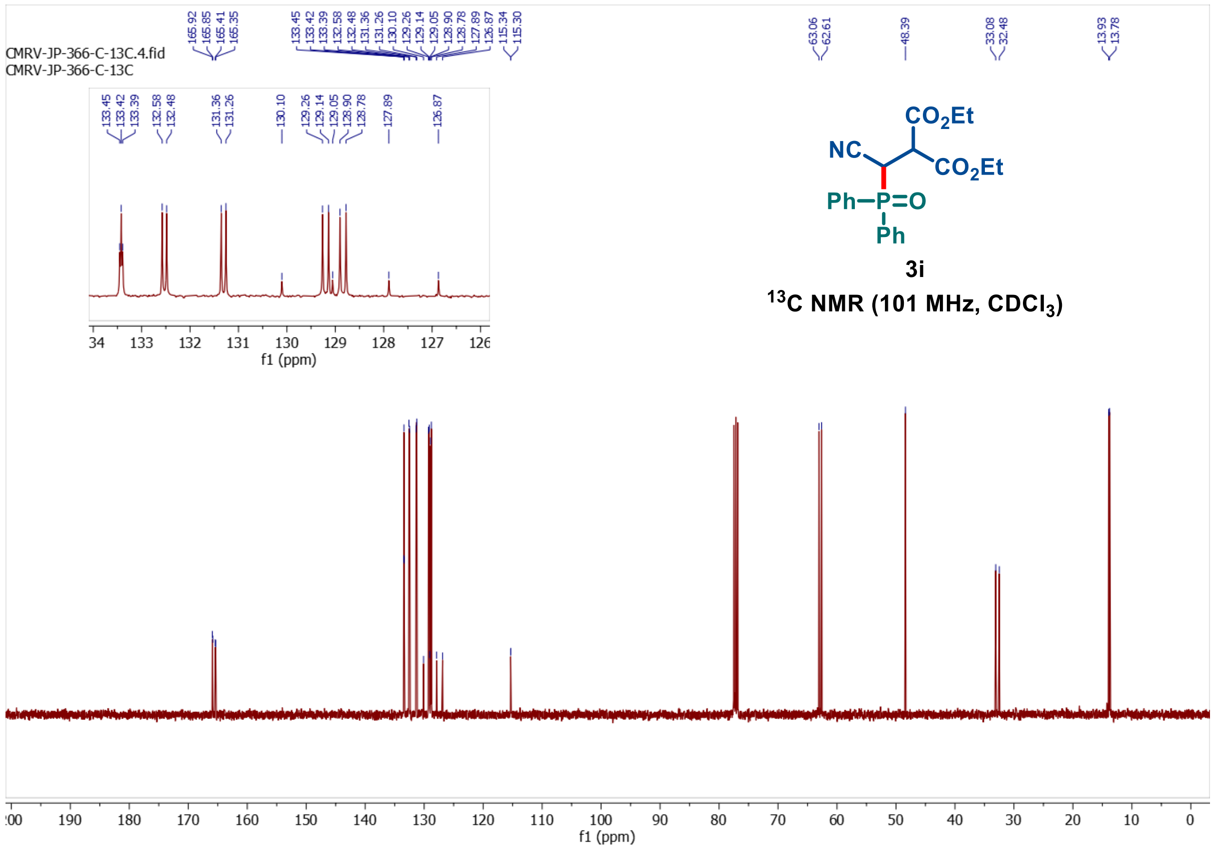
CMRV-JP-366-C-13C.4.fid
CMRV-JP-366-C-13C
165.92
165.85
165.41
165.35
133.45
133.42
133.39
132.58
132.48
131.36
131.26
130.10
129.26
129.14
129.05
128.90
128.78
127.89
126.87
115.34
115.30
63.06
62.61
48.39
33.08
32.48
13.93
13.78
CO2Et
NC
CO2Et
Ph–P=O
Ph
3i
13C NMR (101 MHz, CDCl3)
f1 (ppm)

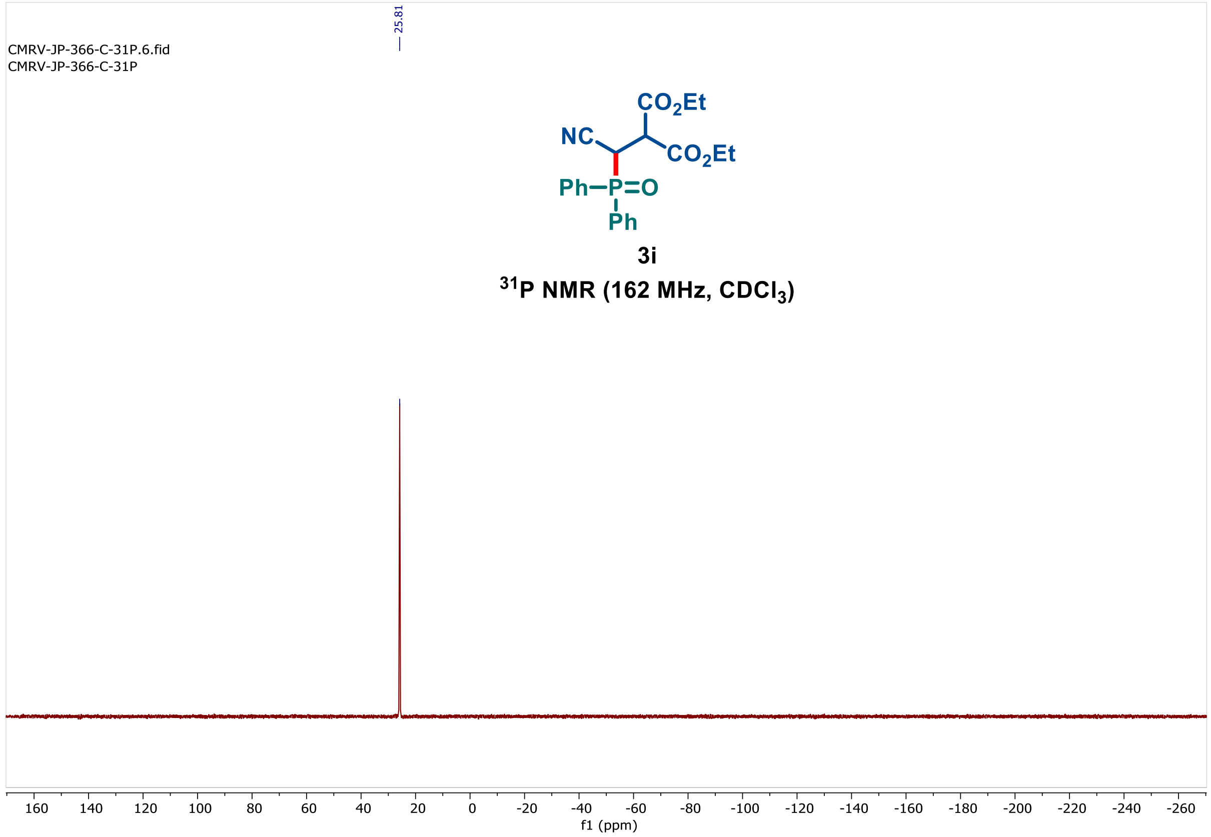
CMRV-JP-366-C-31P.6.fid
CMRV-JP-366-C-31P
25.81
CO2Et
NC
CO2Et
Ph–P=O
Ph
3i
31P NMR (162 MHz, CDCl3)
f1 (ppm)

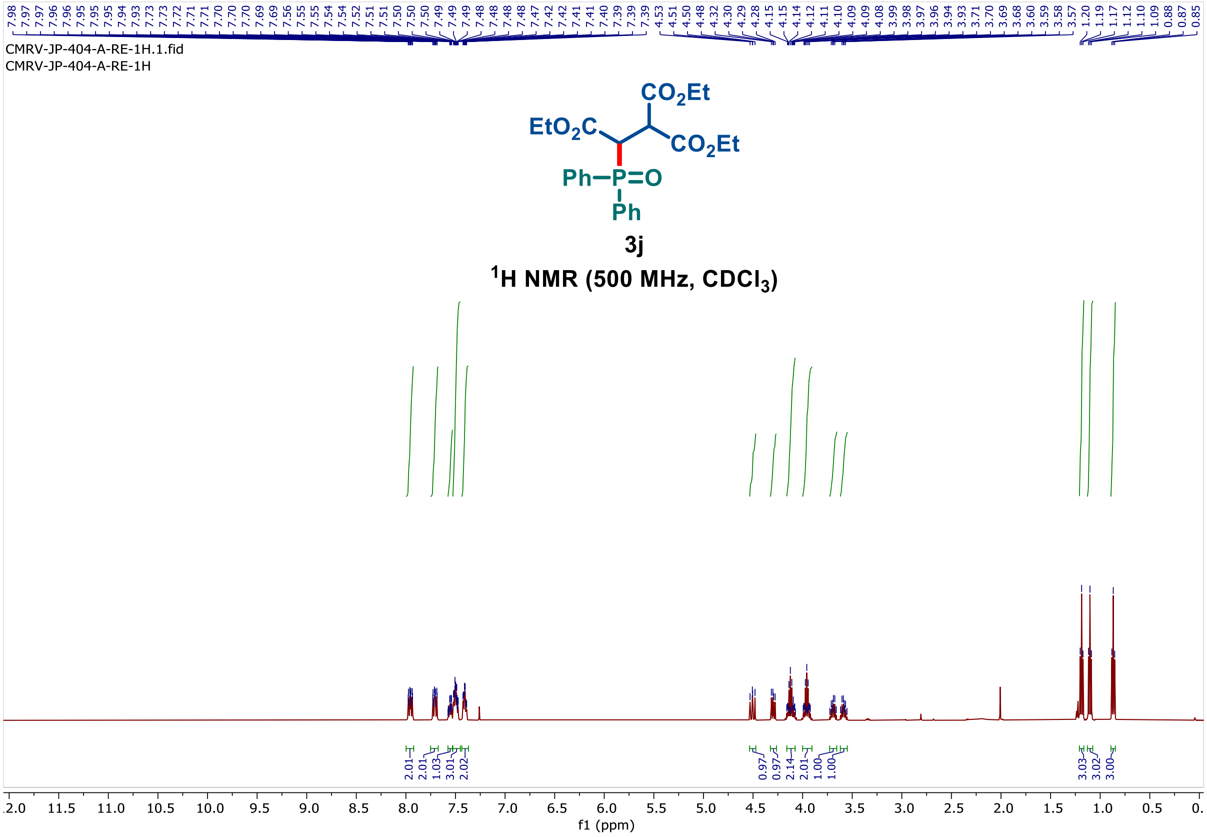
7.98 7.97 7.97 7.96 7.96 7.95 7.95 7.95 7.94 7.93 7.73 7.73 7.72 7.71 7.71 7.70 7.70 7.69 7.69 7.56 7.55 7.55 7.54 7.54 7.52 7.51 7.51 7.50 7.50 7.50 7.49 7.49 7.49 7.48 7.48 7.48 7.47 7.42 7.42 7.41 7.41 7.40 7.39 7.39 7.39 4.53 4.51 4.50 4.48 4.32 4.30 4.29 4.28 4.15 4.15 4.14 4.12 4.11 4.10 4.09 4.09 4.08 3.99 3.98 3.97 3.96 3.94 3.93 3.71 3.70 3.69 3.68 3.60 3.59 3.58 3.57 1.20 1.19 1.17 1.12 1.10 1.09 0.88 0.87 0.85
CMRV-JP-404-A-RE-1H.1.fid
CMRV-JP-404-A-RE-1H
CO_2Et
EtO_2C
CO_2Et
Ph—P=O
Ph
3j
^1H NMR (500 MHz, $CDCl_3$)
2.01 2.01 1.03 3.01 2.02 0.97 0.97 2.14 2.01 1.00 1.00 3.03 3.02 3.00
f1 (ppm)

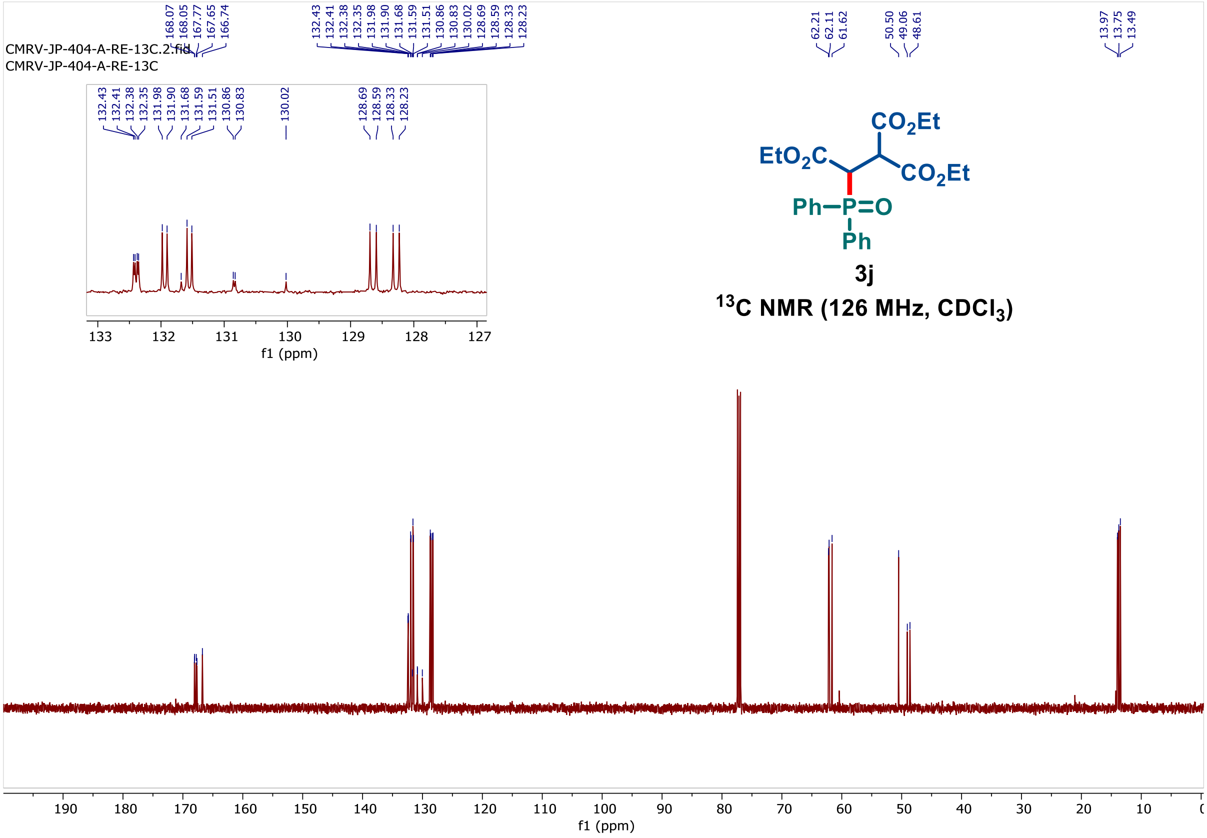
168.07 168.05 167.77 167.65 166.74 132.43 132.41 132.38 132.35 131.98 131.90 131.68 131.59 131.51 130.86 130.83 130.02 128.69 128.59 128.33 128.23 62.21 62.11 61.62 50.50 49.06 48.61 13.97 13.75 13.49
CMRV-JP-404-A-RE-13C.2.fid
CMRV-JP-404-A-RE-13C
f1 (ppm)
CO_2Et
EtO_2C
CO_2Et
Ph—P=O
Ph
3j
^{13}C NMR (126 MHz, $CDCl_3$)
f1 (ppm)

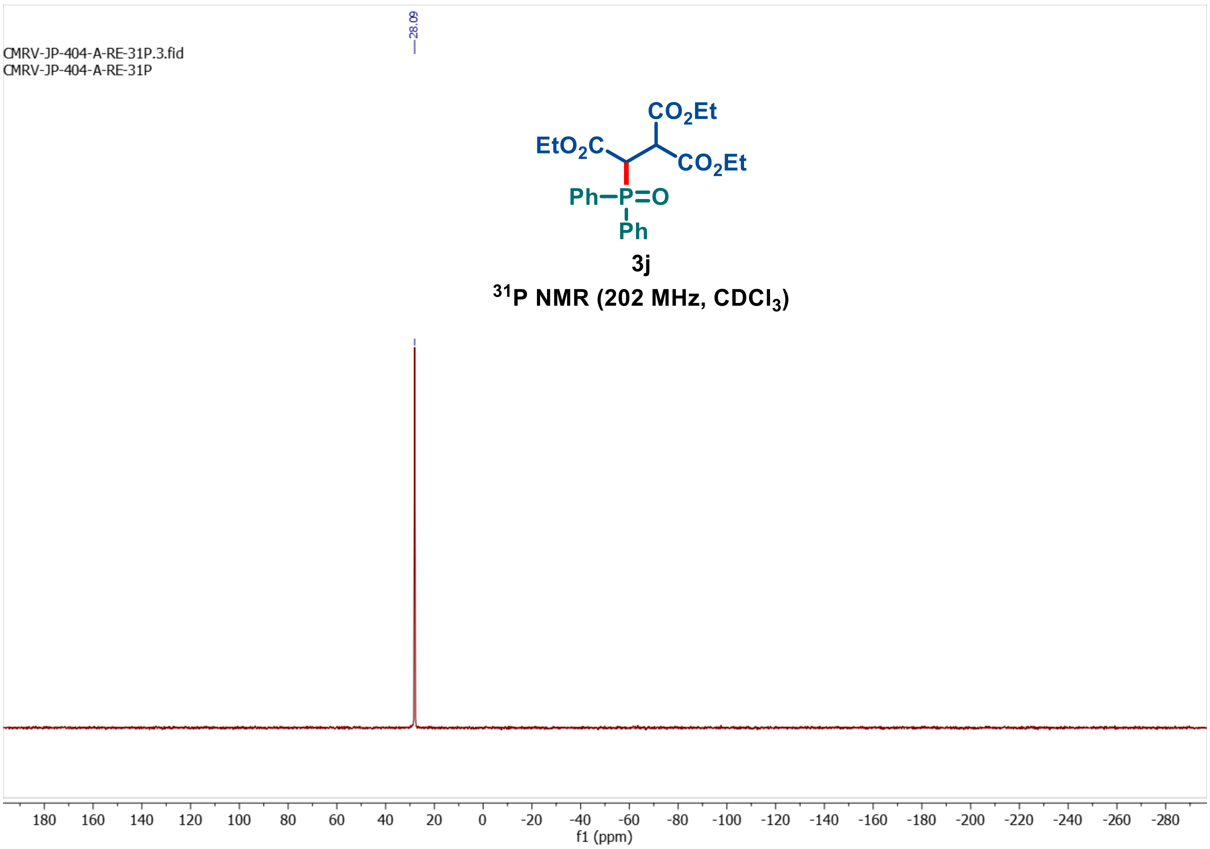
CMRV-JP-404-A-RE-31P.3.fid
CMRV-JP-404-A-RE-31P
28.09
CO2Et
EtO2C
CO2Et
Ph—P=O
Ph
3j
31P NMR (202 MHz, CDCl3)
180 160 140 120 100 80 60 40 20 0 -20 -40 -60 -80 -100 -120 -140 -160 -180 -200 -220 -240 -260 -280
f1 (ppm)

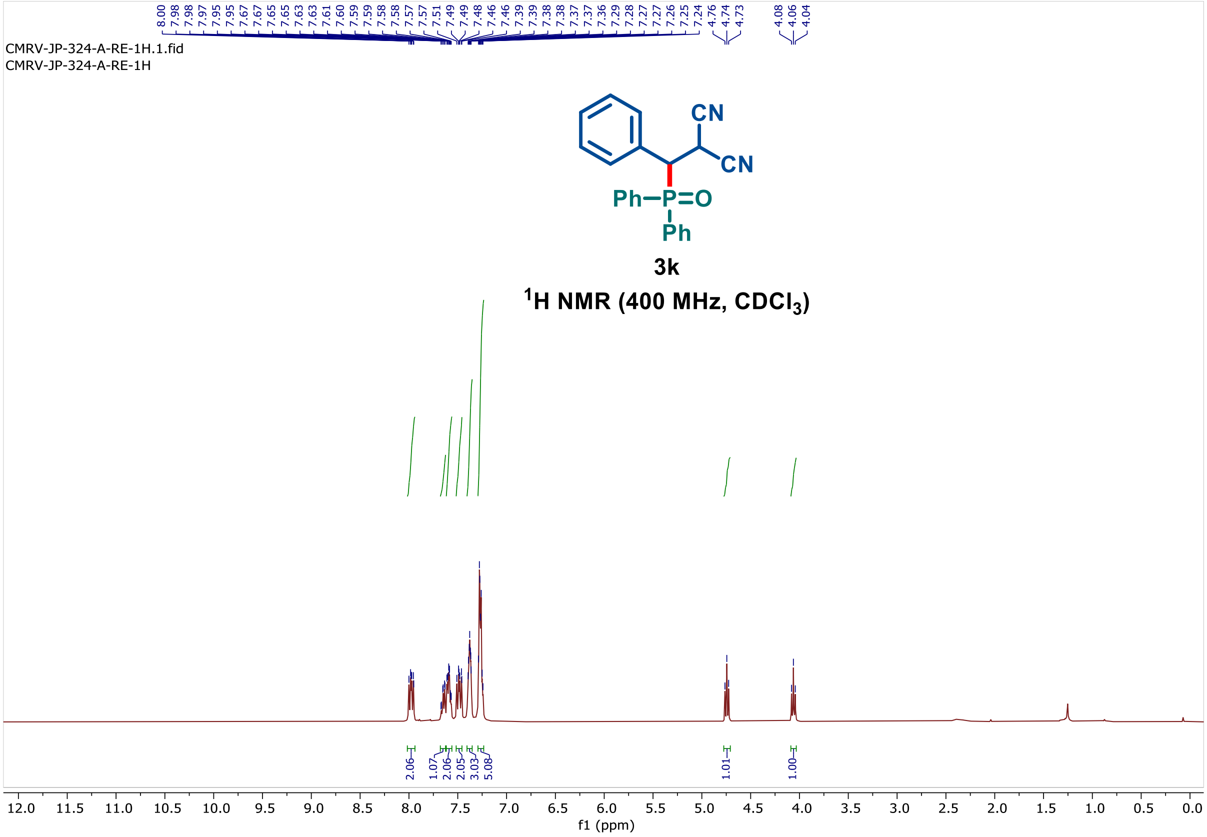
8.00 7.98 7.98 7.97 7.95 7.95 7.67 7.67 7.65 7.65 7.63 7.63 7.61 7.60 7.59 7.59 7.58 7.58 7.57 7.57 7.51 7.49 7.49 7.48 7.46 7.46 7.39 7.39 7.38 7.38 7.37 7.37 7.36 7.29 7.28 7.27 7.27 7.26 7.25 7.24 4.76 4.74 4.73 4.08 4.06 4.04
CMRV-JP-324-A-RE-1H.1.fid
CMRV-JP-324-A-RE-1H
CN
CN
Ph—P=O
Ph
3k
1H NMR (400 MHz, CDCl3)
2.06 1.07 2.06 2.05 3.03 5.08 1.01 1.00
12.0 11.5 11.0 10.5 10.0 9.5 9.0 8.5 8.0 7.5 7.0 6.5 6.0 5.5 5.0 4.5 4.0 3.5 3.0 2.5 2.0 1.5 1.0 0.5 0.0
f1 (ppm)

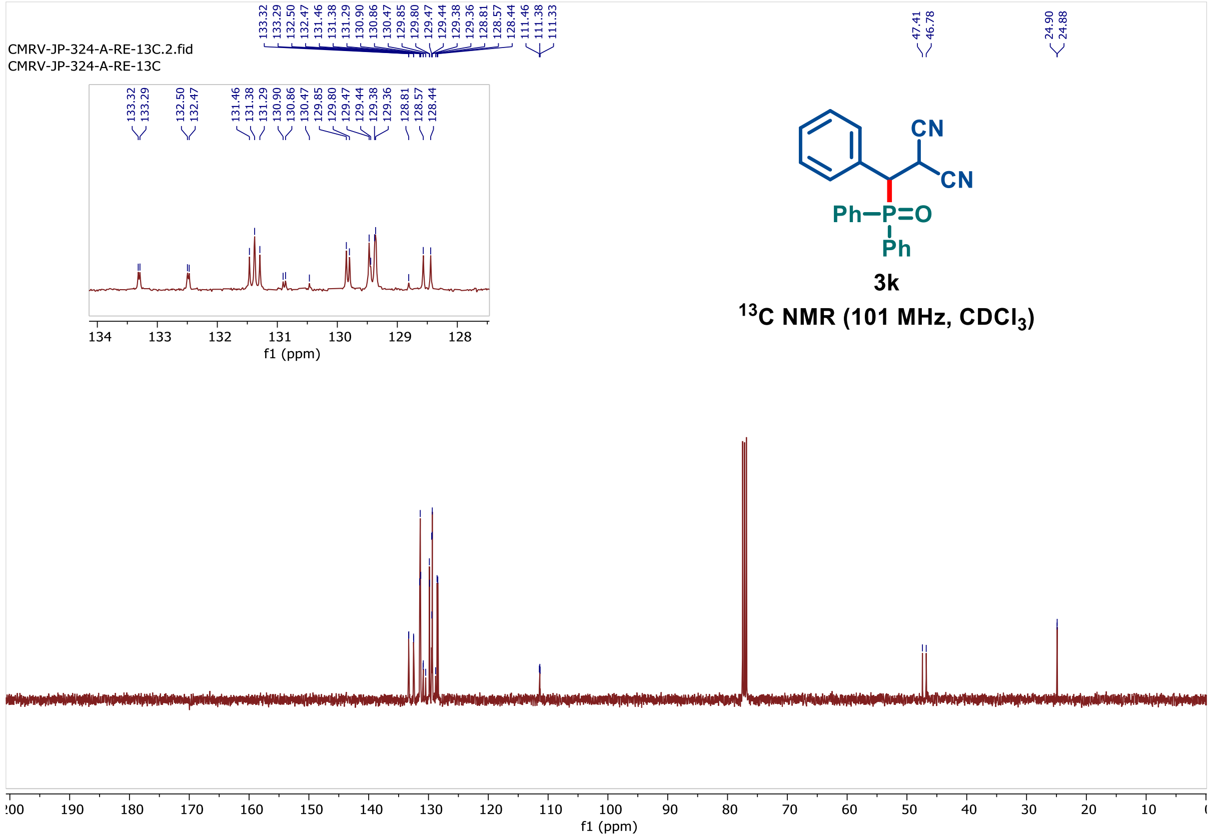
CMRV-JP-324-A-RE-13C.2.fid
CMRV-JP-324-A-RE-13C
133.32 133.29 132.50 132.47 131.46 131.38 131.29 130.90 130.86 130.47 129.85 129.80 129.47 129.44 129.38 129.36 128.81 128.57 128.44 111.46 111.38 111.33
47.41 46.78
24.90 24.88
CN
CN
Ph
P
O
Ph
3k
13C NMR (101 MHz, CDCl3)
f1 (ppm)

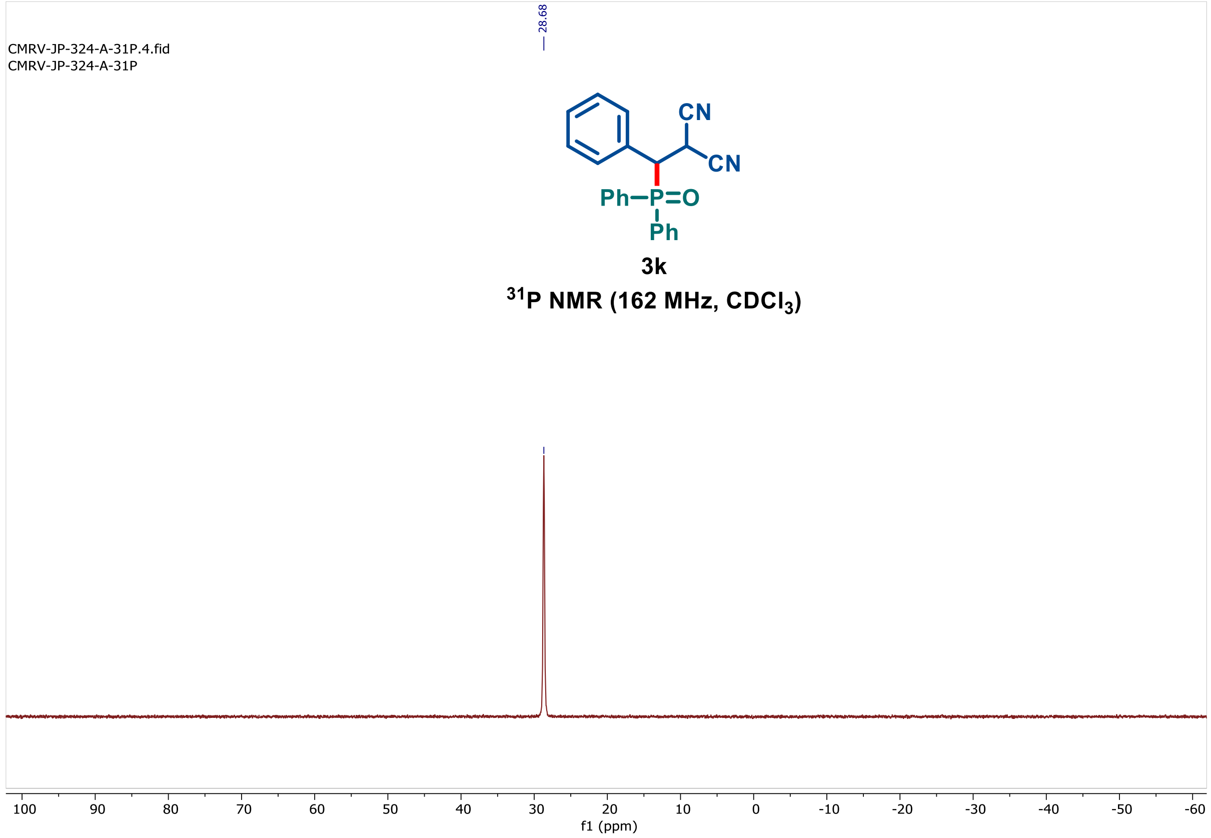
28.68
CMRV-JP-324-A-31P.4.fid
CMRV-JP-324-A-31P
CN
CN
Ph
P
O
Ph
3k
31P NMR (162 MHz, CDCl3)
f1 (ppm)

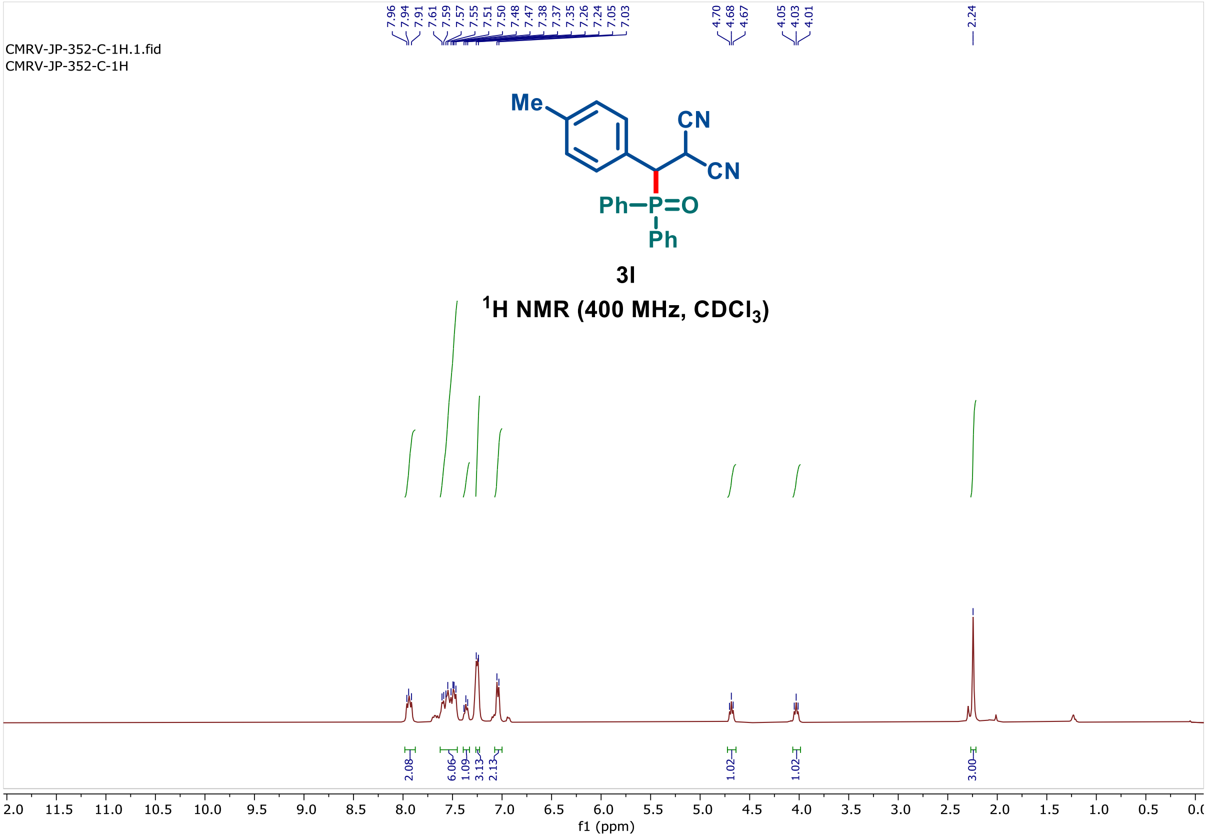
CMRV-JP-352-C-1H.1.fid
CMRV-JP-352-C-1H
7.96
7.94
7.91
7.61
7.59
7.57
7.55
7.51
7.50
7.48
7.47
7.38
7.37
7.35
7.26
7.24
7.05
7.03
4.70
4.68
4.67
4.05
4.03
4.01
2.24
Me
CN
CN
Ph—P=O
Ph
3l
1H NMR (400 MHz, CDCl3)
2.08
6.06
1.09
3.13
2.13
1.02
1.02
3.00
f1 (ppm)

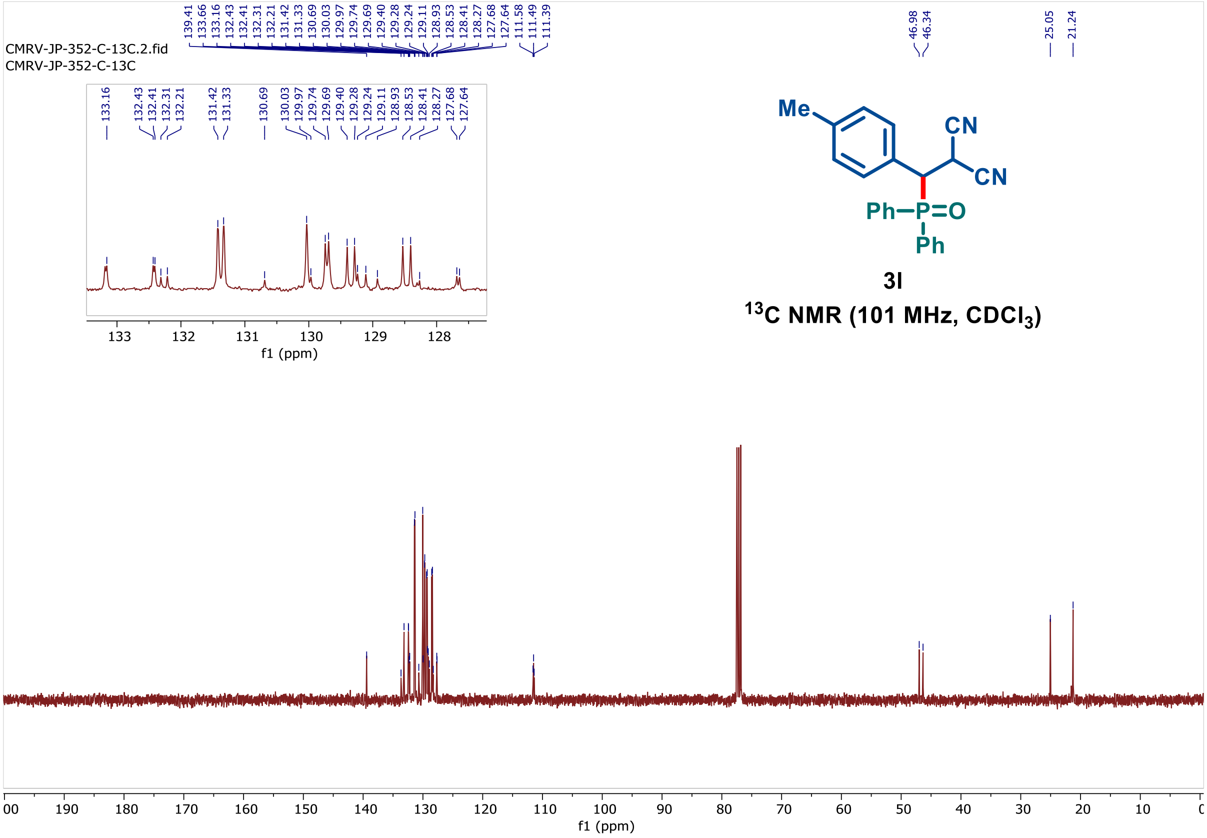
CMRV-JP-352-C-13C.2.fid
CMRV-JP-352-C-13C
139.41
133.66
133.16
132.43
132.41
132.31
132.21
131.42
131.33
130.69
130.03
129.97
129.74
129.69
129.40
129.28
129.24
129.11
128.93
128.53
128.41
128.27
127.68
127.64
111.58
111.49
111.39
46.98
46.34
25.05
21.24
Me
CN
CN
Ph—P=O
Ph
3l
13C NMR (101 MHz, CDCl3)
f1 (ppm)

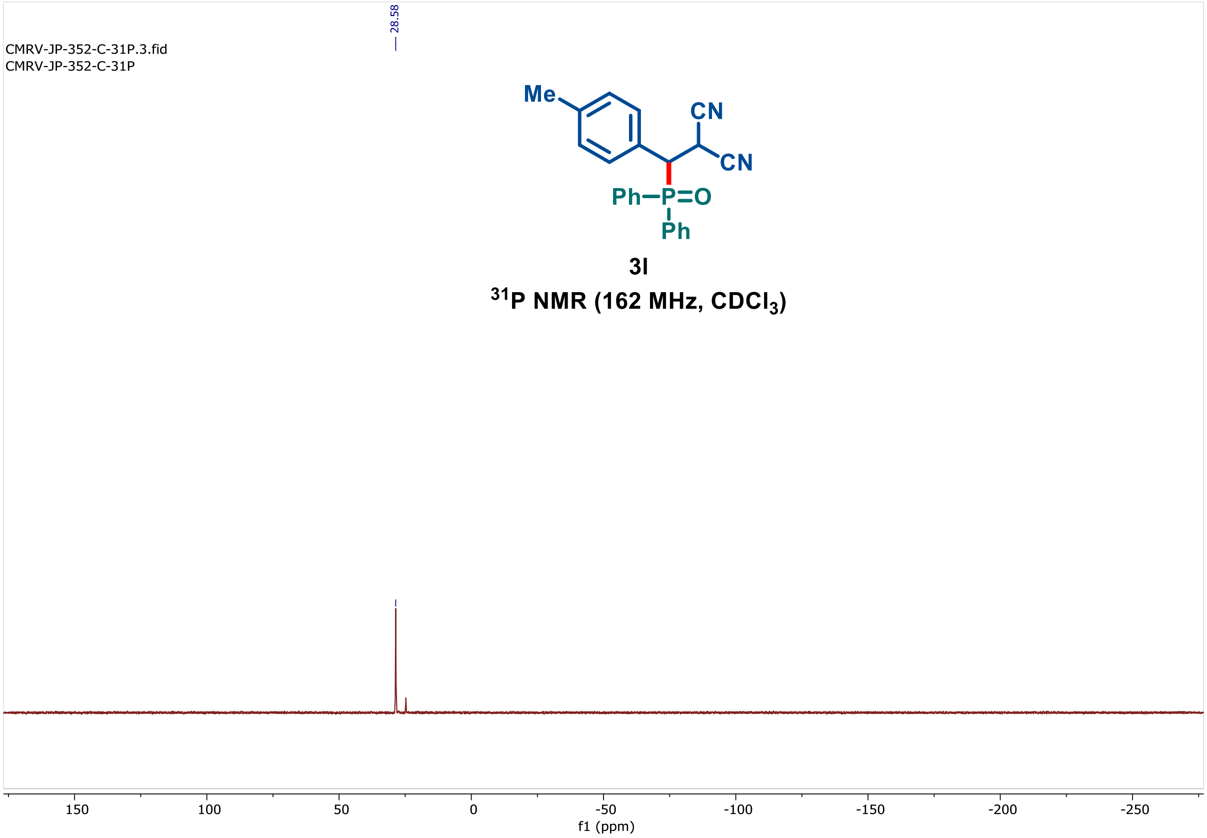

CMRV-JP-352-C-31P.3.fid
CMRV-JP-352-C-31P
28.58
Me
CN
CN
Ph—P=O
Ph
3l
31P NMR (162 MHz, CDCl3)
150 100 50 0 -50 -100 -150 -200 -250
f1 (ppm)

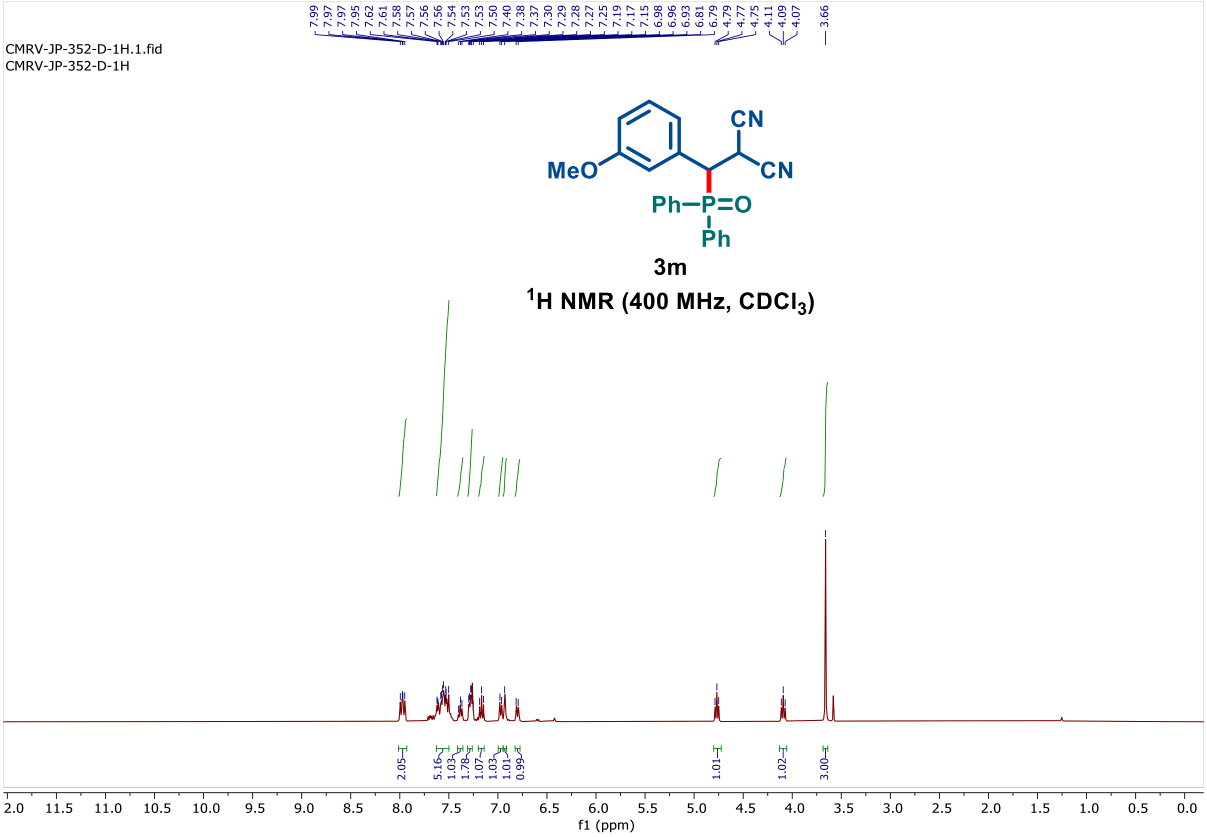

CMRV-JP-352-D-1H.1.fid
CMRV-JP-352-D-1H
7.99 7.97 7.97 7.95 7.62 7.61 7.58 7.57 7.56 7.56 7.54 7.53 7.53 7.50 7.40 7.38 7.37 7.30 7.29 7.28 7.27 7.25 7.19 7.17 7.15 6.98 6.96 6.93 6.81 6.79 4.79 4.77 4.75 4.11 4.09 4.07 3.66
MeO
CN
CN
Ph—P=O
Ph
3m
1H NMR (400 MHz, CDCl3)
2.05 5.16 1.03 1.78 1.07 1.03 1.01 0.99 1.01 1.02 3.00
2.0 11.5 11.0 10.5 10.0 9.5 9.0 8.5 8.0 7.5 7.0 6.5 6.0 5.5 5.0 4.5 4.0 3.5 3.0 2.5 2.0 1.5 1.0 0.5 0.0
f1 (ppm)

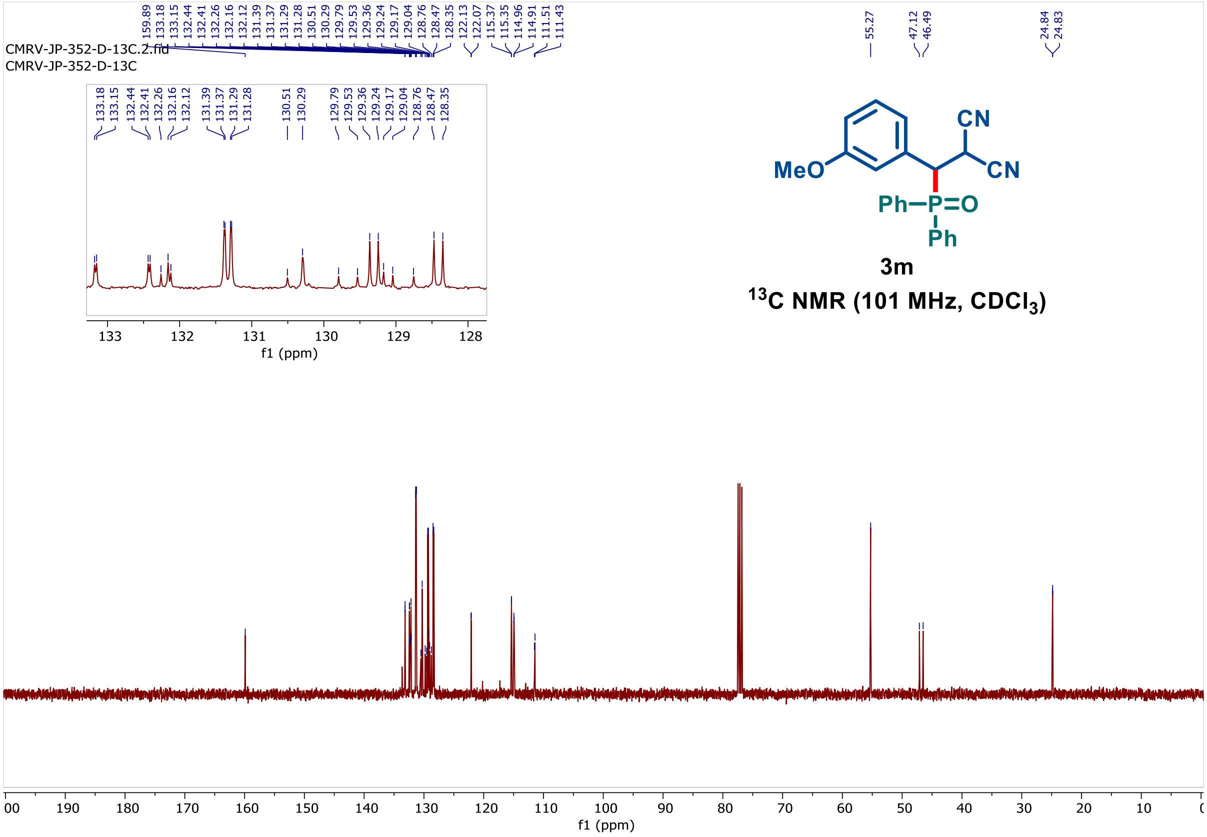
CMRV-JP-352-D-13C.2.fid
CMRV-JP-352-D-13C
159.89 133.18 133.15 132.44 132.41 132.26 132.16 132.12 131.39 131.37 131.29 131.28 130.51 130.29 129.79 129.53 129.36 129.24 129.17 129.04 128.76 128.47 128.35 122.13 122.07 115.37 115.35 114.96 114.91 111.51 111.43
55.27
47.12 46.49
24.84 24.83
MeO
CN
CN
Ph—P=O
Ph
3m
^{13}C NMR (101 MHz, $CDCl_3$)
f1 (ppm)

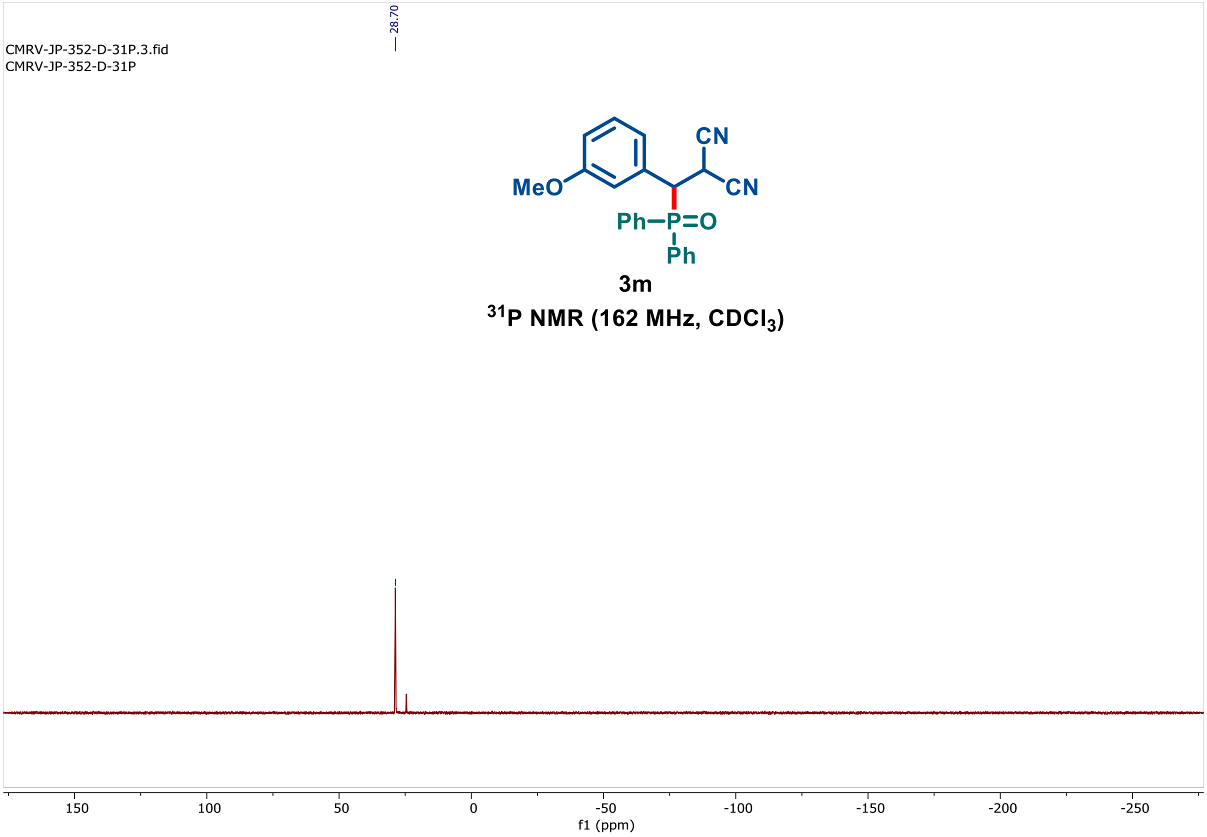
CMRV-JP-352-D-31P.3.fid
CMRV-JP-352-D-31P
28.70
MeO
CN
CN
Ph—P=O
Ph
3m
^{31}P NMR (162 MHz, $CDCl_3$)
f1 (ppm)

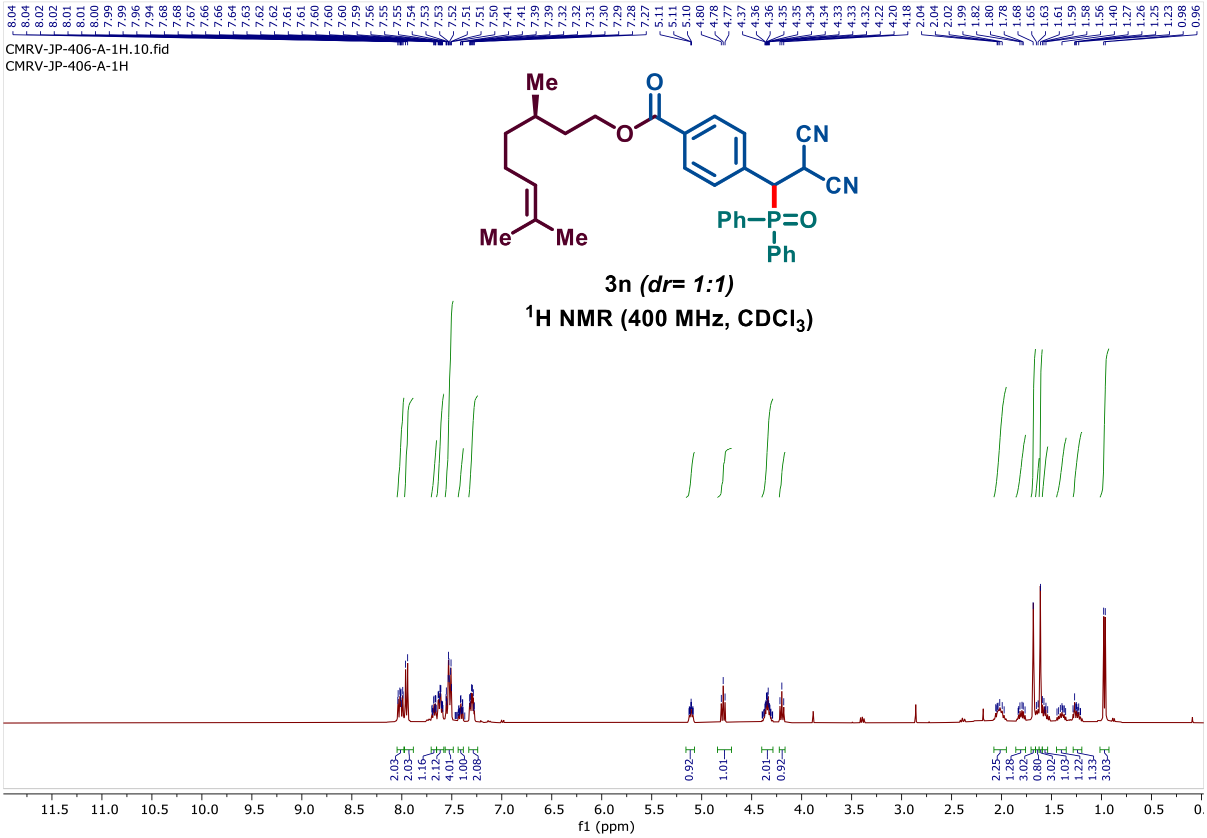
CMRV-JP-406-A-1H.10.fid
CMRV-JP-406-A-1H
Me
O
O
CN
CN
Me
Me
Ph—P=O
Ph
3n (dr= 1:1)
1H NMR (400 MHz, CDCl3)
f1 (ppm)

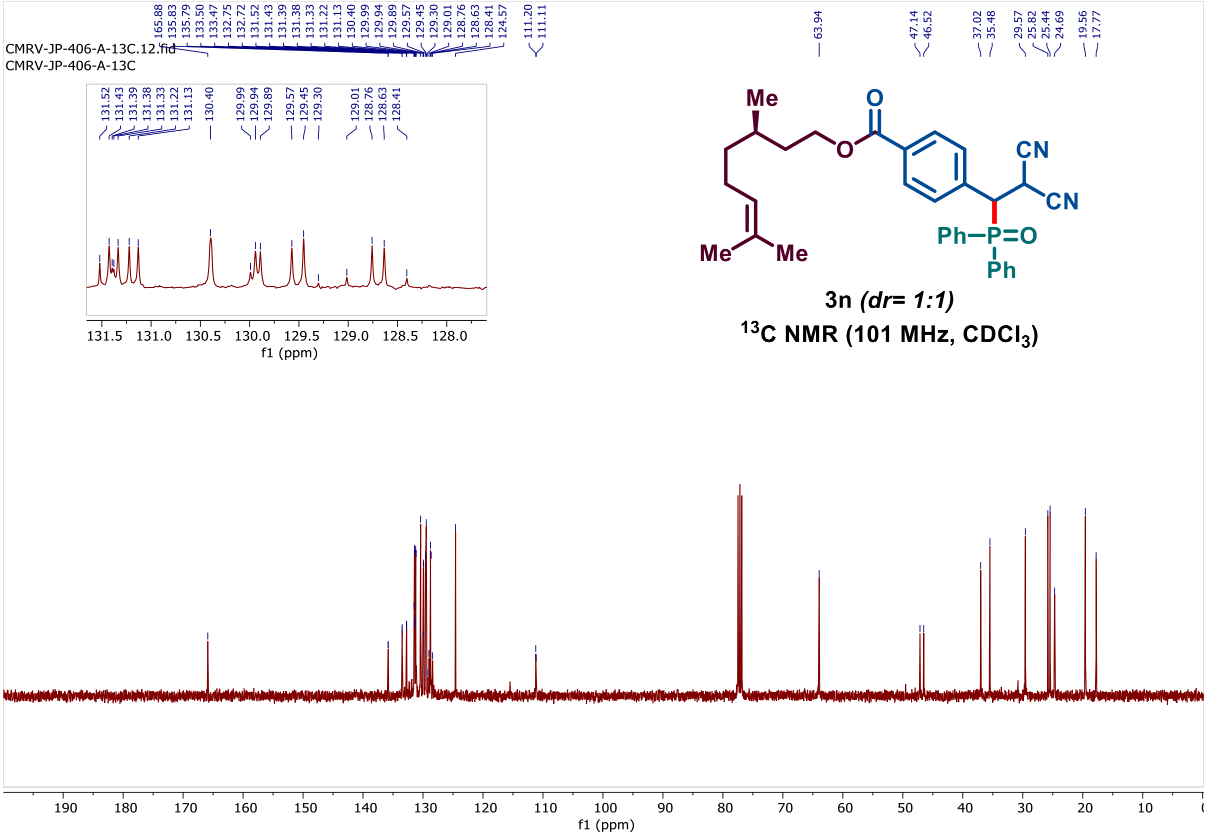
CMRV-JP-406-A-13C.12.fid
CMRV-JP-406-A-13C
Me
O
O
CN
CN
Me
Me
Ph—P=O
Ph
3n (dr= 1:1)
13C NMR (101 MHz, CDCl3)
f1 (ppm)

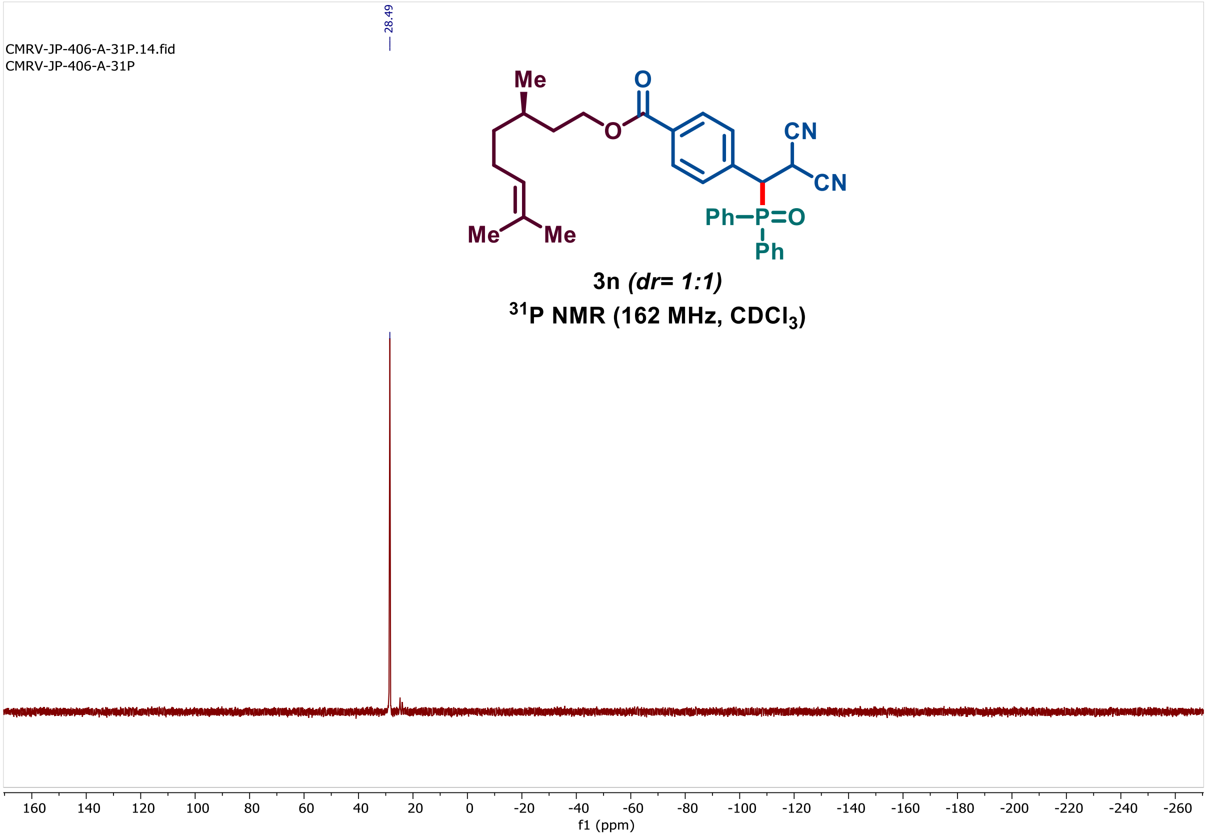
CMRV-JP-406-A-31P.14.fid
CMRV-JP-406-A-31P
28.49
Me
O
O
CN
CN
Ph—P=O
Ph
Me
Me
3n (dr= 1:1)
31P NMR (162 MHz, CDCl3)
f1 (ppm)

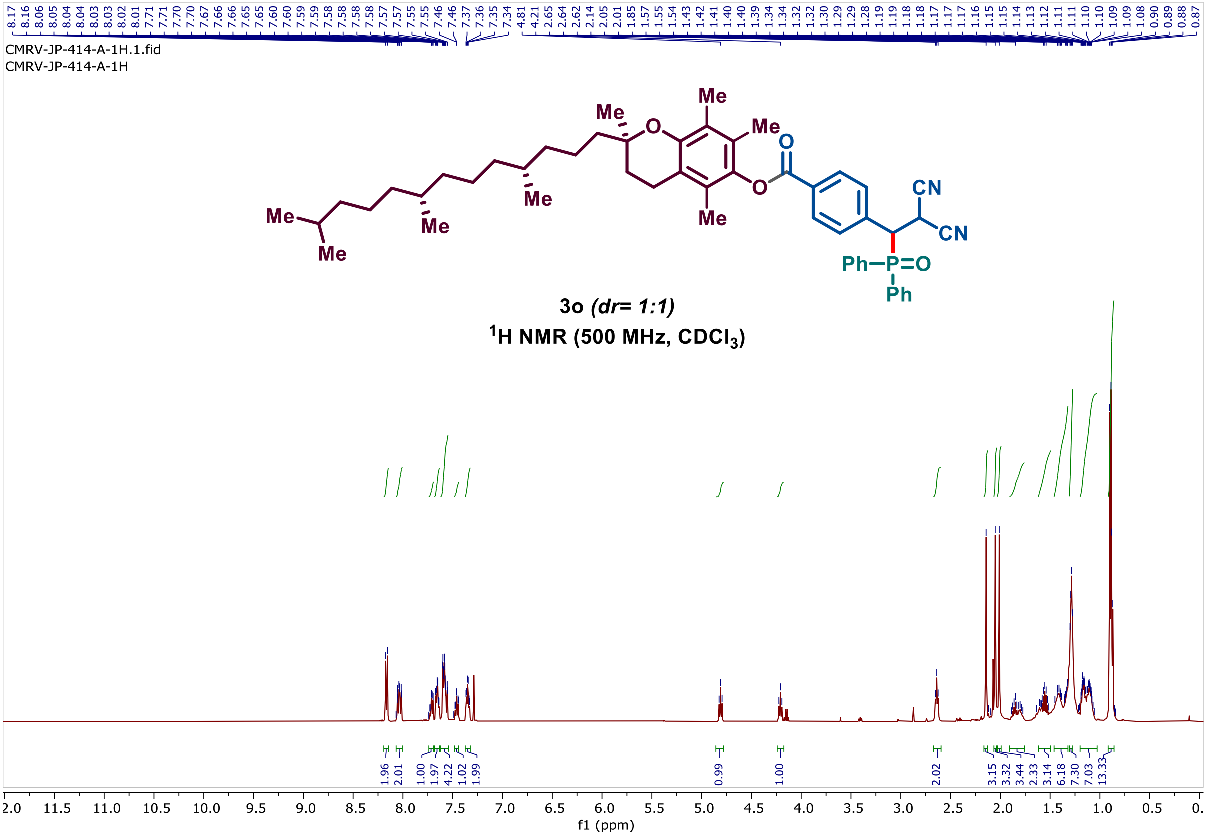
CMRV-JP-414-A-1H.1.fid
CMRV-JP-414-A-1H
Me
O
Me
Me
O
O
CN
CN
Me
Me
Me
Me
Me
Ph—P=O
Ph
3o (dr= 1:1)
1H NMR (500 MHz, CDCl3)
f1 (ppm)

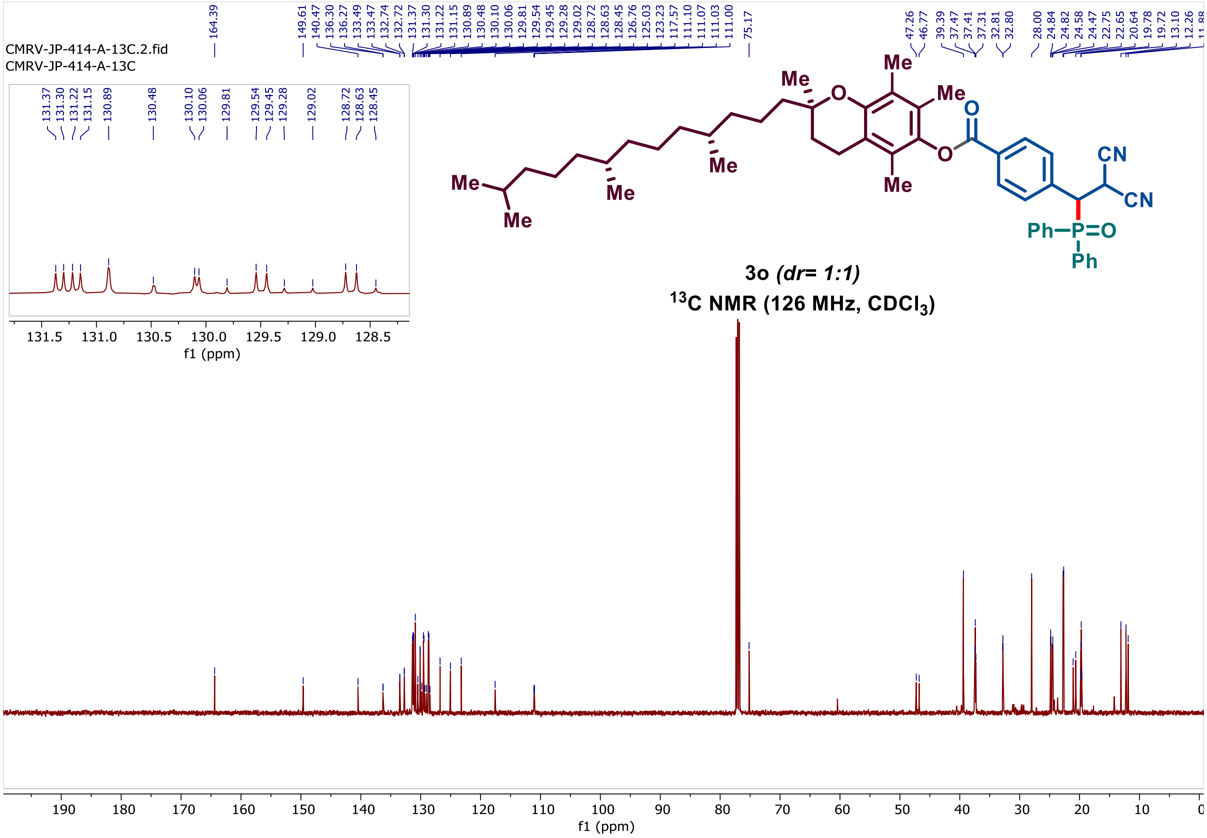
CMRV-JP-414-A-13C.2.fid
CMRV-JP-414-A-13C
164.39
149.61 140.47 136.30 136.27 133.49 133.47 132.74 132.72 131.37 131.30 131.22 131.15 130.89 130.48 130.10 130.06 129.81 129.54 129.45 129.28 129.02 128.72 128.63 128.45 126.76 125.03 123.23 117.57 111.10 111.07 111.03 111.00
75.17
47.26 46.77 39.39 37.47 37.41 37.31 32.81 32.80 28.00 24.84 24.82 24.58 24.47 22.75 22.65 20.64 19.78 19.72 13.10 12.26
131.37 131.30 131.22 131.15 130.89 130.48 130.10 130.06 129.81 129.54 129.45 129.28 129.02 128.72 128.63 128.45
f1 (ppm)
3o (dr= 1:1)
13C NMR (126 MHz, CDCl3)
f1 (ppm)

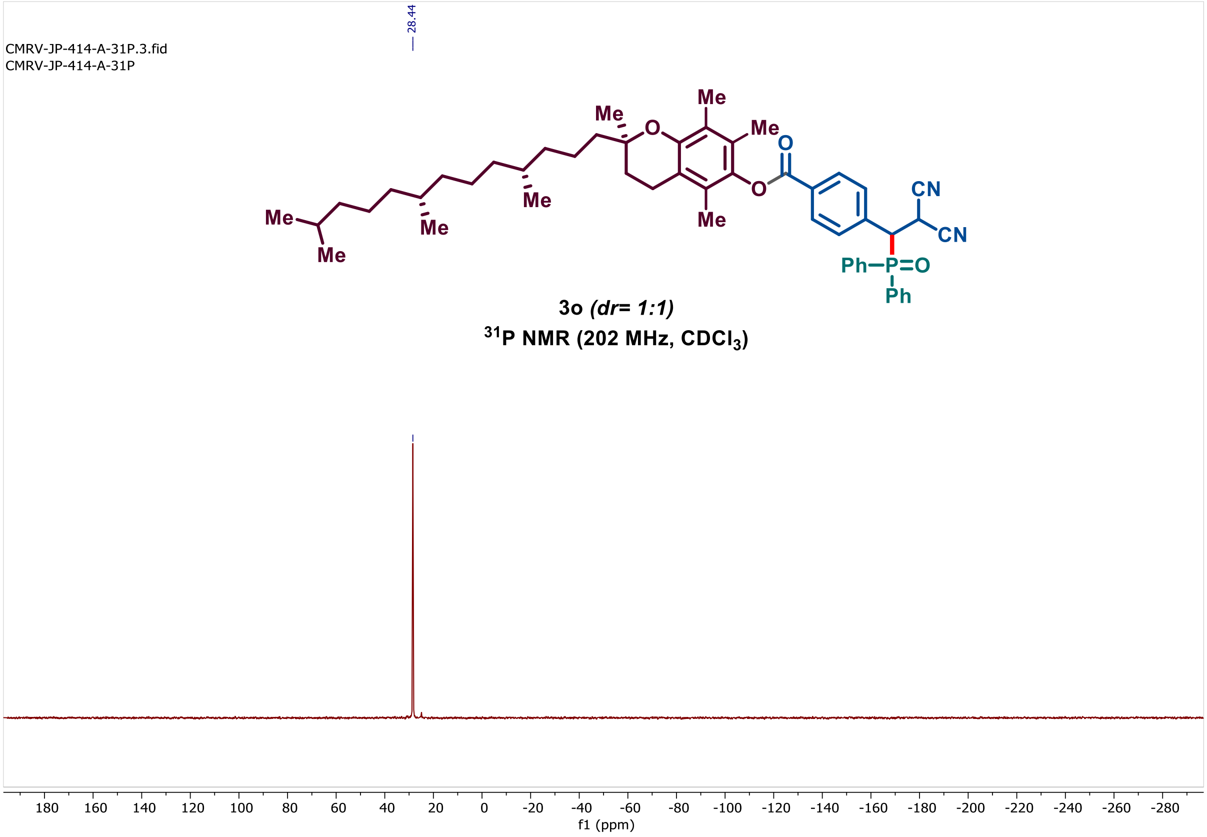
CMRV-JP-414-A-31P.3.fid
CMRV-JP-414-A-31P
28.44
3o (dr= 1:1)
31P NMR (202 MHz, CDCl3)
f1 (ppm)

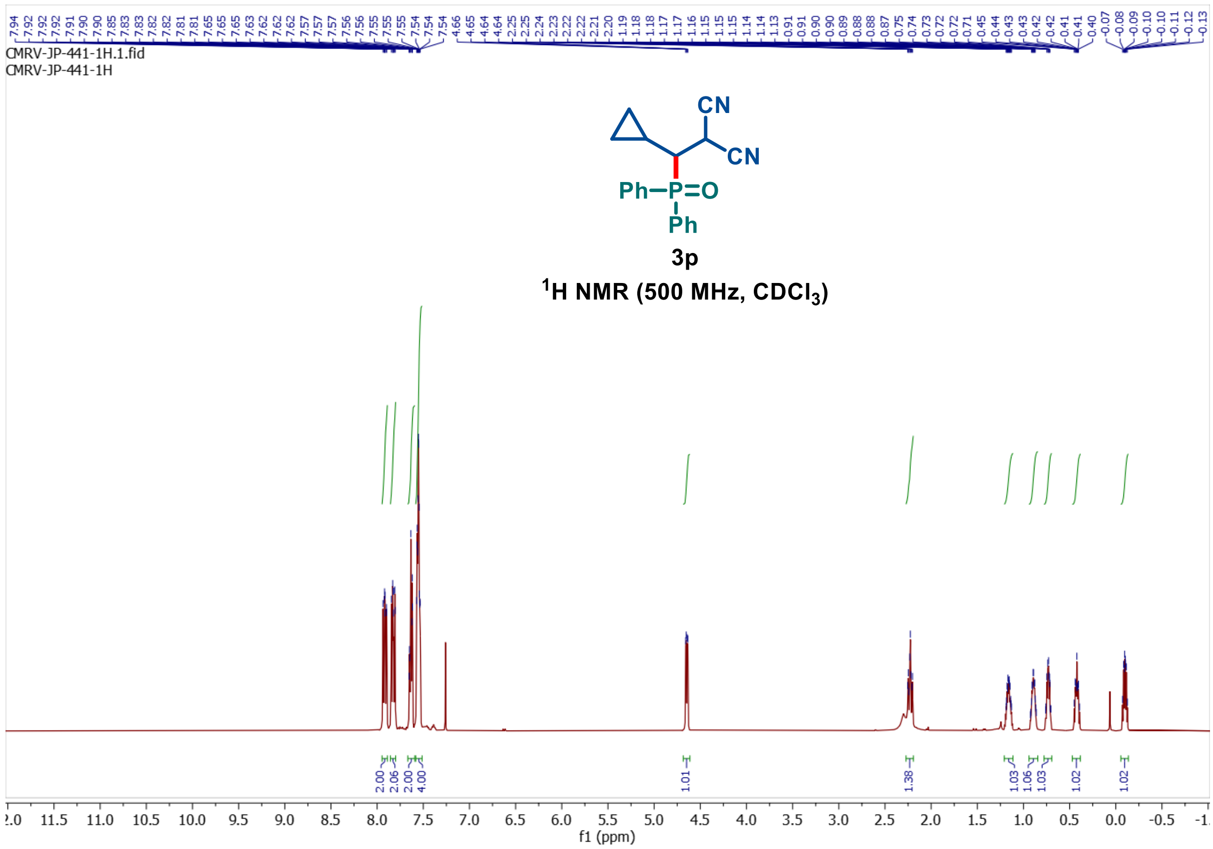
CMRV-JP-441-1H.1.fid
CMRV-JP-441-1H
CN
CN
Ph
P=O
Ph
3p
¹H NMR (500 MHz, CDCl₃)
f1 (ppm)

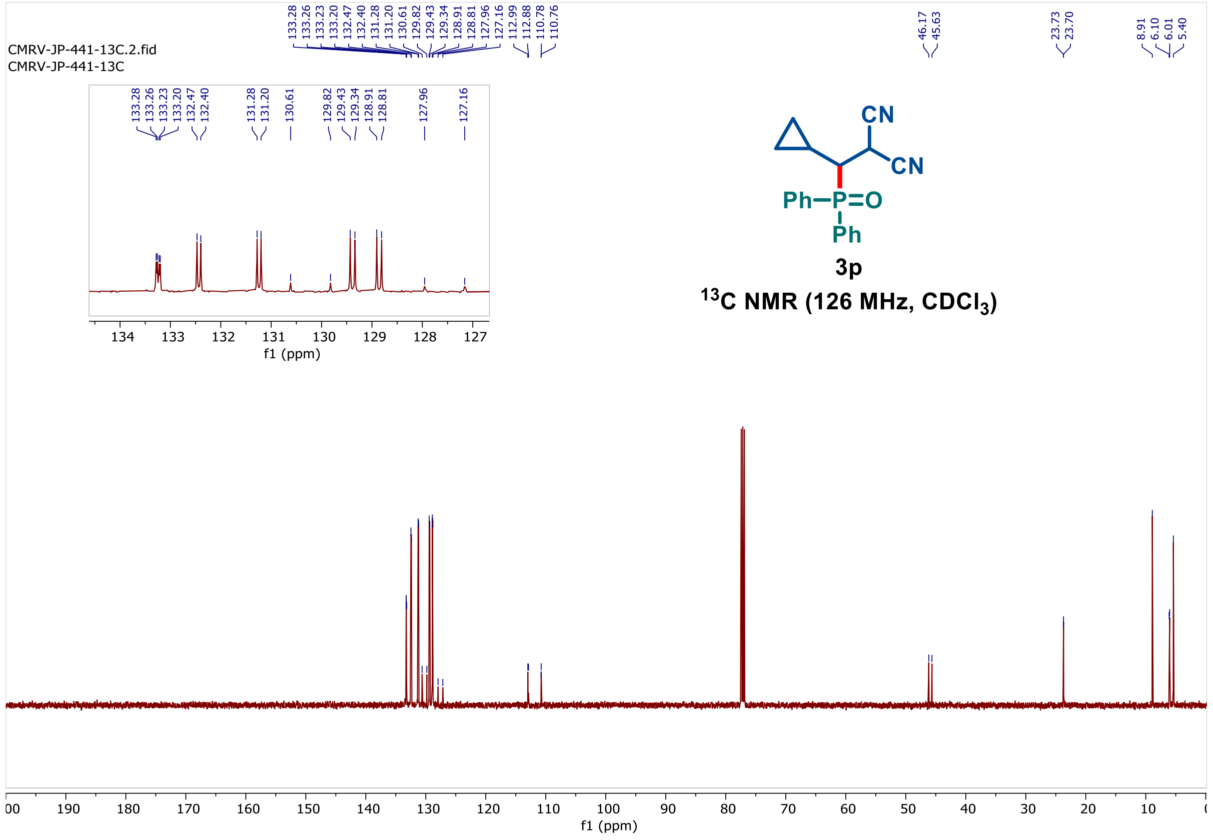
CMRV-JP-441-13C.2.fid
CMRV-JP-441-13C
CN
CN
Ph
P=O
Ph
3p
¹³C NMR (126 MHz, CDCl₃)
f1 (ppm)

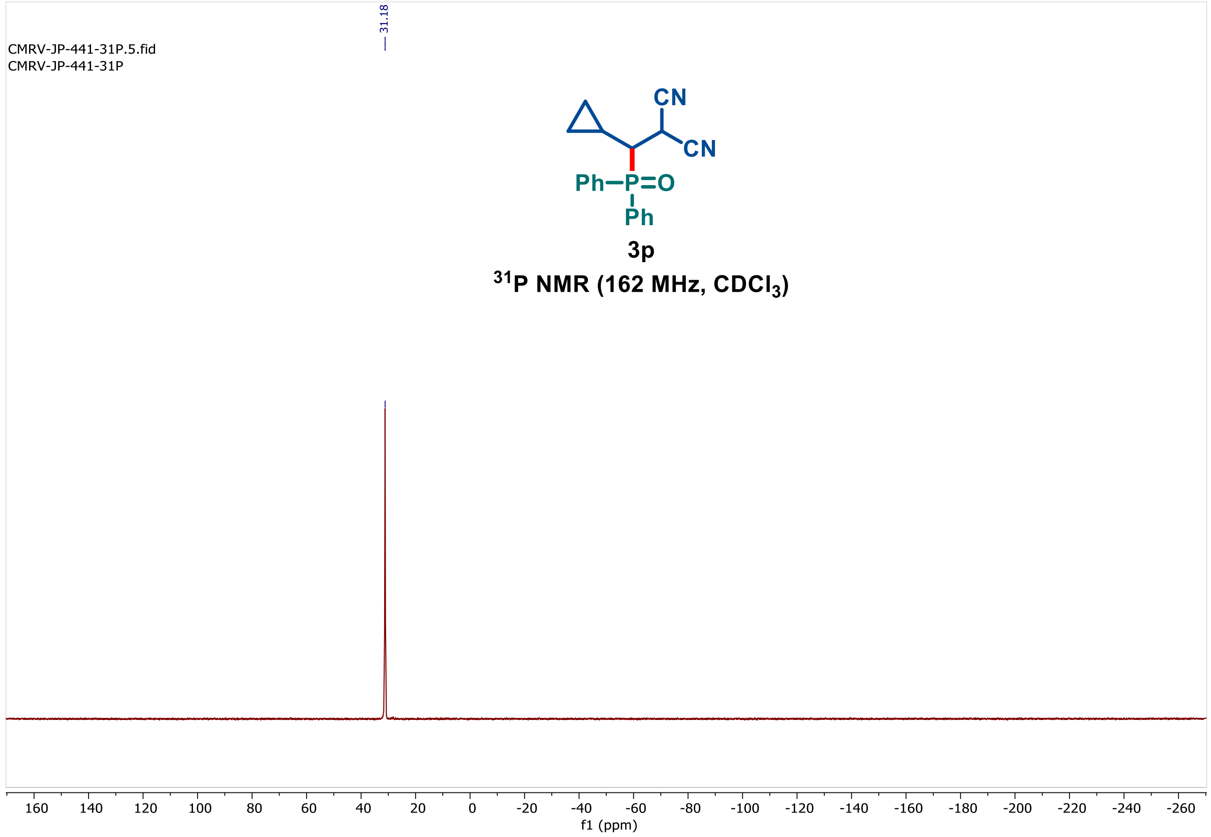

31.18
CMRV-JP-441-31P.5.fid
CMRV-JP-441-31P
CN
CN
Ph–P=O
Ph
3p
^{31}P NMR (162 MHz, CDCl$_3$)
160 140 120 100 80 60 40 20 0 -20 -40 -60 -80 -100 -120 -140 -160 -180 -200 -220 -240 -260
f1 (ppm)

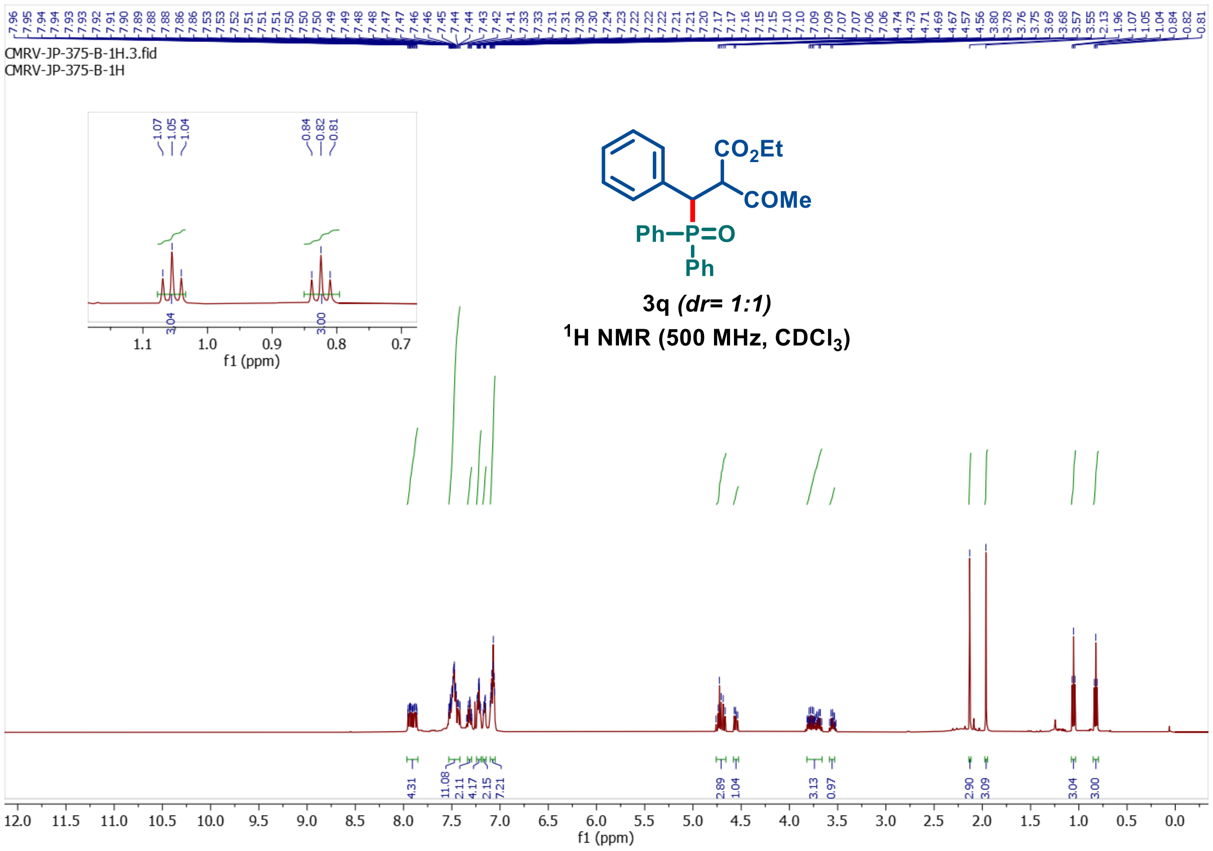

7.96 7.95 7.94 7.94 7.93 7.93 7.92 7.91 7.90 7.89 7.88 7.88 7.86 7.86 7.53 7.53 7.52 7.51 7.51 7.51 7.50 7.50 7.49 7.49 7.48 7.48 7.47 7.47 7.46 7.46 7.45 7.44 7.44 7.43 7.42 7.41 7.33 7.33 7.31 7.31 7.30 7.30 7.24 7.23 7.22 7.22 7.22 7.21 7.21 7.20 7.17 7.17 7.16 7.15 7.15 7.10 7.10 7.09 7.09 7.07 7.07 7.06 7.06 4.74 4.73 4.71 4.69 4.67 4.57 4.56 3.80 3.78 3.76 3.75 3.69 3.68 3.57 3.55 2.13 1.96 1.07 1.05 1.04 0.84 0.82 0.81
CMRV-JP-375-B-1H.3.fid
CMRV-JP-375-B-1H
1.07 1.05 1.04
0.84 0.82 0.81
3.04
3.00
1.1 1.0 0.9 0.8 0.7
f1 (ppm)
CO$_2$Et
COMe
Ph–P=O
Ph
3q (dr= 1:1)
^{1}H NMR (500 MHz, CDCl$_3$)
4.31 11.08 2.11 4.17 2.15 7.21 2.89 1.04 3.13 0.97 2.90 3.09 3.04 3.00
12.0 11.5 11.0 10.5 10.0 9.5 9.0 8.5 8.0 7.5 7.0 6.5 6.0 5.5 5.0 4.5 4.0 3.5 3.0 2.5 2.0 1.5 1.0 0.5 0.0
f1 (ppm)

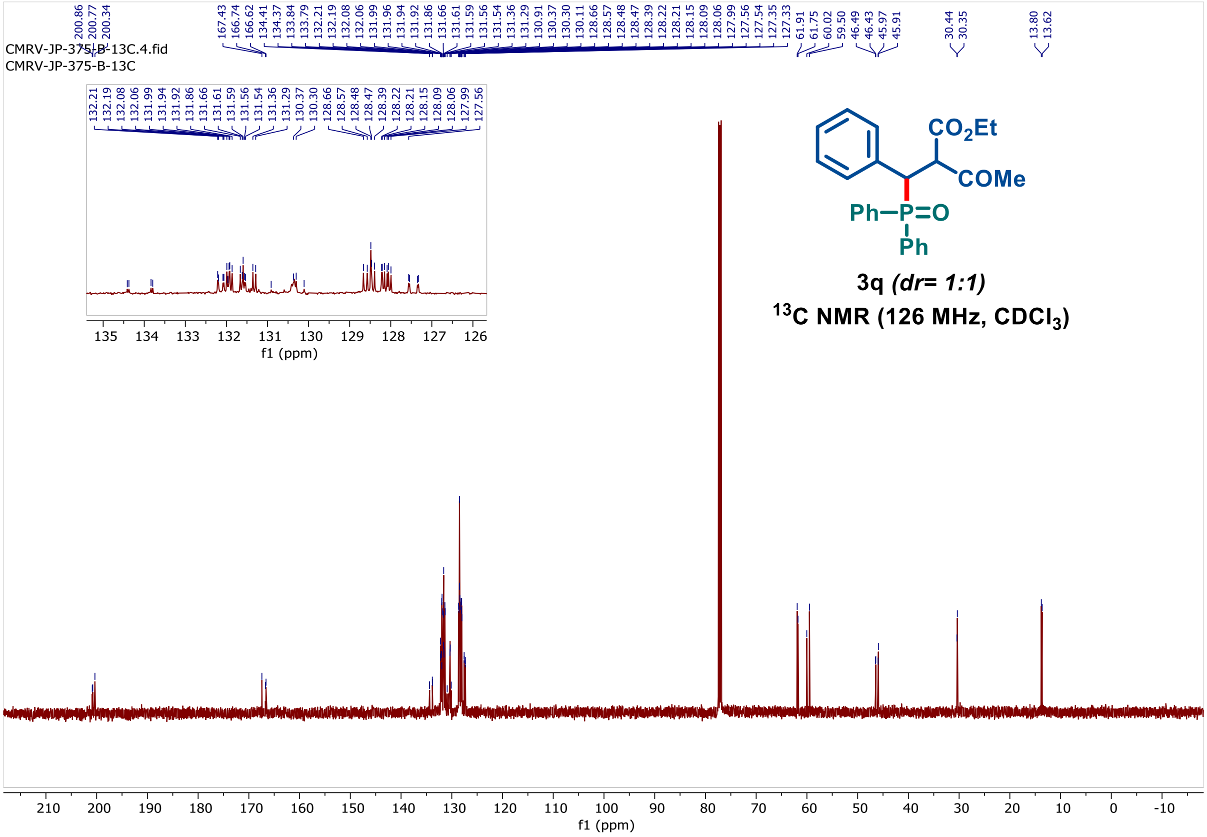
CMRV-JP-375-B-13C.4.fid
CMRV-JP-375-B-13C
200.86 200.77 200.34
167.43 166.74 166.62 134.41 134.37 133.84 133.79 132.21 132.19 132.08 132.06 131.99 131.96 131.94 131.92 131.86 131.66 131.61 131.59 131.56 131.54 131.36 131.29 130.91 130.37 130.30 130.11 128.66 128.57 128.48 128.47 128.39 128.22 128.21 128.15 128.09 128.06 127.99 127.56 127.54 127.35 127.33
61.91 61.75 60.02 59.50 46.49 46.43 45.97 45.91
30.44 30.35
13.80 13.62
CO2Et
COMe
Ph–P=O
Ph
3q (dr= 1:1)
13C NMR (126 MHz, CDCl3)
f1 (ppm)

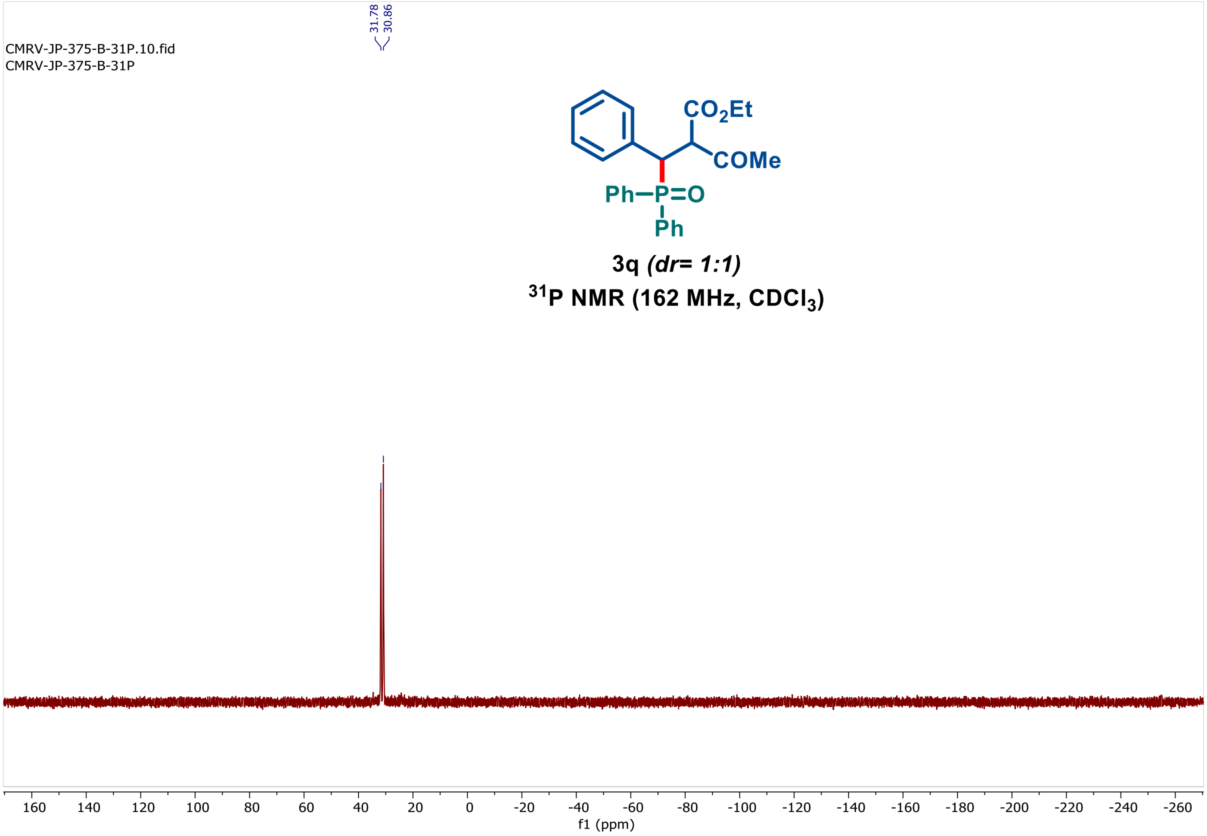
CMRV-JP-375-B-31P.10.fid
CMRV-JP-375-B-31P
31.78 30.86
CO2Et
COMe
Ph–P=O
Ph
3q (dr= 1:1)
31P NMR (162 MHz, CDCl3)
f1 (ppm)

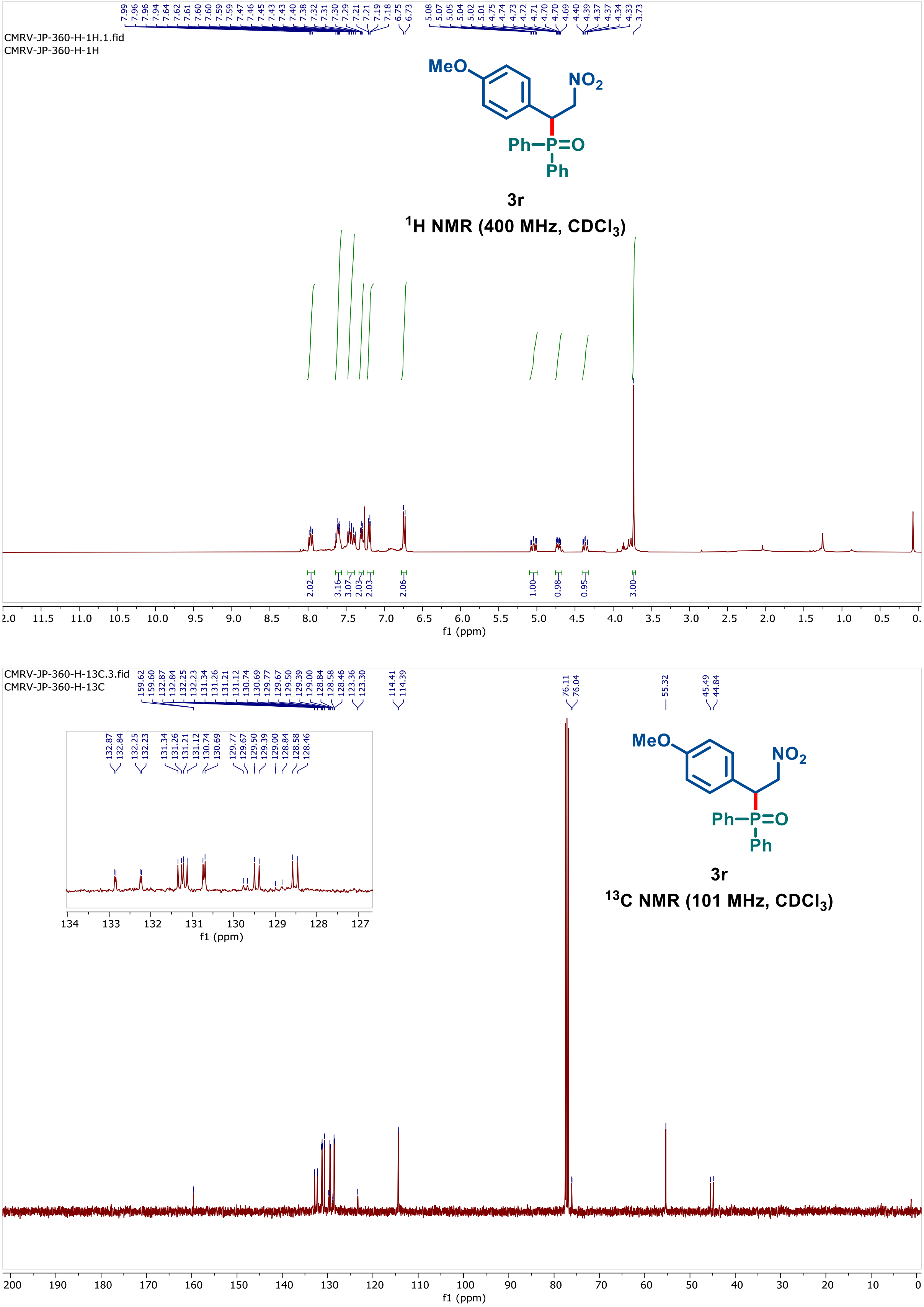
CMRV-JP-360-H-1H.1.fid
CMRV-JP-360-H-1H
MeO
NO2
Ph—P=O
Ph
3r
1H NMR (400 MHz, CDCl3)
f1 (ppm)
CMRV-JP-360-H-13C.3.fid
CMRV-JP-360-H-13C
13C NMR (101 MHz, CDCl3)

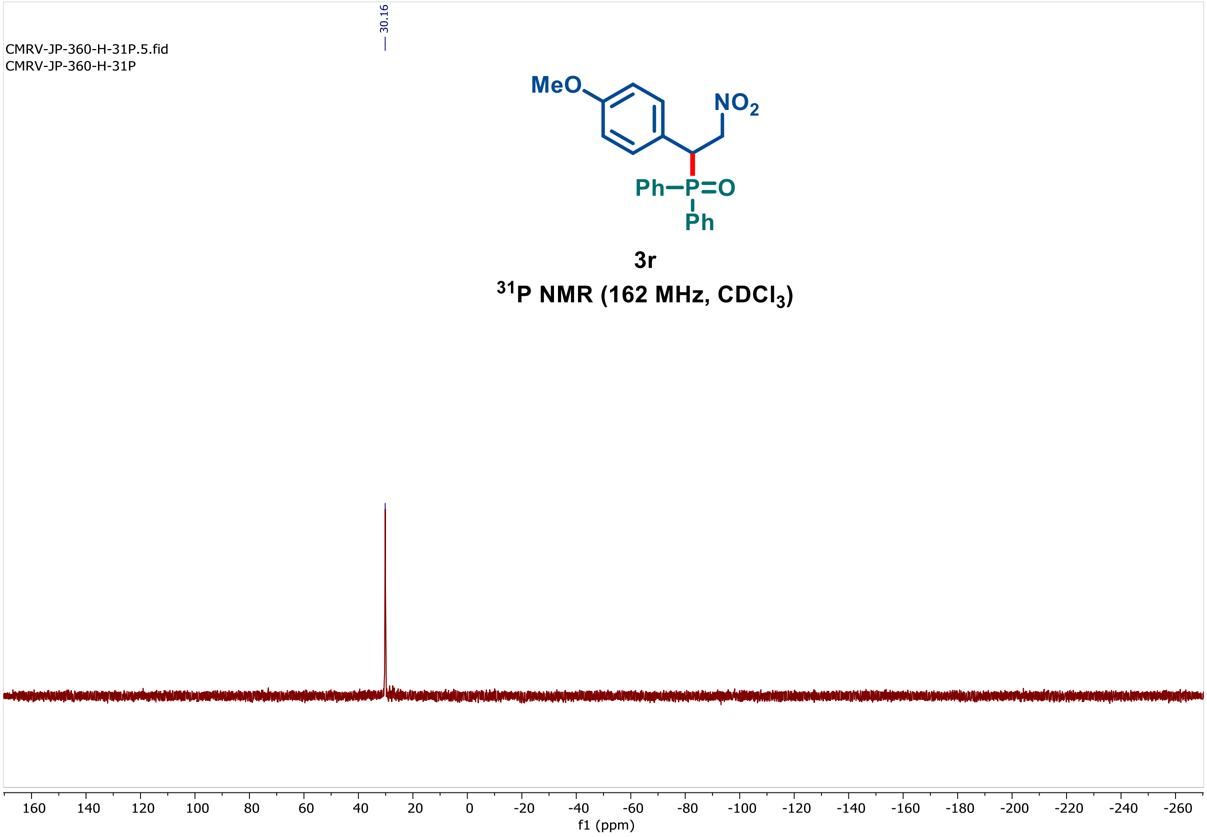
30.16
CMRV-JP-360-H-31P.5.fid
CMRV-JP-360-H-31P
MeO
NO2
Ph—P=O
Ph
3r
31P NMR (162 MHz, CDCl3)
f1 (ppm)

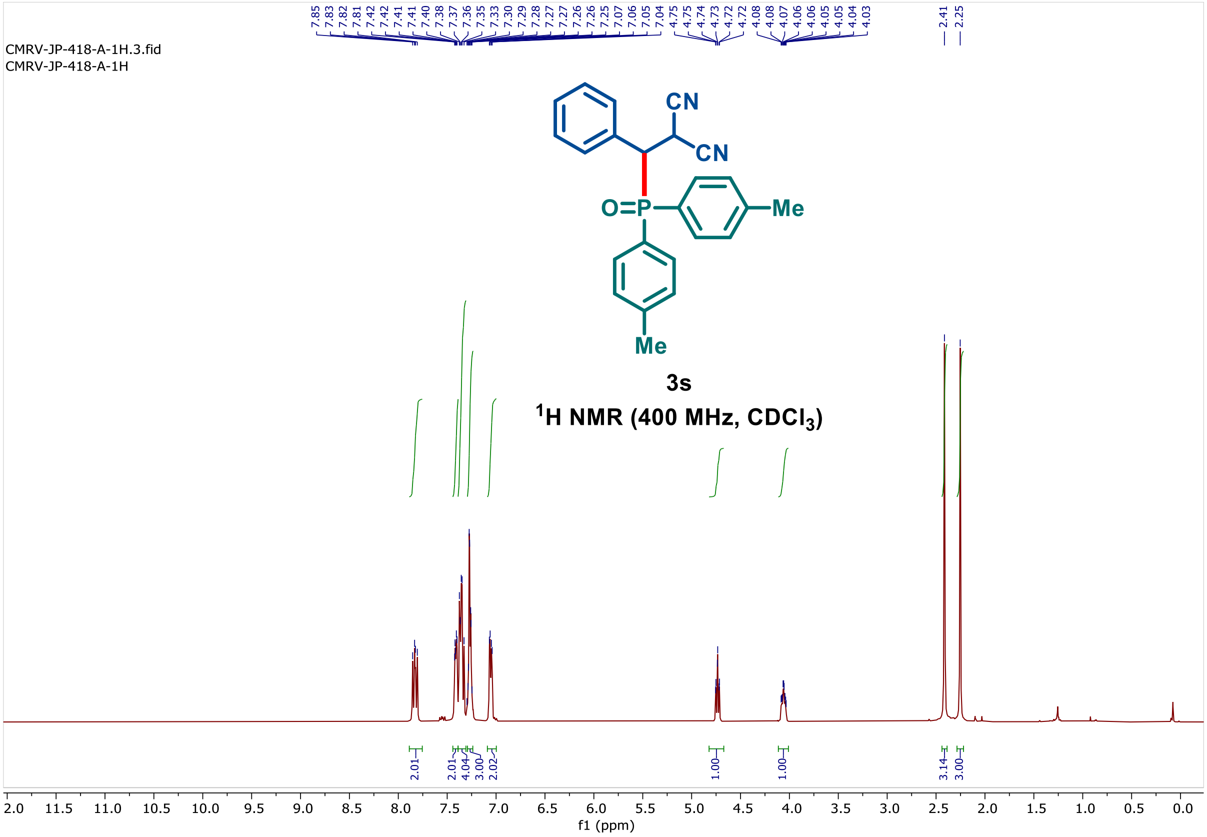
CMRV-JP-418-A-1H.3.fid
CMRV-JP-418-A-1H
CN
CN
O=P
Me
Me
3s
1H NMR (400 MHz, CDCl3)
2.41
2.25
2.01
2.01
4.04
3.00
2.02
1.00
1.00
3.14
3.00
f1 (ppm)

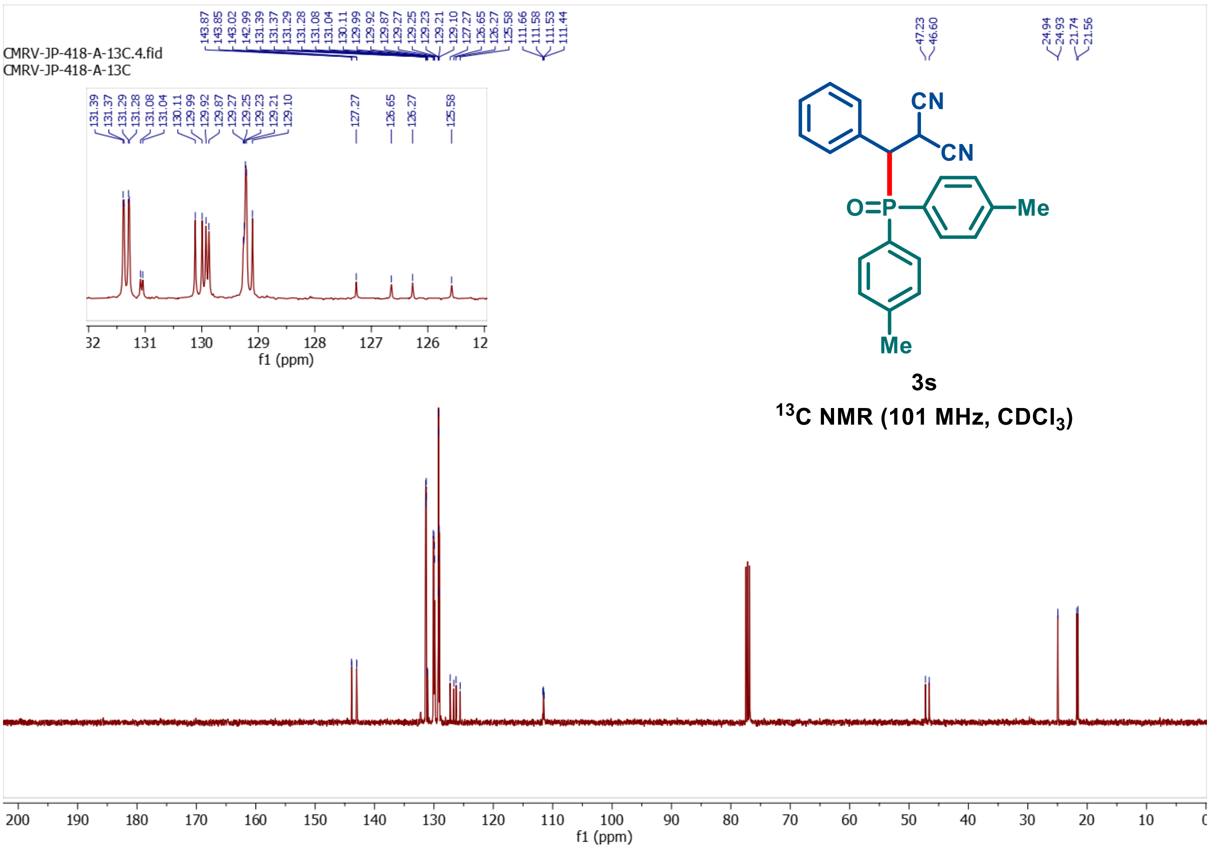
CMRV-JP-418-A-13C.4.fid
CMRV-JP-418-A-13C
143.87
143.85
143.02
142.99
131.39
131.37
131.29
131.28
131.08
131.04
130.11
129.99
129.92
129.87
129.27
129.25
129.23
129.21
129.10
127.27
126.65
126.27
125.58
111.66
111.58
111.53
111.44
47.23
46.60
24.94
24.93
21.74
21.56
CN
CN
O=P
Me
Me
3s
13C NMR (101 MHz, CDCl3)
f1 (ppm)

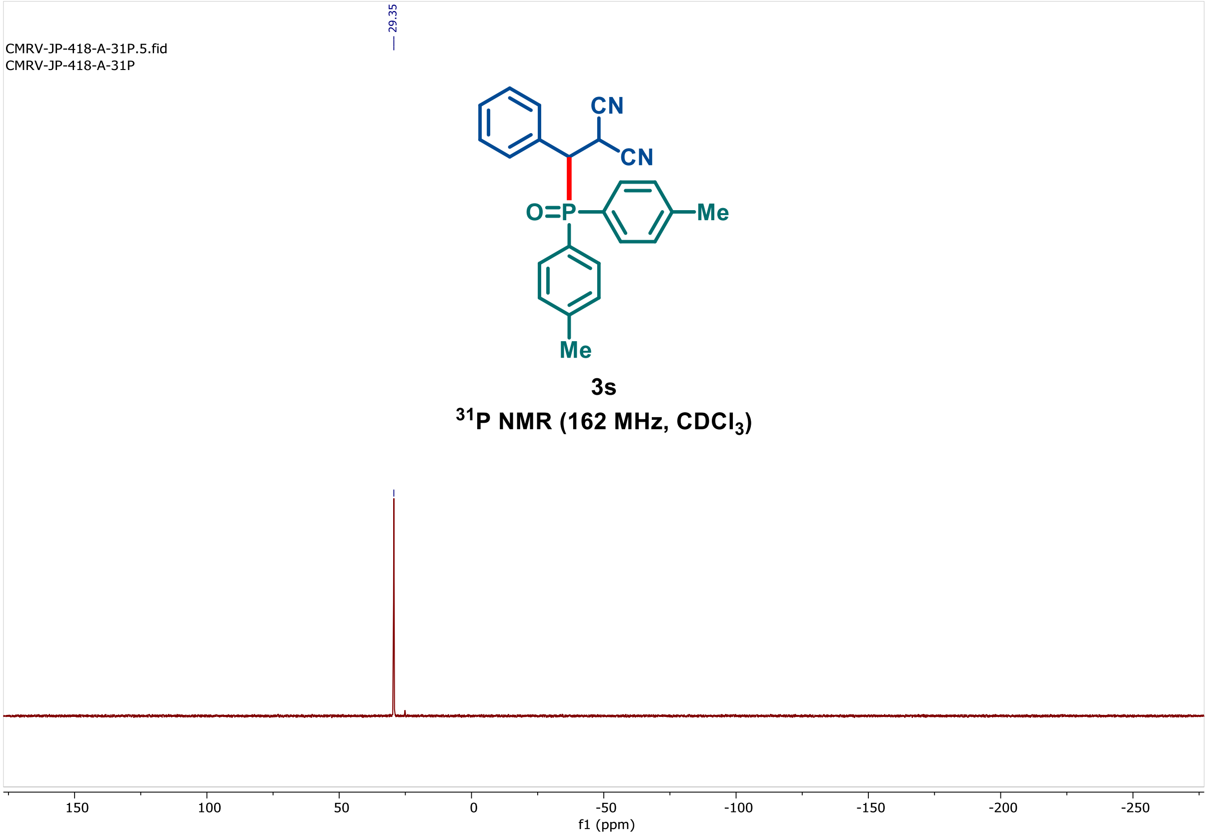
CMRV-JP-418-A-31P.5.fid
CMRV-JP-418-A-31P
29.35
CN
CN
O=P
Me
Me
3s
31P NMR (162 MHz, CDCl3)
f1 (ppm)

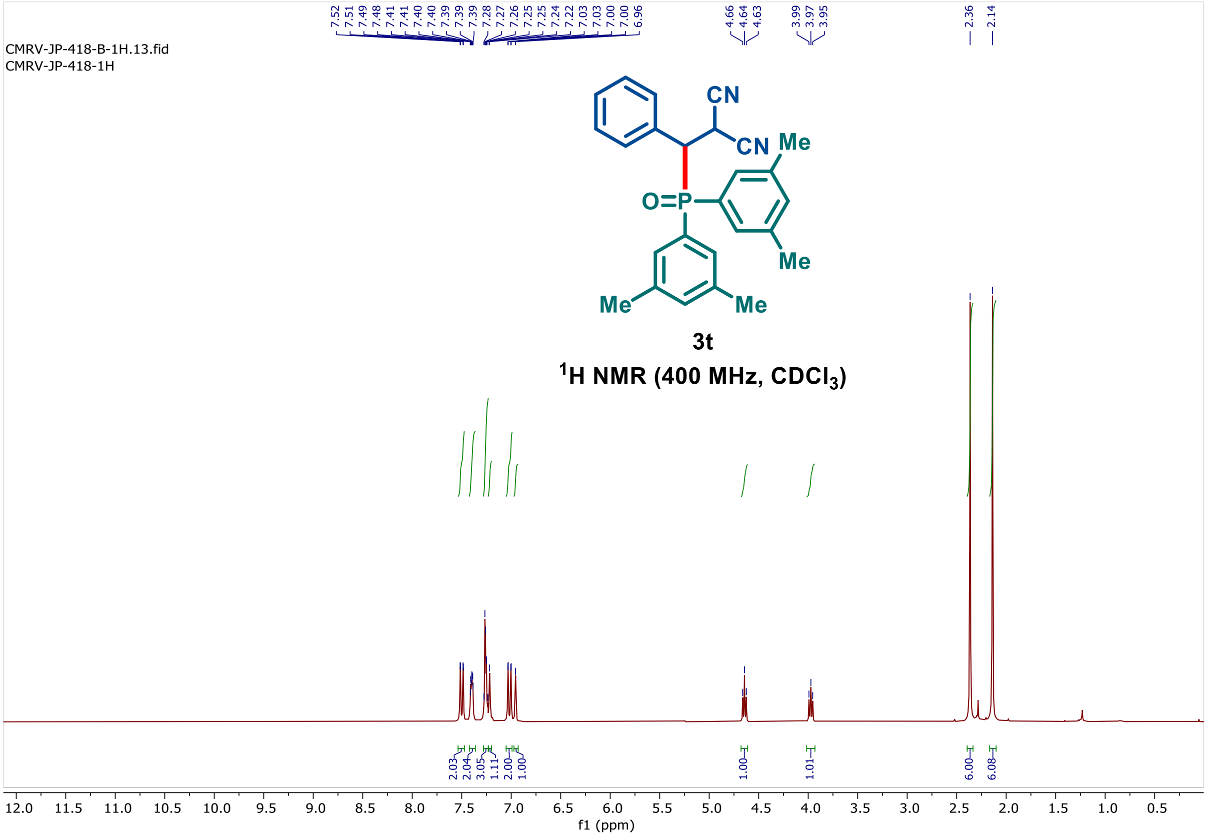
CMRV-JP-418-B-1H.13.fid
CMRV-JP-418-1H
3t
1H NMR (400 MHz, CDCl3)
f1 (ppm)

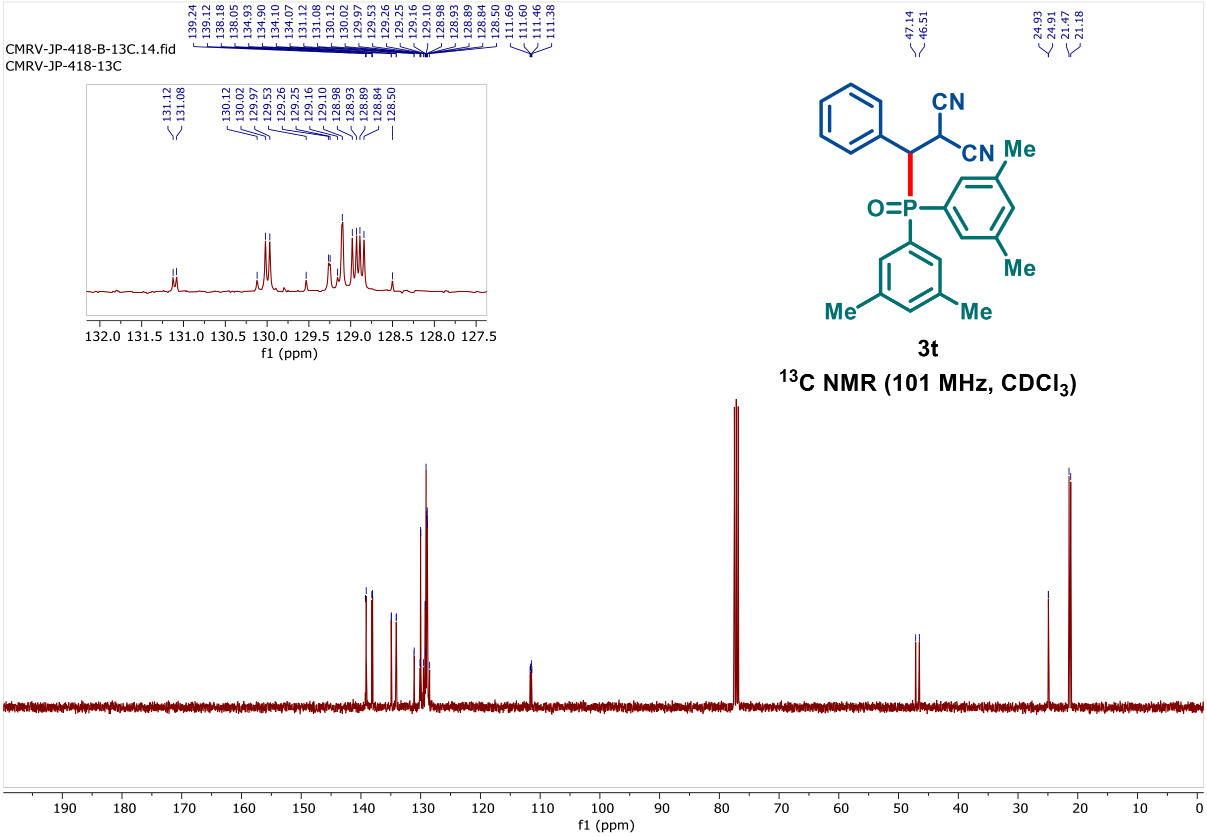
CMRV-JP-418-B-13C.14.fid
CMRV-JP-418-13C
3t
13C NMR (101 MHz, CDCl3)
f1 (ppm)

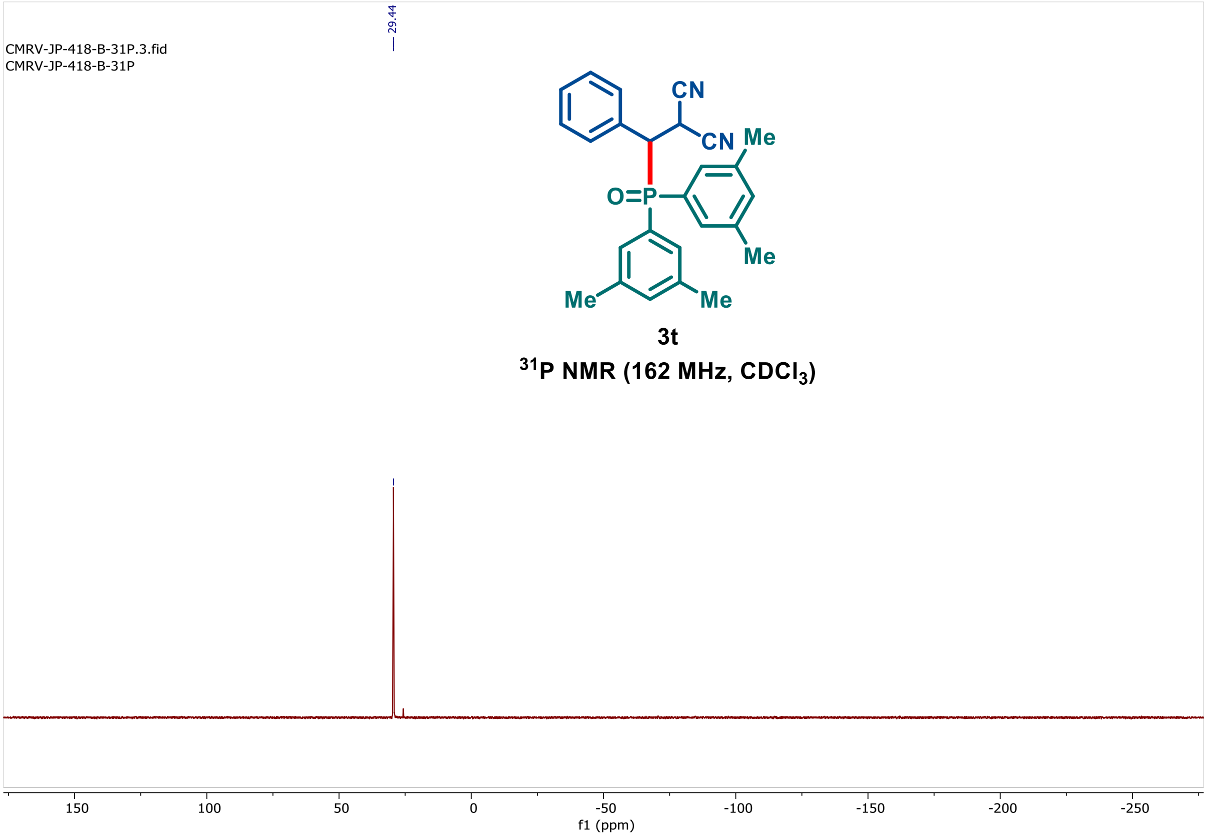
CMRV-JP-418-B-31P.3.fid
CMRV-JP-418-B-31P
29.44
CN
CN
Me
O=P
Me
Me
Me
3t
^{31}P NMR (162 MHz, CDCl$_3$)
f1 (ppm)

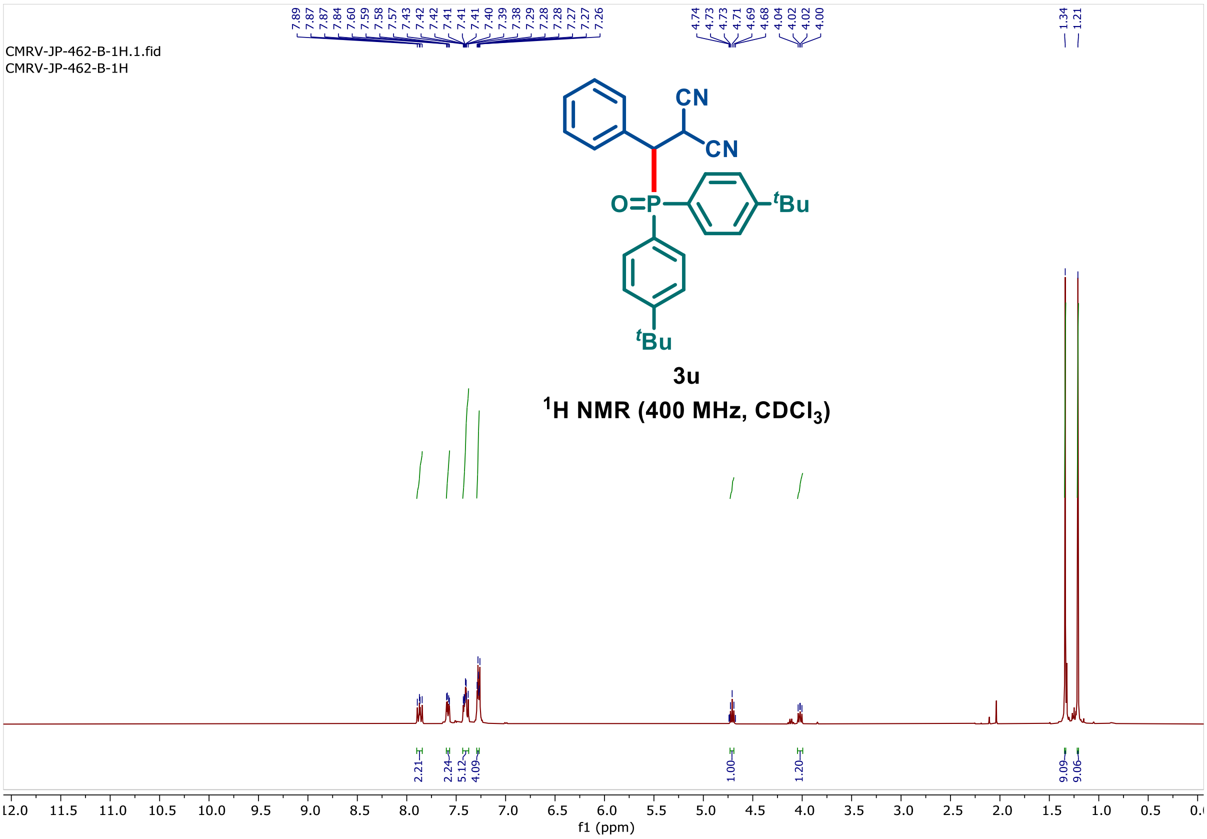
CMRV-JP-462-B-1H.1.fid
CMRV-JP-462-B-1H
CN
CN
O=P
^{t}Bu
^{t}Bu
3u
^{1}H NMR (400 MHz, CDCl$_3$)
f1 (ppm)

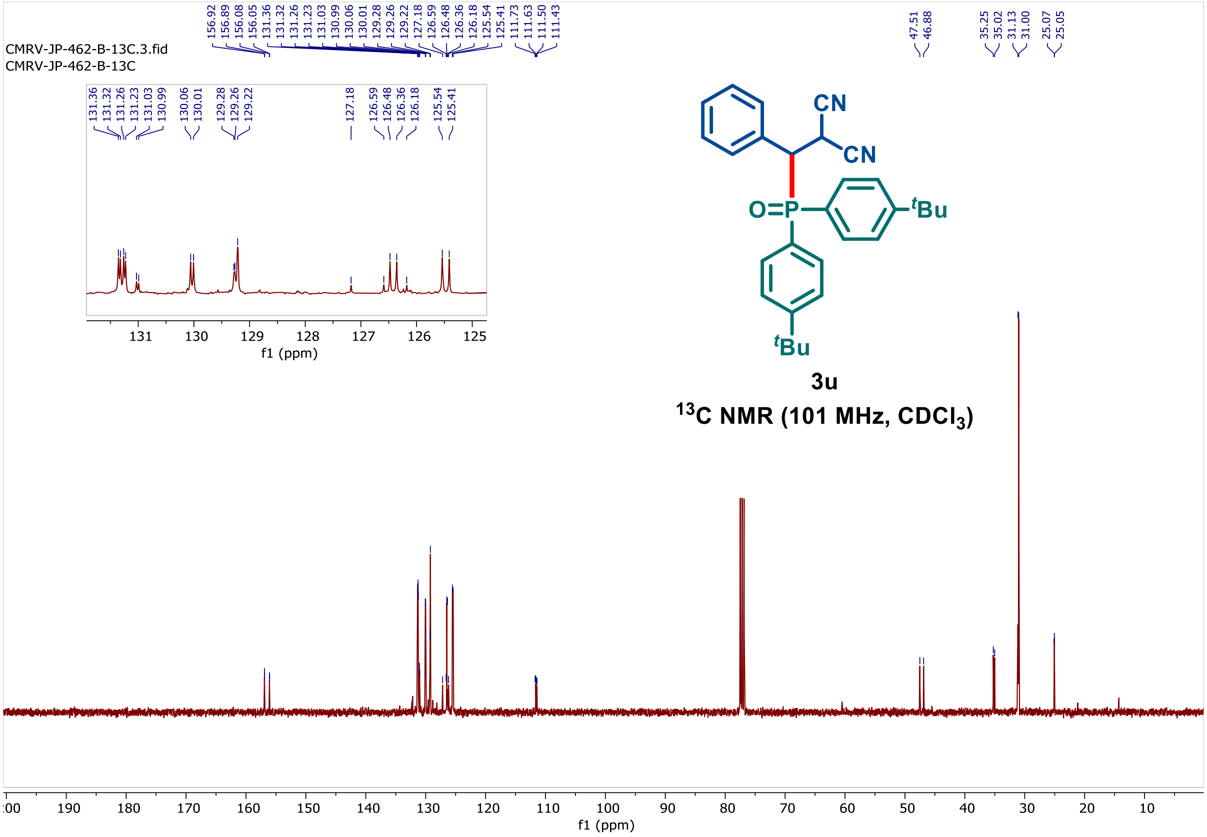
CMRV-JP-462-B-13C.3.fid
CMRV-JP-462-B-13C
3u
^{13}C NMR (101 MHz, CDCl$_3$)
f1 (ppm)

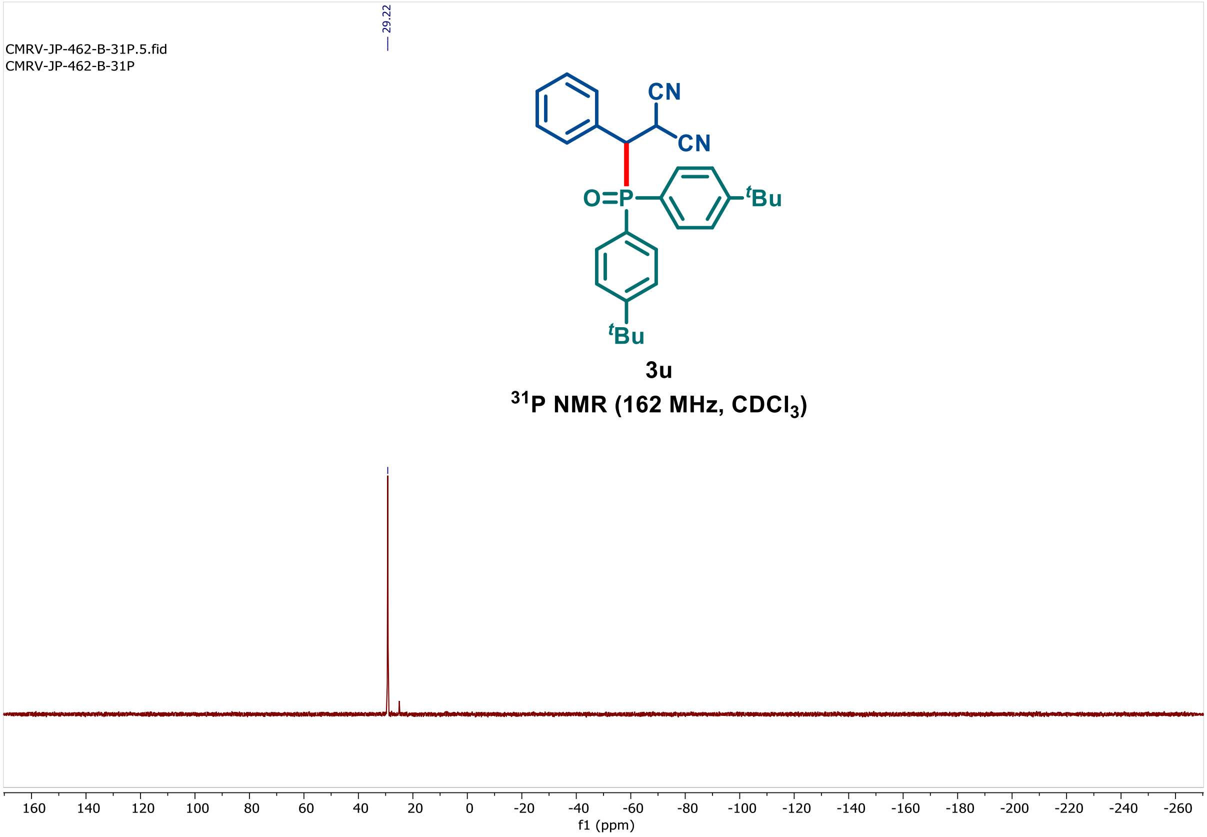
CMRV-JP-462-B-31P.5.fid
CMRV-JP-462-B-31P
29.22
3u
^{31}P NMR (162 MHz, CDCl$_3$)
f1 (ppm)

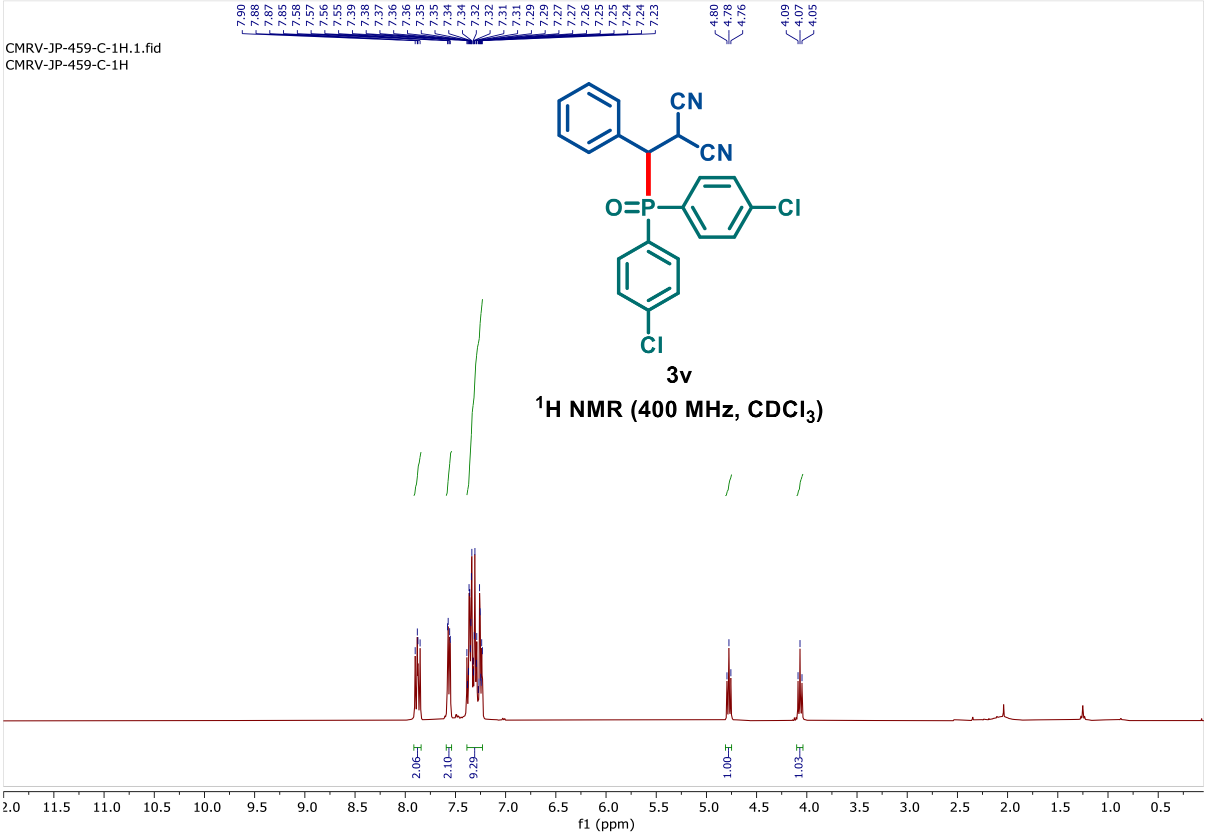
CMRV-JP-459-C-1H.1.fid
CMRV-JP-459-C-1H
7.90 7.88 7.87 7.85 7.58 7.57 7.56 7.55 7.39 7.38 7.37 7.36 7.36 7.35 7.35 7.34 7.34 7.32 7.32 7.31 7.31 7.29 7.29 7.27 7.27 7.26 7.25 7.25 7.24 7.24 7.23
4.80 4.78 4.76
4.09 4.07 4.05
CN
CN
O=P
Cl
Cl
3v
¹H NMR (400 MHz, CDCl₃)
2.06
2.10
9.29
1.00
1.03
f1 (ppm)

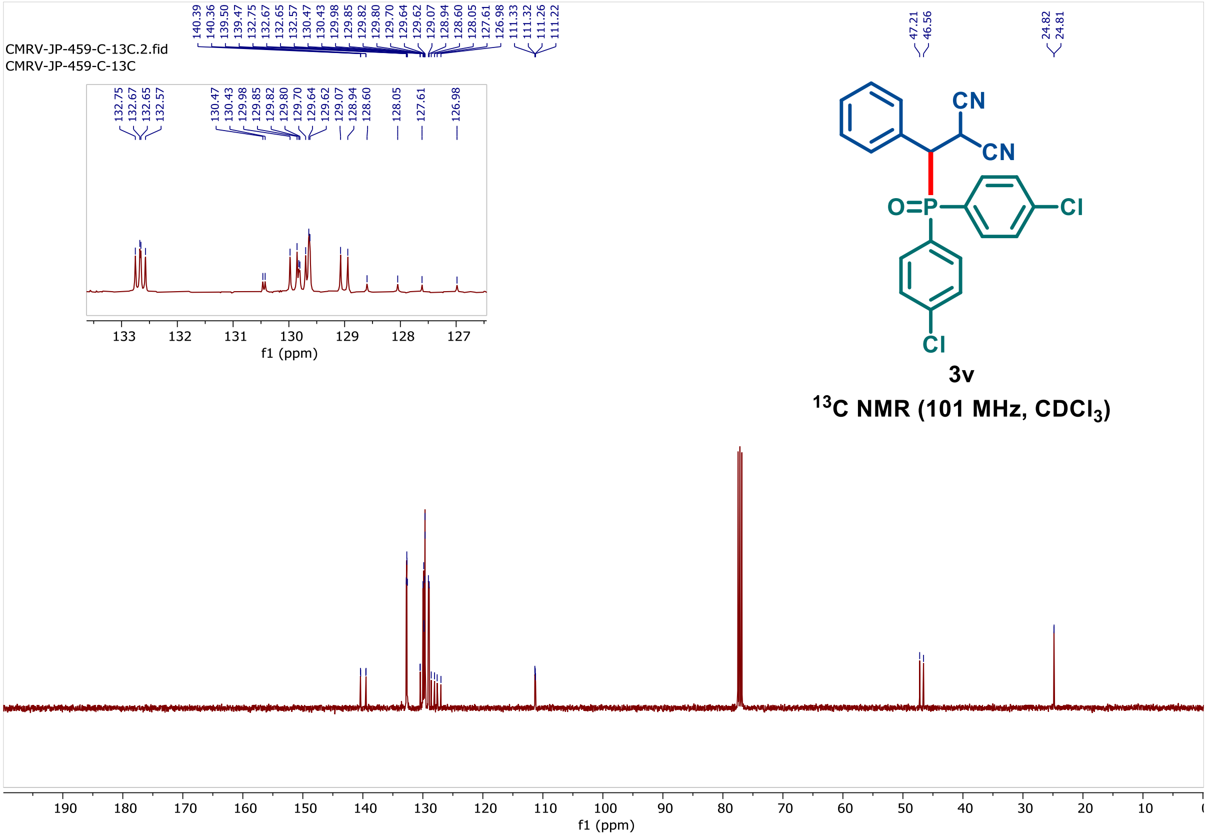
CMRV-JP-459-C-13C.2.fid
CMRV-JP-459-C-13C
140.39 140.36 139.50 139.47 132.75 132.67 132.65 132.57 130.47 130.43 129.98 129.85 129.82 129.80 129.70 129.64 129.62 129.07 128.94 128.60 128.05 127.61 126.98 111.33 111.32 111.26 111.22
47.21 46.56
24.82 24.81
f1 (ppm)
CN
CN
O=P
Cl
Cl
3v
¹³C NMR (101 MHz, CDCl₃)
f1 (ppm)

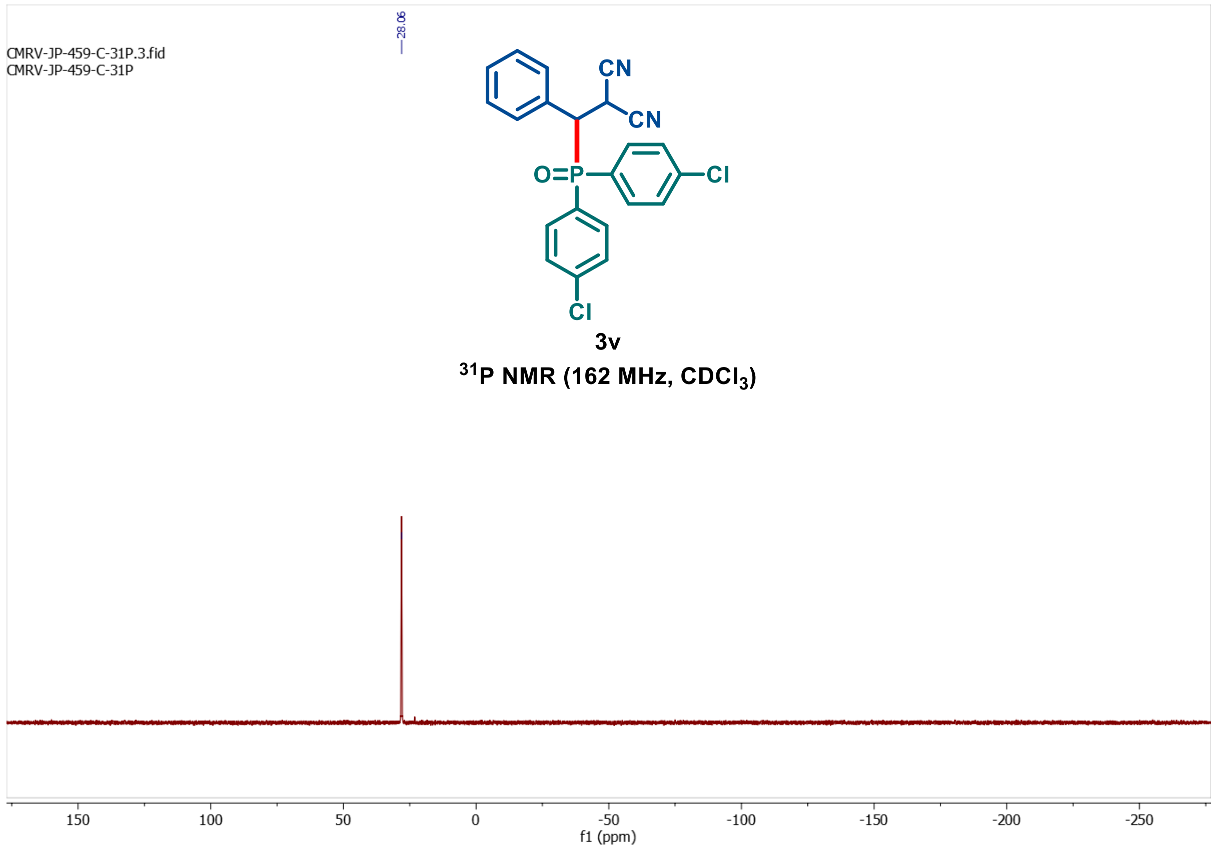

28.06
CMRV-JP-459-C-31P.3.fid
CMRV-JP-459-C-31P
CN
CN
O=P
Cl
Cl
3v
^{31}P NMR (162 MHz, CDCl$_3$)
150
100
50
0
-50
-100
-150
-200
-250
f1 (ppm)

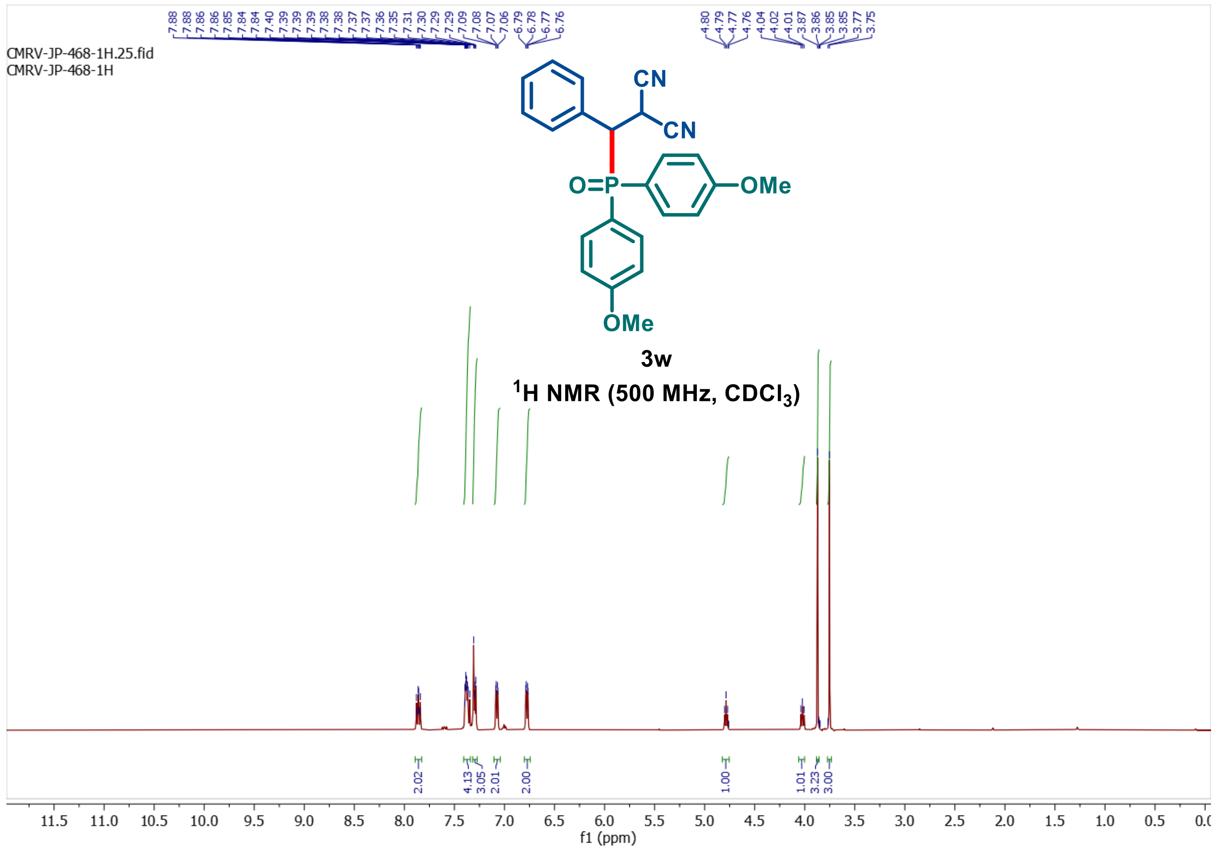

7.88 7.88 7.86 7.86 7.85 7.84 7.40 7.39 7.39 7.39 7.38 7.38 7.37 7.37 7.36 7.35 7.31 7.30 7.29 7.29 7.09 7.08 7.07 7.06 6.79 6.78 6.77 6.76
4.80 4.79 4.77 4.76 4.04 4.02 4.01 3.87 3.86 3.85 3.85 3.77 3.75
CMRV-JP-468-1H.25.fid
CMRV-JP-468-1H
CN
CN
O=P
OMe
OMe
3w
^{1}H NMR (500 MHz, CDCl$_3$)
2.02
4.13
3.05
2.01
2.00
1.00
1.01
3.23
3.00
11.5 11.0 10.5 10.0 9.5 9.0 8.5 8.0 7.5 7.0 6.5 6.0 5.5 5.0 4.5 4.0 3.5 3.0 2.5 2.0 1.5 1.0 0.5 0.0
f1 (ppm)

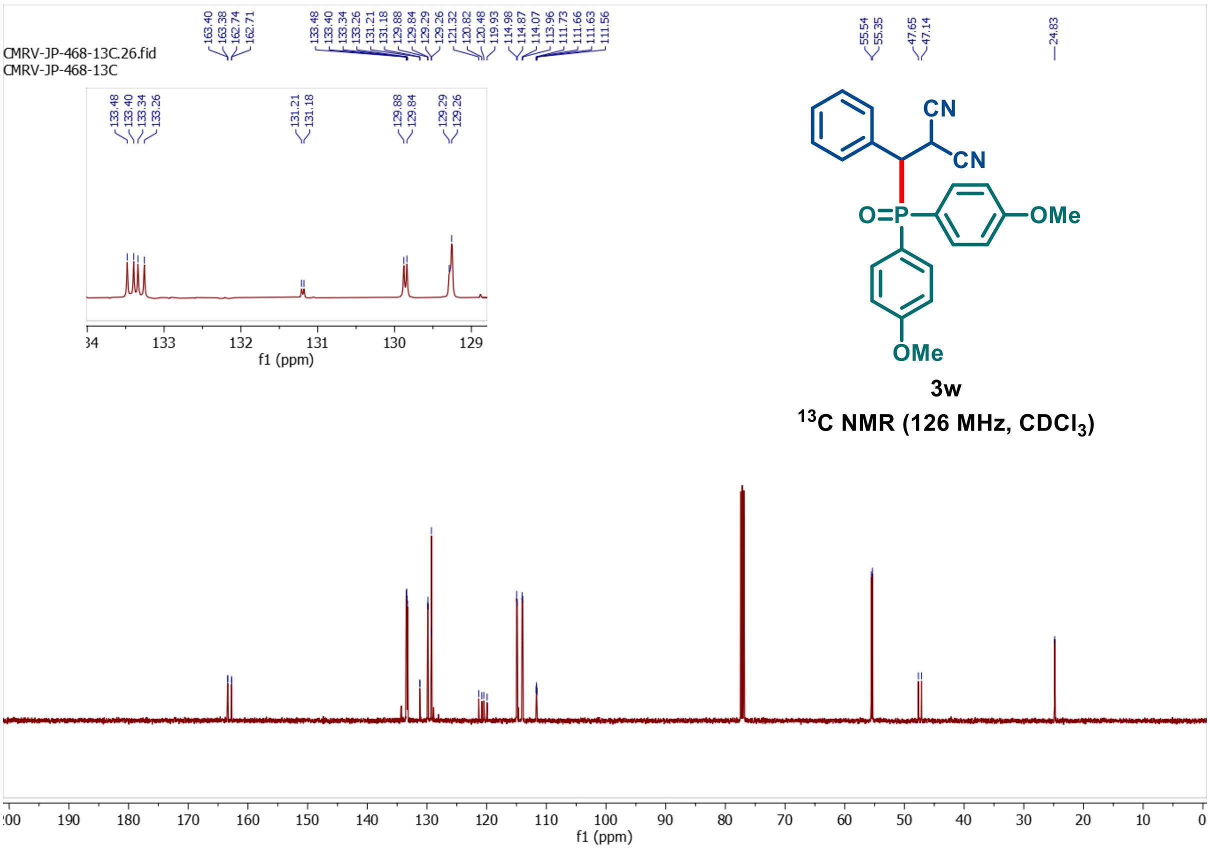
CMRV-JP-468-13C.26.fid
CMRV-JP-468-13C
163.40
163.38
162.74
162.71
133.48
133.40
133.34
133.26
131.21
131.18
129.88
129.84
129.29
129.26
121.32
120.82
120.48
119.93
114.98
114.87
114.07
113.96
111.73
111.66
111.63
111.56
55.54
55.35
47.65
47.14
24.83
CN
CN
O=P
OMe
OMe
3w
13C NMR (126 MHz, CDCl3)
f1 (ppm)

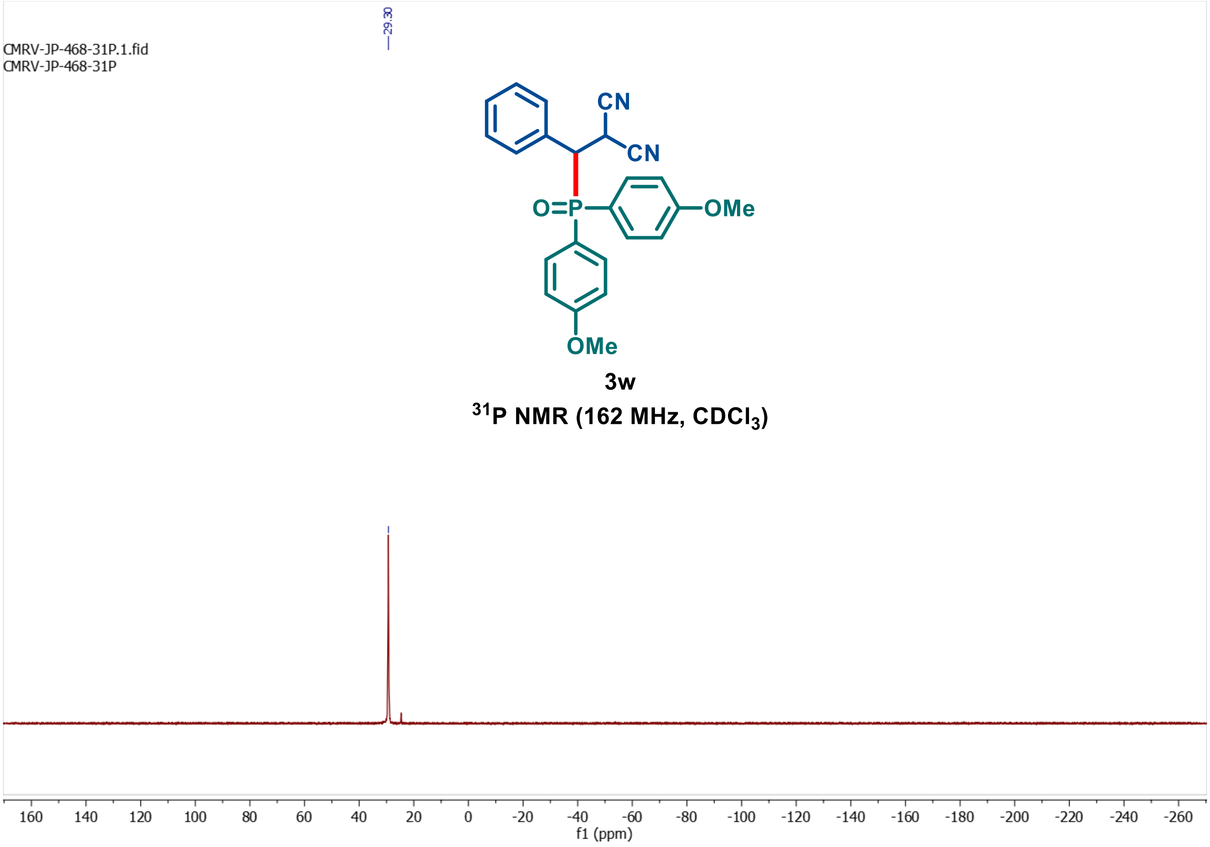
CMRV-JP-468-31P.1.fid
CMRV-JP-468-31P
29.30
CN
CN
O=P
OMe
OMe
3w
31P NMR (162 MHz, CDCl3)
f1 (ppm)

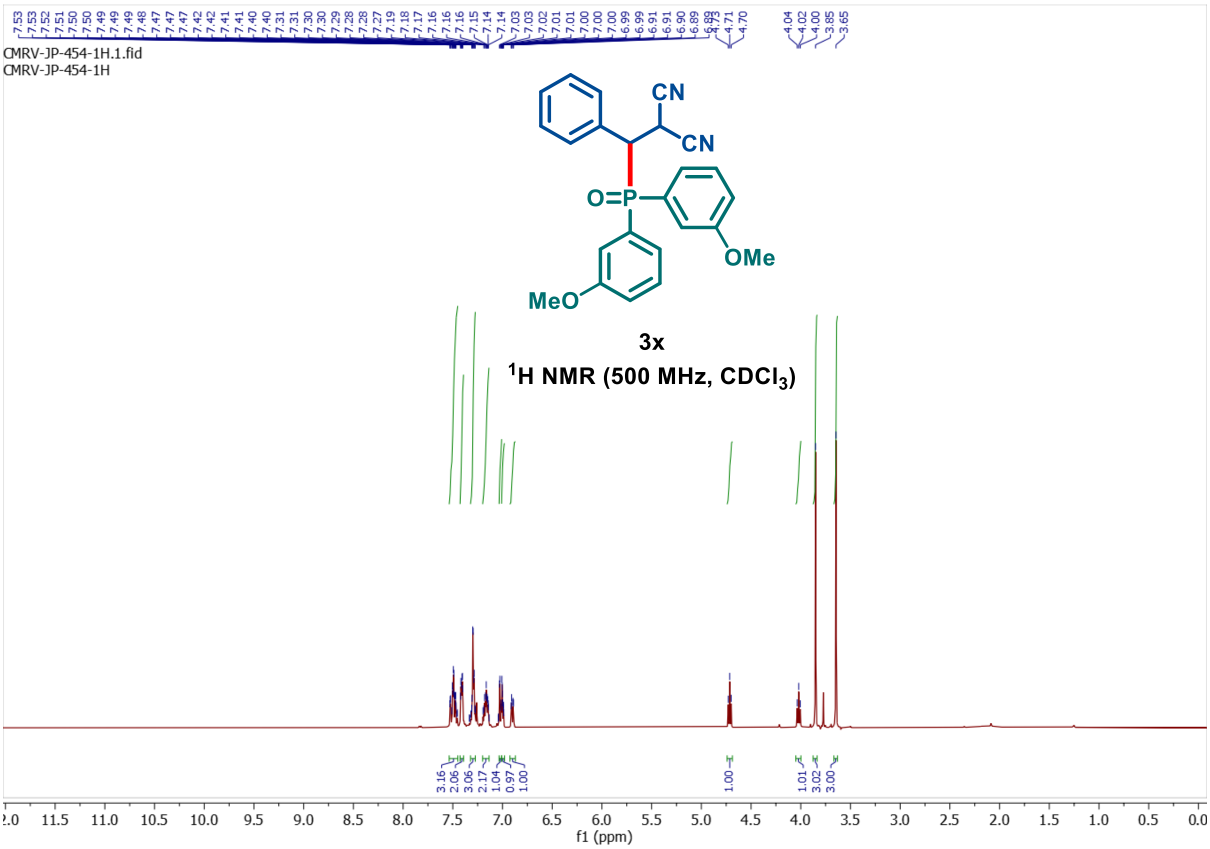
7.53 7.53 7.52 7.51 7.50 7.50 7.49 7.49 7.49 7.48 7.47 7.47 7.42 7.42 7.41 7.40 7.31 7.31 7.30 7.30 7.29 7.28 7.28 7.27 7.19 7.18 7.17 7.16 7.16 7.15 7.14 7.14 7.03 7.03 7.02 7.01 7.01 7.00 7.00 7.00 6.99 6.99 6.91 6.91 6.90 6.89 6.89 4.73 4.71 4.70
4.04 4.02 4.00 3.85 3.65
CMRV-JP-454-1H.1.fid
CMRV-JP-454-1H
CN
CN
O=P
OMe
MeO
3x
1H NMR (500 MHz, CDCl3)
3.16 2.06 3.06 2.17 1.04 0.97 1.00
1.00
1.01 3.02 3.00
f1 (ppm)

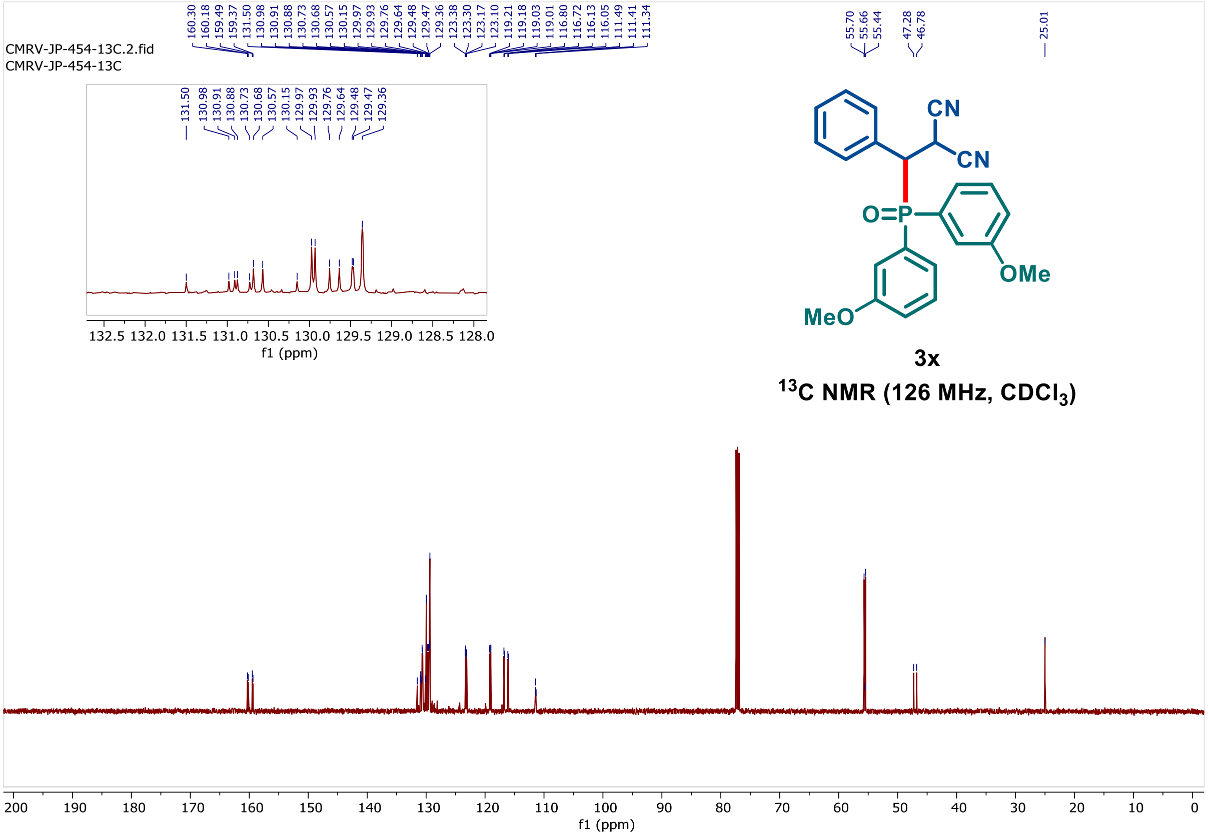
160.30 160.18 159.49 159.37 131.50 130.98 130.91 130.88 130.73 130.68 130.57 130.15 129.97 129.93 129.76 129.64 129.48 129.47 129.36 123.38 123.30 123.17 123.10 119.21 119.18 119.03 119.01 116.80 116.72 116.13 116.05 111.49 111.41 111.34
55.70 55.66 55.44
47.28 46.78
25.01
CMRV-JP-454-13C.2.fid
CMRV-JP-454-13C
131.50 130.98 130.91 130.88 130.73 130.68 130.57 130.15 129.97 129.93 129.76 129.64 129.48 129.47 129.36
f1 (ppm)
CN
CN
O=P
OMe
MeO
3x
13C NMR (126 MHz, CDCl3)
f1 (ppm)

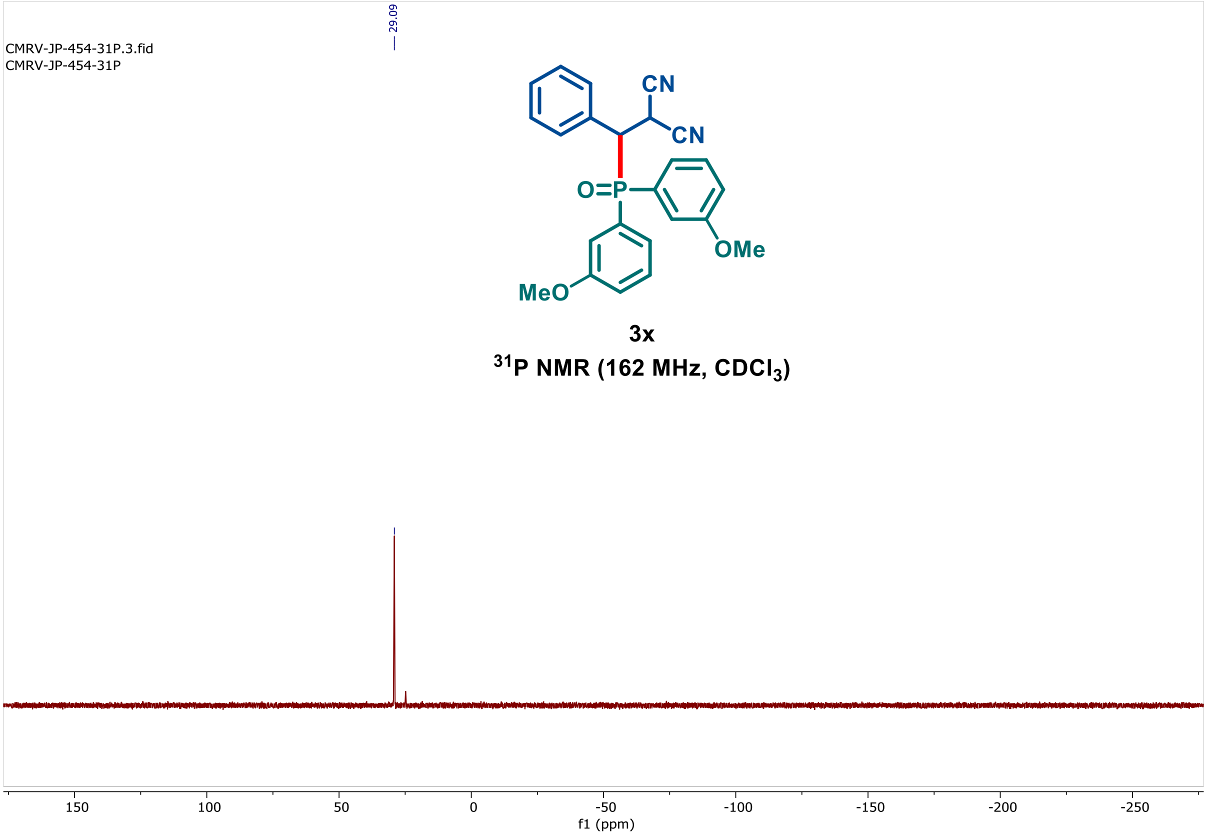

29.09
CMRV-JP-454-31P.3.fid
CMRV-JP-454-31P
CN
CN
O=P
OMe
MeO
3x
31P NMR (162 MHz, CDCl3)
f1 (ppm)

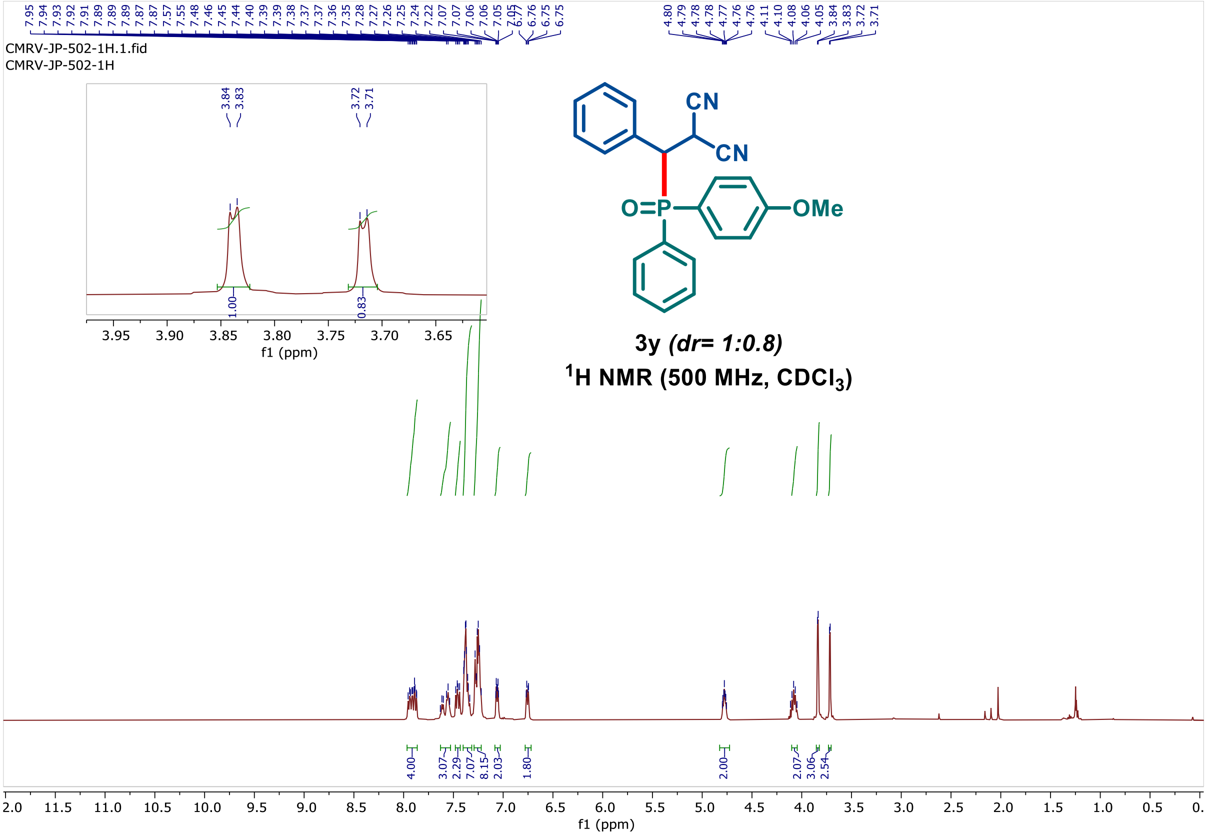

CMRV-JP-502-1H.1.fid
CMRV-JP-502-1H
3.84
3.83
3.72
3.71
1.00
0.83
f1 (ppm)
CN
CN
O=P
OMe
3y (dr= 1:0.8)
1H NMR (500 MHz, CDCl3)
4.00
3.07
2.29
7.07
8.15
2.03
1.80
2.00
2.07
3.06
2.54
f1 (ppm)

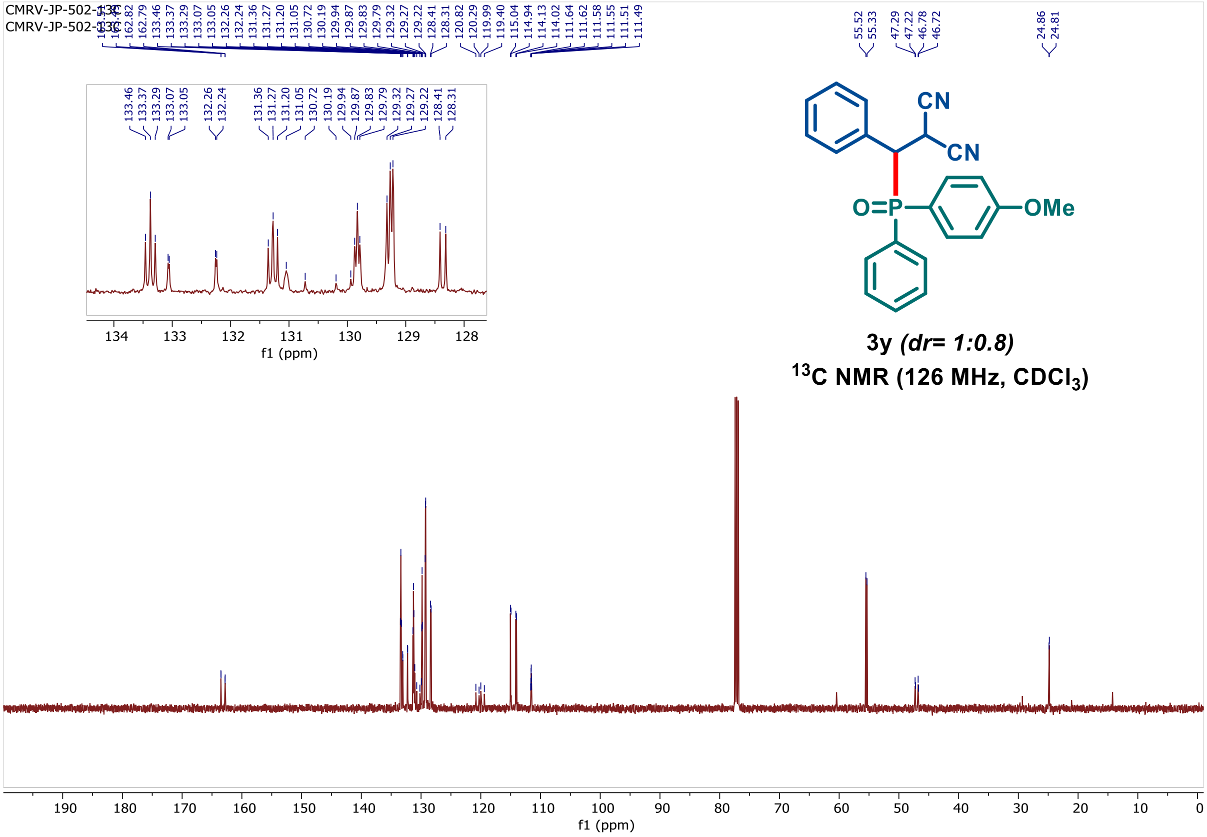
CMRV-JP-502-13C
CMRV-JP-502-13C
162.82
162.79
133.46
133.37
133.29
133.07
133.05
132.26
132.24
131.36
131.27
131.20
131.05
130.72
130.19
129.94
129.87
129.83
129.79
129.32
129.27
129.22
128.41
128.31
120.82
120.29
119.99
119.40
115.04
114.94
114.13
114.02
111.64
111.62
111.58
111.55
111.51
111.49
55.52
55.33
47.29
47.22
46.78
46.72
24.86
24.81
CN
CN
O=P
OMe
3y (dr= 1:0.8)
13C NMR (126 MHz, CDCl3)
f1 (ppm)

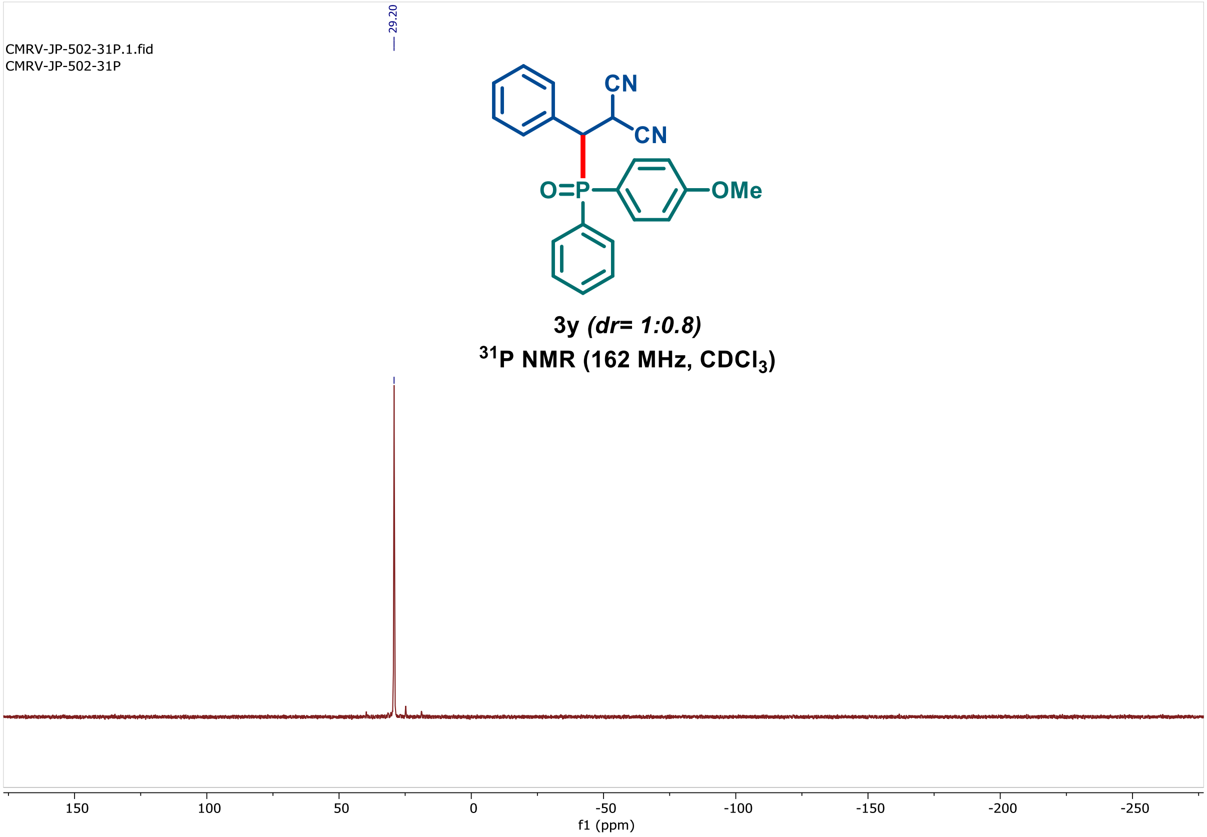
29.20
CMRV-JP-502-31P.1.fid
CMRV-JP-502-31P
CN
CN
O=P
OMe
3y (dr= 1:0.8)
31P NMR (162 MHz, CDCl3)
f1 (ppm)

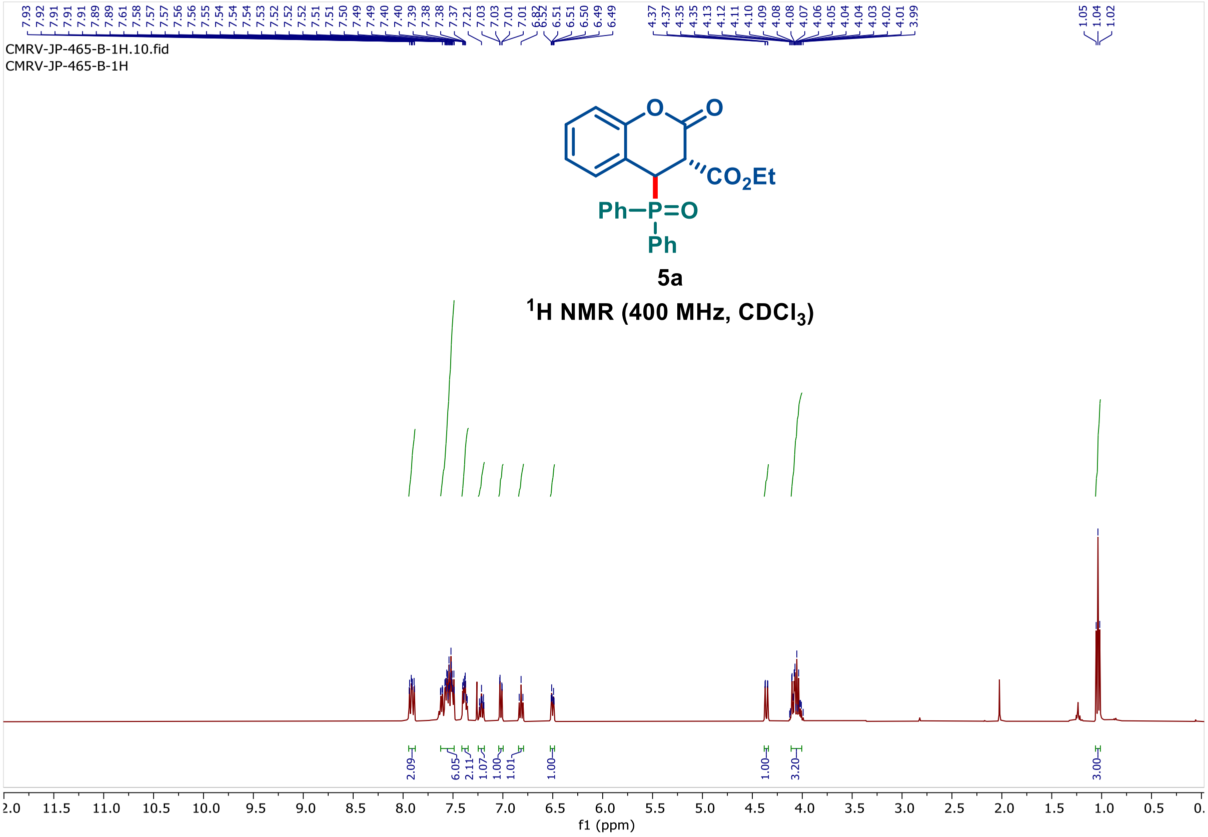
CMRV-JP-465-B-1H.10.fid
CMRV-JP-465-B-1H
5a
1H NMR (400 MHz, CDCl3)
7.93 7.92 7.91 7.91 7.89 7.89 7.61 7.58 7.57 7.57 7.56 7.56 7.55 7.54 7.54 7.54 7.53 7.52 7.52 7.52 7.51 7.51 7.50 7.49 7.49 7.40 7.40 7.39 7.38 7.38 7.37 7.21 7.03 7.03 7.01 7.01 6.82 6.52 6.51 6.51 6.50 6.49 6.49
4.37 4.37 4.35 4.35 4.13 4.12 4.11 4.10 4.09 4.08 4.08 4.07 4.06 4.05 4.04 4.04 4.03 4.02 4.01 3.99
1.05 1.04 1.02
2.09 6.05 2.11 1.07 1.00 1.01 1.00 1.00 3.20 3.00
f1 (ppm)

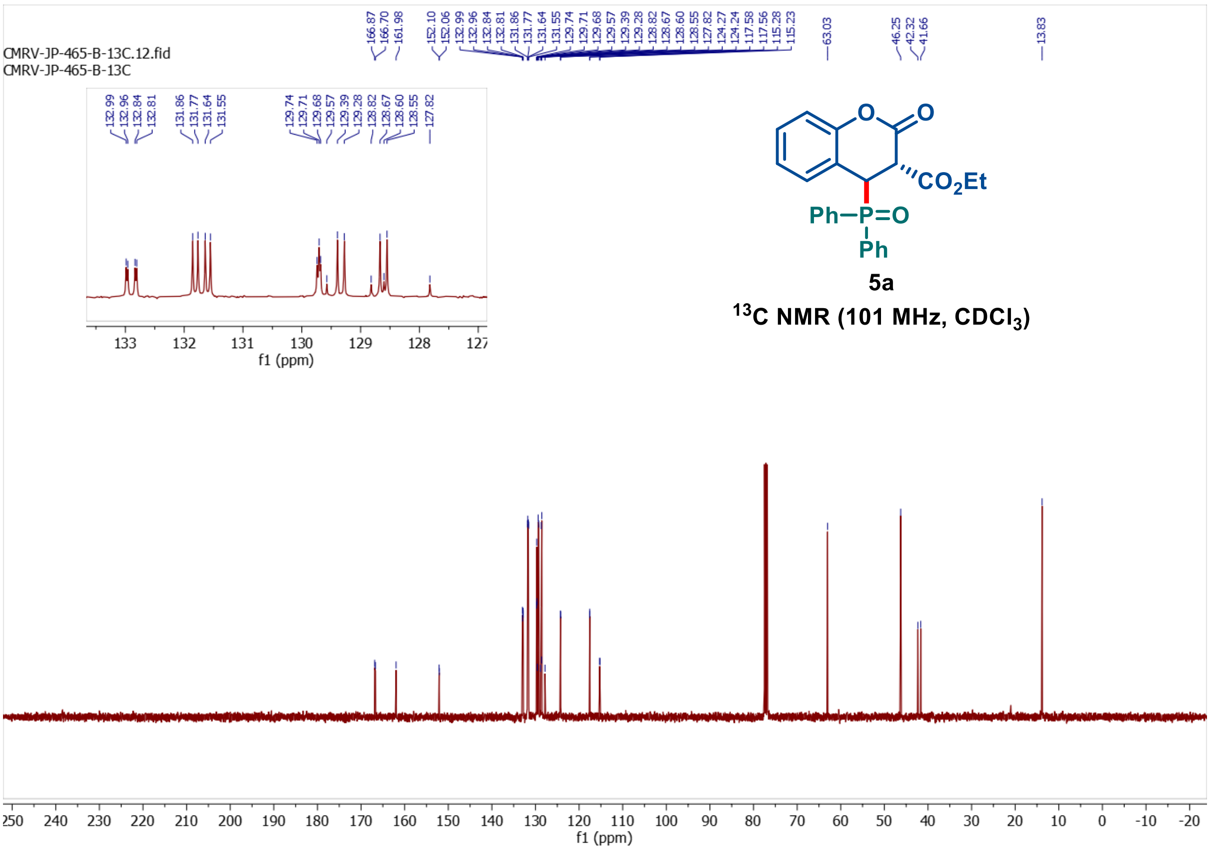
CMRV-JP-465-B-13C.12.fid
CMRV-JP-465-B-13C
166.87 166.70 161.98 152.10 152.06 132.99 132.96 132.84 132.81 131.86 131.77 131.64 131.55 129.74 129.71 129.68 129.57 129.39 129.28 128.82 128.67 128.60 128.55 127.82 124.27 124.24 117.58 117.56 115.28 115.23 63.03 46.25 42.32 41.66 13.83
5a
13C NMR (101 MHz, CDCl3)
f1 (ppm)

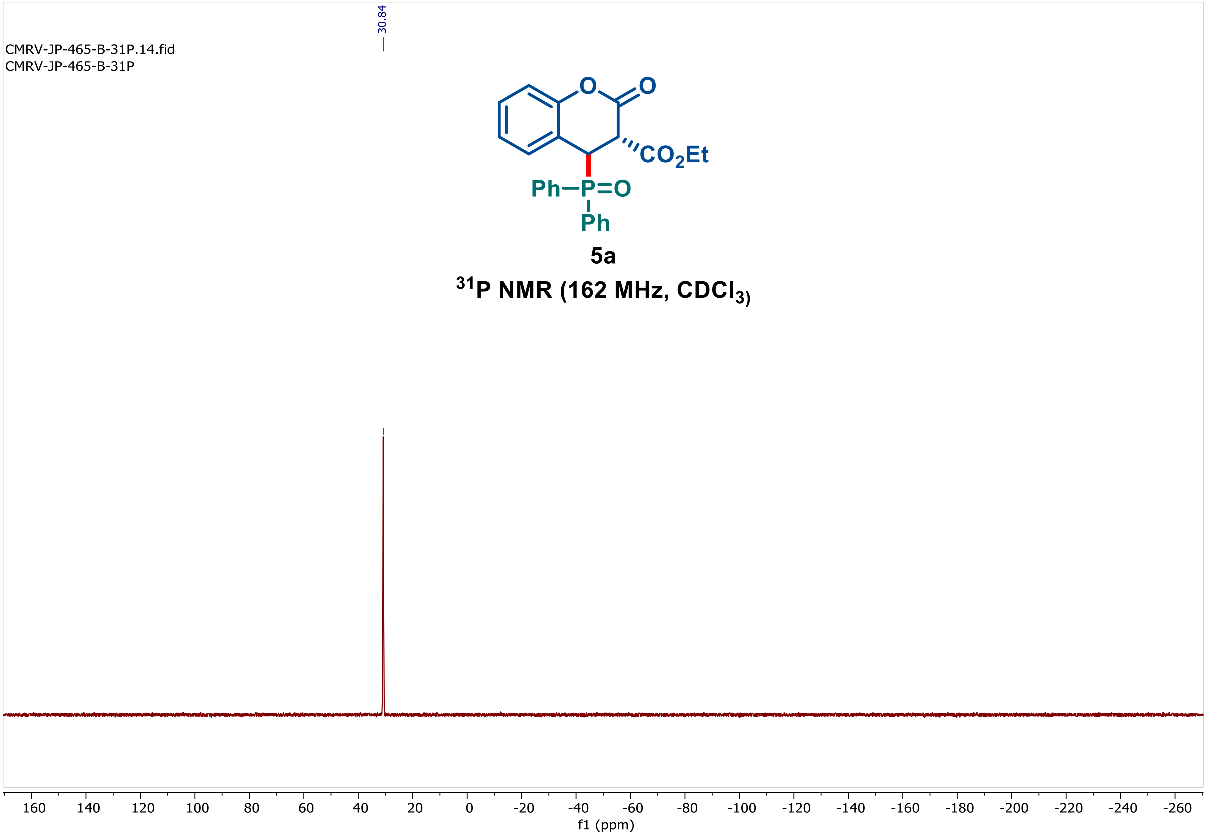
CMRV-JP-465-B-31P.14.fid
CMRV-JP-465-B-31P
30.84
CO_2Et
Ph
P=O
Ph
5a
^{31}P NMR (162 MHz, $CDCl_3$)
f1 (ppm)

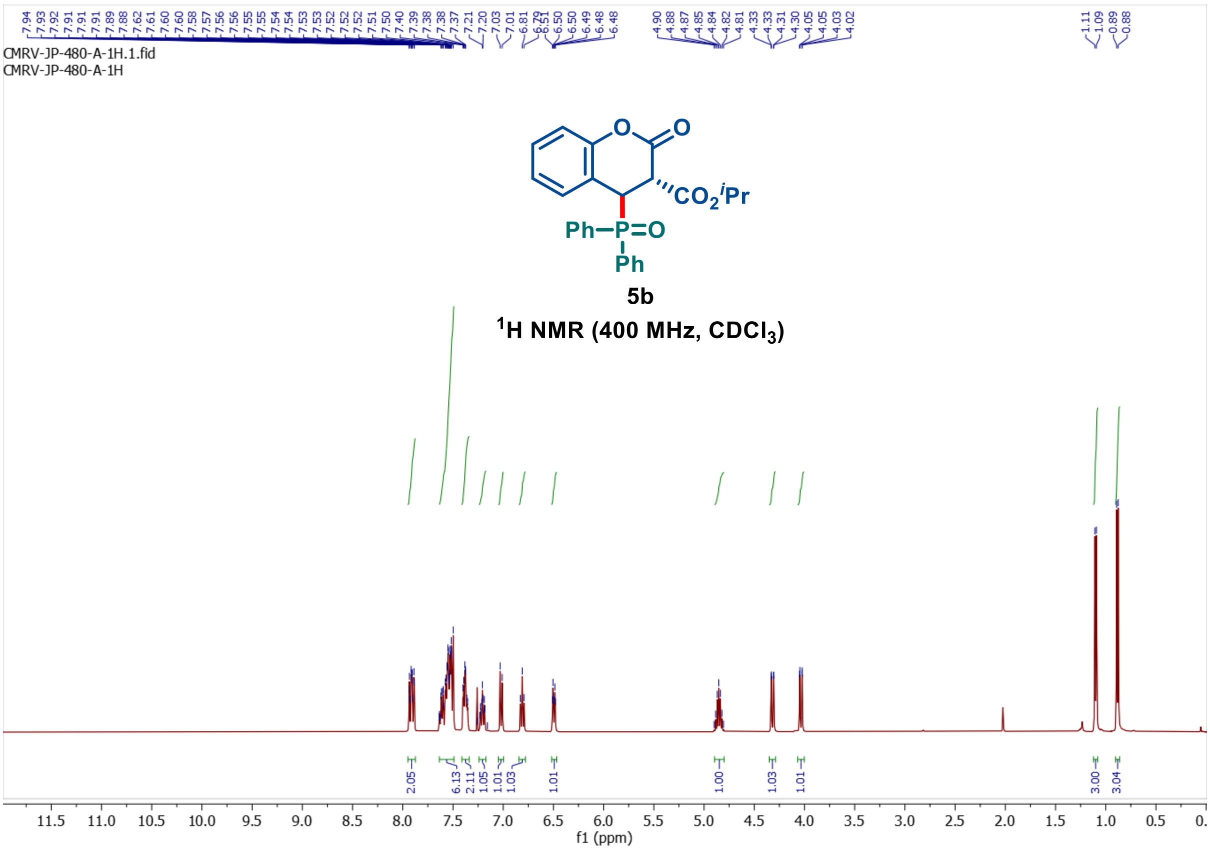
CMRV-JP-480-A-1H.1.fid
CMRV-JP-480-A-1H
$CO_2{}^iPr$
Ph
P=O
Ph
5b
^1H NMR (400 MHz, $CDCl_3$)
2.05
6.13
2.11
1.05
1.01
1.03
1.01
1.00
1.03
1.01
3.00
3.04
f1 (ppm)

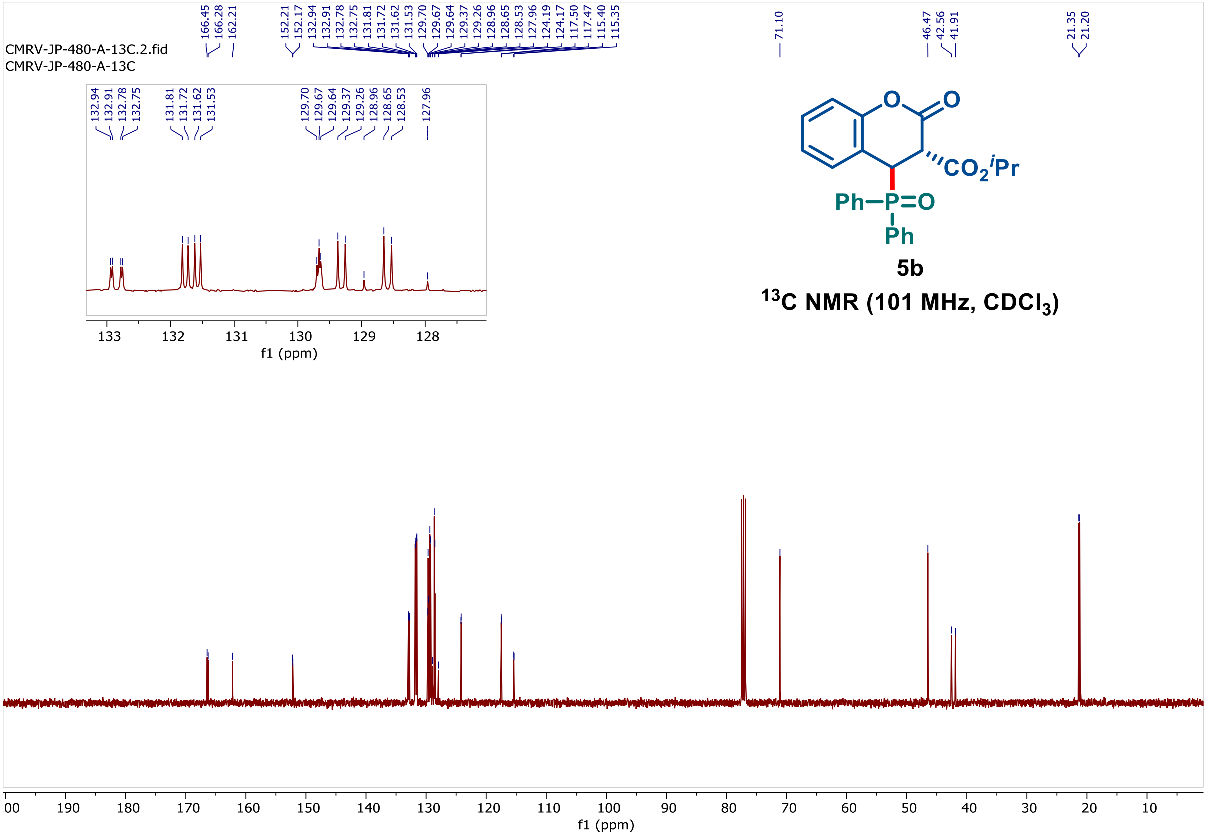
CMRV-JP-480-A-13C.2.fid
CMRV-JP-480-A-13C
166.45
166.28
162.21
152.21
152.17
132.94
132.91
132.78
132.75
131.81
131.72
131.62
131.53
129.70
129.67
129.64
129.37
129.26
128.96
128.65
128.53
127.96
124.19
124.17
117.50
117.47
115.40
115.35
71.10
46.47
42.56
41.91
21.35
21.20
5b
^{13}C NMR (101 MHz, $CDCl_3$)
f1 (ppm)

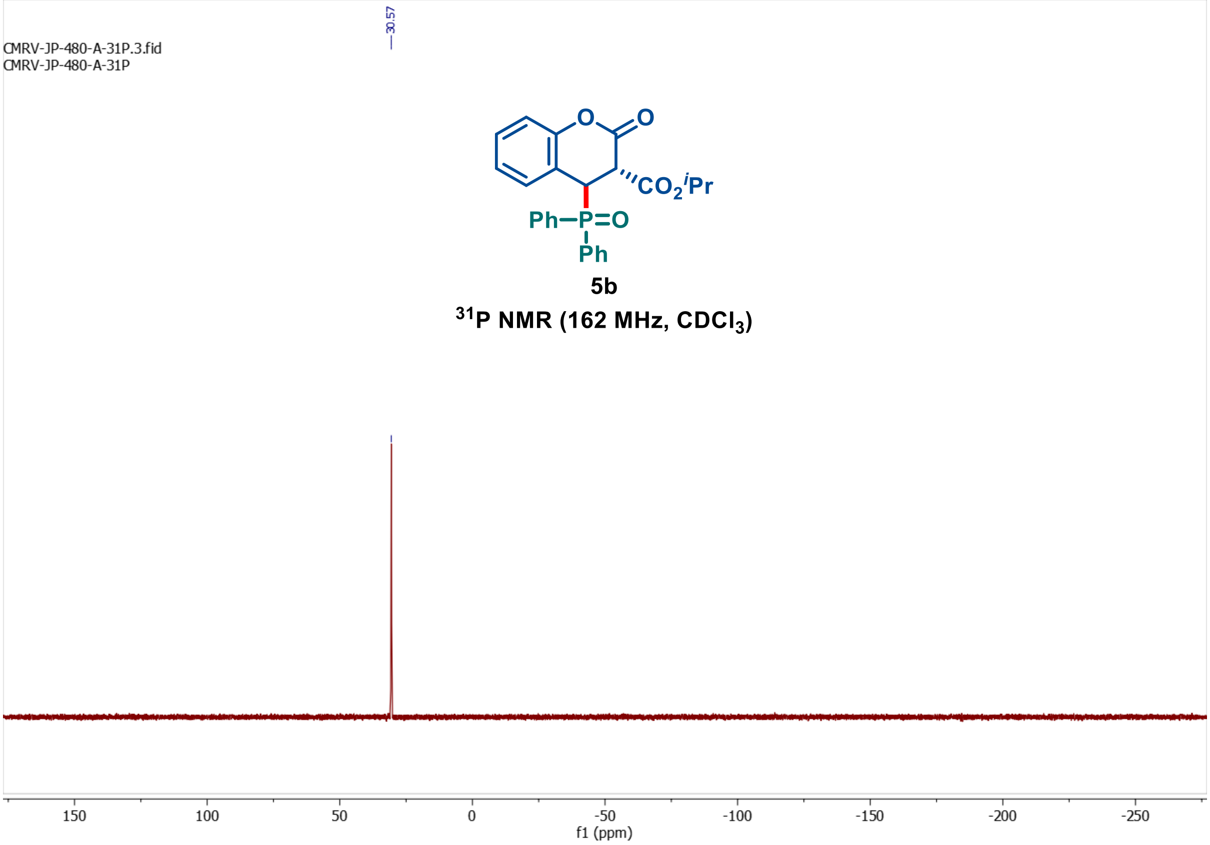
CMRV-JP-480-A-31P.3.fid
CMRV-JP-480-A-31P
30.57
5b
^{31}P NMR (162 MHz, $CDCl_3$)
f1 (ppm)

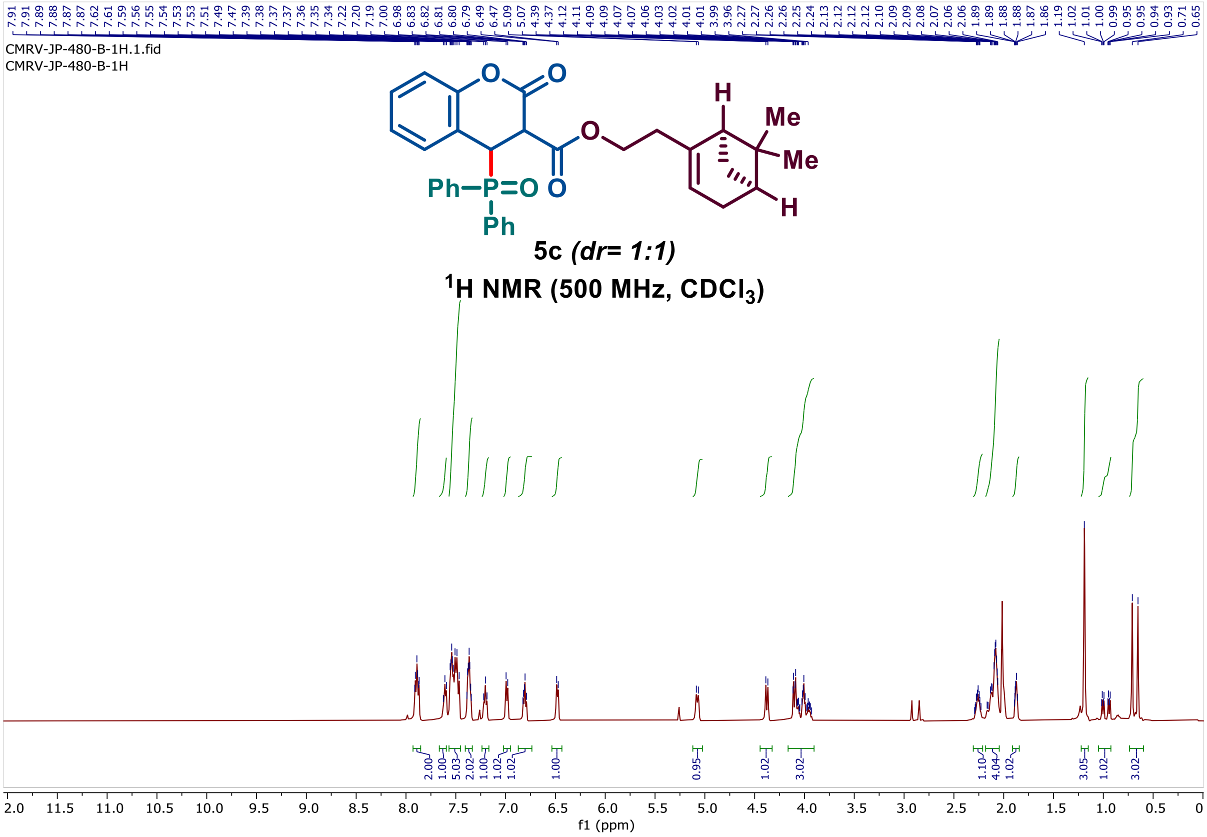
7.91 7.91 7.89 7.88 7.87 7.87 7.62 7.61 7.59 7.56 7.55 7.54 7.53 7.53 7.51 7.49 7.47 7.39 7.38 7.37 7.37 7.36 7.35 7.34 7.22 7.20 7.19 7.00 6.98 6.83 6.82 6.81 6.80 6.79 6.49 6.47 5.09 5.07 4.39 4.37 4.12 4.11 4.09 4.09 4.07 4.06 4.03 4.02 4.01 4.01 3.99 3.96 2.27 2.27 2.26 2.26 2.25 2.24 2.13 2.12 2.12 2.12 2.10 2.09 2.09 2.08 2.07 2.06 2.06 1.89 1.89 1.88 1.88 1.87 1.86 1.19 1.02 1.01 1.00 0.99 0.95 0.95 0.94 0.93 0.71 0.65
CMRV-JP-480-B-1H.1.fid
CMRV-JP-480-B-1H
O
O
O
O
H
Me
Me
H
Ph—P=O
Ph
5c (dr= 1:1)
1H NMR (500 MHz, CDCl3)
2.00 1.00 5.03 2.02 1.00 1.02 1.02 1.00 0.95 1.02 3.02 1.10 4.04 1.02 3.05 1.02 3.02
f1 (ppm)

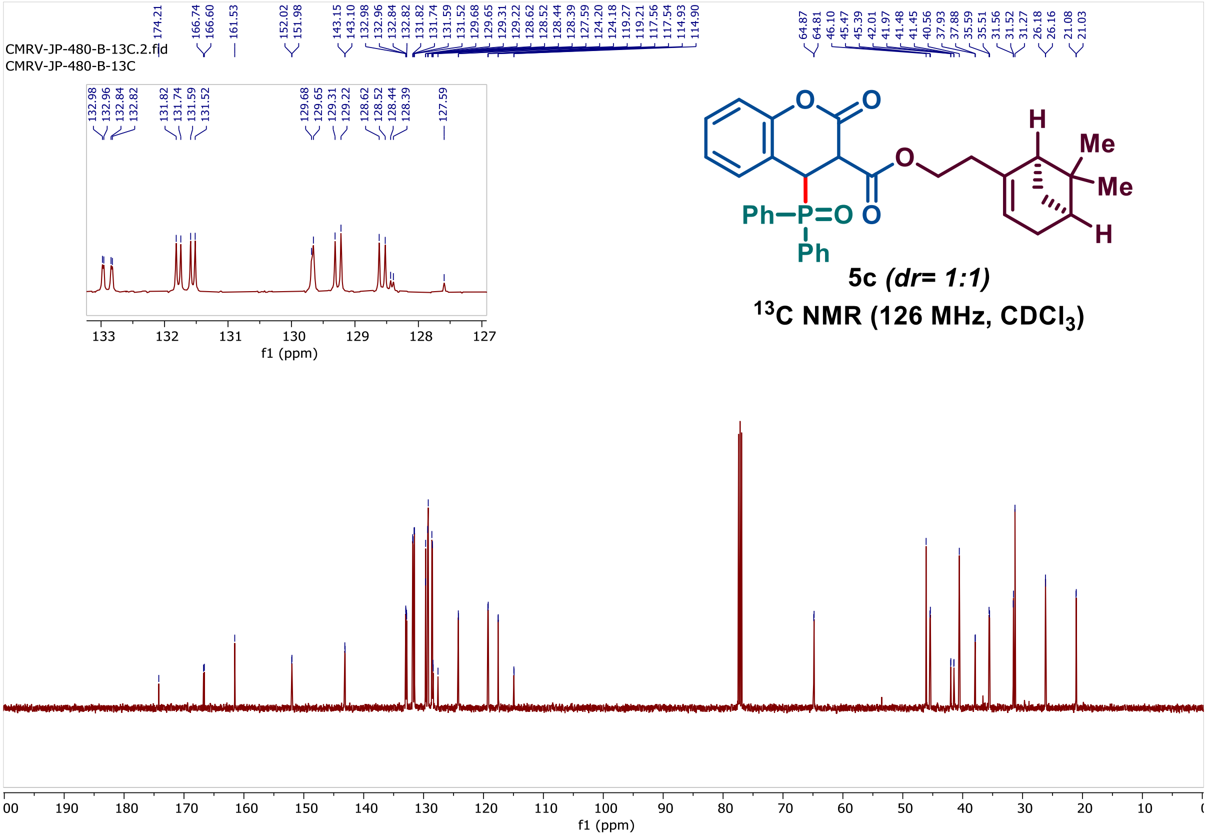
174.21 166.74 166.60 161.53 152.02 151.98 143.15 143.10 132.98 132.96 132.84 132.82 131.82 131.74 131.59 131.52 129.68 129.65 129.31 129.22 128.62 128.52 128.44 128.39 127.59 124.20 124.18 119.27 119.21 117.56 117.54 114.93 114.90 64.87 64.81 46.10 45.47 45.39 42.01 41.97 41.48 41.45 40.56 37.93 37.88 35.59 35.51 31.56 31.52 31.27 26.18 26.16 21.08 21.03
CMRV-JP-480-B-13C.2.fid
CMRV-JP-480-B-13C
f1 (ppm)
5c (dr= 1:1)
13C NMR (126 MHz, CDCl3)
f1 (ppm)

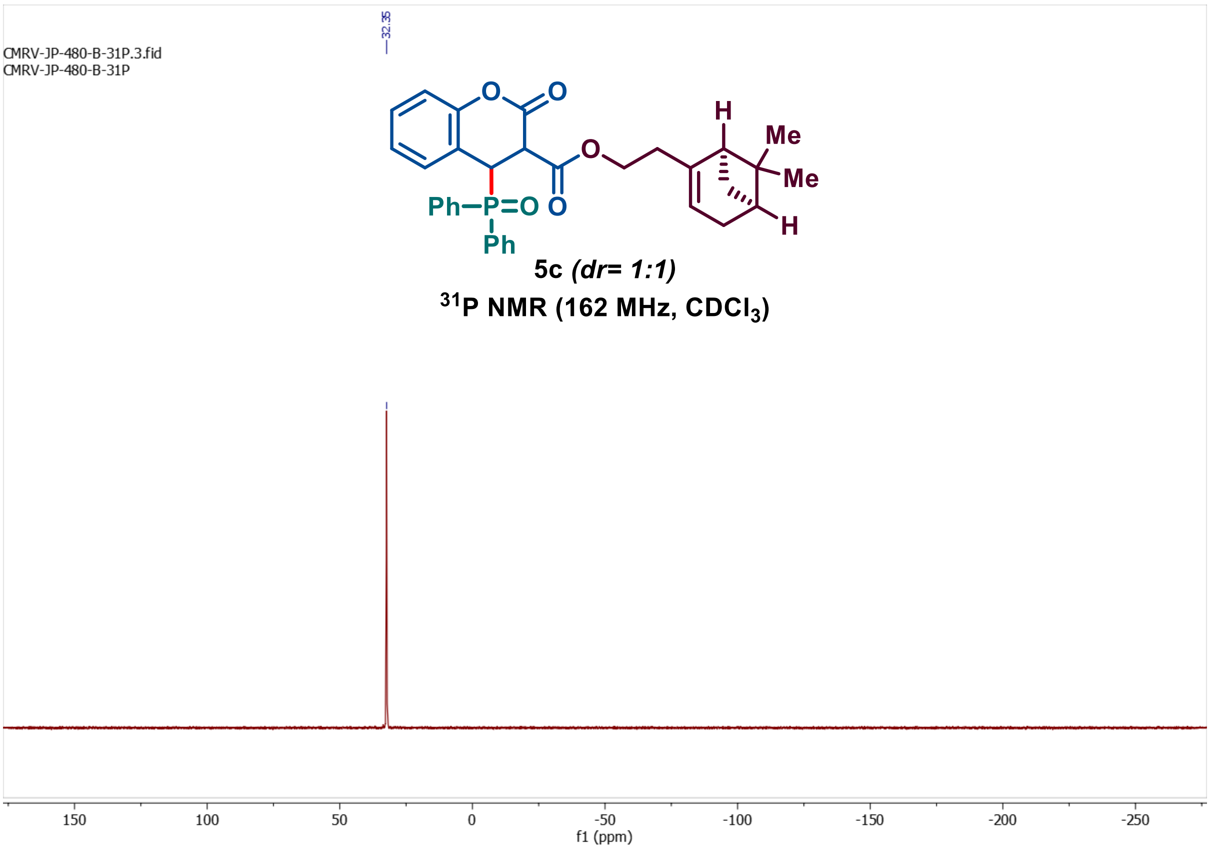
32.35
CMRV-JP-480-B-31P.3.fid
CMRV-JP-480-B-31P
O
O
O
O
H
Me
Me
H
Ph–P=O
Ph
5c (dr= 1:1)
31P NMR (162 MHz, CDCl3)
150
100
50
0
-50
-100
-150
-200
-250
f1 (ppm)

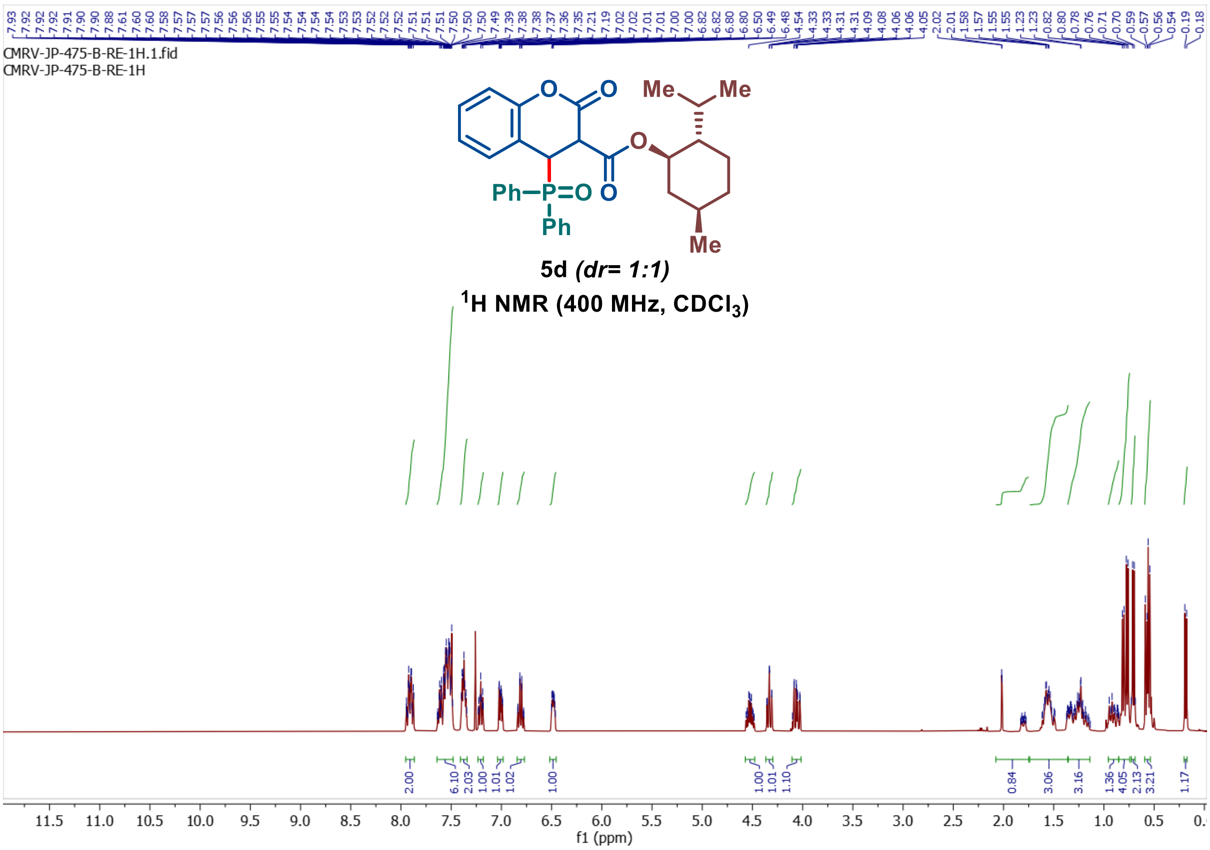
7.93 7.92 7.92 7.91 7.90 7.88 7.61 7.60 7.58 7.57 7.57 7.56 7.56 7.55 7.54 7.54 7.53 7.53 7.52 7.52 7.51 7.51 7.51 7.50 7.50 7.49 7.39 7.38 7.38 7.37 7.36 7.35 7.21 7.19 7.02 7.02 7.01 7.01 7.00 6.82 6.80 6.80 6.50 6.49 6.48 4.54 4.33 4.33 4.31 4.31 4.09 4.08 4.06 4.05 2.02 2.01 1.58 1.57 1.55 1.55 1.23 1.23 0.82 0.80 0.78 0.76 0.71 0.70 0.59 0.57 0.56 0.54 0.19 0.18
CMRV-JP-475-B-RE-1H.1.fid
CMRV-JP-475-B-RE-1H
O
O
Me
Me
O
O
Ph–P=O
Ph
Me
5d (dr= 1:1)
1H NMR (400 MHz, CDCl3)
2.00
6.10
2.03
1.00
1.01
1.02
1.00
1.00
1.01
1.10
0.84
3.06
3.16
1.36
4.05
2.13
3.21
1.17
11.5
11.0
10.5
10.0
9.5
9.0
8.5
8.0
7.5
7.0
6.5
6.0
5.5
5.0
4.5
4.0
3.5
3.0
2.5
2.0
1.5
1.0
0.5
0.
f1 (ppm)

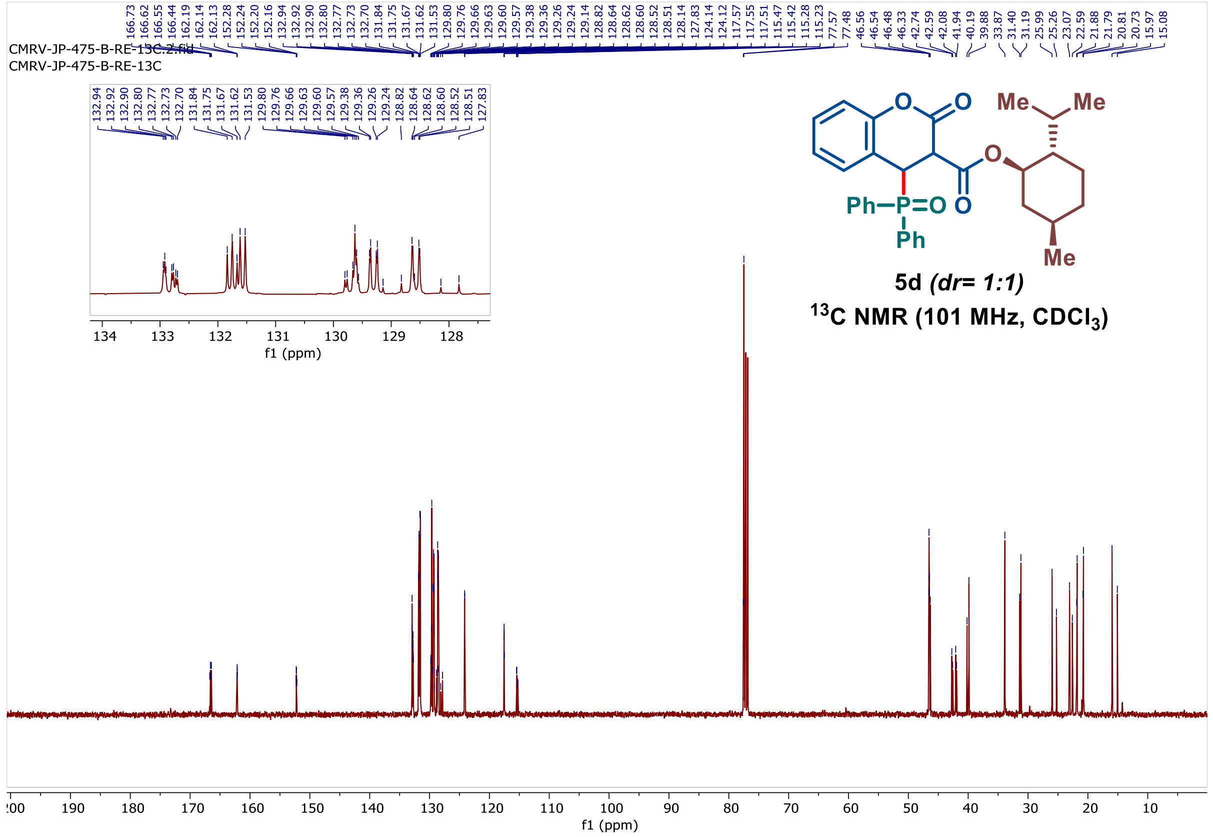
CMRV-JP-475-B-RE-13C.2.fid
CMRV-JP-475-B-RE-13C
5d (dr= 1:1)
13C NMR (101 MHz, CDCl3)
f1 (ppm)

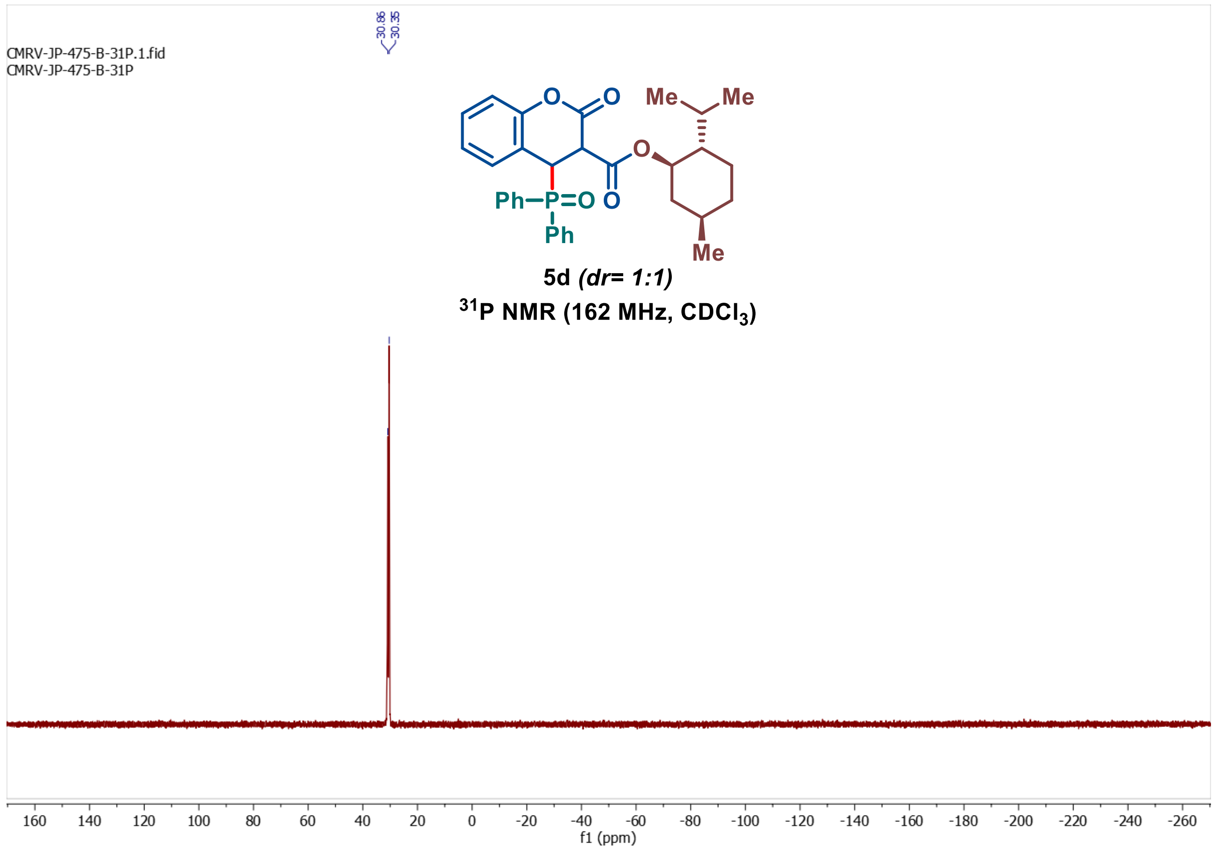
CMRV-JP-475-B-31P.1.fid
CMRV-JP-475-B-31P
30.86
30.35
5d (dr= 1:1)
31P NMR (162 MHz, CDCl3)
f1 (ppm)

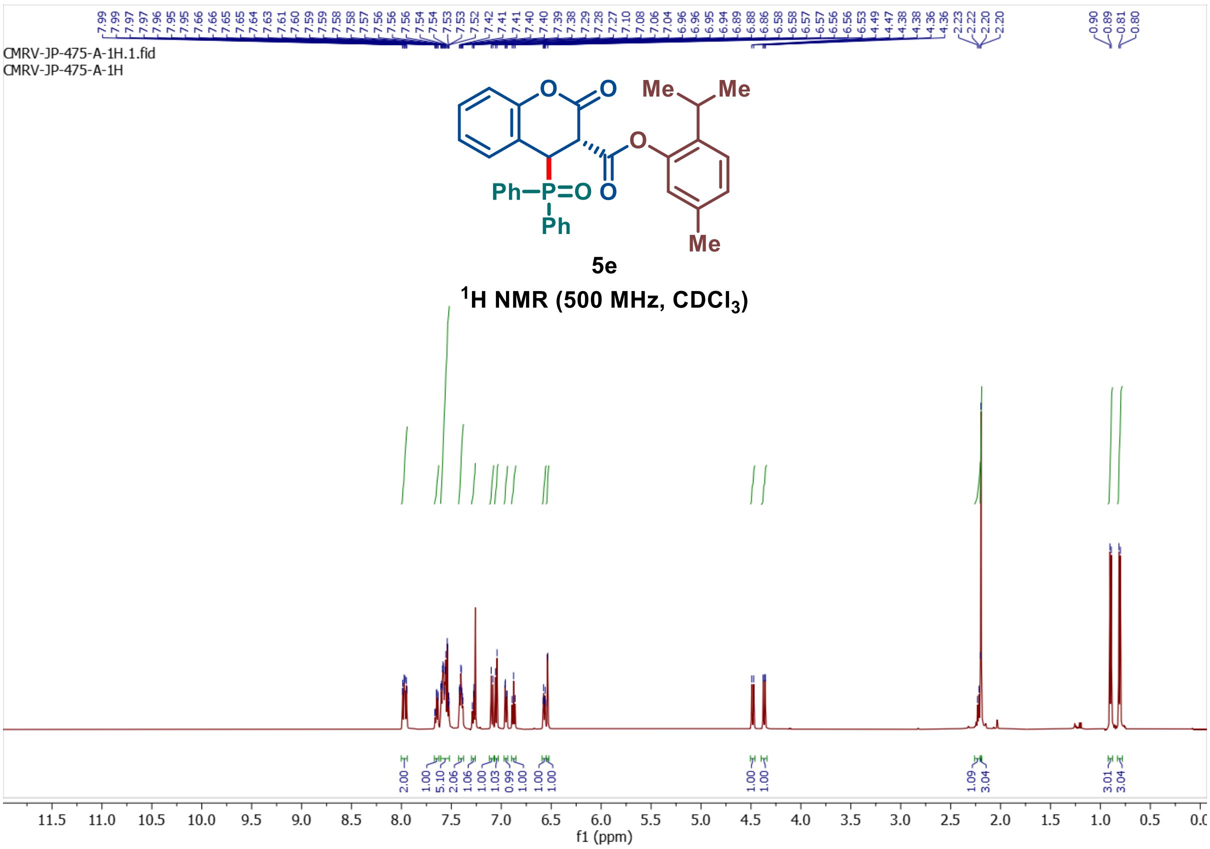

CMRV-JP-475-A-1H.1.fid
CMRV-JP-475-A-1H
5e
^{1}H NMR (500 MHz, CDCl$_3$)
f1 (ppm)

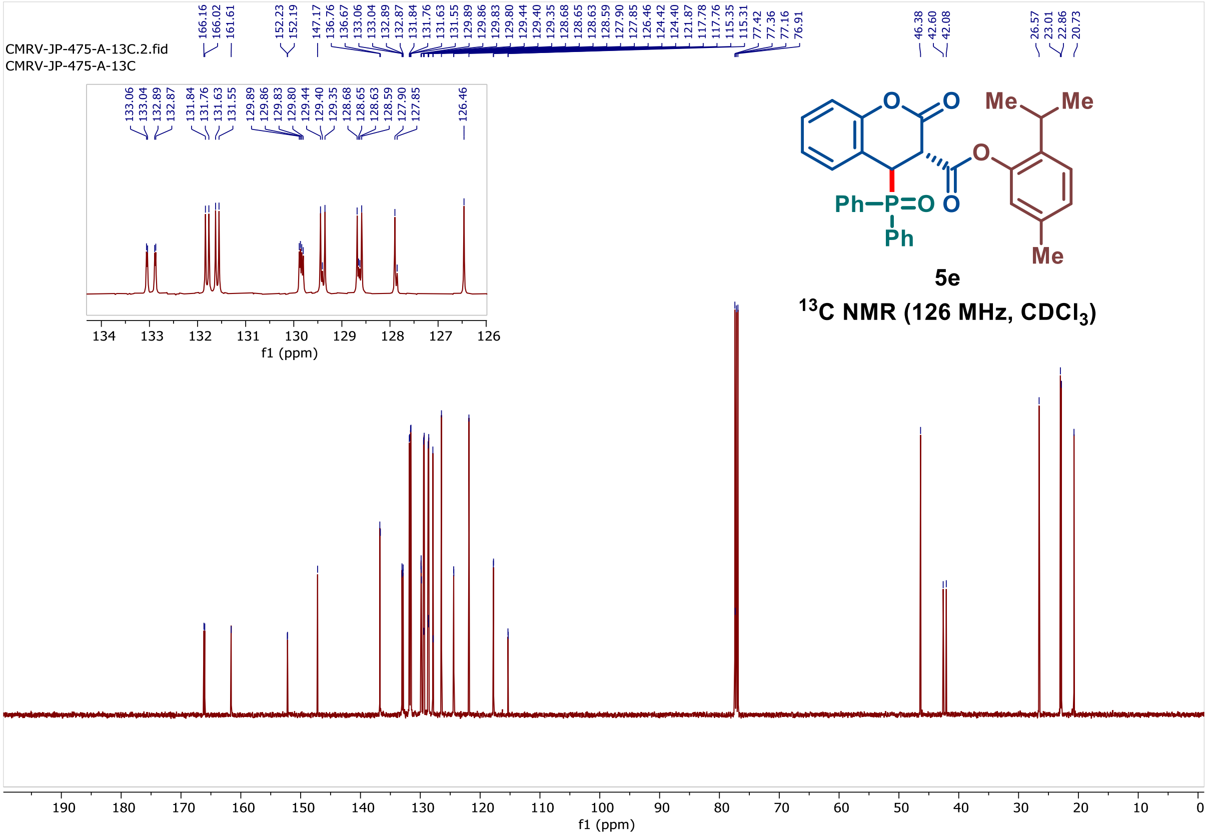

CMRV-JP-475-A-13C.2.fid
CMRV-JP-475-A-13C
5e
^{13}C NMR (126 MHz, CDCl$_3$)
f1 (ppm)

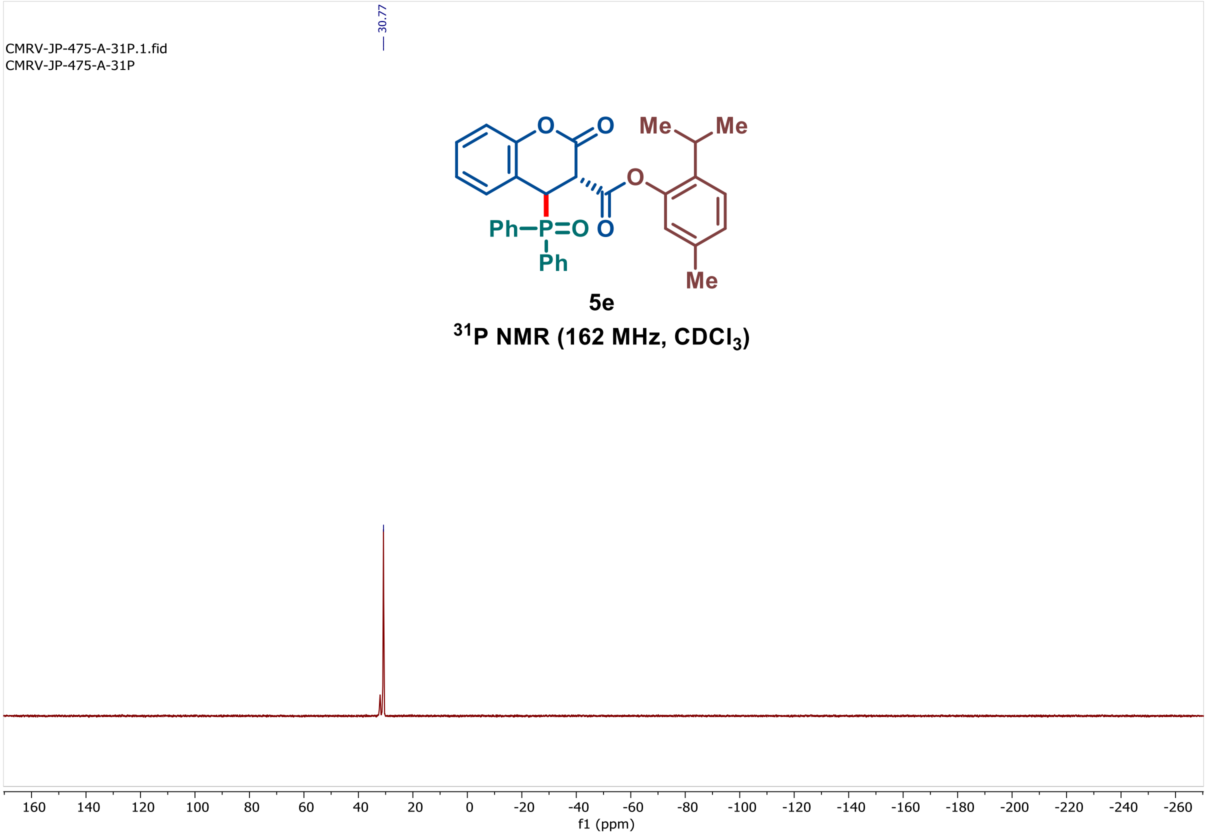
30.77
CMRV-JP-475-A-31P.1.fid
CMRV-JP-475-A-31P
Me
Me
O
O
O
O
Ph–P=O
Ph
Me
5e
^{31}P NMR (162 MHz, CDCl$_3$)
160 140 120 100 80 60 40 20 0 -20 -40 -60 -80 -100 -120 -140 -160 -180 -200 -220 -240 -260
f1 (ppm)

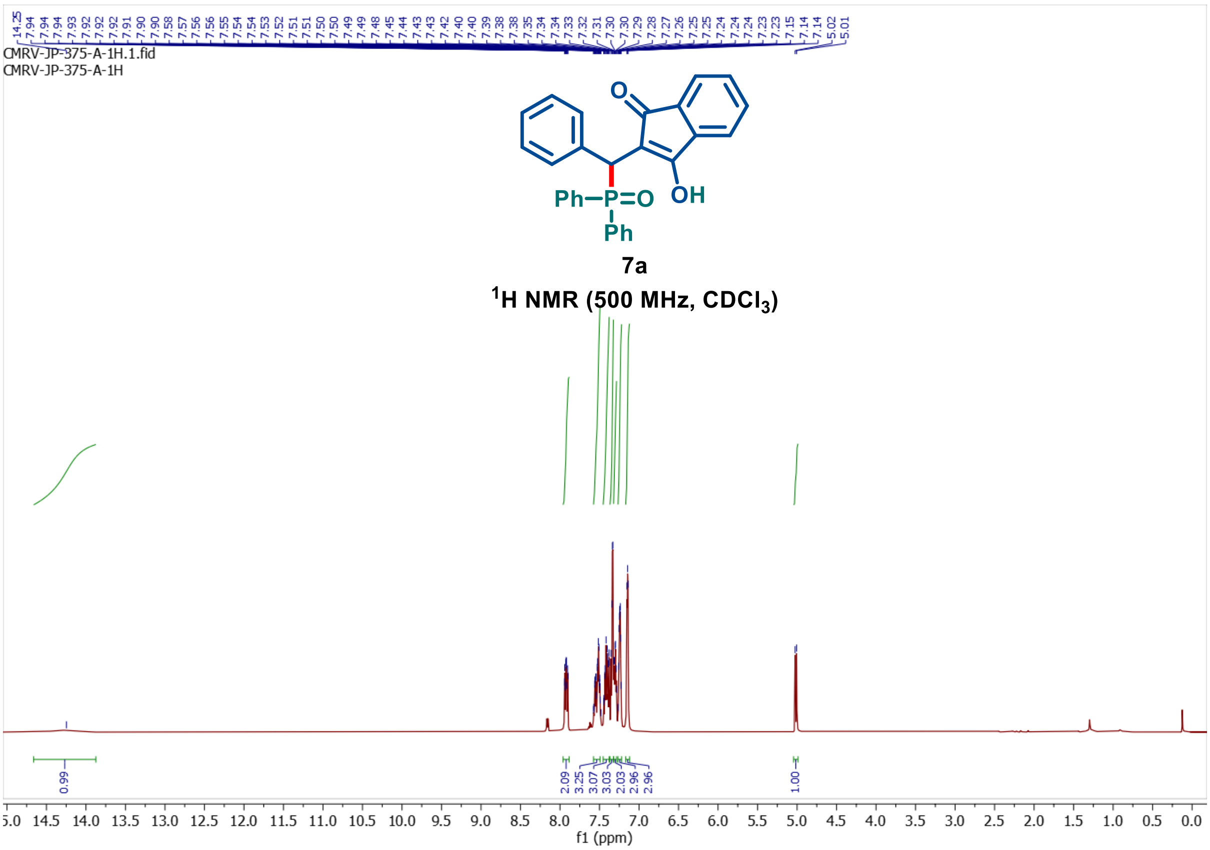
14.25 7.94 7.94 7.94 7.93 7.92 7.92 7.92 7.91 7.90 7.90 7.58 7.57 7.56 7.55 7.54 7.54 7.53 7.52 7.51 7.51 7.50 7.50 7.49 7.49 7.48 7.45 7.44 7.43 7.43 7.42 7.40 7.40 7.39 7.38 7.38 7.35 7.34 7.34 7.33 7.32 7.31 7.30 7.30 7.29 7.28 7.27 7.26 7.25 7.25 7.24 7.24 7.24 7.23 7.23 7.15 7.14 7.14 5.02 5.01
CMRV-JP-375-A-1H.1.fid
CMRV-JP-375-A-1H
O
Ph–P=O
Ph
OH
7a
^{1}H NMR (500 MHz, CDCl$_3$)
0.99
2.09 3.25 3.07 3.03 2.03 2.96 2.96
1.00
15.0 14.5 14.0 13.5 13.0 12.5 12.0 11.5 11.0 10.5 10.0 9.5 9.0 8.5 8.0 7.5 7.0 6.5 6.0 5.5 5.0 4.5 4.0 3.5 3.0 2.5 2.0 1.5 1.0 0.5 0.0
f1 (ppm)

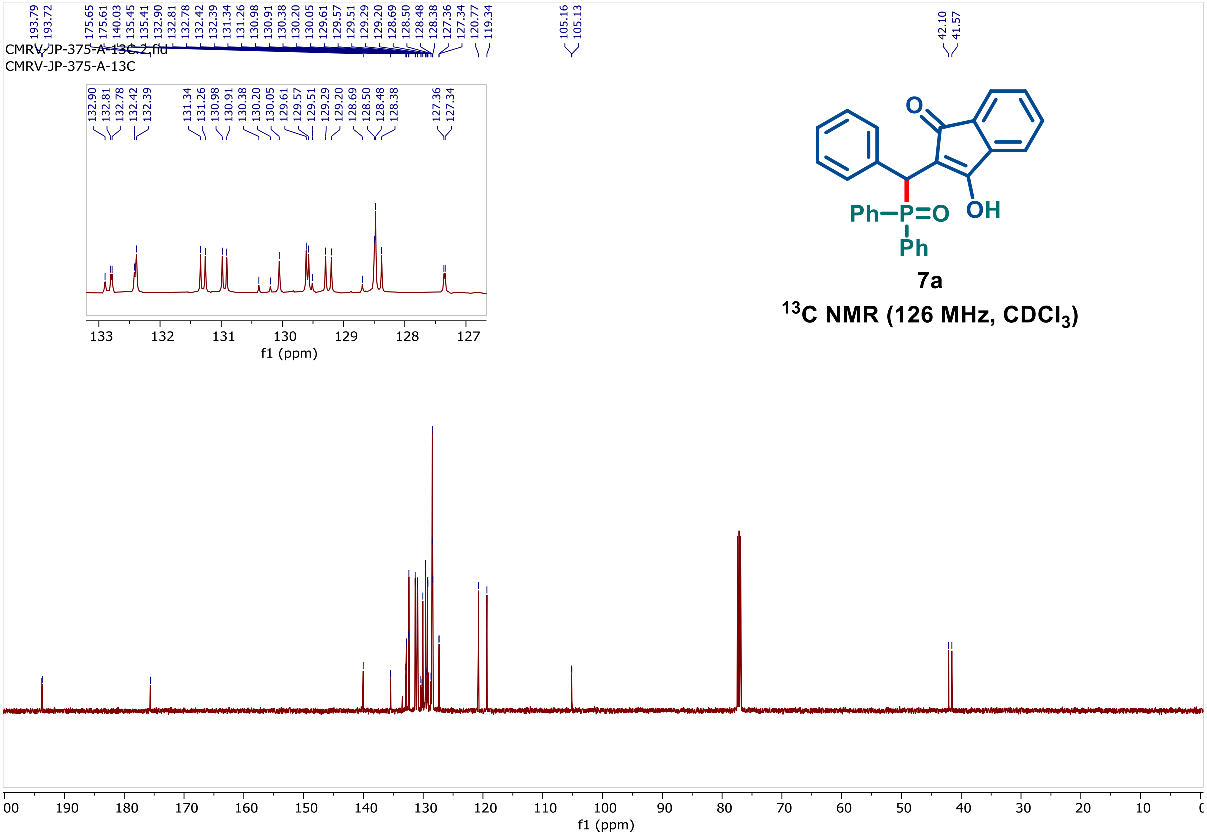
CMRV-JP-375-A-13C.2.fid
CMRV-JP-375-A-13C
193.79
193.72
175.65
175.61
140.03
135.45
135.41
132.90
132.81
132.78
132.42
132.39
131.34
131.26
130.98
130.91
130.38
130.20
130.05
129.61
129.57
129.51
129.29
129.20
128.69
128.50
128.48
128.38
127.36
127.34
120.77
119.34
105.16
105.13
42.10
41.57
7a
^{13}C NMR (126 MHz, $CDCl_3$)
f1 (ppm)

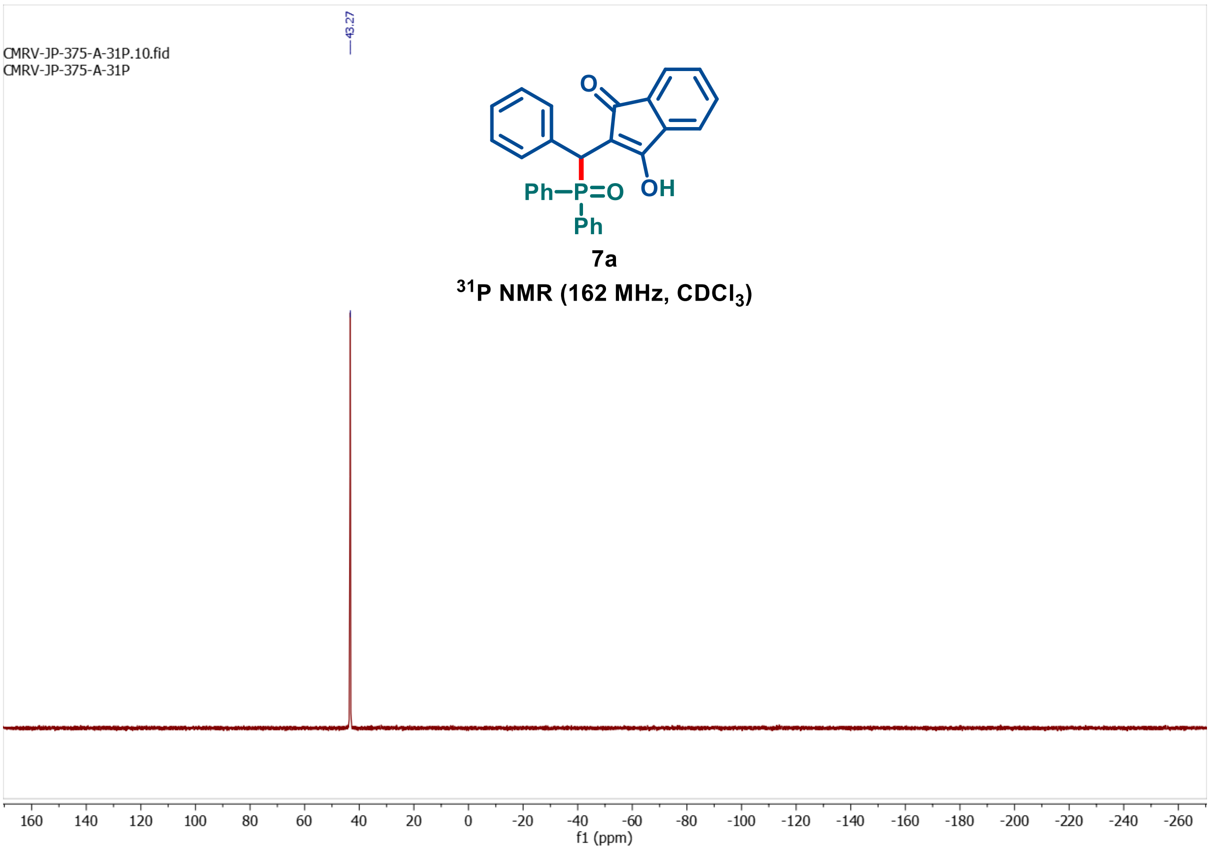
CMRV-JP-375-A-31P.10.fid
CMRV-JP-375-A-31P
43.27
7a
^{31}P NMR (162 MHz, $CDCl_3$)
f1 (ppm)

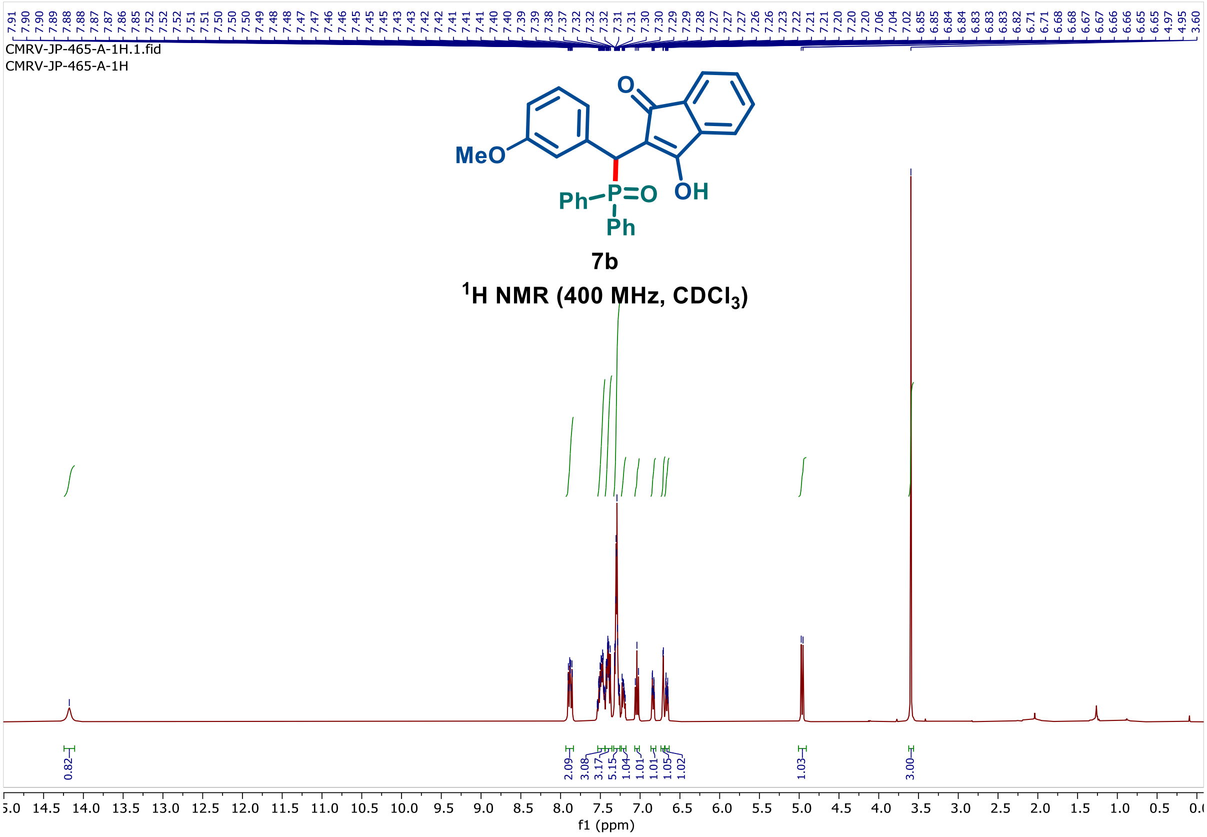
7.91 7.90 7.90 7.89 7.88 7.88 7.87 7.87 7.86 7.85 7.52 7.52 7.52 7.51 7.51 7.50 7.50 7.49 7.48 7.48 7.47 7.47 7.46 7.46 7.45 7.45 7.45 7.43 7.43 7.42 7.42 7.41 7.41 7.41 7.40 7.40 7.39 7.39 7.38 7.37 7.32 7.32 7.32 7.31 7.31 7.30 7.30 7.29 7.29 7.28 7.27 7.27 7.27 7.26 7.26 7.23 7.22 7.21 7.21 7.20 7.20 7.20 7.06 7.04 7.02 6.85 6.85 6.84 6.83 6.83 6.83 6.82 6.71 6.71 6.68 6.68 6.67 6.67 6.66 6.66 6.65 6.65 4.97 4.95 3.60
CMRV-JP-465-A-1H.1.fid
CMRV-JP-465-A-1H
MeO
O
OH
Ph
P=O
Ph
7b
^{1}H NMR (400 MHz, CDCl$_3$)
0.82
2.09
3.08
3.17
5.15
1.04
1.01
1.01
1.05
1.02
1.03
3.00
f1 (ppm)

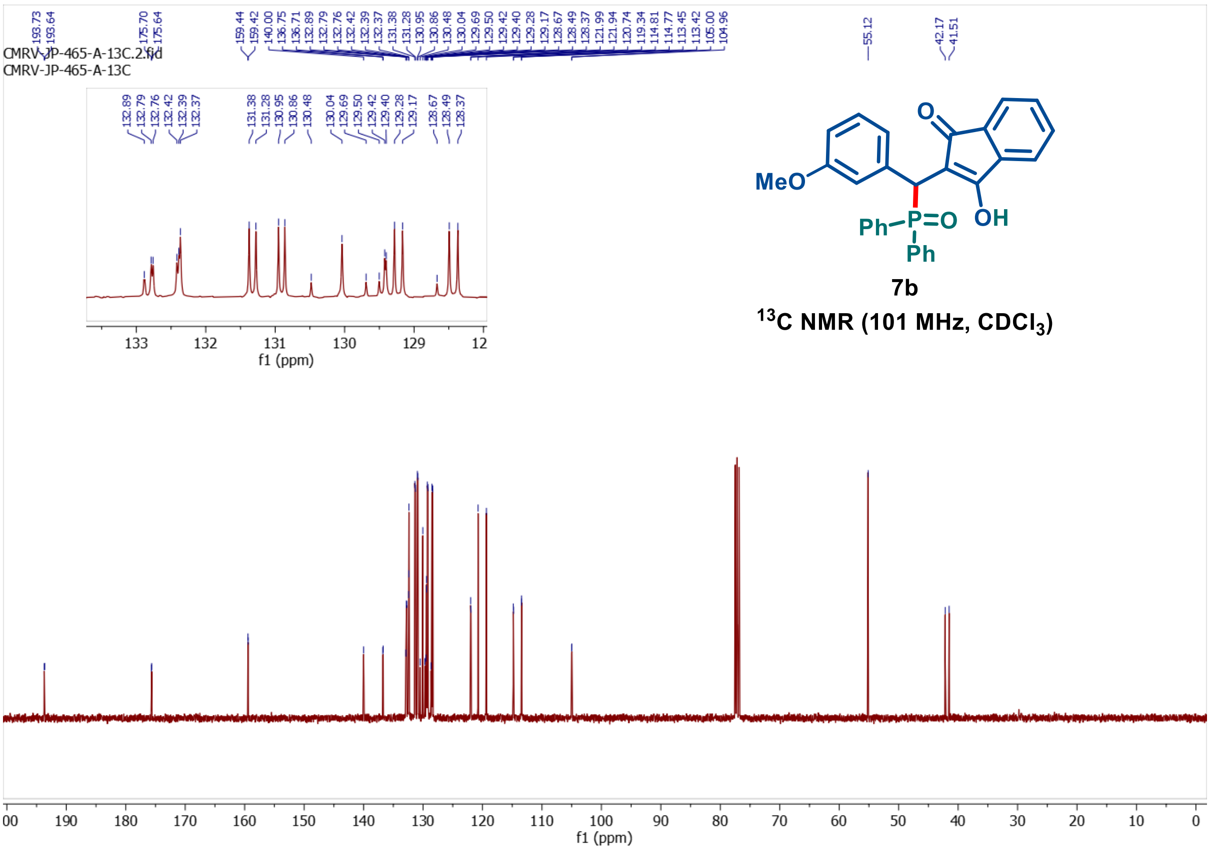
193.73 193.64 175.70 175.64 159.44 159.42 140.00 136.75 136.71 132.89 132.79 132.76 132.42 132.39 132.37 131.38 131.28 130.95 130.86 130.48 130.04 129.69 129.50 129.42 129.40 129.28 129.17 128.67 128.49 128.37 121.99 121.94 120.74 119.34 114.81 114.77 113.45 113.42 105.00 104.96
55.12
42.17 41.51
CMRV-JP-465-A-13C.2.fid
CMRV-JP-465-A-13C
f1 (ppm)
MeO
O
OH
Ph
P=O
Ph
7b
^{13}C NMR (101 MHz, CDCl$_3$)
f1 (ppm)

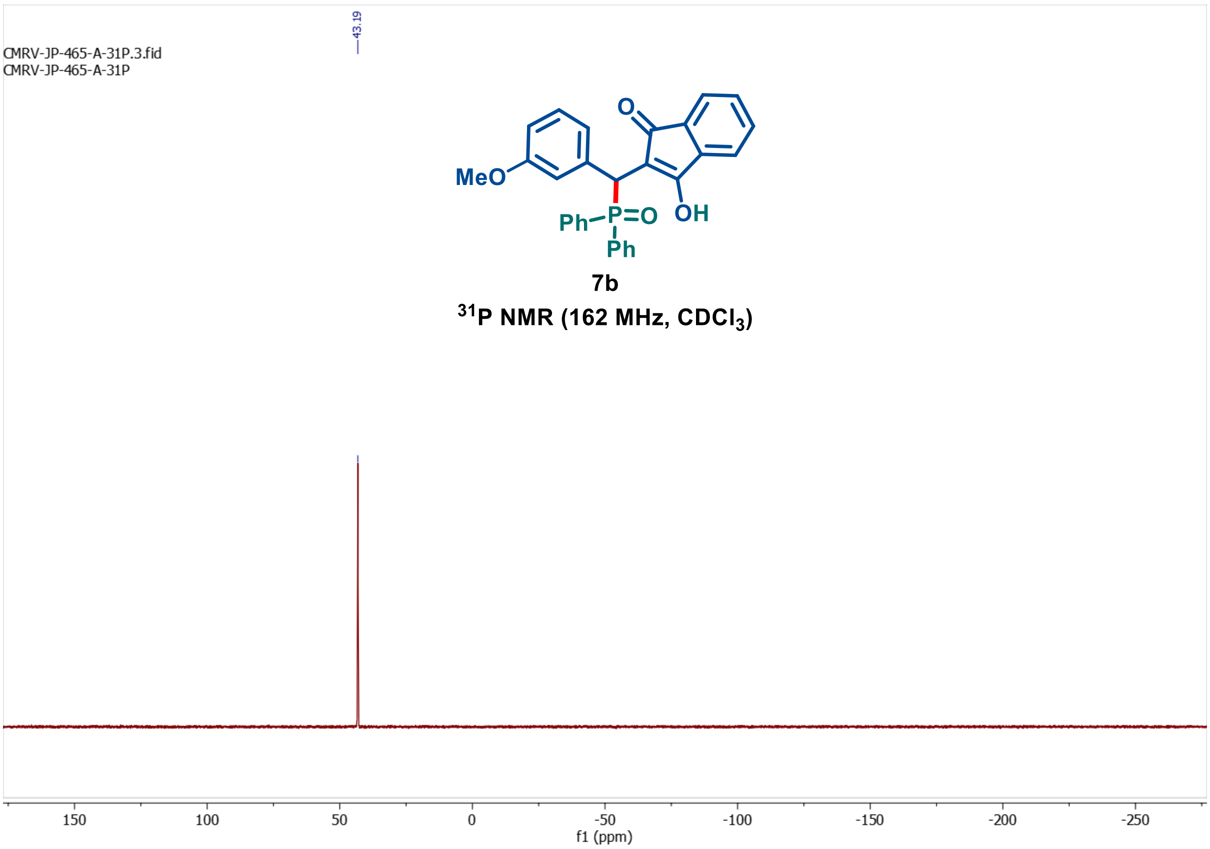
CMRV-JP-465-A-31P.3.fid
CMRV-JP-465-A-31P
43.19
MeO
O
OH
Ph
P=O
Ph
7b
^{31}P NMR (162 MHz, $CDCl_3$)
150
100
50
0
-50
-100
-150
-200
-250
f1 (ppm)

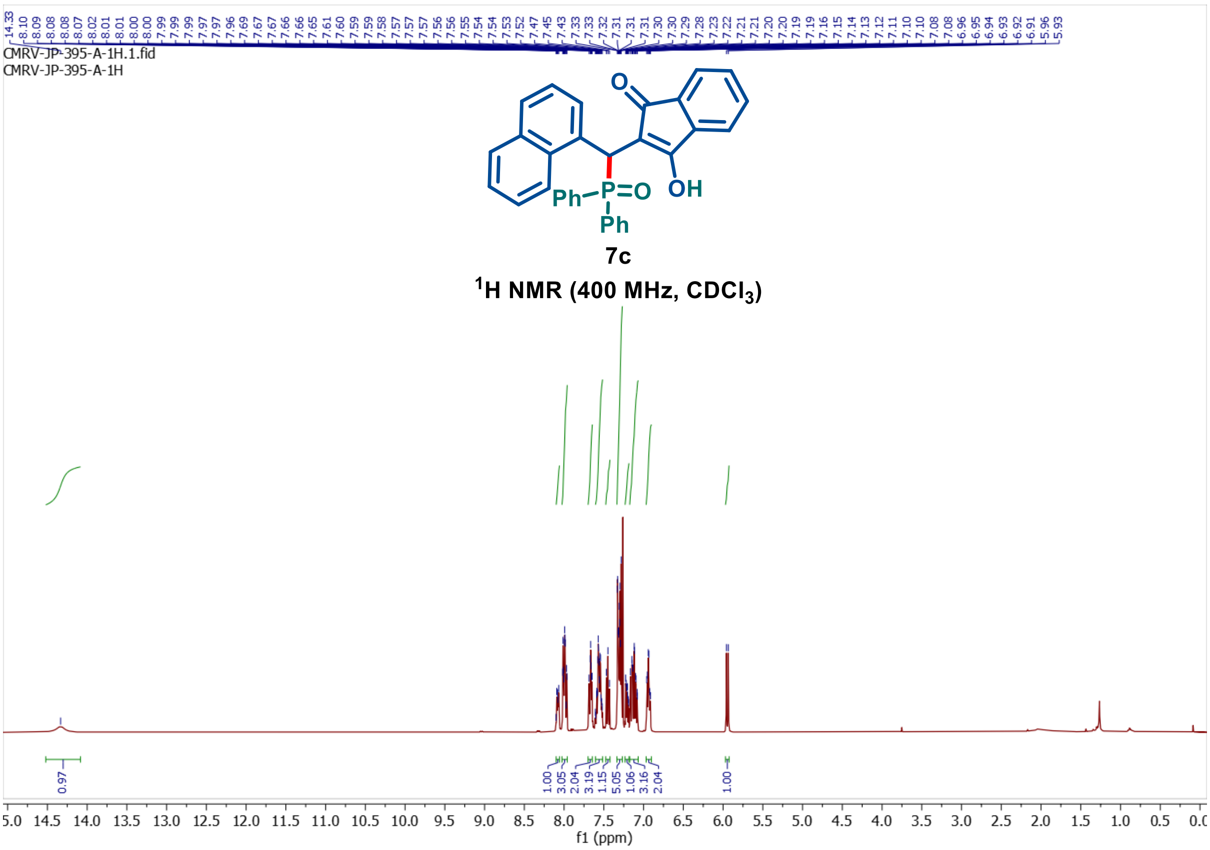
14.33 8.10 8.09 8.08 8.07 8.02 8.01 8.00 7.99 7.99 7.97 7.97 7.96 7.69 7.67 7.66 7.65 7.61 7.60 7.59 7.59 7.58 7.57 7.57 7.56 7.55 7.54 7.54 7.53 7.52 7.47 7.45 7.43 7.33 7.33 7.32 7.31 7.31 7.31 7.30 7.30 7.29 7.28 7.23 7.22 7.21 7.21 7.21 7.20 7.20 7.19 7.19 7.16 7.15 7.14 7.13 7.12 7.11 7.10 7.10 7.08 6.96 6.95 6.94 6.93 6.92 6.91 5.96 5.93
CMRV-JP-395-A-1H.1.fid
CMRV-JP-395-A-1H
O
OH
Ph
P=O
Ph
7c
^{1}H NMR (400 MHz, $CDCl_3$)
0.97
1.00
3.05
2.04
3.19
1.15
5.05
1.06
3.16
2.04
1.00
f1 (ppm)

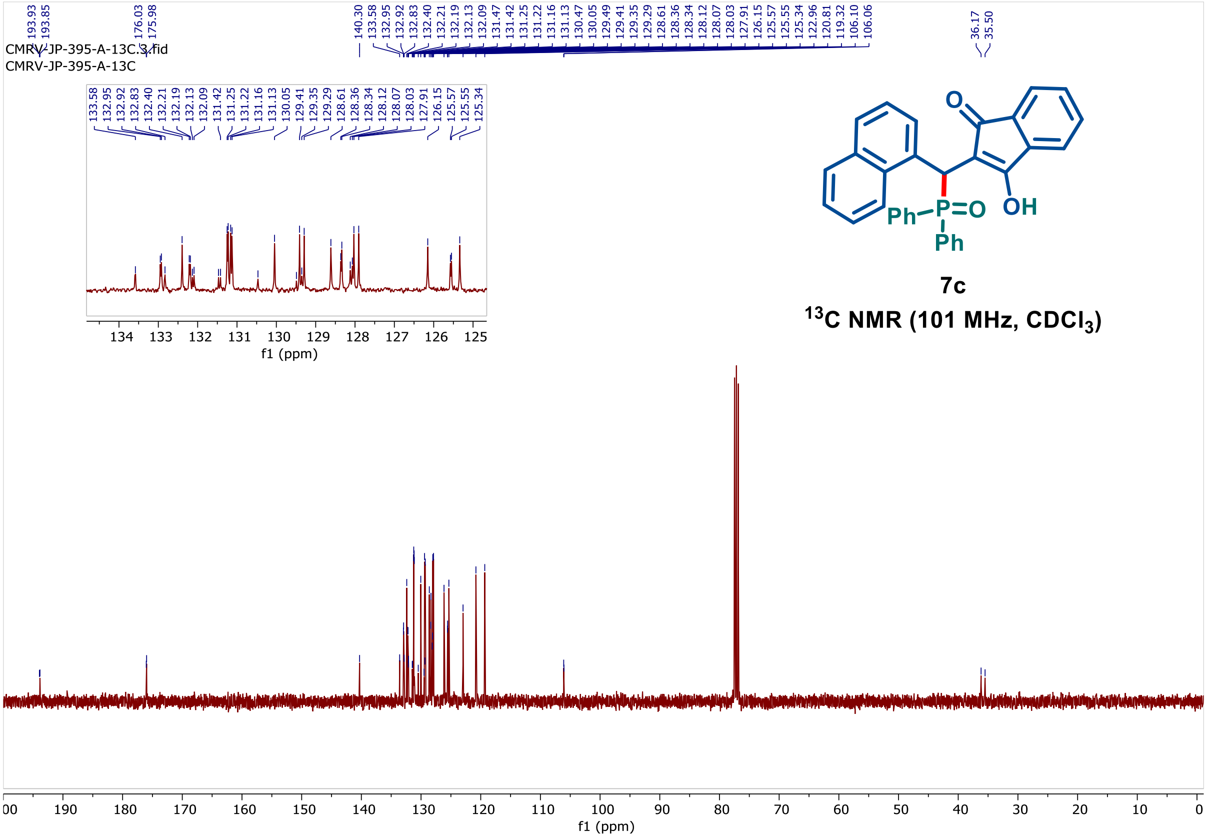
CMRV-JP-395-A-13C.3.fid
CMRV-JP-395-A-13C
193.93
193.85
176.03
175.98
140.30
133.58
132.95
132.92
132.83
132.40
132.21
132.19
132.13
132.09
131.47
131.42
131.25
131.22
131.16
131.13
130.47
130.05
129.49
129.41
129.35
129.29
128.61
128.36
128.34
128.12
128.07
128.03
127.91
126.15
125.57
125.55
125.34
122.96
120.81
119.32
106.10
106.06
36.17
35.50
O
OH
Ph
P=O
Ph
7c
13C NMR (101 MHz, CDCl3)
f1 (ppm)

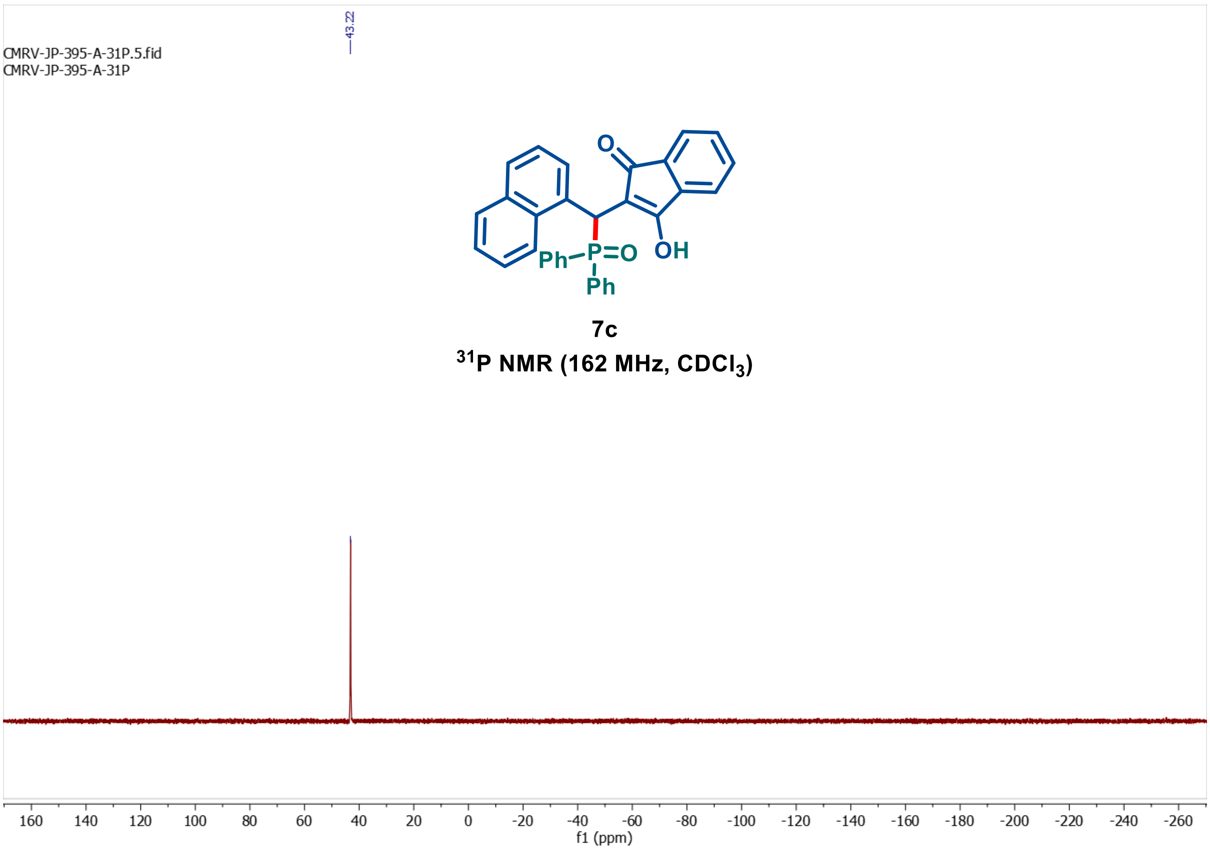
CMRV-JP-395-A-31P.5.fid
CMRV-JP-395-A-31P
43.22
O
OH
Ph
P=O
Ph
7c
31P NMR (162 MHz, CDCl3)
f1 (ppm)

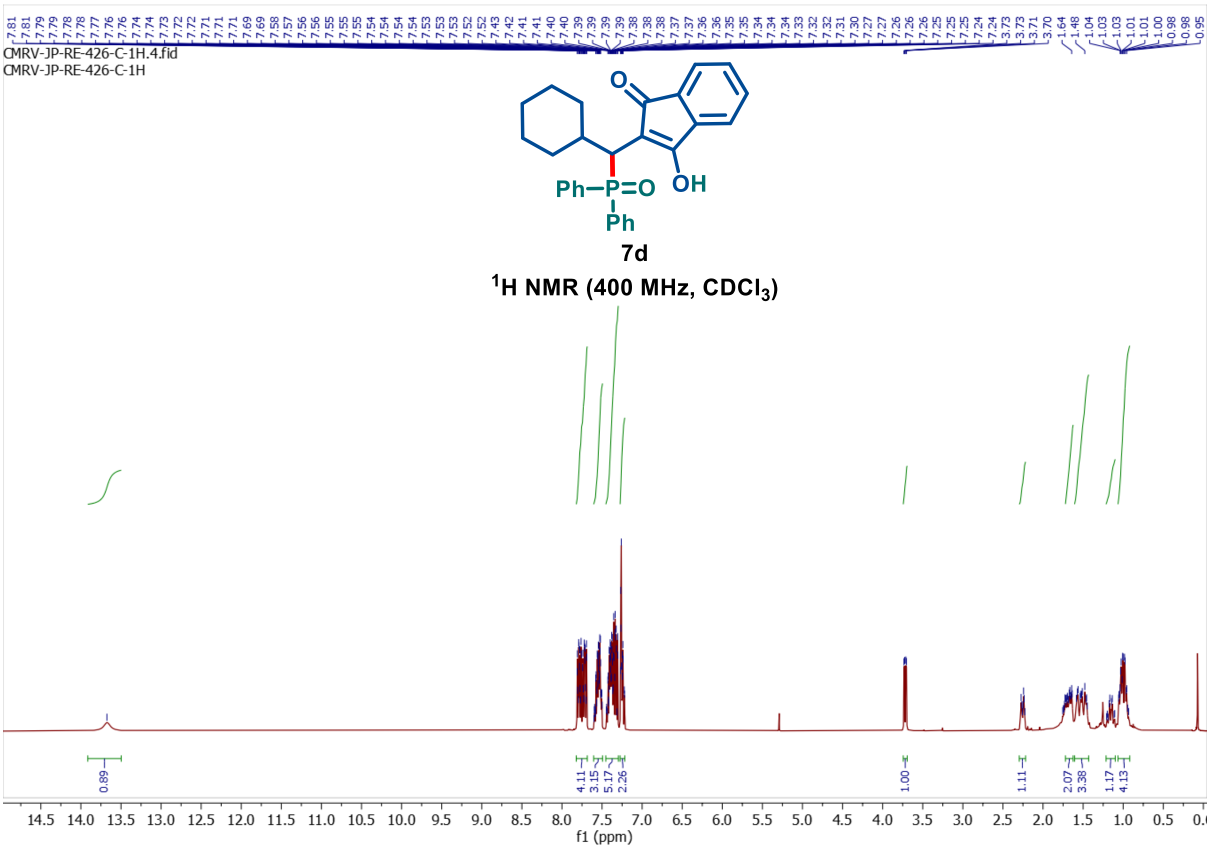

CMRV-JP-RE-426-C-1H.4.fid
CMRV-JP-RE-426-C-1H
O
OH
Ph–P=O
Ph
7d
1H NMR (400 MHz, CDCl3)
f1 (ppm)

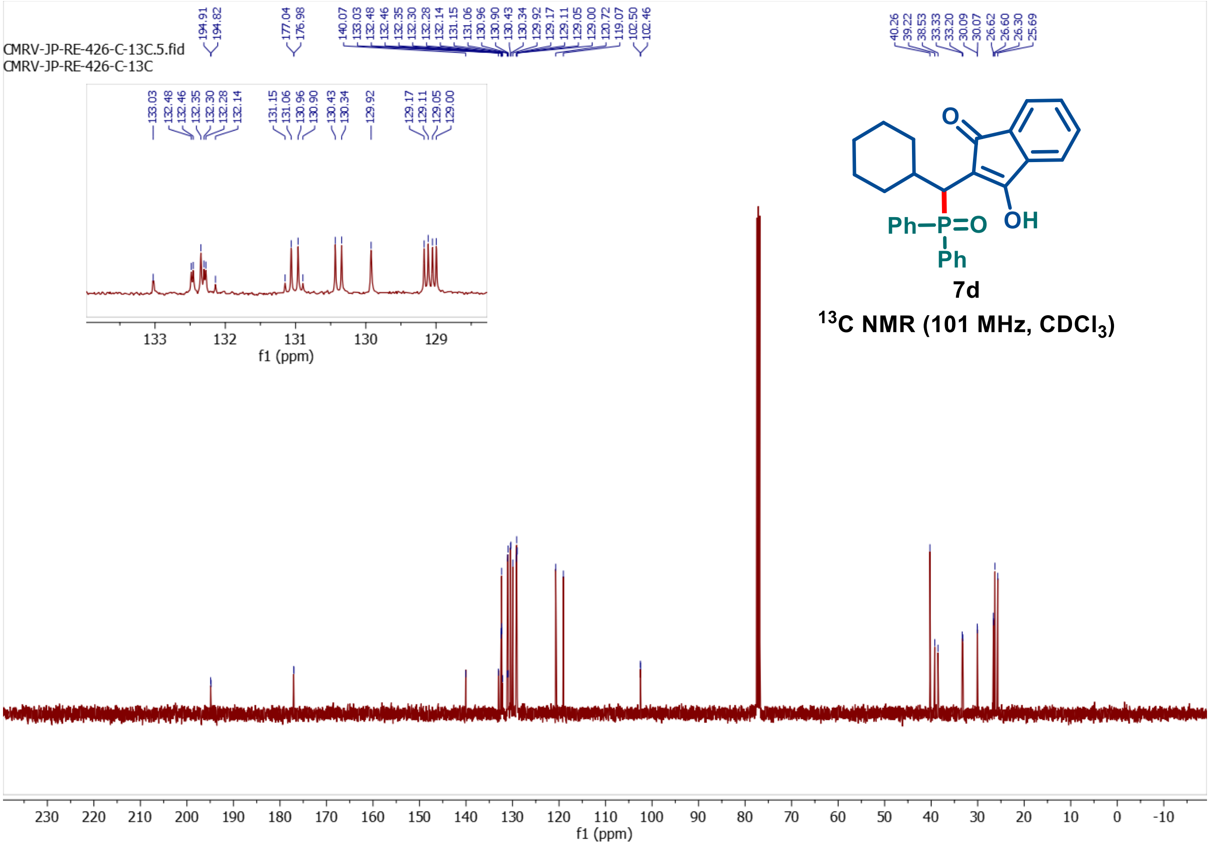

CMRV-JP-RE-426-C-13C.5.fid
CMRV-JP-RE-426-C-13C
O
OH
Ph–P=O
Ph
7d
13C NMR (101 MHz, CDCl3)
f1 (ppm)

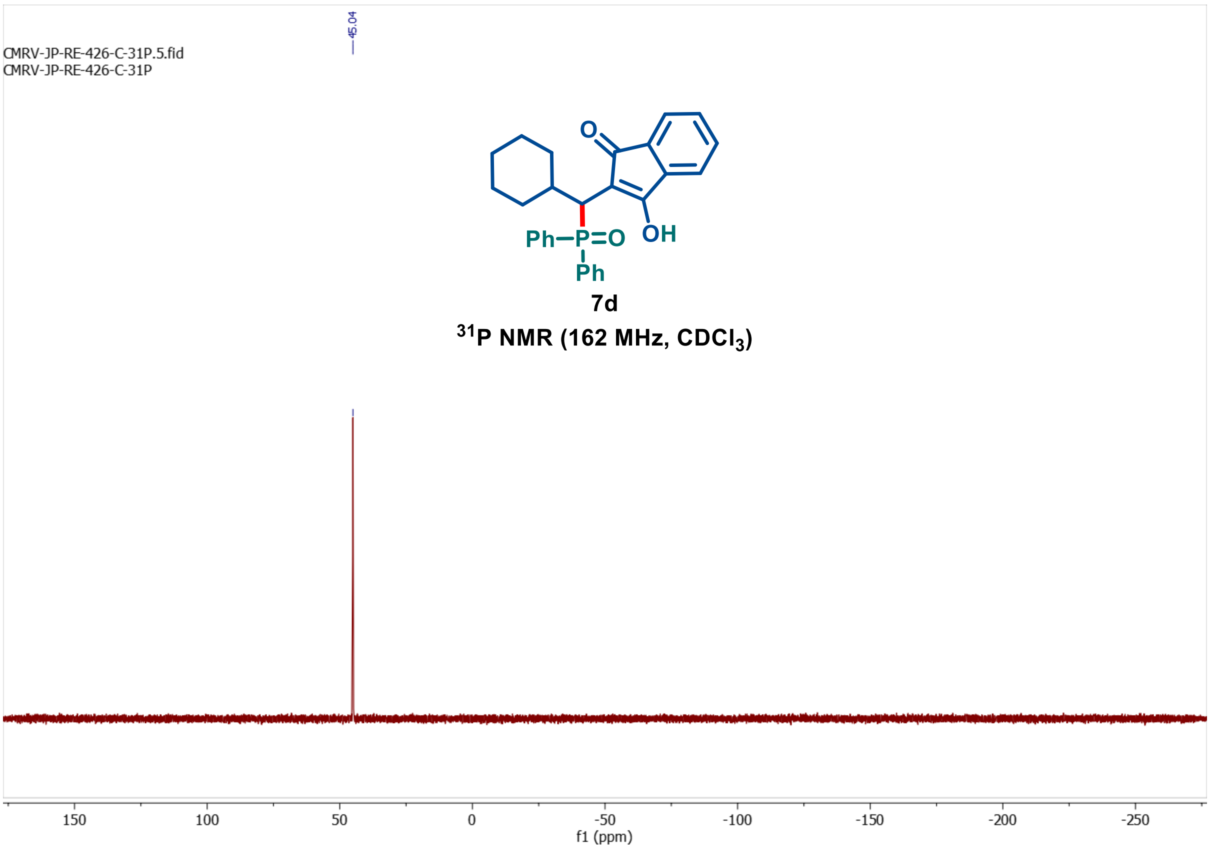
CMRV-JP-RE-426-C-31P.5.fid
CMRV-JP-RE-426-C-31P
45.04
O
OH
Ph–P=O
Ph
7d
31P NMR (162 MHz, CDCl3)
150 100 50 0 -50 -100 -150 -200 -250
f1 (ppm)

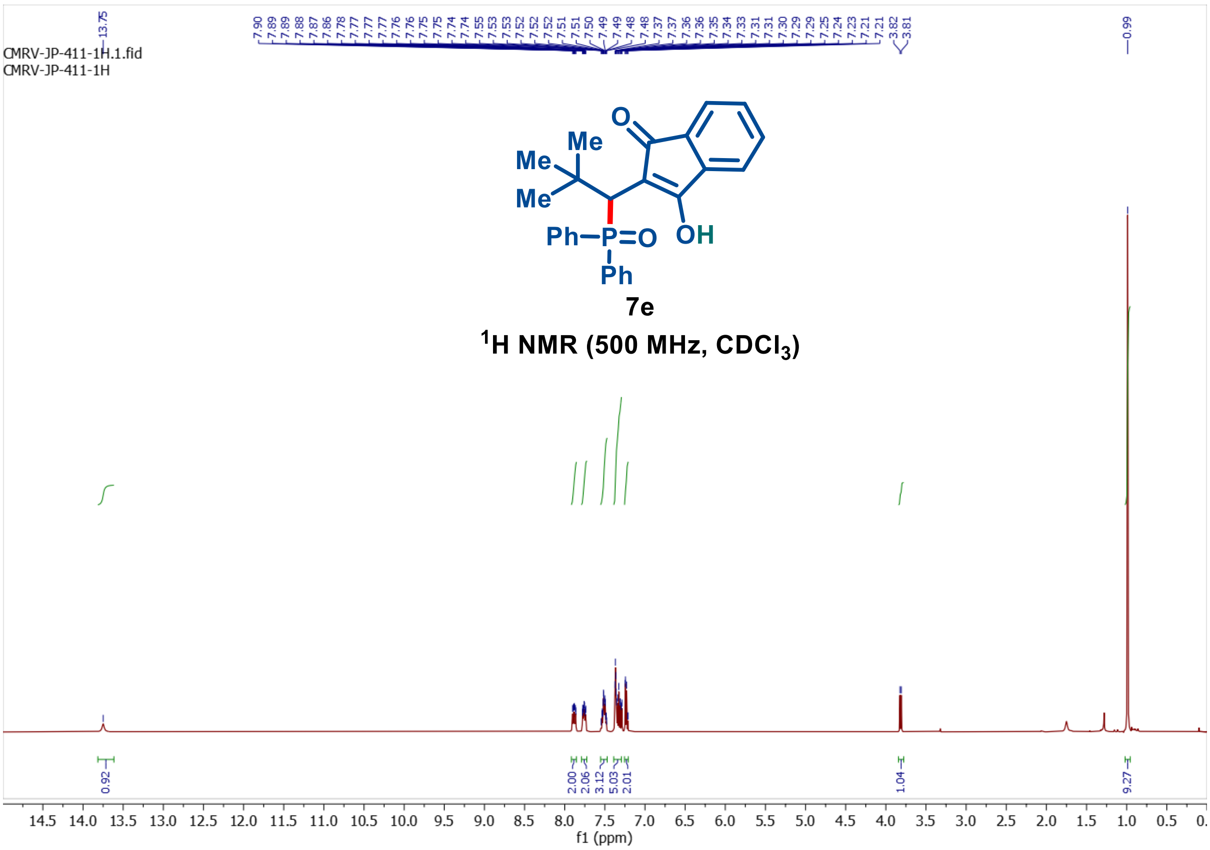
CMRV-JP-411-1H.1.fid
CMRV-JP-411-1H
13.75
7.90 7.89 7.89 7.88 7.87 7.86 7.78 7.77 7.77 7.76 7.76 7.75 7.75 7.74 7.74 7.55 7.53 7.53 7.52 7.52 7.52 7.51 7.51 7.50 7.49 7.49 7.48 7.48 7.37 7.37 7.36 7.36 7.35 7.34 7.33 7.31 7.31 7.30 7.29 7.29 7.25 7.24 7.23 7.21 7.21
3.82 3.81
0.99
O
Me
Me
Me
OH
Ph–P=O
Ph
7e
1H NMR (500 MHz, CDCl3)
0.92
2.00 2.06 3.12 5.03 2.01
1.04
9.27
14.5 14.0 13.5 13.0 12.5 12.0 11.5 11.0 10.5 10.0 9.5 9.0 8.5 8.0 7.5 7.0 6.5 6.0 5.5 5.0 4.5 4.0 3.5 3.0 2.5 2.0 1.5 1.0 0.5 0.
f1 (ppm)

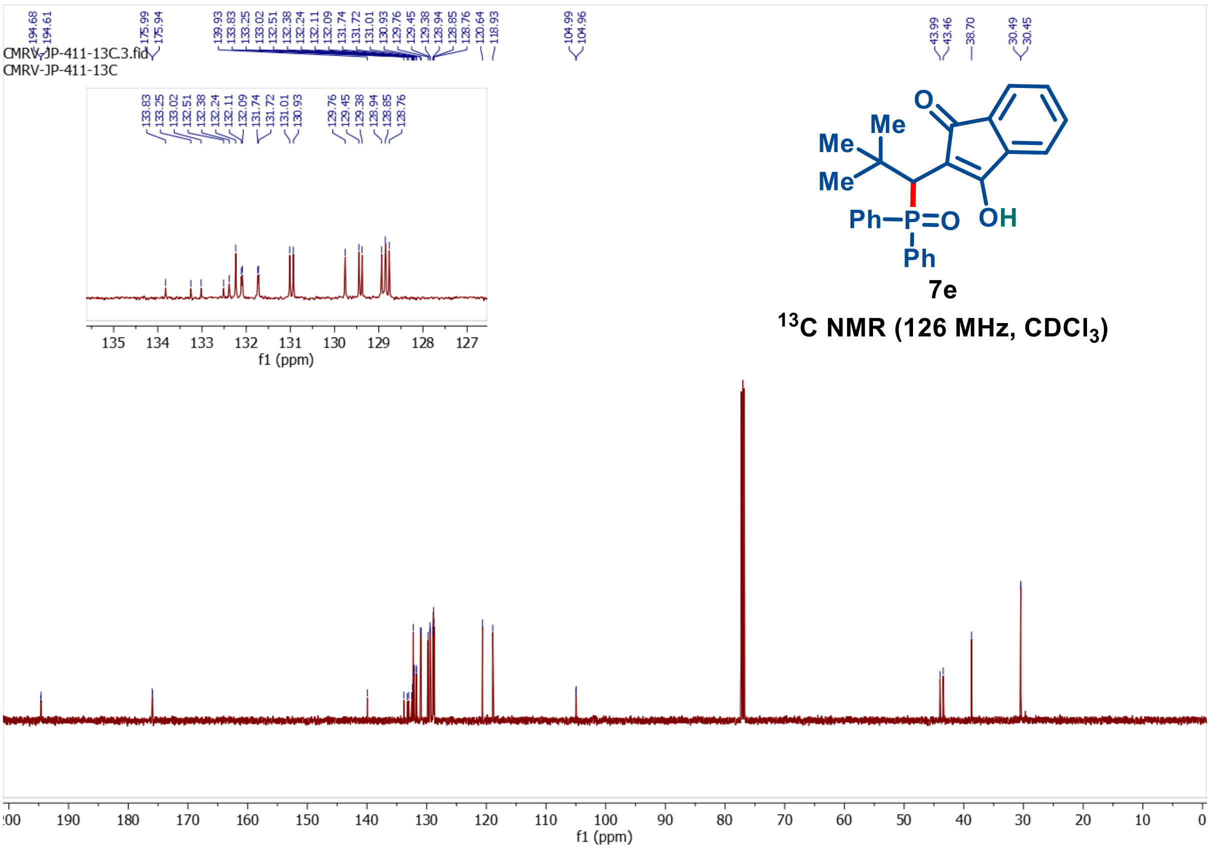

7e

^{13}C NMR (126 MHz, $CDCl_3$)

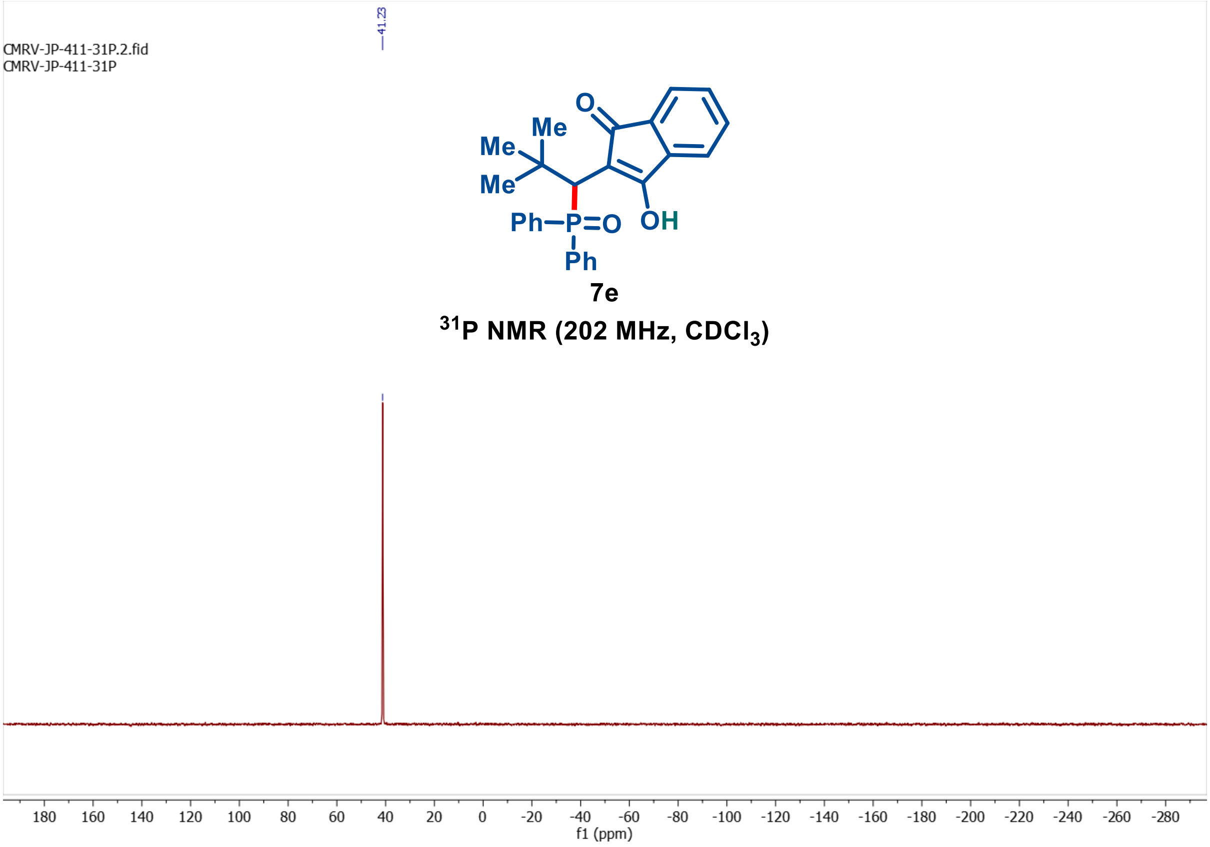

7e

^{31}P NMR (202 MHz, $CDCl_3$)

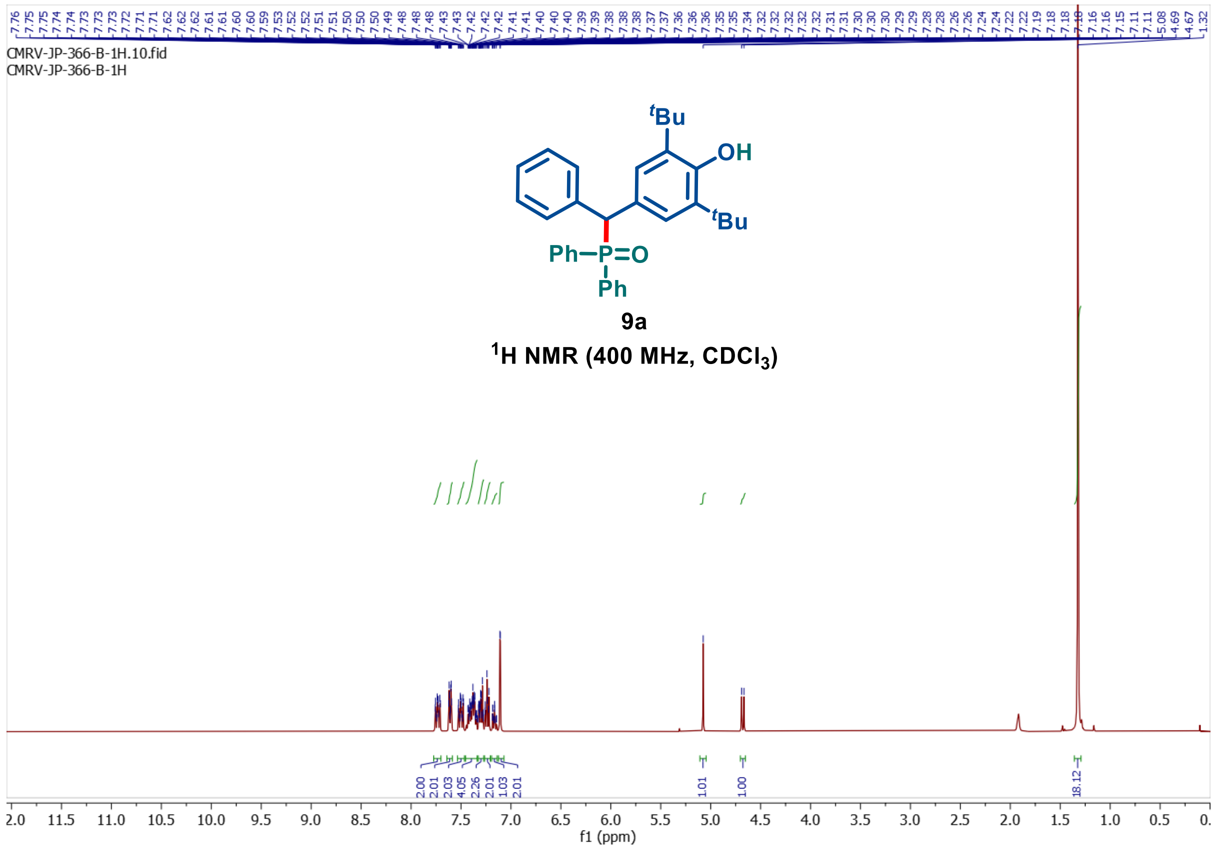
CMRV-JP-366-B-1H.10.fid
CMRV-JP-366-B-1H
tBu
OH
tBu
Ph—P=O
Ph
9a
1H NMR (400 MHz, CDCl3)
f1 (ppm)

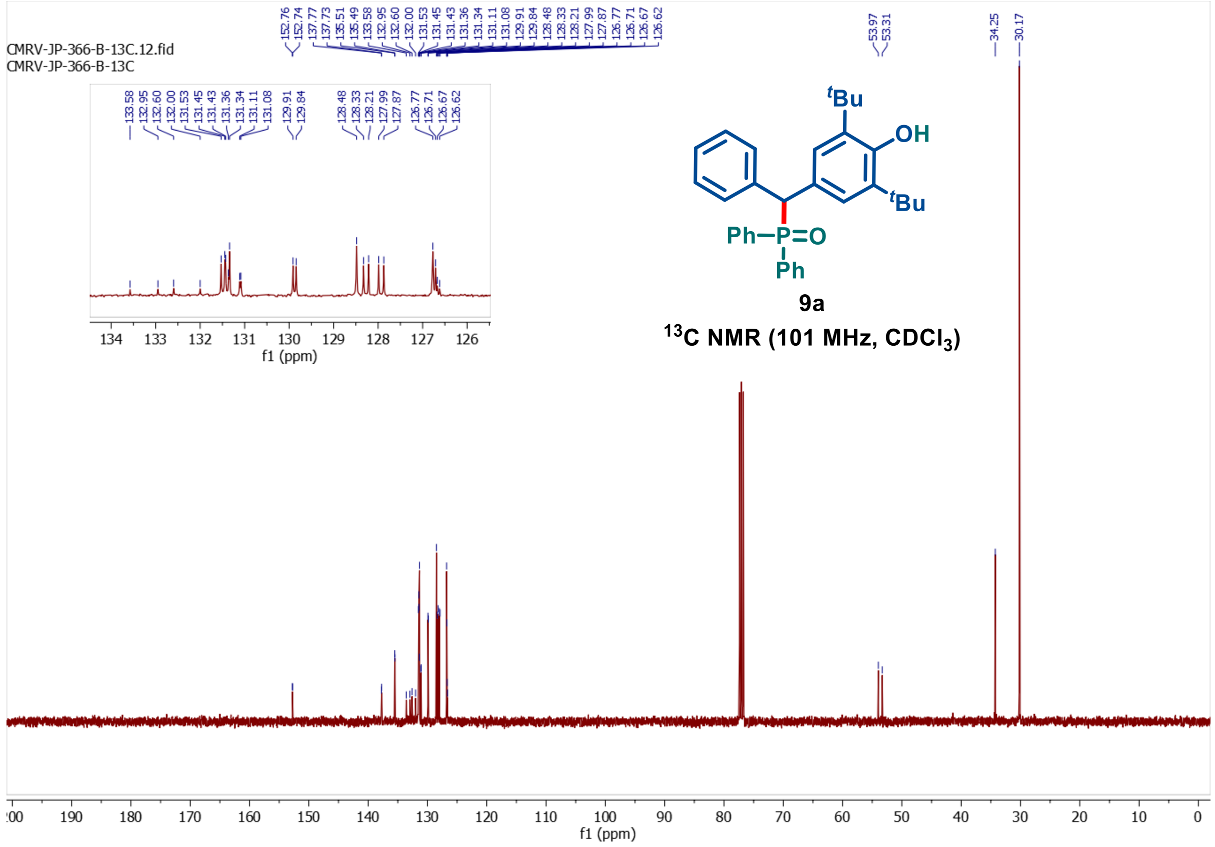
CMRV-JP-366-B-13C.12.fid
CMRV-JP-366-B-13C
tBu
OH
tBu
Ph—P=O
Ph
9a
13C NMR (101 MHz, CDCl3)
f1 (ppm)

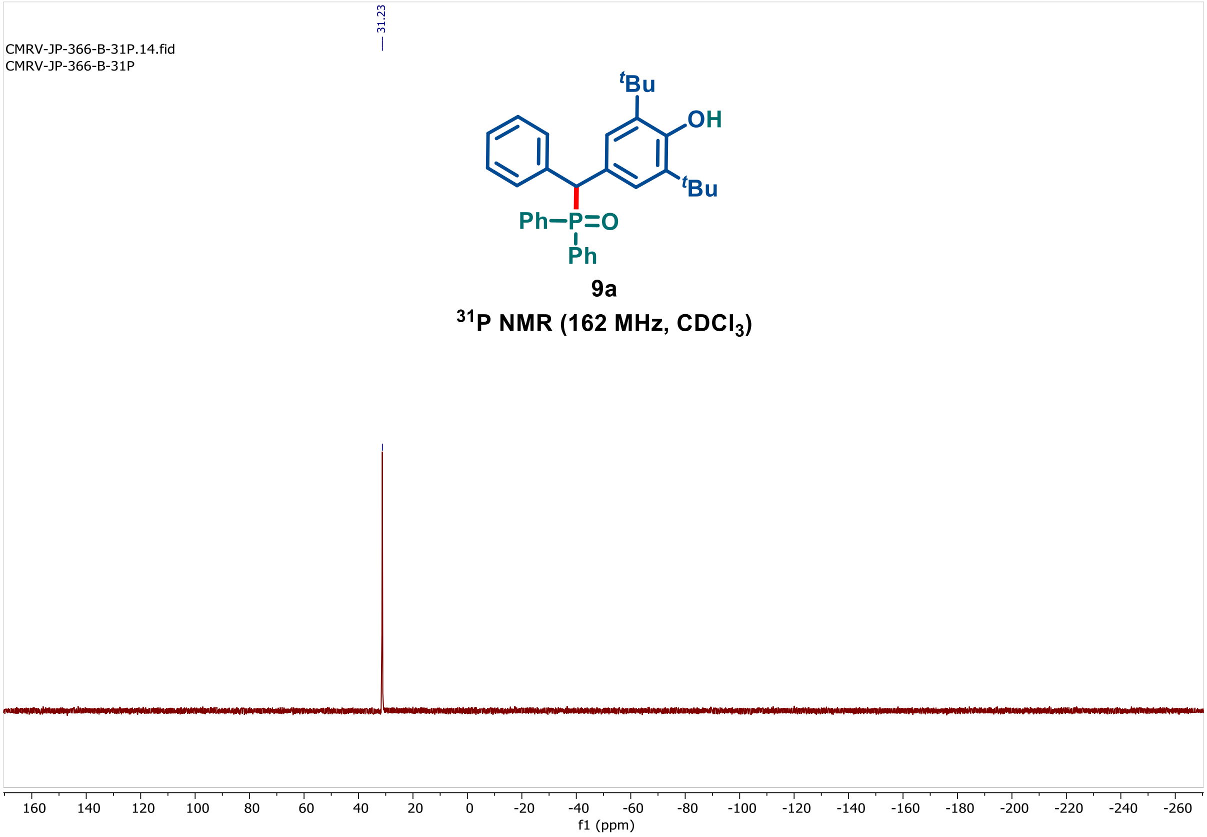

31.23
CMRV-JP-366-B-31P.14.fid
CMRV-JP-366-B-31P
tBu
OH
tBu
Ph–P=O
Ph
9a
31P NMR (162 MHz, CDCl3)
f1 (ppm)

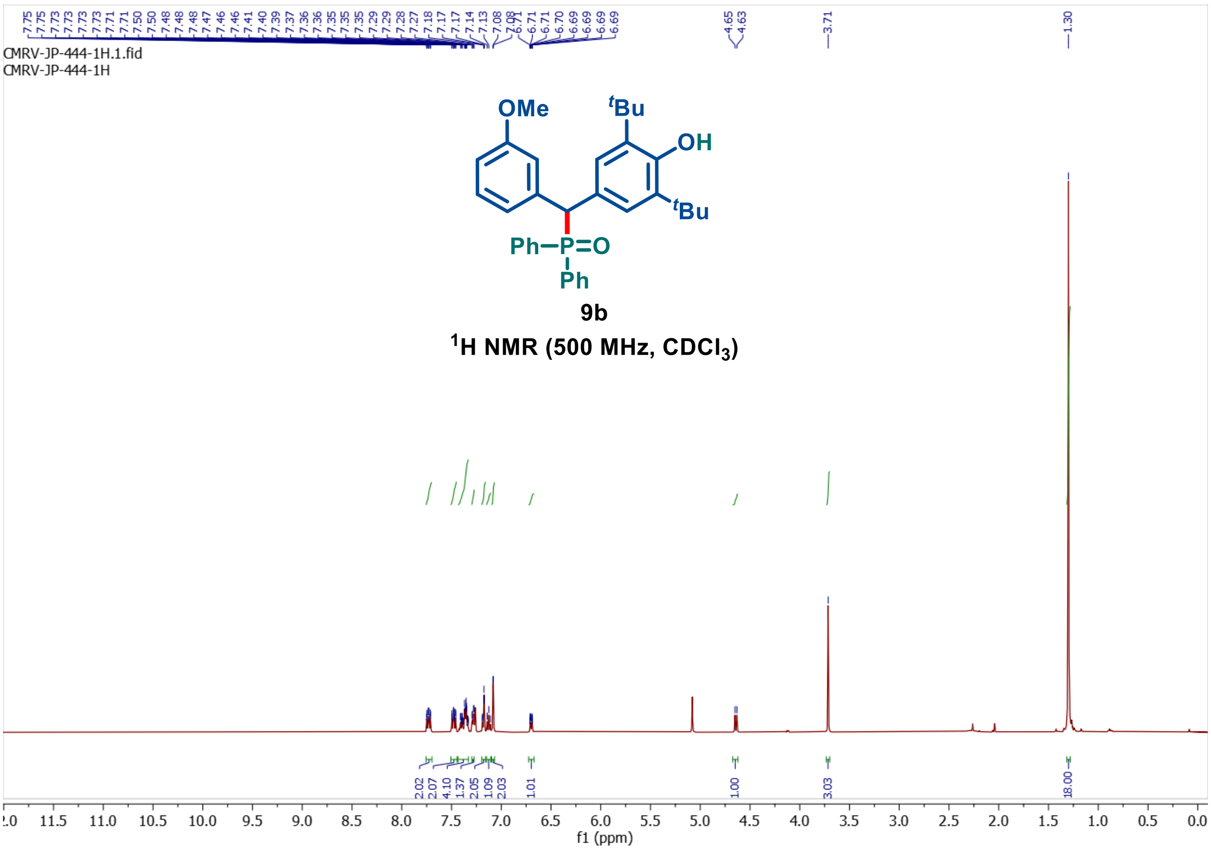

4.65
4.63
3.71
1.30
CMRV-JP-444-1H.1.fid
CMRV-JP-444-1H
OMe
tBu
OH
tBu
Ph–P=O
Ph
9b
1H NMR (500 MHz, CDCl3)
f1 (ppm)

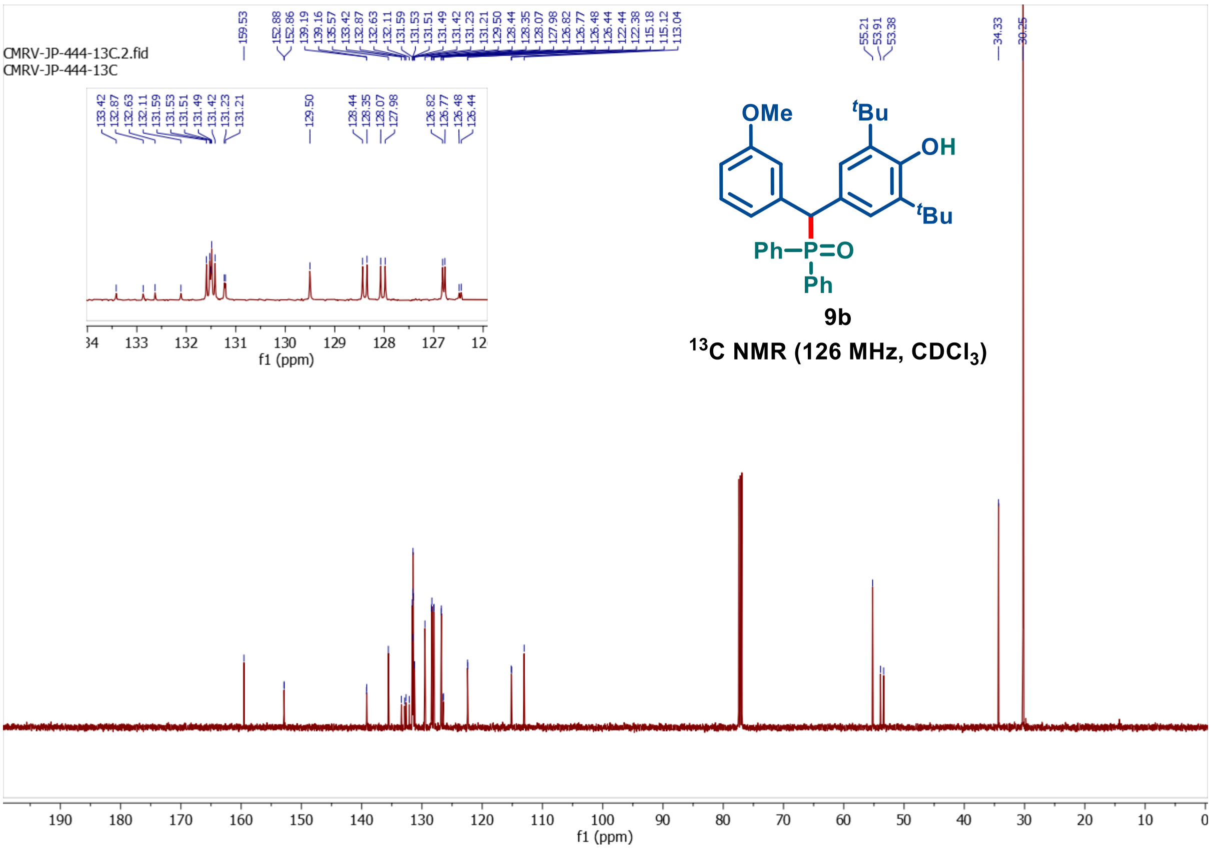
CMRV-JP-444-13C.2.fid
CMRV-JP-444-13C
159.53
152.88
152.86
139.19
139.16
135.57
133.42
132.87
132.63
132.11
131.59
131.53
131.51
131.49
131.42
131.23
131.21
129.50
128.44
128.35
128.07
127.98
126.82
126.77
126.48
126.44
122.44
122.38
115.18
115.12
113.04
55.21
53.91
53.38
34.33
30.25
OMe
tBu
OH
tBu
Ph—P=O
Ph
9b
13C NMR (126 MHz, CDCl3)
f1 (ppm)

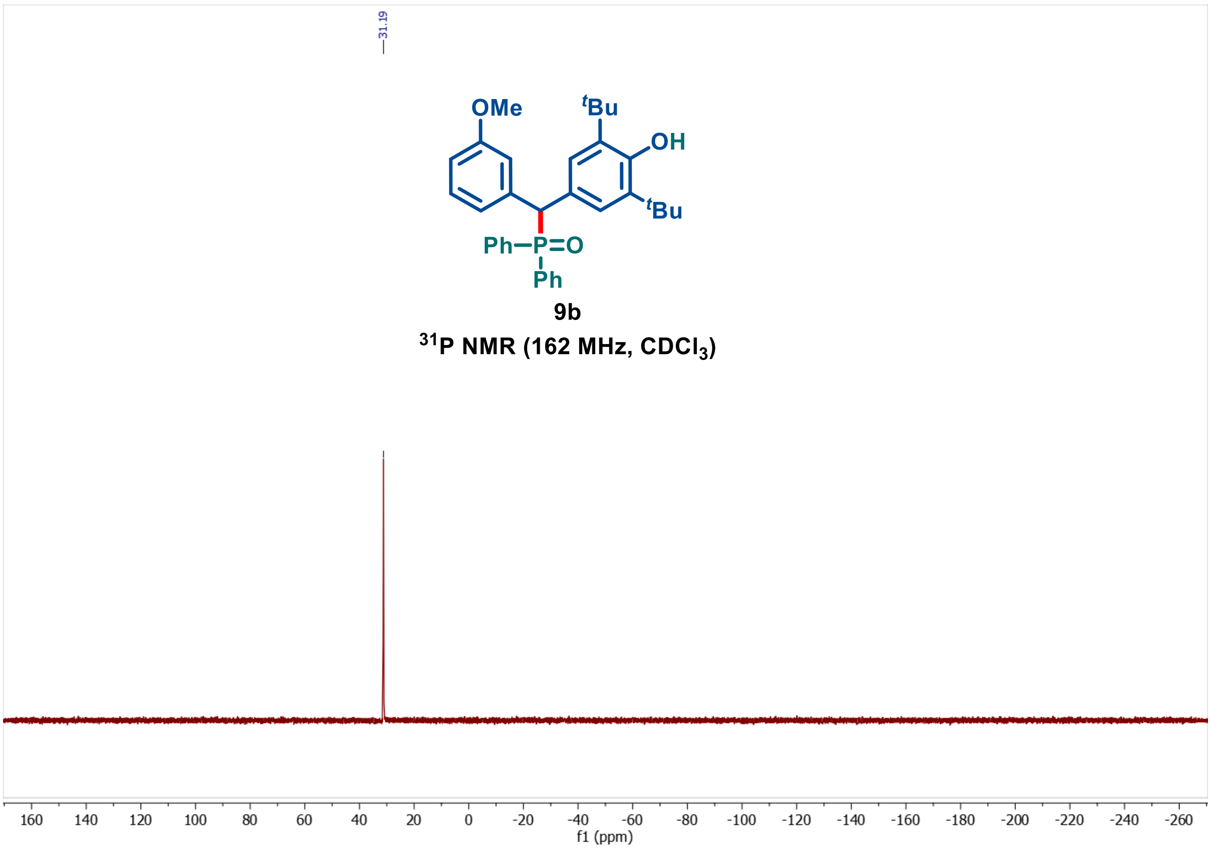
31.19
OMe
tBu
OH
tBu
Ph—P=O
Ph
9b
31P NMR (162 MHz, CDCl3)
f1 (ppm)

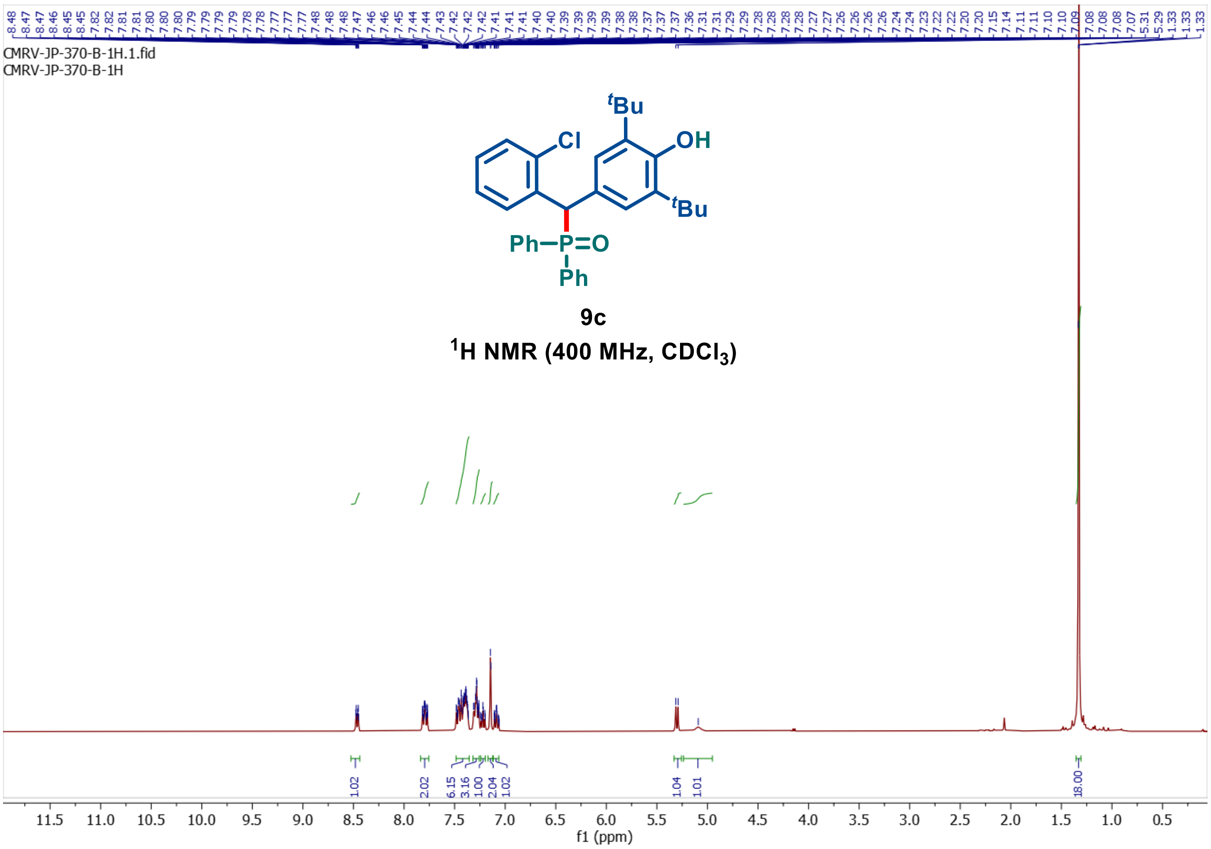
CMRV-JP-370-B-1H.1.fid
CMRV-JP-370-B-1H
9c
1H NMR (400 MHz, CDCl3)
f1 (ppm)

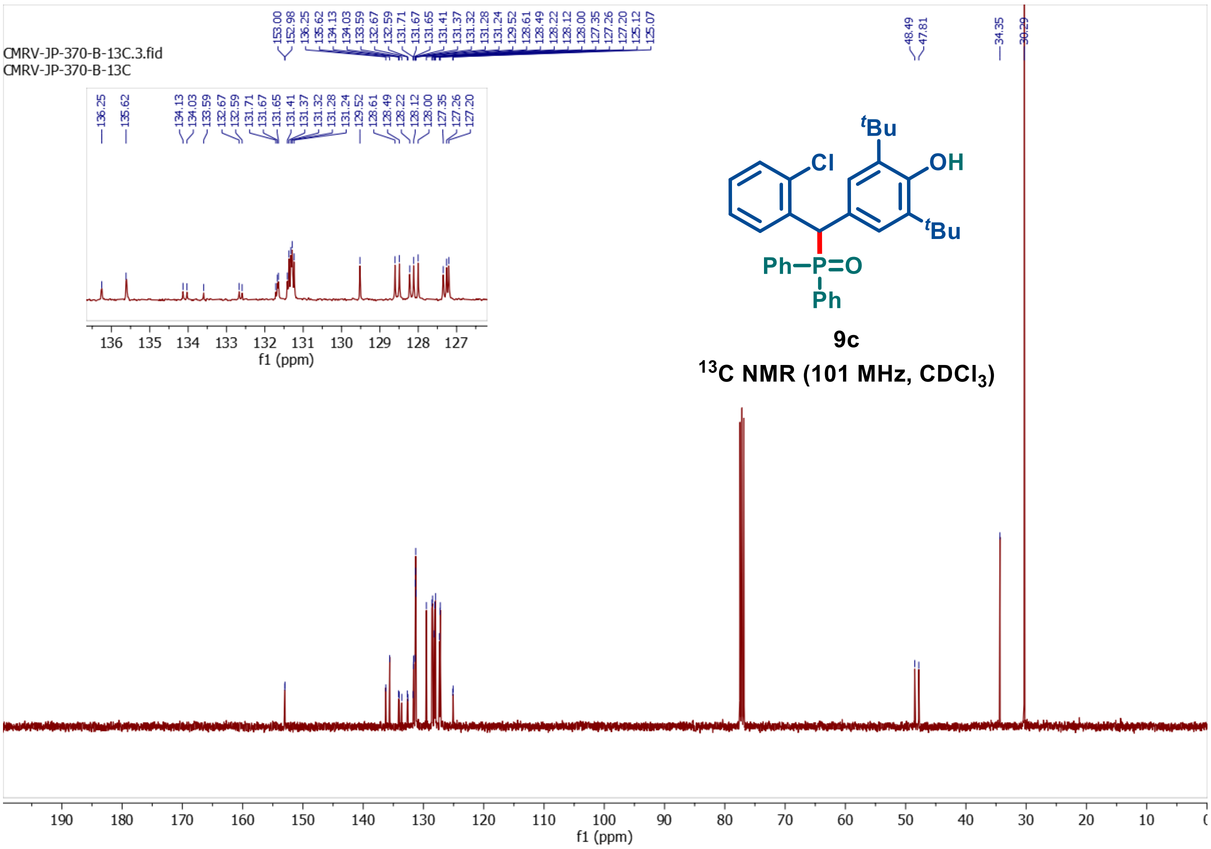
CMRV-JP-370-B-13C.3.fid
CMRV-JP-370-B-13C
9c
13C NMR (101 MHz, CDCl3)
f1 (ppm)

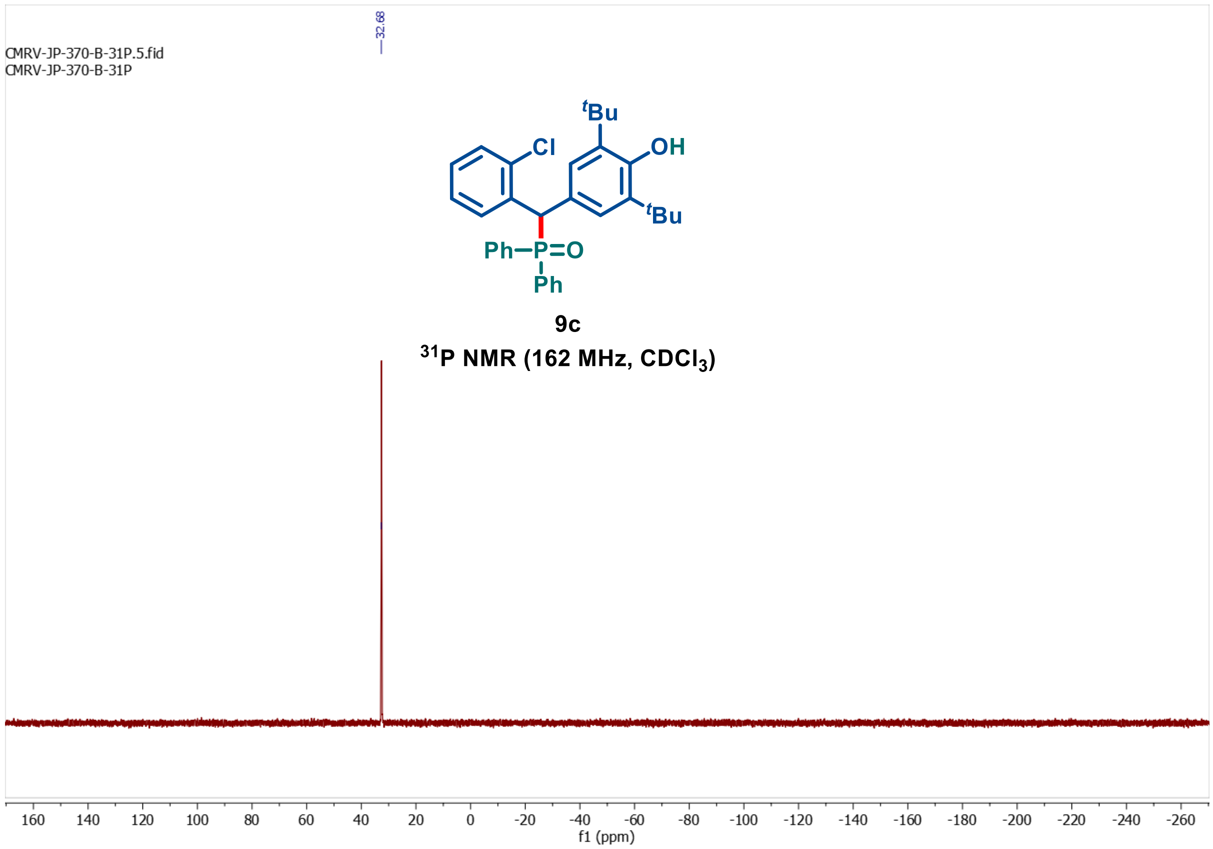
CMRV-JP-370-B-31P.5.fid
CMRV-JP-370-B-31P
32.68
tBu
Cl
OH
tBu
Ph–P=O
Ph
9c
31P NMR (162 MHz, CDCl3)
f1 (ppm)

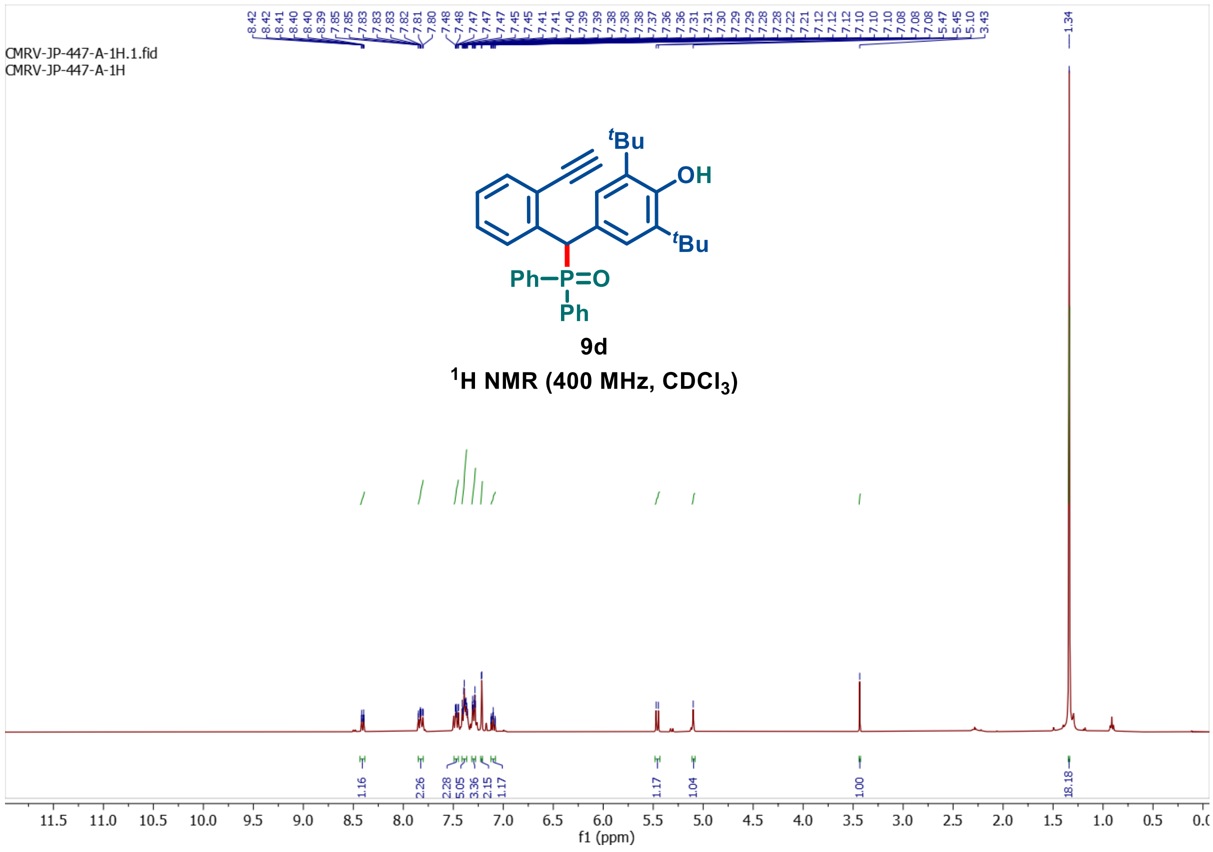
CMRV-JP-447-A-1H.1.fid
CMRV-JP-447-A-1H
1.34
tBu
OH
tBu
Ph–P=O
Ph
9d
1H NMR (400 MHz, CDCl3)
1.16
2.26
2.28
5.05
3.36
2.15
1.17
1.17
1.04
1.00
18.18
f1 (ppm)

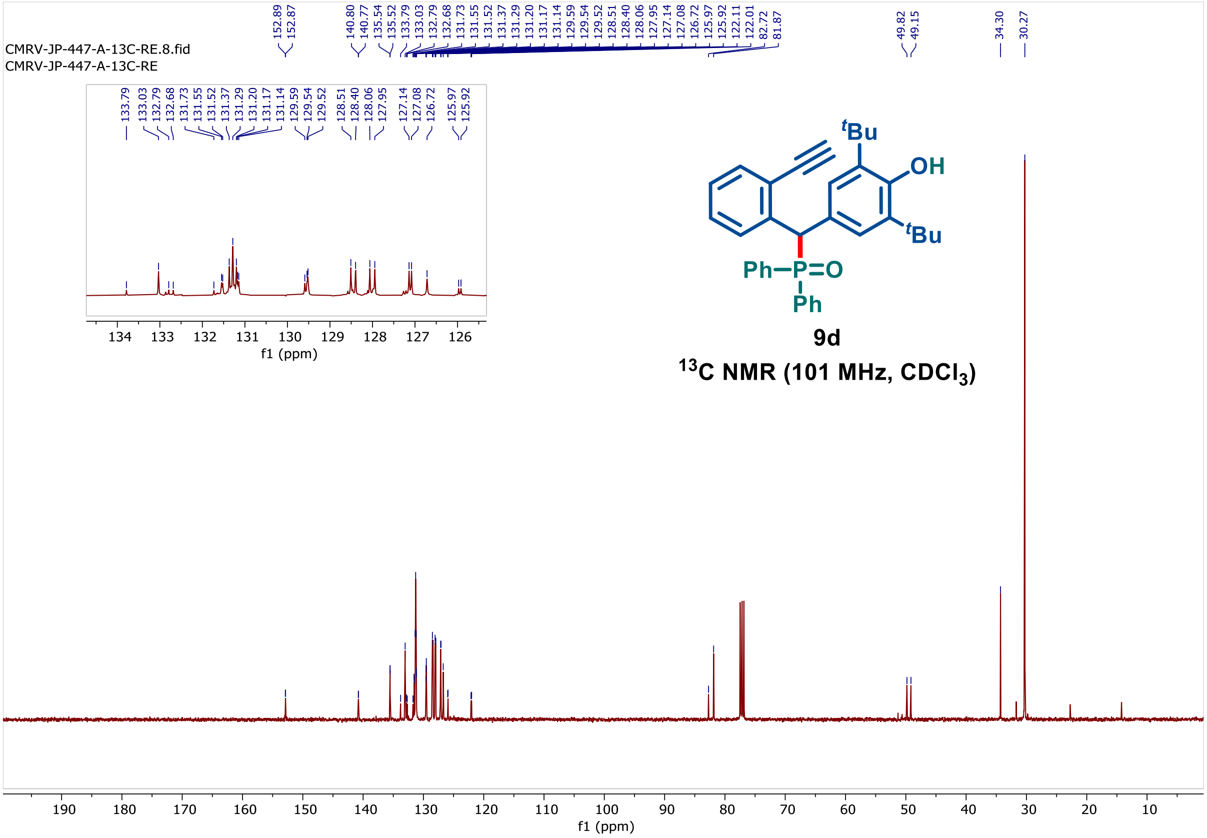
CMRV-JP-447-A-13C-RE.8.fid
CMRV-JP-447-A-13C-RE
152.89
152.87
140.80
140.77
135.54
135.52
133.79
133.03
132.79
132.68
131.73
131.55
131.52
131.37
131.29
131.20
131.17
131.14
129.59
129.54
129.52
128.51
128.40
128.06
127.95
127.14
127.08
126.72
125.97
125.92
122.11
122.01
82.72
81.87
49.82
49.15
34.30
30.27
tBu
OH
tBu
Ph–P=O
Ph
9d
13C NMR (101 MHz, CDCl3)
f1 (ppm)

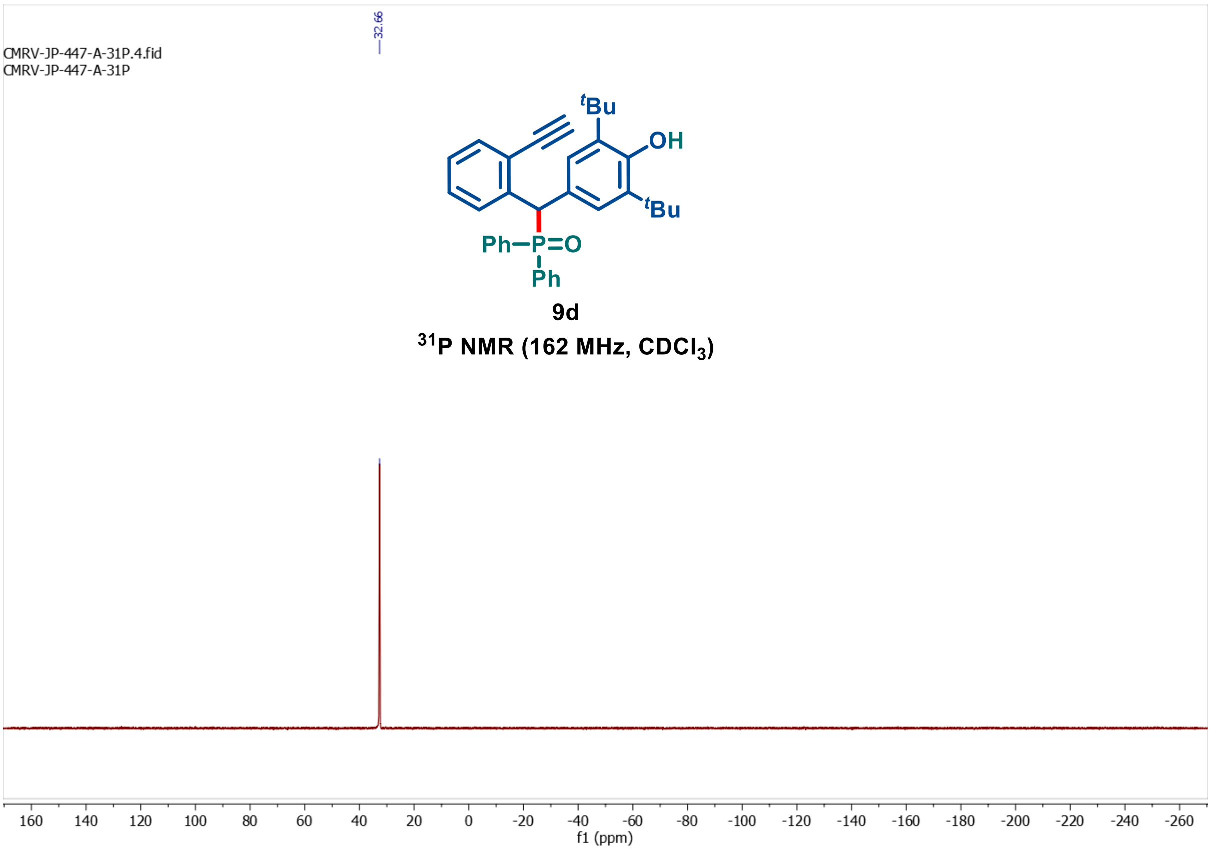
CMRV-JP-447-A-31P.4.fid
CMRV-JP-447-A-31P
32.66
tBu
OH
tBu
Ph–P=O
Ph
9d
31P NMR (162 MHz, CDCl3)
f1 (ppm)

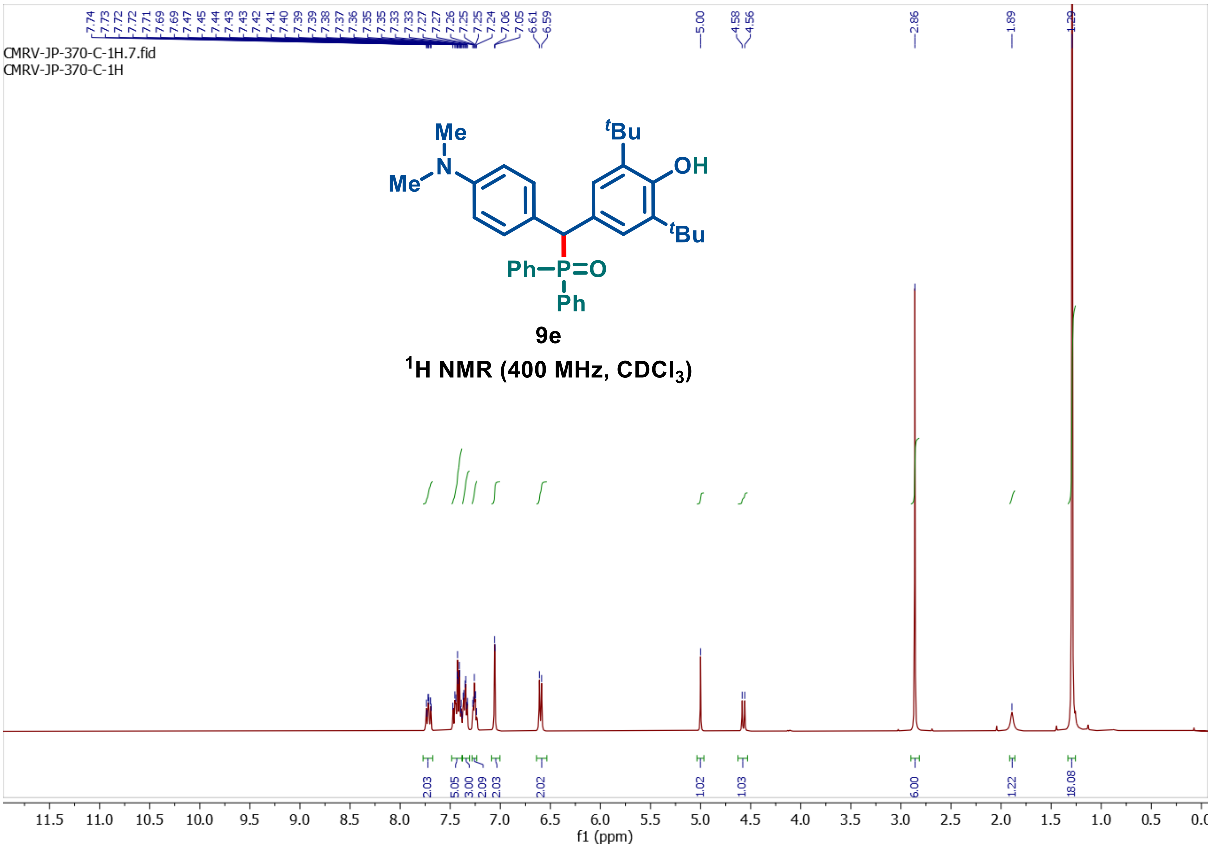
CMRV-JP-370-C-1H.7.fid
CMRV-JP-370-C-1H
7.74 7.73 7.72 7.72 7.71 7.69 7.47 7.45 7.44 7.43 7.43 7.42 7.41 7.40 7.39 7.39 7.38 7.37 7.36 7.35 7.35 7.33 7.33 7.27 7.26 7.25 7.25 7.24 7.06 7.05 6.61 6.59
5.00
4.58 4.56
2.86
1.89
1.29
Me
N
Me
tBu
OH
tBu
Ph–P=O
Ph
9e
1H NMR (400 MHz, CDCl3)
2.03 5.05 3.00 2.09 2.03 2.02 1.02 1.03 6.00 1.22 18.08
f1 (ppm)

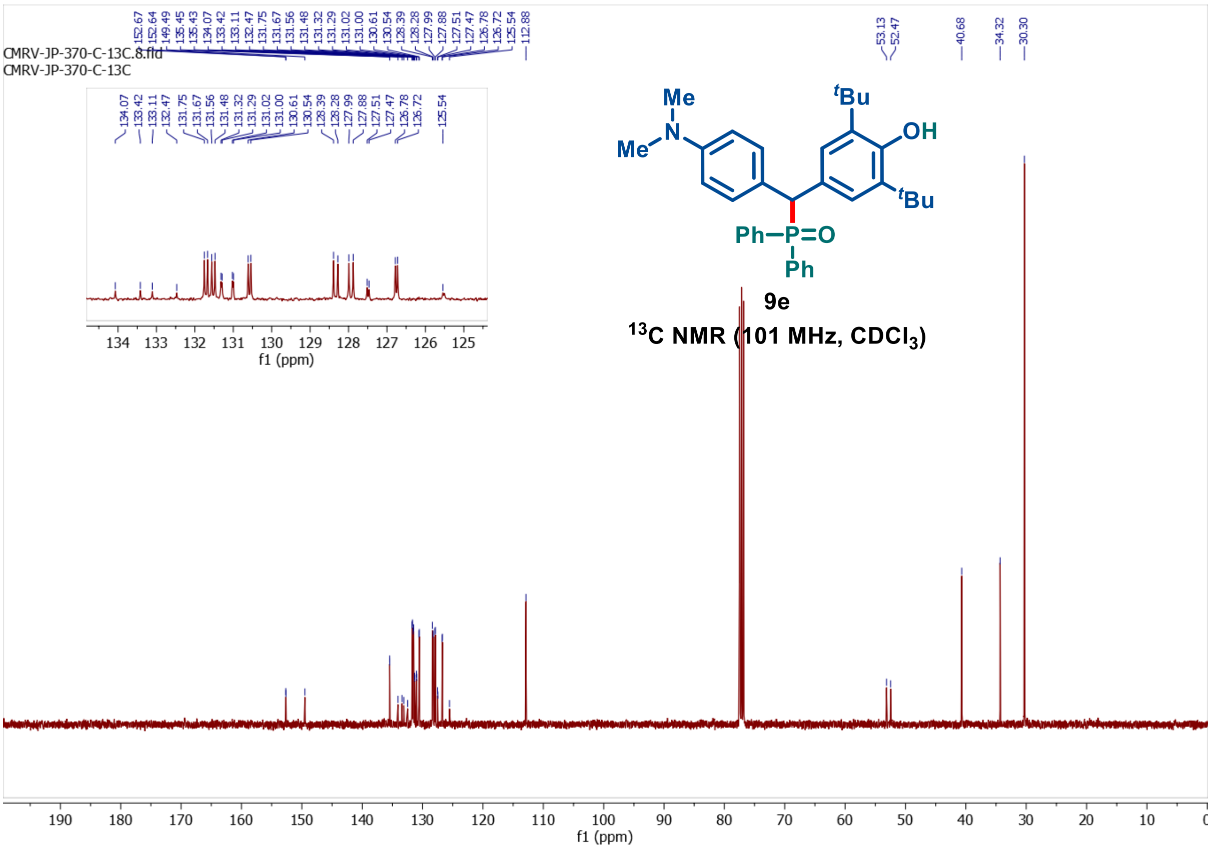
CMRV-JP-370-C-13C.8.fid
CMRV-JP-370-C-13C
152.67 152.64 149.49 135.45 135.43 134.07 133.42 133.11 132.47 131.75 131.67 131.56 131.48 131.32 131.29 131.02 131.00 130.61 130.54 128.39 128.28 127.99 127.88 127.51 127.47 126.78 126.72 125.54 112.88
53.13 52.47
40.68
34.32
30.30
134.07 133.42 133.11 132.47 131.75 131.67 131.56 131.48 131.32 131.29 131.02 131.00 130.61 130.54 128.39 128.28 127.99 127.88 127.51 127.47 126.78 126.72 125.54
f1 (ppm)
Me
N
Me
tBu
OH
tBu
Ph–P=O
Ph
9e
13C NMR (101 MHz, CDCl3)
f1 (ppm)

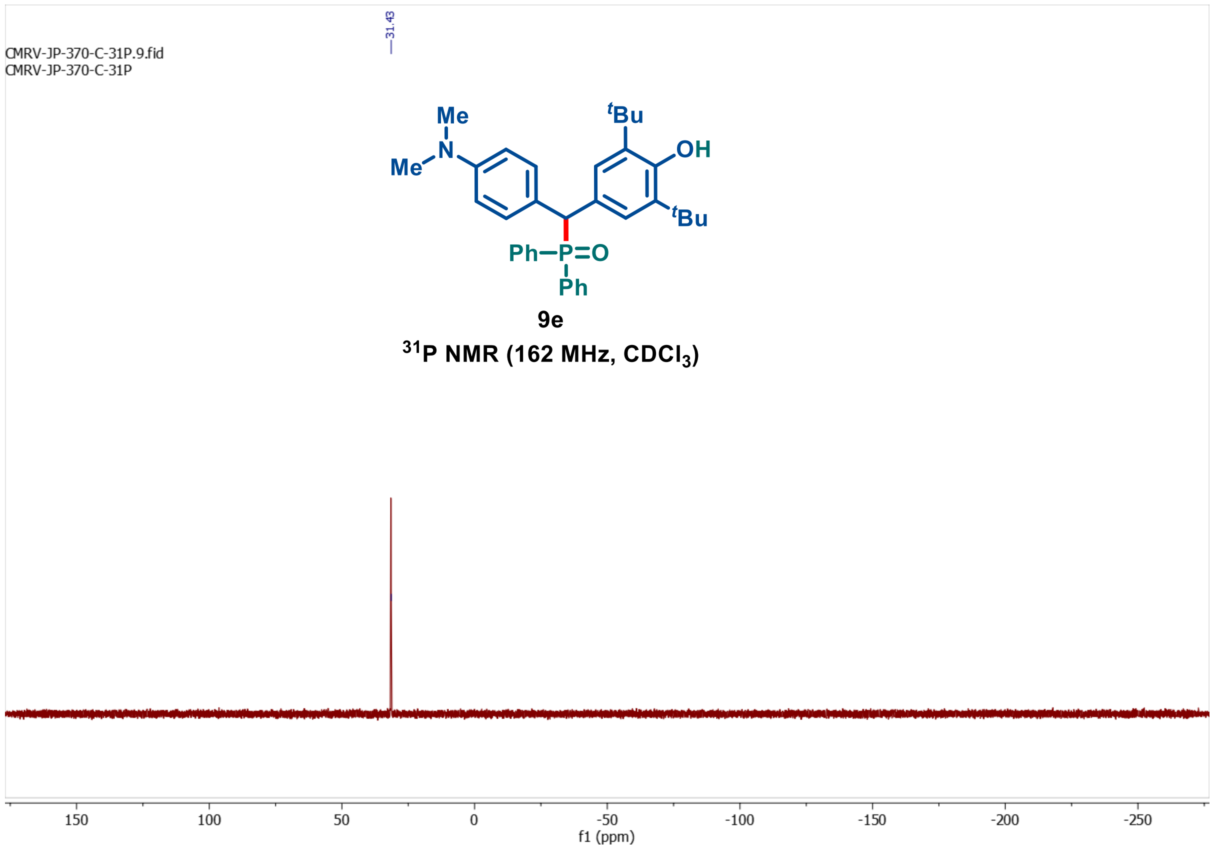
CMRV-JP-370-C-31P.9.fid
CMRV-JP-370-C-31P
31.43
9e
^{31}P NMR (162 MHz, CDCl$_3$)
f1 (ppm)

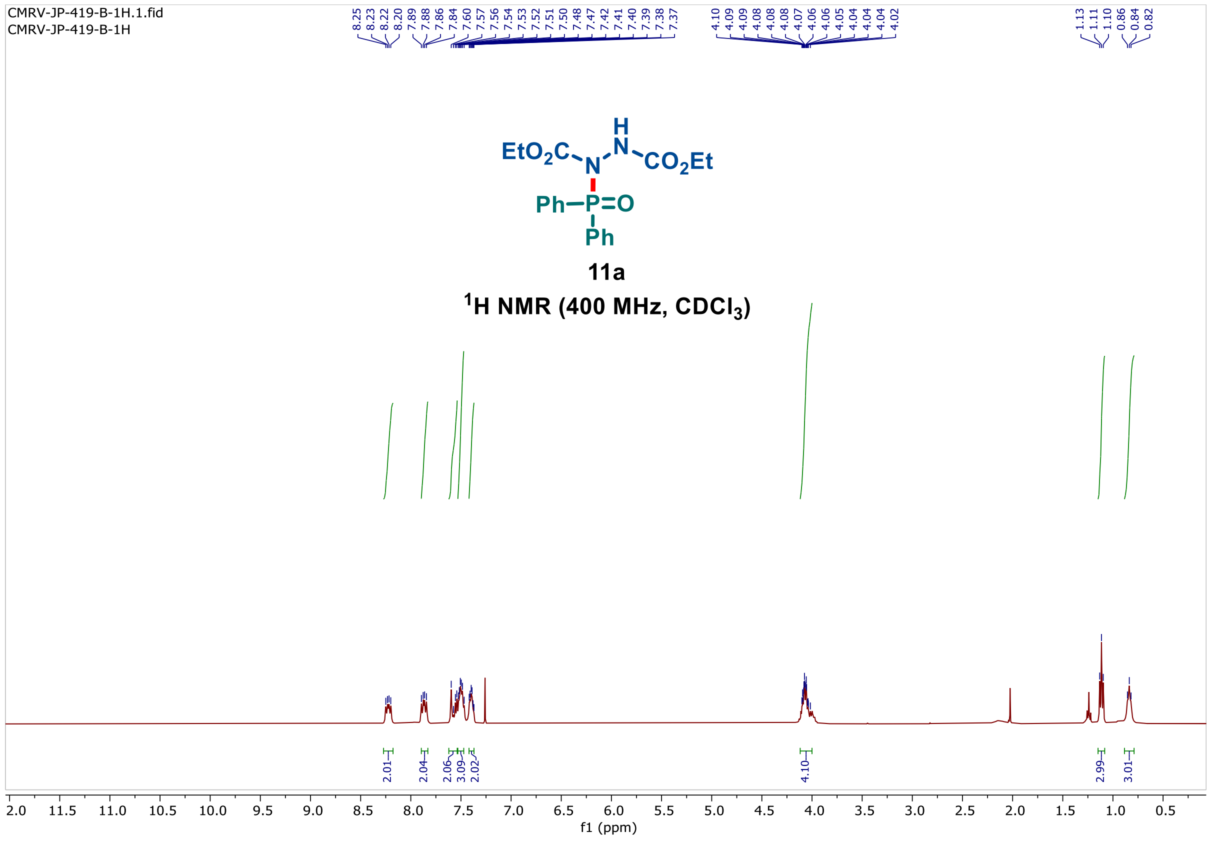
CMRV-JP-419-B-1H.1.fid
CMRV-JP-419-B-1H
11a
^{1}H NMR (400 MHz, CDCl$_3$)
f1 (ppm)

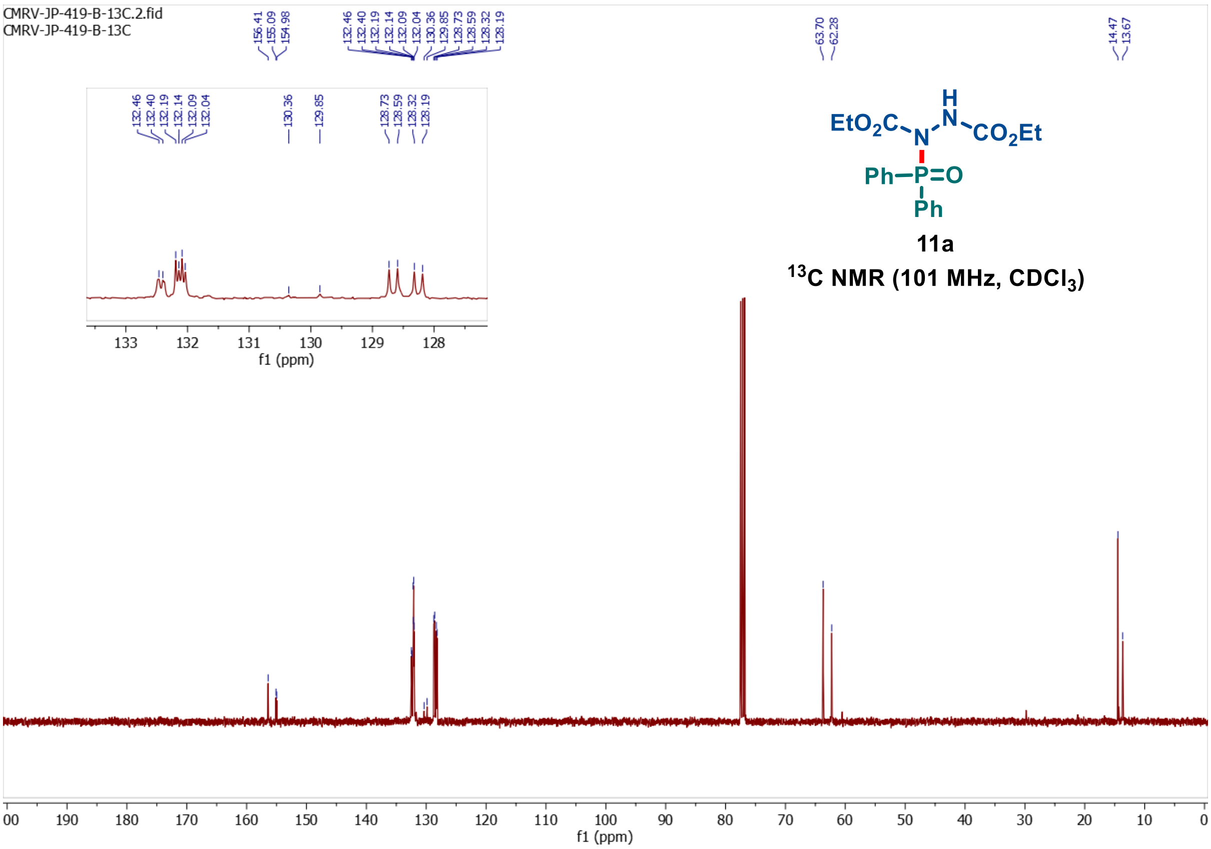
CMRV-JP-419-B-13C.2.fid
CMRV-JP-419-B-13C
11a
^{13}C NMR (101 MHz, $CDCl_3$)
f1 (ppm)

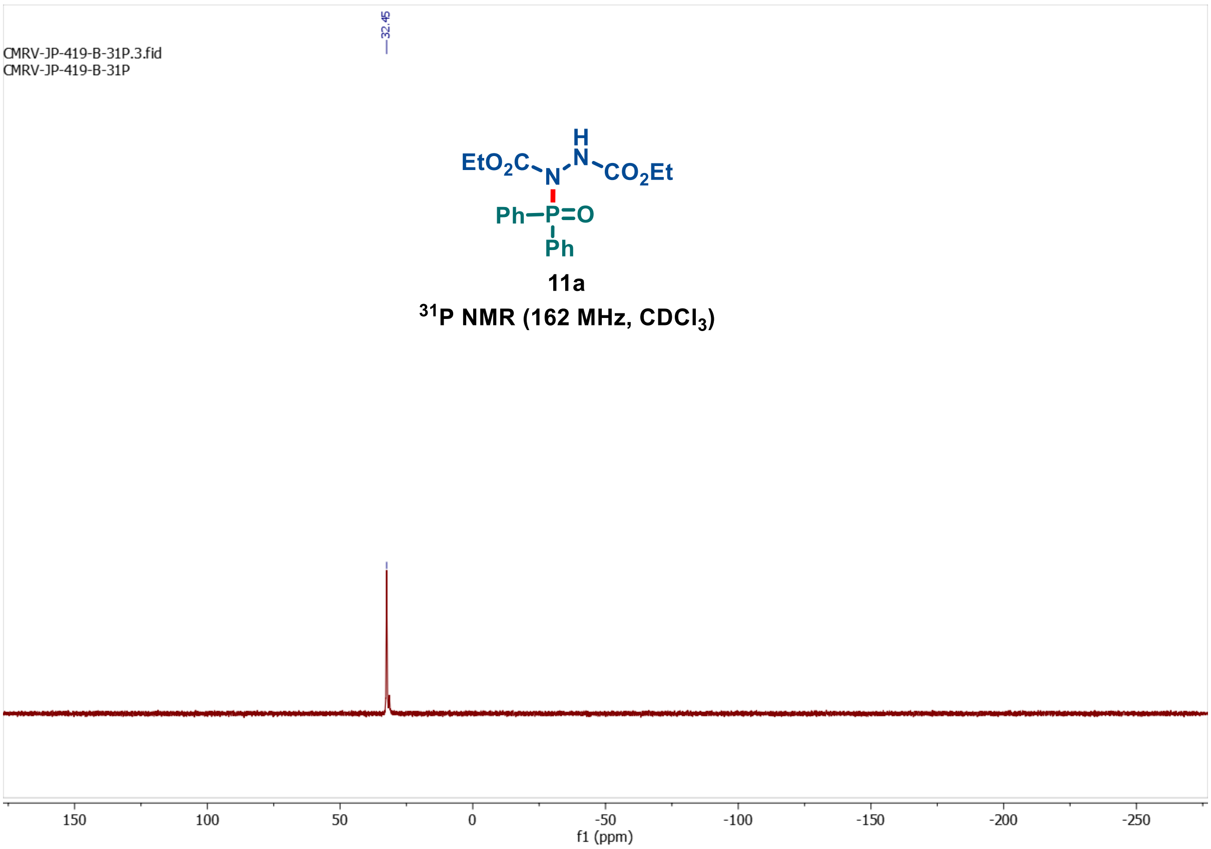
CMRV-JP-419-B-31P.3.fid
CMRV-JP-419-B-31P
11a
^{31}P NMR (162 MHz, $CDCl_3$)
f1 (ppm)

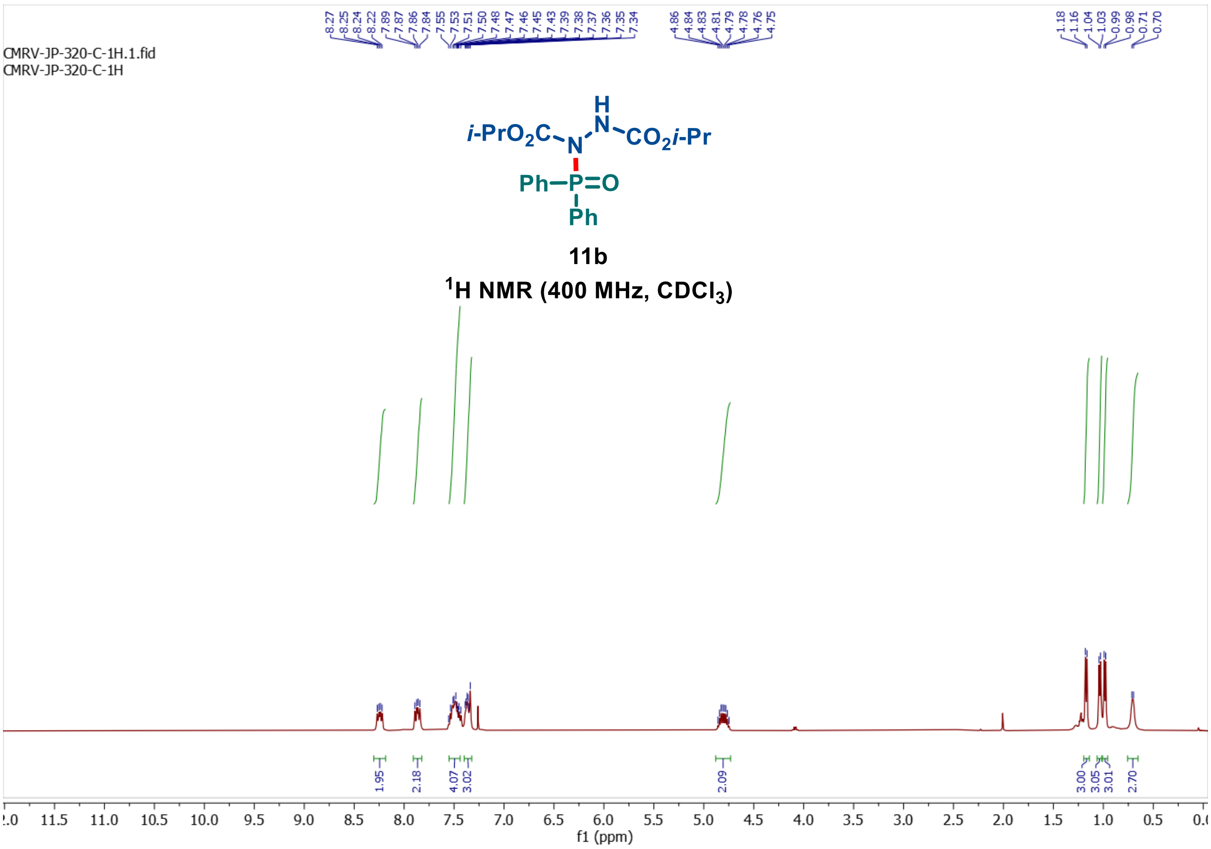
CMRV-JP-320-C-1H.1.fid
CMRV-JP-320-C-1H
11b
^{1}H NMR (400 MHz, $CDCl_3$)
f1 (ppm)

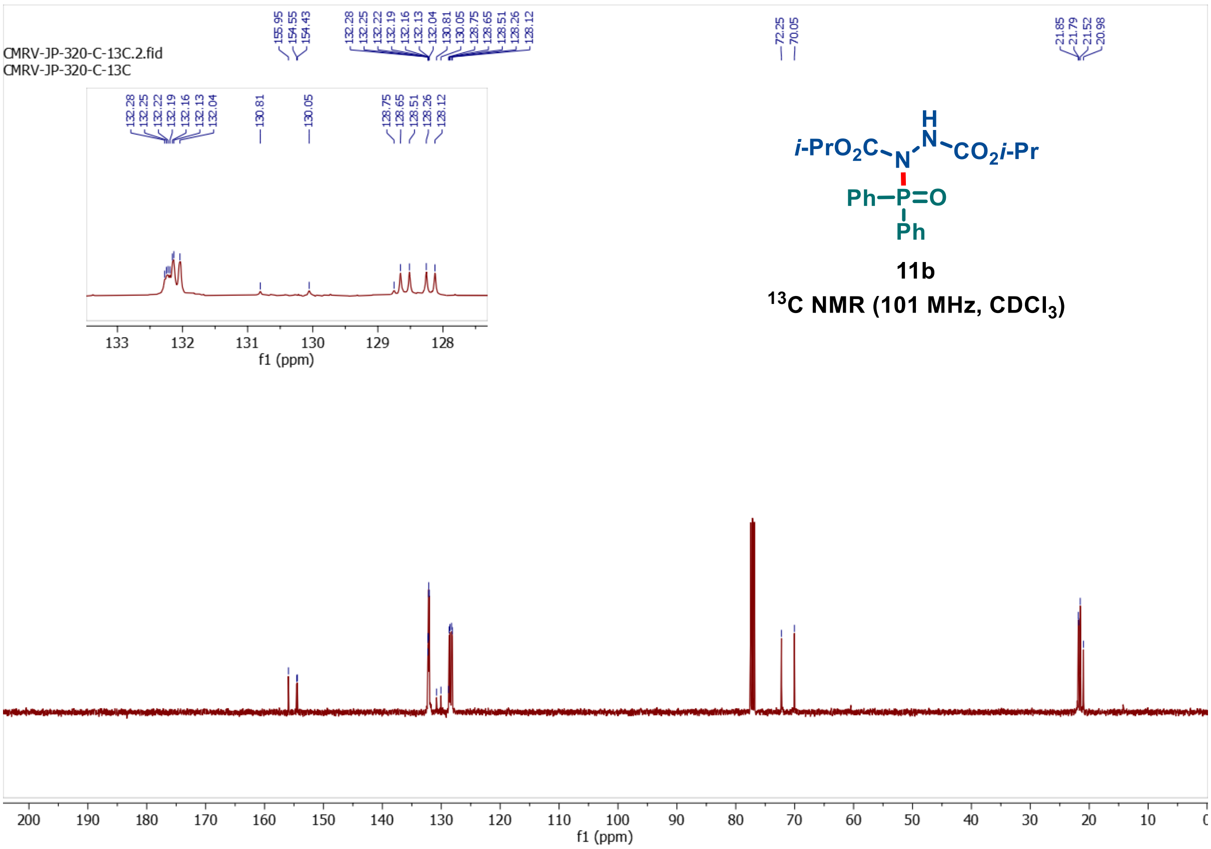
CMRV-JP-320-C-13C.2.fid
CMRV-JP-320-C-13C
11b
^{13}C NMR (101 MHz, $CDCl_3$)
f1 (ppm)

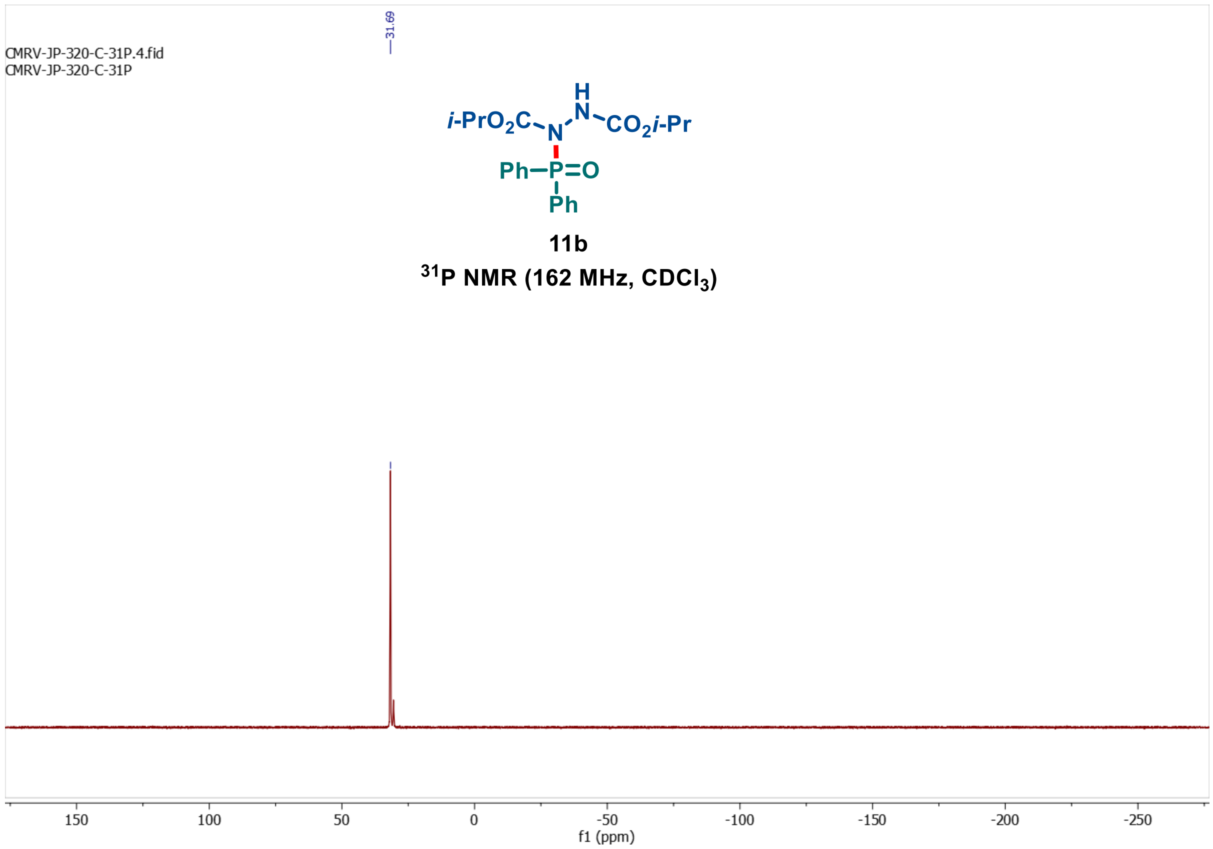

CMRV-JP-320-C-31P.4.fid
CMRV-JP-320-C-31P
31.69
i-PrO2C
N
H
N
CO2i-Pr
Ph
P=O
Ph
11b
31P NMR (162 MHz, CDCl3)
f1 (ppm)

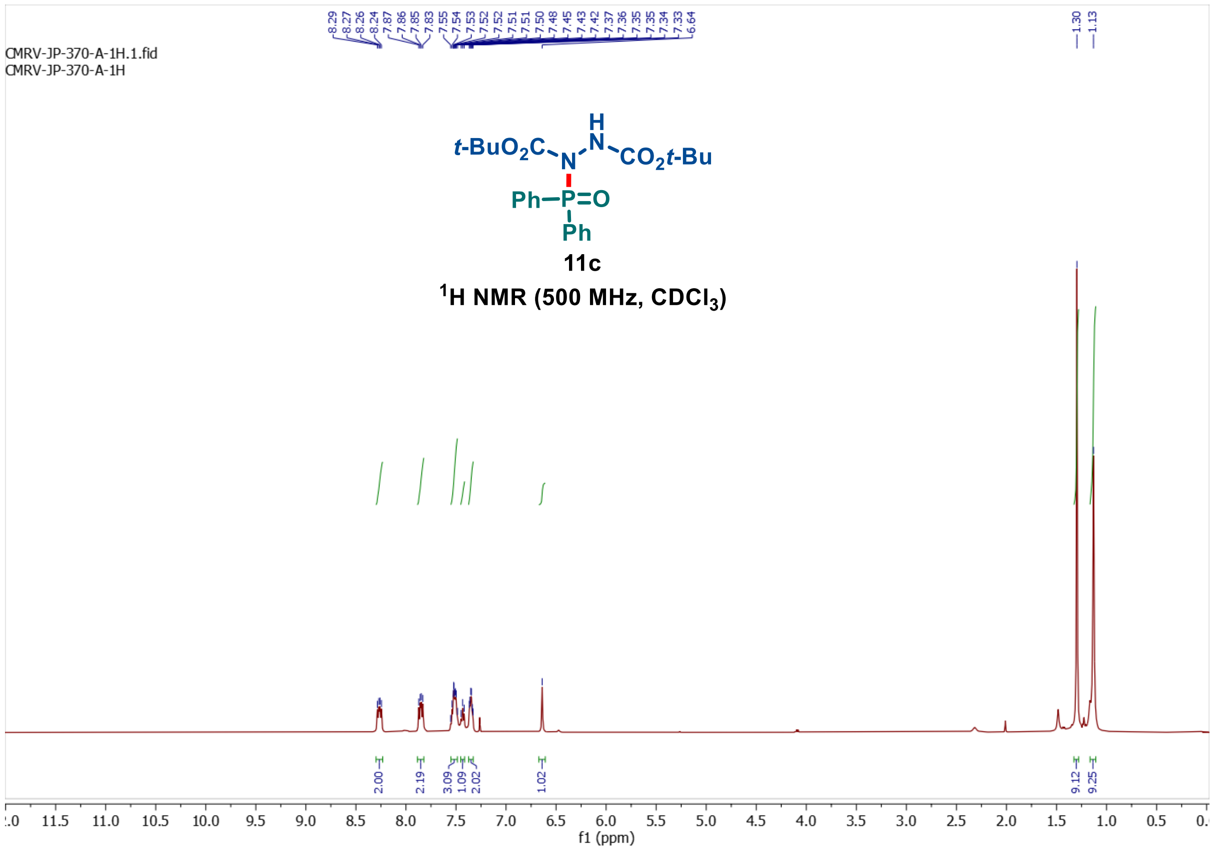

CMRV-JP-370-A-1H.1.fid
CMRV-JP-370-A-1H
t-BuO2C
N
H
N
CO2t-Bu
Ph
P=O
Ph
11c
1H NMR (500 MHz, CDCl3)
1.30
1.13
2.00
2.19
3.09
1.09
2.02
1.02
9.12
9.25
f1 (ppm)

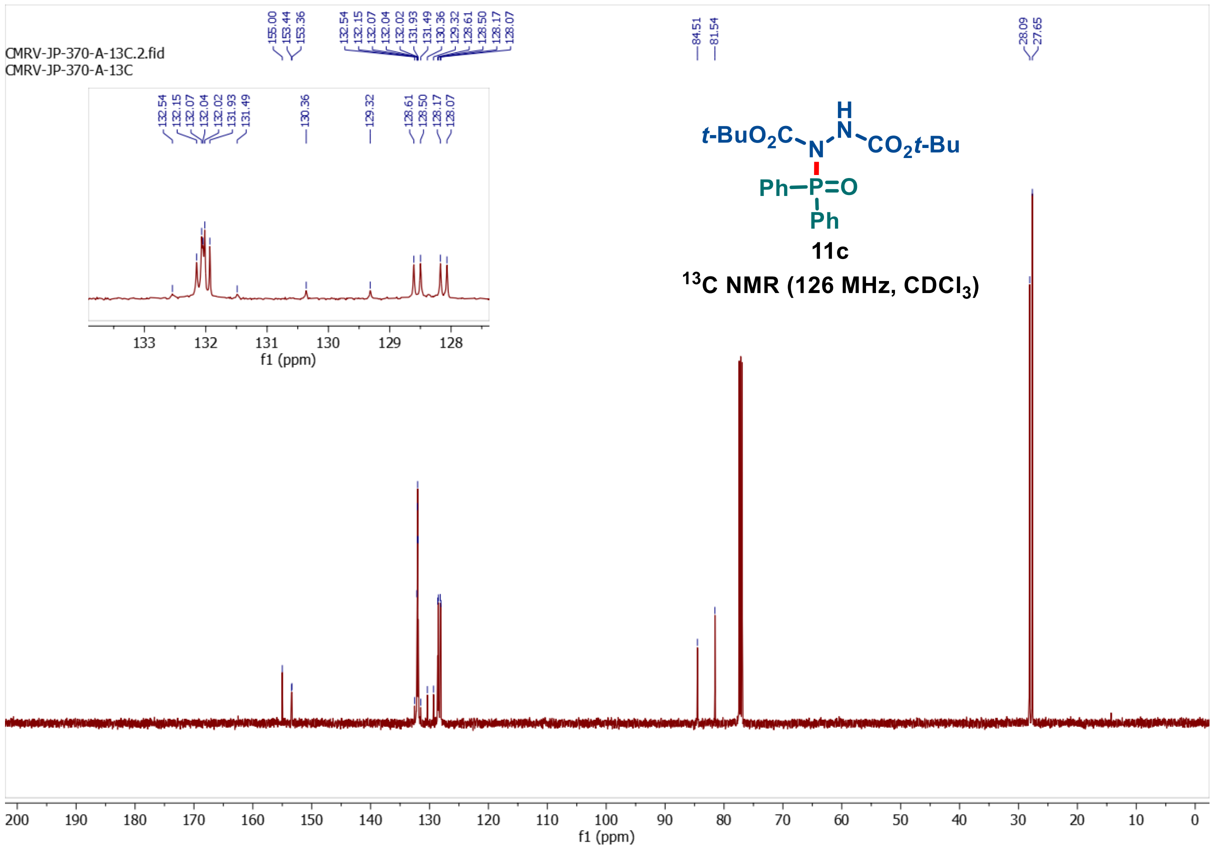
CMRV-JP-370-A-13C.2.fid
CMRV-JP-370-A-13C
155.00
153.44
153.36
132.54
132.15
132.07
132.04
132.02
131.93
131.49
130.36
129.32
128.61
128.50
128.17
128.07
84.51
81.54
28.09
27.65
t-BuO2C
N
H
N
CO2t-Bu
Ph
P=O
Ph
11c
13C NMR (126 MHz, CDCl3)
f1 (ppm)

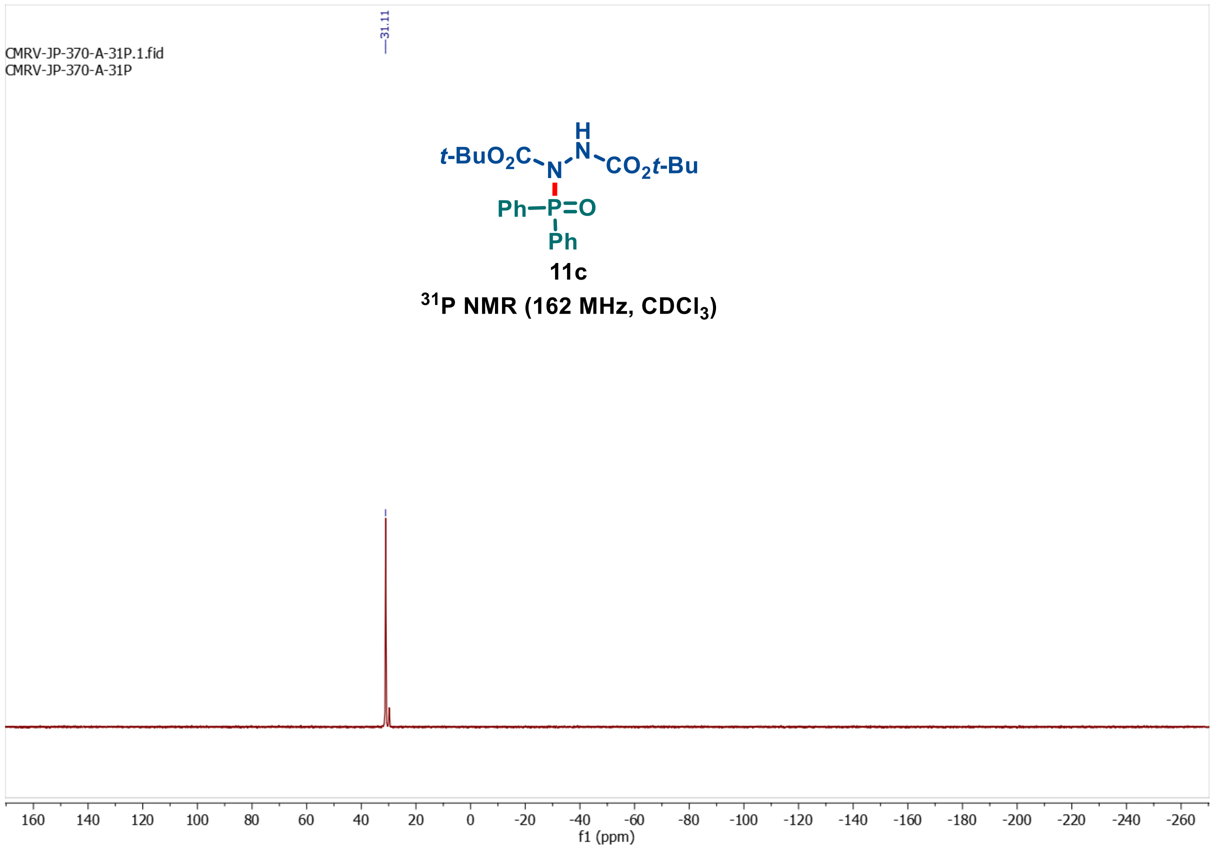
CMRV-JP-370-A-31P.1.fid
CMRV-JP-370-A-31P
31.11
t-BuO2C
N
H
N
CO2t-Bu
Ph
P=O
Ph
11c
31P NMR (162 MHz, CDCl3)
f1 (ppm)

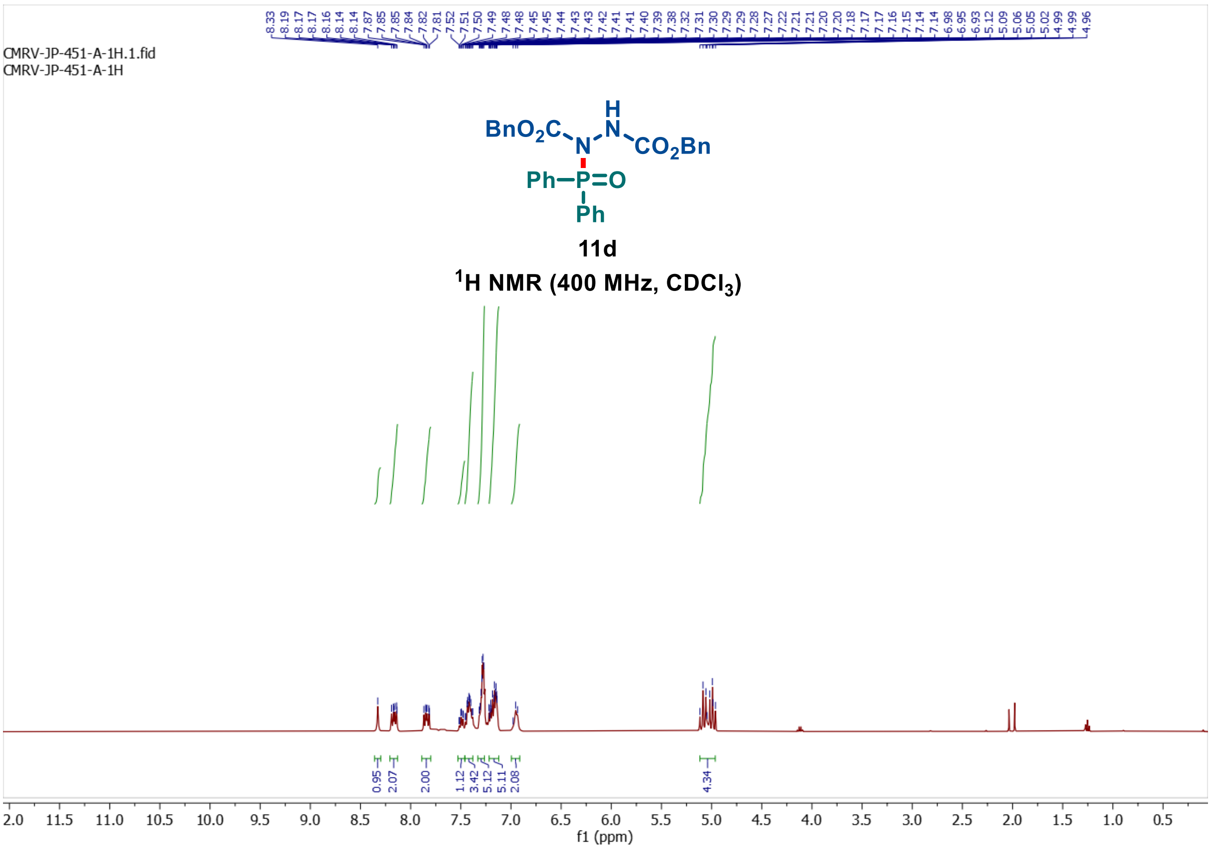
CMRV-JP-451-A-1H.1.fid
CMRV-JP-451-A-1H
8.33 8.19 8.17 8.17 8.16 8.14 8.14 7.87 7.85 7.84 7.82 7.81 7.52 7.51 7.50 7.49 7.48 7.48 7.45 7.45 7.44 7.43 7.43 7.42 7.41 7.41 7.40 7.39 7.38 7.32 7.31 7.30 7.29 7.29 7.28 7.27 7.22 7.21 7.21 7.20 7.18 7.17 7.17 7.16 7.15 7.14 7.14 6.98 6.95 6.93 5.12 5.09 5.06 5.05 5.02 4.99 4.96
11d
¹H NMR (400 MHz, CDCl₃)
0.95 2.07 2.00 1.12 3.42 5.12 5.11 2.08 4.34
f1 (ppm)

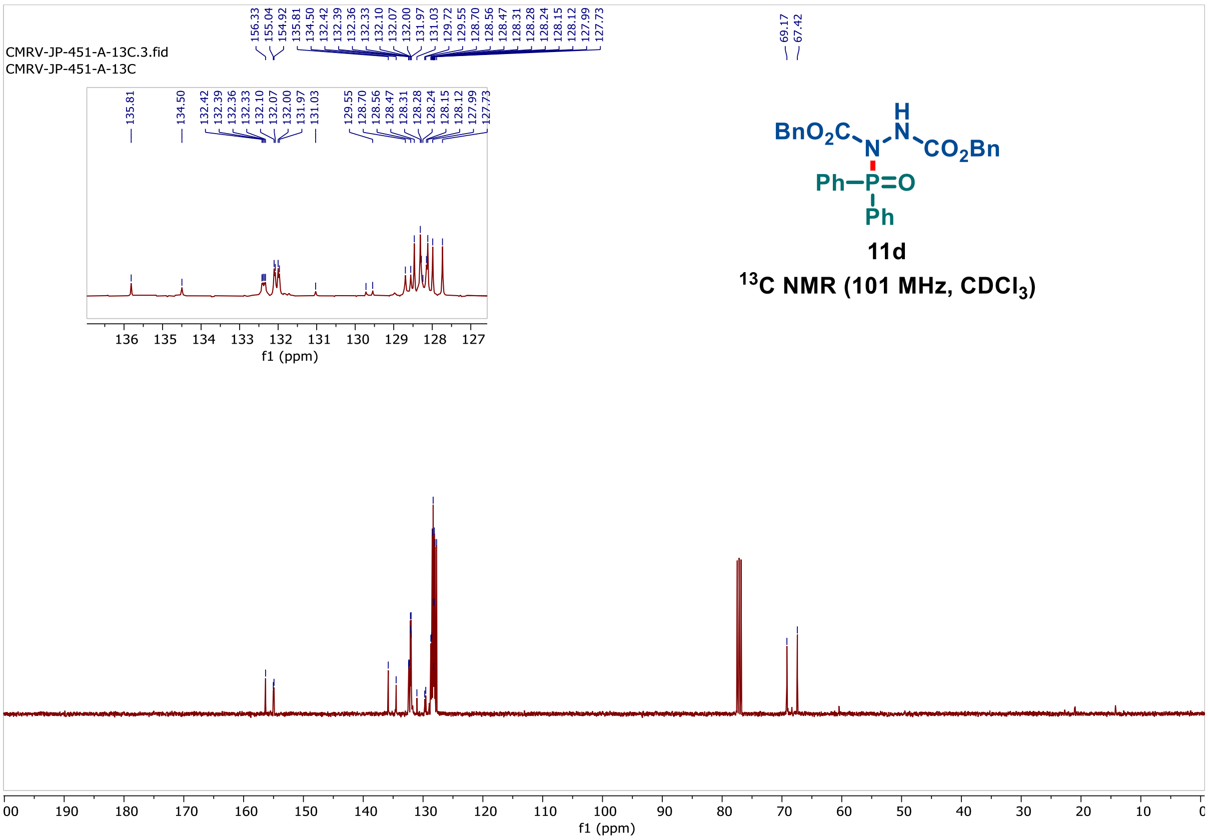
CMRV-JP-451-A-13C.3.fid
CMRV-JP-451-A-13C
156.33 155.04 154.92 135.81 134.50 132.42 132.39 132.36 132.33 132.10 132.07 132.00 131.97 131.03 129.72 129.55 128.70 128.56 128.47 128.31 128.28 128.24 128.15 128.12 127.99 127.73
69.17 67.42
11d
¹³C NMR (101 MHz, CDCl₃)
f1 (ppm)

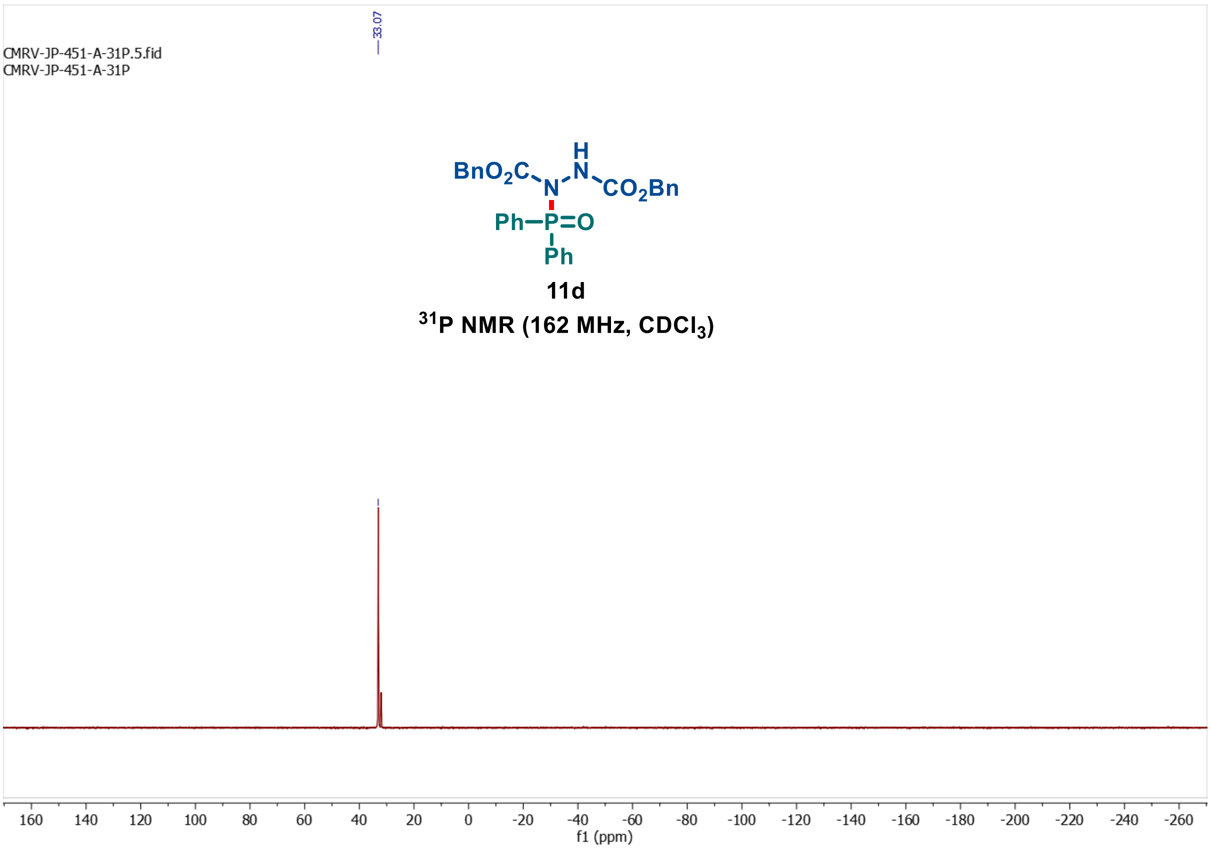

33.07
CMRV-JP-451-A-31P.5.fid
CMRV-JP-451-A-31P
BnO2C
N
H
N
CO2Bn
Ph—P=O
Ph
11d
31P NMR (162 MHz, CDCl3)
160 140 120 100 80 60 40 20 0 -20 -40 -60 -80 -100 -120 -140 -160 -180 -200 -220 -240 -260
f1 (ppm)

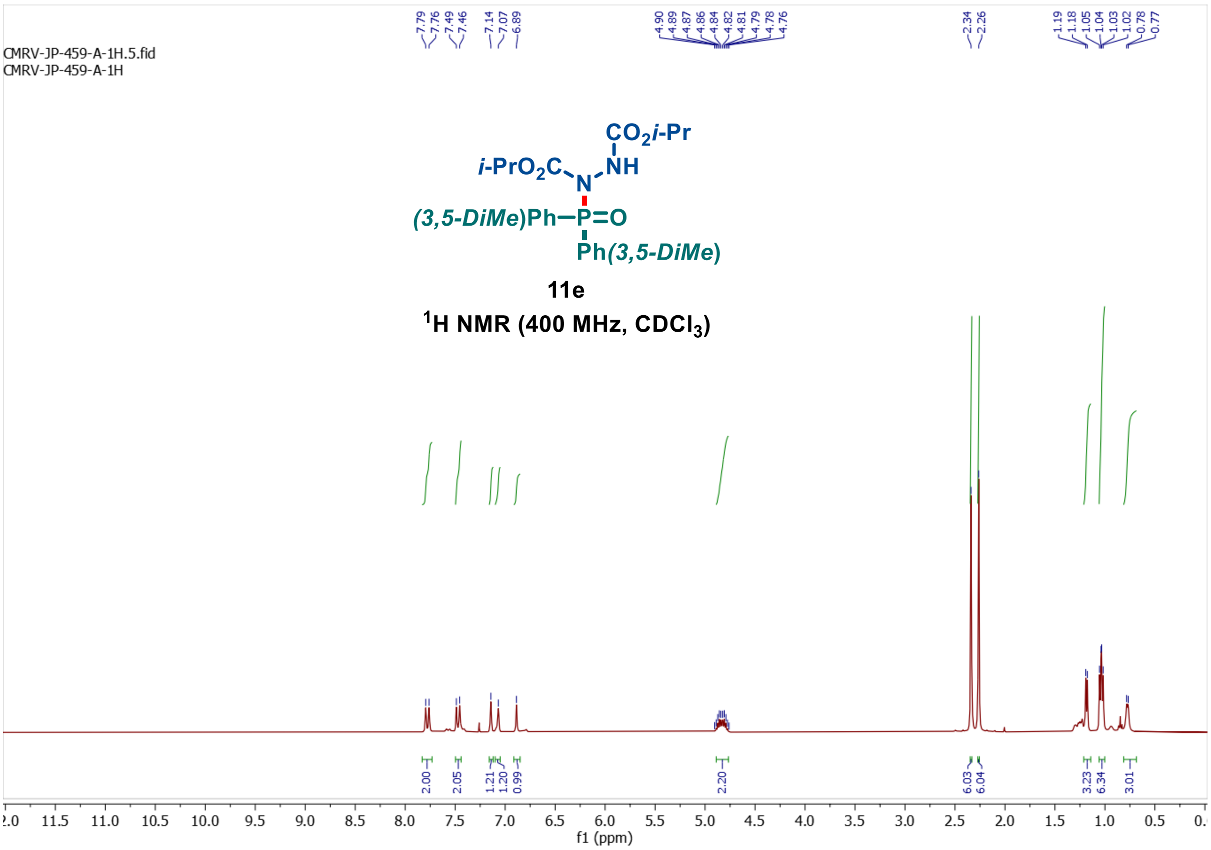

7.79 7.76 7.49 7.46 7.14 7.07 6.89
4.90 4.89 4.87 4.86 4.84 4.82 4.81 4.79 4.78 4.76
2.34 2.26
1.19 1.18 1.05 1.04 1.03 1.02 0.78 0.77
CMRV-JP-459-A-1H.5.fid
CMRV-JP-459-A-1H
CO2i-Pr
i-PrO2C
N
NH
(3,5-DiMe)Ph—P=O
Ph(3,5-DiMe)
11e
1H NMR (400 MHz, CDCl3)
2.00 2.05 1.21 1.20 0.99 2.20 6.03 6.04 3.23 6.34 3.01
11.5 11.0 10.5 10.0 9.5 9.0 8.5 8.0 7.5 7.0 6.5 6.0 5.5 5.0 4.5 4.0 3.5 3.0 2.5 2.0 1.5 1.0 0.5
f1 (ppm)

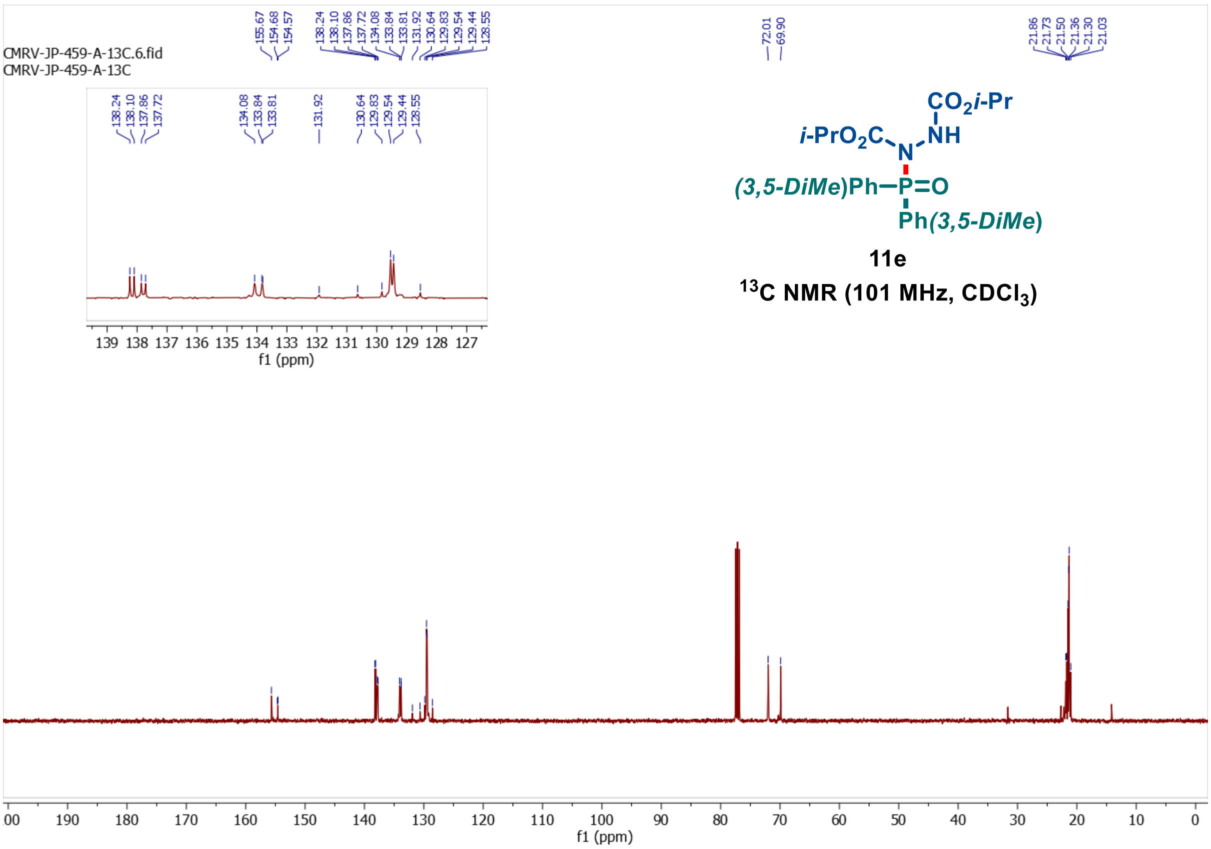
CMRV-JP-459-A-13C.6.fid
CMRV-JP-459-A-13C
155.67
154.68
154.57
138.24
138.10
137.86
137.72
134.08
133.84
133.81
131.92
130.64
129.83
129.54
129.44
128.55
72.01
69.90
21.86
21.73
21.50
21.36
21.30
21.03
CO2i-Pr
i-PrO2C
N
NH
(3,5-DiMe)Ph
P=O
Ph(3,5-DiMe)
11e
13C NMR (101 MHz, CDCl3)
f1 (ppm)

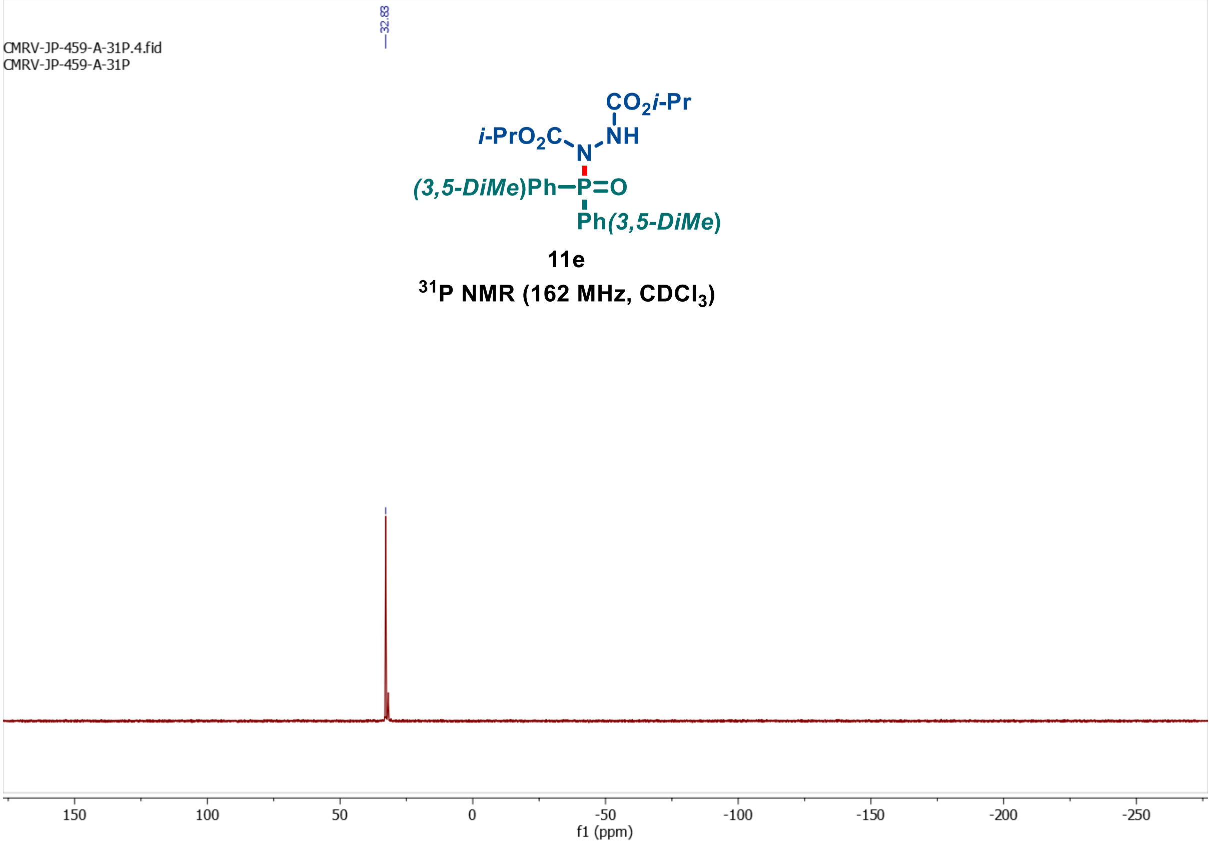
CMRV-JP-459-A-31P.4.fid
CMRV-JP-459-A-31P
32.83
CO2i-Pr
i-PrO2C
N
NH
(3,5-DiMe)Ph
P=O
Ph(3,5-DiMe)
11e
31P NMR (162 MHz, CDCl3)
f1 (ppm)

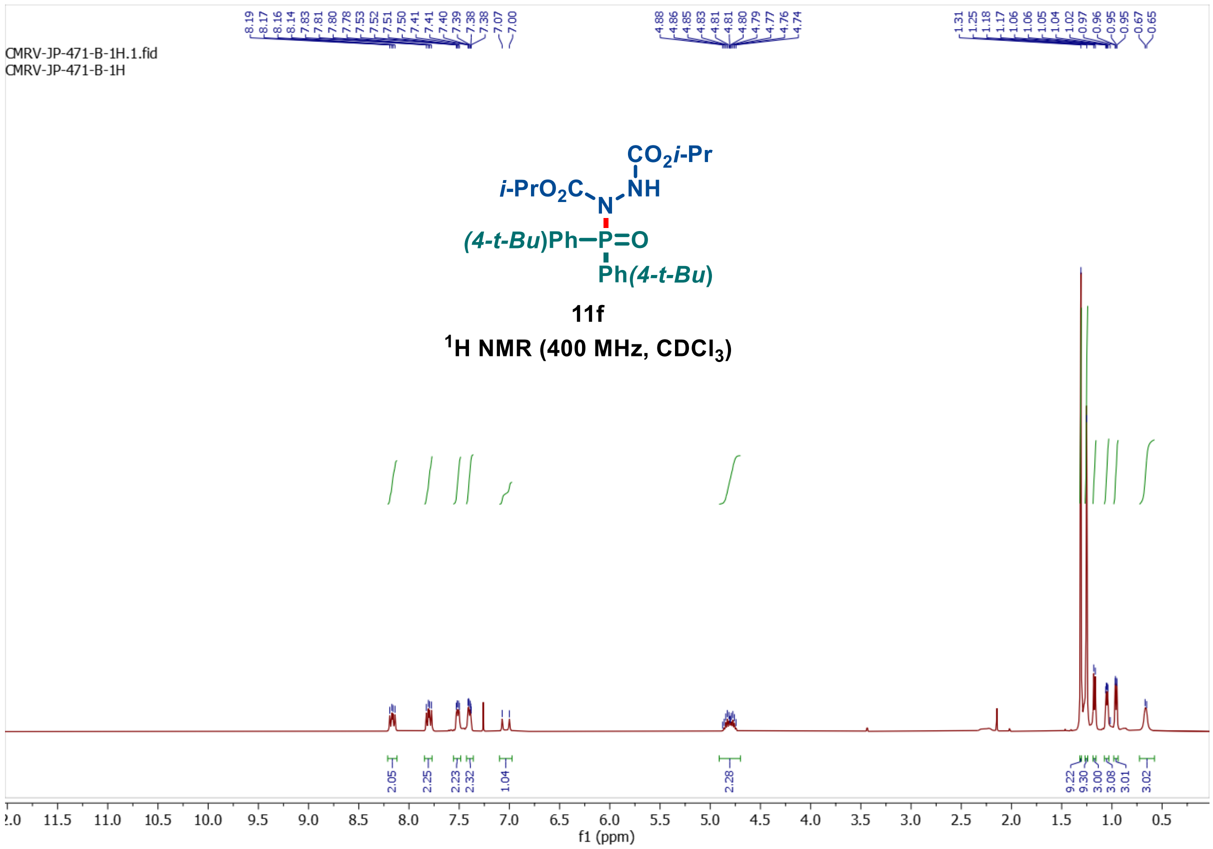
CMRV-JP-471-B-1H.1.fid
CMRV-JP-471-B-1H
11f
1H NMR (400 MHz, CDCl3)
f1 (ppm)

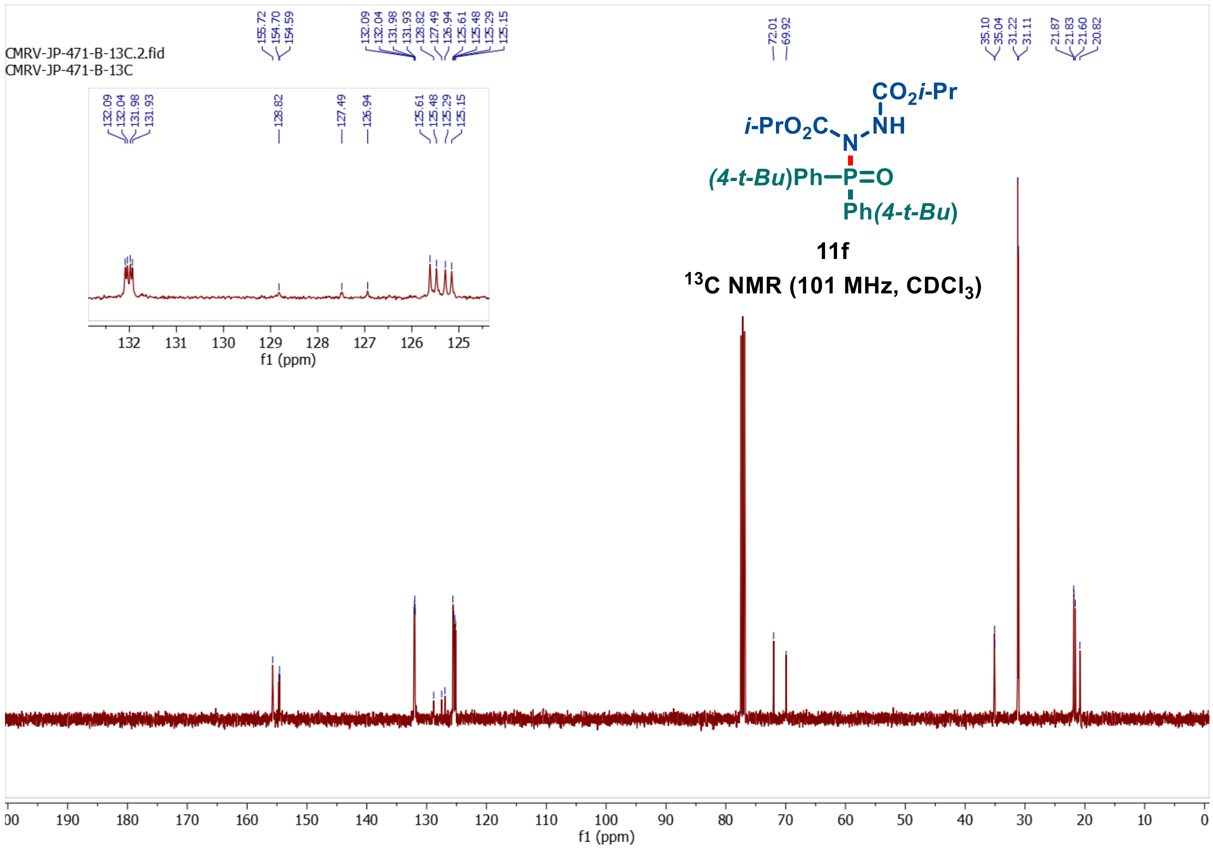
CMRV-JP-471-B-13C.2.fid
CMRV-JP-471-B-13C
11f
13C NMR (101 MHz, CDCl3)
f1 (ppm)

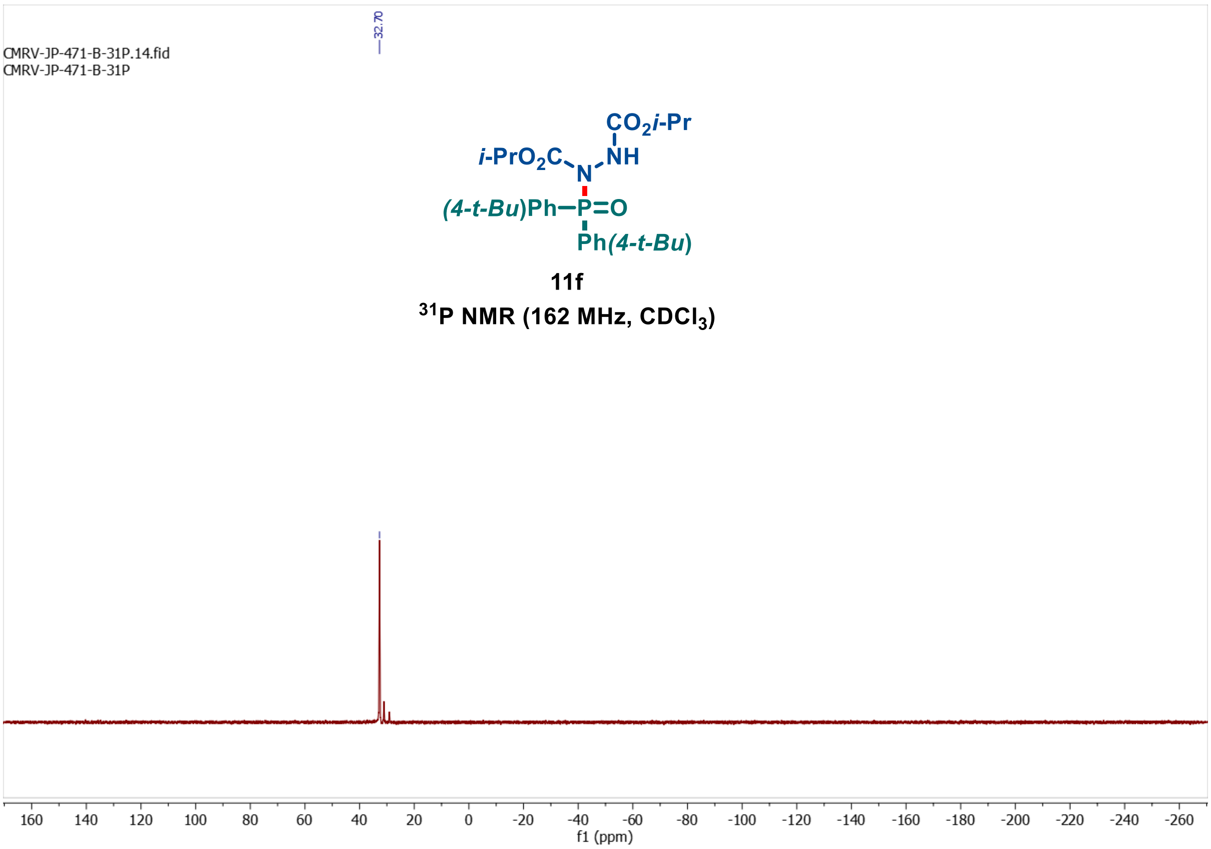
CMRV-JP-471-B-31P.14.fid
CMRV-JP-471-B-31P
32.70
CO2i-Pr
i-PrO2C
N
NH
(4-t-Bu)Ph
P=O
Ph(4-t-Bu)
11f
31P NMR (162 MHz, CDCl3)
f1 (ppm)

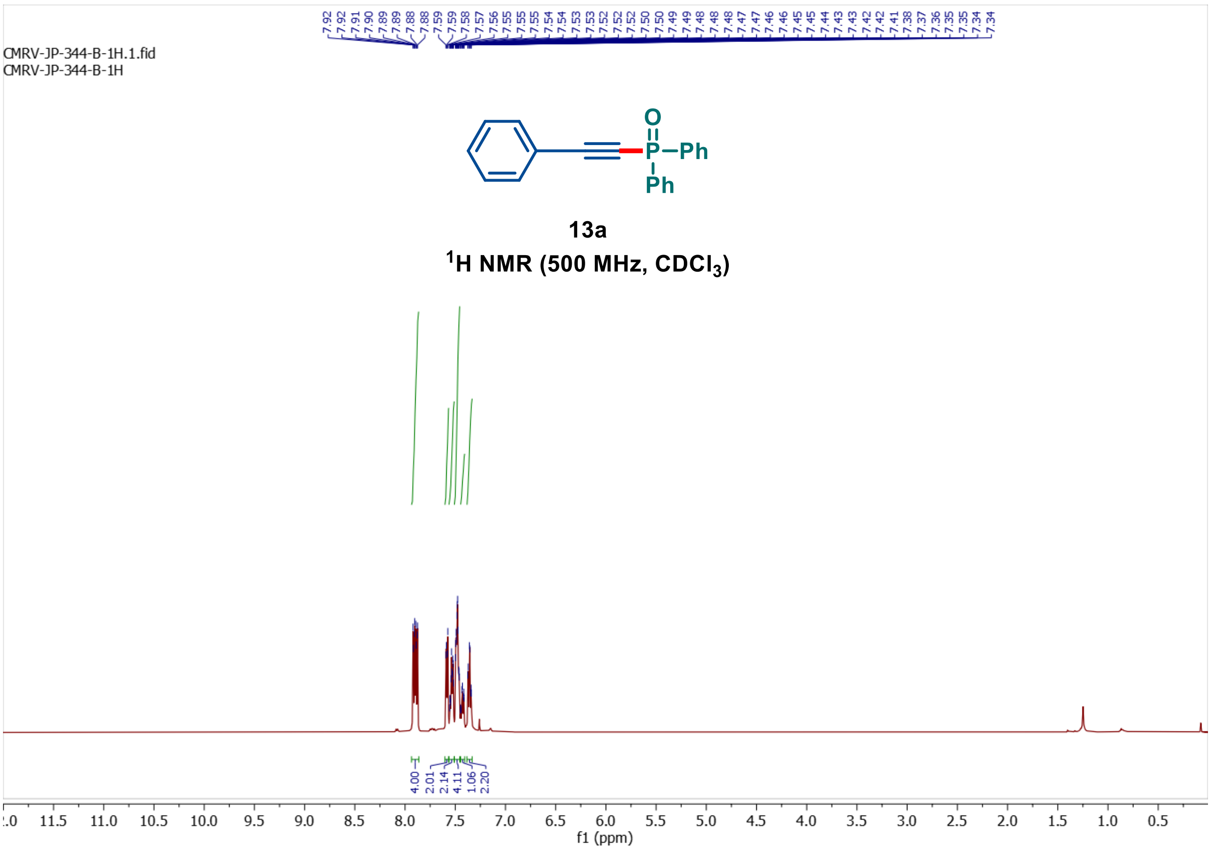
CMRV-JP-344-B-1H.1.fid
CMRV-JP-344-B-1H
7.92 7.92 7.91 7.90 7.89 7.89 7.88 7.88 7.59 7.59 7.58 7.57 7.56 7.55 7.55 7.55 7.54 7.54 7.53 7.53 7.52 7.52 7.52 7.50 7.50 7.49 7.49 7.48 7.48 7.47 7.47 7.46 7.45 7.45 7.44 7.43 7.43 7.42 7.42 7.41 7.38 7.37 7.36 7.35 7.35 7.34 7.34
O
P–Ph
Ph
13a
1H NMR (500 MHz, CDCl3)
4.00 2.01 2.14 4.11 1.06 2.20
f1 (ppm)

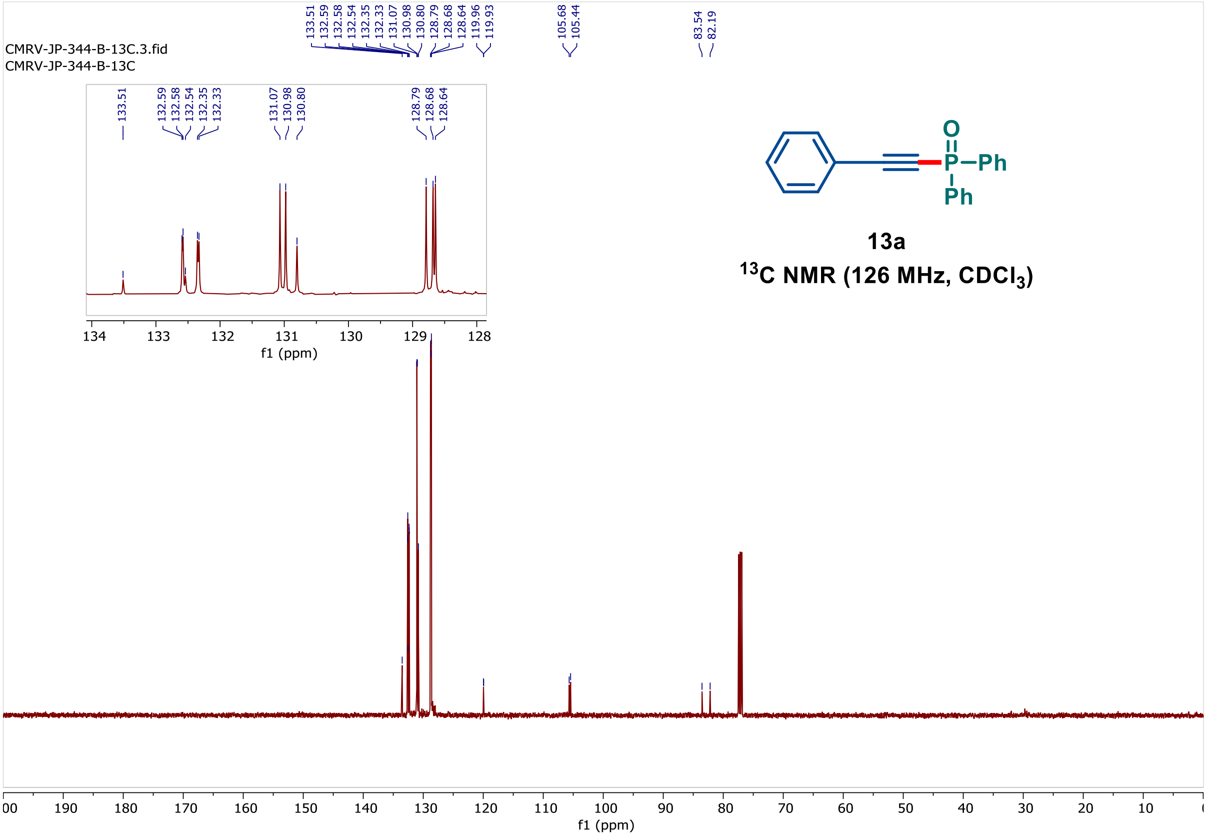
CMRV-JP-344-B-13C.3.fid
CMRV-JP-344-B-13C
133.51
132.59
132.58
132.54
132.35
132.33
131.07
130.98
130.80
128.79
128.68
128.64
119.96
119.93
105.68
105.44
83.54
82.19
O
P–Ph
Ph
13a
13C NMR (126 MHz, CDCl3)
f1 (ppm)

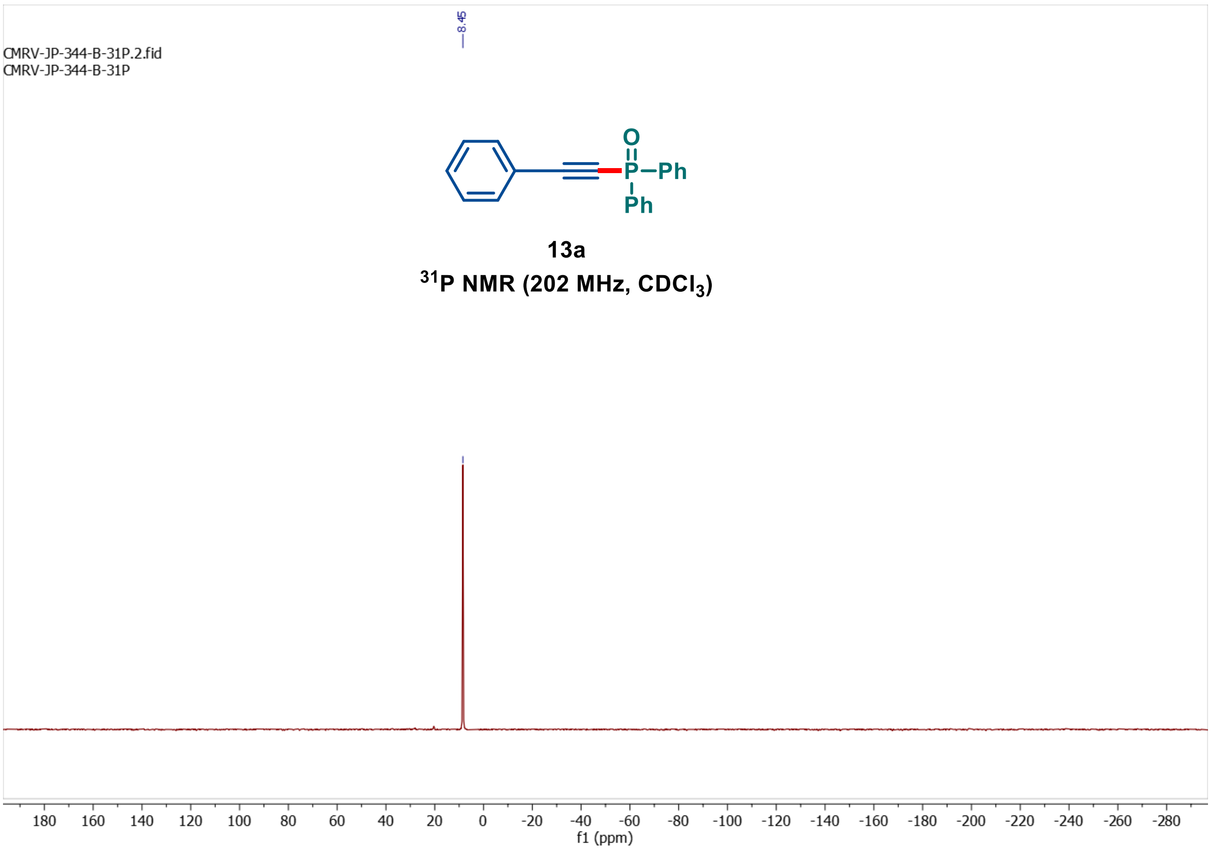
CMRV-JP-344-B-31P.2.fid
CMRV-JP-344-B-31P
8.45
O
P–Ph
Ph
13a
31P NMR (202 MHz, CDCl3)
f1 (ppm)

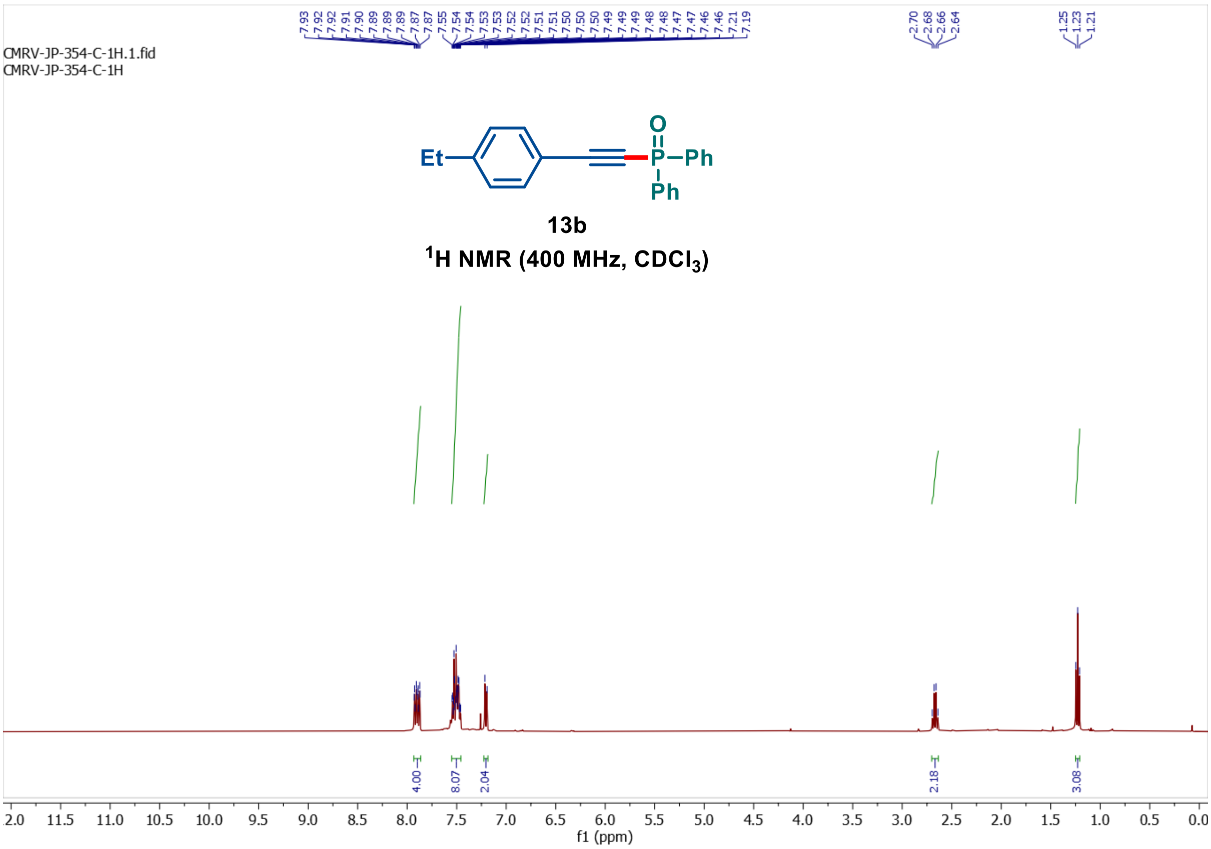
CMRV-JP-354-C-1H.1.fid
CMRV-JP-354-C-1H
13b
1H NMR (400 MHz, CDCl3)
f1 (ppm)

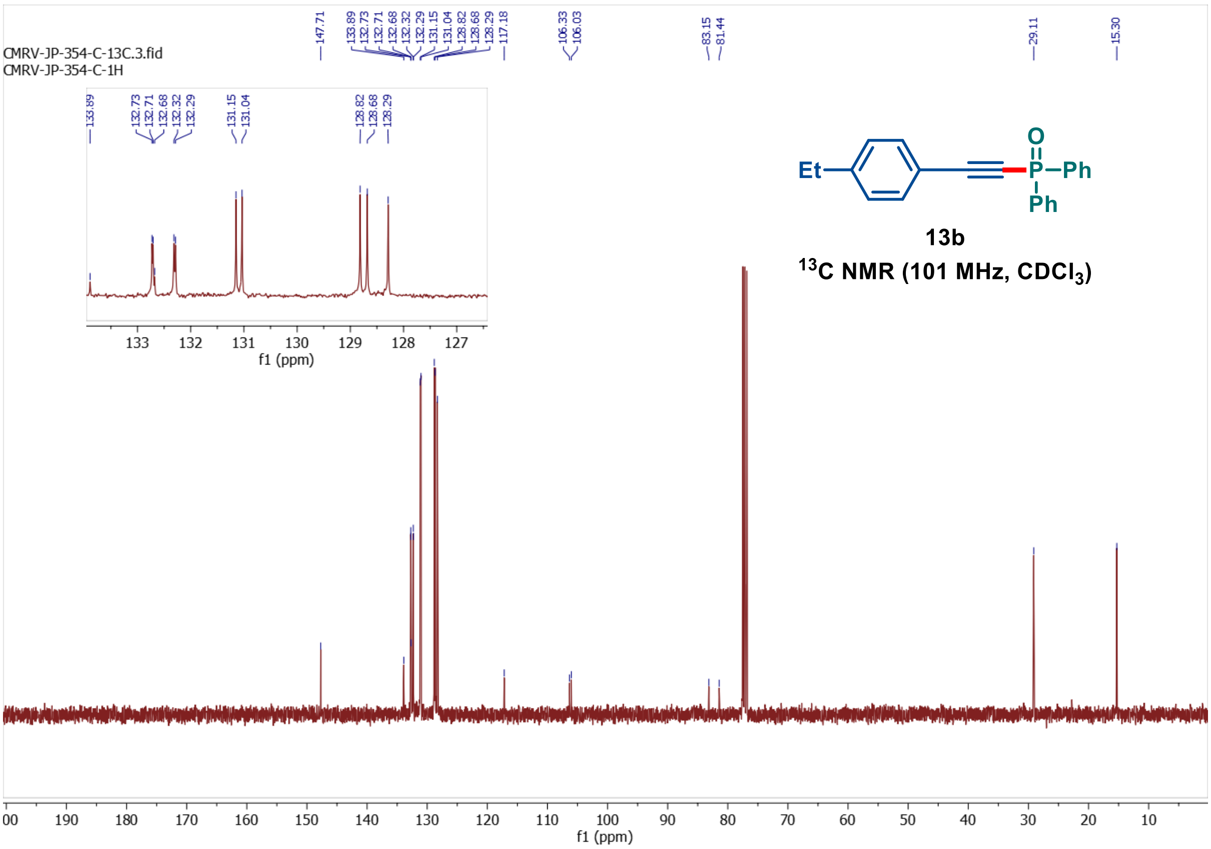
CMRV-JP-354-C-13C.3.fid
CMRV-JP-354-C-1H
13b
13C NMR (101 MHz, CDCl3)
f1 (ppm)

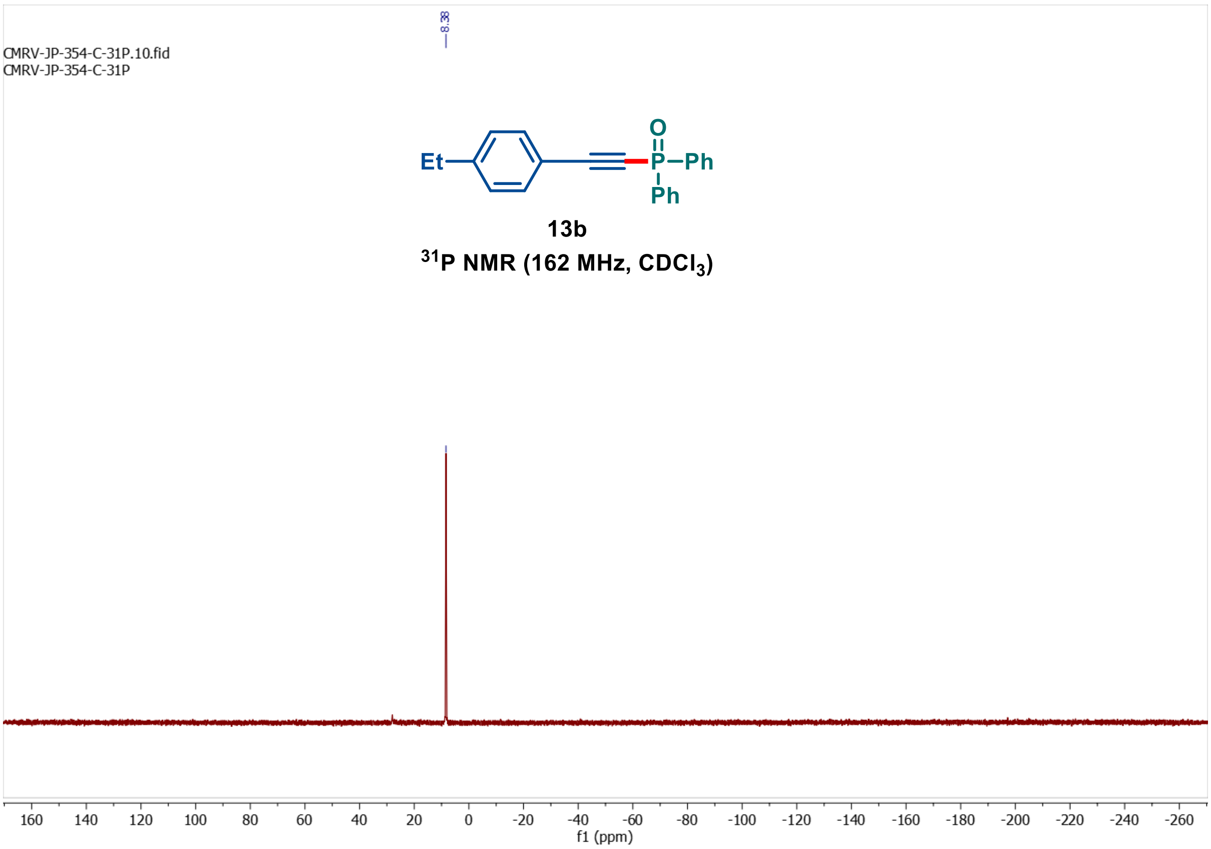
CMRV-JP-354-C-31P.10.fid
CMRV-JP-354-C-31P
8.38
Et
O
P–Ph
Ph
13b
^{31}P NMR (162 MHz, CDCl$_3$)
160 140 120 100 80 60 40 20 0 -20 -40 -60 -80 -100 -120 -140 -160 -180 -200 -220 -240 -260
f1 (ppm)

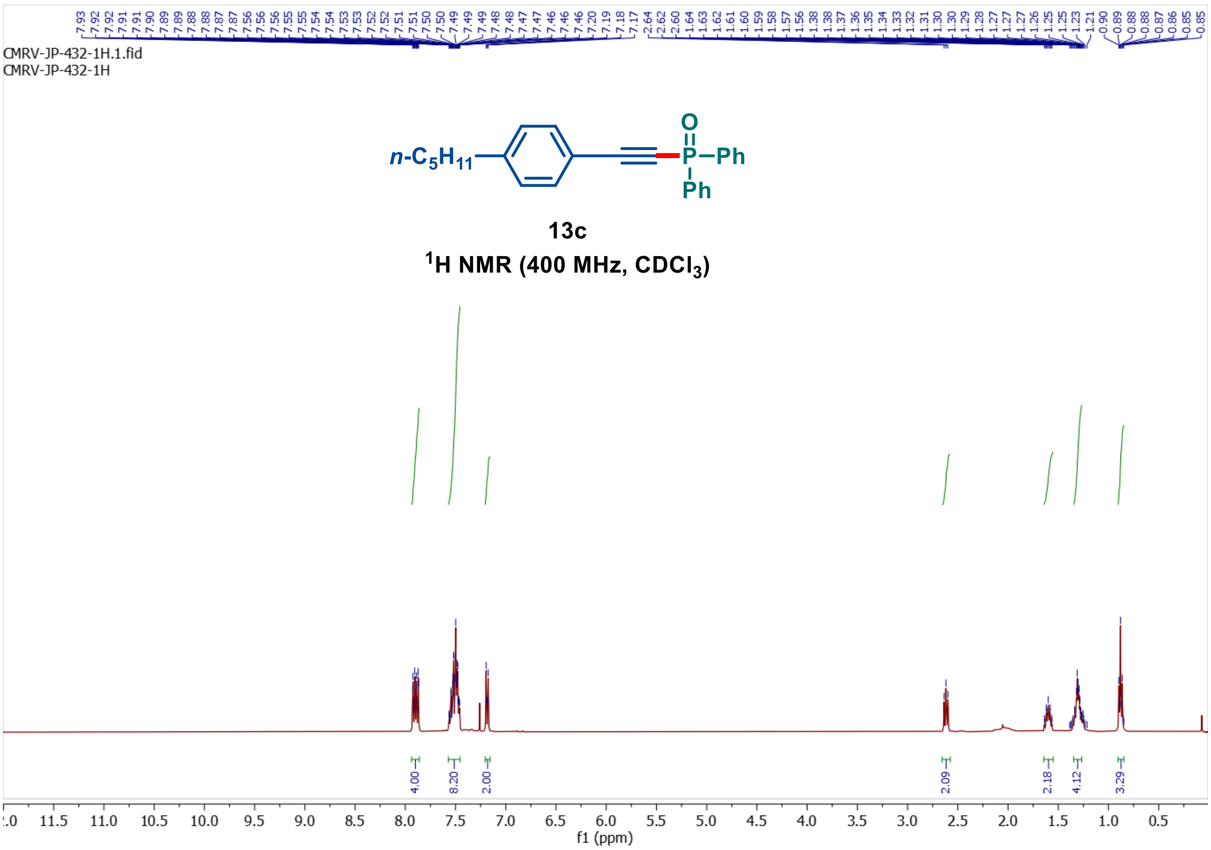
CMRV-JP-432-1H.1.fid
CMRV-JP-432-1H
n-C$_5$H$_{11}$
O
P–Ph
Ph
13c
^{1}H NMR (400 MHz, CDCl$_3$)
4.00 8.20 2.00 2.09 2.18 4.12 3.29
11.5 11.0 10.5 10.0 9.5 9.0 8.5 8.0 7.5 7.0 6.5 6.0 5.5 5.0 4.5 4.0 3.5 3.0 2.5 2.0 1.5 1.0 0.5
f1 (ppm)

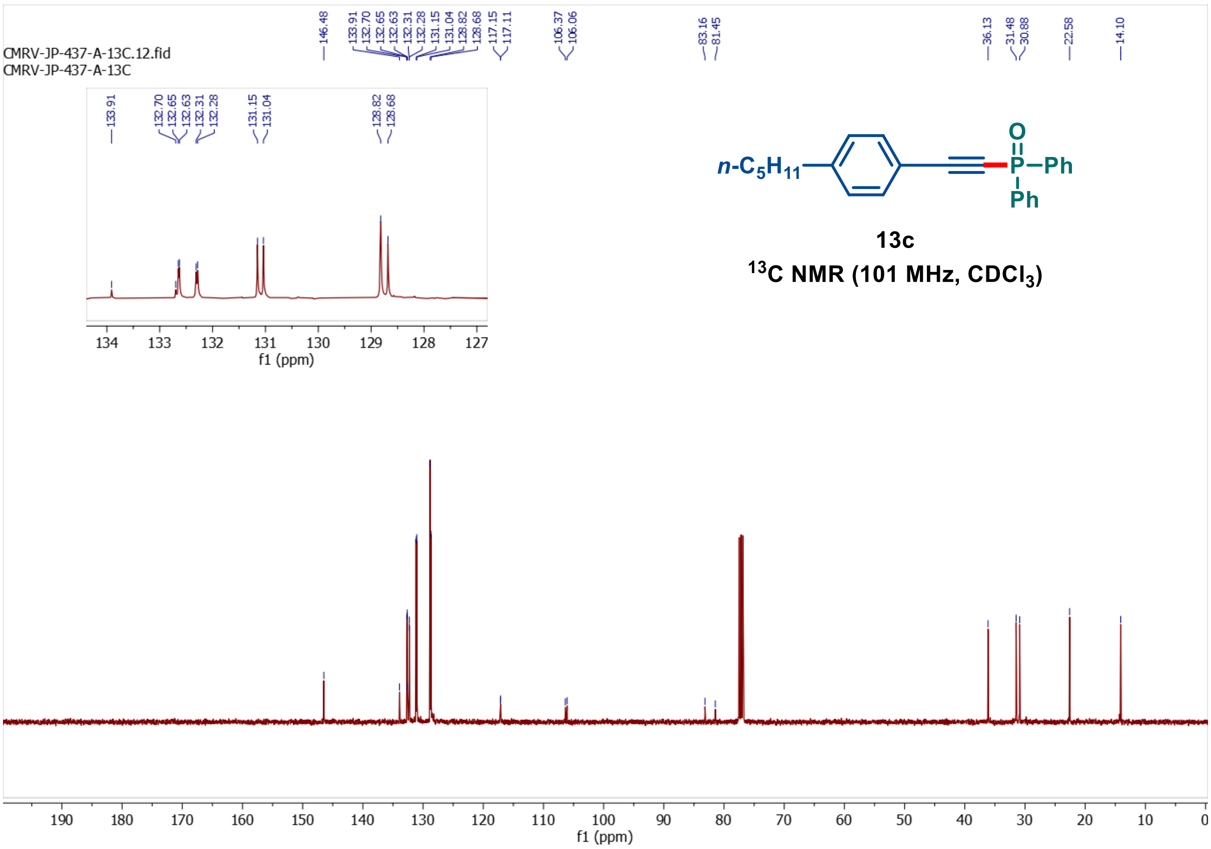
CMRV-JP-437-A-13C.12.fid
CMRV-JP-437-A-13C
146.48
133.91
132.70
132.65
132.63
132.31
132.28
131.15
131.04
128.82
128.68
117.15
117.11
106.37
106.06
83.16
81.45
36.13
31.48
30.88
22.58
14.10
n-C5H11
O
P
Ph
Ph
13c
13C NMR (101 MHz, CDCl3)
f1 (ppm)

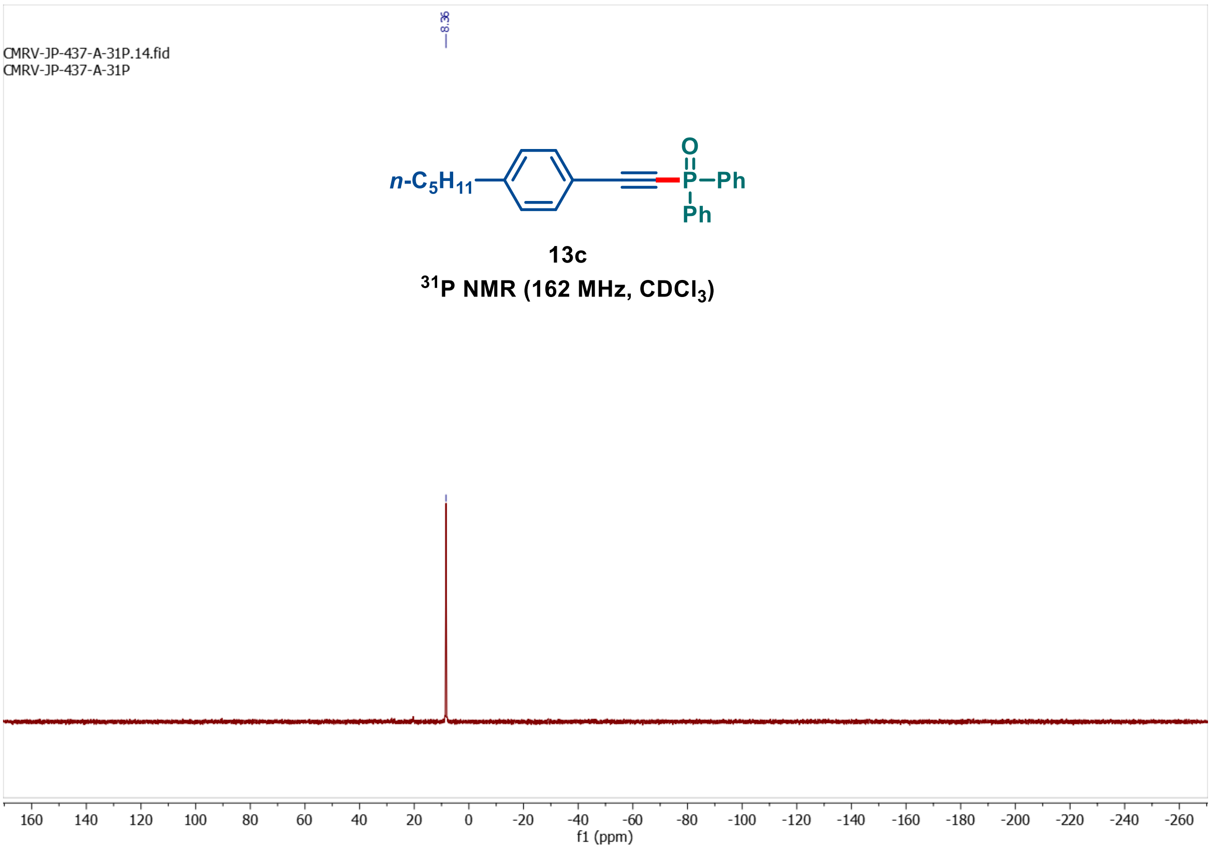
CMRV-JP-437-A-31P.14.fid
CMRV-JP-437-A-31P
8.36
n-C5H11
O
P
Ph
Ph
13c
31P NMR (162 MHz, CDCl3)
f1 (ppm)

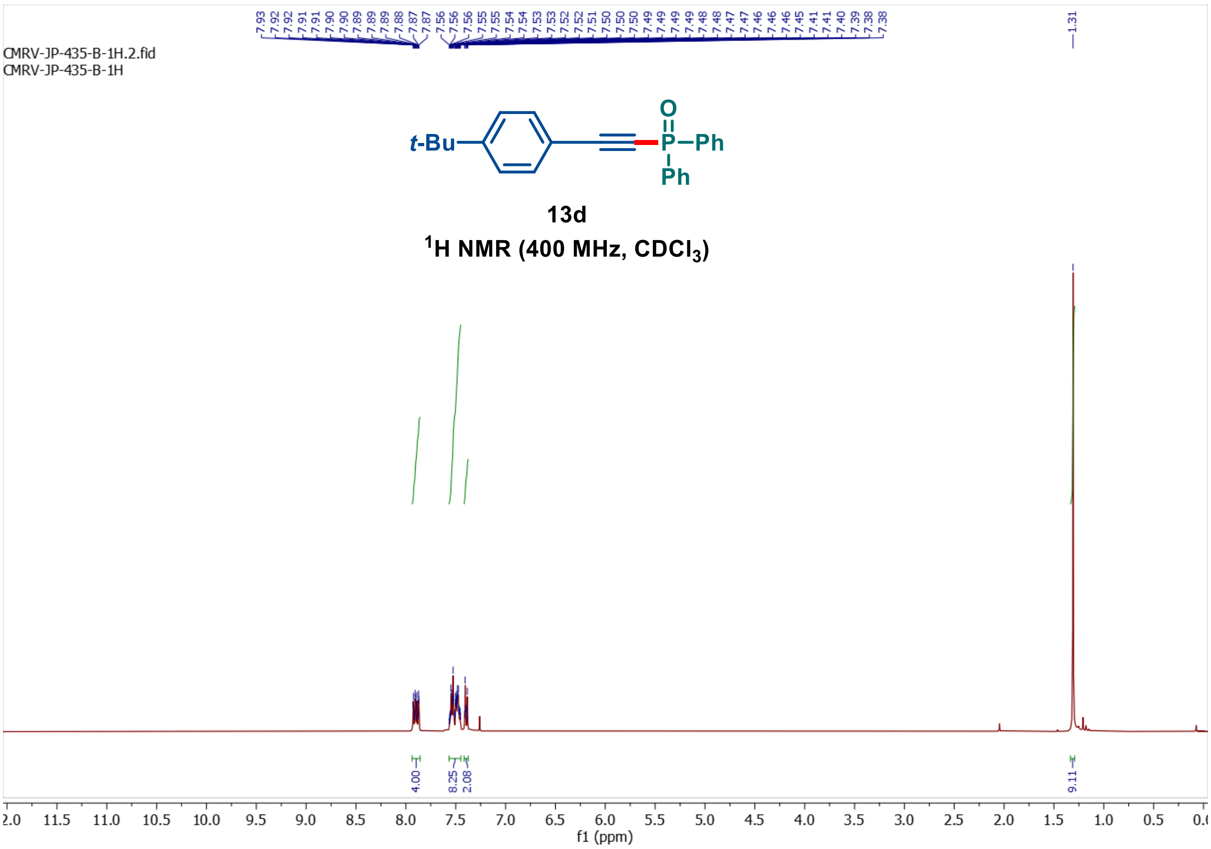
CMRV-JP-435-B-1H.2.fid
CMRV-JP-435-B-1H
t-Bu
O
P
Ph
Ph
13d
1H NMR (400 MHz, CDCl3)
4.00
8.25
2.08
9.11
1.31
f1 (ppm)

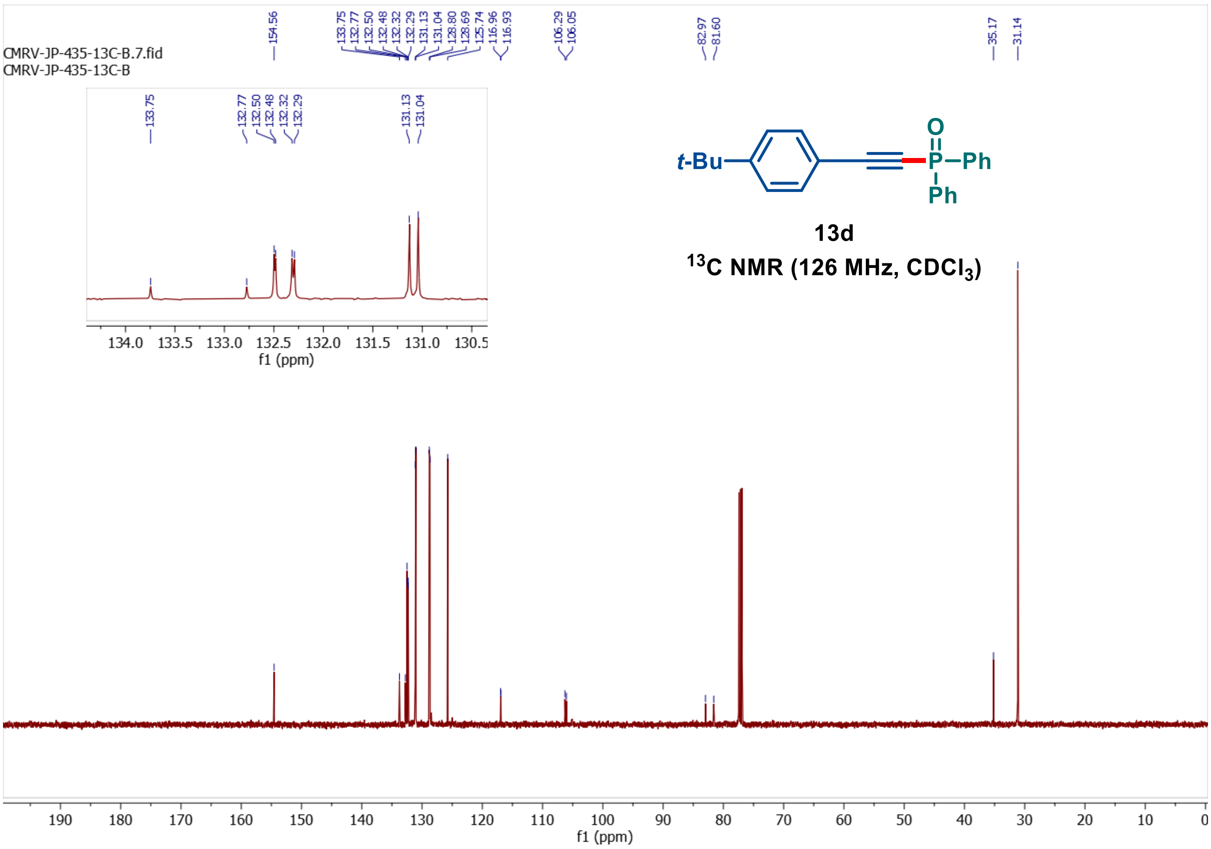
CMRV-JP-435-13C-B.7.fid
CMRV-JP-435-13C-B
154.56
133.75
132.77
132.50
132.48
132.32
132.29
131.13
131.04
128.80
128.69
125.74
116.96
116.93
106.29
106.05
82.97
81.60
35.17
31.14
t-Bu
O
P
Ph
Ph
13d
13C NMR (126 MHz, CDCl3)
f1 (ppm)

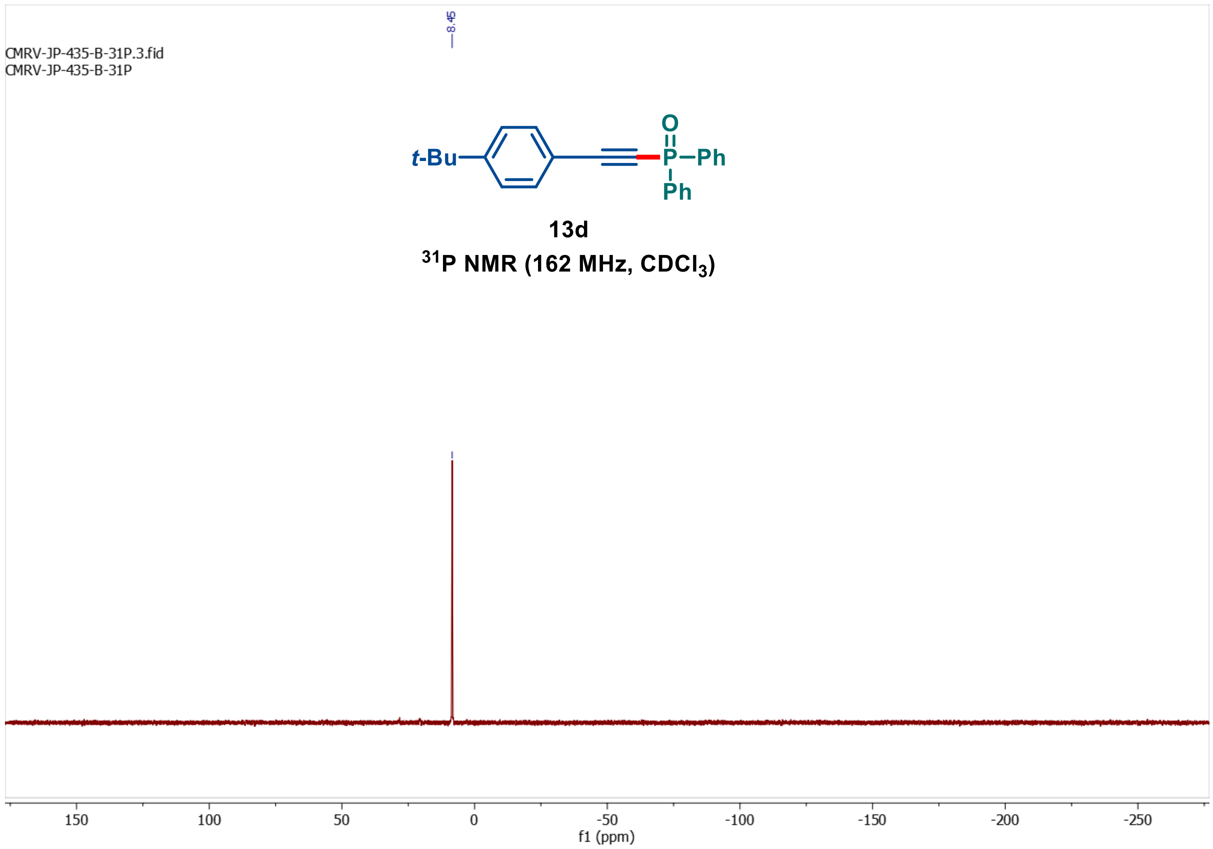

CMRV-JP-435-B-31P.3.fid
CMRV-JP-435-B-31P
8.45
t-Bu
O
P
Ph
Ph
13d
31P NMR (162 MHz, CDCl3)
150
100
50
0
-50
-100
-150
-200
-250
f1 (ppm)

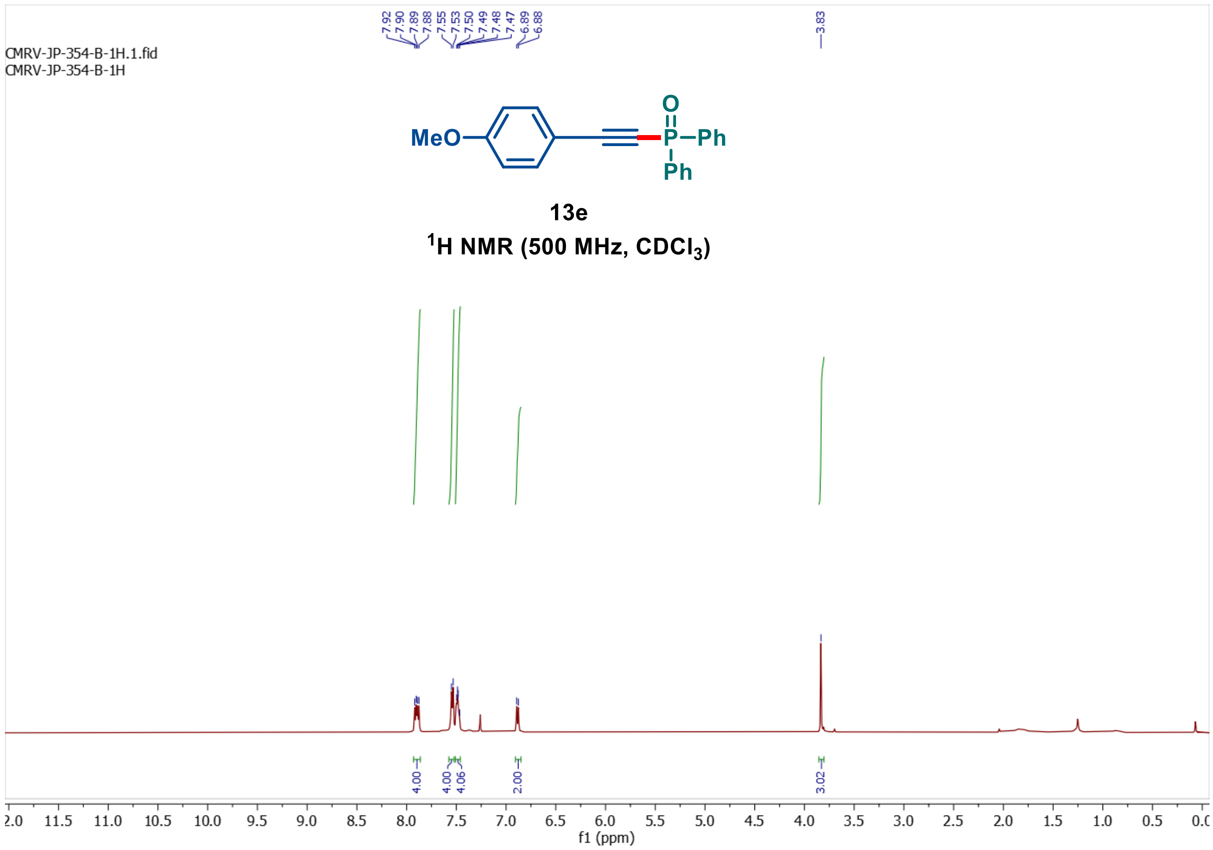

CMRV-JP-354-B-1H.1.fid
CMRV-JP-354-B-1H
7.92
7.90
7.89
7.88
7.55
7.53
7.50
7.49
7.48
7.47
6.89
6.88
3.83
MeO
O
P
Ph
Ph
13e
1H NMR (500 MHz, CDCl3)
4.00
4.00
4.06
2.00
3.02
11.5
11.0
10.5
10.0
9.5
9.0
8.5
8.0
7.5
7.0
6.5
6.0
5.5
5.0
4.5
4.0
3.5
3.0
2.5
2.0
1.5
1.0
0.5
f1 (ppm)

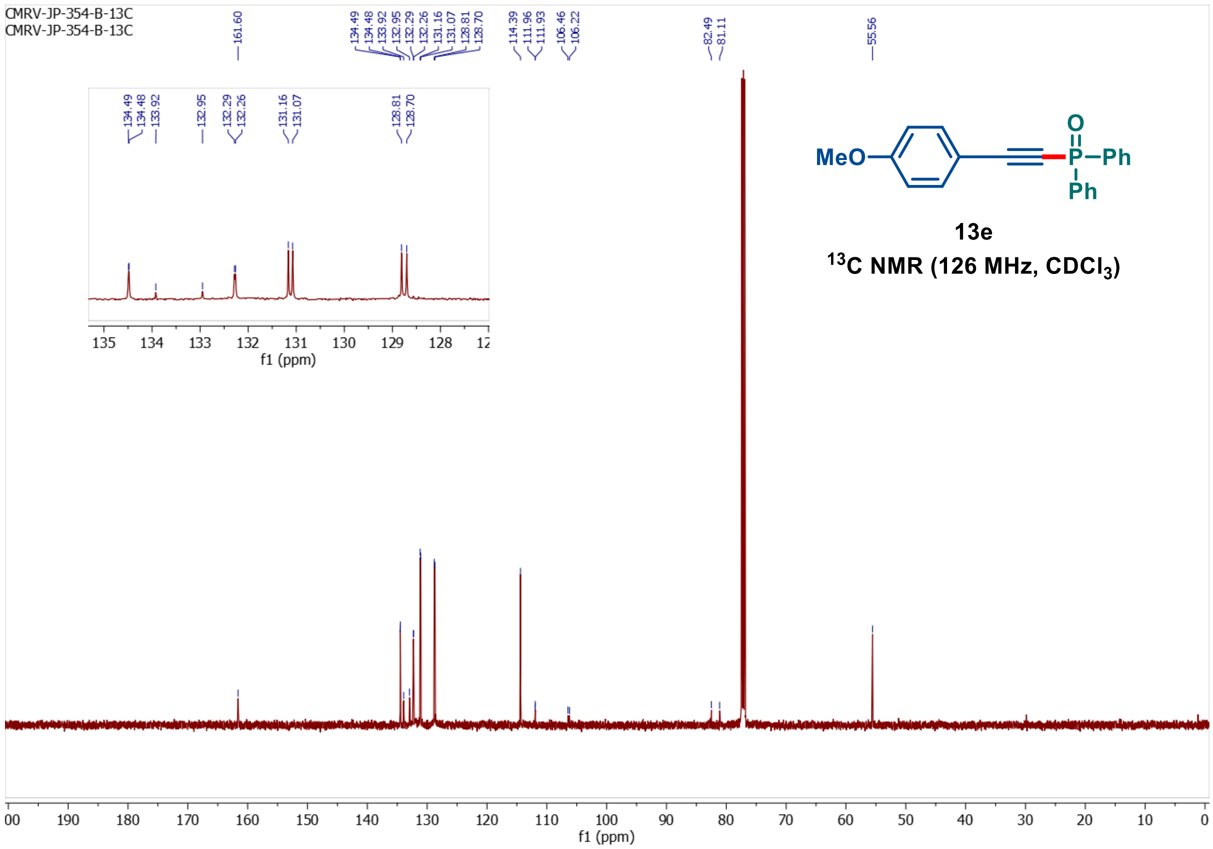
CMRV-JP-354-B-13C
CMRV-JP-354-B-13C
161.60
134.49
134.48
133.92
132.95
132.29
132.26
131.16
131.07
128.81
128.70
114.39
111.96
111.93
106.46
106.22
82.49
81.11
55.56
MeO
O
P
Ph
Ph
13e
13C NMR (126 MHz, CDCl3)
f1 (ppm)

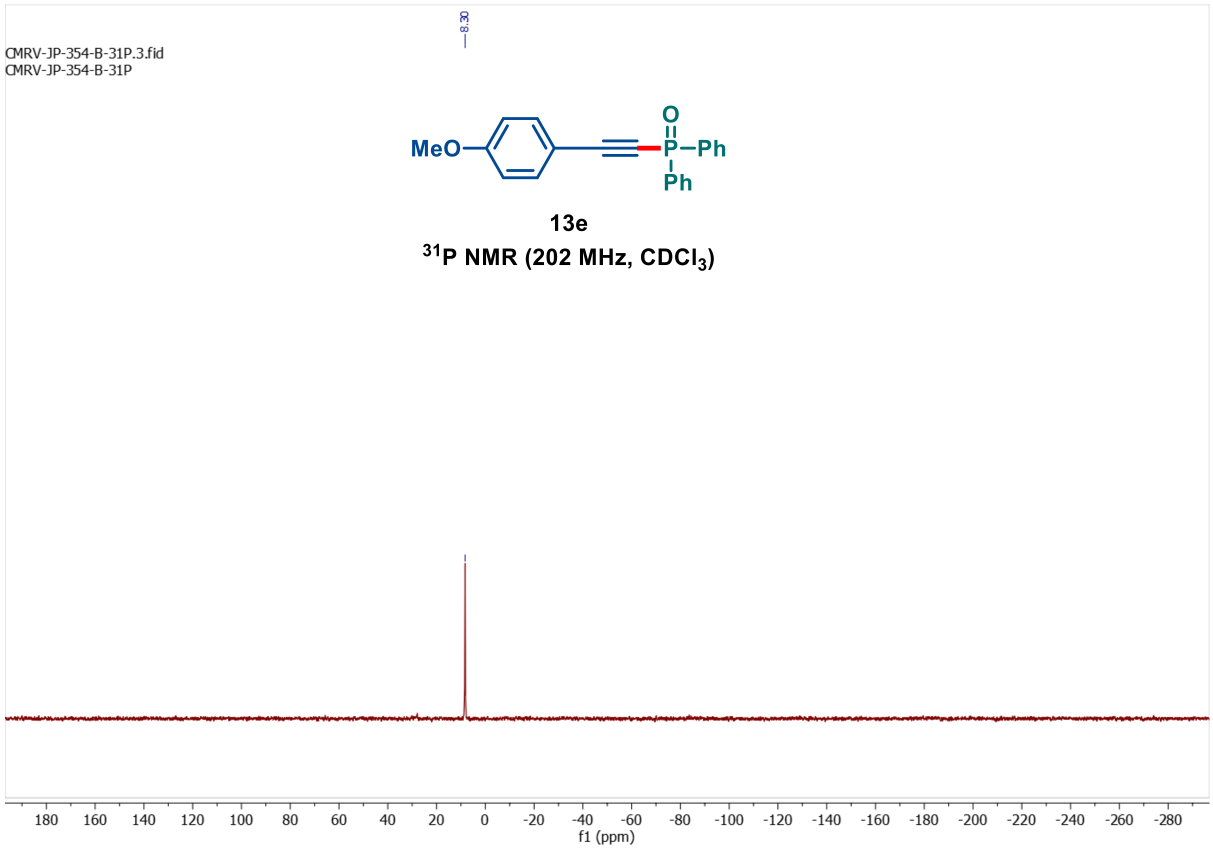
8.30
CMRV-JP-354-B-31P.3.fid
CMRV-JP-354-B-31P
MeO
O
P
Ph
Ph
13e
31P NMR (202 MHz, CDCl3)
f1 (ppm)

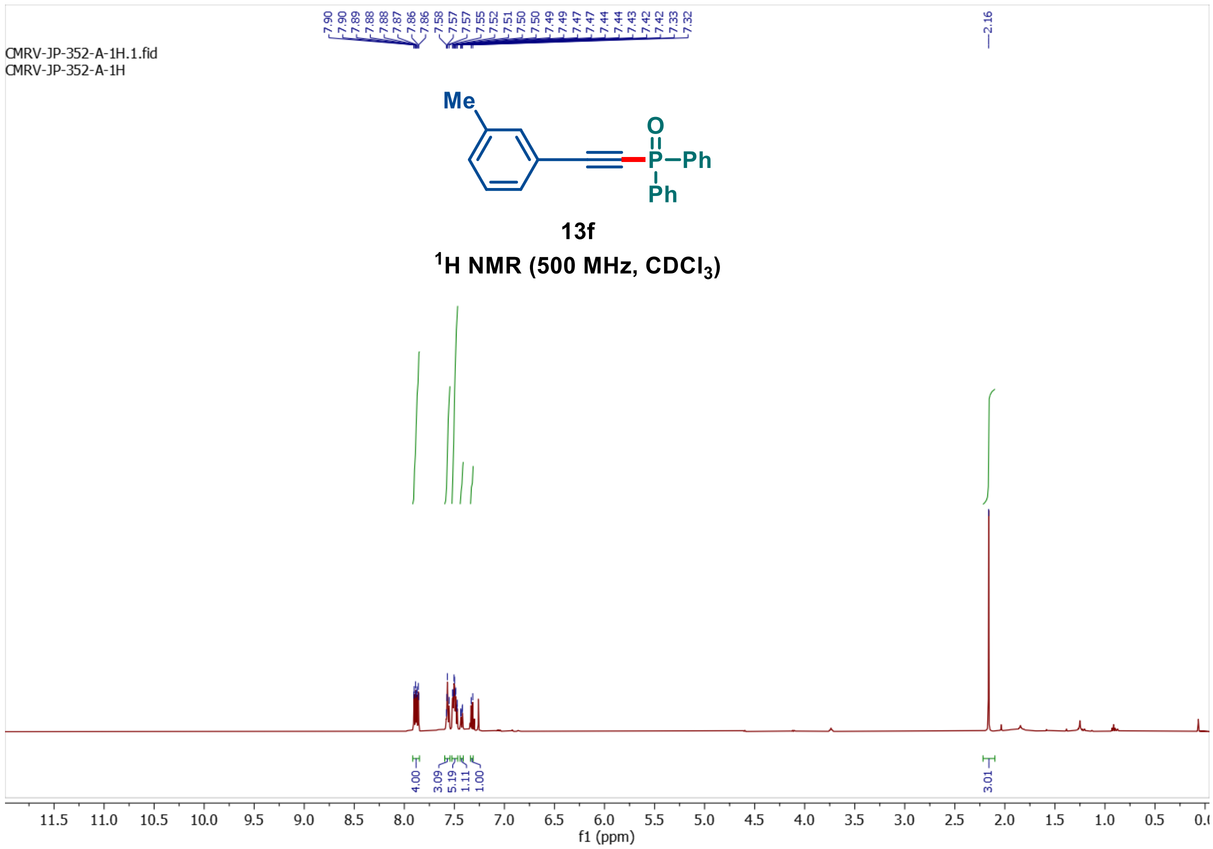
CMRV-JP-352-A-1H.1.fid
CMRV-JP-352-A-1H
7.90 7.90 7.89 7.88 7.88 7.87 7.86 7.86 7.58 7.57 7.57 7.55 7.52 7.51 7.50 7.50 7.49 7.49 7.47 7.44 7.44 7.43 7.42 7.42 7.33 7.32
2.16
Me
O
P
Ph
Ph
13f
1H NMR (500 MHz, CDCl3)
4.00 3.09 5.19 1.11 1.00 3.01
f1 (ppm)

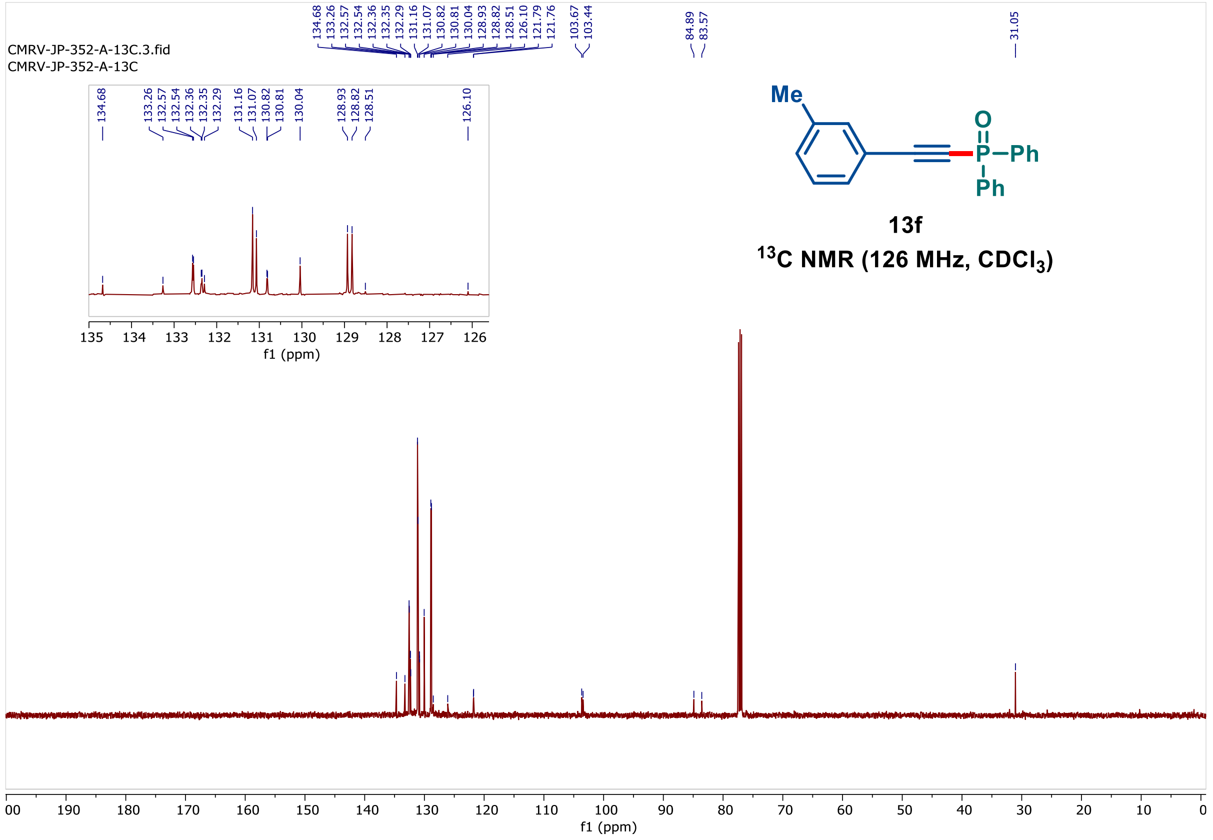
CMRV-JP-352-A-13C.3.fid
CMRV-JP-352-A-13C
134.68 133.26 132.57 132.54 132.36 132.35 132.29 131.16 131.07 130.82 130.81 130.04 128.93 128.82 128.51 126.10 121.79 121.76 103.67 103.44
84.89 83.57
31.05
Me
O
P
Ph
Ph
13f
13C NMR (126 MHz, CDCl3)
f1 (ppm)

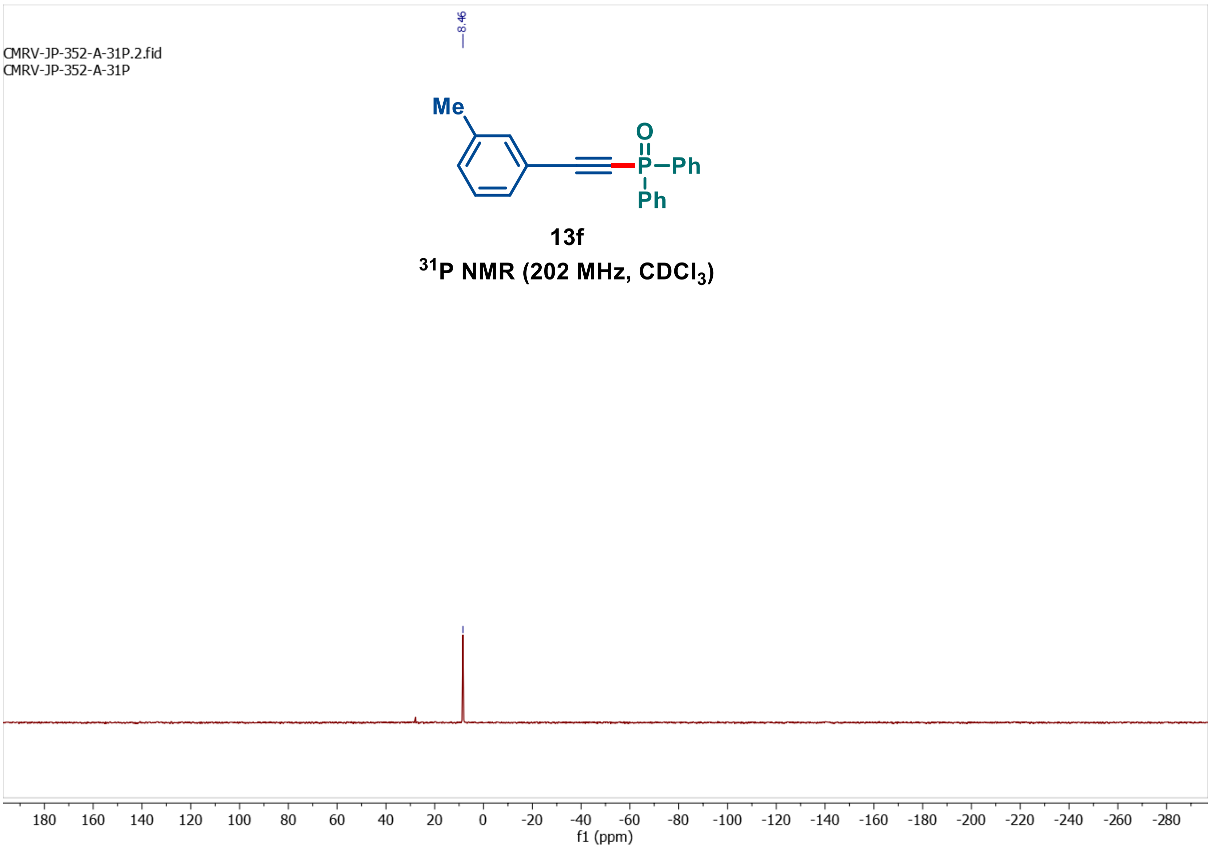
8.46
CMRV-JP-352-A-31P.2.fid
CMRV-JP-352-A-31P
Me
O
P–Ph
Ph
13f
31P NMR (202 MHz, CDCl3)
f1 (ppm)

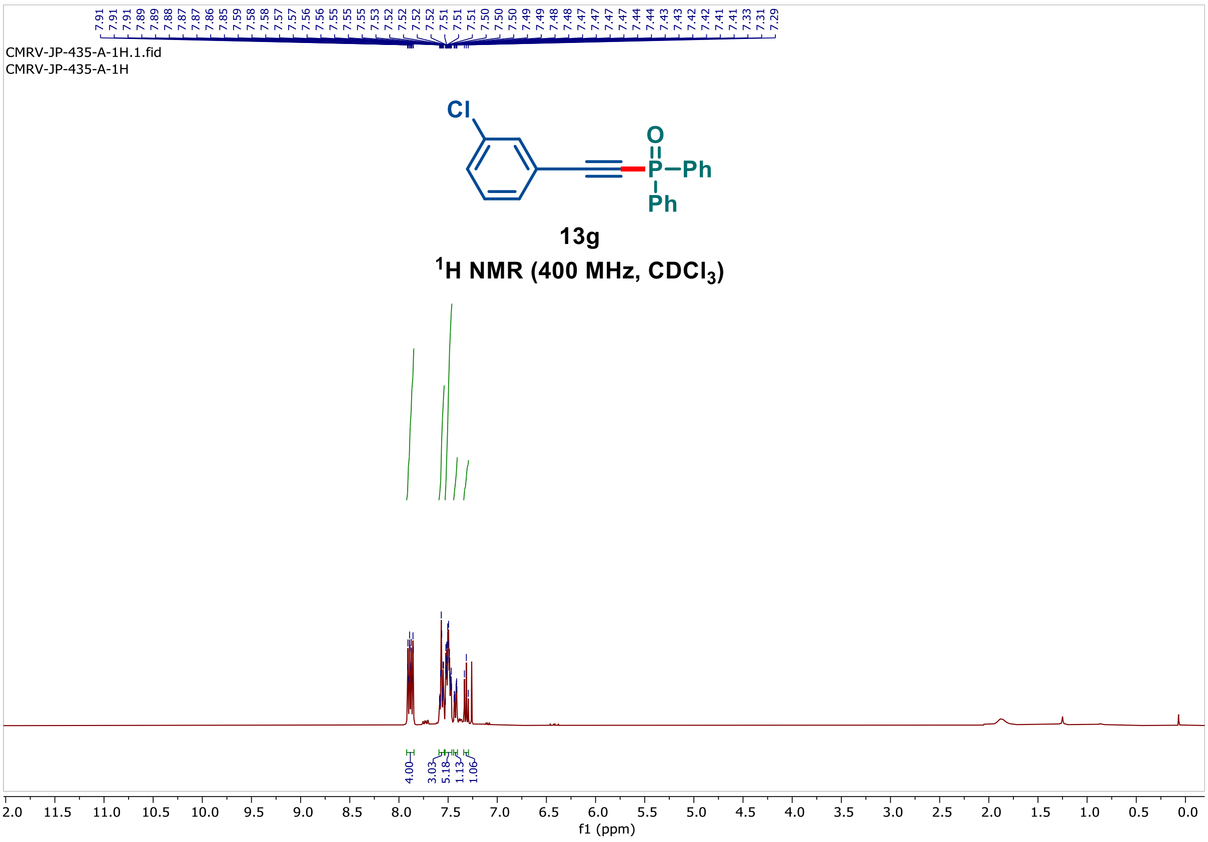
7.91 7.91 7.91 7.89 7.88 7.87 7.87 7.86 7.85 7.59 7.58 7.58 7.57 7.56 7.56 7.55 7.55 7.53 7.52 7.52 7.52 7.51 7.51 7.51 7.50 7.50 7.49 7.48 7.48 7.47 7.47 7.47 7.44 7.44 7.43 7.43 7.42 7.42 7.41 7.41 7.33 7.31 7.29
CMRV-JP-435-A-1H.1.fid
CMRV-JP-435-A-1H
Cl
O
P–Ph
Ph
13g
1H NMR (400 MHz, CDCl3)
4.00
3.03
5.18
1.13
1.06
f1 (ppm)

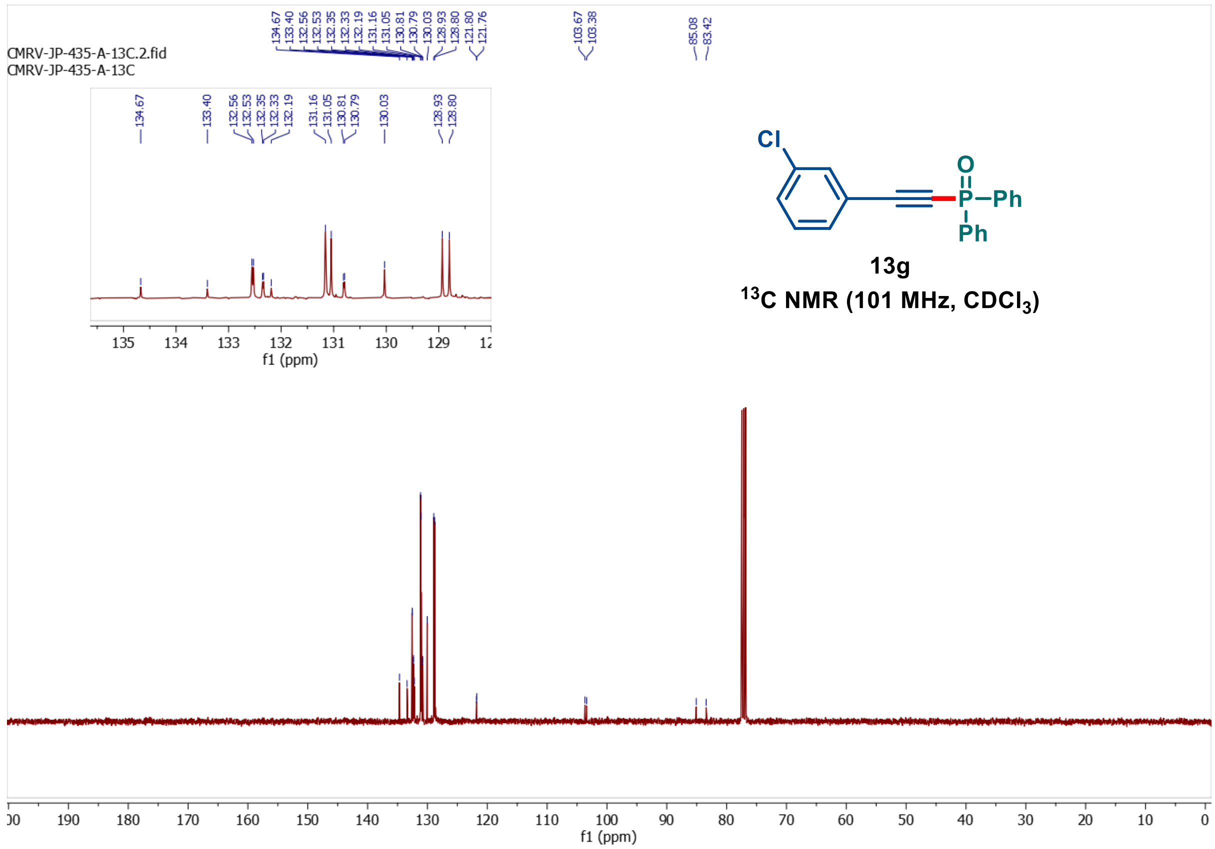
CMRV-JP-435-A-13C.2.fid
CMRV-JP-435-A-13C
134.67
133.40
132.56
132.53
132.35
132.33
132.19
131.16
131.05
130.81
130.79
130.03
128.93
128.80
121.80
121.76
103.67
103.38
85.08
83.42
Cl
O
P–Ph
Ph
13g
13C NMR (101 MHz, CDCl3)
f1 (ppm)

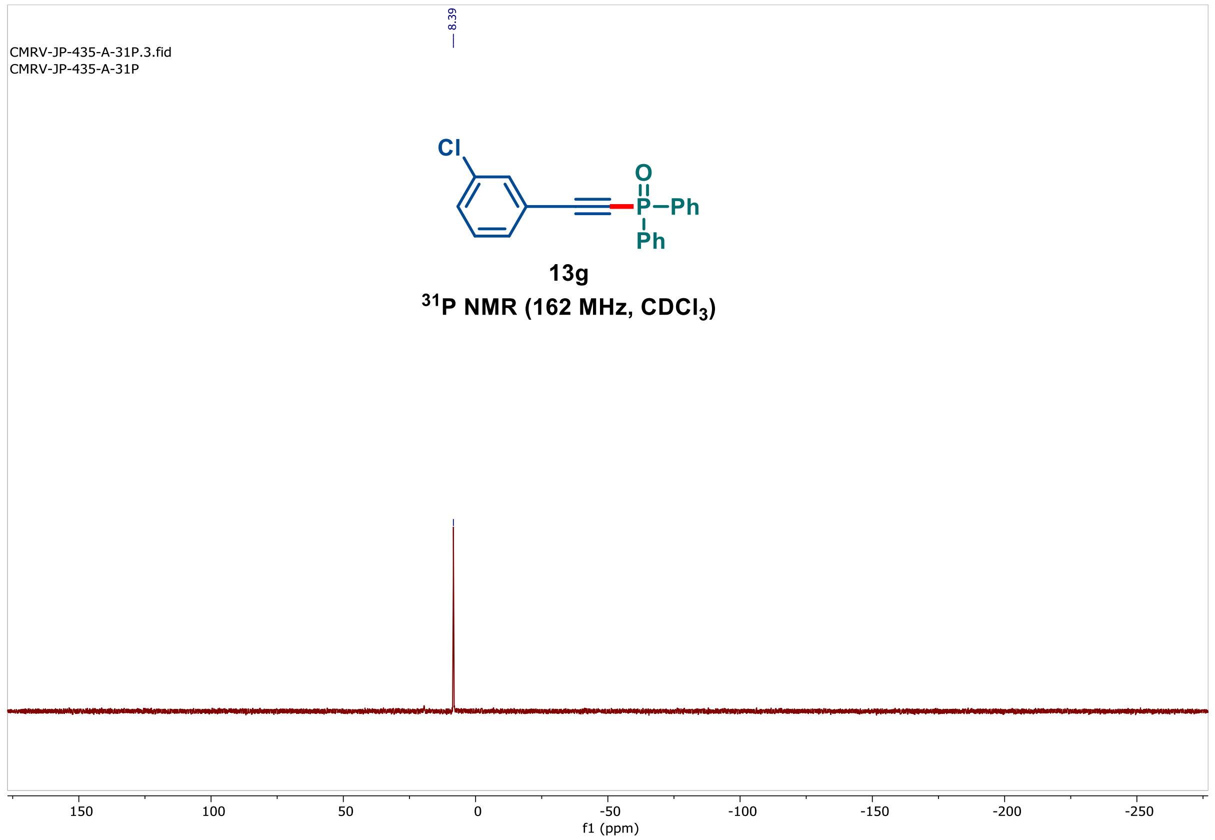
CMRV-JP-435-A-31P.3.fid
CMRV-JP-435-A-31P
8.39
Cl
O
P–Ph
Ph
13g
31P NMR (162 MHz, CDCl3)
f1 (ppm)

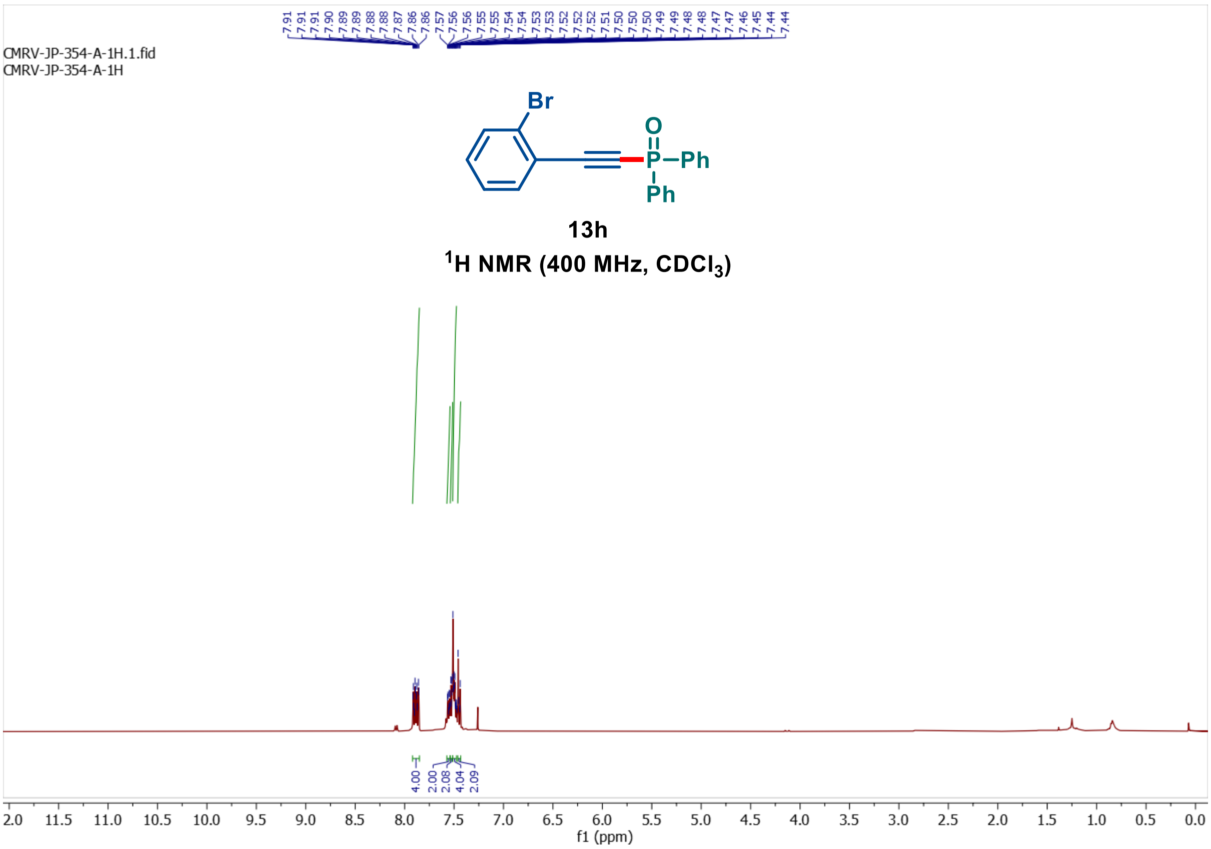
CMRV-JP-354-A-1H.1.fid
CMRV-JP-354-A-1H
7.91 7.91 7.91 7.90 7.89 7.89 7.88 7.88 7.87 7.86 7.86 7.57 7.56 7.56 7.55 7.55 7.54 7.54 7.53 7.53 7.52 7.52 7.52 7.51 7.50 7.50 7.50 7.49 7.49 7.48 7.48 7.47 7.47 7.46 7.45 7.44 7.44
Br
O
P
Ph
Ph
13h
^{1}H NMR (400 MHz, CDCl$_3$)
4.00 2.00 2.08 4.04 2.09
f1 (ppm)

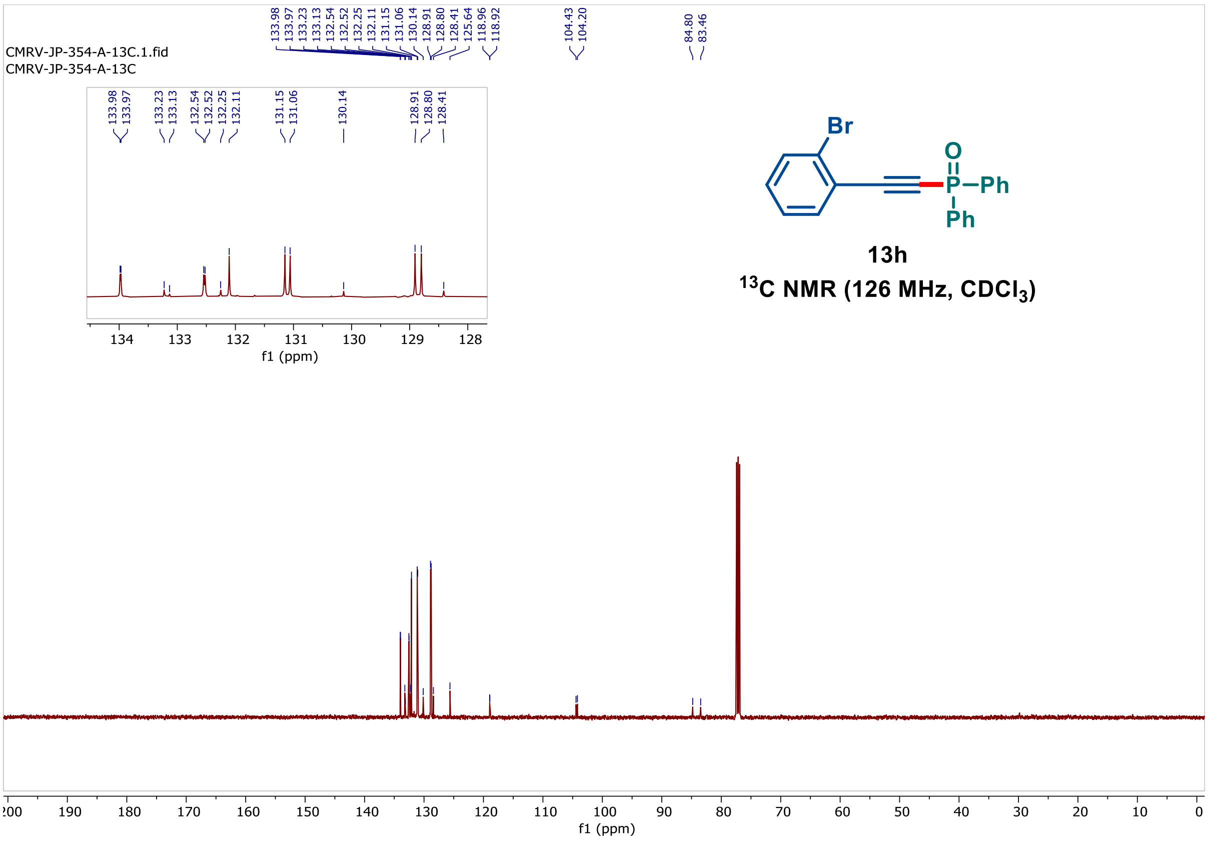
CMRV-JP-354-A-13C.1.fid
CMRV-JP-354-A-13C
133.98 133.97 133.23 133.13 132.54 132.52 132.25 132.11 131.15 131.06 130.14 128.91 128.80 128.41 125.64 118.96 118.92 104.43 104.20 84.80 83.46
Br
O
P
Ph
Ph
13h
^{13}C NMR (126 MHz, CDCl$_3$)
f1 (ppm)

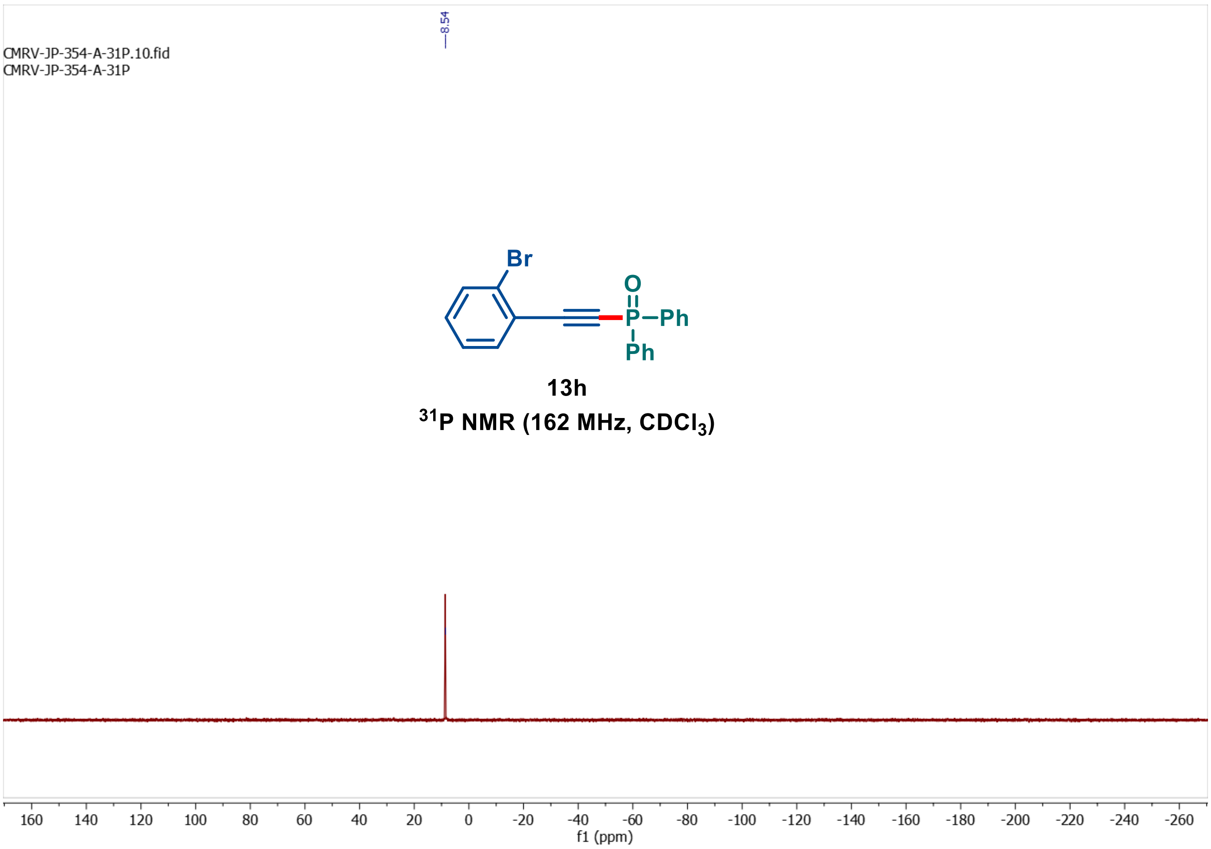
CMRV-JP-354-A-31P.10.fid
CMRV-JP-354-A-31P
8.54
Br
O
P
Ph
Ph
13h
31P NMR (162 MHz, CDCl3)
160 140 120 100 80 60 40 20 0 -20 -40 -60 -80 -100 -120 -140 -160 -180 -200 -220 -240 -260
f1 (ppm)

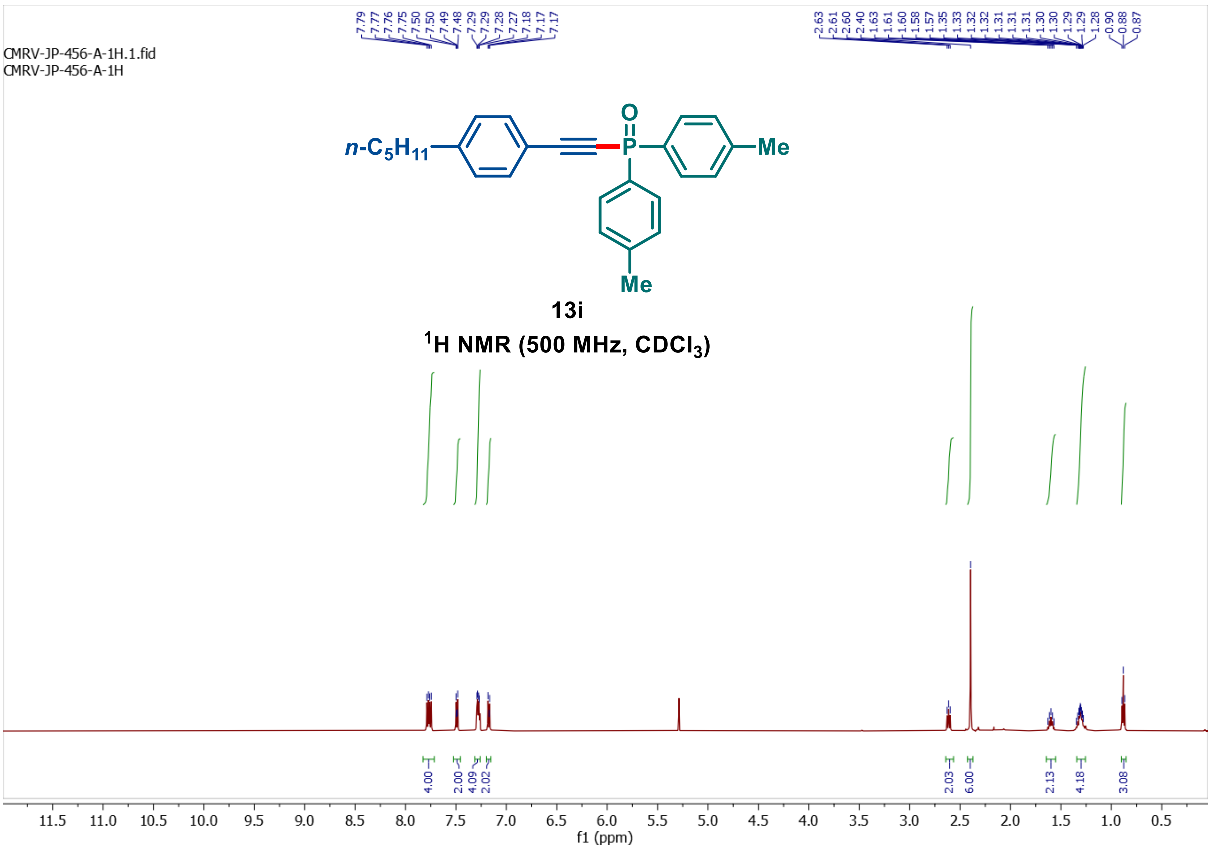
CMRV-JP-456-A-1H.1.fid
CMRV-JP-456-A-1H
7.79 7.77 7.76 7.75 7.50 7.49 7.48 7.29 7.29 7.28 7.27 7.18 7.17 7.17
2.63 2.61 2.60 2.40 1.63 1.61 1.60 1.58 1.57 1.35 1.33 1.32 1.32 1.31 1.31 1.30 1.30 1.29 1.29 1.28 0.90 0.88 0.87
n-C5H11
O
P
Me
Me
13i
1H NMR (500 MHz, CDCl3)
4.00 2.00 4.09 2.02 2.03 6.00 2.13 4.18 3.08
11.5 11.0 10.5 10.0 9.5 9.0 8.5 8.0 7.5 7.0 6.5 6.0 5.5 5.0 4.5 4.0 3.5 3.0 2.5 2.0 1.5 1.0 0.5
f1 (ppm)

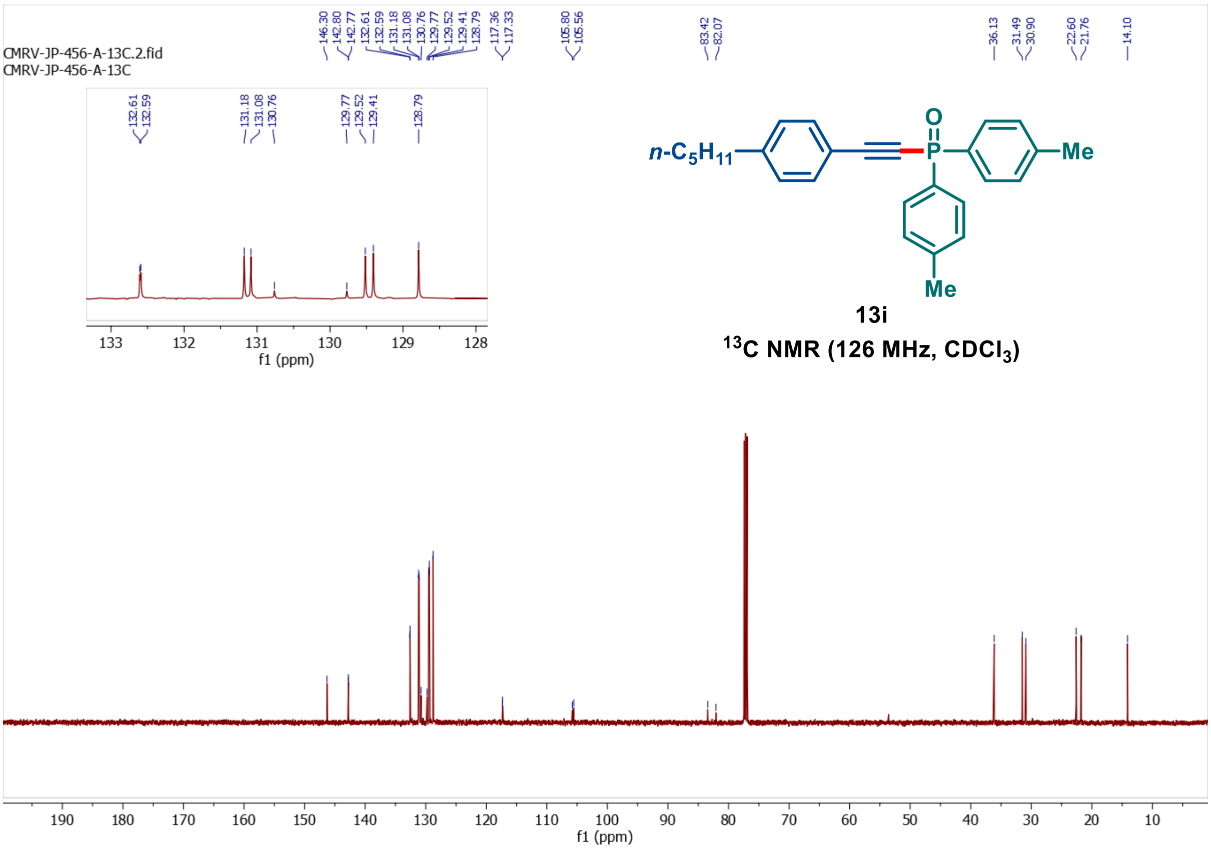

13i

^{13}C NMR (126 MHz, $CDCl_3$)

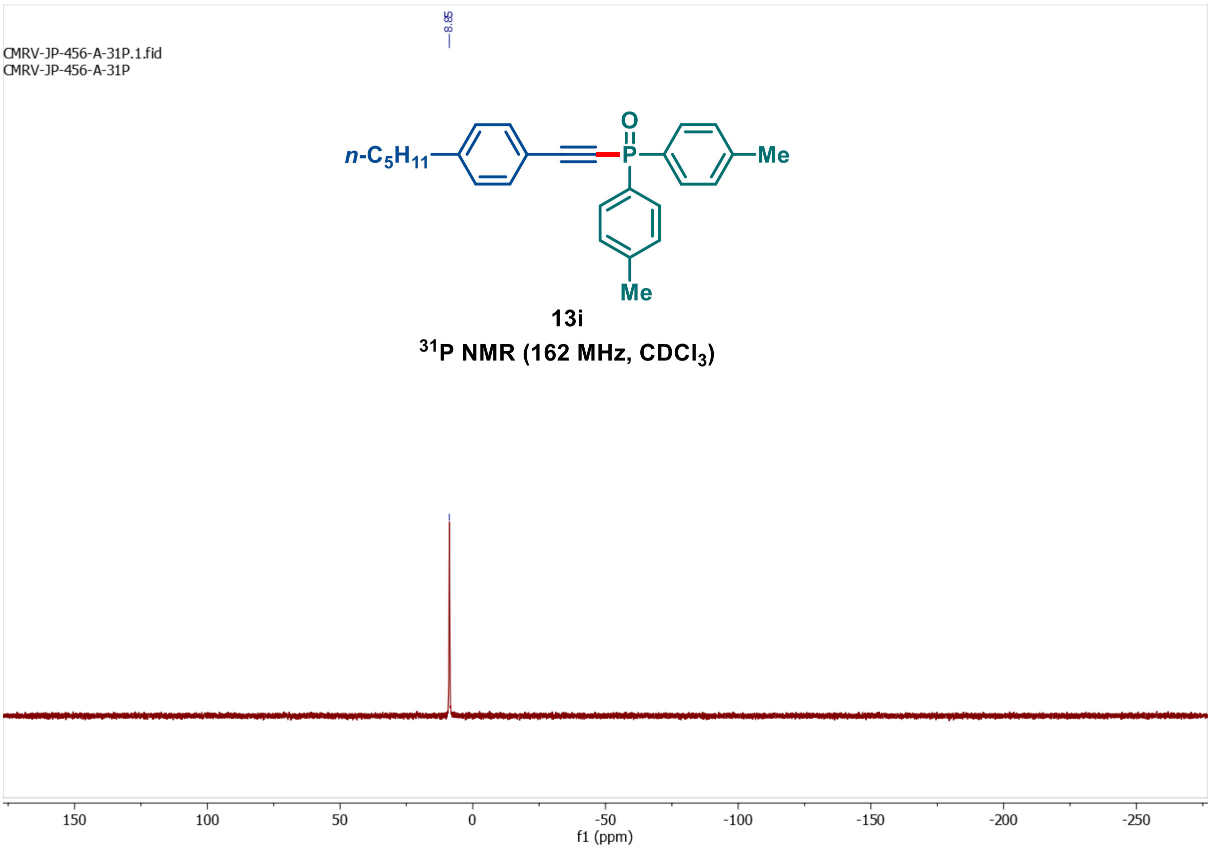

13i

^{31}P NMR (162 MHz, $CDCl_3$)

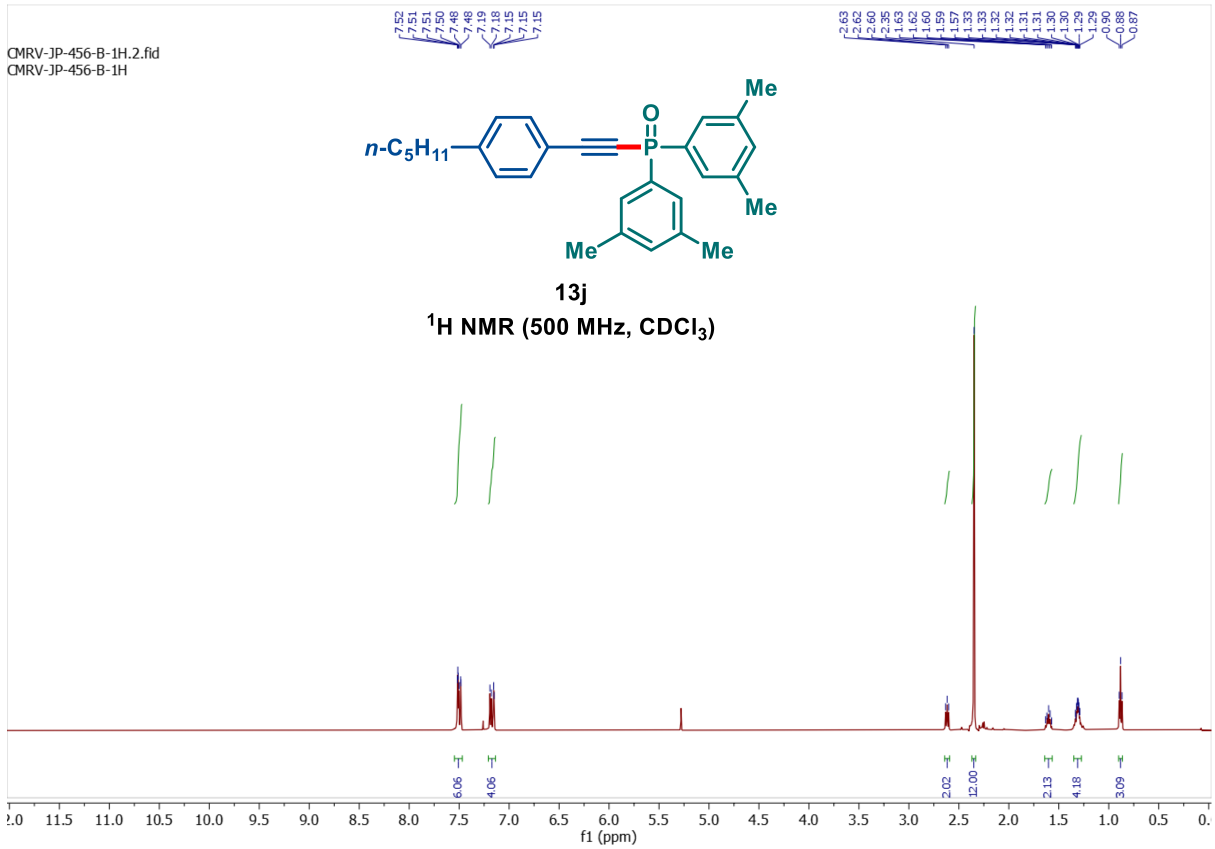
CMRV-JP-456-B-1H.2.fid
CMRV-JP-456-B-1H
13j
^{1}H NMR (500 MHz, $CDCl_3$)
f1 (ppm)

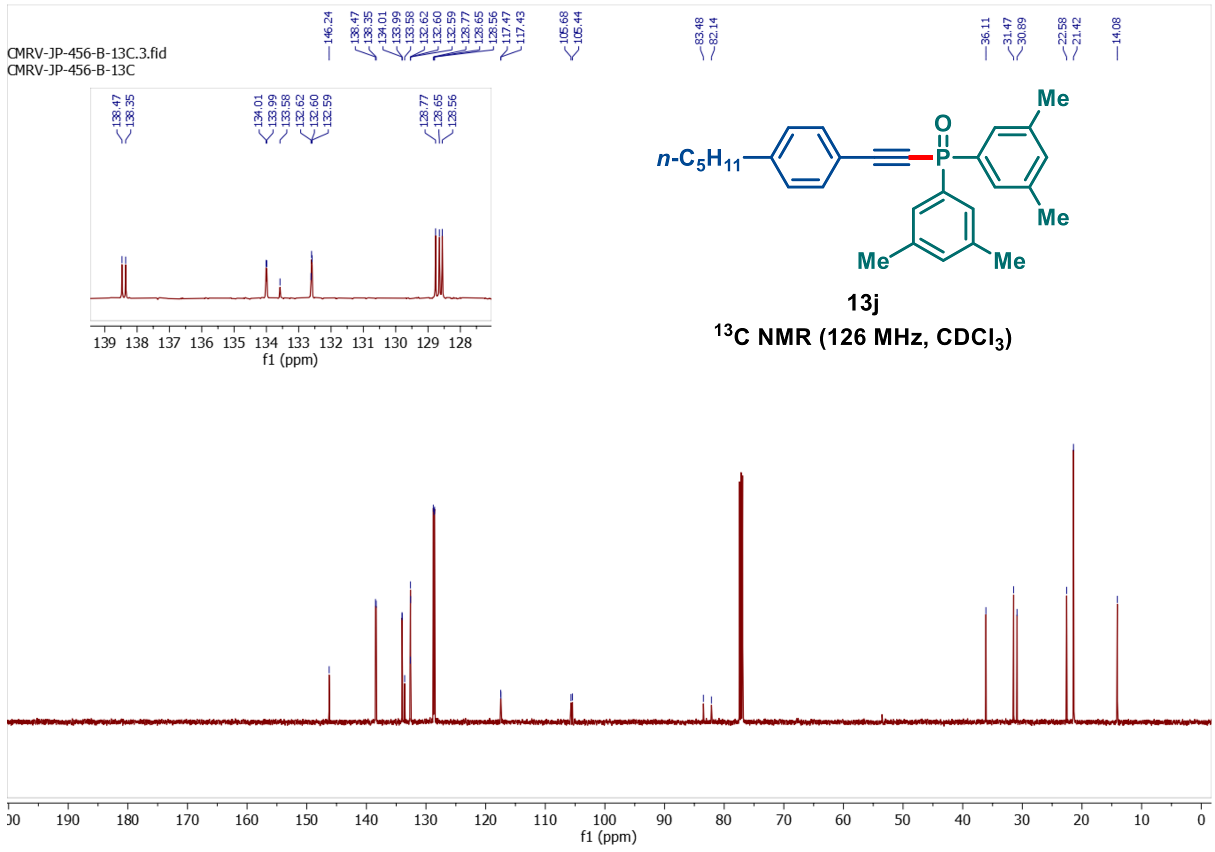
CMRV-JP-456-B-13C.3.fid
CMRV-JP-456-B-13C
13j
^{13}C NMR (126 MHz, $CDCl_3$)
f1 (ppm)

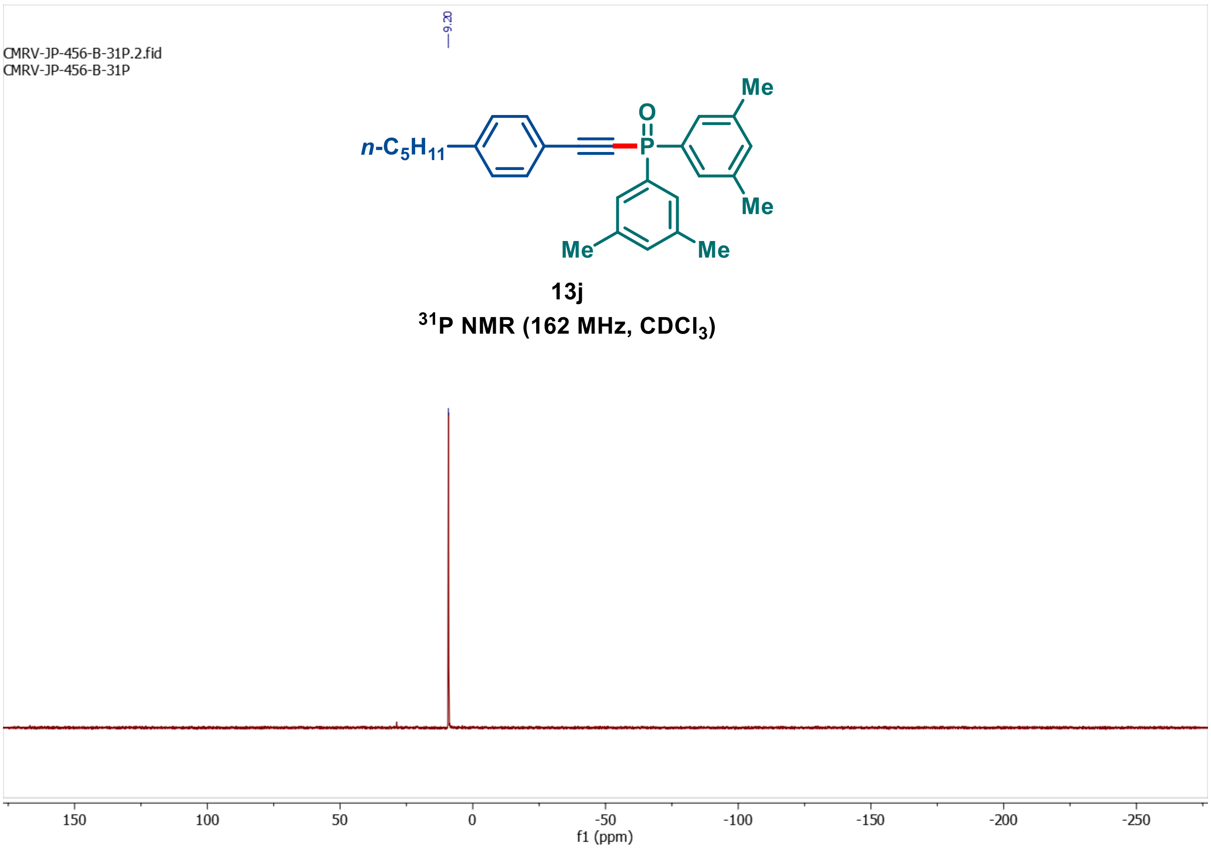
9.20
CMRV-JP-456-B-31P.2.fid
CMRV-JP-456-B-31P
n-C5H11
O
P
Me
Me
Me
Me
13j
31P NMR (162 MHz, CDCl3)
150
100
50
0
-50
-100
-150
-200
-250
f1 (ppm)

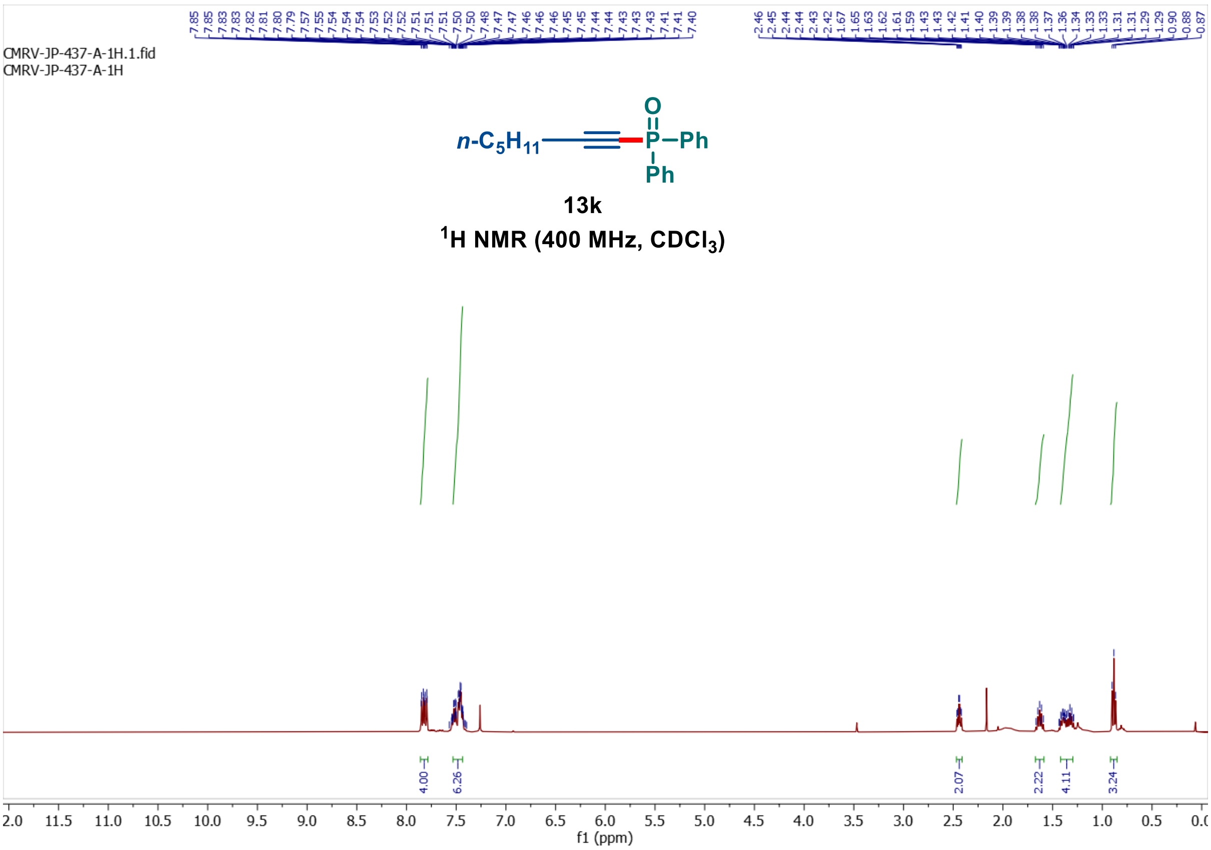
7.85 7.85 7.83 7.83 7.82 7.81 7.80 7.79 7.57 7.55 7.54 7.54 7.54 7.53 7.52 7.52 7.51 7.51 7.51 7.50 7.50 7.48 7.47 7.46 7.46 7.46 7.45 7.44 7.44 7.43 7.43 7.43 7.41 7.41 7.40
2.46 2.45 2.44 2.44 2.43 2.42 1.67 1.65 1.63 1.62 1.61 1.59 1.43 1.43 1.42 1.41 1.40 1.39 1.39 1.38 1.38 1.37 1.36 1.34 1.33 1.33 1.31 1.31 1.29 0.90 0.88 0.87
CMRV-JP-437-A-1H.1.fid
CMRV-JP-437-A-1H
n-C5H11
O
P–Ph
Ph
13k
1H NMR (400 MHz, CDCl3)
4.00
6.26
2.07
2.22
4.11
3.24
f1 (ppm)

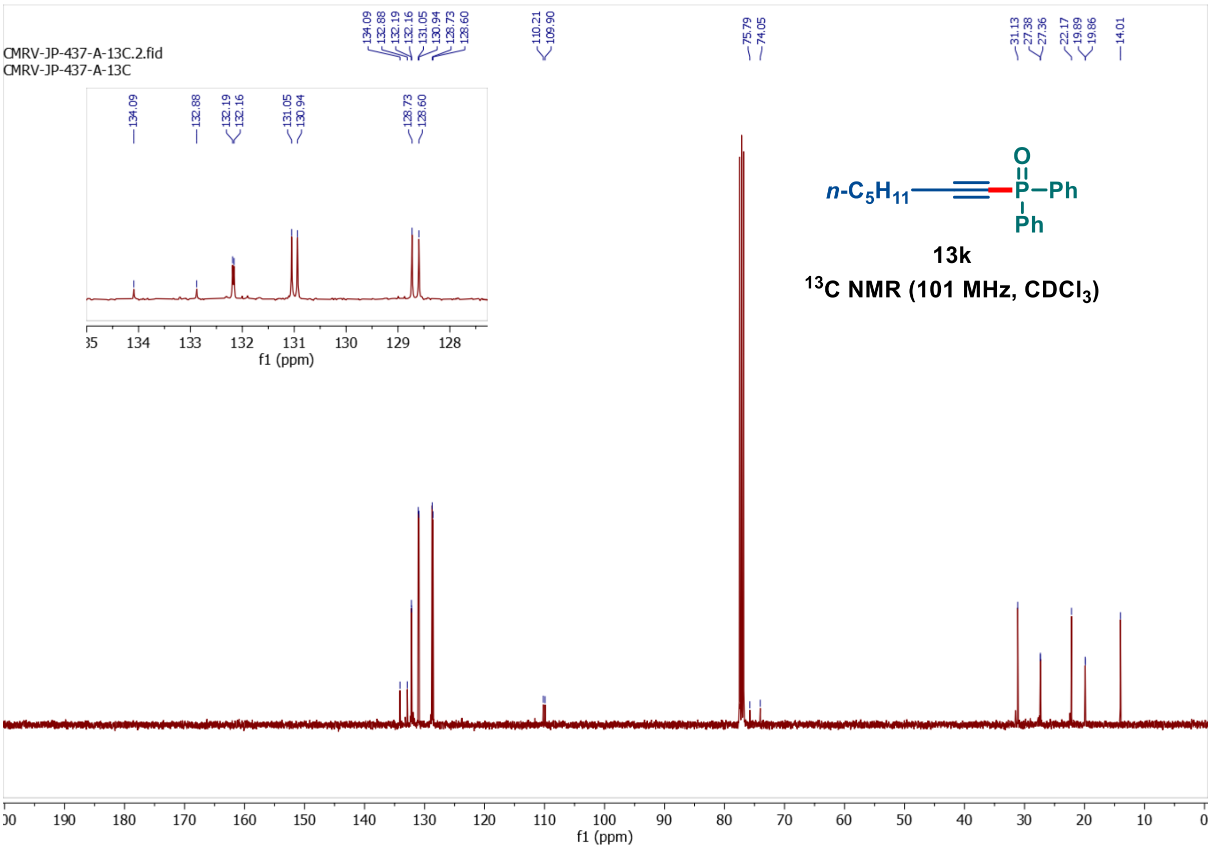
CMRV-JP-437-A-13C.2.fid
CMRV-JP-437-A-13C
134.09
132.88
132.19
132.16
131.05
130.94
128.73
128.60
110.21
109.90
75.79
74.05
31.13
27.38
27.36
22.17
19.89
19.86
14.01
n-C5H11
O
P
Ph
Ph
13k
13C NMR (101 MHz, CDCl3)
f1 (ppm)

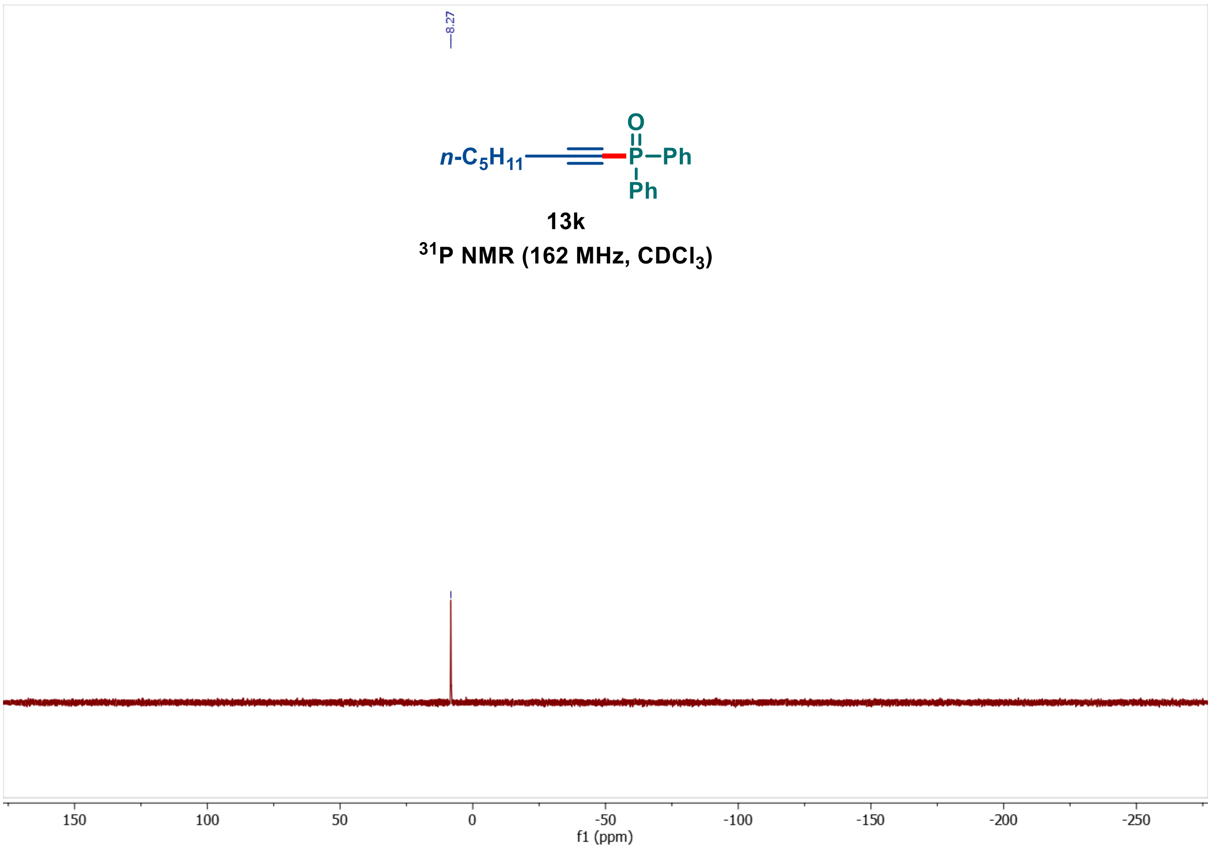
8.27
n-C5H11
O
P
Ph
Ph
13k
31P NMR (162 MHz, CDCl3)
f1 (ppm)

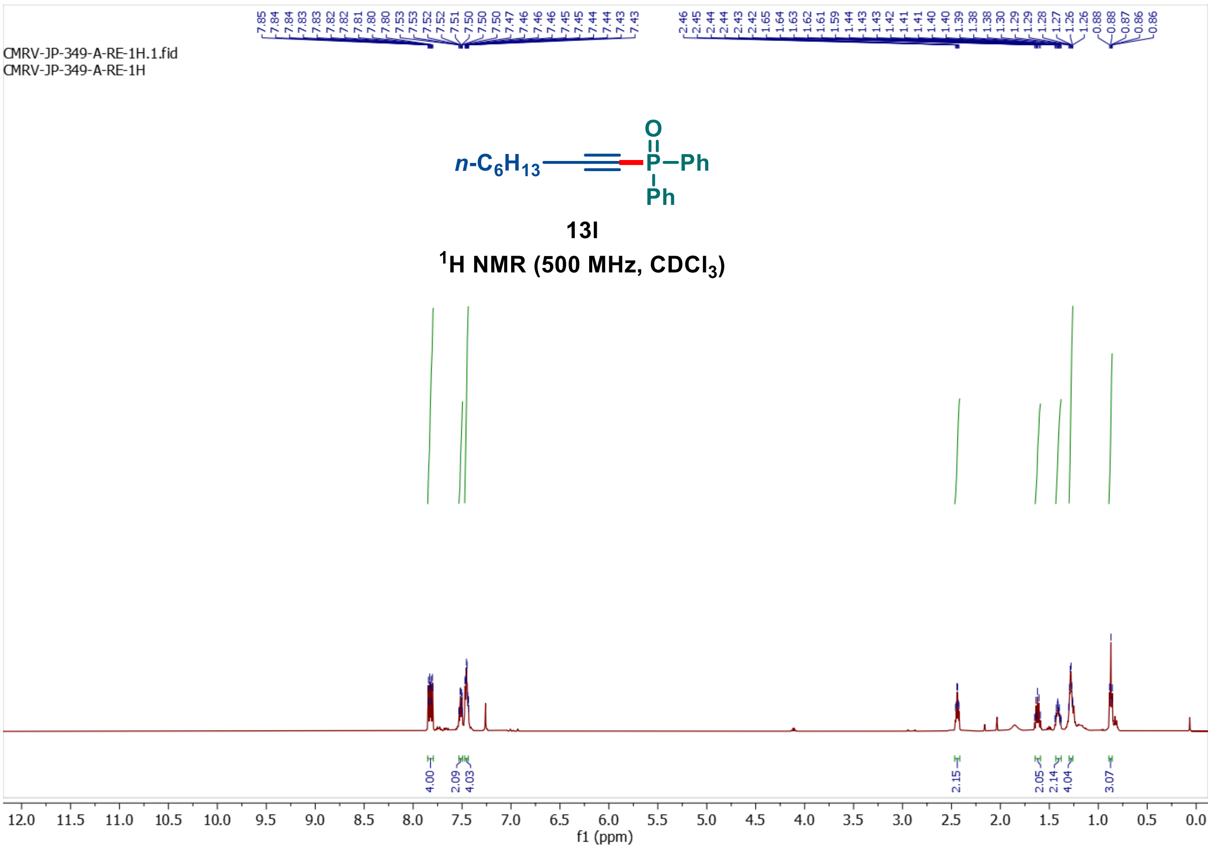

CMRV-JP-349-A-RE-1H.1.fid
CMRV-JP-349-A-RE-1H
n-C6H13
O
P
Ph
Ph
13l
1H NMR (500 MHz, CDCl3)
7.85
7.84
7.84
7.83
7.83
7.82
7.82
7.81
7.80
7.80
7.53
7.53
7.52
7.52
7.51
7.50
7.50
7.50
7.47
7.46
7.46
7.45
7.45
7.44
7.43
7.43
2.46
2.45
2.44
2.44
2.43
2.42
1.65
1.64
1.63
1.62
1.61
1.59
1.44
1.43
1.43
1.42
1.41
1.41
1.40
1.39
1.38
1.38
1.30
1.29
1.29
1.28
1.27
1.26
0.88
0.88
0.87
0.86
0.86
4.00
2.09
4.03
2.15
2.05
2.14
4.04
3.07
f1 (ppm)

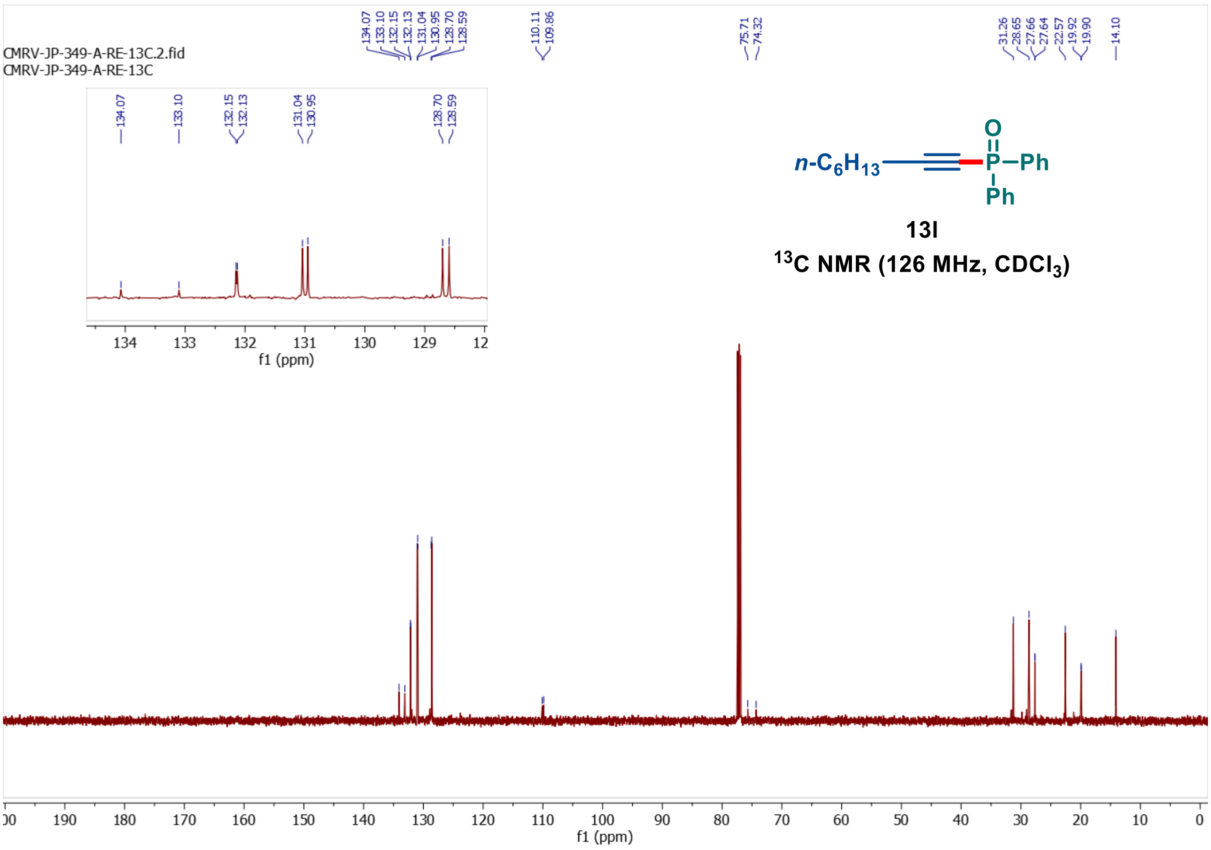

CMRV-JP-349-A-RE-13C.2.fid
CMRV-JP-349-A-RE-13C
134.07
133.10
132.15
132.13
131.04
130.95
128.70
128.59
110.11
109.86
75.71
74.32
31.26
28.65
27.66
27.64
22.57
19.92
19.90
14.10
n-C6H13
O
P
Ph
Ph
13l
13C NMR (126 MHz, CDCl3)
f1 (ppm)

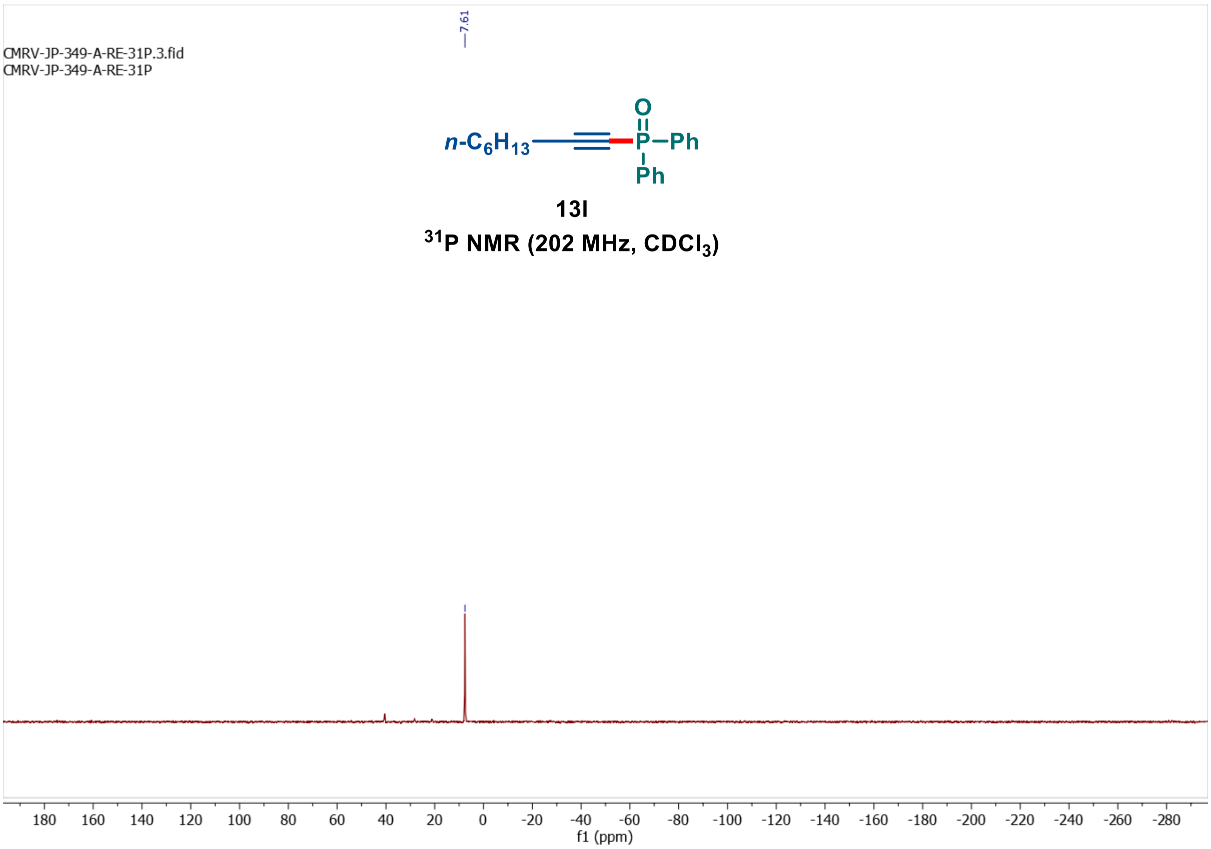
CMRV-JP-349-A-RE-31P.3.fid
CMRV-JP-349-A-RE-31P
7.61
n-C6H13
O
P
Ph
13l
31P NMR (202 MHz, CDCl3)
f1 (ppm)

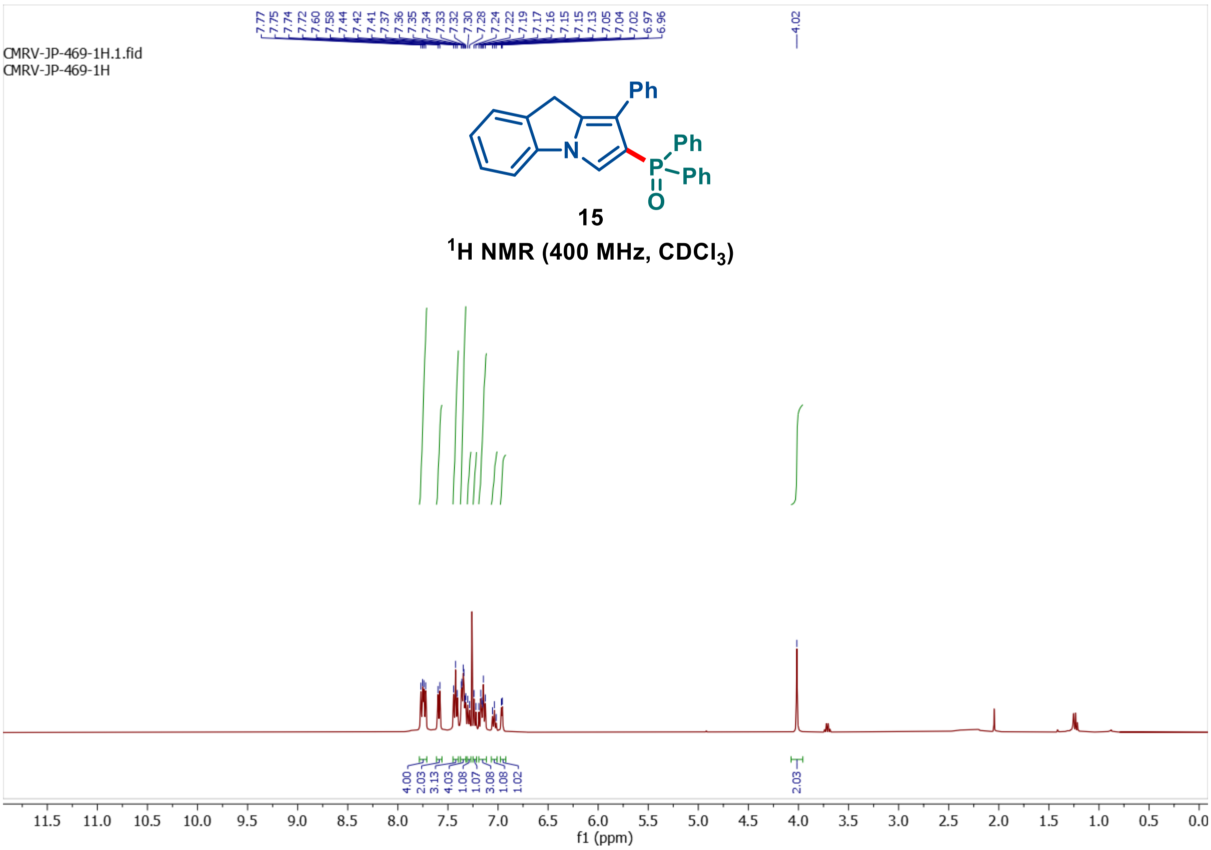
CMRV-JP-469-1H.1.fid
CMRV-JP-469-1H
7.77 7.75 7.74 7.72 7.60 7.58 7.44 7.42 7.41 7.37 7.36 7.35 7.34 7.33 7.32 7.30 7.28 7.24 7.22 7.19 7.17 7.16 7.15 7.15 7.13 7.05 7.04 7.02 6.97 6.96
4.02
Ph
N
P
O
15
1H NMR (400 MHz, CDCl3)
4.00 2.03 3.13 4.03 1.08 1.07 3.08 1.08 1.02
2.03
f1 (ppm)

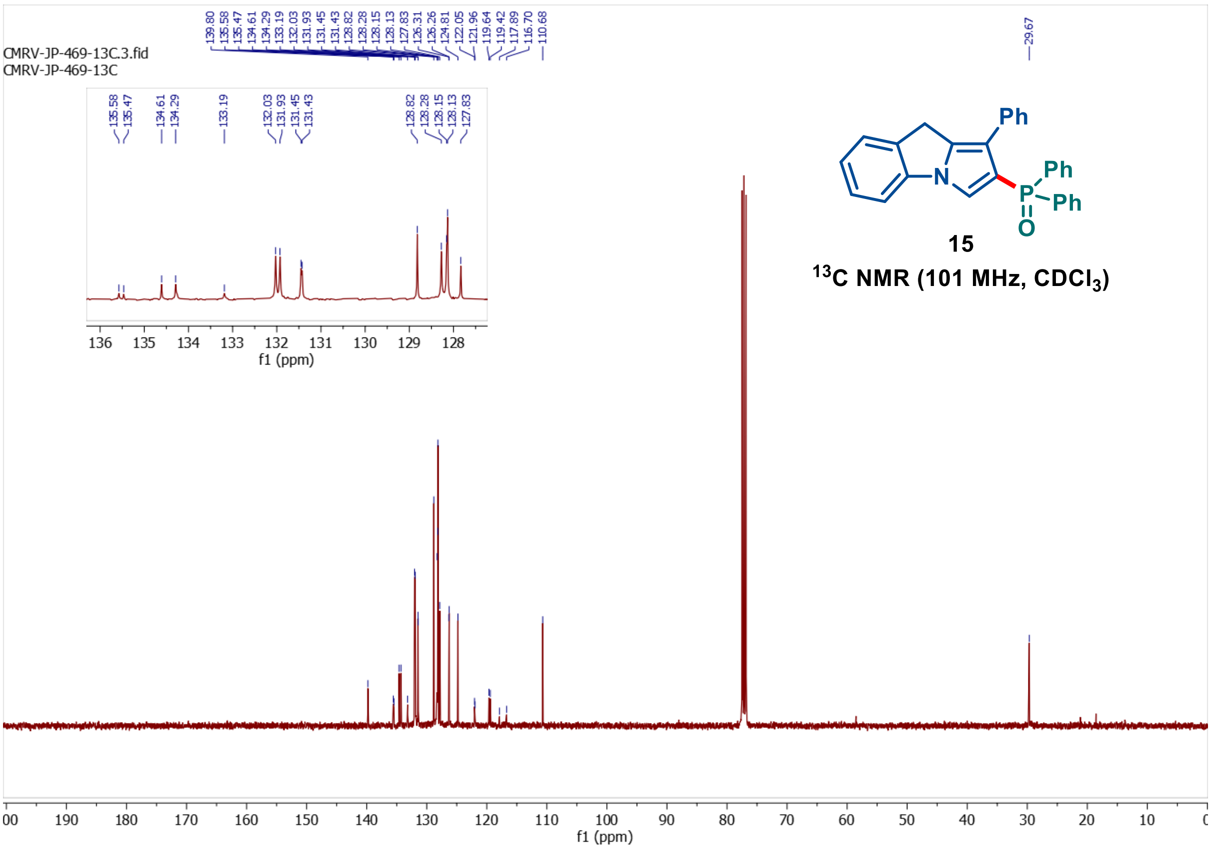
CMRV-JP-469-13C.3.fid
CMRV-JP-469-13C
139.80
135.58
135.47
134.61
134.29
133.19
132.03
131.93
131.45
131.43
128.82
128.28
128.15
128.13
127.83
126.31
126.26
124.81
122.05
121.96
119.64
119.42
117.89
116.70
110.68
29.67
Ph
N
P
Ph
Ph
O
15
13C NMR (101 MHz, CDCl3)
f1 (ppm)

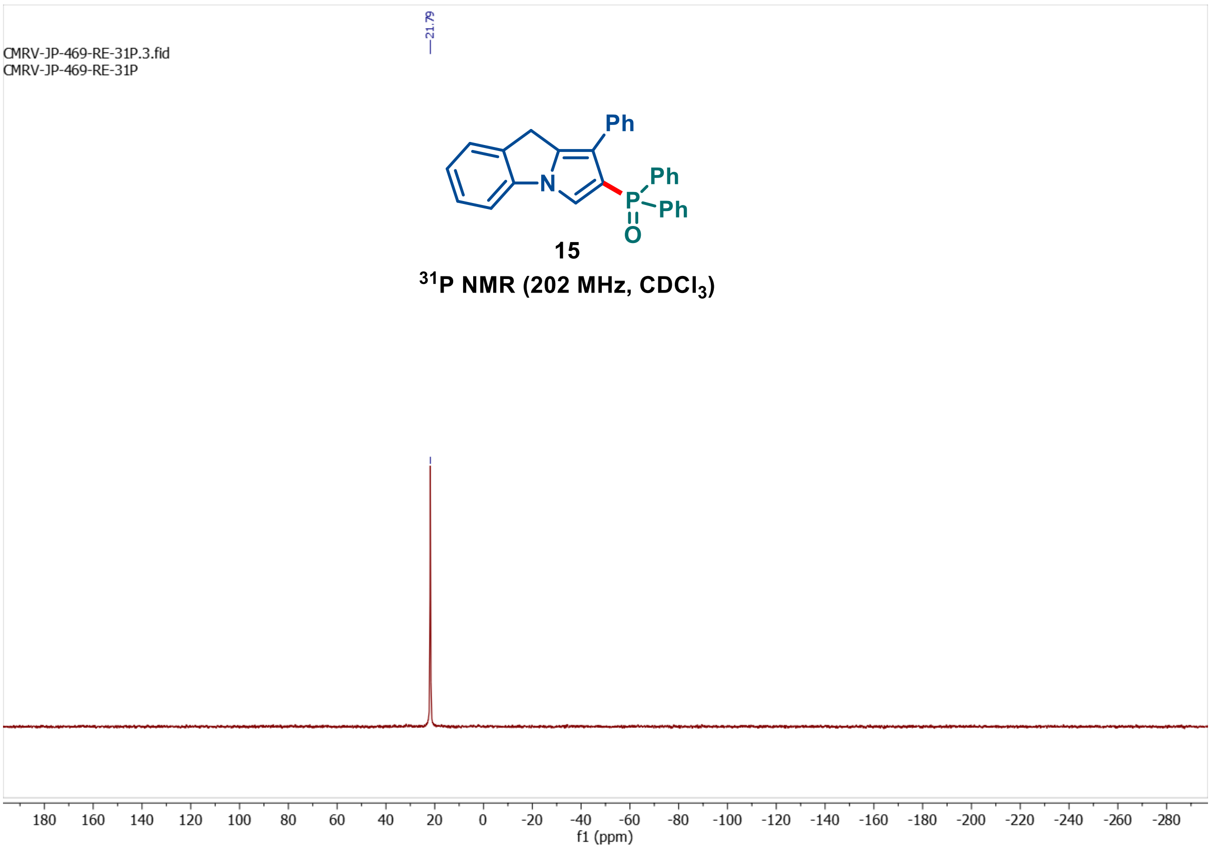
CMRV-JP-469-RE-31P.3.fid
CMRV-JP-469-RE-31P
21.79
Ph
N
P
Ph
Ph
O
15
31P NMR (202 MHz, CDCl3)
f1 (ppm)

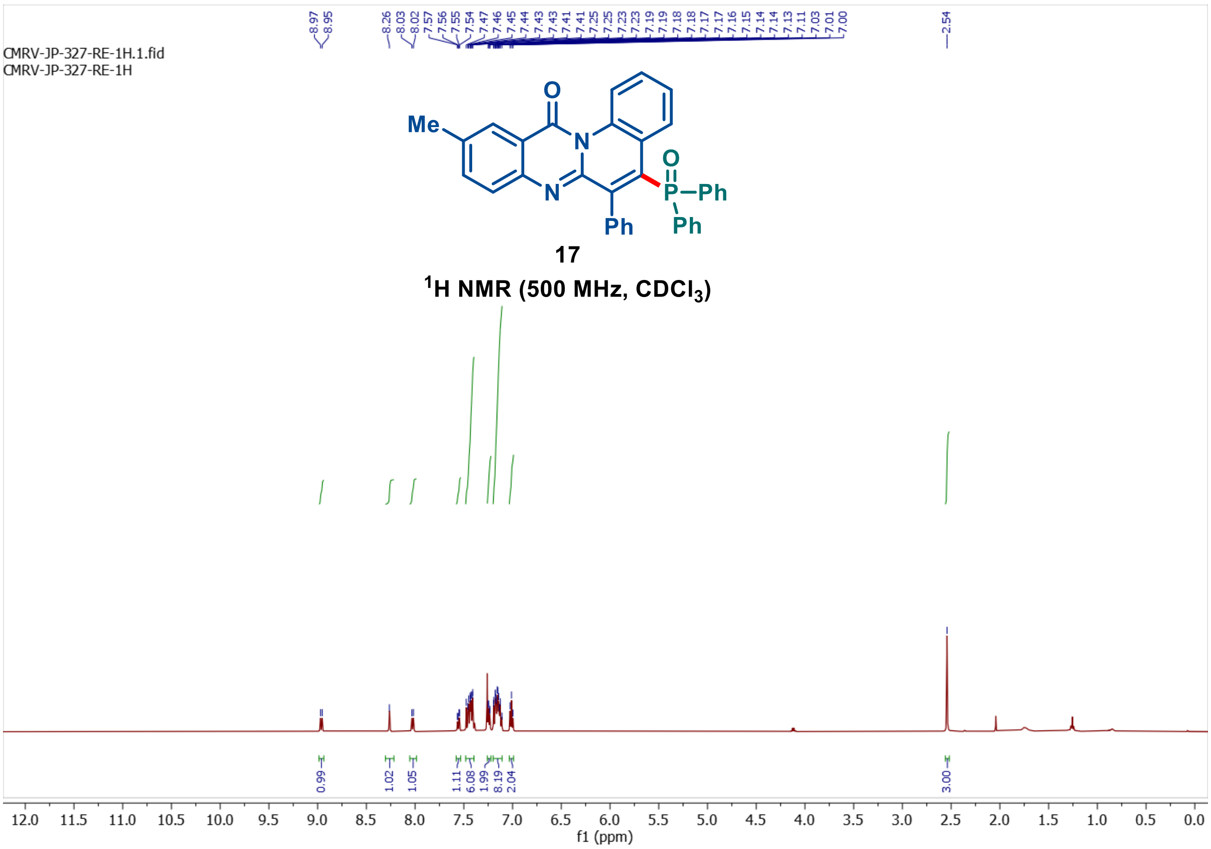
CMRV-JP-327-RE-1H.1.fid
CMRV-JP-327-RE-1H
17
1H NMR (500 MHz, CDCl3)
f1 (ppm)

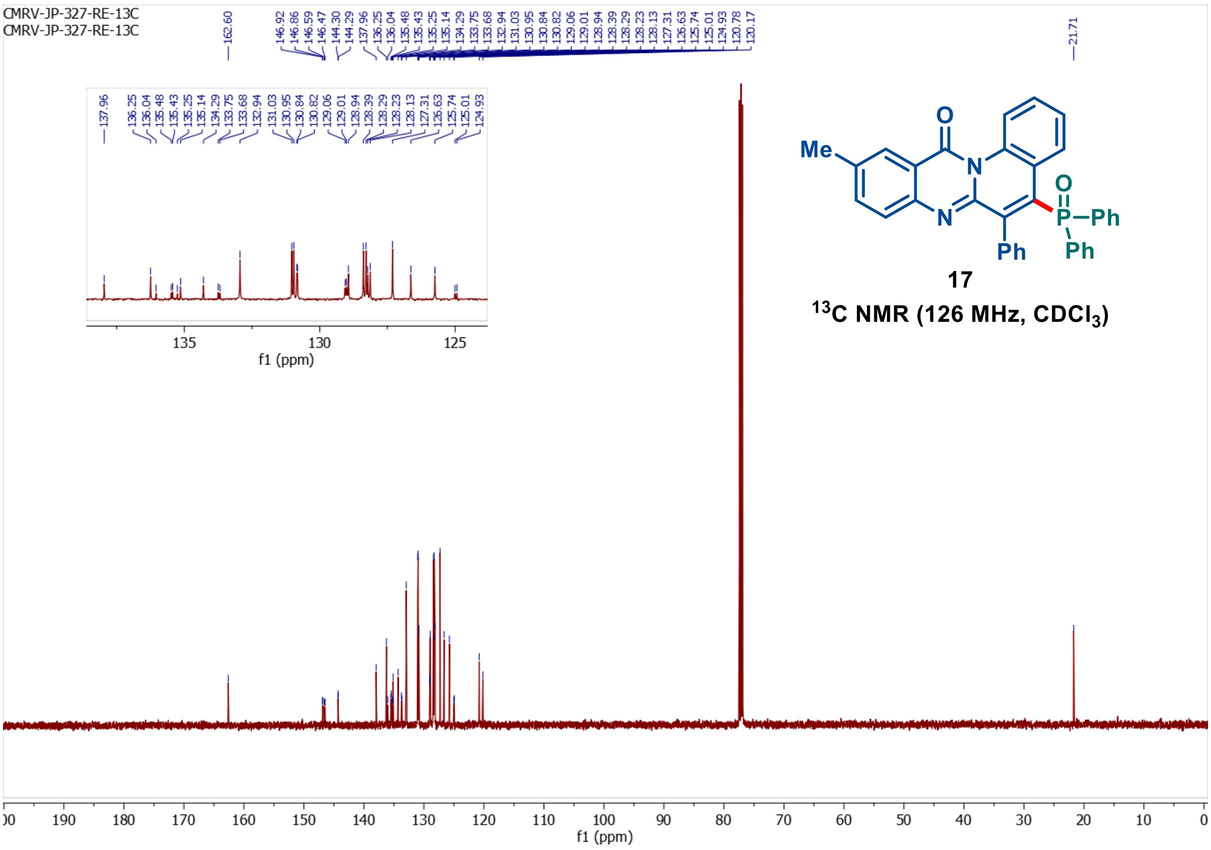
CMRV-JP-327-RE-13C
CMRV-JP-327-RE-13C
17
13C NMR (126 MHz, CDCl3)
f1 (ppm)

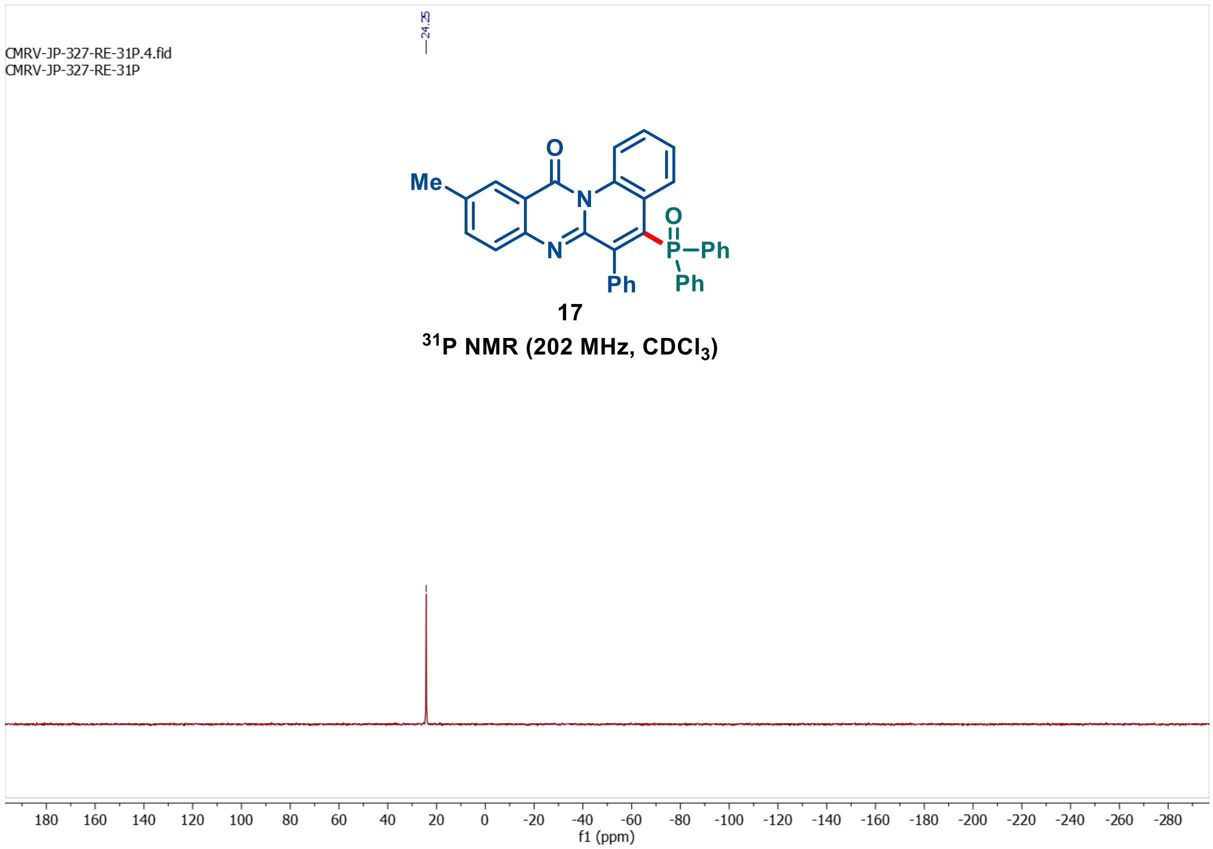
24.25
CMRV-JP-327-RE-31P.4.fid
CMRV-JP-327-RE-31P
Me
O
N
N
O
P
Ph
Ph
Ph
17
31P NMR (202 MHz, CDCl3)
180 160 140 120 100 80 60 40 20 0 -20 -40 -60 -80 -100 -120 -140 -160 -180 -200 -220 -240 -260 -280
f1 (ppm)
